# LHPE-nets: A lightweight 2D and 3D human pose estimation model with well-structural deep networks and multi-view pose sample simplification method

**DOI:** 10.1371/journal.pone.0264302

**Published:** 2022-02-23

**Authors:** Hao Wang, Ming-hui Sun, Hao Zhang, Li-yan Dong

**Affiliations:** 1 College of Computer Science and Technology, Jilin University, Changchun, China; 2 Key Laboratory of Symbolic Computation and Knowledge Engineering of Ministry of Education, Jilin University, Changchun, China; Taipei Medical University, TAIWAN

## Abstract

The cross-view 3D human pose estimation model has made significant progress, it better completed the task of human joint positioning and skeleton modeling in 3D through multi-view fusion method. The multi-view 2D pose estimation part of this model is very important, but its training cost is also very high. It uses some deep learning networks to generate heatmaps for each view. Therefore, in this article, we tested some new deep learning networks for pose estimation tasks. These deep networks include Mobilenetv2, Mobilenetv3, Efficientnetv2 and Resnet. Then, based on the performance and drawbacks of these networks, we built multiple deep learning networks with better performance. We call our network in this article LHPE-nets, which mainly includes Low-Span network and RDNS network. LHPE-nets uses a network structure with evenly distributed channels, inverted residuals, external residual blocks and a framework for processing small-resolution samples to achieve training saturation faster. And we also designed a static pose sample simplification method for 3D pose data. It implemented low-cost sample storage, and it was also convenient for models to read these samples. In the experiment, we used several recent models and two public estimation indicators. The experimental results show the superiority of this work in fast start-up and network lightweight, it is about 1-5 epochs faster than the Resnet-34 during training. And they also show the accuracy improvement of this work in estimating different joints, the estimated performance of approximately 60% of the joints is improved. Its performance in the overall human pose estimation exceeds other networks by more than 7*mm*. The experiment analyzes the network size, fast start-up and the performance in 2D and 3D pose estimation of the model in this paper in detail. Compared with other pose estimation models, its performance has also reached a higher level of application.

## Introduction

The Resnet series network [1] has already obtained mature applications in many fields. In the estimation of human pose, this Resnet series network is superior in training speed and effectiveness due to its residual network. While, the Mobilenet series network [2] uses the inverted residual to extract more refined features by expanding the dimension of the tensor. Similarly, the structure of the Efficientnetv2 [3] network is lighter. But these networks also have some disadvantages. The Resnet network has a relatively large number of parameters, while the Mobilenet and Efficientnetv2 networks are not satisfactory in terms of fast start-up. And these networks have room for improvement in pose estimation performance. In our work, we used these networks as experimental comparisons to reflect the superiority of the network designed in this paper in terms of network size and estimation performance.

Recently, 3D human pose estimation has become a very important practical task [4, 5]. The new cross-view fusion 3D human pose estimation model (CVF3D) [6] generates human movements in three-dimensional space by fusing the multi-view 2D poses [7, 8] heatmap more accurately. The multi-view fusion strategy in this model is a novel and long-acting optimization framework. Its actual performance is better (Table 4). The performance of the CVF3D model is also better than Tri-CPM [9] and AutoEnc [10]. But CVF3D model has some problems and shortcomings. It has a dynamic sample conversion and a heavy 2D pose analysis section. Regardless of start-up speed and performance of the neural network used in this section, there are deficiencies. The purpose of this article is to design a better network to replace them. We hope that this network has a smaller number of parameters, can achieve training saturation quickly, and enhance the model’s pose estimation performance. For the process of dynamic sample conversion during the training of the CVF3D model, we designed a static sample simplification method to replace it, and hope this method can reduce the training time.

We have realized a Low-span deep network with external residual layer (Low-S network) and a residual deep network based on small resolution samples (RDNS) by referring to the efforts and achievements made by the predecessors. We used three datasets in the experiment, they are FLIC [11], MPII [12] and MPI-INF-3DHP [13]. 2D and 3D human joint positioning and skeleton modeling were carried out in the experiment.

In Low-S, we found that in the analysis of pose samples, the dimensionality of the shallow neural network may determine the initial training speed of the model. Correspondingly, when the dimensions of the deep network are less, the later training of the model will be limited, it caused the ultimate failure to achieve the expected result (as shown in the experiment). The deep layer of the Resnet network usually uses multiple high-dimensional network stacks, so it can achieve satisfactory estimation results in the later stages of training, but it requires more training parameters. In order for the network to achieve a faster running speed in the initial stage, and to achieve a more satisfactory estimation result in the later training. We built a network with lower shallow dimensionality and higher deep dimensionality, and set a transition layer in the middle. Then, we set up external convolutional residuals for each layer of the network, hoping that these residual networks can accelerate the training speed of the network. The first-layer in the network uses direct-connect residuals, because this layer does not reduce the size of the feature map. The transition layer is composed of inverted residual. It is used to collect features between the shallow and deep layers. Because if we do not use this form of transition layer like the Resnet network, more parameters will be added to the network. Table 1 shows the difference in the number of parameters used in training between our network and the Resnet network. And the pose estimation comparison experiments (Fig 7) also reflect the optimization effect of our network on fast start-up and pose estimation.

**Table 1:** Comparison of Resnet-34 network and Low-S network in the number of parameters and memory usage.

network	Total params	Trainable params	Estimated Total
Resnet-18	11176512	11176512	291.39MB
Resnet-34	21284672	21284672	504.94MB
Low-S	9037312	9037312	485.04MB

In addition, we also designed an optimization method for the 3D analysis of human pose. We achieved the improvement of 3D estimation performance through a residual neural network with a small recognition domain and small resolution sample (RDNS). The difference between this network and the previous one is that it needs more training to achieve the desired estimation effect. This network with small recognition domain seems to be better to adapt to the challenges of small resolution images. The pose estimation comparison experiments (Fig 8, Tables 2 and 3) reflect the optimization effect of our network. The generation of these small resolution sample benefits from the previously mentioned sample simplification method. The static sample simplification method realizes the storage of 3D samples of multiple views before training, and omits the dynamic sample processing during training, which is conducive to optimizing the running speed of the 3D model.

**Table 2:** The JDR results of each joint on the MPI-INF-3DHP dataset. The better results in our method are written in bold. R18 = Resnet-18, R34 = Resnet-34, M2 = Mobilenetv2.

Method	R18	R34	M2	RDNS
Right hip	0.947	0.946	0.82	**0.967**
Right knee	0.529	0.544	0.568	**0.553**
Right ankle	0.363	0.432	0.497	**0.446**
Left hip	0.942	0.953	0.828	**0.964**
Left knee	0.521	0.537	0.601	0.52
Left ankle	0.416	0.435	0.512	0.394
Belly	0.914	0.953	0.87	**0.959**
Neck	0.922	0.921	0.809	0.916
Nose	0.912	0.916	0.827	0.912
Head	0.871	0.875	0.735	0.872
Left shoulder	0.792	0.835	0.598	**0.842**
Left elbow	0.592	0.623	0.404	**0.677**
Left wrist	0.476	0.503	0.254	**0.583**
Right shoulder	0.738	0.772	0.571	**0.798**
Right elbow	0.612	0.6	0.397	**0.678**
Right wrist	0.547	0.494	0.293	**0.576**

**Table 3:** The JDR (%) results of each joint on the MPII dataset. The better results in our method are written in bold.

Method	Resnet-18	Resnet-34	RDNS
Right hip	0.428	0.438	0.437
Right knee	0.535	0.539	**0.542**
Right ankle	0.727	0.727	**0.731**
Left hip	0.725	0.725	**0.731**
Left knee	0.537	0.543	**0.545**
Left ankle	0.424	0.448	0.444
Belly	0.77	0.77	**0.772**
Neck	0.924	0.928	0.926
Nose	0.928	0.931	**0.932**
Head	0.884	0.893	**0.894**
Left shoulder	0.564	0.579	0.572
Left elbow	0.676	0.693	0.687
Left wrist	0.813	0.827	**0.832**
Right shoulder	0.823	0.837	0.837
Right elbow	0.684	0.691	0.691
Right wrist	0.561	0.571	**0.585**

In this sample simplification method, we redesigned the storage mode and size of 3D data through the initial camera parameters and various coordinates. The 3D datasets usually consist of a series of video files, we need to disassemble these files into images for storage before training. The challenge here is how to reduce the resolution of frames in the projection while retaining the correlation and continuity of the joints in the 3D space. In the paper, we recalculated all 3D pose-related data to adapt to these low-resolution frames and eliminated the sample resolution conversion process during CVF3D model training. The 3D pose-related data include *box*, *center*, *scaler*, and joint positions *et* *al* of all images in each projection (Fig 5). In addition, we also eliminated some irrelevant frames in these video files. We have made more contributions in this article.

Our contributions in this article are as follows:

1. Our Low-S network achieves the fast start-up of the training and gets a better 2D pose estimation performance. In the fast start-up experiment, when the epoch gradually increases, the accuracy of the Low-S network is improved faster compared to other networks (Fig 7).
2. The static sample simplification method reduces the pressure on the operating environment of 3D pose estimation. In the latter part of Section 3.2, we calculated and estimated the degree of lightweight of the RDNS network relative to the Resnet network (Table 1).
3. The network of the RDNS is slightly better than the Resnet-34 network in terms of network scale and it performs better in multi-view 3D pose estimation. All comparative experiments in Section 4.2 show the effect of RDNS method in 3D pose estimation. And in the experiment of estimating different joints, it can also improve the estimation accuracy of most joints.

This article introduced the model of human pose estimation and the neural network used in related work. At the same time, we also introduced the advantages and disadvantages of these previous works and compare them with the work of this article. In Section 3, we introduced the main content of this article and divided them into two parts for explanation. Section 3.1 mainly introduces the realization of Low-span network; and Section 3.2 mainly introduces the realization of sample simplification method and RDNS, and further analyzes the scale of RDNS network parameters. In the experimental section, we roughly divide the experiment into 2D pose estimation experiment and 3D pose estimation experiment. In the 2D pose estimation experiment, we tested the parameter scale of the Low-span network training. At the end of the 3D pose estimation experiment, we added some comprehensive comparative experiments with different networks and models.

## Related work

In this section, we introduced some technical background related to this article. These technologies involve some different models of 3D pose estimation, and various popular deep networks. Then we introduced their characteristics and the points where the work of this article may be superior or inferior to them.

Many human pose estimation methods use single-image deep learning models [14, 15], but the single-image model is not accurate enough to predict the joint, especially for 3D pose estimation. The reasons include: joint points are blocked, blurry action, *et al*. For example, VNect [16], The VNect is a model implemented to solve the error caused by the sparse and blurred joint positions in 2D pose estimation, therefore, it adds the content of monocular 3D pose estimation and a series of continuous processing. Its backbone network is Resnet-50. Because it is a single-view estimation model, its performance is slightly worse than the multi-view estimation in this article, and the benchmark network in this article is a smaller Resnet-32 network. However, because of the single-view recognition pattern, the training cost of VNect should be lower for multi-views models. The OpenPose model [17, 18] can effectively detect the 2D pose of multiple people in the image. Its characteristic is that it can better recognize the poses of multiple people in a more complicated situation, its estimation accuracy is also higher in the single-view field, and its real-time performance is good. The Smart-VPoseNet model [19, 20] realizes the use of a single-view model to process multi-view data. The model is very inspiring. It implements a more perfect high-quality view jumping technology in the multi-view dataset, which is better than the traditional single-view model. But because of its single-view recognition mode, its performance is weaker than the multi-view model. The method proposed in this paper is based on multi-view pose estimation, and this model naturally does not have some of the problems that exist in the single-view model. However, the training cost of the multi-view model and its real-time performance are worse than that of the single-view model. In general, the 2D pose estimation [21, 22] directly determines the performance of 3D pose estimation, but using only one view will inevitably have some estimation errors. Therefore, the CVF3D model solved this problem. It uses cross-view pose analysis and heatmap fusion to greatly reduce the estimation errors of the 3D pose estimation model. The CVF3D model used a CNN-based deep learning network [23] (e.g., Resnet, Mobilenetv2 or other) to form more accurate multi-view joint composition and heatmaps. The F-RPSM method used in CVF3D model is introduced in the experimental section. This model has very good practicality. Recently, in the model of multi-view pose estimation, someone proposed a target labeling method [24–26] based on active learning, which implements a self-training process, so its characteristic is that it can greatly save the workload of manually labeling datasets. And it also helps to achieve a higher degree of automated pose estimation process. Our method does not have more features in terms of active learning, but what we have achieved is the optimization of existing neural networks.

**Fig 1:**
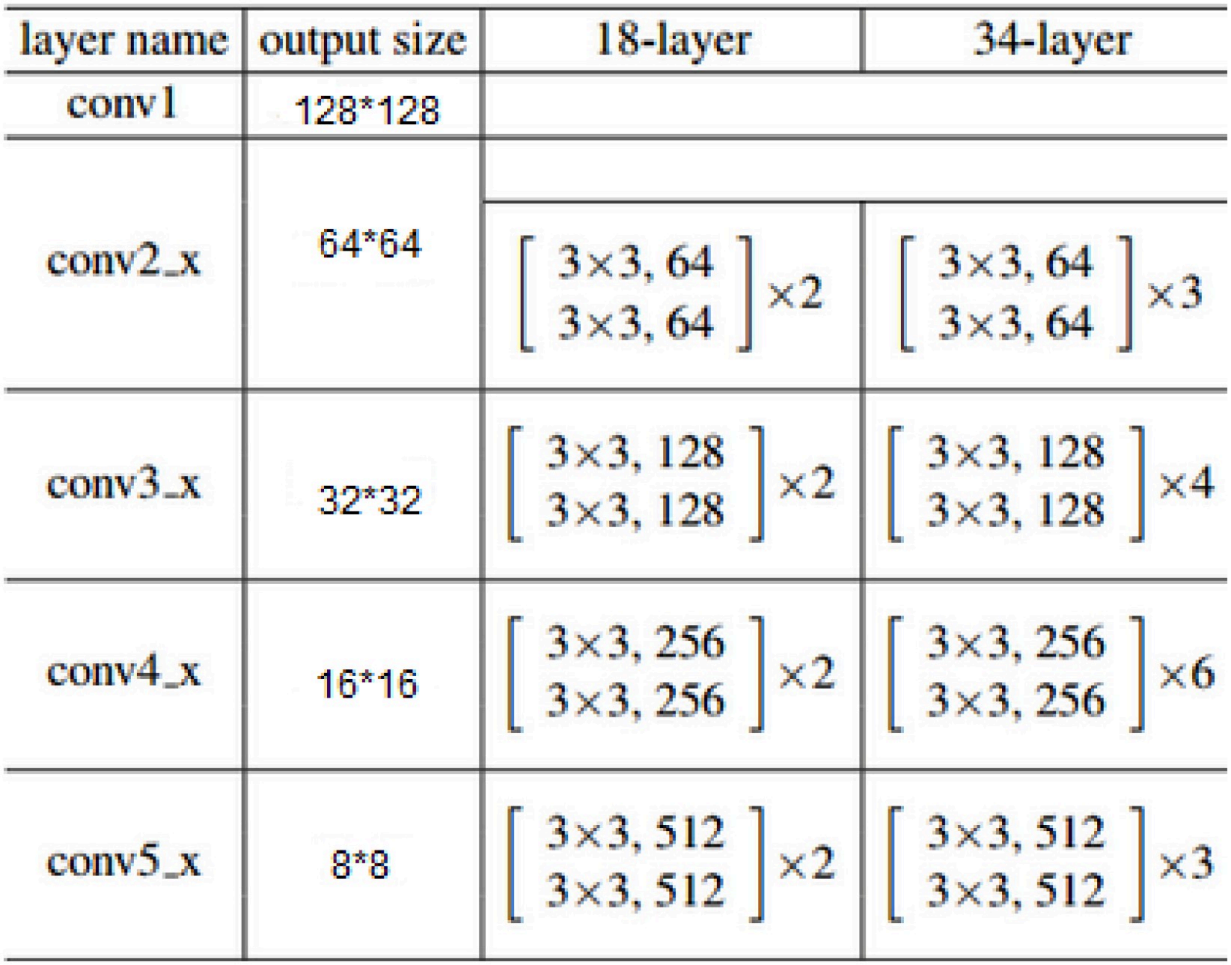
The structure of the Resnet-18 and Resnet-34 network.

In addition to the pose estimation method that generates the intermediate product (heatmap), earlier models use the joint regression scheme (e.g., DeepPose) [27] to obtain pose estimation, but these models have some problems: it is difficult to return to the *xy* coordinate position, which leads to the complexity of learning and poor generalization ability. In the pose analysis method that combines global and local information, Stacked Hourglass Networks [28] is a representative framework. The network captures information at every scale and it is excellent in pose estimating performance. The performance of the VGG network [29] in this field is also relatively good. Its network structure is simple, and the estimation accuracy can be improved by deepening the number of layers of the network, but the network consumes more computing resources. The Efficientnetv2 network uses Fused-MBConv into the search space. The published article of the network tested its classification performance on small images, and the experiment showed its superior performance. And with the size of the input image, it can adjust the regularization factor adaptively. Compared with the Mobilenetv2 network, the Mobilenetv3 network [30] has a higher training speed. However, in our experiments, the Mobilenetv3 network does not perform as well as Mobilenetv2 in pose estimation. ResNet was proposed in the past few years and won the first place in the ImageNet competition classification task because it coexists with “simple and practical”. it is widely used in detection, segmentation, recognition and other fields. The depth of the network can be set to be very large, and its effect is excellent. The structure of the Resnet-18 and Resnet-34 network (Fig 1) is helpful for introducing our algorithm. For multi-view pose analysis, it is not suitable to use ultra-large-scale deep networks. According to the characteristics of some of the networks mentioned above, the network improvement method proposed in this article combines the advantages of the network mentioned above as much as possible, and overcomes their shortcomings in the field of human pose estimation. We hope that our work can achieve the characteristics of small size network and fast saturation of training. We still use the regression training method of heatmap, instead of higher cost direct regression of human joint points.

In the experiment, we used a 3D dataset MPI-INF-3DHP. The dataset was collected and processed by researchers such as Dushyant Mehta *et al*. Its introduction is in the experiment section.

In this article, we used the Resnet-18, Resnet-34, Mobilenetv2(v3), Efficientnetv2, other networks and our methods in pose estimation. Although CVF3D model using Resnet-101 or Resnet-152 perform better (Table 4), but their scale is too large and they may be more suitable for heavier learning tasks [31].

**Table 4:** The performance of CVF3D model (with Resnet-152) compared with other models in 3D pose estimation. The metrics (MPJPE) here is introduced in the experiment section of this article. This experiment uses the H36M dataset.

Methods	PVH-TSP [34]	Wei *et al* [35]	Pavlakos *et al* [36]	Tome *et al* [37]	Zheng *et al* [38]	CVF3D
MPJPE	87.3mm	57.1mm	56.9mm	52.8mm	49.5mm	31.17mm

## LHPE-nets and static sample simplification method

**Fig 2:**
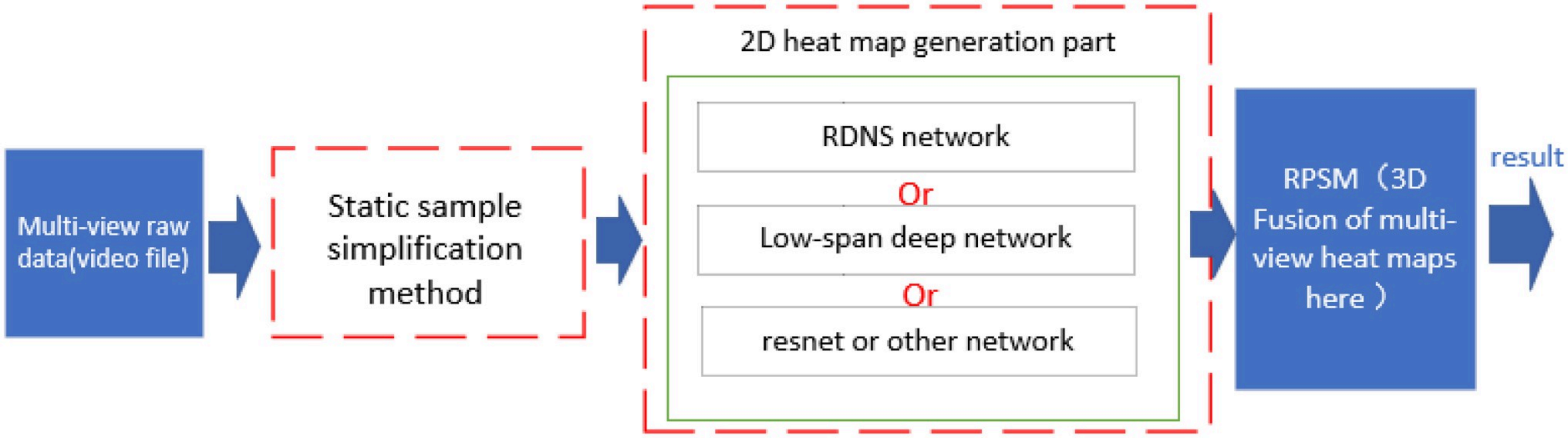
The realization and structure of our work, and its operation process.

In this section, we introduced the details of our work. They are divided into two parts. Our work is mainly in the data processing part and the 2D heatmap generation part (Fig 2). The static sample simplification method realizes the process of pre-processing the input samples, and saves the images of each pose with the required resolution. No matter how big the box where the poses in the original data are, it can always map these poses to small-resolution images. In the 2D heatmap generation part, the RDNS network and Low-span network in this article can replace other neural networks.

### Low-span deep network

This section described our first work, including Low-span deep network with external residual layer, and its two previous versions of the network. We have obtained the best-performing network by gradually adjusting the network structure in a large number of experiments. In the process, we discovered the improvement strategies needed in pose estimation applications.

**Fig 3:**
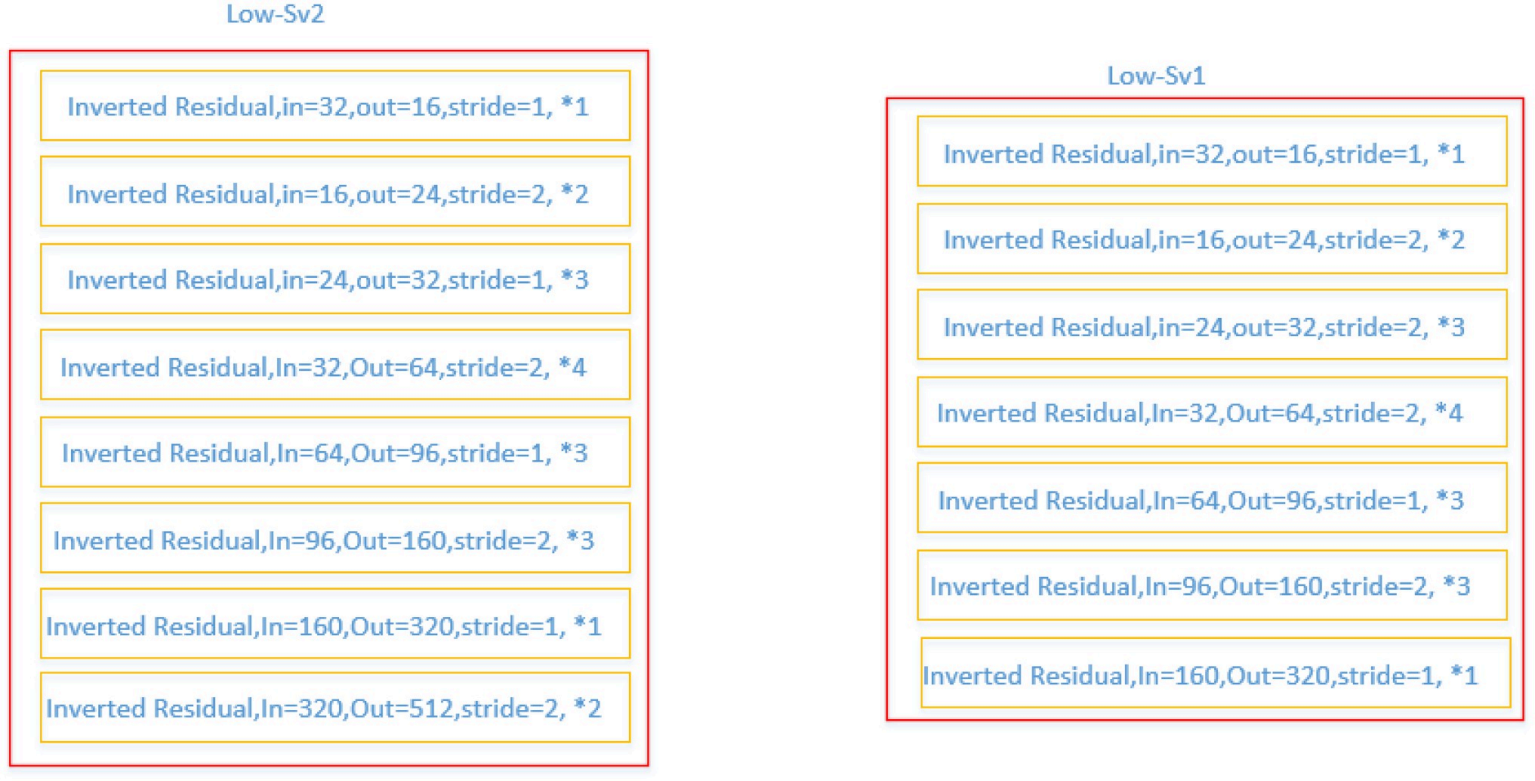
Low-Sv1 and Low-Sv2 network structure.

**Fig 4:**
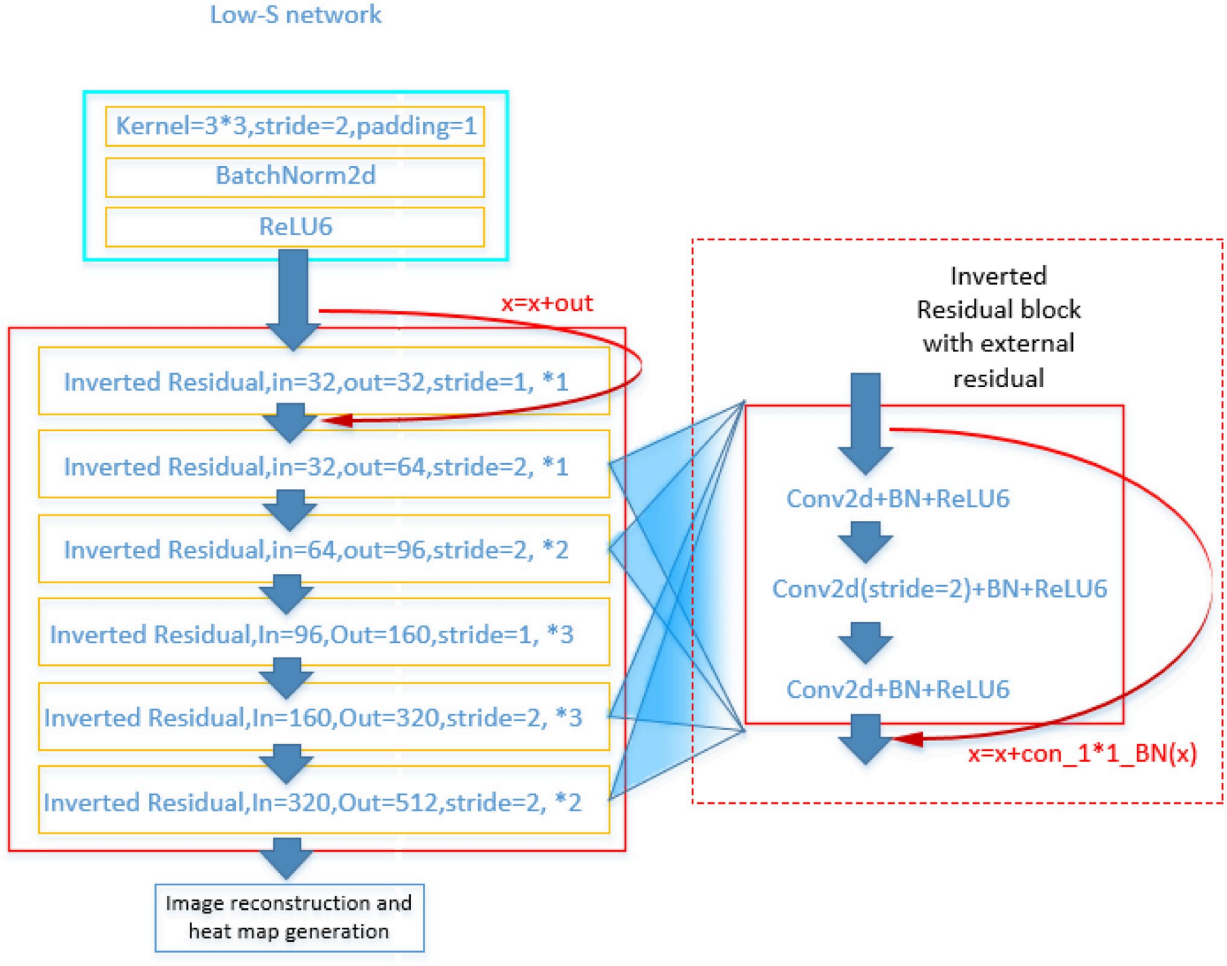
Low-S network structure. The “inverted residual block with external residual” on the right is the specific implementation inside the “inverted residual”.

We have followed the bottleneck structure of the spindle in the Mobilenet network. We think it will bring about the re-extraction of data features of the same dimension. The three network structures we used in this process are shown in Figs 3 and 4. The Low-S network (Fig 4) is the final form of the structure. And Low-Sv1 and Low-Sv2 are its previous version, their performance is not superior enough, we only draw its general structure (Fig 3). In the experimental section, we compared the performance of Low-Sv2 and Low-S to illustrate the reasonableness of the Low-S network. The Low-Sv1 network performs poorly and did not participate in the comparison.

In these three networks, we all use external convolution residual layer (Fig 4). According to our previous experience, the residual layer in the Resnet network plays an important role in the deep network. But we did not use the external convolutional residuals for the first layer network (Fig 4). Because this layer does not change the dimension and size of the feature map, it is better to use direct connection residuals for this layer.

We found in the experiment of this structural network that when we use the Low-Sv1 network, the bottleneck of each layer in Low-Sv1 involves fewer feature dimensions. Although the network can fast start-up, but after more training, it cannot achieve satisfactory results. In the pose recognition based on the heatmap, we found that when the dimension of the feature map after analysis is low, the network is not good enough to recognize the small features in the sample (because the network can fast start-up, but it reaches the training saturation prematurely.). We explained this phenomenon in the experimental section.

In addition to ensuring the fast start-up of the network, we also need to enhance the deep recognition ability of the network. In this case, we increased the output dimension of the tail of the network. But we don’t want to increase the total number of network layers. Therefore, we chose to expand the output dimensions of all layers. In order to ensure that the network can fast start-up, the shallow layer of the deep network still does not exceed 32 dimensions (the shallow network dimension of the Resnet series network starts from 64 dimensions). The tail dimension of the network after expansion is 512. We have also removed several shallow layers from the original Low-Sv1 network, because the results are still better through experiments. In the transition layer, we completely use one bottleneck in Mobilenetv2 (including the dimension expansion layer), and all the convolutional layers stride are 1. It is also reserved the size of the feature map. We believe that its structure meets the requirements of fast start-up, guarantees the training depth, and also reduces the size of the middle layer of the network as much as possible.

In our experiments, we verified its fast start-up and the effect it achieves in multiple deep networks and datasets.

### Static sample simplification method and RDNS

**Fig 5:**
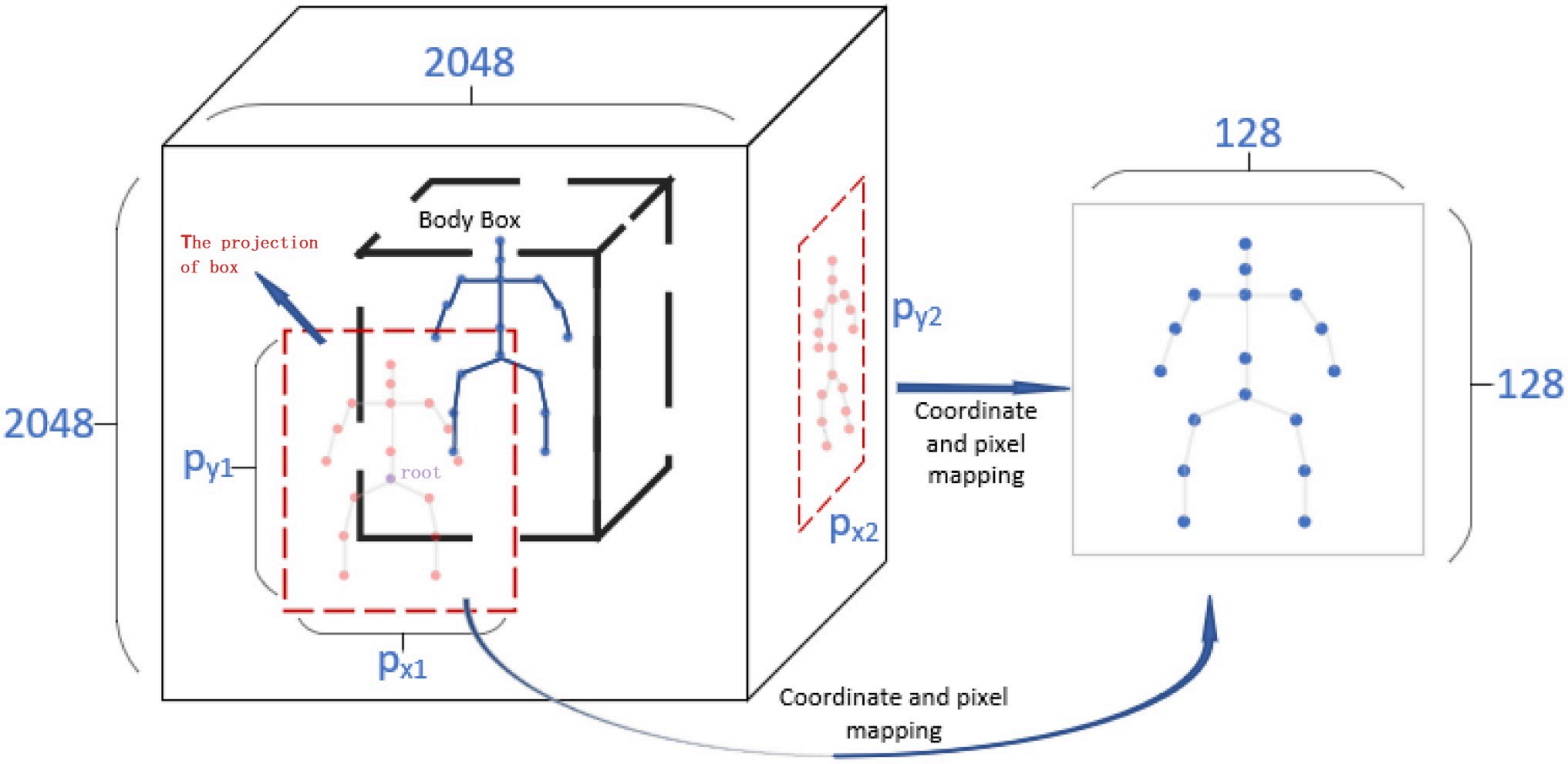
The projection of the 3D pose data in the four views is converted to four small images with a resolution of 128*128 (or 256*256) without losing their relevance and continuity.

As mentioned earlier, saving all 3D data original frames will use huge hard disk space, and it also takes time to dynamically convert the resolution of the image during training. Therefore, we chose to save the 3D data samples that have been converted frame by frame in the hard disk. The key point to implement this method is to divide the area where the all joints in the image are located. The original 3D video dataset includes the camera coordinates, pixel coordinates of these joints and camera parameters. In the projection of each view, these joints are shown in the Fig 5.

We need to get the center position of all joints in the projection of each view and the area where all the joints are located (usually people regard the root joint *P*_*root*_ or the pelvic joint as the central location), then set the left and right, up and down borders of the area to get a square area (*box*). By observing the body shape of the data collector and experimenting with box cropping, it is found that setting the pixel positions of the four borders to (-1023, 1023, -900, 1146) has the best effect (Eq (1)). The reason for this setting is that on the y-axis of the pixel coordinates, the position of the root is actually offset relative to the center point. Therefore, the value on the y-axis is not symmetrical. However, this problem does not exist on the x-axis, so the settings for them are symmetrical. The camera intrinsics are *f*_*x*_, *f*_*y*_, *c*_*x*_, *c*_*y*_. The *box* is the space area where the body is located (Fig 5), but the size and position of the area in the projection of each view are different. Therefore, the box calculation for each view is as Eqs (1)–(3).

The *box* (intermediate matrix), *center*, and *scaler* obtained are the general parameters of the projection. Through these parameters, the coordinates of three important points can be calculated: center point, upper left point (*center*_*x* − *scaler*_*x* * 200, *center*_*y* − *scaler*_*y* * 200), and center point of upper edge (*center*_*x*, *center*_*y* − *scaler*_*y* * 200). The 200 here is a uniform multiple. When the *scaler* is saved, it is divided by 200, and then it is multiplied by this multiple when it is used. *center* represents the center coordinates of the pose in the image pixels, and *scaler* represents the length and width of the box including all joints in pixel coordinates. Therefore, in the video, the pixel distance spanned by the box of each frame of image is almost different, but by affine transformation, they can be mapped to boxes of the similar size. We have calculated the pixel coordinates of the left, right, upper and lower borders of the box (Eqs (2) and (3)). And we also get the required affine matrix. The three points are the pixel coordinates of the original frame. They can be used to calculate the affine transformation matrix of different resolution images. The affine operation here is to scale the coordinates and pixels of the box in projection. The affine transformation equation (Eqs (6) and (7)) is used to calculate each parameter in the affine transformation matrix.

$$\begin{array}{c} \begin{array}{c} P{\left(x\right)}_{l}^{c}=P{\left(x\right)}_{root}^{c}-1023,P{\left(x\right)}_{r}^{c}=P{\left(x\right)}_{root}^{c}+1023,\\ P{\left(y\right)}_{l}^{c}=P{\left(y\right)}_{root}^{c}-900,P{\left(y\right)}_{r}^{c}=P{\left(y\right)}_{root}^{c}+1146, \end{array} \end{array}$$ (1)

$$\begin{array}{c} \begin{array}{c} P{\left(x\right)}_{r}=\frac{P{\left(x\right)}_{r}^{c}}{P{\left(z\right)}_{r}^{c}}f_{x}+c_{x},P{\left(x\right)}_{l}=\frac{P{\left(x\right)}_{l}^{c}}{P{\left(z\right)}_{l}^{c}}f_{x}+c_{x},\\ P{\left(y\right)}_{r}=\frac{P{\left(y\right)}_{r}^{c}}{P{\left(z\right)}_{r}^{c}}f_{y}+c_{y},P{\left(y\right)}_{l}=\frac{P{\left(y\right)}_{l}^{c}}{P{\left(z\right)}_{l}^{c}}f_{y}+c_{y}, \end{array} \end{array}$$ (2)

$$\begin{array}{c} box=(P{\left(x\right)}_{l},P{\left(x\right)}_{r},P{\left(y\right)}_{l},P{\left(y\right)}_{r}) \end{array}$$ (3)

$$\begin{array}{c} center=\left(center\_x,center\_y\right)=\left(\frac{P{\left(x\right)}_{r}+P{\left(x\right)}_{l}}{2},\frac{P{\left(y\right)}_{r}+P{\left(y\right)}_{l}}{2}\right) \end{array}$$ (4)

$$\begin{array}{c} scaler=\left(scaler\_x,scaler\_y\right)=(P{\left(x\right)}_{r}-P{\left(x\right)}_{l},P{\left(y\right)}_{r}-P{\left(y\right)}_{l}) \end{array}$$ (5)

$$\begin{array}{c} {\left[x^{\prime },y^{\prime },1\right]}^{T}=(\begin{array}{ccc} a_{1} & a_{2} & t_{x}\\ a_{3} & a_{4} & t_{y}\\ 0 & 0 & 1 \end{array}){\left[x,y,1\right]}^{T} \end{array}$$ (6)

$$\begin{array}{c} \begin{array}{c} a_{1}x_{1}+a_{2}y_{1}+t_{x}=x_{1}^{\prime },a_{3}x_{1}+a_{4}y_{1}+t_{y}=y_{1}^{\prime },\\ a_{1}x_{2}+a_{2}y_{2}+t_{x}=x_{2}^{\prime },a_{3}x_{2}+a_{4}y_{2}+t_{y}=y_{2}^{\prime },\\ a_{1}x_{3}+a_{2}y_{3}+t_{x}=x_{3}^{\prime },a_{3}x_{3}+a_{4}y_{3}+t_{y}=y_{3}^{\prime }, \end{array} \end{array}$$ (7)

It (Eq (7)) is obtained by the three important point coordinates of the original image and the target image. The *a*_*i*_(*i* = 1, 2, 3 and 4) and *t* (Eq (7)) can be calculated. In general, solving six unknown parameters requires six uncorrelated equations (Eq (7)). However, in this article, some special value methods can be used. For example using input data:(0, 0), (0, *y*_*center*), (*x*_*center*, 0), such special input data can be obtained by operations such as translation. Substitute this special value into Eq (7), which will give you an easily solved system of equations. However, in the program, it should be the solution method of the matrix operation. For the key pixel area of the original image (*box*) and all joint positions, their transformed position can be obtained by Eq (6). The converted box always maintains a relatively fixed size in the target image (Fig 5).

In the MPI dataset, this operation converts the original 2048*2048 resolution to 256*256 or 128*128. Mapping poses into small graphs has two advantages. Firstly, the irrelevant information in the background is well eliminated, and only the target and surrounding pixels are retained. Second, as mentioned before, this reduces the space for storing samples. In practice, storing a 2048*2048 resolution original image uses approximately 220KB of space, while storing a 128*128 resolution image only uses approximately 4KB of space.

If the action executor is too close to the camera or outside the sampling range of the camera, the invalid part will be filled with a black background. This is an inevitable situation. Under normal circumstances, when lots of images are required, the resolution of the sample generally does not exceed 320*320. With the previous work, because the Resnet series of networks perform better in 3D pose estimation, we hope to adjust some parts of the network to better adapt to small-resolution samples (128*128) and get better estimation results. We call this network RDNS.

**Fig 6:**
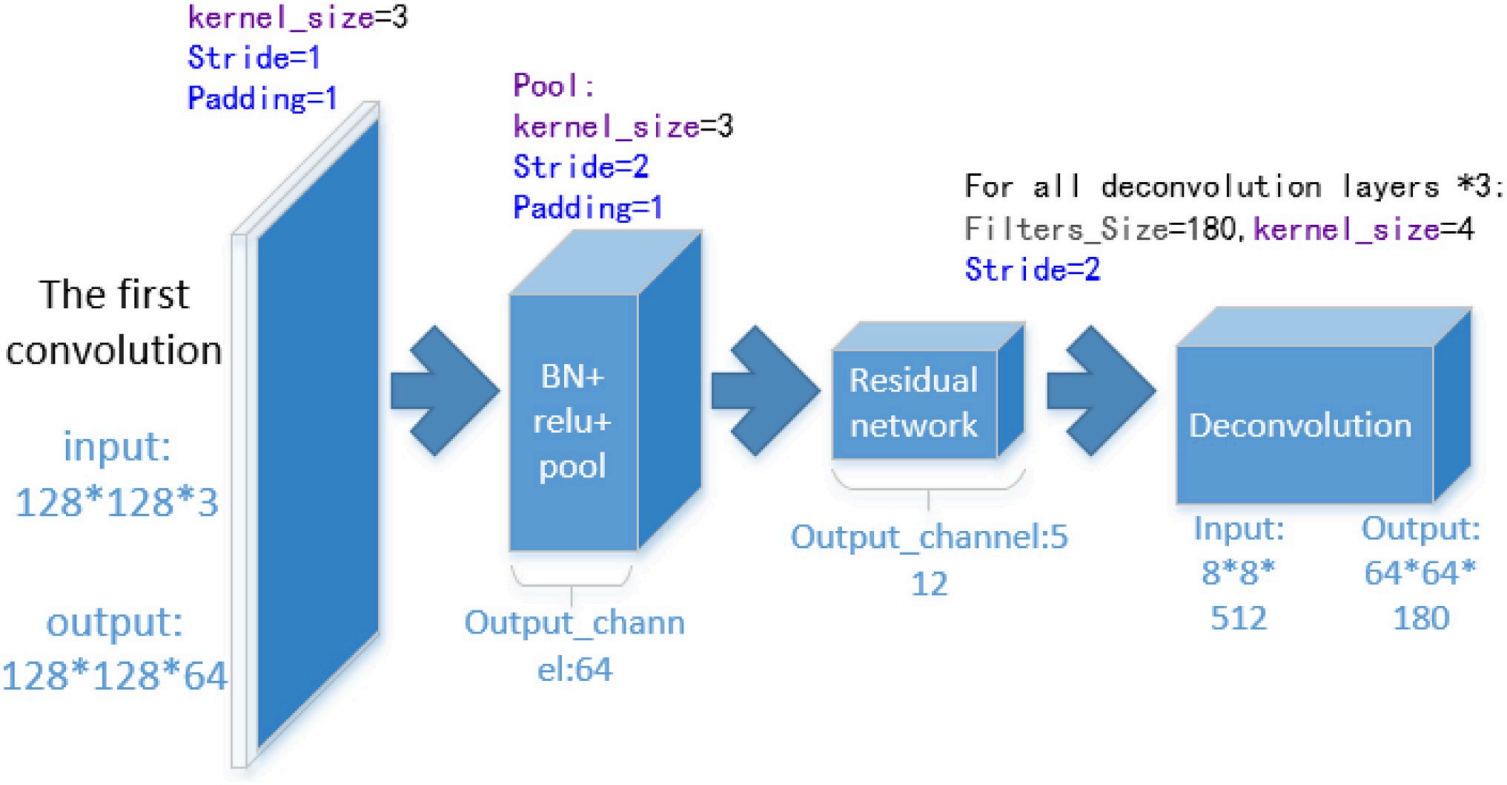
The structure of the RDNS deep network.

In the CVF3D, there is a four-way deep learning network. It is composed of deep learning networks such as Resnet-34 and Resnet-18. When we use the low-resolution sample, we adjust this part (Fig 6), and we take the Resnet-34 network as an example to explain. In order to ensure the effectiveness of the 2D pose estimation network, we retained the residual part of this network. The first convolution layer of the Resnet is very important. It guarantees the quality of feature map for each layer network. If we assume that the number of features (useful features and useless features) are proportional to the number of pixels of the image, we will not need a larger perception field for the first layer. Therefore, we set the convolution kernel size of the layer to 3 (this setting has better nonlinear expression ability and more suitable for processing small images), reduce stride to 1, and reduce the padding of the layer, so as to retain more useful information. The layer condenses the features while keeping the output size of the layer. The CNN output formula is *output*_*size* = (*W* − *F* + 2*P*)/*S* + 1. Here, *W* is the size of the upper layer output, *F* is the size of the current layer convolution kernel, *P* is the pixel width of padding, and *S* is the stride. In general, the formula for the parameters used in a certain layer of CNN is as follows, *parameter*_*amount* = *input* + *network*_*parameter*_*amount* + *output*. The input and output here are the size of the image, they are generally the *channel* * *image*_*size*^2^. The number of network parameters is generally *network*_*parameter*_*amount* = *kernel*_*size*^2^ * *input*_*channel* * *output*_*channel*. The output of this layer is *output*_*size* = (256 + 2 * 3 − 7)/2 + 1 = (128 + 2 * 1 − 3) + 1. The left and right terms in the formula are the same in the CNN output. The first term of the formula is the first convolution layer output of the original model, and the latter term is the first layer output of the improved network. The theoretical memory consumption level of this layer (input + the convolution layer parameters): 256^2^ * 3 + 7^2^ * 3 * 64 (original model); 128^2^ * 3 + 3^3^ * 64(Ours).

The high-density feature map of the first-layer convolutional network gives the residual layer network a more saturated feature map and makes the network learn these images faster as the experiment.

In addition to the first convolution layer, at the end of the 2D pose estimation network, three deconvolution layers recover and fill the feature map. Compared with the original Resnet-34, these deconvolution layers do not need to expand the feature map channel like the original network. Therefore, we reduced the filters of these deconvolution layers. If we use the previous assumption that the feature information of the image is proportional to its resolution. When using a 256*256 resolution image, the stride of the first convolution layer is 2, it can be considered that the feature sampled is: 256*256/2. And when we used a low-resolution image and the stride was set to 1, the feature sampled is 128*128. Therefore, it may be more appropriate to use 128 filters in these deconvolution layers. But we found in experiments that when the filters are 128, the performance of the model is not satisfactory. And on the other hand, the filters cannot be greater than 256, because some filters will become redundant for low-resolution images. Actually, in this part, we should consider the characteristics of the output of the residual layer more than the characteristics of the sample. Therefore, we found in the experiment that when the filters are set to about 180, the effect of the network is better. Then, the video memory usage will be reduced here, sometimes, the batch of this network can be set larger. The theoretical memory consumption level of the three deconvolution layers (the deconvolution layer parameters + output): the original model: 512 * 4^2^ * 256 + 256^2^ * 4^2^ * 2 + (16^2^ + 32^2^ + 64^2^) * 256; Ours: 512 * 4^2^ * 180 + 180^2^ * 4^2^ * 2 + (16^2^ + 32_2_ + 64_2_) * 180.

## Experiment

In this section, we showed the effect of the CVF3D model (Table 4) (Using large-scale learning networks and Fusion Recursive Pictorial Structure 3D Model). Then, we compared LHPE-nets with the previously mentioned deep learning network and the CVF3D model in 2D and 3D pose estimation. At last, the paper used our model and other mainstream models to perform comparison experiments.

The data we used in the experiment includes two 2D pose datasets (FLIC, MPII) and a 3D pose dataset (MPI-INF-3DHP). The information of these datasets is as follows.

**FLIC**: The images in this dataset are intercepted from some popular movies. The images were obtained by running a state-of-the-art person detector on every tenth frame of 30 movies. The dataset includes 5003 human pose images. We use the first 3987 images for training, and use the last 1016 images for testing. The pose of this dataset includes 30 joints annotated, but most of these joints are not used, so we only leave the 13 more important joint positions for experiment. The resolution of all images is 720 * 480.(S1–S4 Datasets).

**MPII**: MPII human pose dataset is a state-of-the-art benchmark for estimation of articulated human pose estimation. The dataset includes around 25000 images. The human pose of the dataset includes 16 joints annotated. There are 7247 human poses for testing, and another 22246 human poses for training. Some images include multiple human poses.

**MPI-INF-3DHP**: The 3D dataset includes a large number of continuous action frames taken by eight cameras from eight angles. The actions of the dataset are completed by eight people. Therefore, the dataset has eight subjects (S1-S8), and each subject includes two sequences (Seq1 and Seq2), the actions in Seq1 are: Walking/Standing, Exercise, Sitting (1), and Crouch/Reach, the actions in Seq2 are: On the Floor, Sports, Sitting (2), and Miscellaneous. There are eight *avi* files in each sequence. They were taken by eight cameras at different angles. Each action in *avi* file is about one minute. We have deleted the interval frames between these actions. And choosing the *avi* videos taken by four cameras with better angle as the dataset. We use S1, S2, S3, S4, S5, S6 for training, and S7, S8 for testing. Moreover, we use S1, S3, S5, and S7 in the experiment of 3D pose prediction, and use all subject in the experiment of 2D pose prediction. The frame rates of these *avi* videos are 25*frames*/*s* and 50*frames*/*s*. In the experiment, we used 1/10 of the total number of frames for training, and 1/64 of the total number of frames for testing.

### Metrics and 3D model

#### Metrics

The 2D pose estimation is measured by Joint Position Detection Rate (JDR) and Accuracy. Their estimation criteria are uniformly set in the experiment.

For 3D pose estimation, we use the mean error per joint position (MPJPE) (Eq (8)).

$$\begin{array}{c} MPJPE=\frac{1}{M}\sum_{i=1}^{M}\|p_{i}^{grou}-p_{i}^{esti}\| \end{array}$$ (8)

*p*^*i*^ is the position of the joint *i*. *M* is the number of joints. *p*^*grou*^ is the groundtruth location, *p*^*esti*^ is the predicted 3D pose location.

#### Fusion Recursive Pictorial Structure 3D Model

The RPSM model is used in 3D pose estimation. It mentioned in the CVF3D paper that RPSM’s 3D estimation method performs better. The PSM model [32] causes huge quantization errors due to space discretization (Larger *N*). In PSM, you need to define the edge length *N* of a 3D space grid. The author of CVF3D defines the joint position through a multiple stage process, and uses a smaller *N*. In first stage, applying the PSM method to get an initial 3D pose grid, which makes *N* = 16. In the later stage, the area where the bone is located is further divided into smaller grids, the edge length *N* of this small grid is 2. Therefore, it is more convenient to calculate the location of every joint in the grids. This is a faster method.

### 2D pose estimation experiment

**Fig 7:**
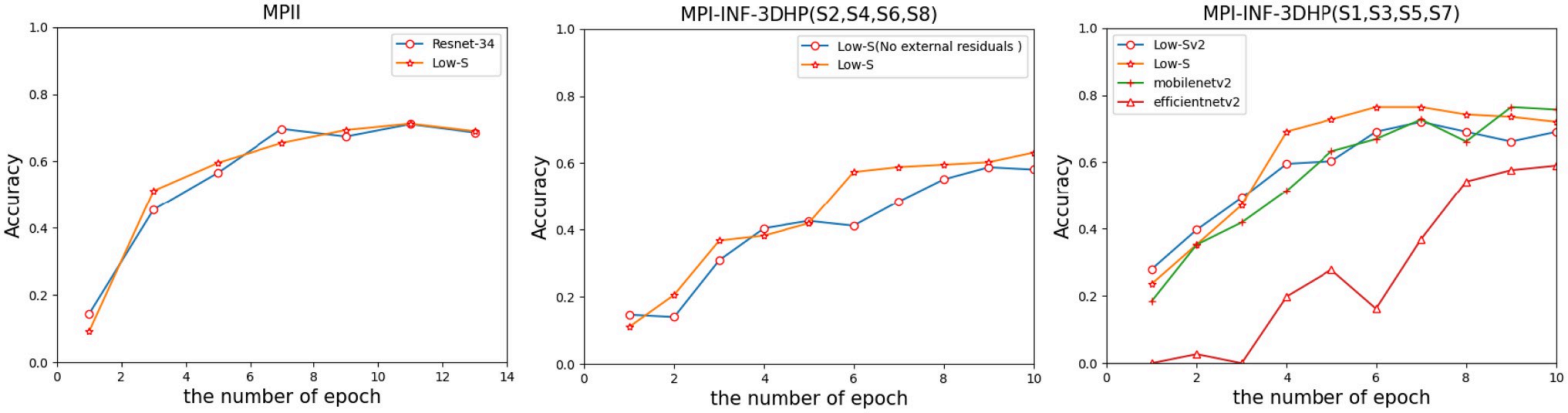
Comparative experiment results of Low-S, Low-Sv1, Low-Sv2 and other deep networks.

In this section, we used the results of Resnet, Mobilenetv2 and other networks as the comparison baseline for the experiment. This experiment uses all datasets. In each of the following comparison experiments, the batch settings are the same. The results under different epochs were shown in Fig 7. In our experiments, we compared Low-Sv2, Low-S and Low-S networks without external residuals. It shows the rationality of the Low-S network structure.

We found that the fast start-up of Low-Sv2 is better than Low-S (Fig 7), because Low-Sv2 has more shallow networks than Low-S. However, the performance of the Low-S network is better in the later stage, because too many shallow networks in Low-Sv2 cannot extract more sample details. And the performance of the Low-S network is stronger than that of the Low-S network without the external residual layer, which shows the effectiveness of the external residual. In addition, Low-S network achieves the highest performance faster than Mobilenetv2 and Efficientnetv2 network. Its performance is close to Resnet-34, but in the experiment, the parameters of Low-S network are smaller than the Resnet-34 network (Table 1).

In actual experiments, we found that the Resnet-34 network performed better. Therefore, it is more representative to use it for comparison. In the estimated memory usage, the advantage of Low-S network is not obvious (Table 1). This shows that perhaps the advantages of the Low-S network in size can be highlighted only when the hardware conditions are good.

**Fig 8:**
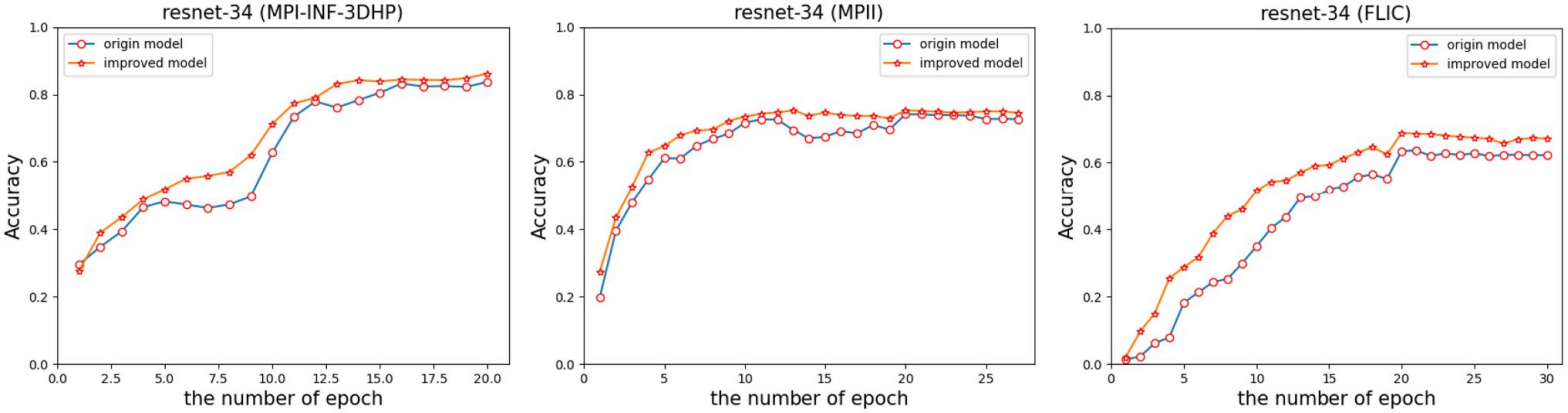
Comparative experiment results between RDNS network (the network is represented as an improved model) and Resnet-34 network.

In the 2D performance of the RDNS network, we experimented with its performance on the MPI-INF-3DHP, FLIC and MPII datasets, and we used the original Resnet-34 network for comparison (Fig 8).

The experiment (Fig 8) shows that when the RDNS network is trained more times, the RDNS network performs better in the later stage. And when the training epoch is the same, the RDNS network can always achieve better estimation performance faster. In addition to the overall joint estimated performance, we should also further explore the estimated performance of the RDNS network for all different joints (Tables 2 and 3). The estimation metric is JDR [33].

### 3D pose estimation experiment

In the paper that proposed the CVF3D model, it (with Resnet-152) performs is quite well compared to the latest model (Table 4). This shows that CVF3D model is available in our article.

In this section, we used our RDNS method to complete the 3D estimation performance on the MPI-INF-3DHP dataset. We conducted experiments on different actions in the MPI-INF-3DHP dataset. Before 3D pose estimation, we chose a set of heatmaps that performed best in the multi-view analysis as the test set. In this experiment, the fusion RPSM 3D estimation model was used. The parameter settings of the model remain unchanged. In the first group (Table 5), no action is distinguished. In the second group (Table 7), we used MPI-INF-3DHP datasets and Resnet networks to experiment on different actions.

**Table 5:** The performance of our method in 3D pose estimation. The data in the table is the mean error per joint position (MPJPE). The better results in our method are written in bold. No action is distinguished in the table. The unit of MPJPE is *mm*.

Method	Resnet-18	Resnet-34	Mobilenetv2	RDNS method
Origin model	125.26	120.17	175.74	**112.36**

We find that in addition to the Mobilenetv2 model, the RDNS performs better in 3D pose estimation (Table 5). Of course, the trends of 3D pose estimation performance and 2D pose estimation performance are similar. Moreover, the performance of the RDNS network has reached a high level no matter in the comparison of the network of the same scale or different models (Tables 5 and 6).

**Table 6:** Comparison of our method and multiple mainstream 3D pose estimation models in MPI-INF-3DHP. The better results are written in bold, they include multi-view models.

Method	Mehta *et al*	VNect	Multi Person [39]	Zhou *et al* [40]	Kanazawa	CVF3D	Wei *et al*	Biswas *et al*	Ours
MPJPE	117.6	124.7	122.2	137.1	113.2	120.17	117.2	120.17	**112.36**

Table 7 shows that in complex actions such as Exercise, Sitting (1), and On the floor, the Resnet model performs poorly. But our method can optimize the performance in these activities. We have bolded the part of the RDNS network that is better than the R34 network. It includes a total of five actions. Of course, it has 6 actions that perform better than R18 network.

**Table 7:** The 3D estimation performance of our method in different actions. The data in the table is the mean error per joint position (MPJPE). The better results in our method are written in bold. R18 = Resnet-18, R34 = Resnet-34, W/S = Walking/Standing, C/R = Crouch/Reach. The unit of MPJPE is *mm*.

Method	R18	R34	RDNS method
W/S	95.9	97.63	**81.69**
Exercise	181.5	197.88	**168.79**
Sitting (1)	181.26	210.65	**202.62**
C/R	104.76	138.5	**111.23**
On the Floor	186.25	161.18	166.54
Sports	70.42	58.75	65.78
Sitting (2)	103.24	86.27	86.53
Miscellaneous	93.1	94.55	**79.55**

Finally, we compared our model with other mainstream models for 3D pose estimation experiments (Table 6). The Biswas *et al* [41] used H36M and MPI-INF-3DHP datasets during training. Under the premise of reducing training costs, our model outperforms recent algorithms in 3D pose estimation. Our model has reached an applicable level. In actual experiments, although our neural network takes up less memory by the measurement, its actual lightweight performance is very weak. Therefore, we may further learn from the available lightweight methods in the future, for example, the lightweight neural network [42] that has been proposed before. Of course, from another point of view, enhancing the generalization ability of neural networks can also achieve the design of low-cost neural networks. Using a complex-valued neural network [43] may be able to solve such problems. In addition, for this multi-view pose estimation framework, the use of multi-view datasets means that they can be trained in parallel. Therefore, for a training environment with better hardware conditions, distributed federated learning [44] may be a better acceleration method. The detailed information extraction network mentioned in our paper can also be applied to other image retrieval fields, for example, image retrieval based on spectral domain information [45]. Because in the image search based on heuristics, for certain fixed image features, our network may have a relatively high application value.

## Conclusions

Judging from the performance of the experiment, we think that these networks are more practical. It meets our requirements for fast start-up and deep learning. Our low-resolution recognition network has also obtained satisfactory results in test, and its performance has reached the current application level. In addition, the separation of the sample processing method and training process in this article also increases the flexibility of the model.

Nevertheless, our model still has some tricky problems in training. We use the network parameter estimation function of the torch framework to measure the estimated value of the video memory occupation of the network in this paper. But in fact, in our experimental tests, the video memory occupied by the network in this article is not smaller than other networks. This also shows that although our network pays more attention to the details of the image, it also presents a greater challenge for the lightweight of the network.

## Supporting information

S1 Dataset The first part of the FLIC dataset image. (ZIP)

S2 Dataset The second part of the FLIC dataset image. (ZIP)

S3 Dataset The third part of the FLIC dataset image. (ZIP)

S4 Dataset The fourth part of the FLIC dataset image. It also includes pose data and camera parameters.
(ZIP)
