# Supplementary figures and images for: LHPE-nets: A lightweight 2D and 3D human pose estimation model with well-structural deep networks and multi-view pose sample simplification method (part 2 of 8)
Source: PLoS One. 2022 Feb 23;17(2):e0264302. doi: 10.1371/journal.pone.0264302 (PMC8865690; doi:10.1371/journal.pone.0264302)

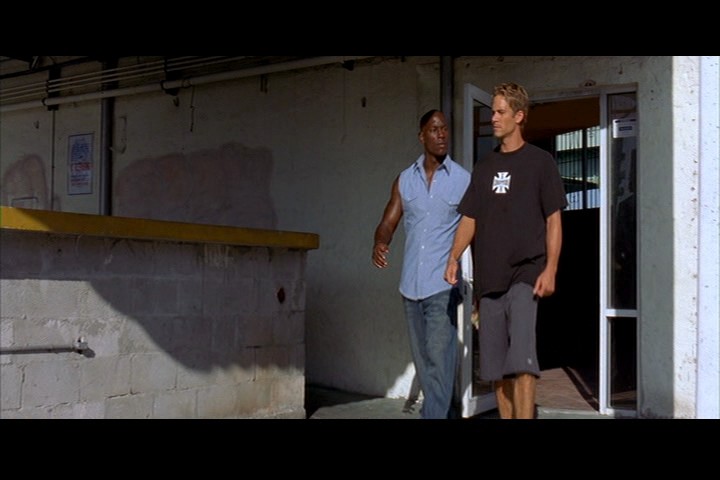

Supplement: S1 Dataset — (ZIP) [file pone.0264302.s001.zip › 2-fast-2-furious-00067291.jpg]

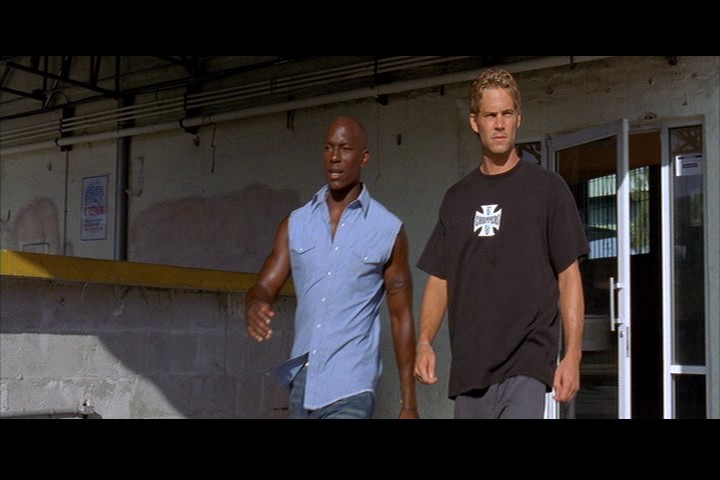

Supplement: S1 Dataset — (ZIP) [file pone.0264302.s001.zip › 2-fast-2-furious-00067321.jpg]

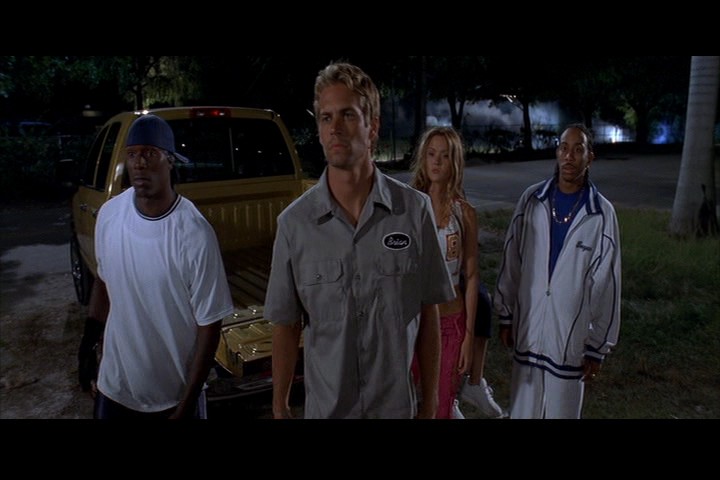

Supplement: S1 Dataset — (ZIP) [file pone.0264302.s001.zip › 2-fast-2-furious-00068731.jpg]

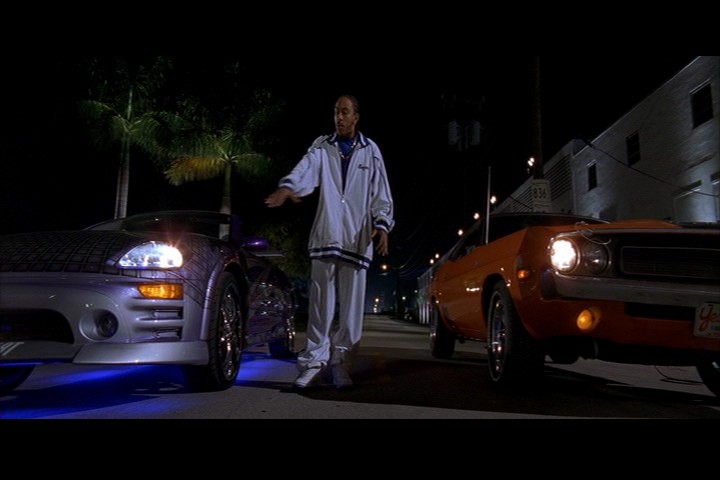

Supplement: S1 Dataset — (ZIP) [file pone.0264302.s001.zip › 2-fast-2-furious-00070611.jpg]

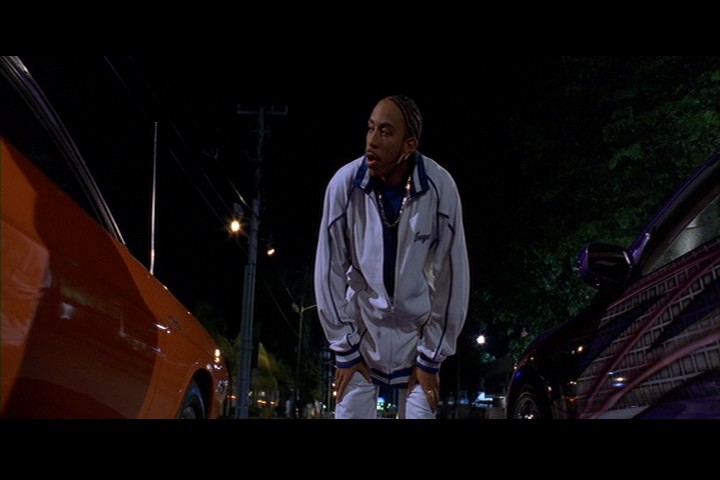

Supplement: S1 Dataset — (ZIP) [file pone.0264302.s001.zip › 2-fast-2-furious-00070851.jpg]

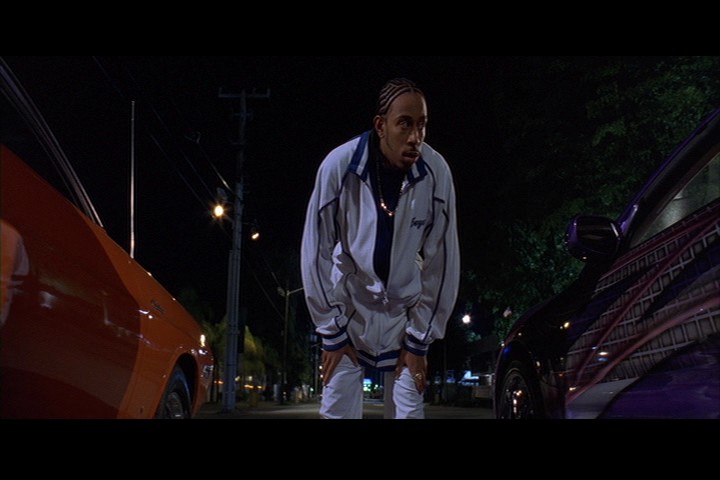

Supplement: S1 Dataset — (ZIP) [file pone.0264302.s001.zip › 2-fast-2-furious-00071201.jpg]

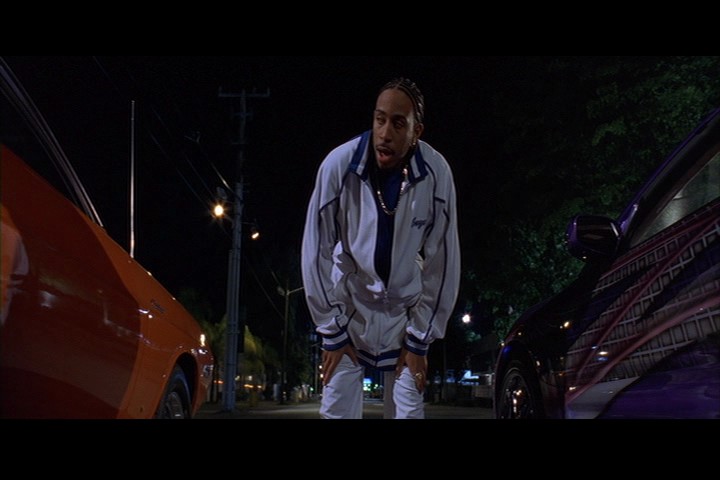

Supplement: S1 Dataset — (ZIP) [file pone.0264302.s001.zip › 2-fast-2-furious-00071211.jpg]

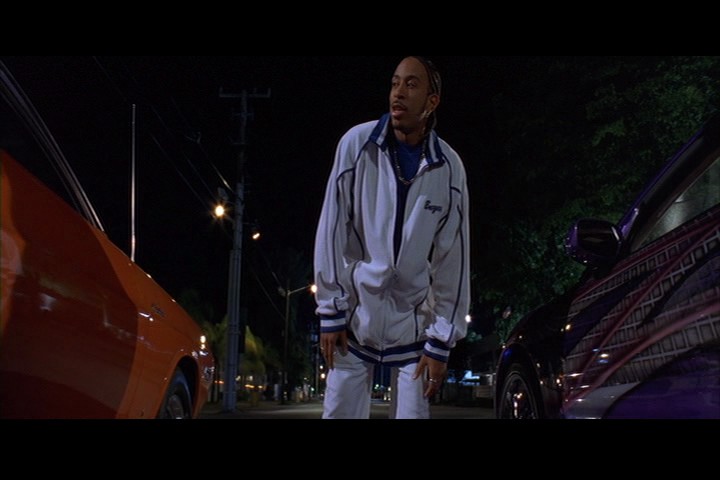

Supplement: S1 Dataset — (ZIP) [file pone.0264302.s001.zip › 2-fast-2-furious-00071241.jpg]

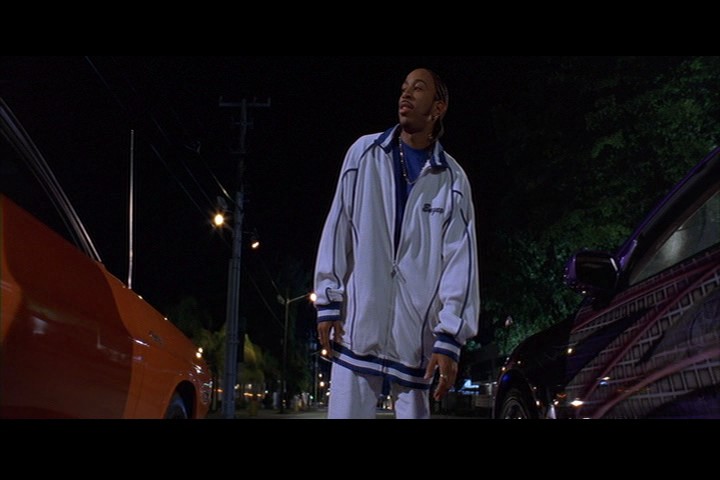

Supplement: S1 Dataset — (ZIP) [file pone.0264302.s001.zip › 2-fast-2-furious-00071251.jpg]

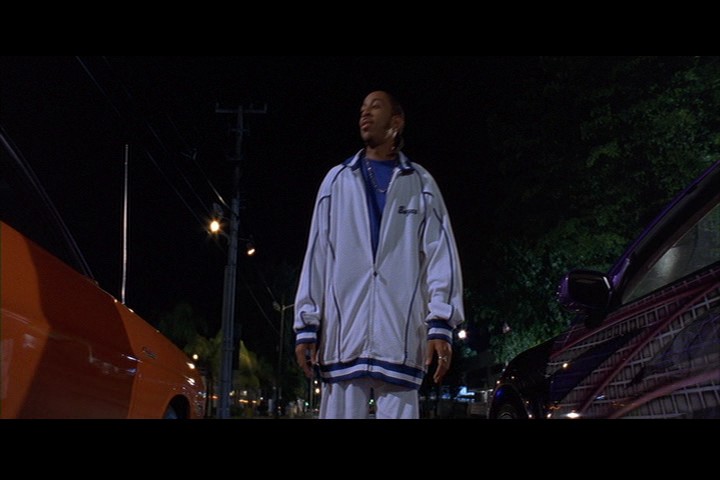

Supplement: S1 Dataset — (ZIP) [file pone.0264302.s001.zip › 2-fast-2-furious-00071261.jpg]

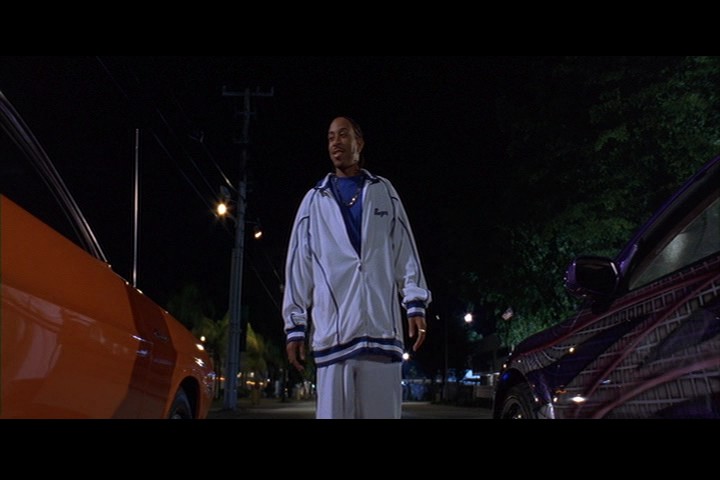

Supplement: S1 Dataset — (ZIP) [file pone.0264302.s001.zip › 2-fast-2-furious-00071281.jpg]

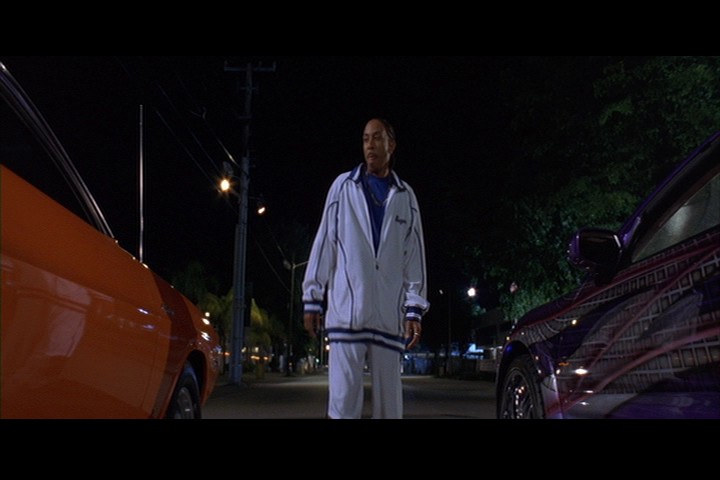

Supplement: S1 Dataset — (ZIP) [file pone.0264302.s001.zip › 2-fast-2-furious-00071291.jpg]

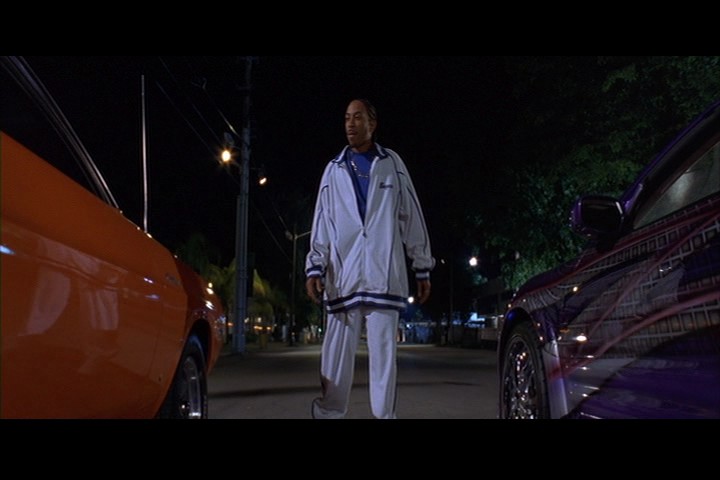

Supplement: S1 Dataset — (ZIP) [file pone.0264302.s001.zip › 2-fast-2-furious-00071301.jpg]

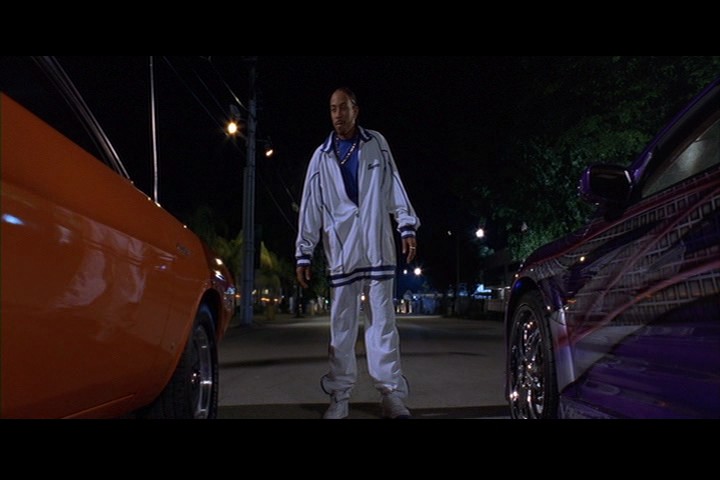

Supplement: S1 Dataset — (ZIP) [file pone.0264302.s001.zip › 2-fast-2-furious-00071311.jpg]

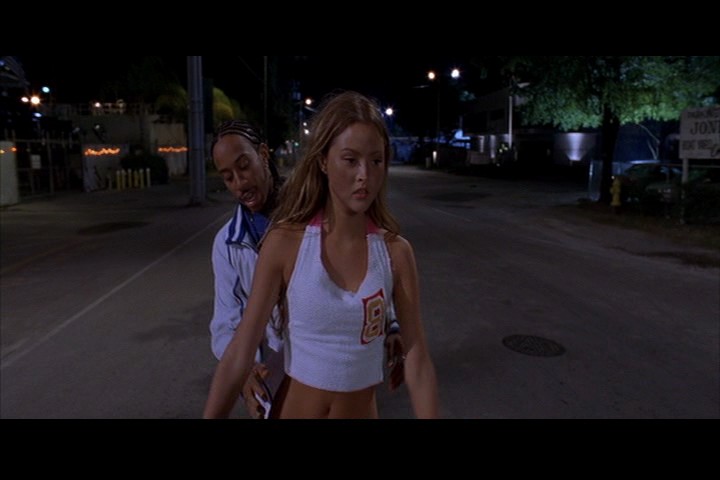

Supplement: S1 Dataset — (ZIP) [file pone.0264302.s001.zip › 2-fast-2-furious-00072471.jpg]

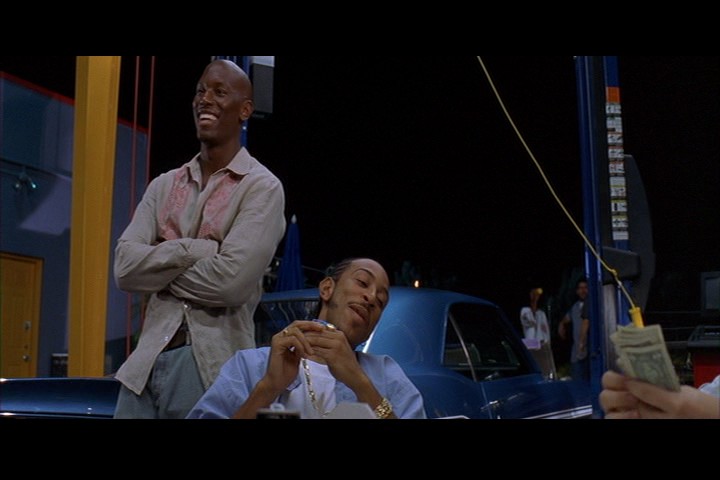

Supplement: S1 Dataset — (ZIP) [file pone.0264302.s001.zip › 2-fast-2-furious-00093061.jpg]

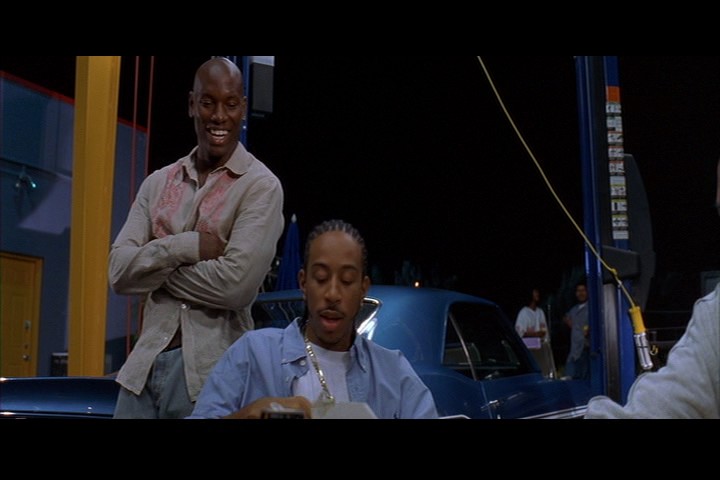

Supplement: S1 Dataset — (ZIP) [file pone.0264302.s001.zip › 2-fast-2-furious-00093171.jpg]

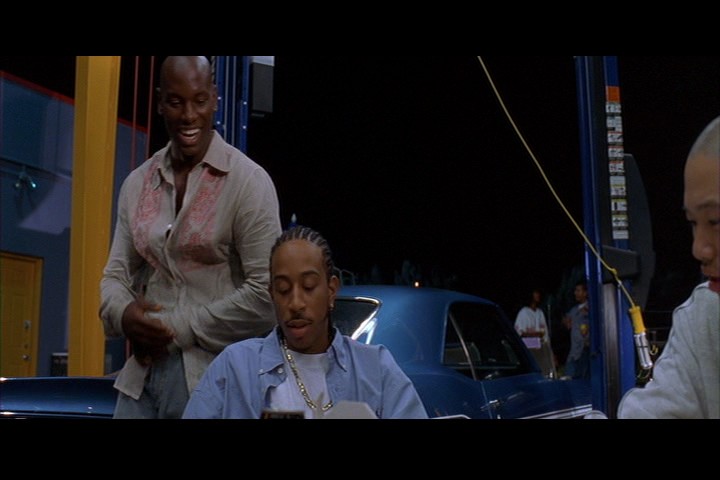

Supplement: S1 Dataset — (ZIP) [file pone.0264302.s001.zip › 2-fast-2-furious-00093181.jpg]

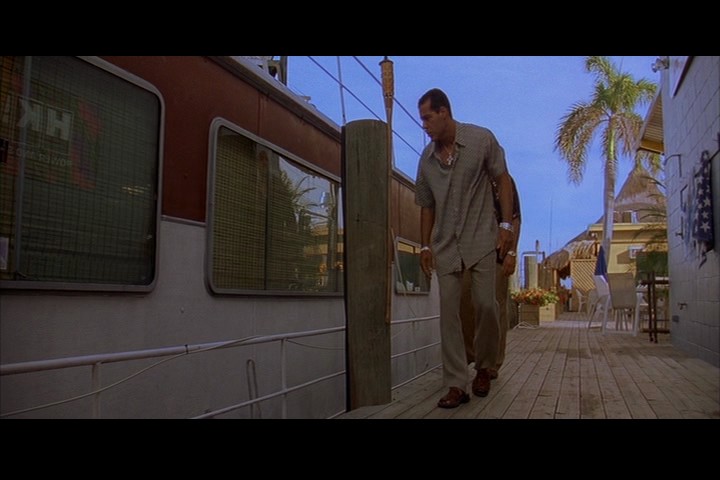

Supplement: S1 Dataset — (ZIP) [file pone.0264302.s001.zip › 2-fast-2-furious-00094941.jpg]

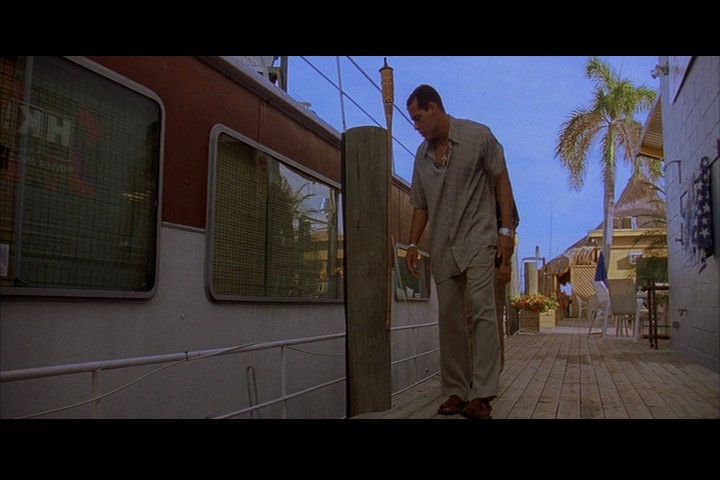

Supplement: S1 Dataset — (ZIP) [file pone.0264302.s001.zip › 2-fast-2-furious-00094951.jpg]

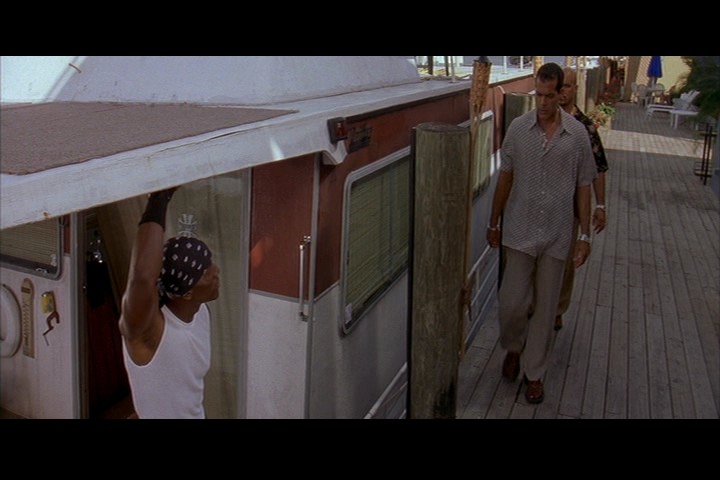

Supplement: S1 Dataset — (ZIP) [file pone.0264302.s001.zip › 2-fast-2-furious-00095141.jpg]

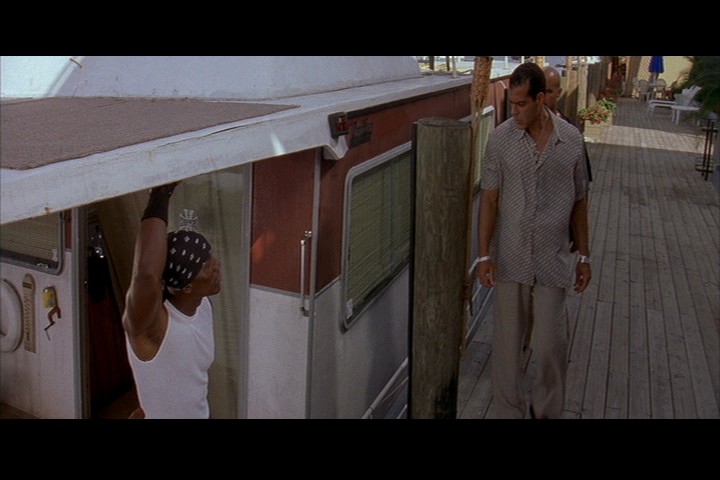

Supplement: S1 Dataset — (ZIP) [file pone.0264302.s001.zip › 2-fast-2-furious-00095161.jpg]

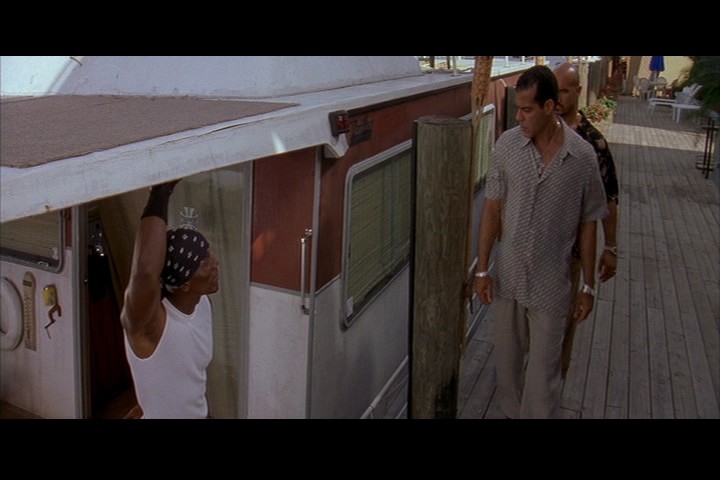

Supplement: S1 Dataset — (ZIP) [file pone.0264302.s001.zip › 2-fast-2-furious-00095171.jpg]

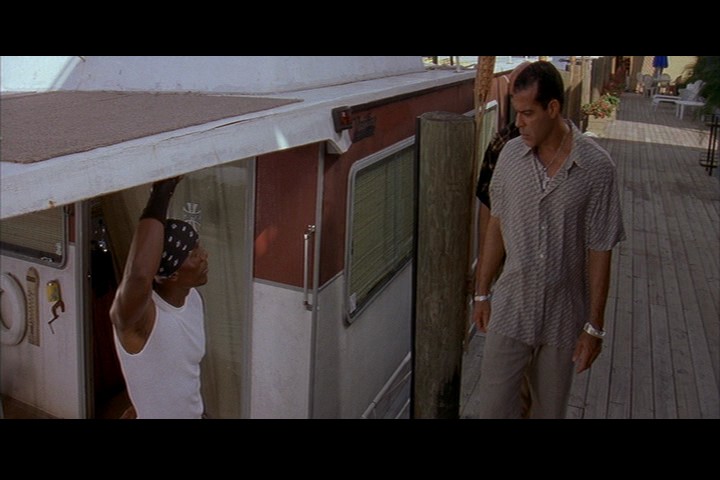

Supplement: S1 Dataset — (ZIP) [file pone.0264302.s001.zip › 2-fast-2-furious-00095191.jpg]

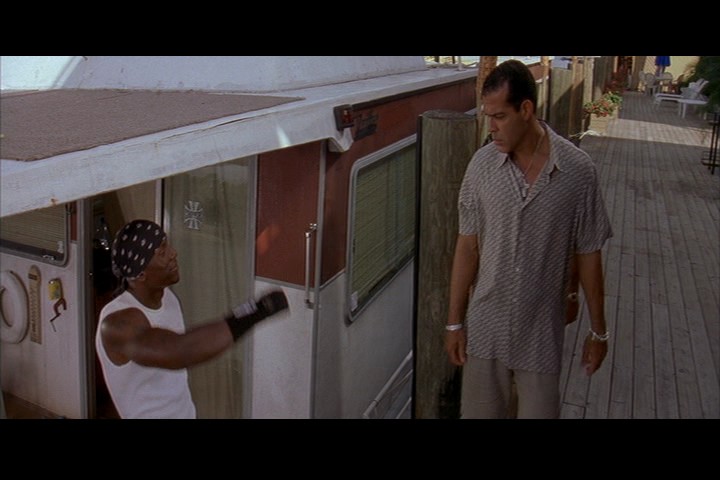

Supplement: S1 Dataset — (ZIP) [file pone.0264302.s001.zip › 2-fast-2-furious-00095201.jpg]

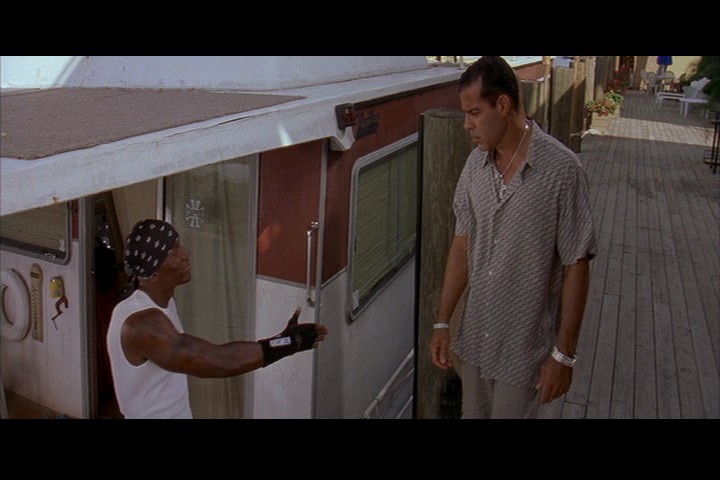

Supplement: S1 Dataset — (ZIP) [file pone.0264302.s001.zip › 2-fast-2-furious-00095211.jpg]

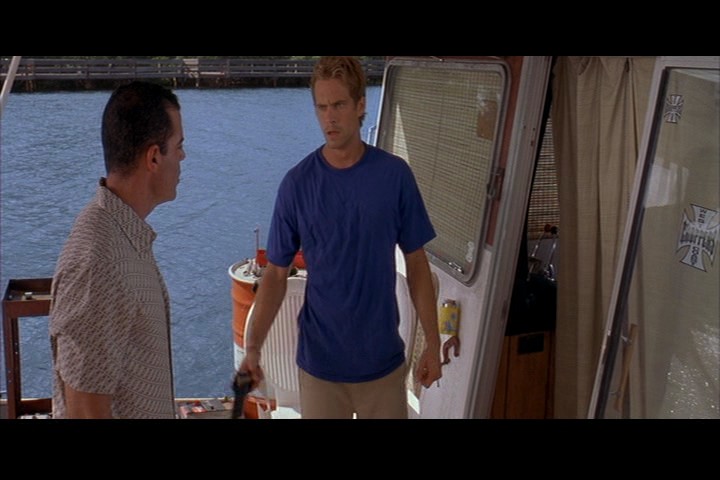

Supplement: S1 Dataset — (ZIP) [file pone.0264302.s001.zip › 2-fast-2-furious-00098111.jpg]

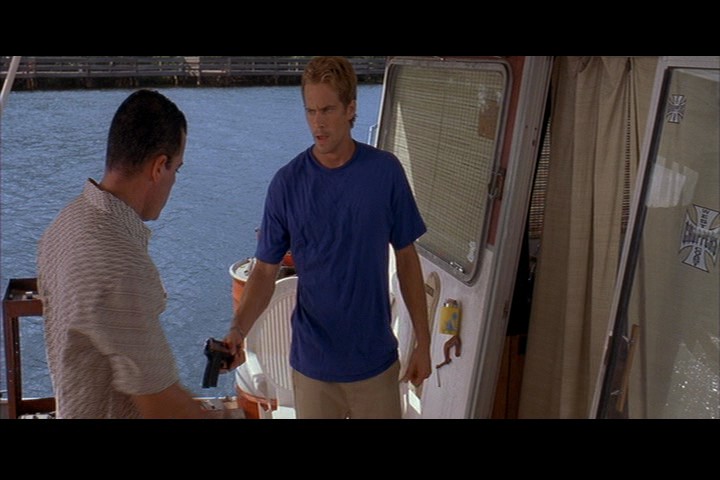

Supplement: S1 Dataset — (ZIP) [file pone.0264302.s001.zip › 2-fast-2-furious-00098131.jpg]

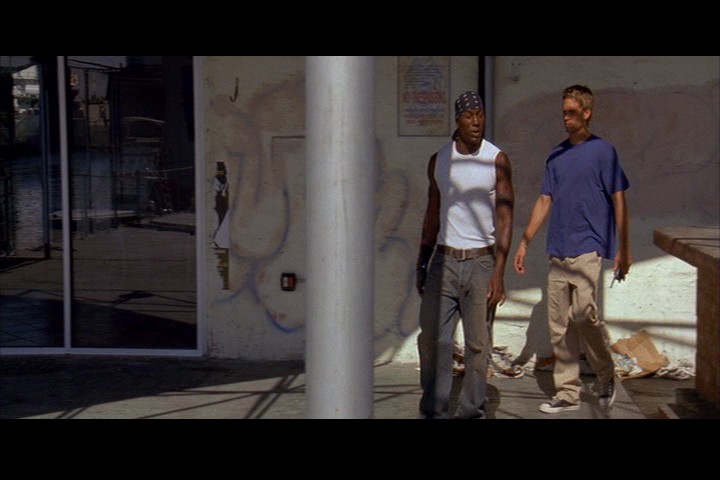

Supplement: S1 Dataset — (ZIP) [file pone.0264302.s001.zip › 2-fast-2-furious-00101611.jpg]

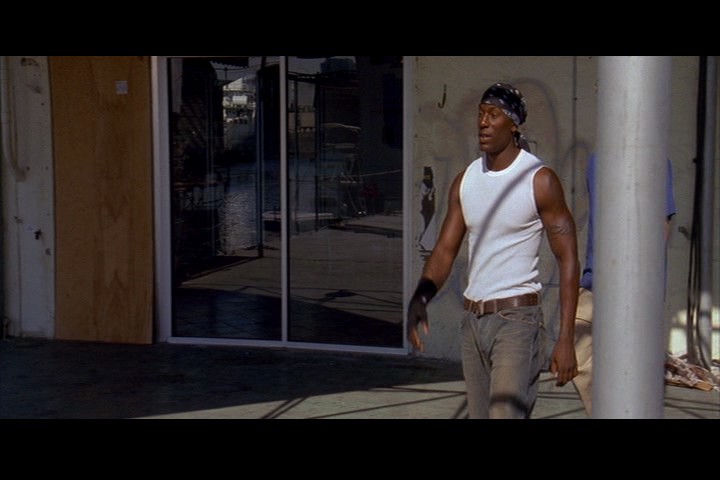

Supplement: S1 Dataset — (ZIP) [file pone.0264302.s001.zip › 2-fast-2-furious-00101661.jpg]

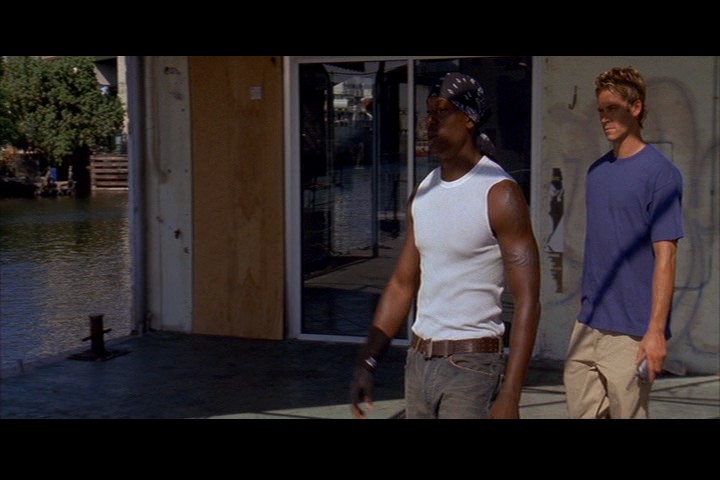

Supplement: S1 Dataset — (ZIP) [file pone.0264302.s001.zip › 2-fast-2-furious-00101691.jpg]

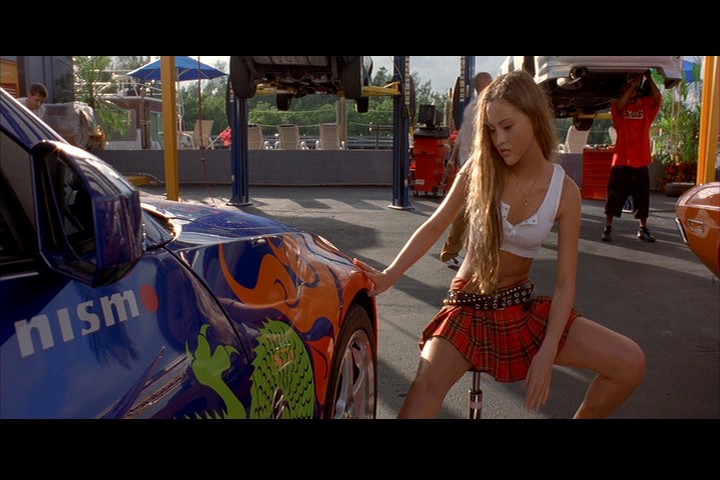

Supplement: S1 Dataset — (ZIP) [file pone.0264302.s001.zip › 2-fast-2-furious-00103201.jpg]

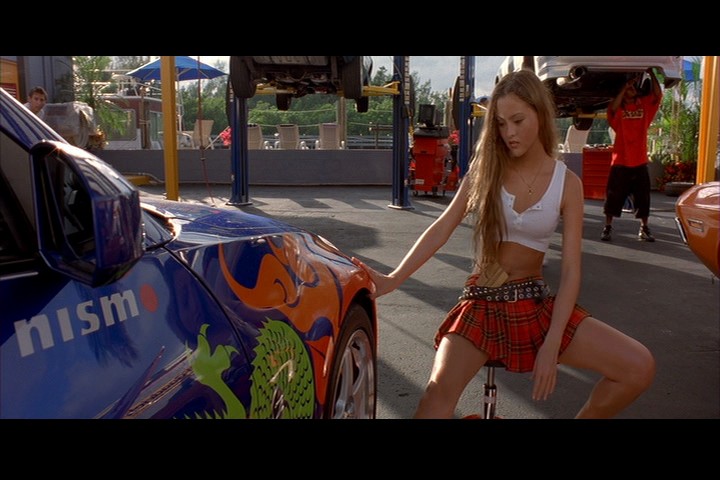

Supplement: S1 Dataset — (ZIP) [file pone.0264302.s001.zip › 2-fast-2-furious-00103211.jpg]

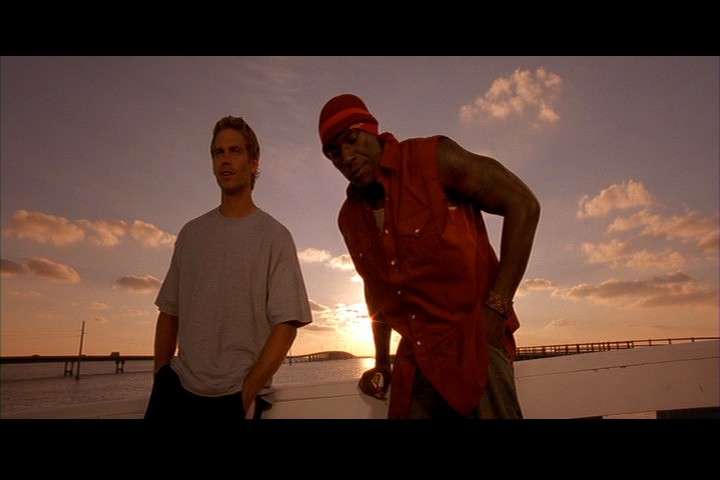

Supplement: S1 Dataset — (ZIP) [file pone.0264302.s001.zip › 2-fast-2-furious-00103821.jpg]

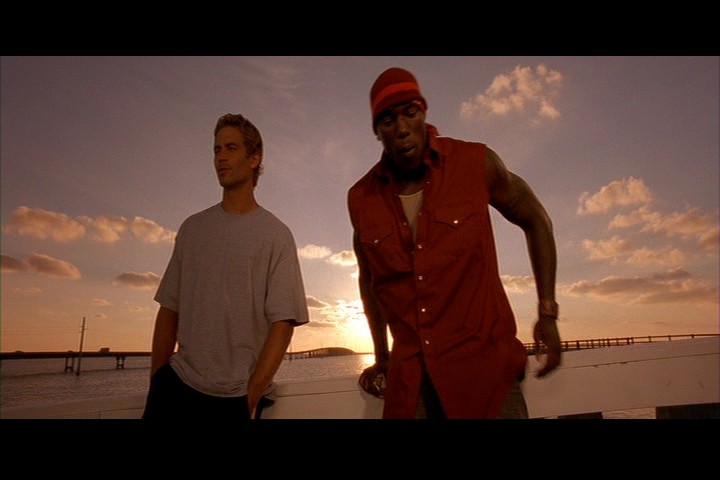

Supplement: S1 Dataset — (ZIP) [file pone.0264302.s001.zip › 2-fast-2-furious-00103831.jpg]

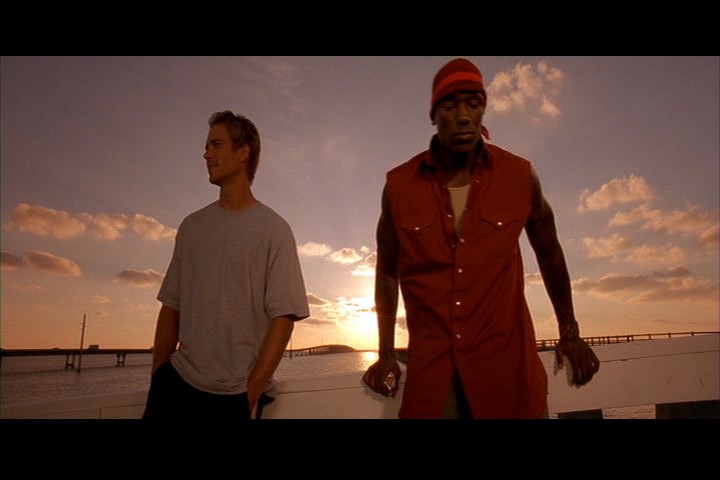

Supplement: S1 Dataset — (ZIP) [file pone.0264302.s001.zip › 2-fast-2-furious-00103841.jpg]

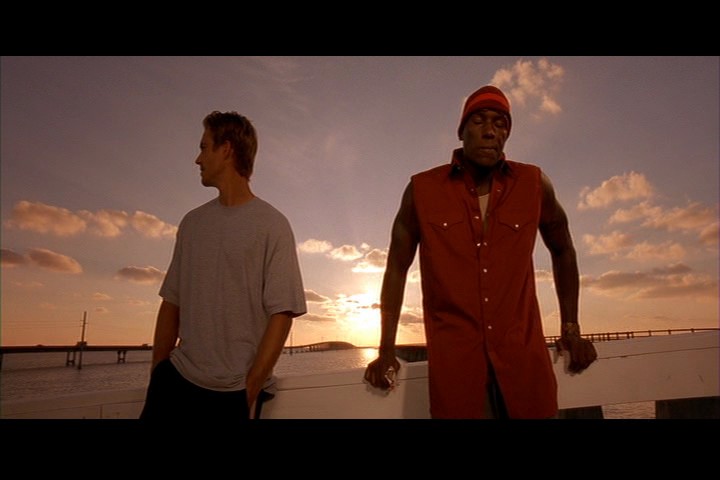

Supplement: S1 Dataset — (ZIP) [file pone.0264302.s001.zip › 2-fast-2-furious-00103851.jpg]

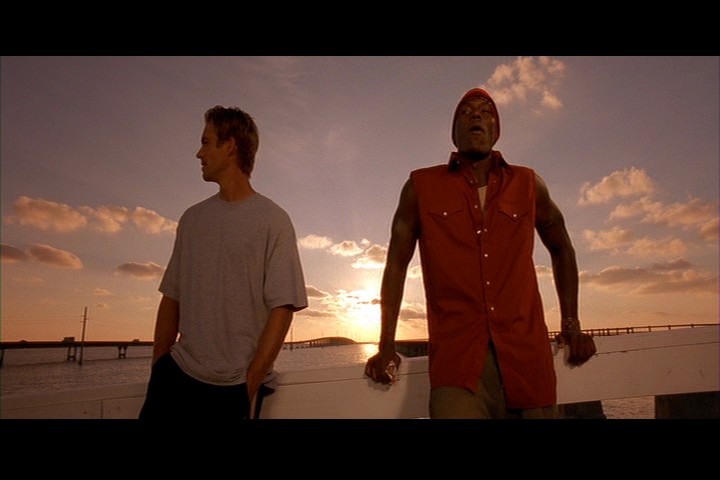

Supplement: S1 Dataset — (ZIP) [file pone.0264302.s001.zip › 2-fast-2-furious-00103861.jpg]

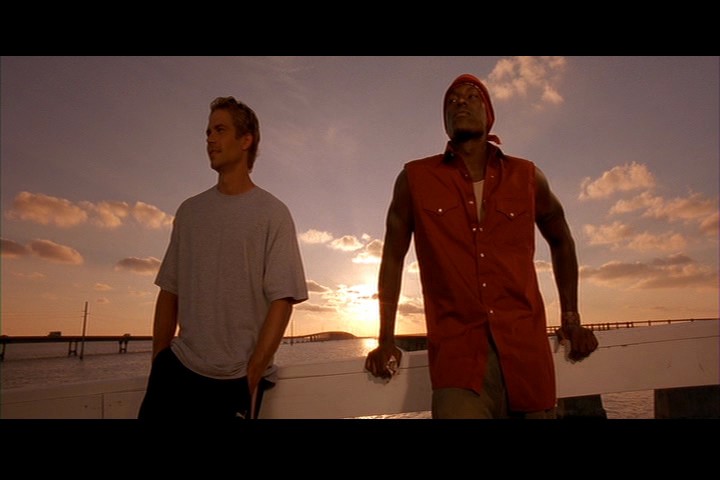

Supplement: S1 Dataset — (ZIP) [file pone.0264302.s001.zip › 2-fast-2-furious-00103871.jpg]

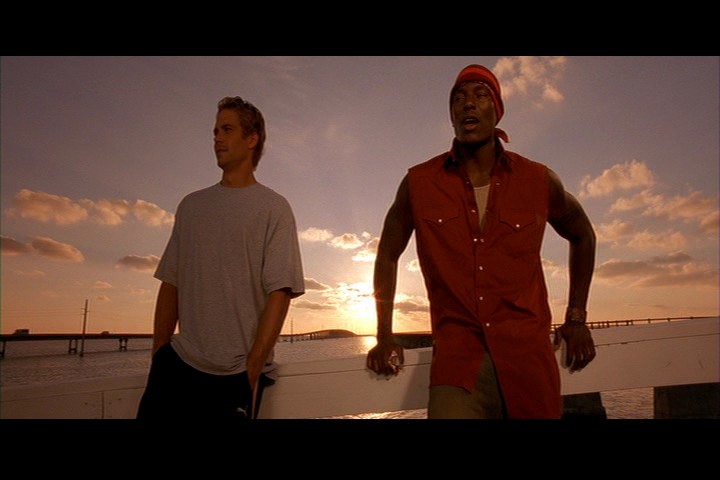

Supplement: S1 Dataset — (ZIP) [file pone.0264302.s001.zip › 2-fast-2-furious-00103891.jpg]

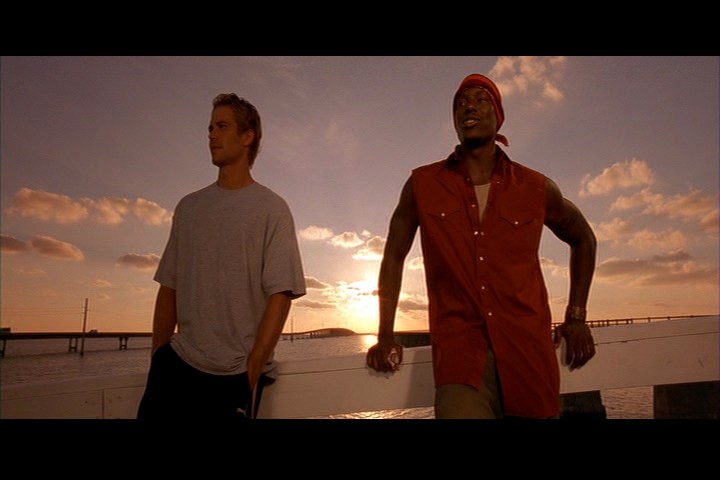

Supplement: S1 Dataset — (ZIP) [file pone.0264302.s001.zip › 2-fast-2-furious-00103901.jpg]

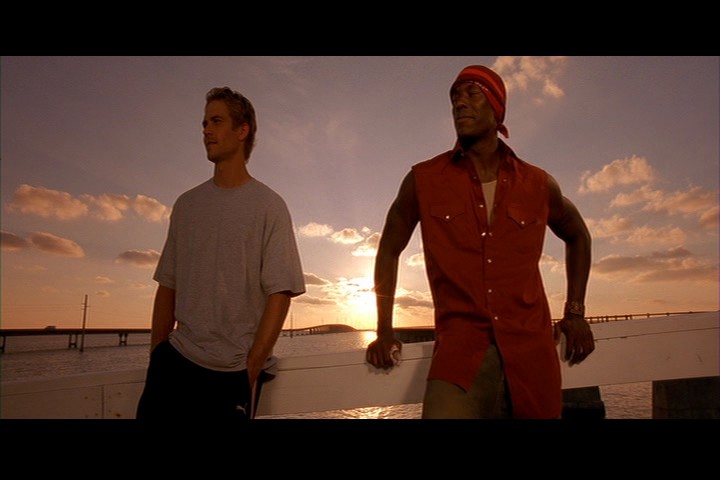

Supplement: S1 Dataset — (ZIP) [file pone.0264302.s001.zip › 2-fast-2-furious-00103931.jpg]

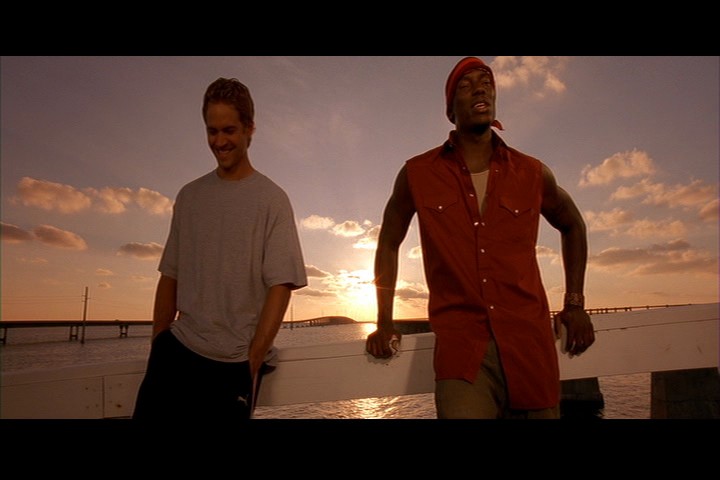

Supplement: S1 Dataset — (ZIP) [file pone.0264302.s001.zip › 2-fast-2-furious-00104051.jpg]

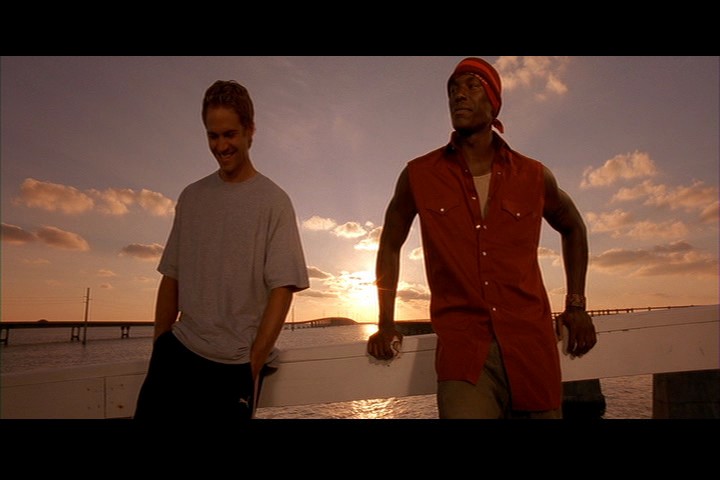

Supplement: S1 Dataset — (ZIP) [file pone.0264302.s001.zip › 2-fast-2-furious-00104091.jpg]

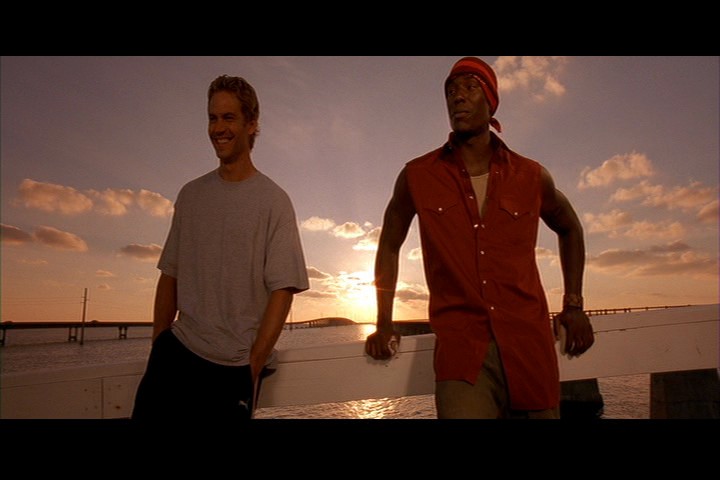

Supplement: S1 Dataset — (ZIP) [file pone.0264302.s001.zip › 2-fast-2-furious-00104131.jpg]

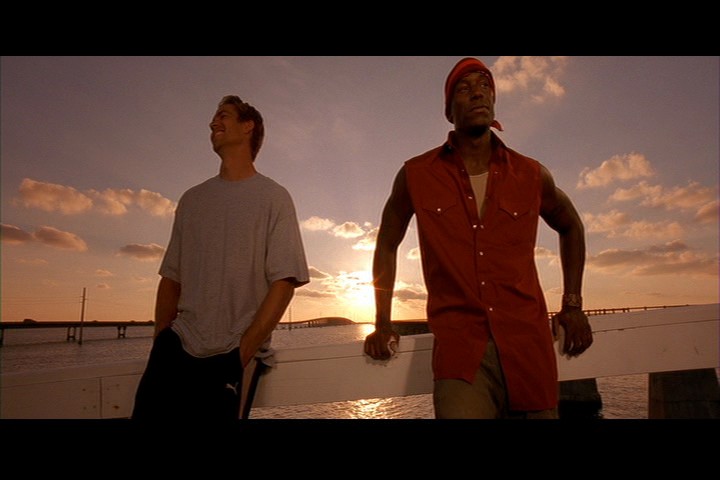

Supplement: S1 Dataset — (ZIP) [file pone.0264302.s001.zip › 2-fast-2-furious-00104191.jpg]

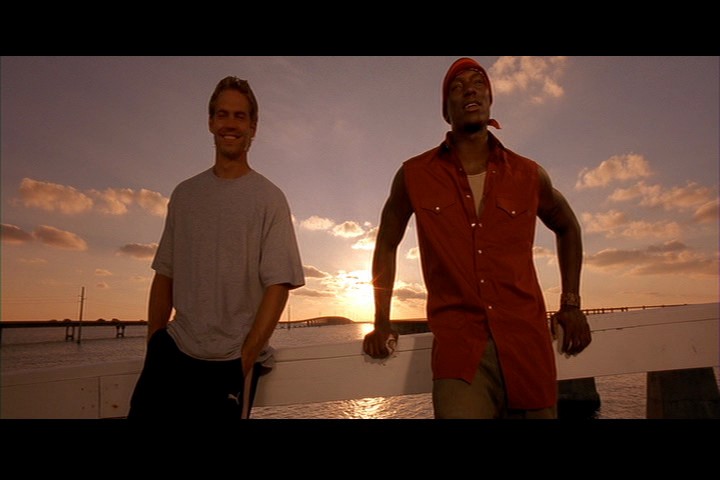

Supplement: S1 Dataset — (ZIP) [file pone.0264302.s001.zip › 2-fast-2-furious-00104261.jpg]

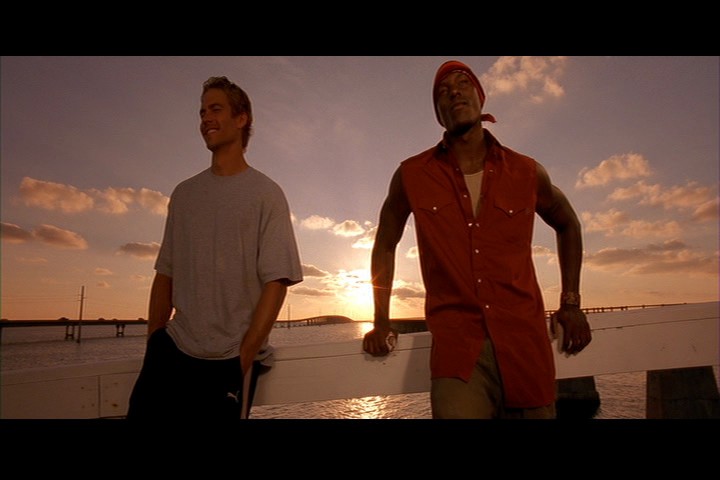

Supplement: S1 Dataset — (ZIP) [file pone.0264302.s001.zip › 2-fast-2-furious-00104291.jpg]

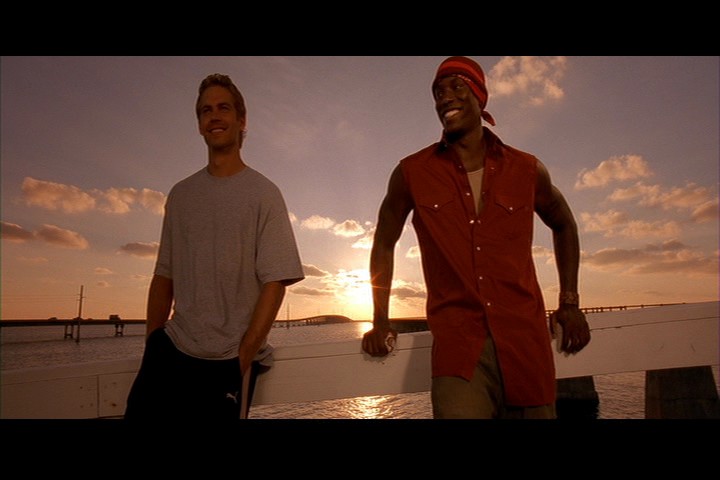

Supplement: S1 Dataset — (ZIP) [file pone.0264302.s001.zip › 2-fast-2-furious-00104401.jpg]

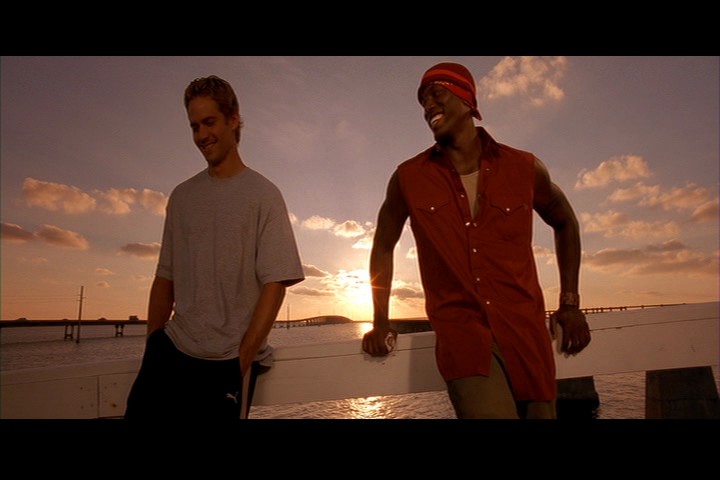

Supplement: S1 Dataset — (ZIP) [file pone.0264302.s001.zip › 2-fast-2-furious-00104421.jpg]

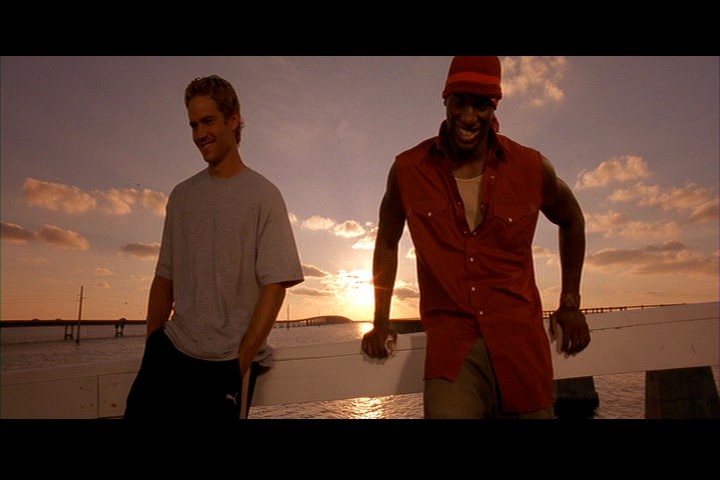

Supplement: S1 Dataset — (ZIP) [file pone.0264302.s001.zip › 2-fast-2-furious-00104441.jpg]

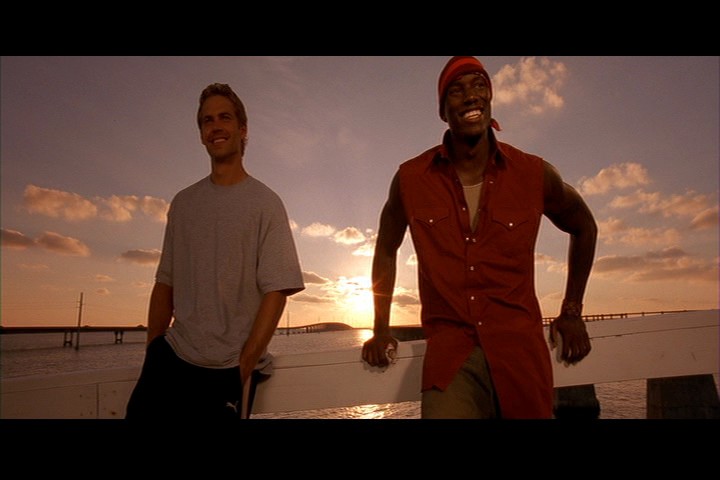

Supplement: S1 Dataset — (ZIP) [file pone.0264302.s001.zip › 2-fast-2-furious-00104461.jpg]

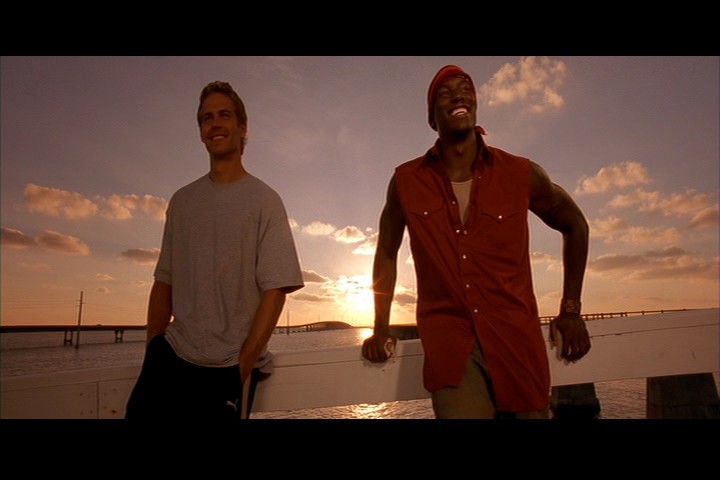

Supplement: S1 Dataset — (ZIP) [file pone.0264302.s001.zip › 2-fast-2-furious-00104471.jpg]

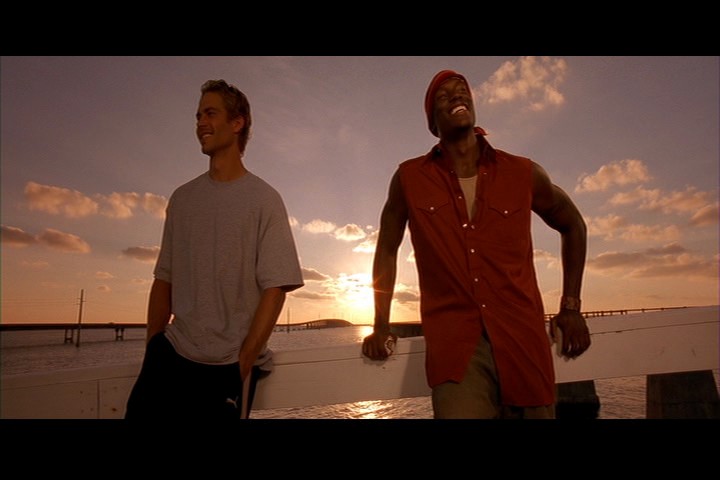

Supplement: S1 Dataset — (ZIP) [file pone.0264302.s001.zip › 2-fast-2-furious-00104481.jpg]

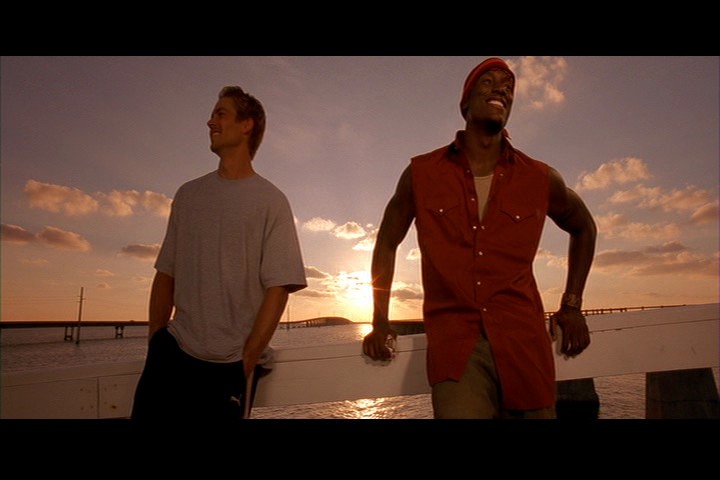

Supplement: S1 Dataset — (ZIP) [file pone.0264302.s001.zip › 2-fast-2-furious-00104501.jpg]

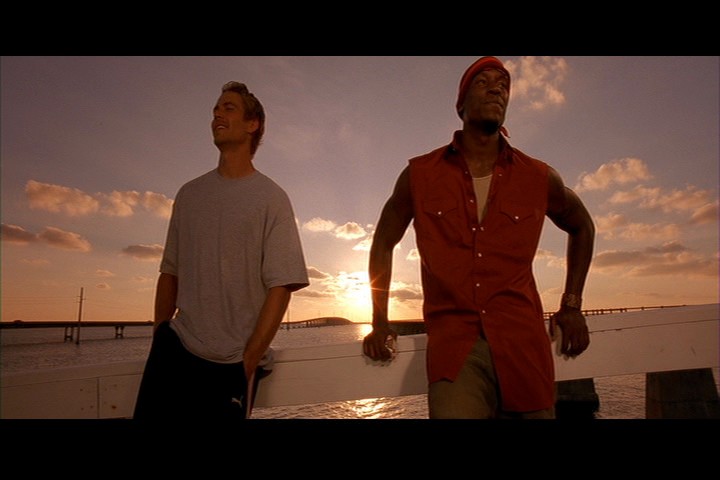

Supplement: S1 Dataset — (ZIP) [file pone.0264302.s001.zip › 2-fast-2-furious-00104521.jpg]

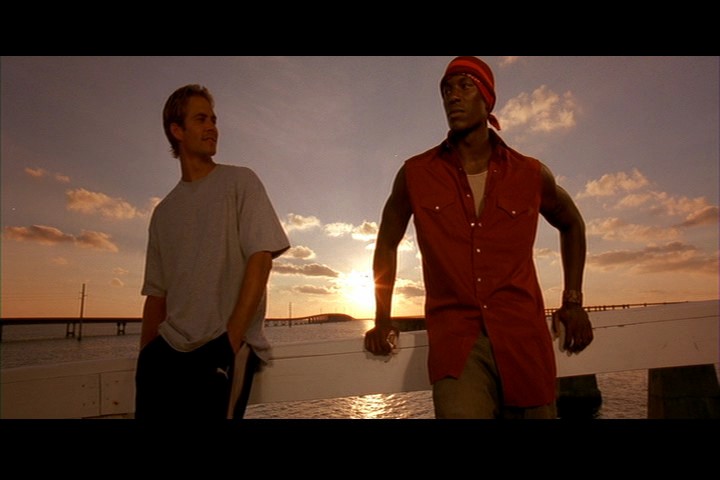

Supplement: S1 Dataset — (ZIP) [file pone.0264302.s001.zip › 2-fast-2-furious-00105701.jpg]

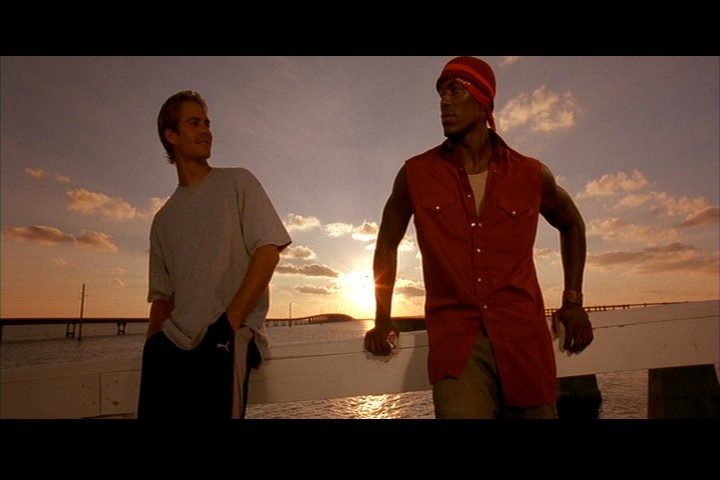

Supplement: S1 Dataset — (ZIP) [file pone.0264302.s001.zip › 2-fast-2-furious-00105711.jpg]

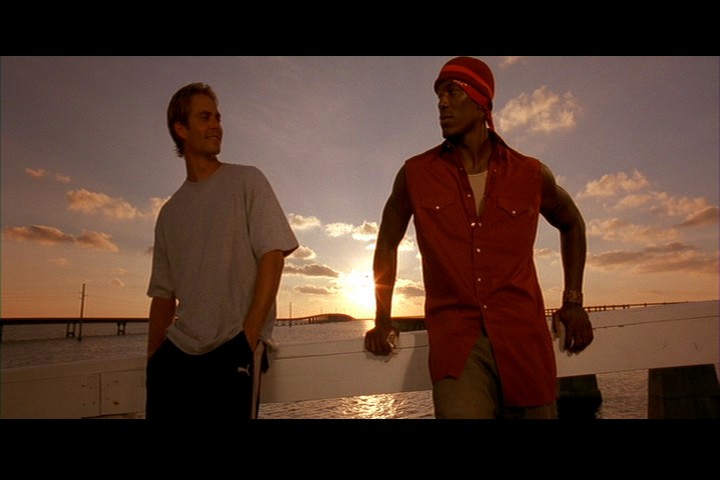

Supplement: S1 Dataset — (ZIP) [file pone.0264302.s001.zip › 2-fast-2-furious-00105721.jpg]

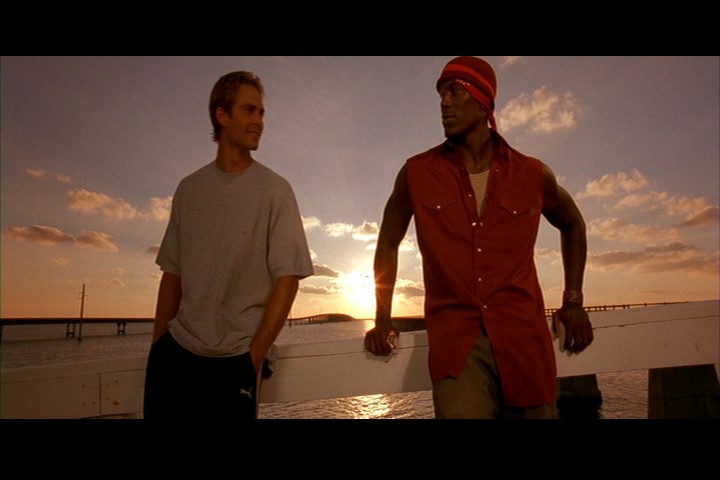

Supplement: S1 Dataset — (ZIP) [file pone.0264302.s001.zip › 2-fast-2-furious-00105731.jpg]

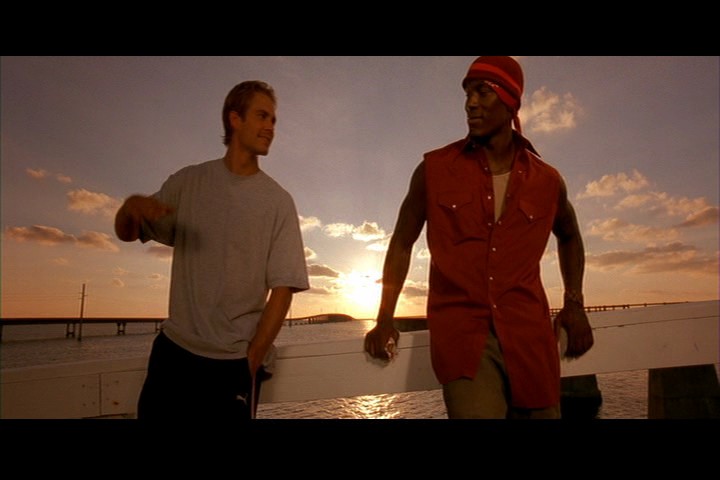

Supplement: S1 Dataset — (ZIP) [file pone.0264302.s001.zip › 2-fast-2-furious-00105751.jpg]

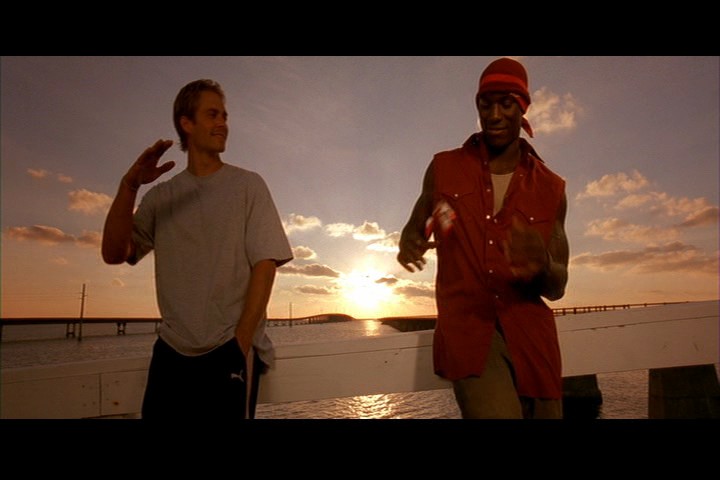

Supplement: S1 Dataset — (ZIP) [file pone.0264302.s001.zip › 2-fast-2-furious-00105761.jpg]

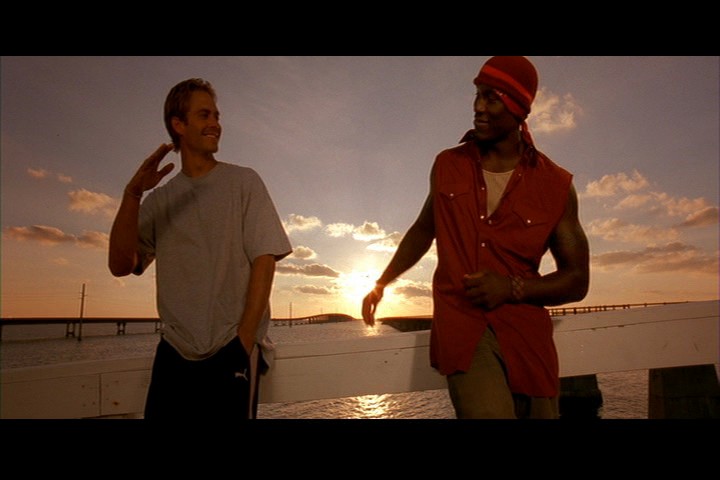

Supplement: S1 Dataset — (ZIP) [file pone.0264302.s001.zip › 2-fast-2-furious-00105771.jpg]

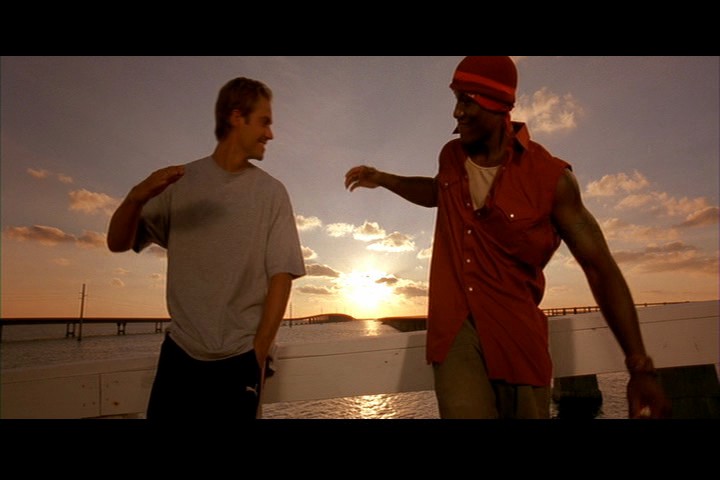

Supplement: S1 Dataset — (ZIP) [file pone.0264302.s001.zip › 2-fast-2-furious-00105781.jpg]

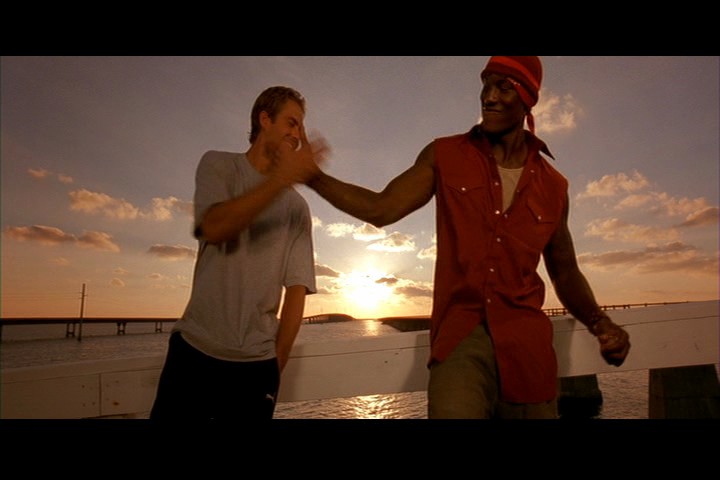

Supplement: S1 Dataset — (ZIP) [file pone.0264302.s001.zip › 2-fast-2-furious-00105791.jpg]

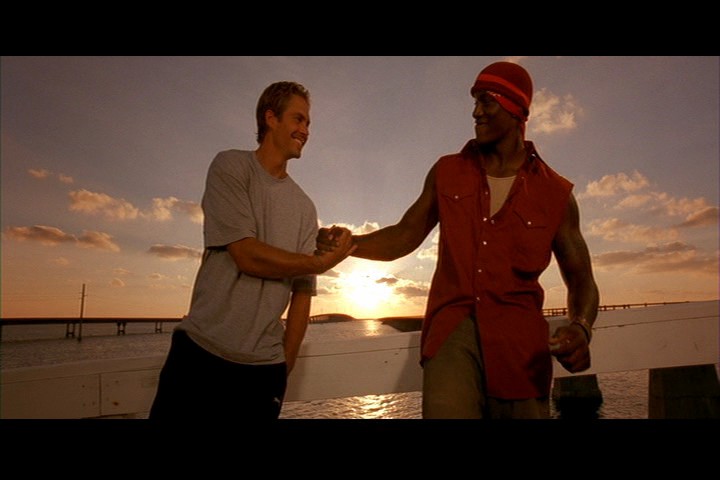

Supplement: S1 Dataset — (ZIP) [file pone.0264302.s001.zip › 2-fast-2-furious-00105801.jpg]

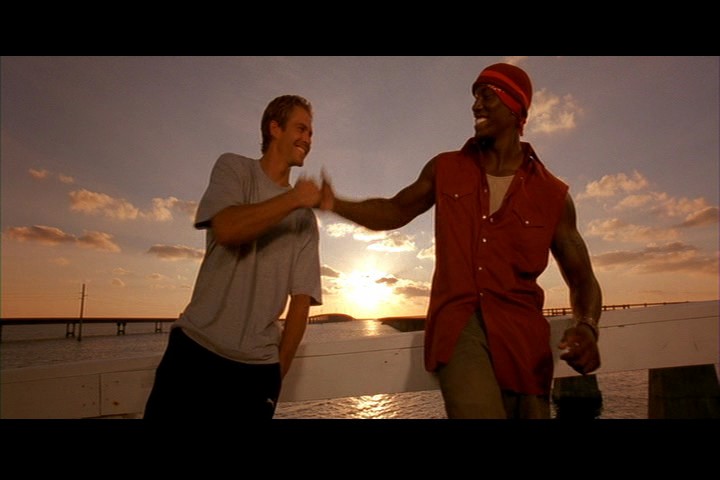

Supplement: S1 Dataset — (ZIP) [file pone.0264302.s001.zip › 2-fast-2-furious-00105811.jpg]

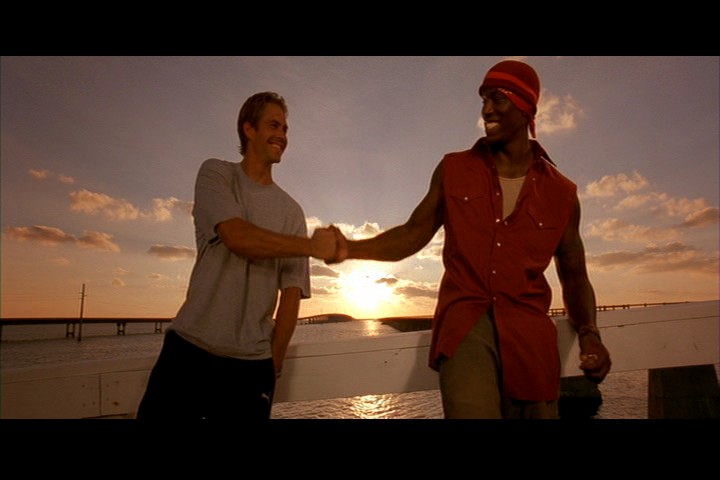

Supplement: S1 Dataset — (ZIP) [file pone.0264302.s001.zip › 2-fast-2-furious-00105821.jpg]

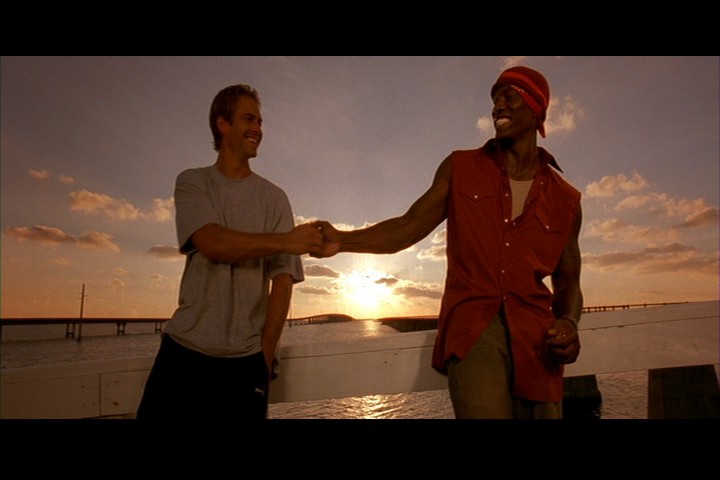

Supplement: S1 Dataset — (ZIP) [file pone.0264302.s001.zip › 2-fast-2-furious-00105831.jpg]

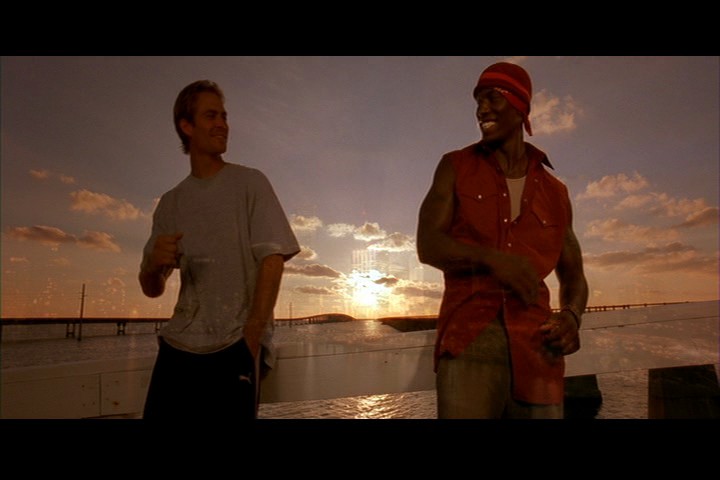

Supplement: S1 Dataset — (ZIP) [file pone.0264302.s001.zip › 2-fast-2-furious-00105841.jpg]

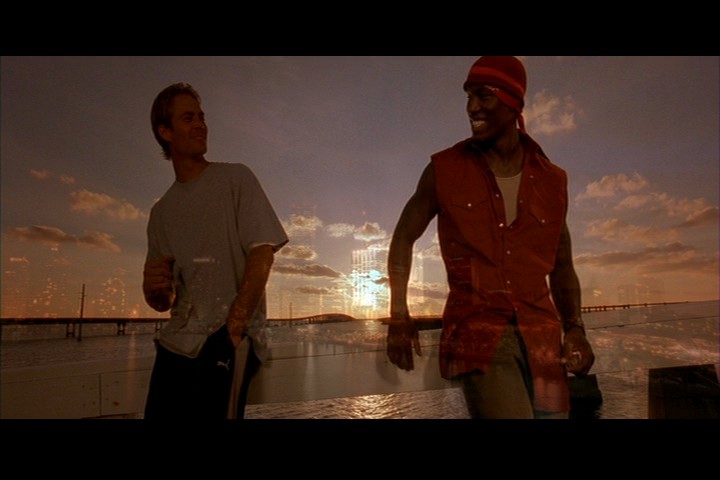

Supplement: S1 Dataset — (ZIP) [file pone.0264302.s001.zip › 2-fast-2-furious-00105851.jpg]

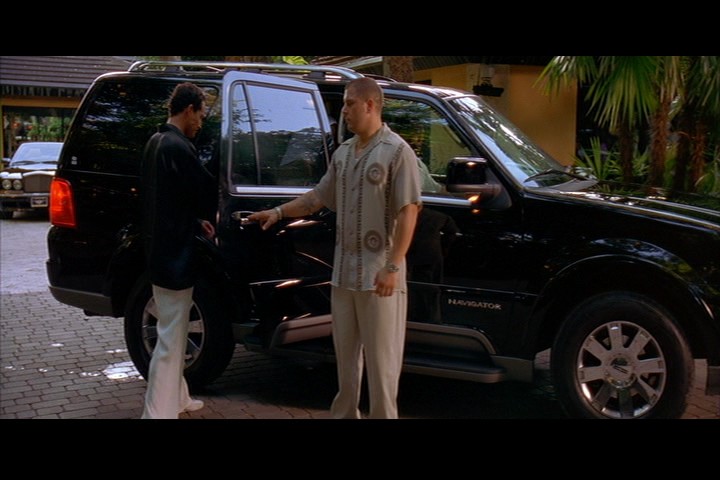

Supplement: S1 Dataset — (ZIP) [file pone.0264302.s001.zip › 2-fast-2-furious-00106261.jpg]

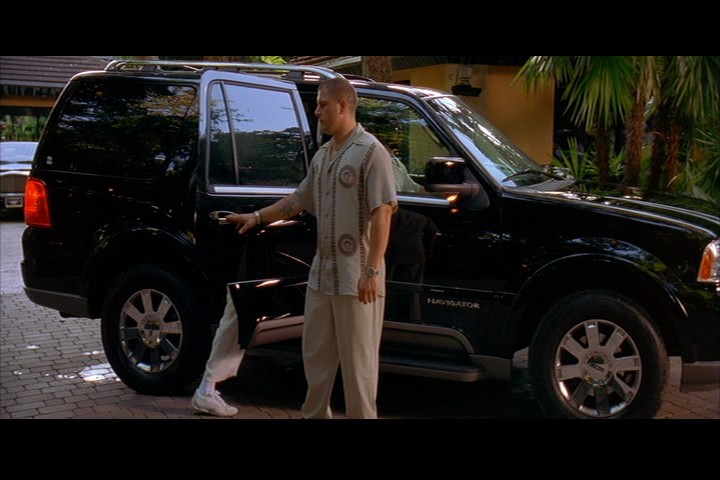

Supplement: S1 Dataset — (ZIP) [file pone.0264302.s001.zip › 2-fast-2-furious-00106291.jpg]

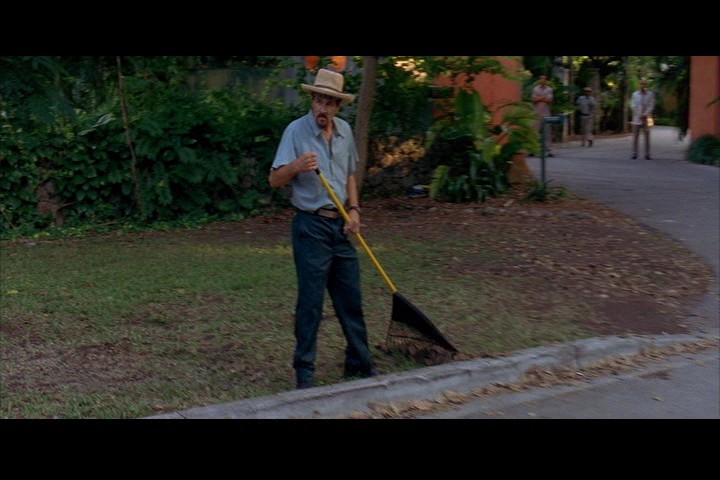

Supplement: S1 Dataset — (ZIP) [file pone.0264302.s001.zip › 2-fast-2-furious-00106461.jpg]

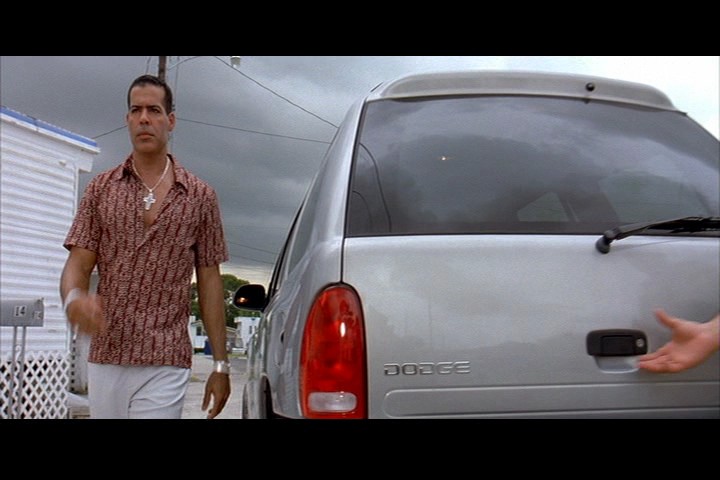

Supplement: S1 Dataset — (ZIP) [file pone.0264302.s001.zip › 2-fast-2-furious-00108691.jpg]

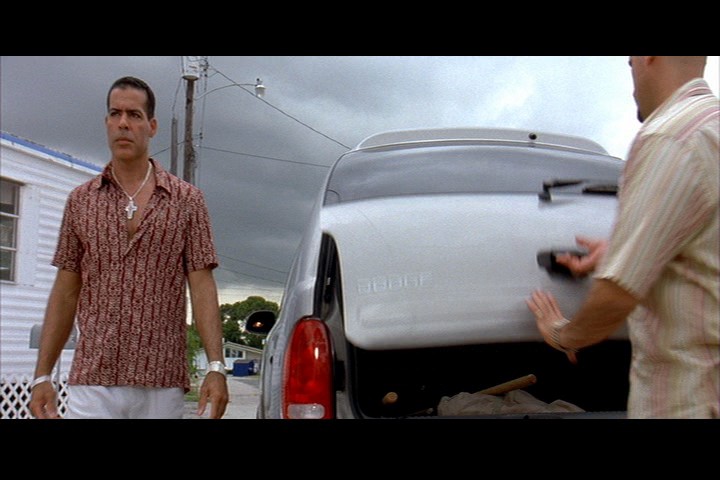

Supplement: S1 Dataset — (ZIP) [file pone.0264302.s001.zip › 2-fast-2-furious-00108711.jpg]

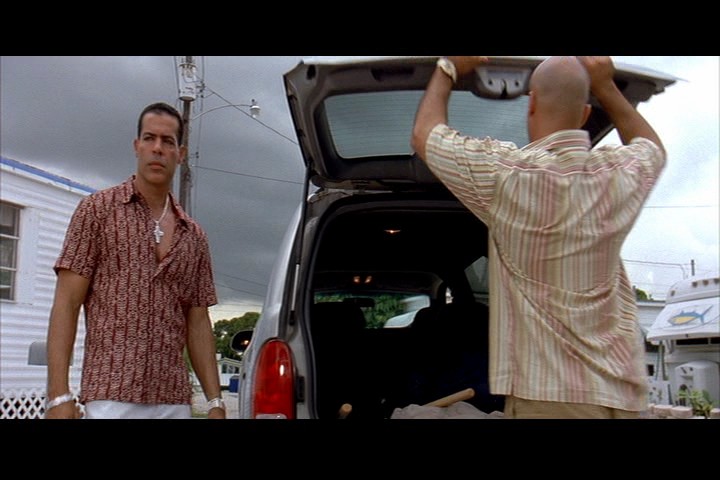

Supplement: S1 Dataset — (ZIP) [file pone.0264302.s001.zip › 2-fast-2-furious-00108731.jpg]

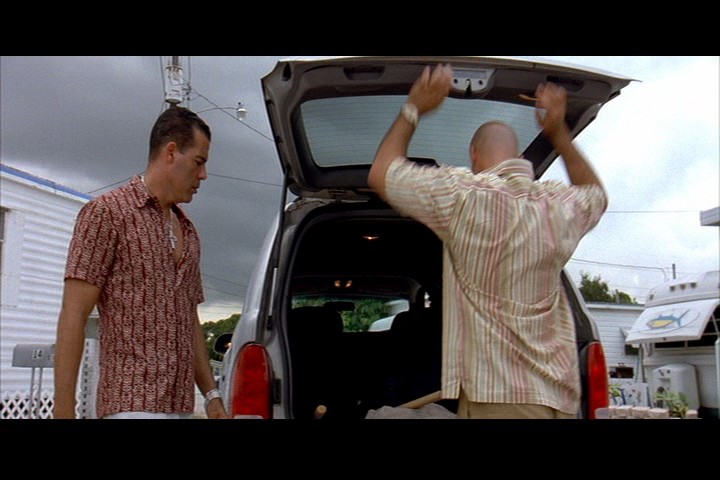

Supplement: S1 Dataset — (ZIP) [file pone.0264302.s001.zip › 2-fast-2-furious-00108741.jpg]

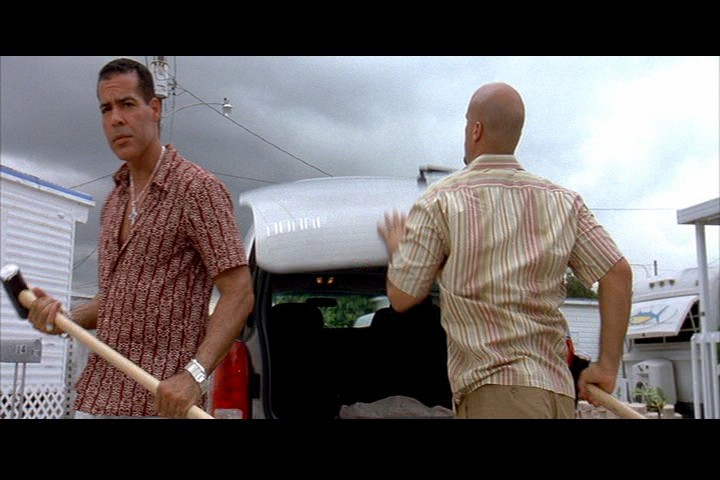

Supplement: S1 Dataset — (ZIP) [file pone.0264302.s001.zip › 2-fast-2-furious-00108831.jpg]

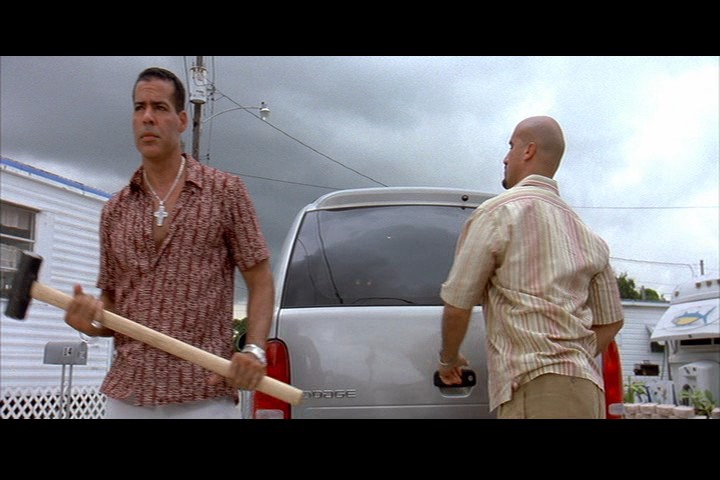

Supplement: S1 Dataset — (ZIP) [file pone.0264302.s001.zip › 2-fast-2-furious-00108851.jpg]

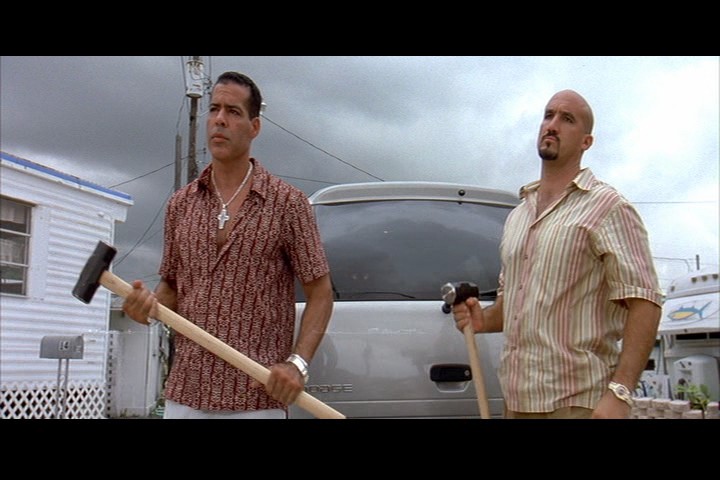

Supplement: S1 Dataset — (ZIP) [file pone.0264302.s001.zip › 2-fast-2-furious-00108881.jpg]

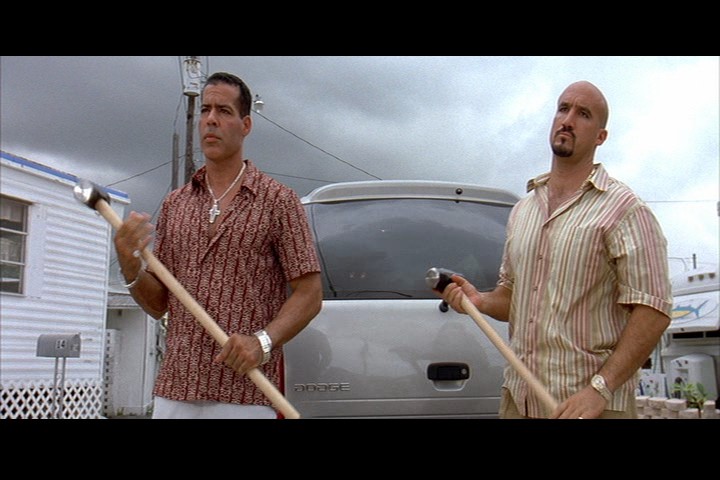

Supplement: S1 Dataset — (ZIP) [file pone.0264302.s001.zip › 2-fast-2-furious-00108891.jpg]

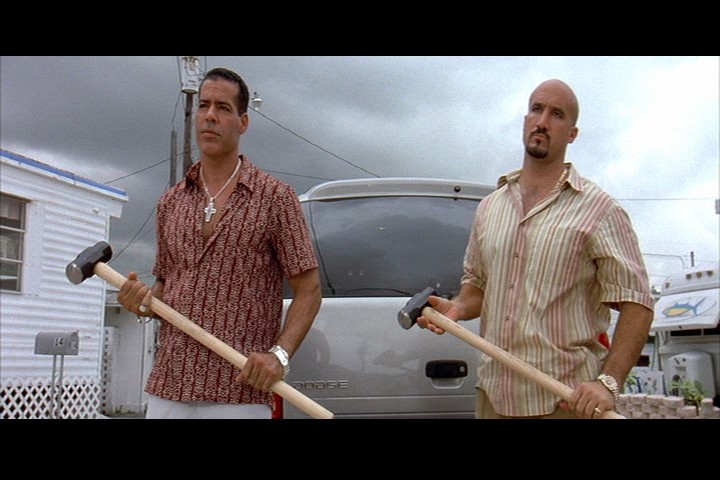

Supplement: S1 Dataset — (ZIP) [file pone.0264302.s001.zip › 2-fast-2-furious-00108901.jpg]

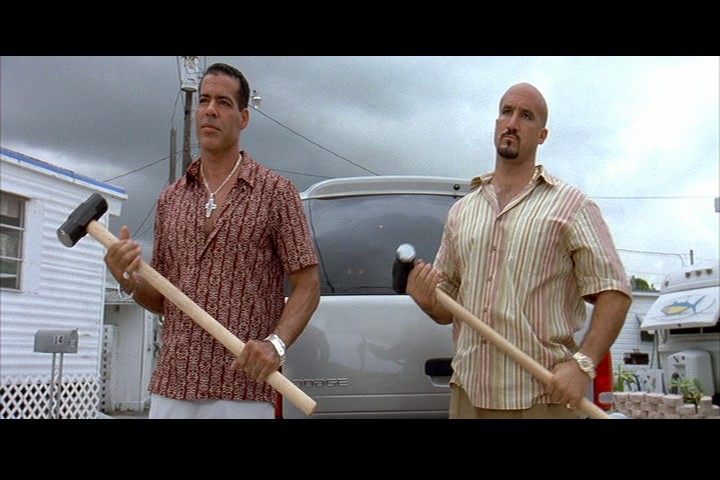

Supplement: S1 Dataset — (ZIP) [file pone.0264302.s001.zip › 2-fast-2-furious-00108941.jpg]

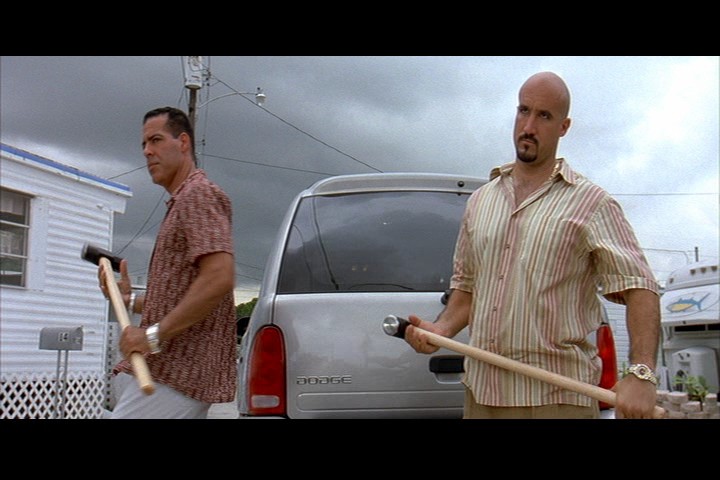

Supplement: S1 Dataset — (ZIP) [file pone.0264302.s001.zip › 2-fast-2-furious-00109011.jpg]

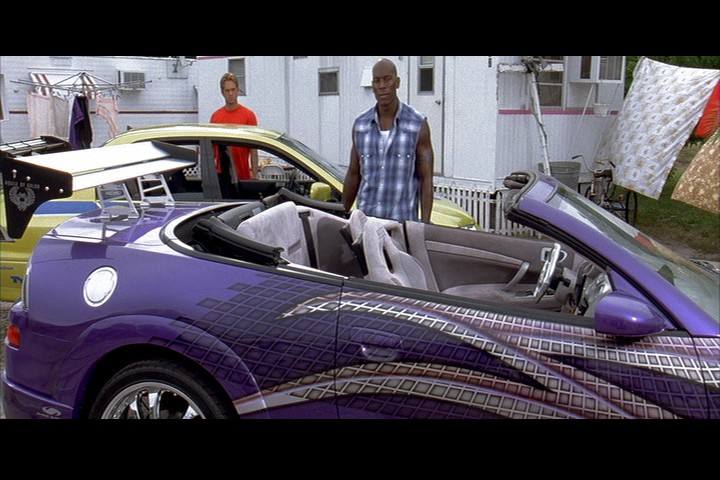

Supplement: S1 Dataset — (ZIP) [file pone.0264302.s001.zip › 2-fast-2-furious-00109051.jpg]

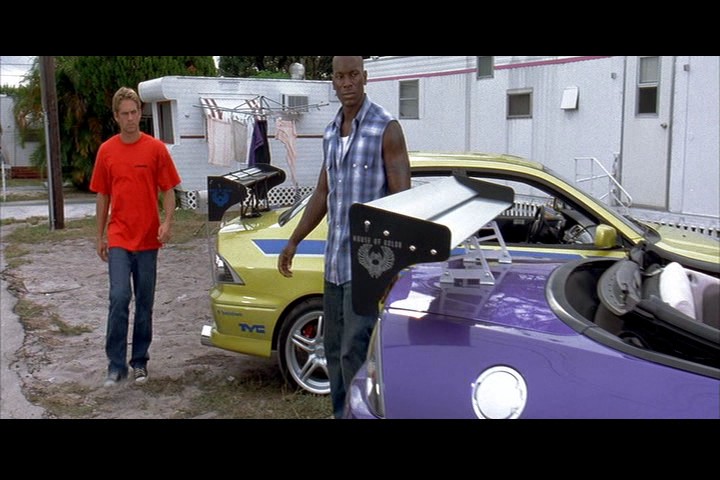

Supplement: S1 Dataset — (ZIP) [file pone.0264302.s001.zip › 2-fast-2-furious-00109171.jpg]

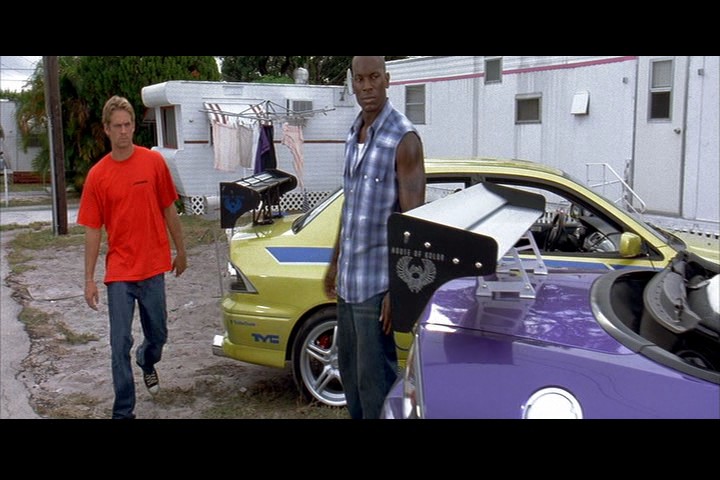

Supplement: S1 Dataset — (ZIP) [file pone.0264302.s001.zip › 2-fast-2-furious-00109181.jpg]

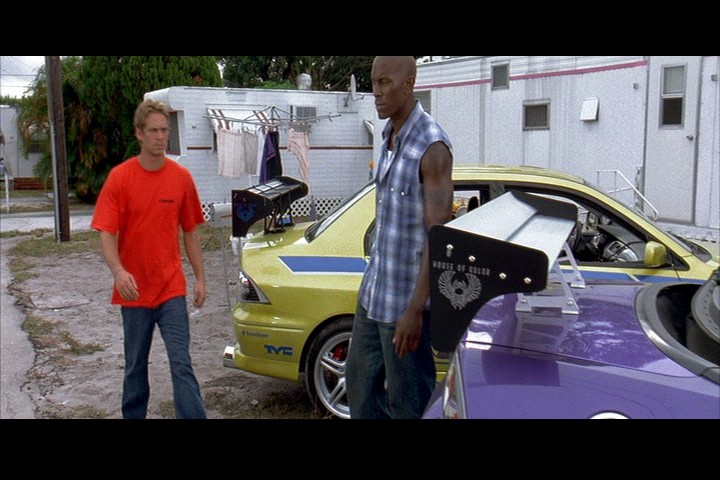

Supplement: S1 Dataset — (ZIP) [file pone.0264302.s001.zip › 2-fast-2-furious-00109191.jpg]

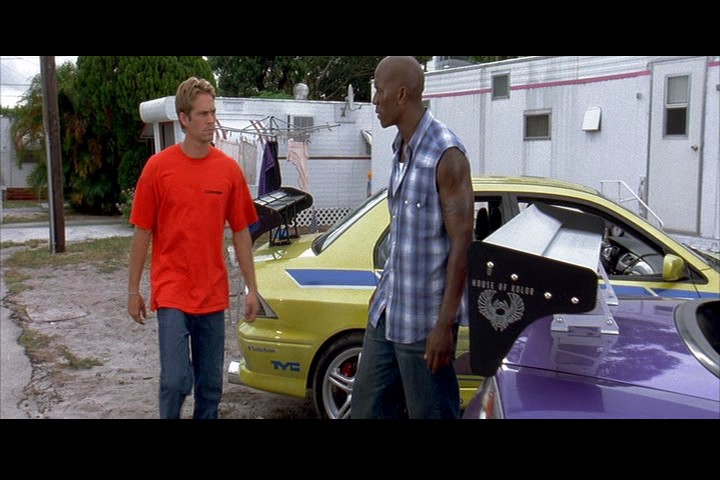

Supplement: S1 Dataset — (ZIP) [file pone.0264302.s001.zip › 2-fast-2-furious-00109201.jpg]

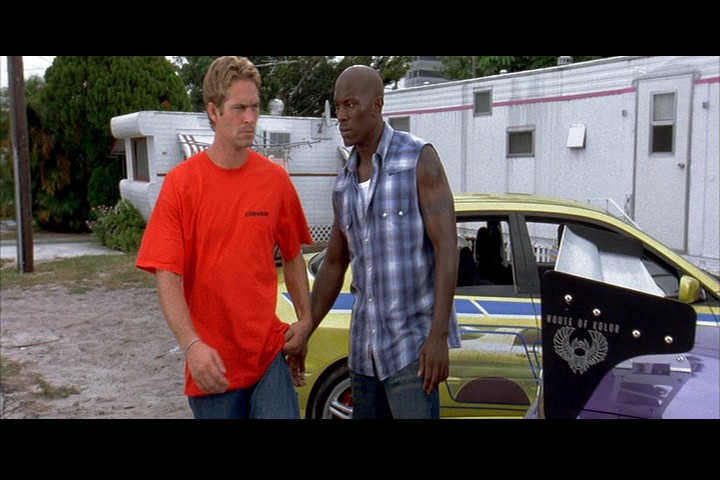

Supplement: S1 Dataset — (ZIP) [file pone.0264302.s001.zip › 2-fast-2-furious-00109221.jpg]

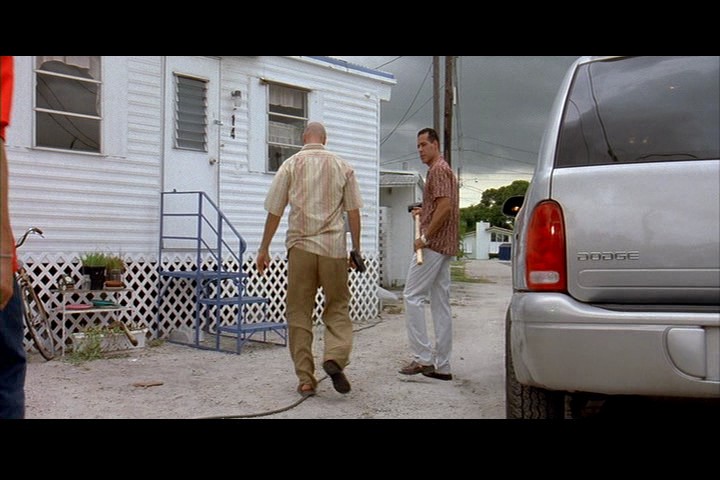

Supplement: S1 Dataset — (ZIP) [file pone.0264302.s001.zip › 2-fast-2-furious-00109241.jpg]

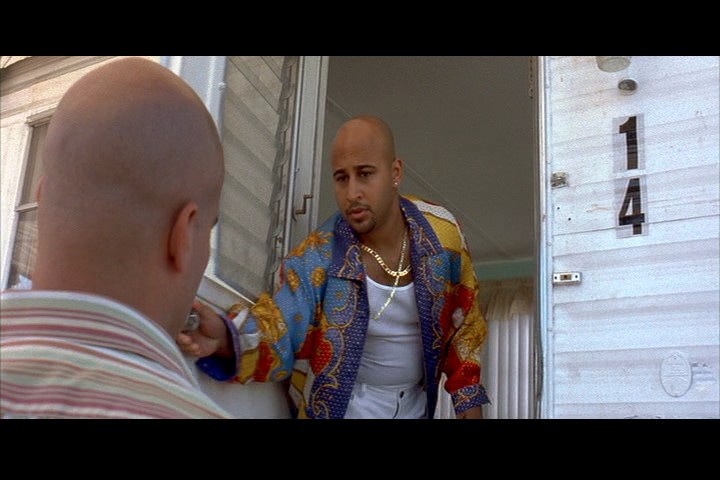

Supplement: S1 Dataset — (ZIP) [file pone.0264302.s001.zip › 2-fast-2-furious-00109351.jpg]

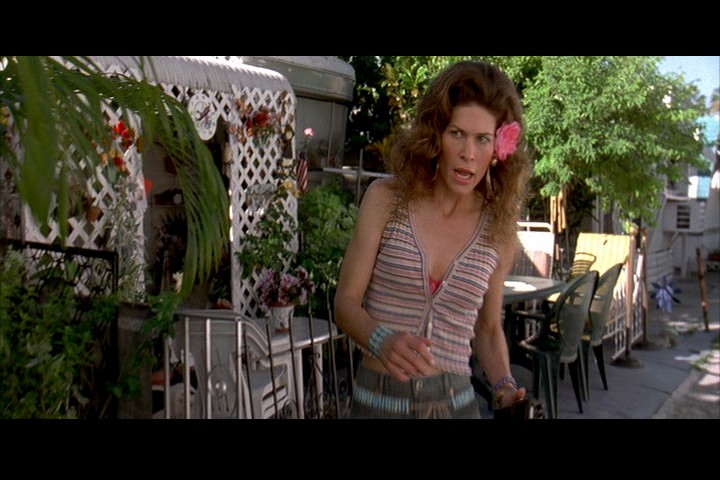

Supplement: S1 Dataset — (ZIP) [file pone.0264302.s001.zip › 2-fast-2-furious-00109571.jpg]

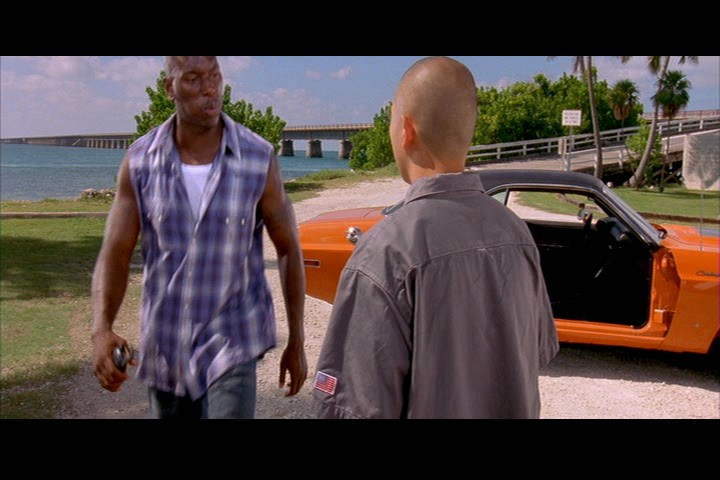

Supplement: S1 Dataset — (ZIP) [file pone.0264302.s001.zip › 2-fast-2-furious-00130661.jpg]

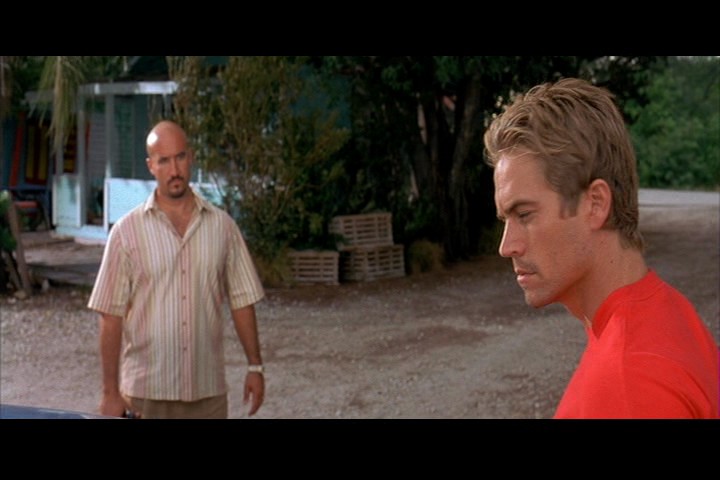

Supplement: S1 Dataset — (ZIP) [file pone.0264302.s001.zip › 2-fast-2-furious-00131371.jpg]

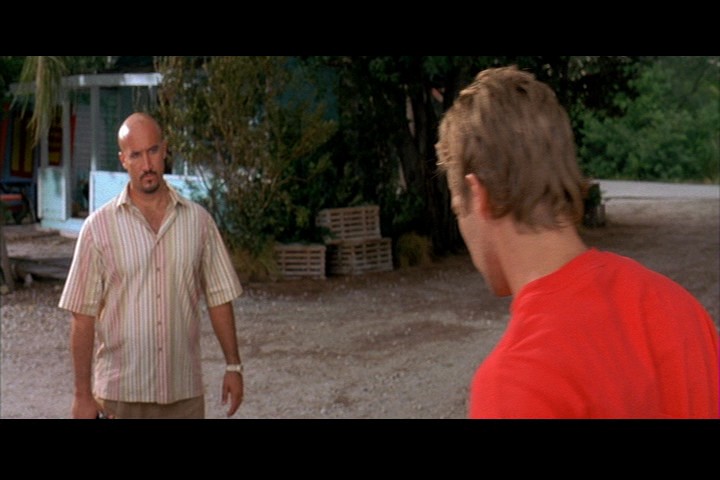

Supplement: S1 Dataset — (ZIP) [file pone.0264302.s001.zip › 2-fast-2-furious-00131381.jpg]

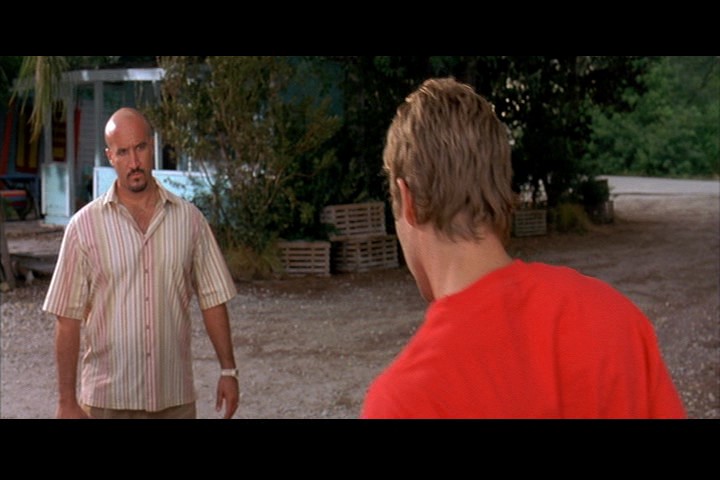

Supplement: S1 Dataset — (ZIP) [file pone.0264302.s001.zip › 2-fast-2-furious-00131391.jpg]

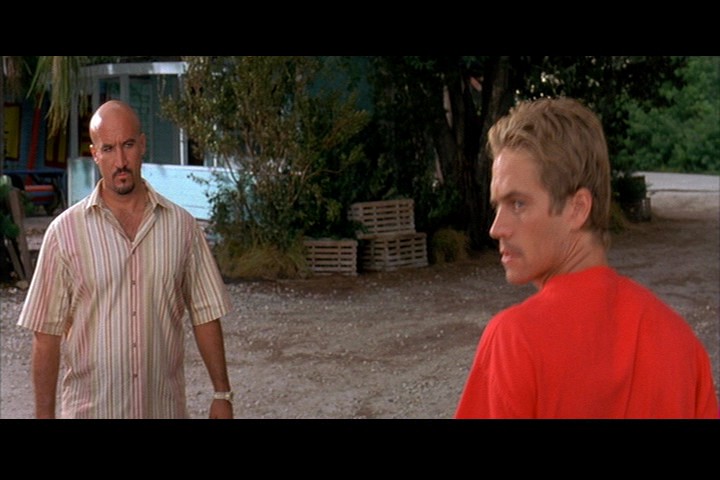

Supplement: S1 Dataset — (ZIP) [file pone.0264302.s001.zip › 2-fast-2-furious-00131411.jpg]

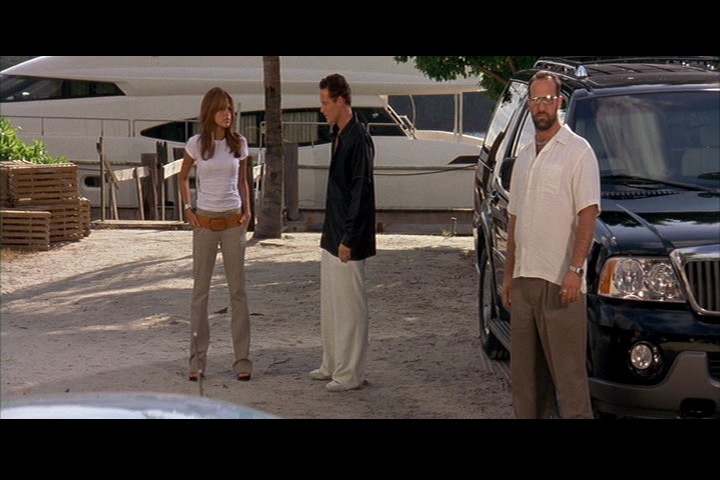

Supplement: S1 Dataset — (ZIP) [file pone.0264302.s001.zip › 2-fast-2-furious-00131551.jpg]
