# Supplementary figures and images for: LHPE-nets: A lightweight 2D and 3D human pose estimation model with well-structural deep networks and multi-view pose sample simplification method (part 1 of 8)
Source: PLoS One. 2022 Feb 23;17(2):e0264302. doi: 10.1371/journal.pone.0264302 (PMC8865690; doi:10.1371/journal.pone.0264302)

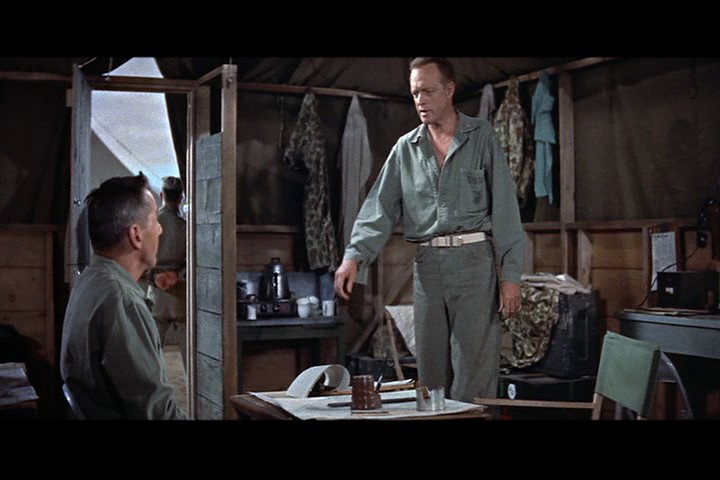

Supplement: S1 Dataset — (ZIP) [file pone.0264302.s001.zip › battle-cry-00135591.jpg]

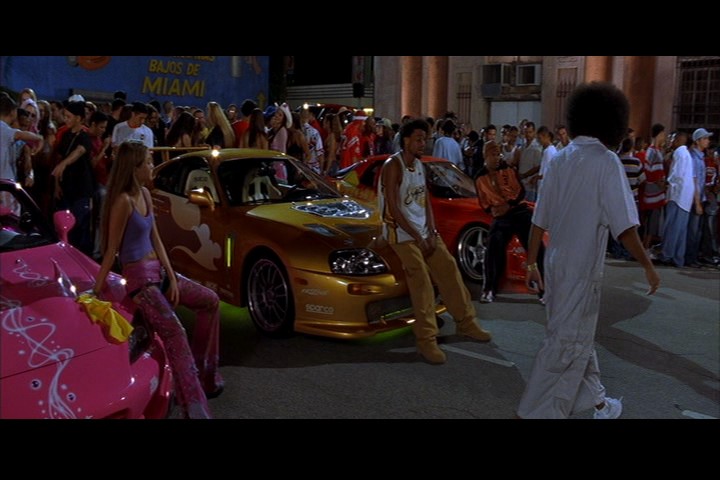

Supplement: S1 Dataset — (ZIP) [file pone.0264302.s001.zip › 2-fast-2-furious-00003631.jpg]

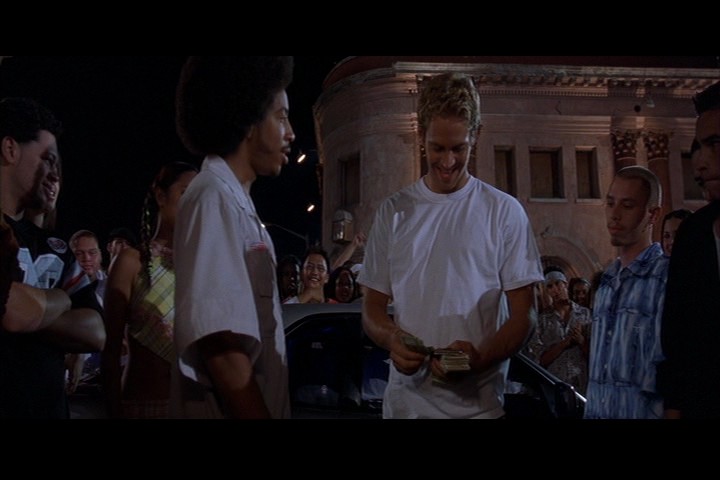

Supplement: S1 Dataset — (ZIP) [file pone.0264302.s001.zip › 2-fast-2-furious-00019861.jpg]

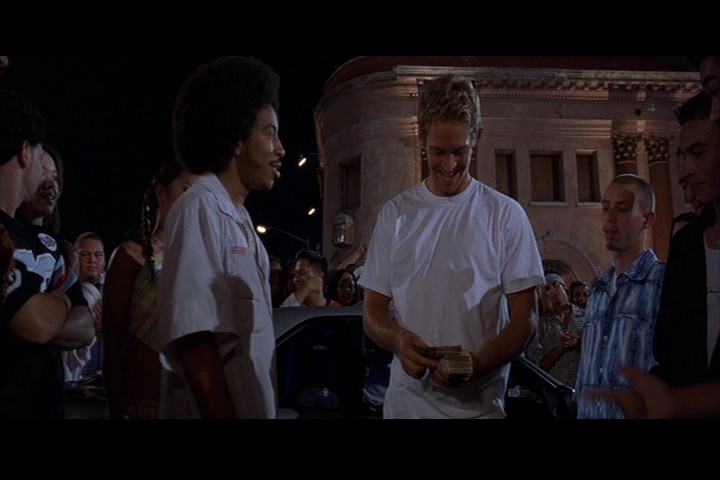

Supplement: S1 Dataset — (ZIP) [file pone.0264302.s001.zip › 2-fast-2-furious-00019871.jpg]

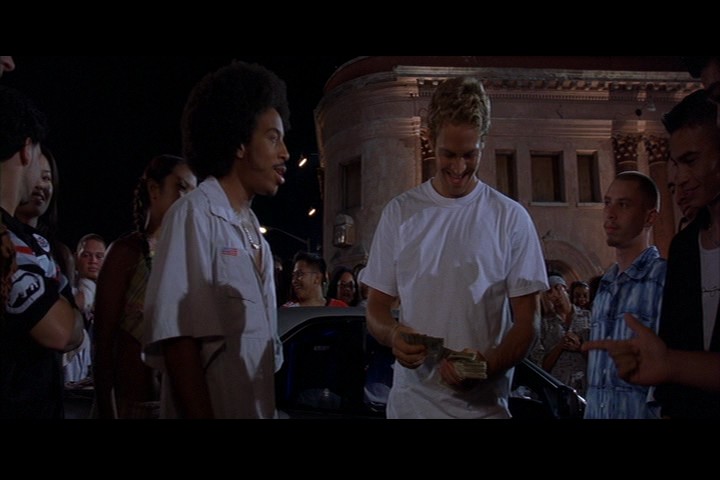

Supplement: S1 Dataset — (ZIP) [file pone.0264302.s001.zip › 2-fast-2-furious-00019881.jpg]

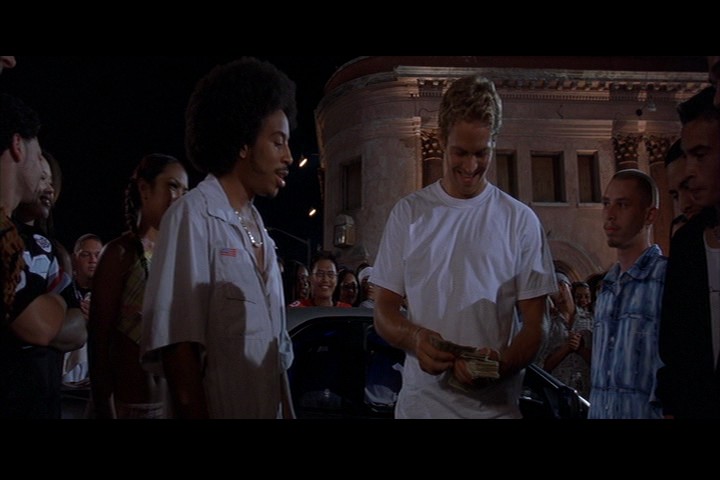

Supplement: S1 Dataset — (ZIP) [file pone.0264302.s001.zip › 2-fast-2-furious-00019891.jpg]

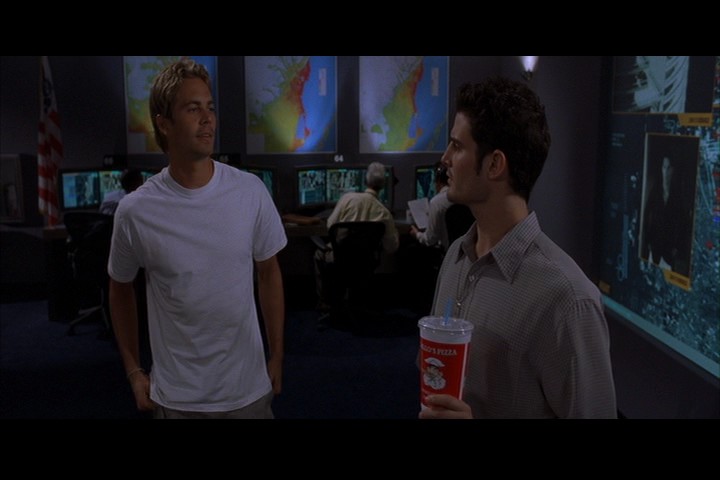

Supplement: S1 Dataset — (ZIP) [file pone.0264302.s001.zip › 2-fast-2-furious-00026221.jpg]

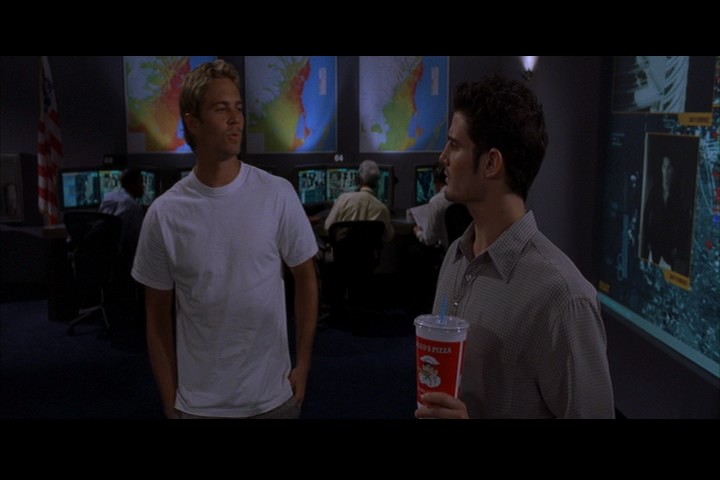

Supplement: S1 Dataset — (ZIP) [file pone.0264302.s001.zip › 2-fast-2-furious-00026231.jpg]

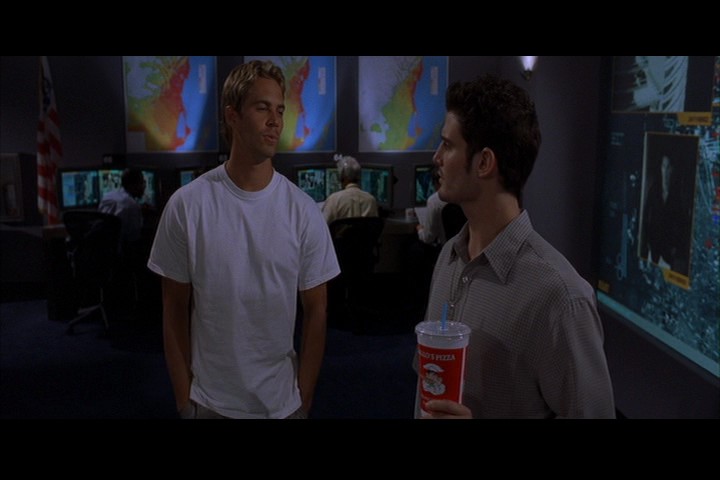

Supplement: S1 Dataset — (ZIP) [file pone.0264302.s001.zip › 2-fast-2-furious-00026251.jpg]

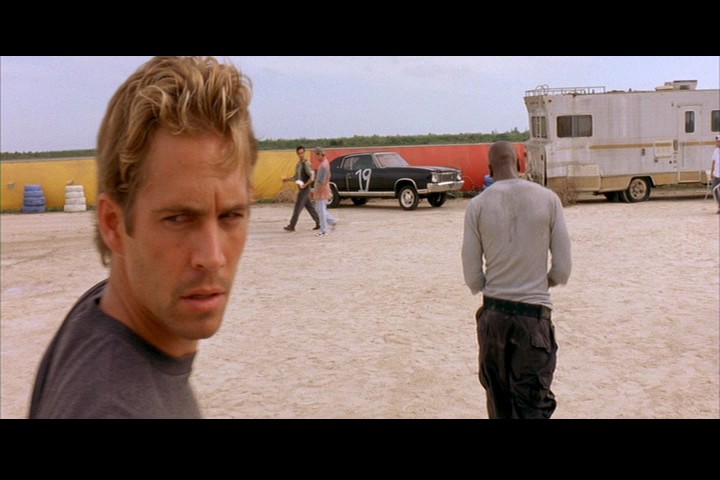

Supplement: S1 Dataset — (ZIP) [file pone.0264302.s001.zip › 2-fast-2-furious-00029661.jpg]

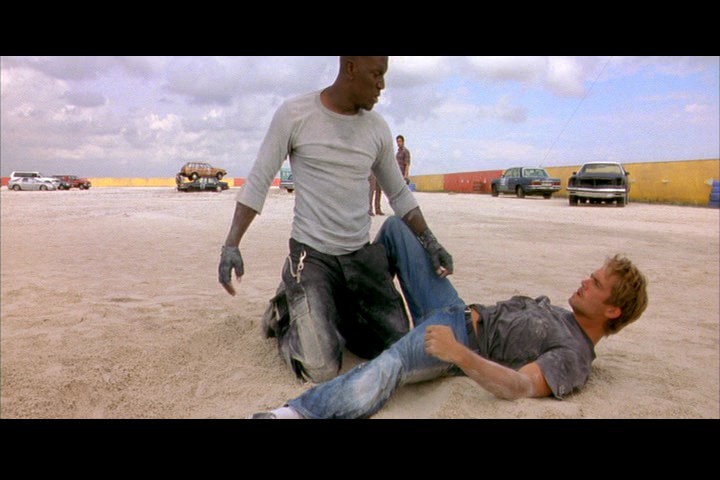

Supplement: S1 Dataset — (ZIP) [file pone.0264302.s001.zip › 2-fast-2-furious-00031661.jpg]

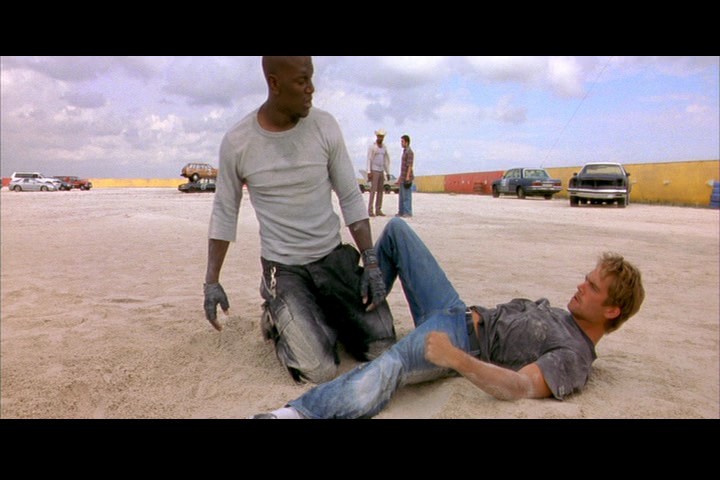

Supplement: S1 Dataset — (ZIP) [file pone.0264302.s001.zip › 2-fast-2-furious-00031691.jpg]

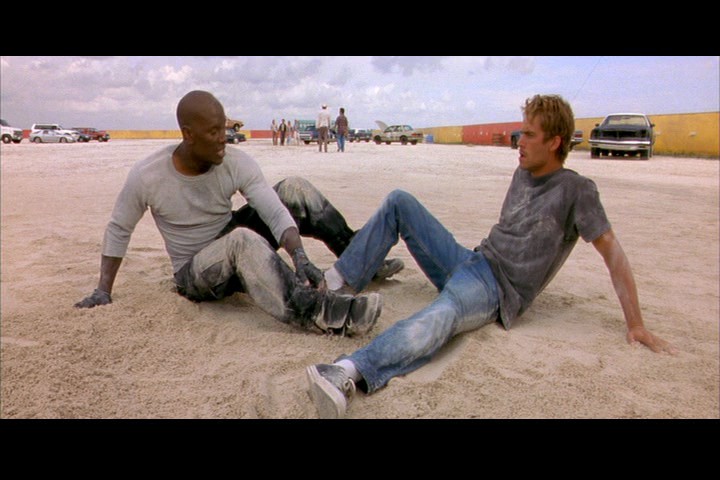

Supplement: S1 Dataset — (ZIP) [file pone.0264302.s001.zip › 2-fast-2-furious-00032131.jpg]

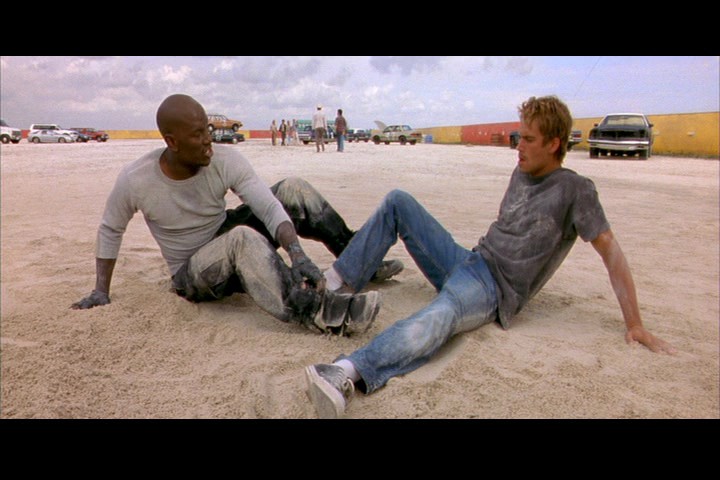

Supplement: S1 Dataset — (ZIP) [file pone.0264302.s001.zip › 2-fast-2-furious-00032141.jpg]

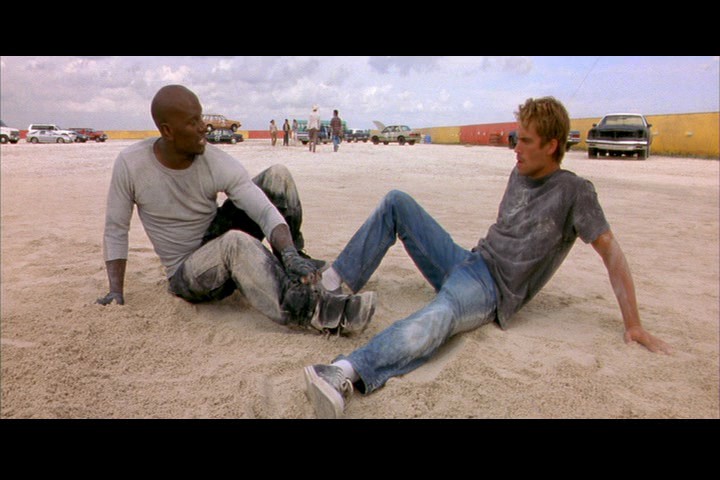

Supplement: S1 Dataset — (ZIP) [file pone.0264302.s001.zip › 2-fast-2-furious-00032161.jpg]

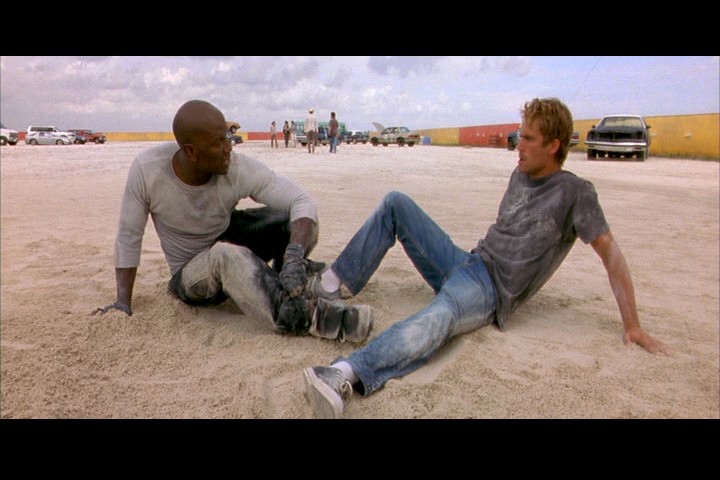

Supplement: S1 Dataset — (ZIP) [file pone.0264302.s001.zip › 2-fast-2-furious-00032171.jpg]

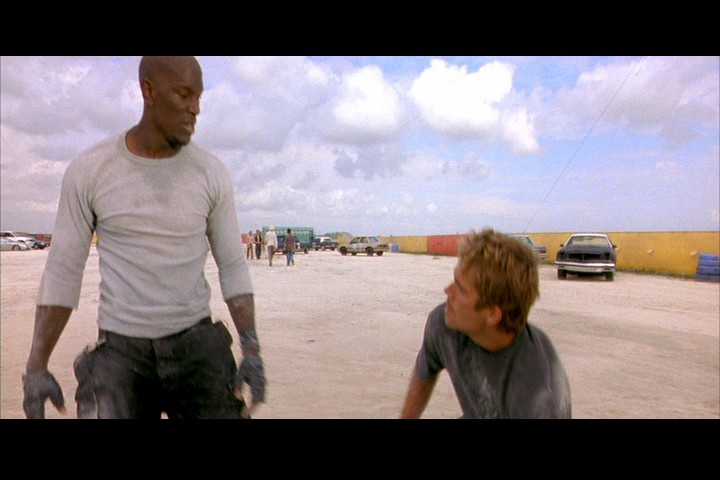

Supplement: S1 Dataset — (ZIP) [file pone.0264302.s001.zip › 2-fast-2-furious-00032241.jpg]

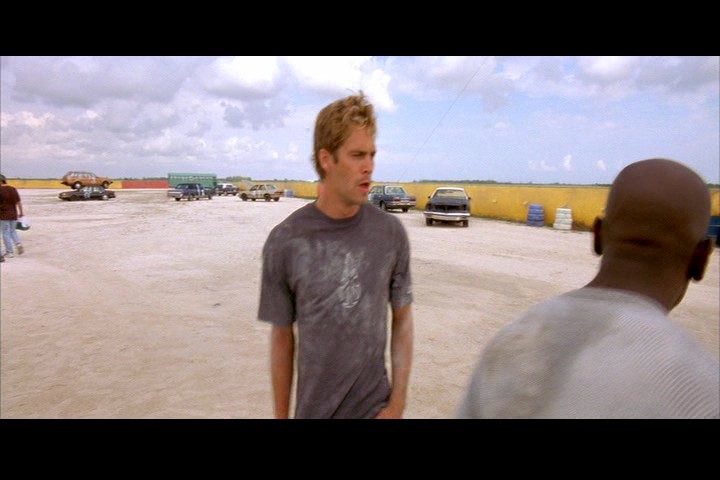

Supplement: S1 Dataset — (ZIP) [file pone.0264302.s001.zip › 2-fast-2-furious-00032651.jpg]

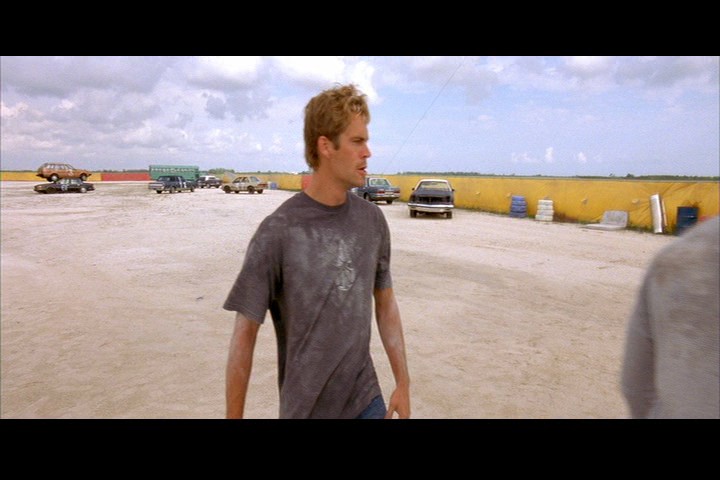

Supplement: S1 Dataset — (ZIP) [file pone.0264302.s001.zip › 2-fast-2-furious-00032661.jpg]

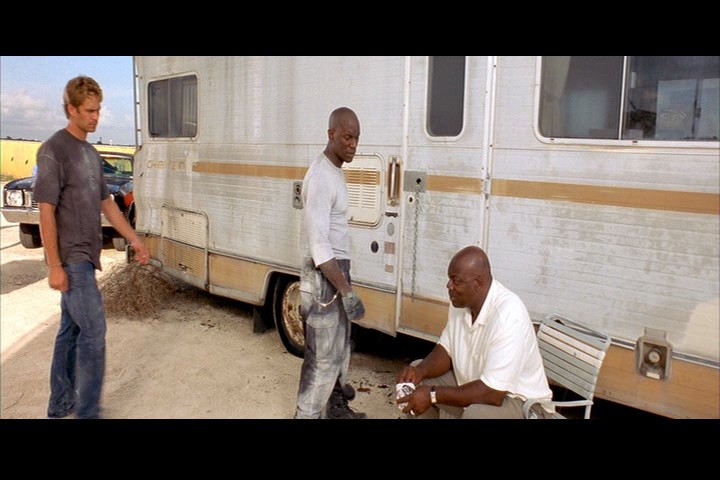

Supplement: S1 Dataset — (ZIP) [file pone.0264302.s001.zip › 2-fast-2-furious-00032761.jpg]

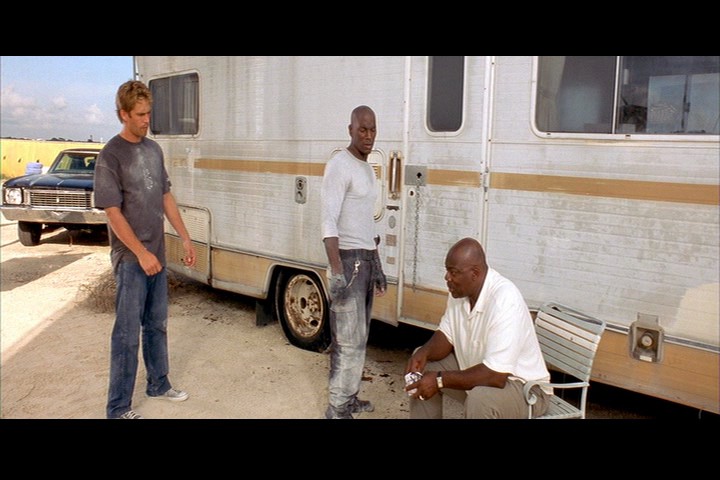

Supplement: S1 Dataset — (ZIP) [file pone.0264302.s001.zip › 2-fast-2-furious-00032781.jpg]

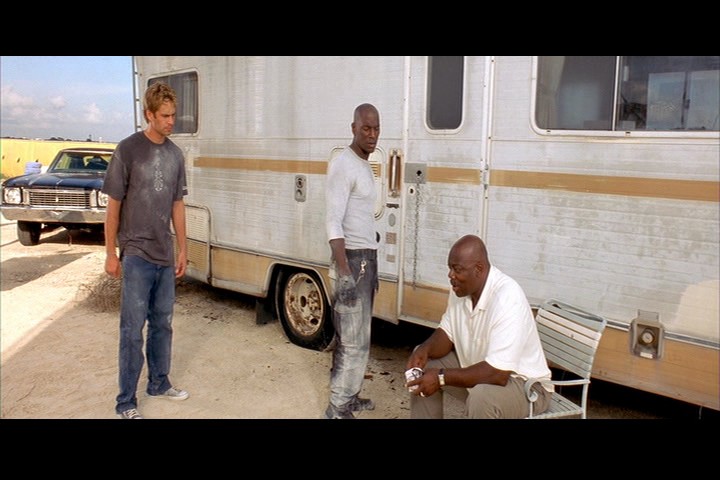

Supplement: S1 Dataset — (ZIP) [file pone.0264302.s001.zip › 2-fast-2-furious-00032791.jpg]

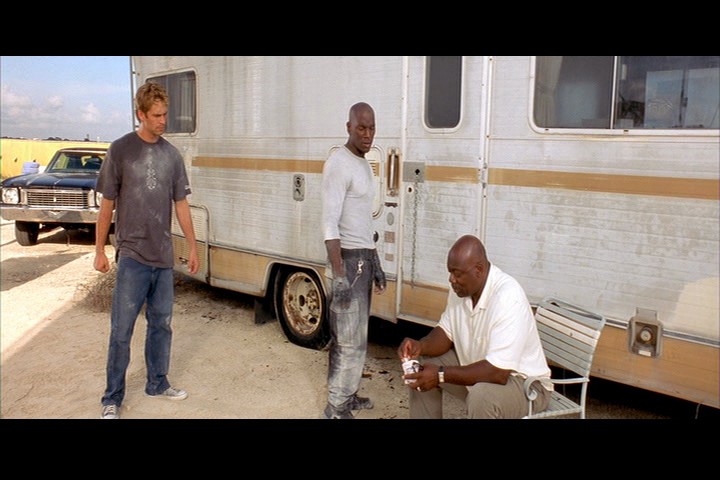

Supplement: S1 Dataset — (ZIP) [file pone.0264302.s001.zip › 2-fast-2-furious-00032801.jpg]

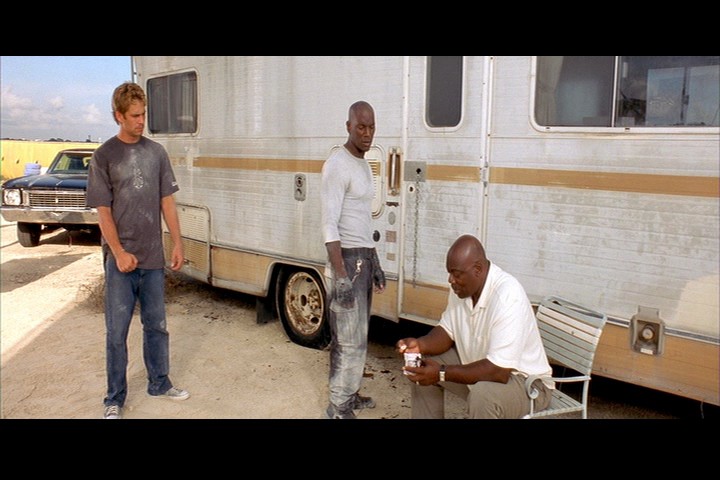

Supplement: S1 Dataset — (ZIP) [file pone.0264302.s001.zip › 2-fast-2-furious-00032811.jpg]

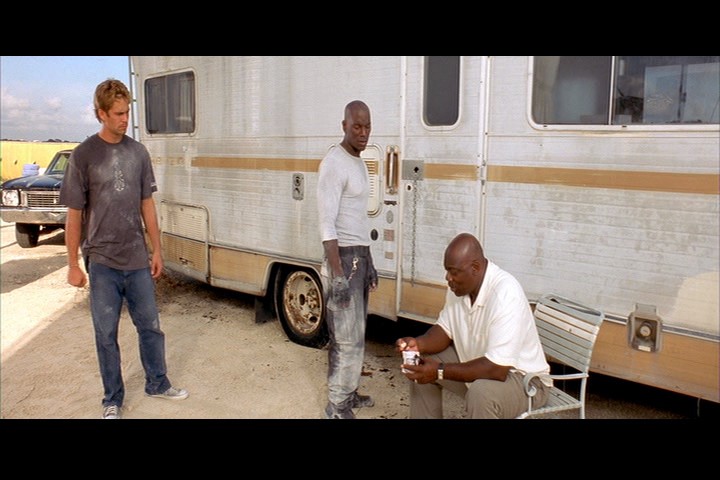

Supplement: S1 Dataset — (ZIP) [file pone.0264302.s001.zip › 2-fast-2-furious-00032821.jpg]

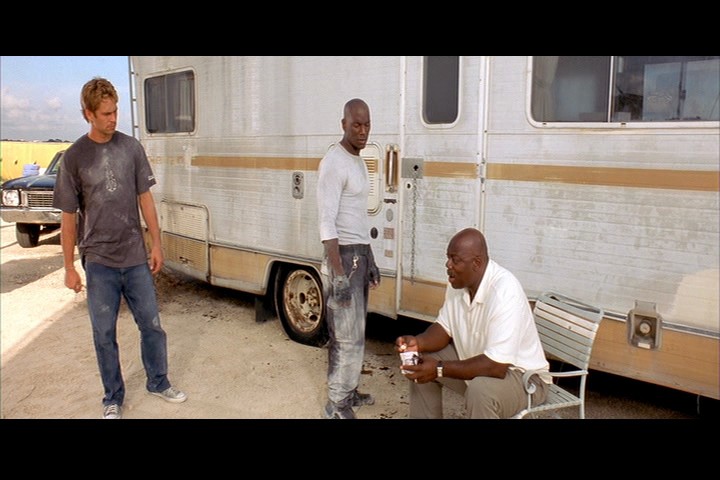

Supplement: S1 Dataset — (ZIP) [file pone.0264302.s001.zip › 2-fast-2-furious-00032831.jpg]

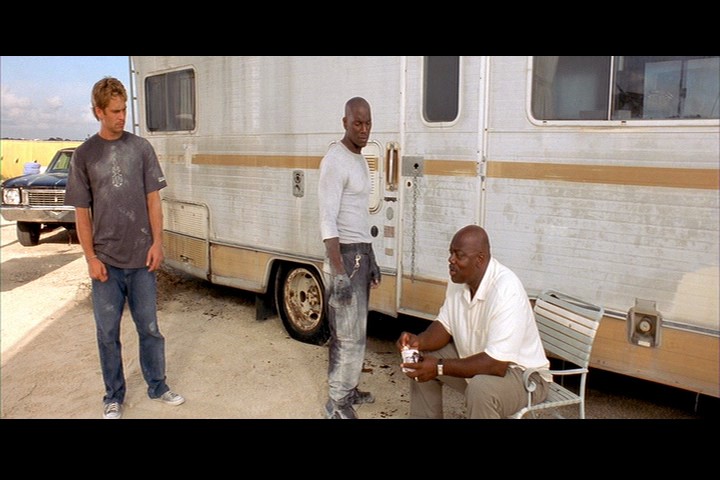

Supplement: S1 Dataset — (ZIP) [file pone.0264302.s001.zip › 2-fast-2-furious-00032841.jpg]

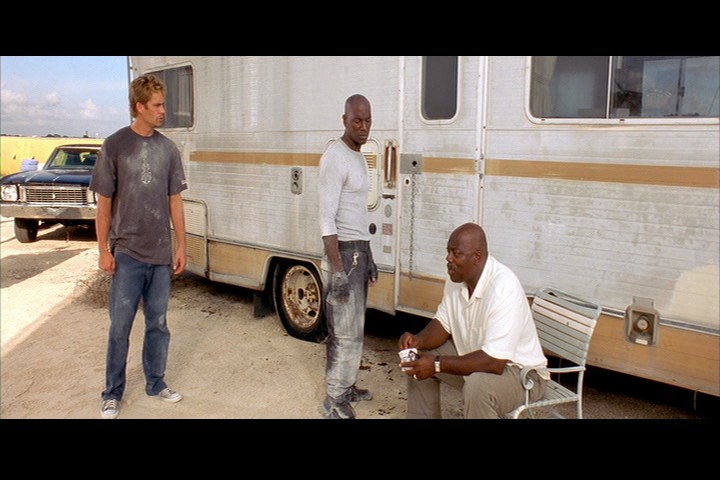

Supplement: S1 Dataset — (ZIP) [file pone.0264302.s001.zip › 2-fast-2-furious-00032861.jpg]

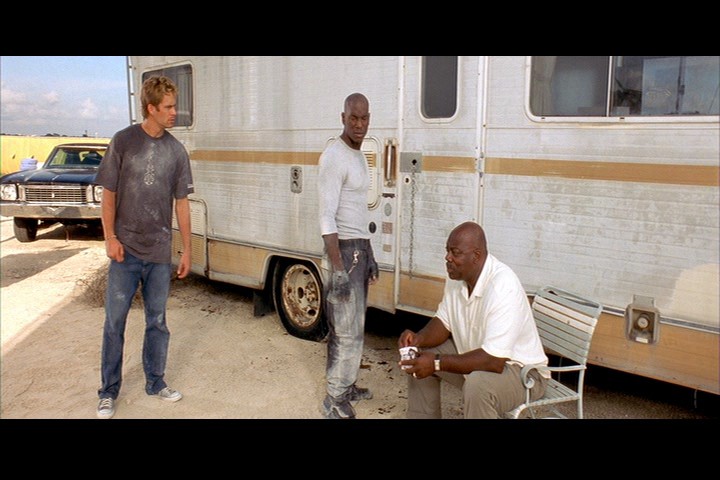

Supplement: S1 Dataset — (ZIP) [file pone.0264302.s001.zip › 2-fast-2-furious-00032871.jpg]

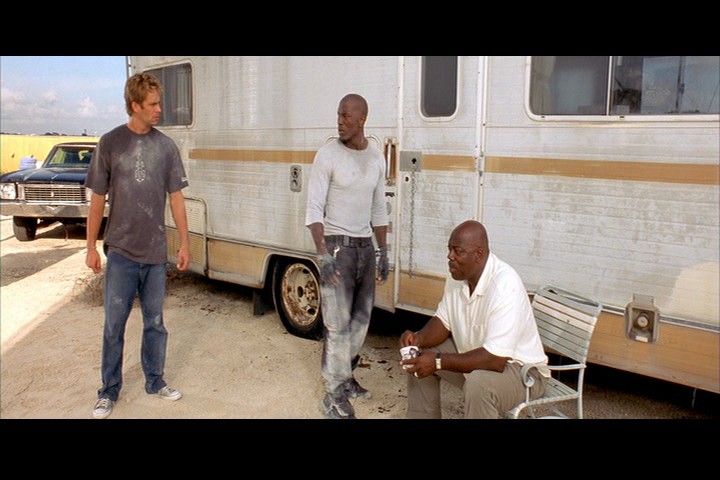

Supplement: S1 Dataset — (ZIP) [file pone.0264302.s001.zip › 2-fast-2-furious-00032881.jpg]

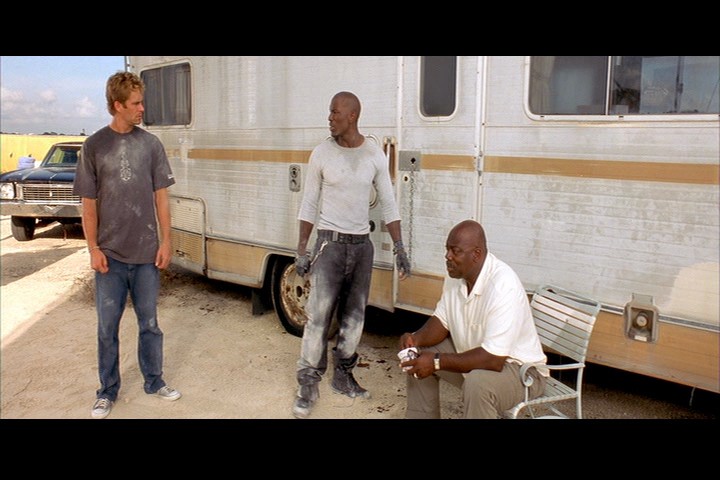

Supplement: S1 Dataset — (ZIP) [file pone.0264302.s001.zip › 2-fast-2-furious-00032891.jpg]

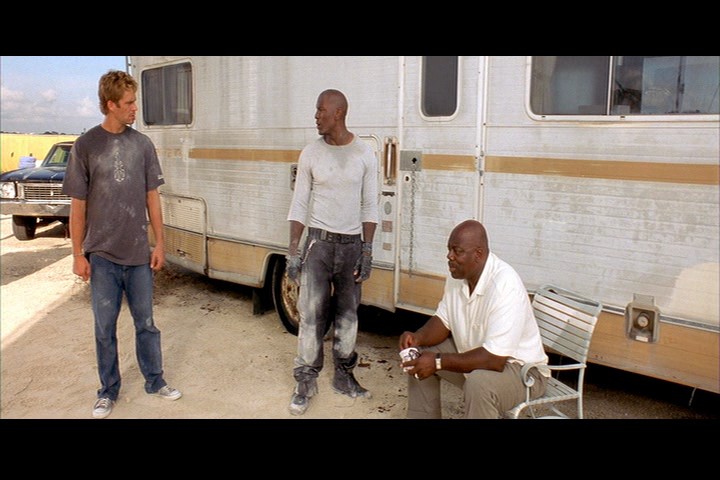

Supplement: S1 Dataset — (ZIP) [file pone.0264302.s001.zip › 2-fast-2-furious-00032901.jpg]

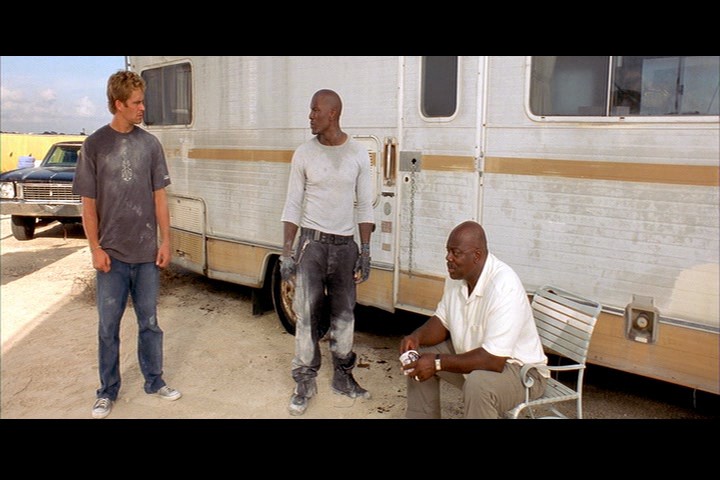

Supplement: S1 Dataset — (ZIP) [file pone.0264302.s001.zip › 2-fast-2-furious-00032921.jpg]

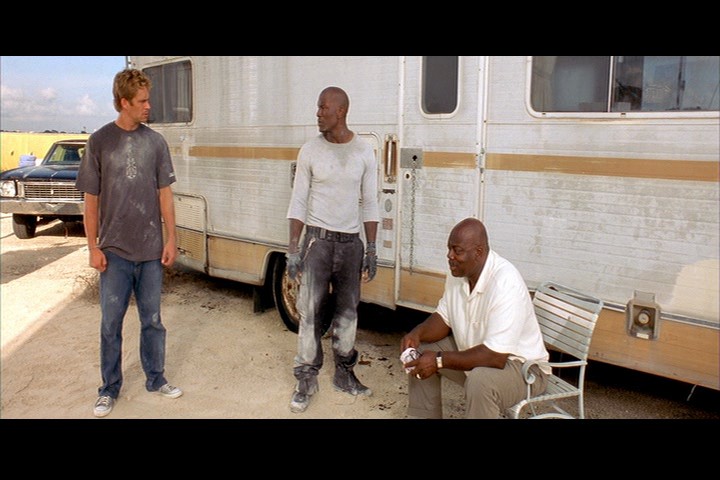

Supplement: S1 Dataset — (ZIP) [file pone.0264302.s001.zip › 2-fast-2-furious-00032941.jpg]

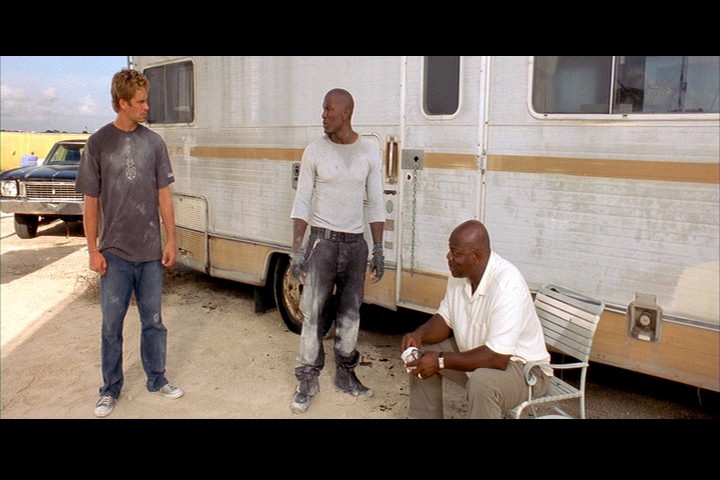

Supplement: S1 Dataset — (ZIP) [file pone.0264302.s001.zip › 2-fast-2-furious-00032951.jpg]

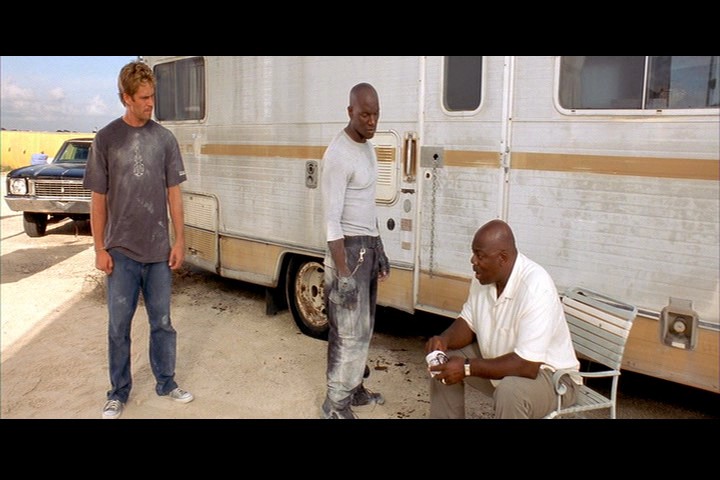

Supplement: S1 Dataset — (ZIP) [file pone.0264302.s001.zip › 2-fast-2-furious-00033091.jpg]

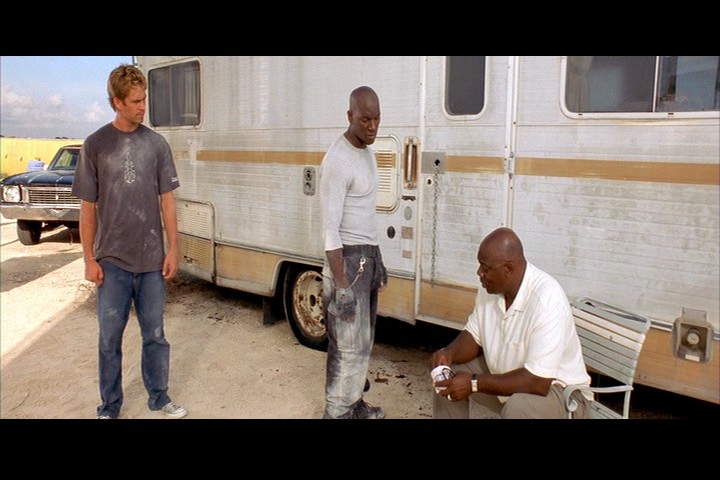

Supplement: S1 Dataset — (ZIP) [file pone.0264302.s001.zip › 2-fast-2-furious-00033101.jpg]

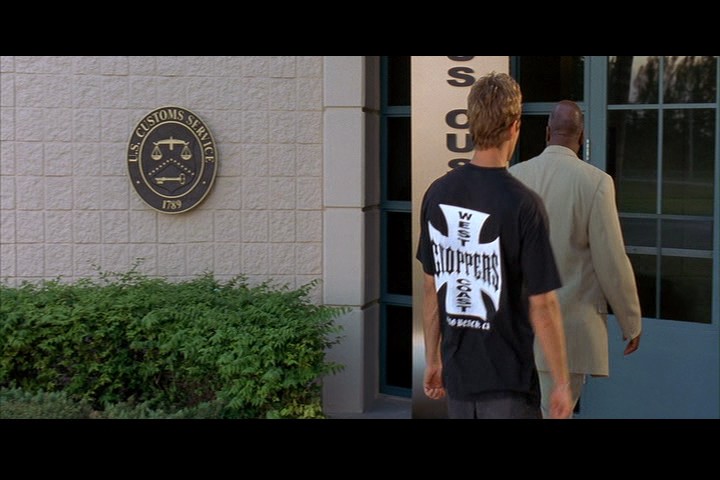

Supplement: S1 Dataset — (ZIP) [file pone.0264302.s001.zip › 2-fast-2-furious-00033961.jpg]

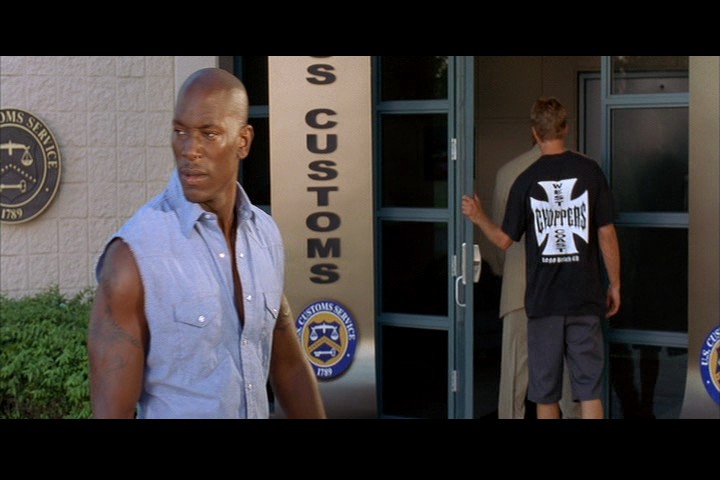

Supplement: S1 Dataset — (ZIP) [file pone.0264302.s001.zip › 2-fast-2-furious-00034031.jpg]

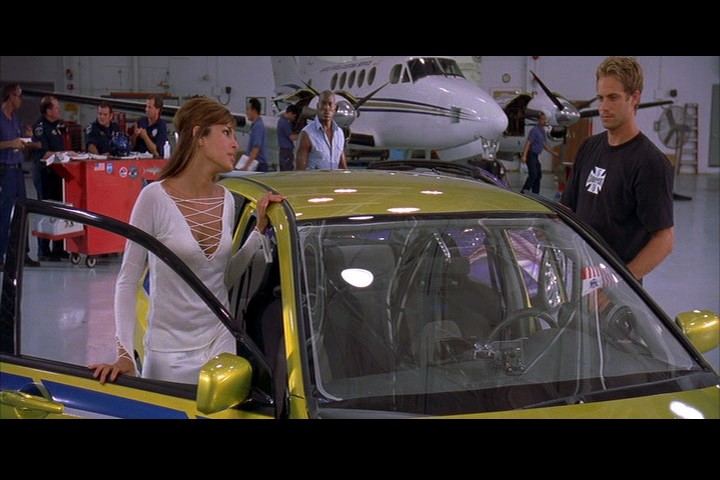

Supplement: S1 Dataset — (ZIP) [file pone.0264302.s001.zip › 2-fast-2-furious-00036661.jpg]

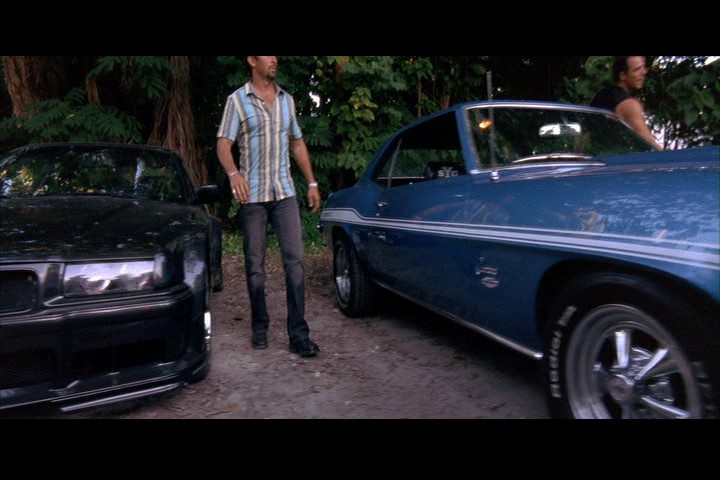

Supplement: S1 Dataset — (ZIP) [file pone.0264302.s001.zip › 2-fast-2-furious-00038791.jpg]

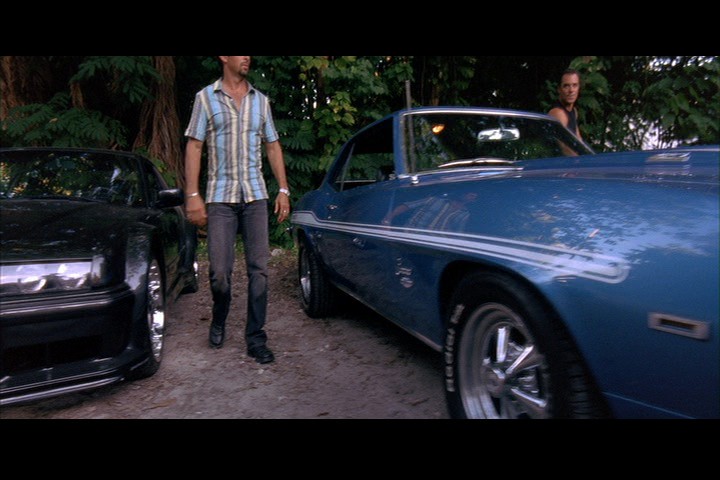

Supplement: S1 Dataset — (ZIP) [file pone.0264302.s001.zip › 2-fast-2-furious-00038801.jpg]

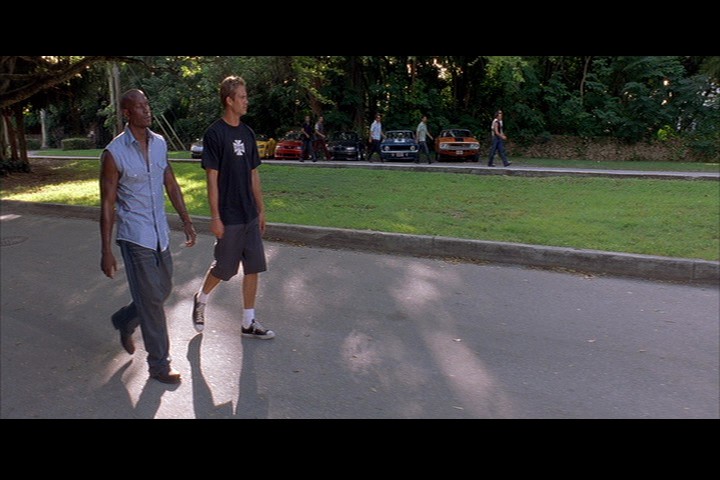

Supplement: S1 Dataset — (ZIP) [file pone.0264302.s001.zip › 2-fast-2-furious-00040181.jpg]

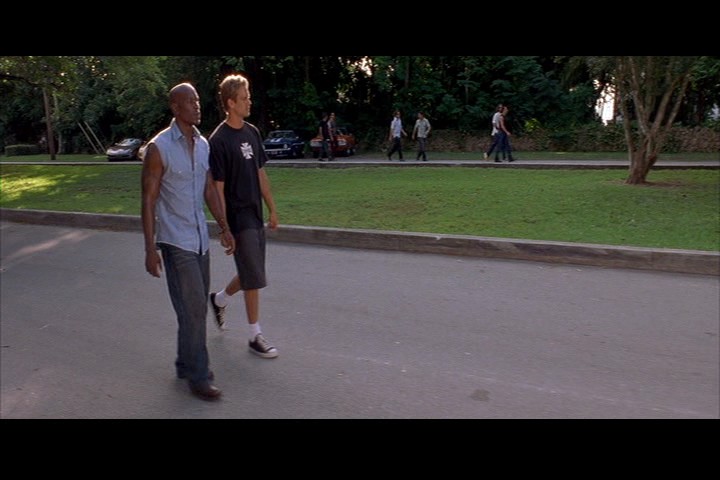

Supplement: S1 Dataset — (ZIP) [file pone.0264302.s001.zip › 2-fast-2-furious-00040321.jpg]

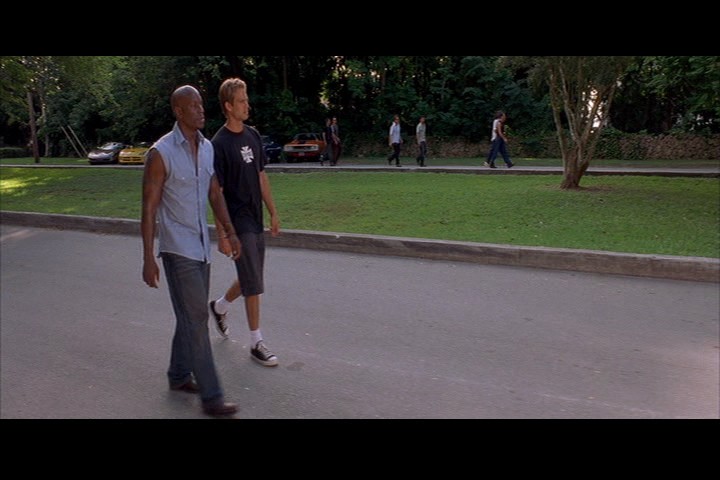

Supplement: S1 Dataset — (ZIP) [file pone.0264302.s001.zip › 2-fast-2-furious-00040351.jpg]

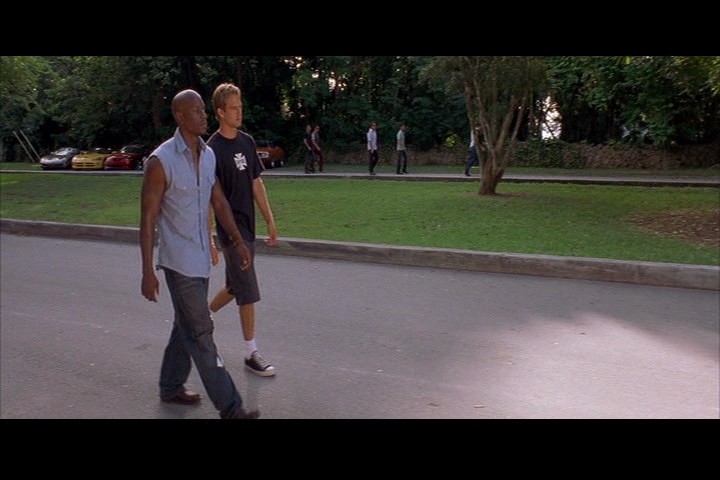

Supplement: S1 Dataset — (ZIP) [file pone.0264302.s001.zip › 2-fast-2-furious-00040381.jpg]

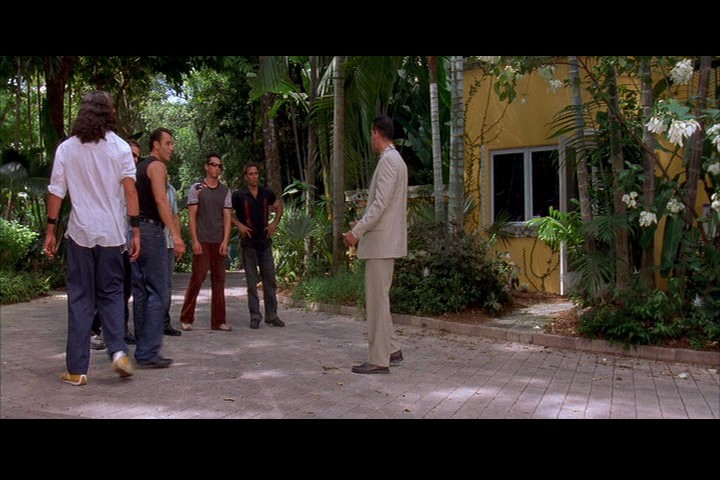

Supplement: S1 Dataset — (ZIP) [file pone.0264302.s001.zip › 2-fast-2-furious-00040401.jpg]

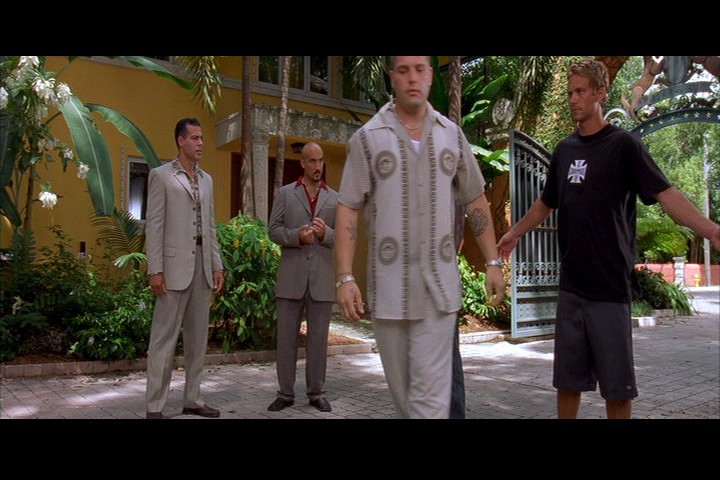

Supplement: S1 Dataset — (ZIP) [file pone.0264302.s001.zip › 2-fast-2-furious-00040591.jpg]

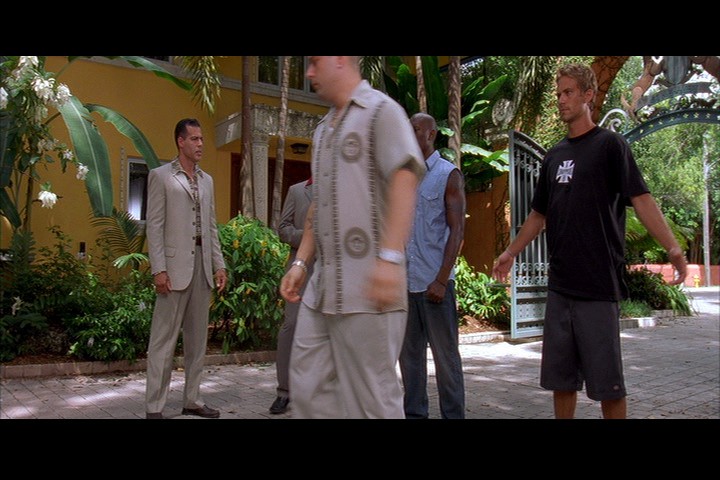

Supplement: S1 Dataset — (ZIP) [file pone.0264302.s001.zip › 2-fast-2-furious-00040601.jpg]

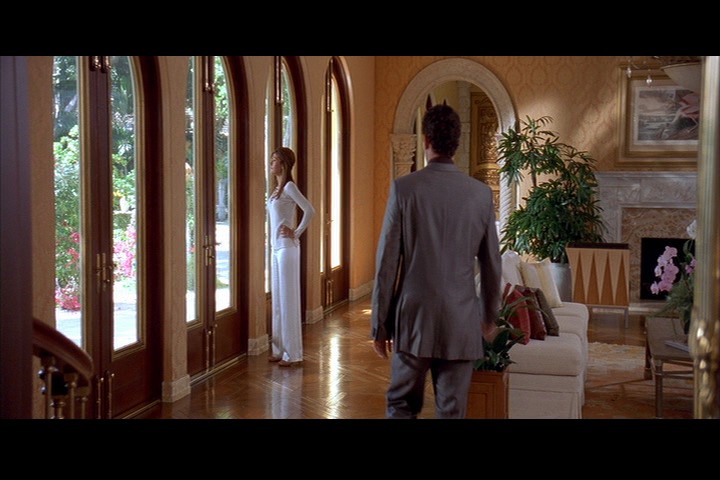

Supplement: S1 Dataset — (ZIP) [file pone.0264302.s001.zip › 2-fast-2-furious-00041081.jpg]

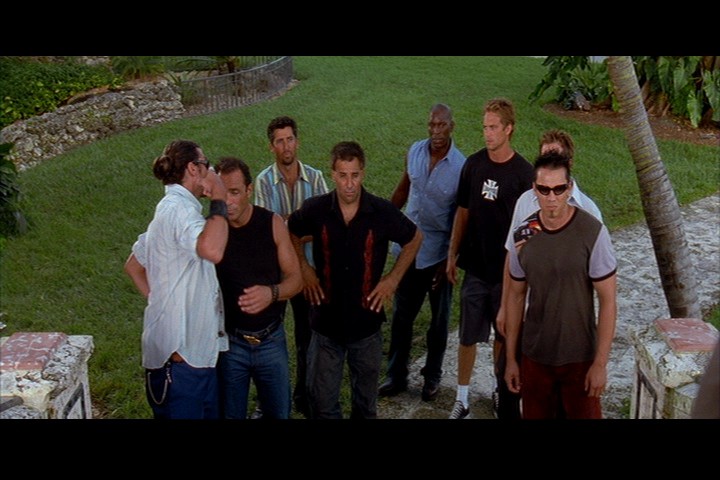

Supplement: S1 Dataset — (ZIP) [file pone.0264302.s001.zip › 2-fast-2-furious-00041381.jpg]

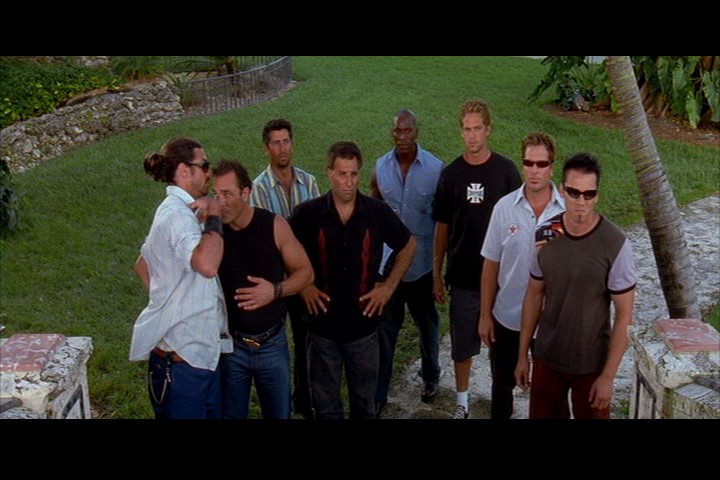

Supplement: S1 Dataset — (ZIP) [file pone.0264302.s001.zip › 2-fast-2-furious-00041391.jpg]

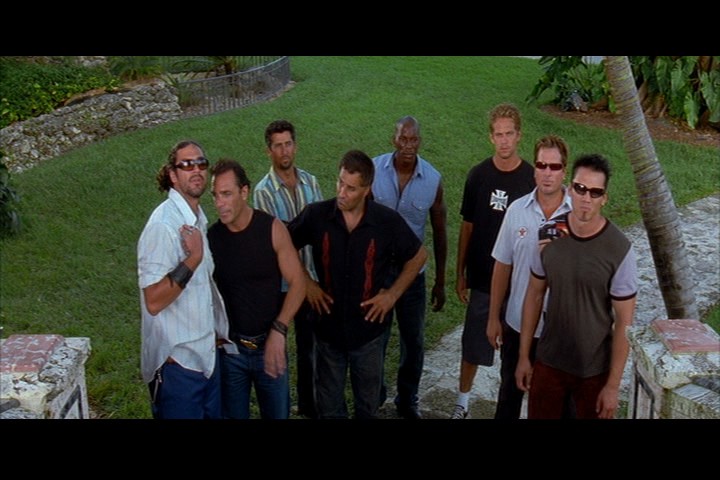

Supplement: S1 Dataset — (ZIP) [file pone.0264302.s001.zip › 2-fast-2-furious-00041421.jpg]

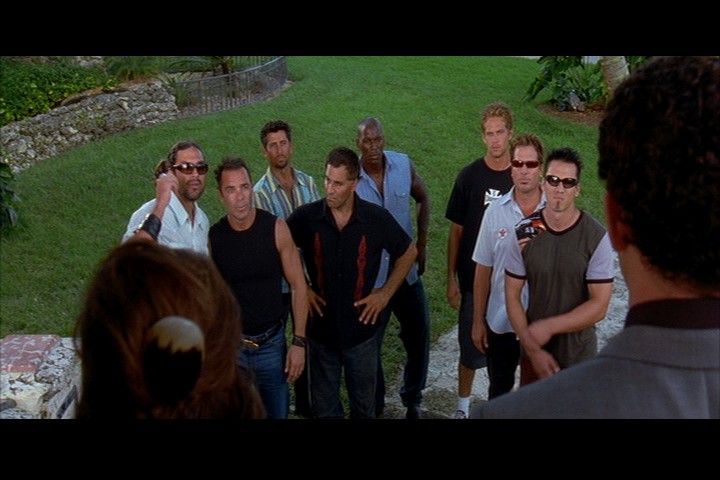

Supplement: S1 Dataset — (ZIP) [file pone.0264302.s001.zip › 2-fast-2-furious-00041441.jpg]

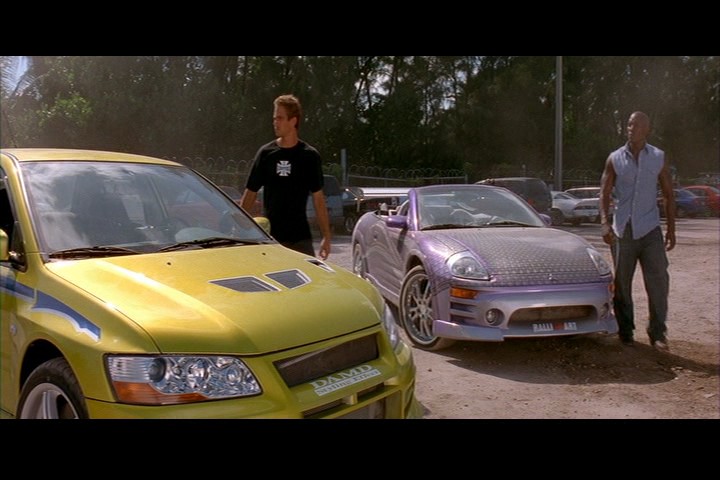

Supplement: S1 Dataset — (ZIP) [file pone.0264302.s001.zip › 2-fast-2-furious-00048861.jpg]

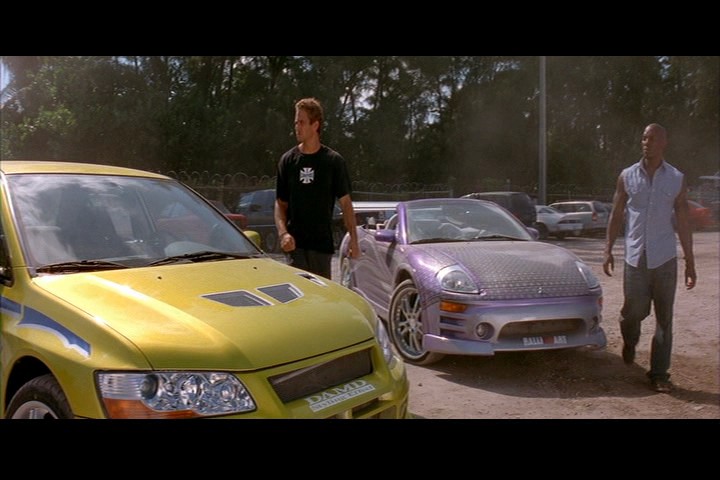

Supplement: S1 Dataset — (ZIP) [file pone.0264302.s001.zip › 2-fast-2-furious-00048871.jpg]

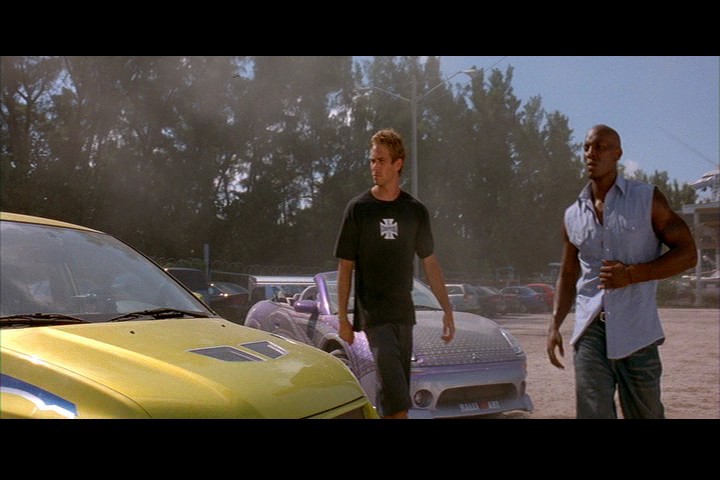

Supplement: S1 Dataset — (ZIP) [file pone.0264302.s001.zip › 2-fast-2-furious-00048911.jpg]

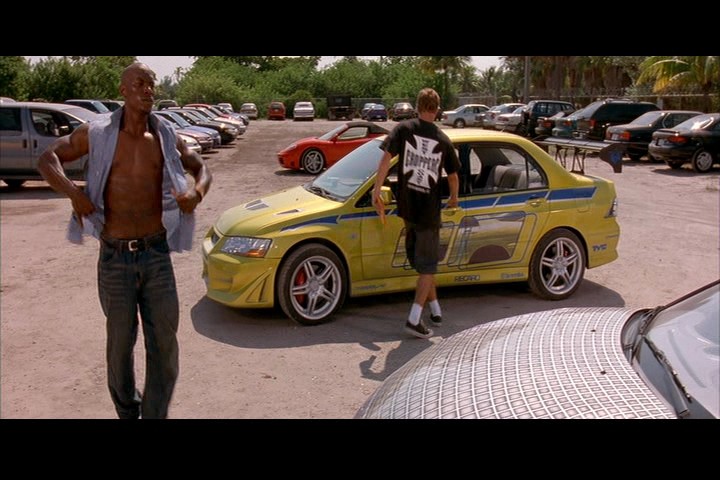

Supplement: S1 Dataset — (ZIP) [file pone.0264302.s001.zip › 2-fast-2-furious-00049621.jpg]

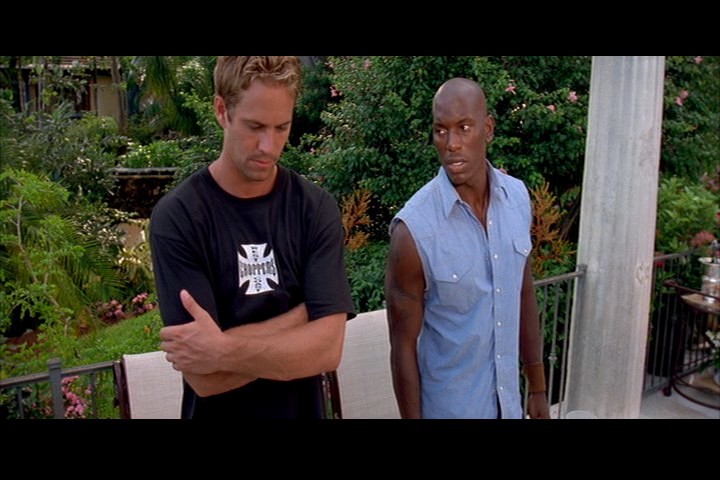

Supplement: S1 Dataset — (ZIP) [file pone.0264302.s001.zip › 2-fast-2-furious-00051901.jpg]

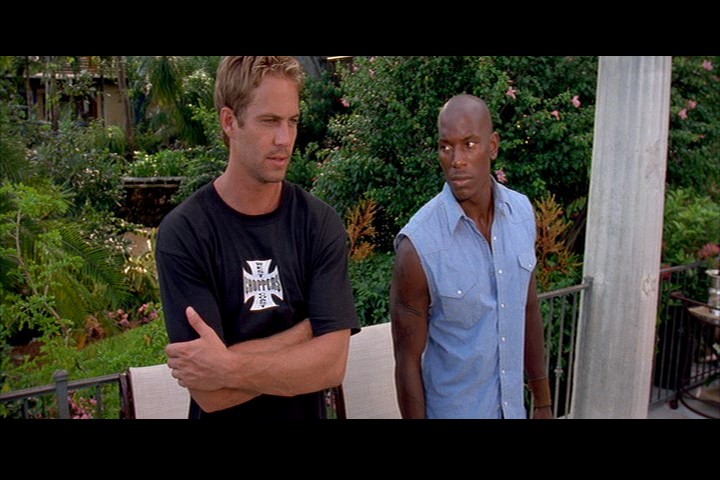

Supplement: S1 Dataset — (ZIP) [file pone.0264302.s001.zip › 2-fast-2-furious-00051941.jpg]

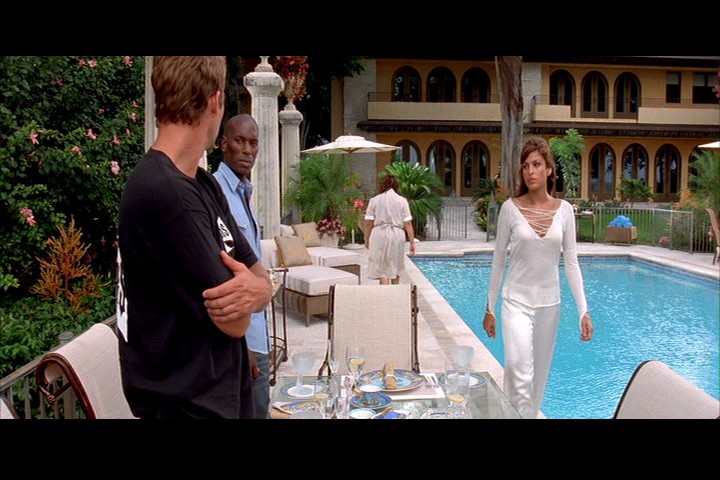

Supplement: S1 Dataset — (ZIP) [file pone.0264302.s001.zip › 2-fast-2-furious-00052081.jpg]

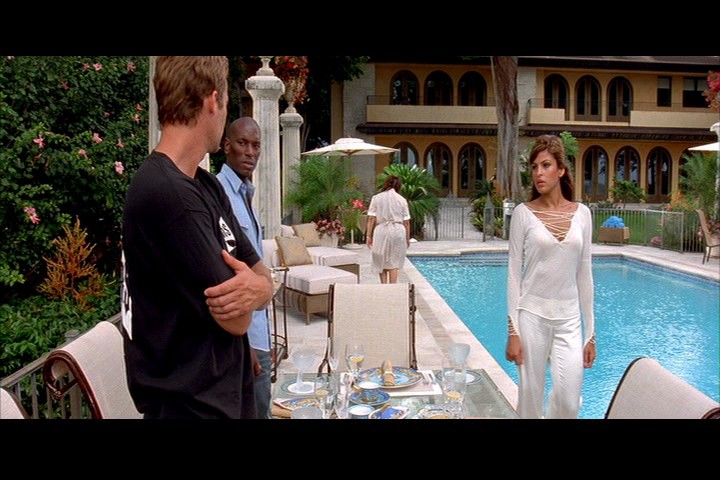

Supplement: S1 Dataset — (ZIP) [file pone.0264302.s001.zip › 2-fast-2-furious-00052091.jpg]

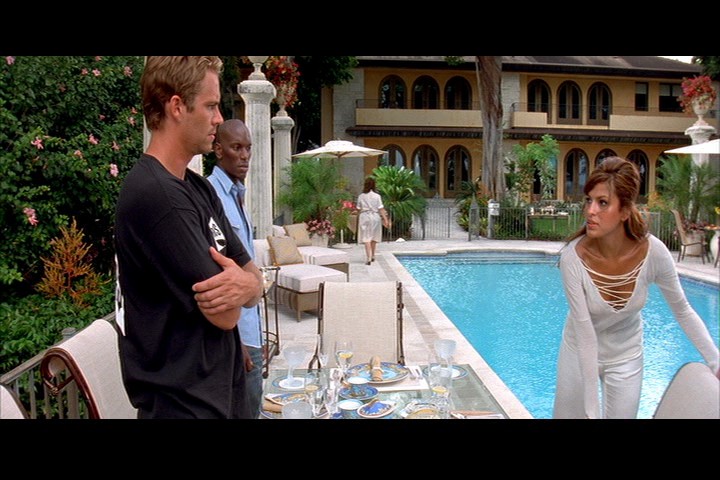

Supplement: S1 Dataset — (ZIP) [file pone.0264302.s001.zip › 2-fast-2-furious-00052181.jpg]

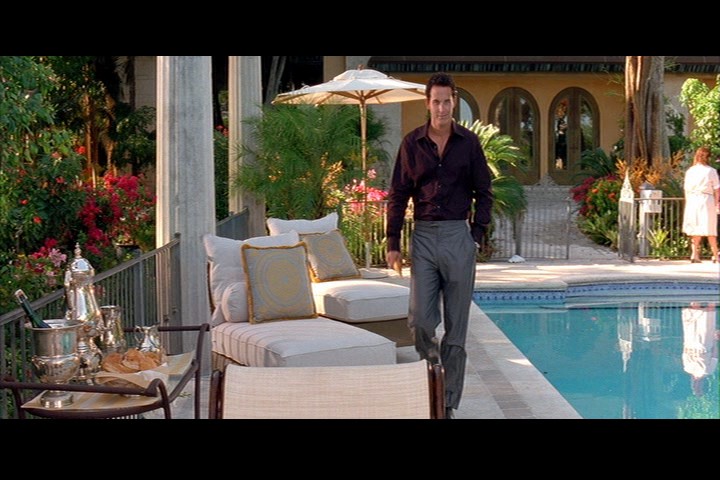

Supplement: S1 Dataset — (ZIP) [file pone.0264302.s001.zip › 2-fast-2-furious-00052721.jpg]

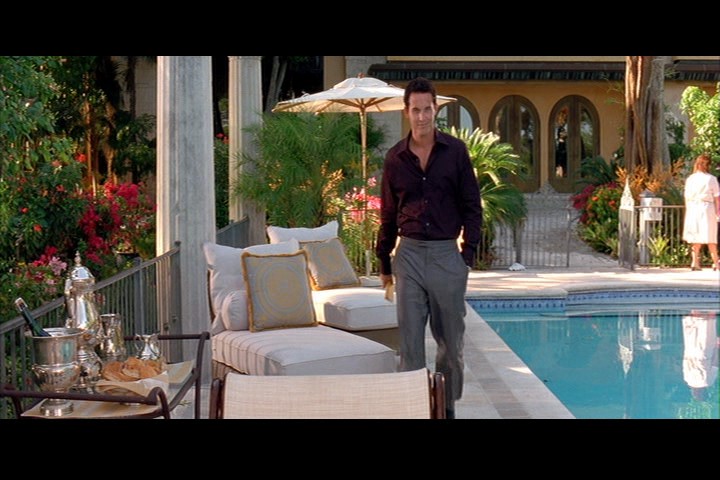

Supplement: S1 Dataset — (ZIP) [file pone.0264302.s001.zip › 2-fast-2-furious-00052731.jpg]

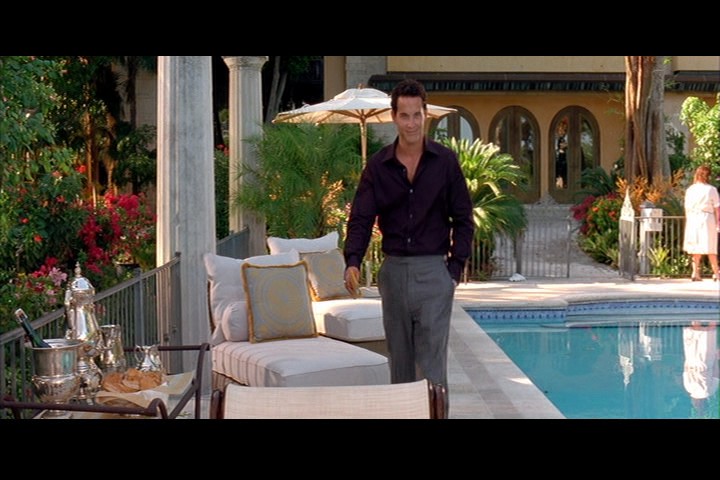

Supplement: S1 Dataset — (ZIP) [file pone.0264302.s001.zip › 2-fast-2-furious-00052741.jpg]

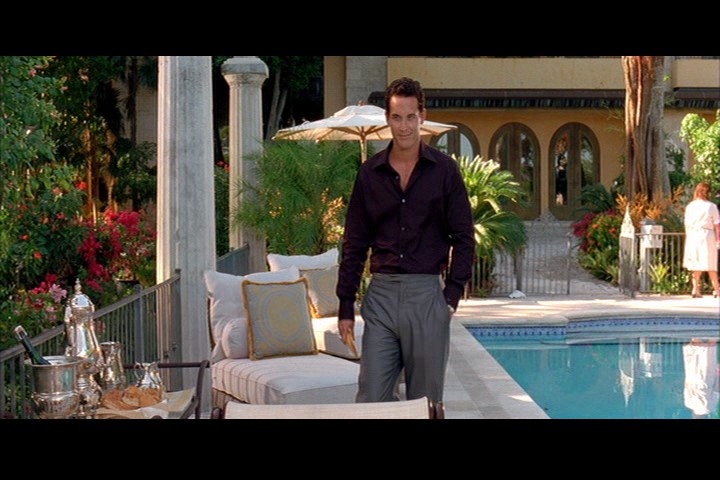

Supplement: S1 Dataset — (ZIP) [file pone.0264302.s001.zip › 2-fast-2-furious-00052751.jpg]

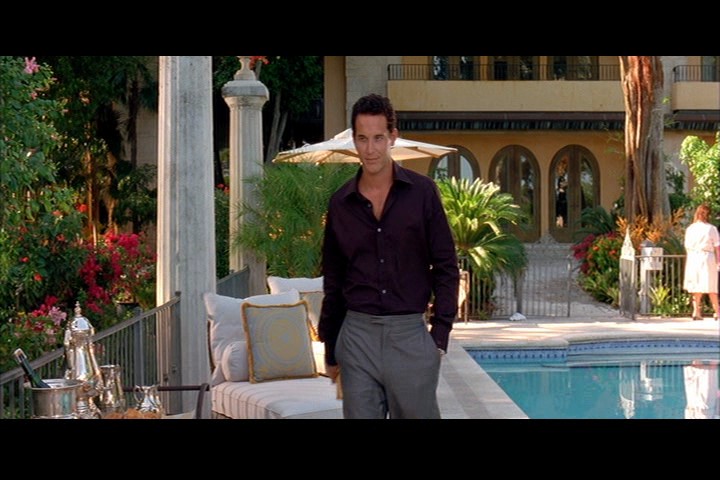

Supplement: S1 Dataset — (ZIP) [file pone.0264302.s001.zip › 2-fast-2-furious-00052761.jpg]

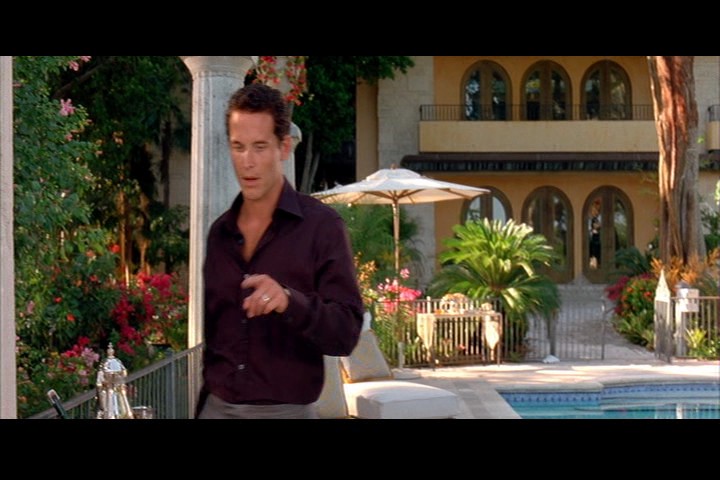

Supplement: S1 Dataset — (ZIP) [file pone.0264302.s001.zip › 2-fast-2-furious-00052791.jpg]

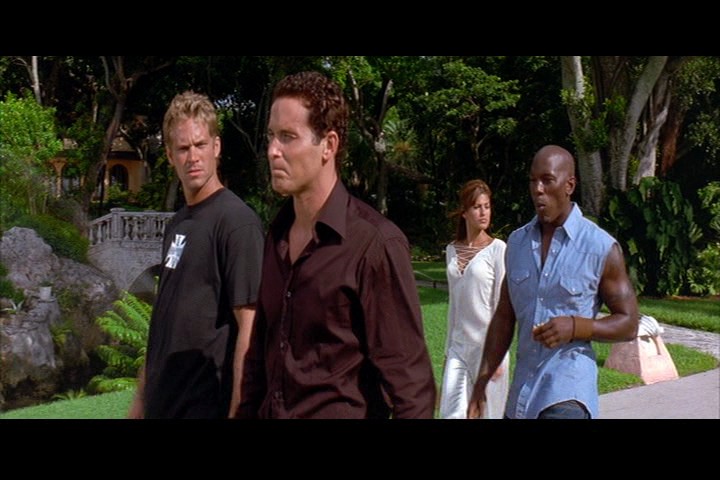

Supplement: S1 Dataset — (ZIP) [file pone.0264302.s001.zip › 2-fast-2-furious-00054501.jpg]

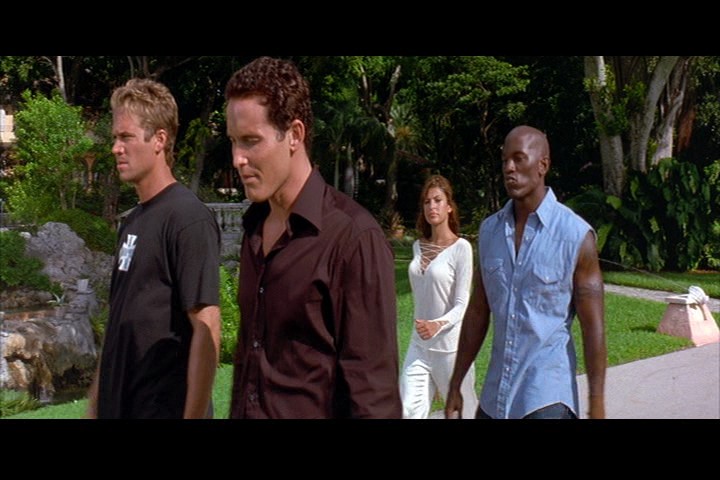

Supplement: S1 Dataset — (ZIP) [file pone.0264302.s001.zip › 2-fast-2-furious-00054531.jpg]

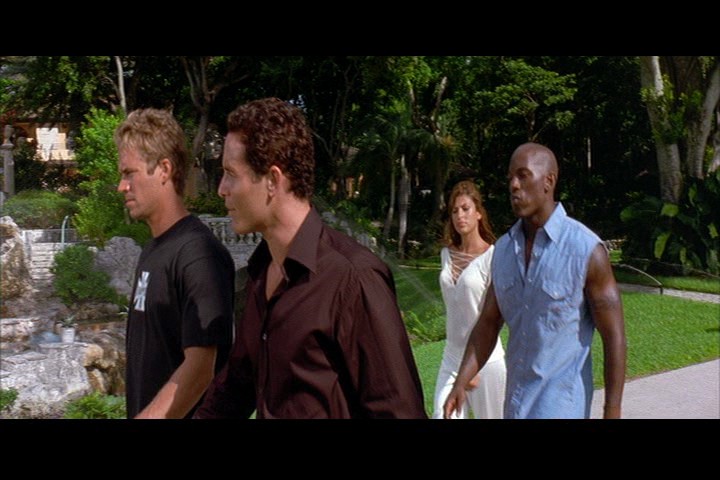

Supplement: S1 Dataset — (ZIP) [file pone.0264302.s001.zip › 2-fast-2-furious-00054551.jpg]

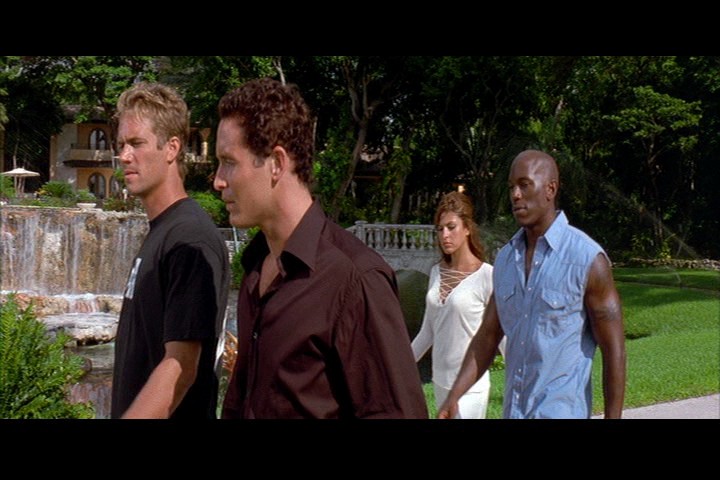

Supplement: S1 Dataset — (ZIP) [file pone.0264302.s001.zip › 2-fast-2-furious-00054641.jpg]

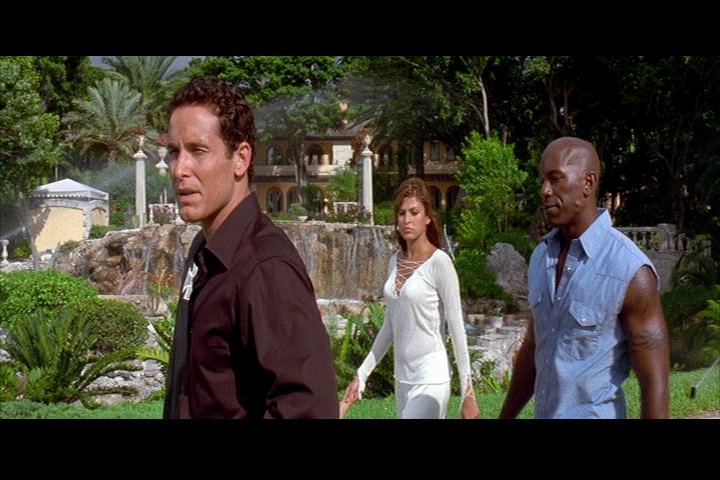

Supplement: S1 Dataset — (ZIP) [file pone.0264302.s001.zip › 2-fast-2-furious-00054741.jpg]

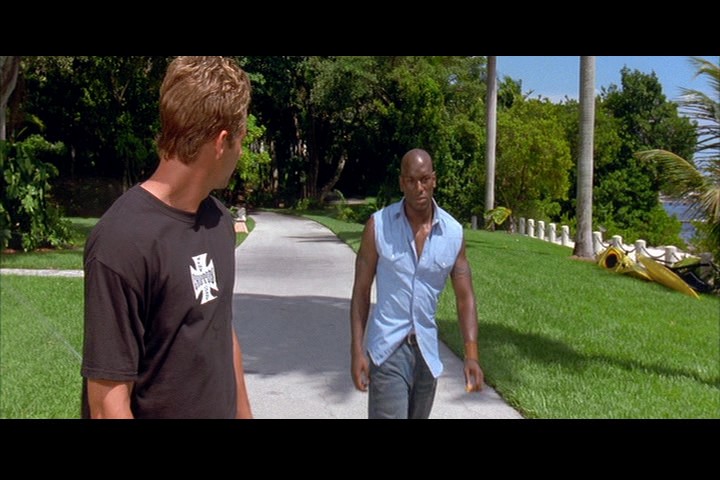

Supplement: S1 Dataset — (ZIP) [file pone.0264302.s001.zip › 2-fast-2-furious-00056401.jpg]

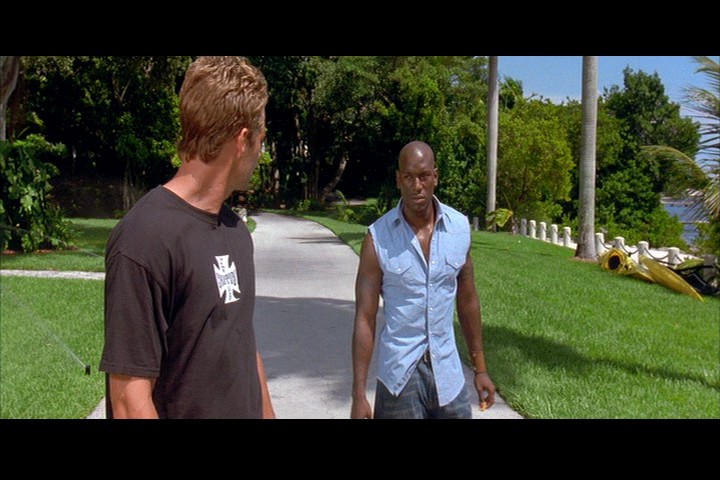

Supplement: S1 Dataset — (ZIP) [file pone.0264302.s001.zip › 2-fast-2-furious-00056411.jpg]

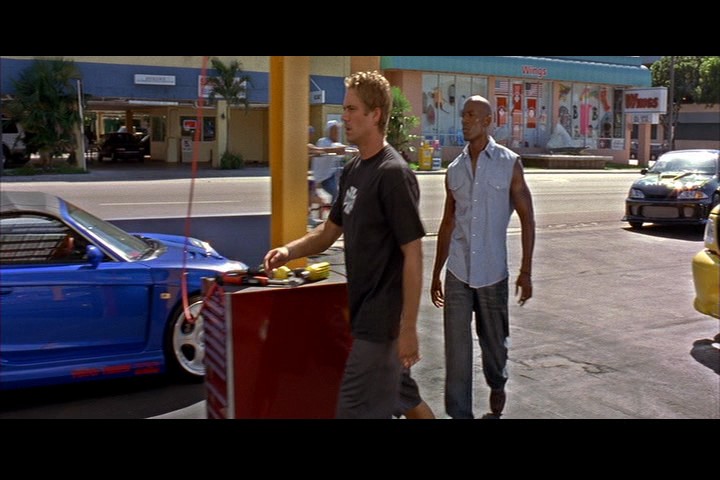

Supplement: S1 Dataset — (ZIP) [file pone.0264302.s001.zip › 2-fast-2-furious-00058101.jpg]

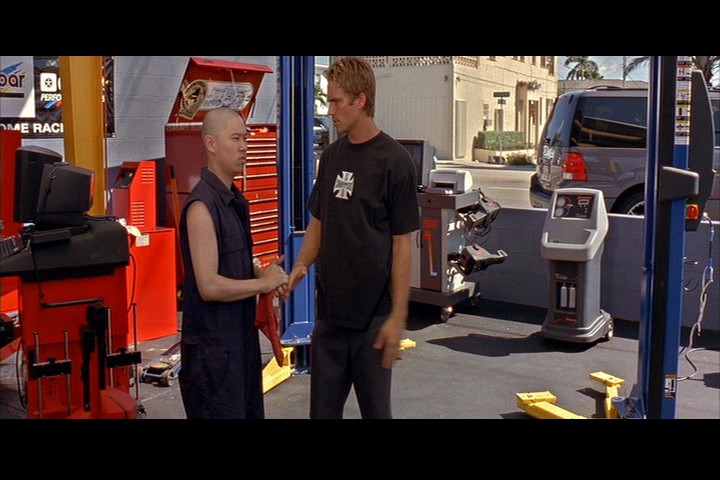

Supplement: S1 Dataset — (ZIP) [file pone.0264302.s001.zip › 2-fast-2-furious-00058291.jpg]

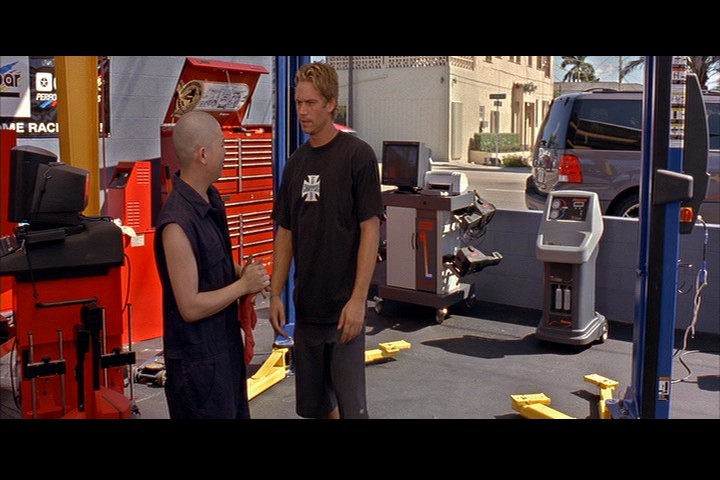

Supplement: S1 Dataset — (ZIP) [file pone.0264302.s001.zip › 2-fast-2-furious-00058321.jpg]

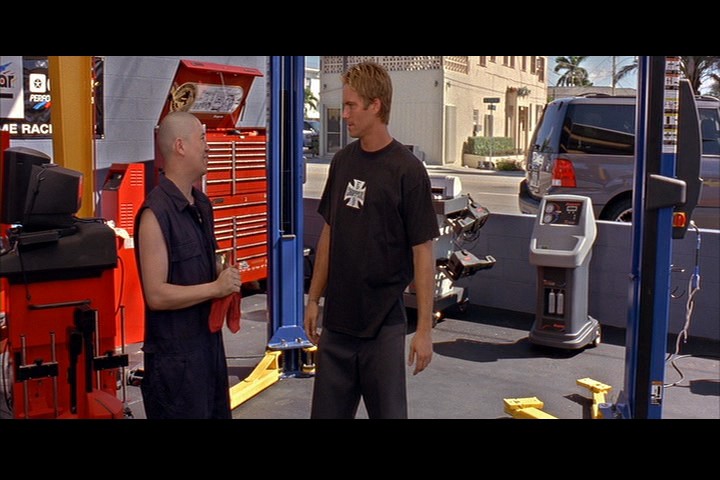

Supplement: S1 Dataset — (ZIP) [file pone.0264302.s001.zip › 2-fast-2-furious-00058351.jpg]

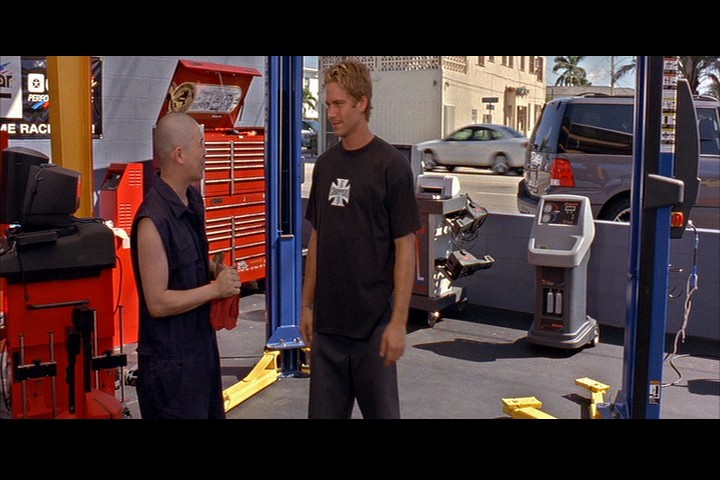

Supplement: S1 Dataset — (ZIP) [file pone.0264302.s001.zip › 2-fast-2-furious-00058361.jpg]

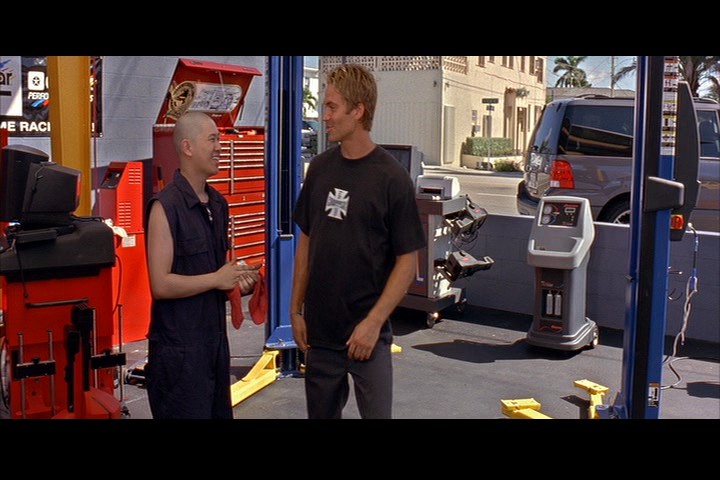

Supplement: S1 Dataset — (ZIP) [file pone.0264302.s001.zip › 2-fast-2-furious-00058381.jpg]

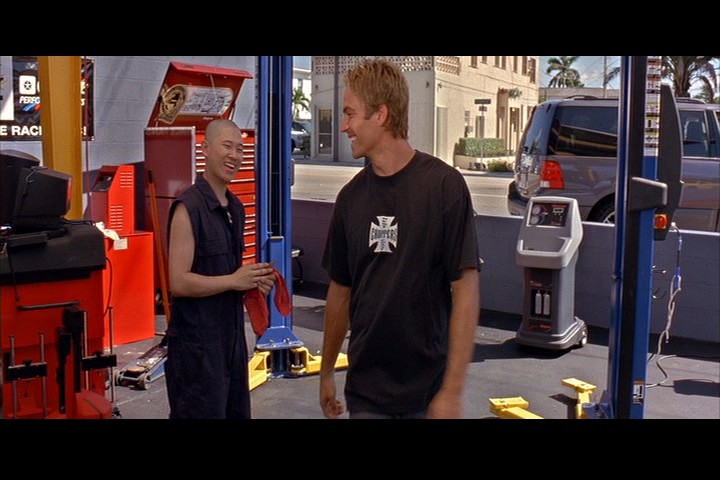

Supplement: S1 Dataset — (ZIP) [file pone.0264302.s001.zip › 2-fast-2-furious-00058401.jpg]

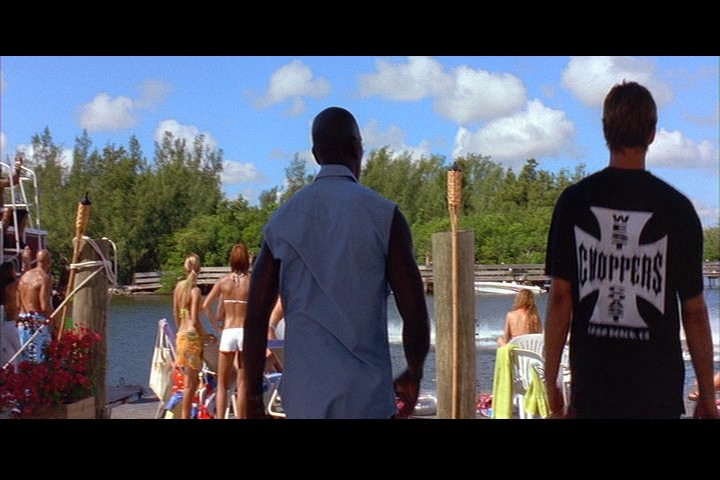

Supplement: S1 Dataset — (ZIP) [file pone.0264302.s001.zip › 2-fast-2-furious-00059001.jpg]

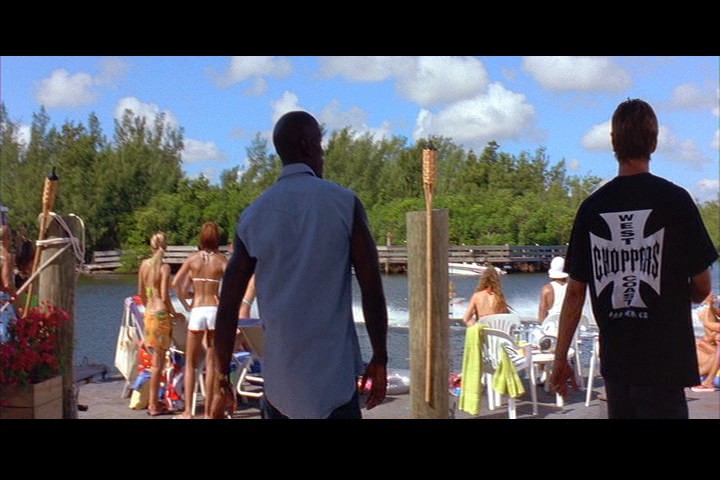

Supplement: S1 Dataset — (ZIP) [file pone.0264302.s001.zip › 2-fast-2-furious-00059011.jpg]

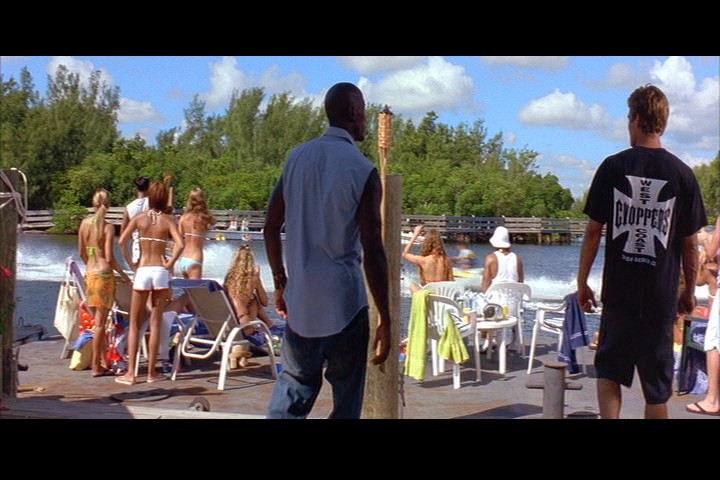

Supplement: S1 Dataset — (ZIP) [file pone.0264302.s001.zip › 2-fast-2-furious-00059031.jpg]

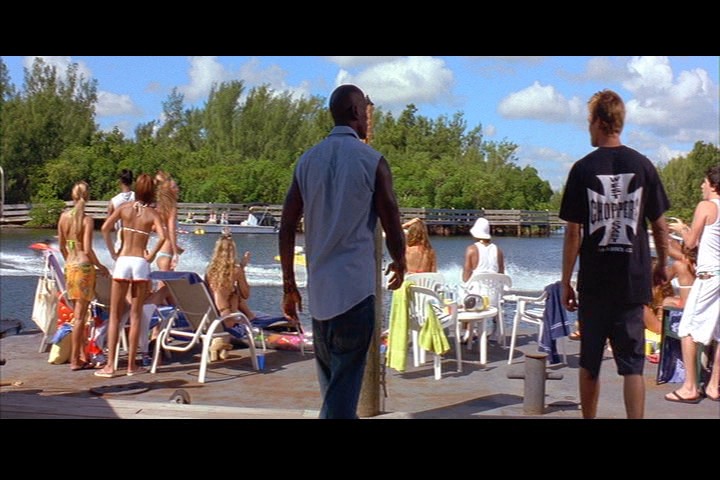

Supplement: S1 Dataset — (ZIP) [file pone.0264302.s001.zip › 2-fast-2-furious-00059041.jpg]

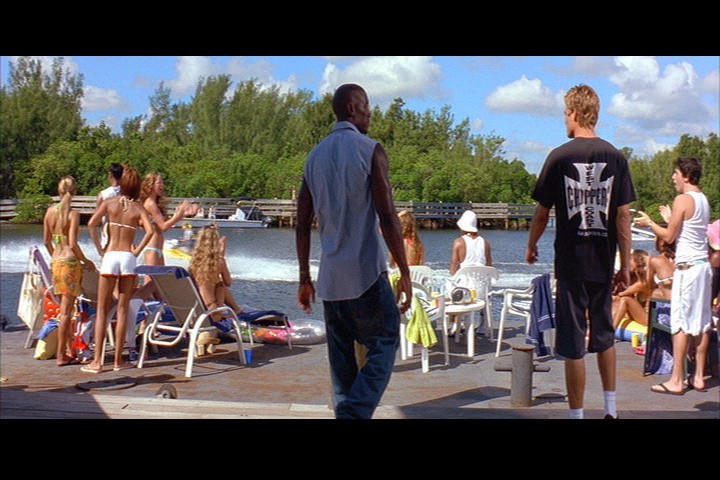

Supplement: S1 Dataset — (ZIP) [file pone.0264302.s001.zip › 2-fast-2-furious-00059051.jpg]

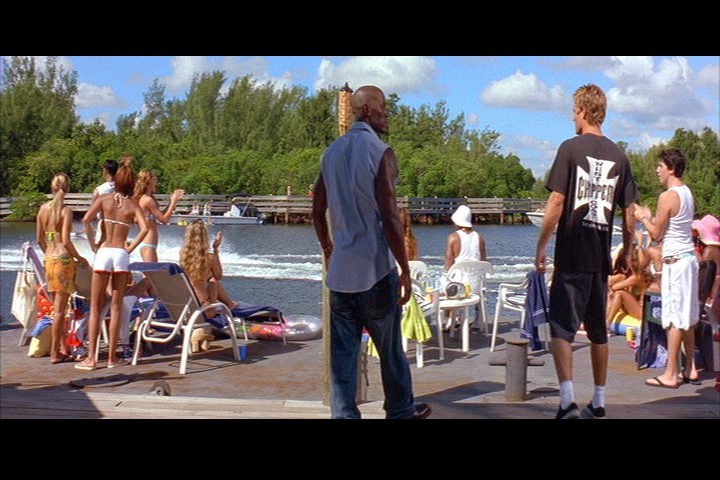

Supplement: S1 Dataset — (ZIP) [file pone.0264302.s001.zip › 2-fast-2-furious-00059061.jpg]

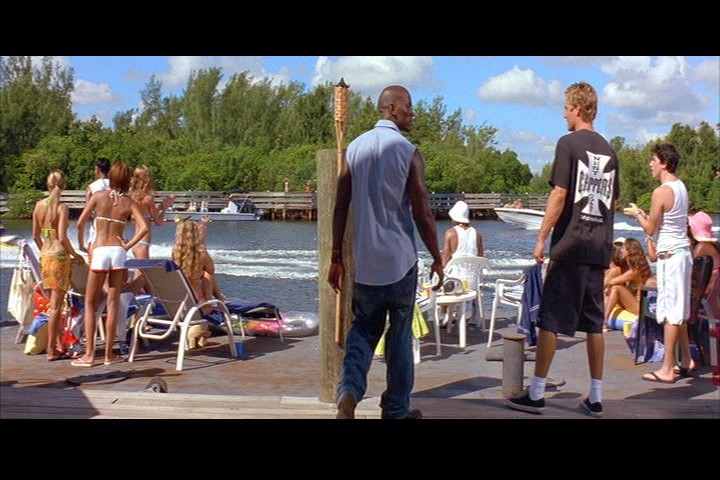

Supplement: S1 Dataset — (ZIP) [file pone.0264302.s001.zip › 2-fast-2-furious-00059071.jpg]

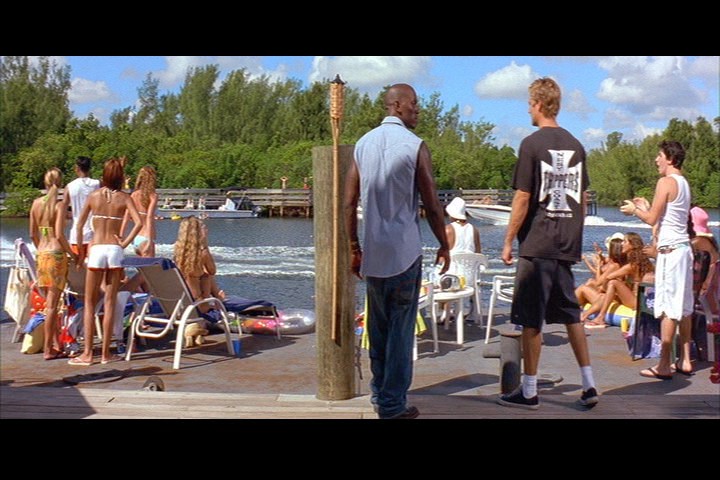

Supplement: S1 Dataset — (ZIP) [file pone.0264302.s001.zip › 2-fast-2-furious-00059081.jpg]

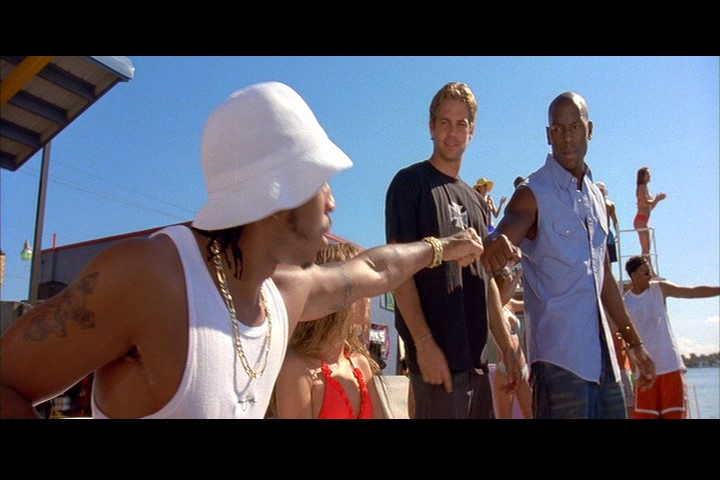

Supplement: S1 Dataset — (ZIP) [file pone.0264302.s001.zip › 2-fast-2-furious-00059611.jpg]

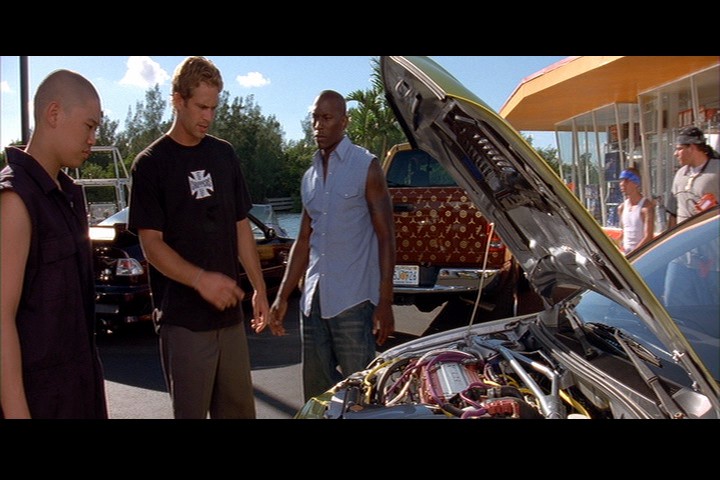

Supplement: S1 Dataset — (ZIP) [file pone.0264302.s001.zip › 2-fast-2-furious-00061751.jpg]

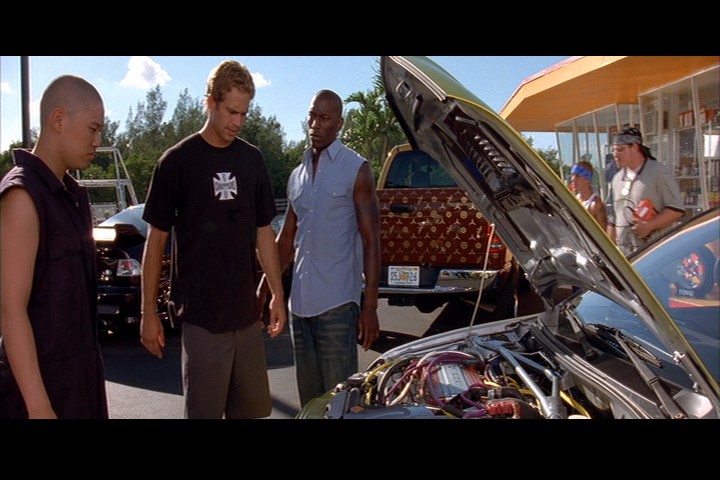

Supplement: S1 Dataset — (ZIP) [file pone.0264302.s001.zip › 2-fast-2-furious-00061761.jpg]

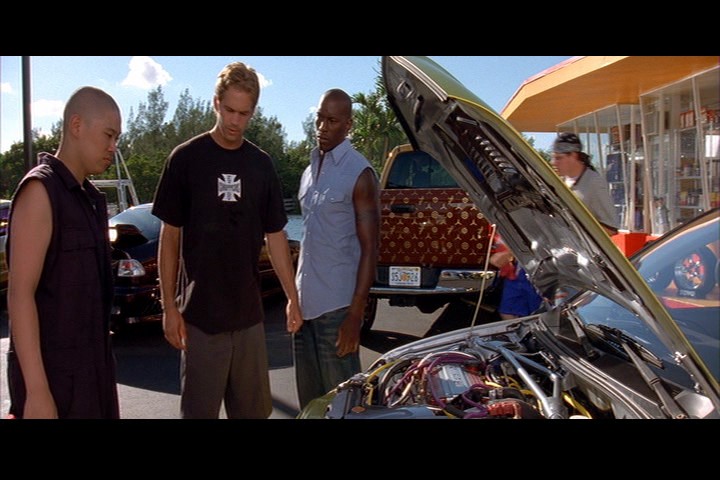

Supplement: S1 Dataset — (ZIP) [file pone.0264302.s001.zip › 2-fast-2-furious-00061771.jpg]

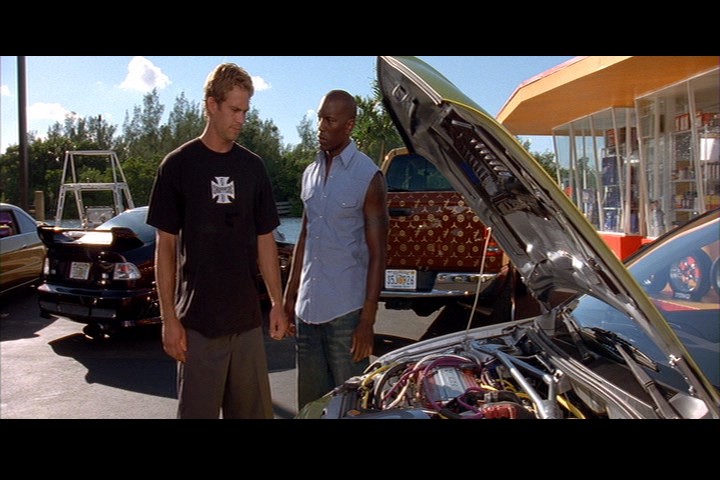

Supplement: S1 Dataset — (ZIP) [file pone.0264302.s001.zip › 2-fast-2-furious-00061891.jpg]

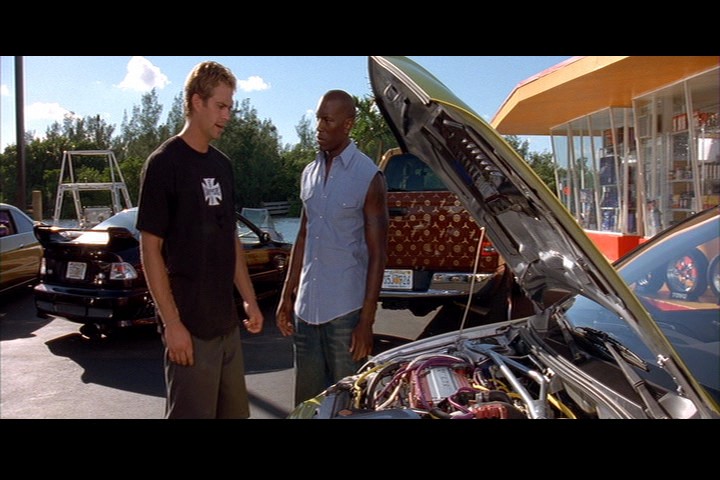

Supplement: S1 Dataset — (ZIP) [file pone.0264302.s001.zip › 2-fast-2-furious-00061911.jpg]

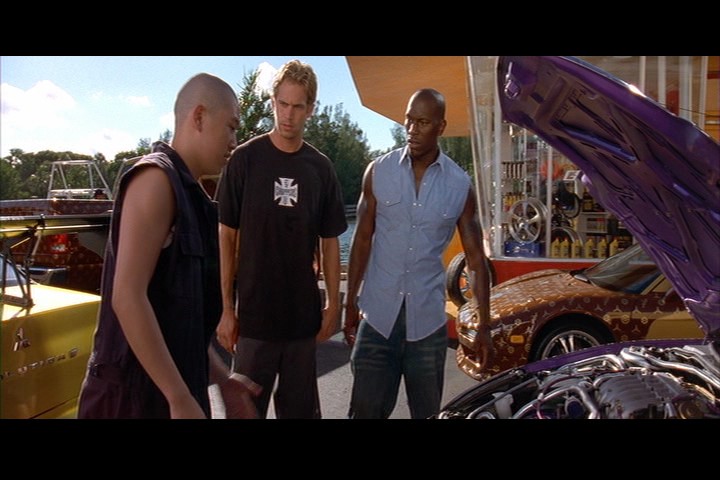

Supplement: S1 Dataset — (ZIP) [file pone.0264302.s001.zip › 2-fast-2-furious-00062121.jpg]

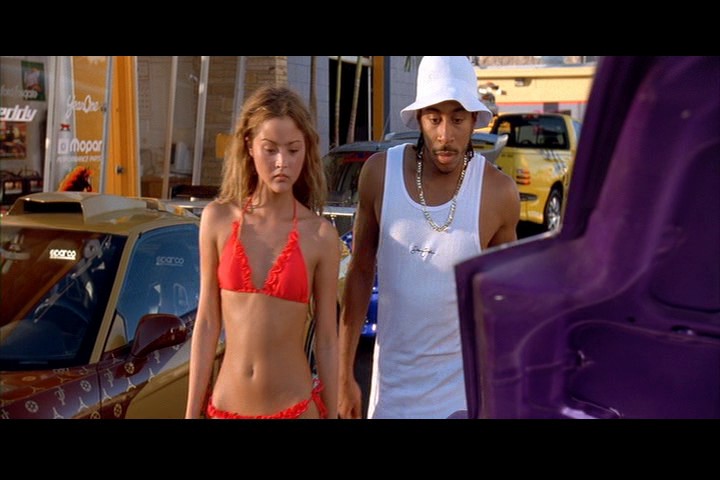

Supplement: S1 Dataset — (ZIP) [file pone.0264302.s001.zip › 2-fast-2-furious-00062211.jpg]

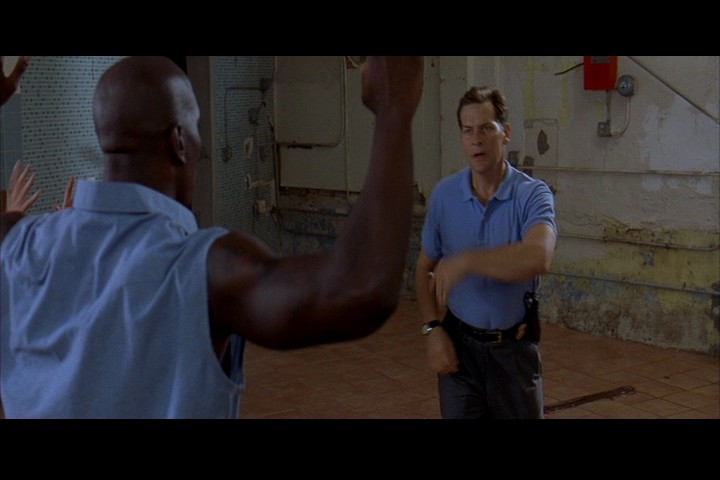

Supplement: S1 Dataset — (ZIP) [file pone.0264302.s001.zip › 2-fast-2-furious-00064481.jpg]
