# Supplementary figures and images for: LHPE-nets: A lightweight 2D and 3D human pose estimation model with well-structural deep networks and multi-view pose sample simplification method (part 4 of 8)
Source: PLoS One. 2022 Feb 23;17(2):e0264302. doi: 10.1371/journal.pone.0264302 (PMC8865690; doi:10.1371/journal.pone.0264302)

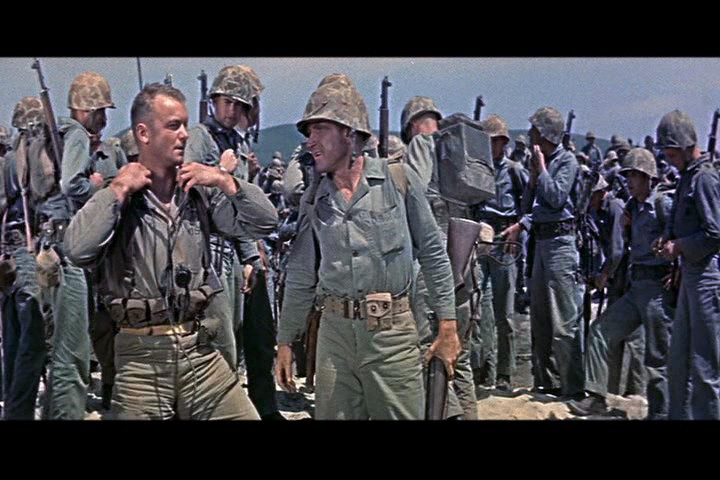

Supplement: S2 Dataset — (ZIP) [file pone.0264302.s002.zip › battle-cry-00157711.jpg]

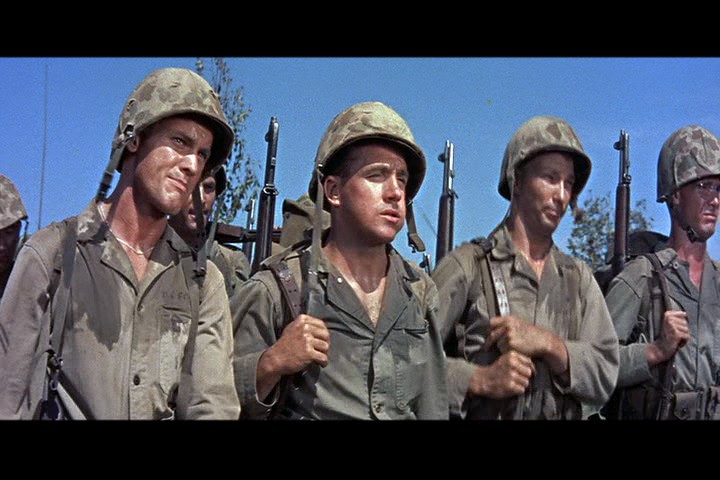

Supplement: S2 Dataset — (ZIP) [file pone.0264302.s002.zip › battle-cry-00158351.jpg]

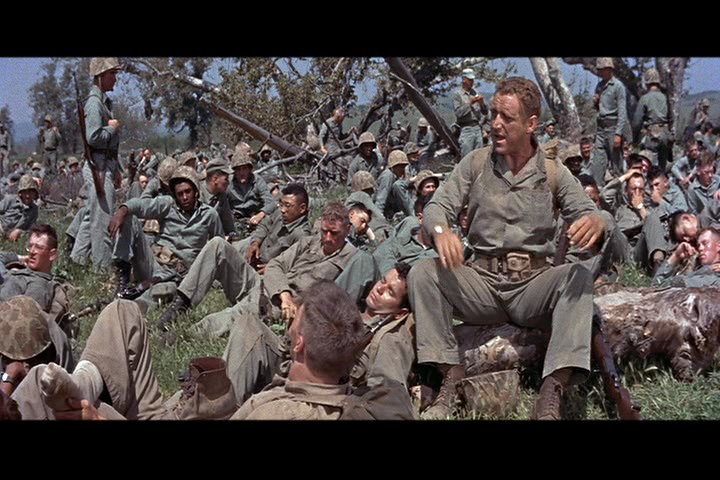

Supplement: S2 Dataset — (ZIP) [file pone.0264302.s002.zip › battle-cry-00159561.jpg]

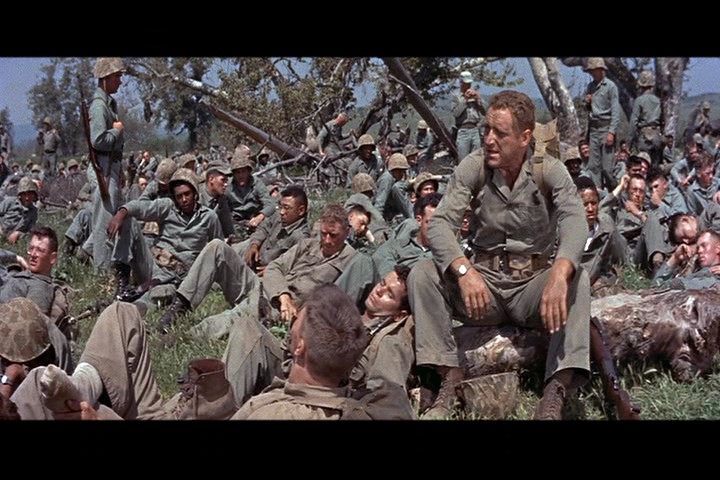

Supplement: S2 Dataset — (ZIP) [file pone.0264302.s002.zip › battle-cry-00159571.jpg]

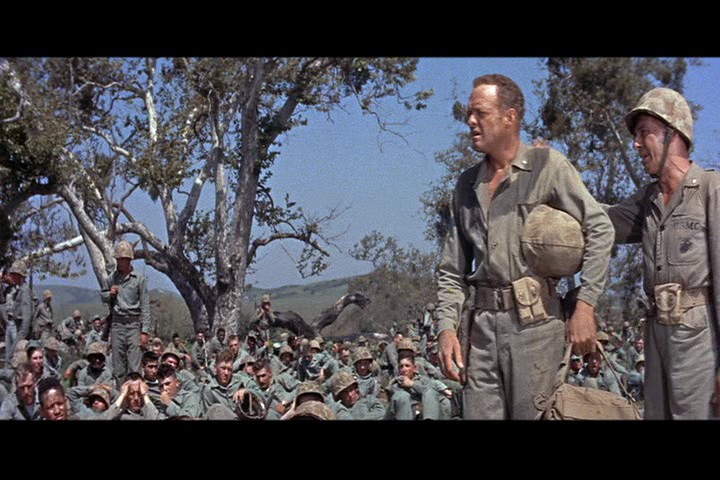

Supplement: S2 Dataset — (ZIP) [file pone.0264302.s002.zip › battle-cry-00159661.jpg]

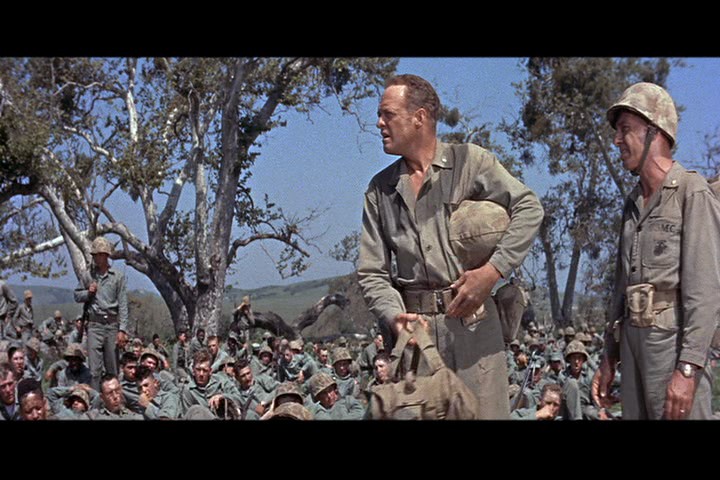

Supplement: S2 Dataset — (ZIP) [file pone.0264302.s002.zip › battle-cry-00159711.jpg]

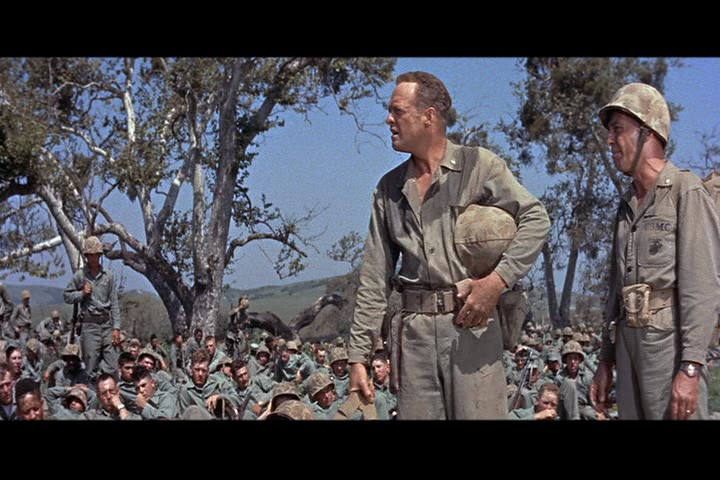

Supplement: S2 Dataset — (ZIP) [file pone.0264302.s002.zip › battle-cry-00159731.jpg]

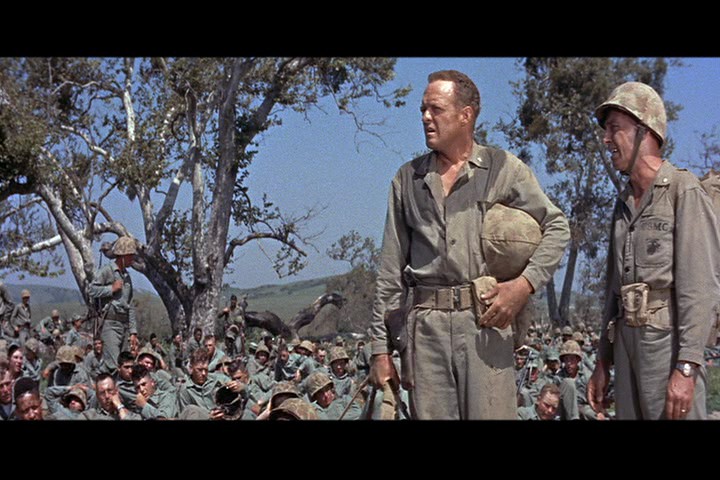

Supplement: S2 Dataset — (ZIP) [file pone.0264302.s002.zip › battle-cry-00159771.jpg]

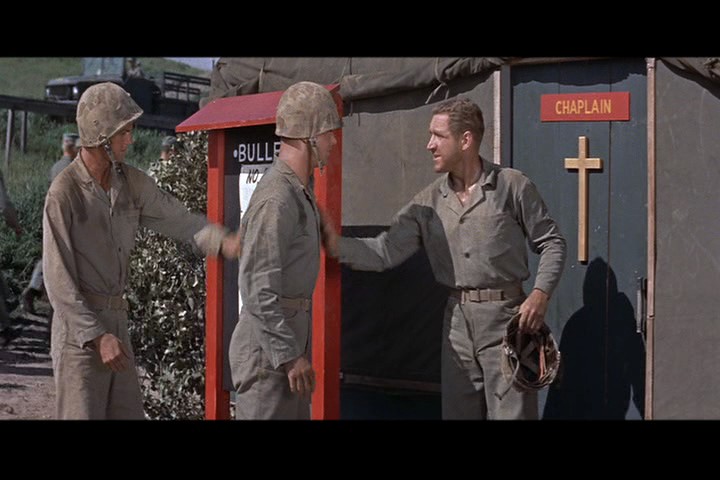

Supplement: S2 Dataset — (ZIP) [file pone.0264302.s002.zip › battle-cry-00162991.jpg]

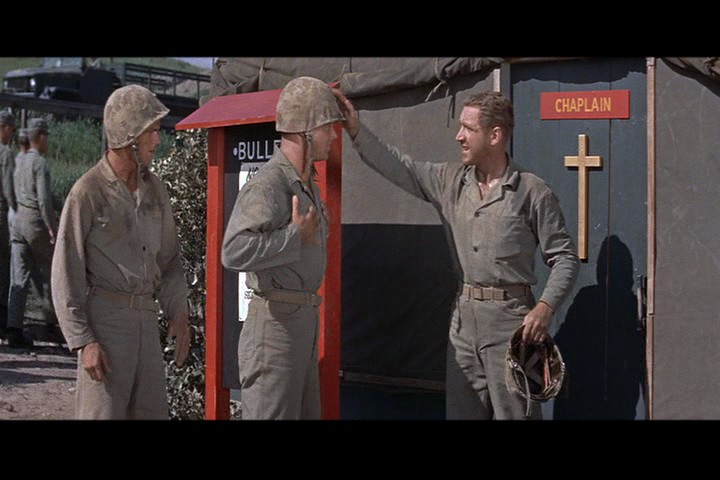

Supplement: S2 Dataset — (ZIP) [file pone.0264302.s002.zip › battle-cry-00163001.jpg]

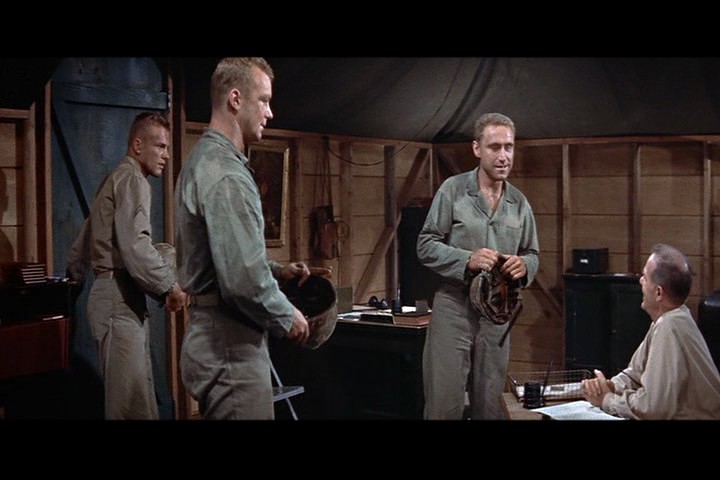

Supplement: S2 Dataset — (ZIP) [file pone.0264302.s002.zip › battle-cry-00163201.jpg]

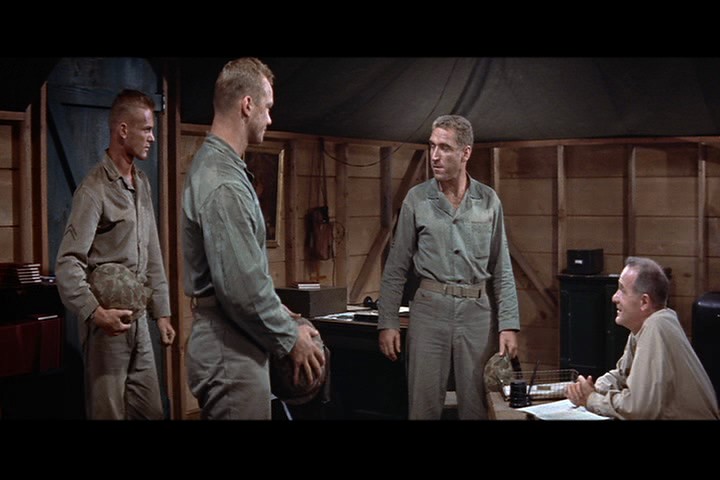

Supplement: S2 Dataset — (ZIP) [file pone.0264302.s002.zip › battle-cry-00163301.jpg]

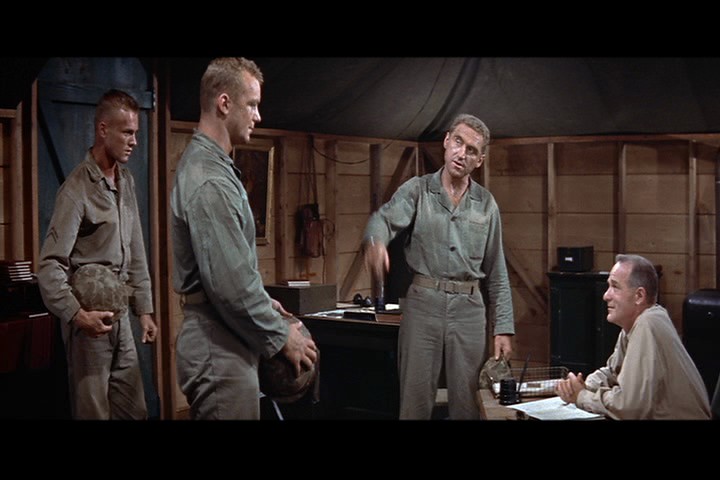

Supplement: S2 Dataset — (ZIP) [file pone.0264302.s002.zip › battle-cry-00163361.jpg]

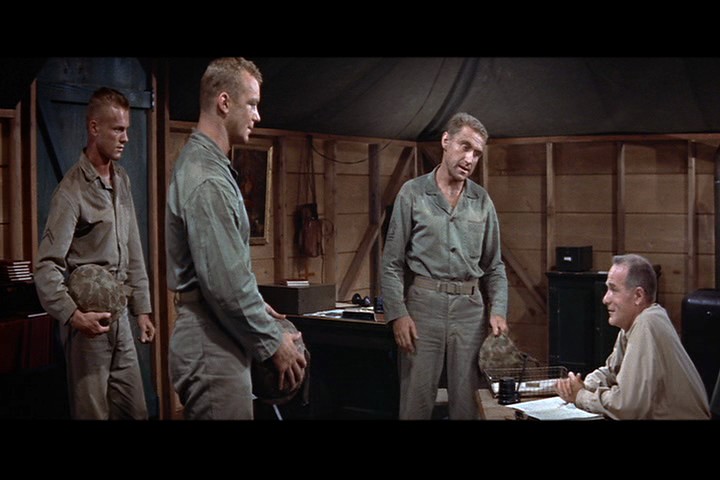

Supplement: S2 Dataset — (ZIP) [file pone.0264302.s002.zip › battle-cry-00163401.jpg]

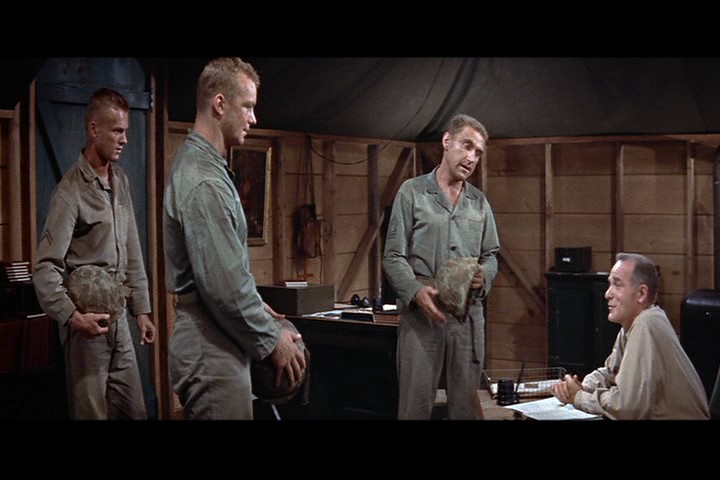

Supplement: S2 Dataset — (ZIP) [file pone.0264302.s002.zip › battle-cry-00163411.jpg]

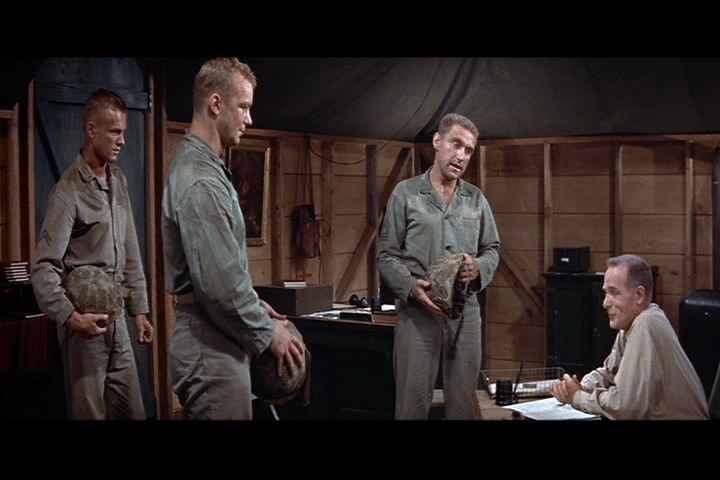

Supplement: S2 Dataset — (ZIP) [file pone.0264302.s002.zip › battle-cry-00163421.jpg]

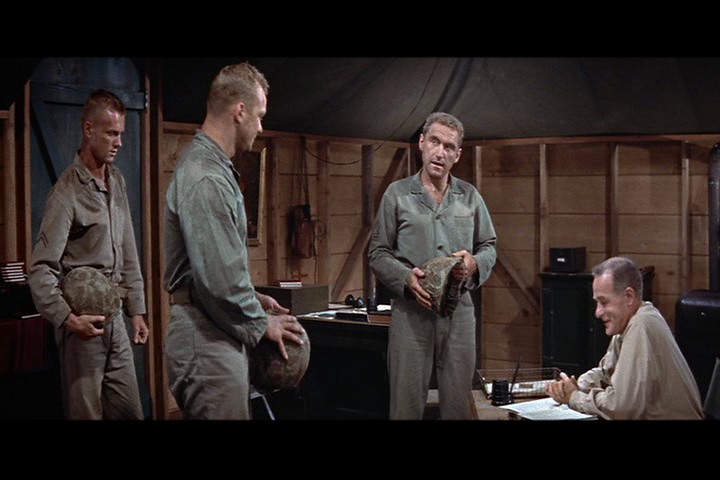

Supplement: S2 Dataset — (ZIP) [file pone.0264302.s002.zip › battle-cry-00163441.jpg]

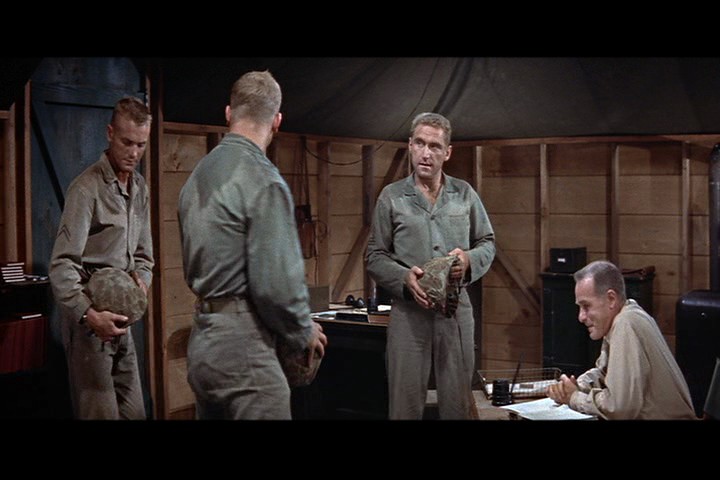

Supplement: S2 Dataset — (ZIP) [file pone.0264302.s002.zip › battle-cry-00163451.jpg]

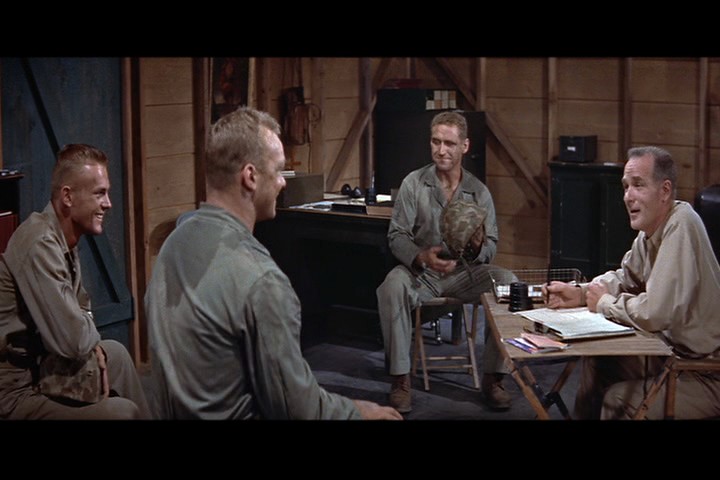

Supplement: S2 Dataset — (ZIP) [file pone.0264302.s002.zip › battle-cry-00164211.jpg]

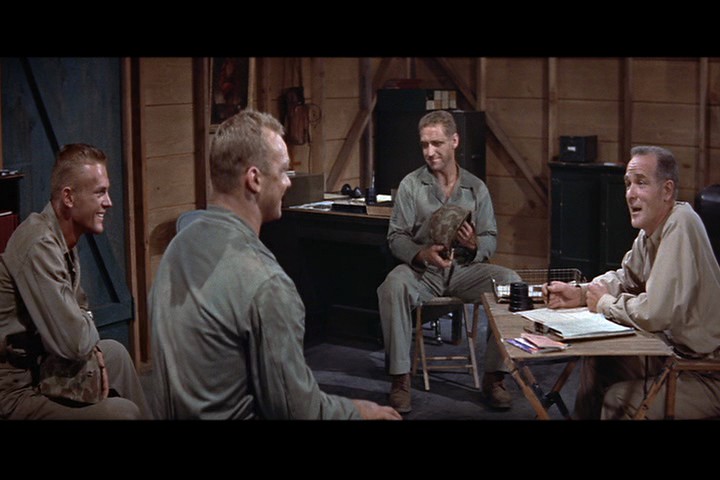

Supplement: S2 Dataset — (ZIP) [file pone.0264302.s002.zip › battle-cry-00164221.jpg]

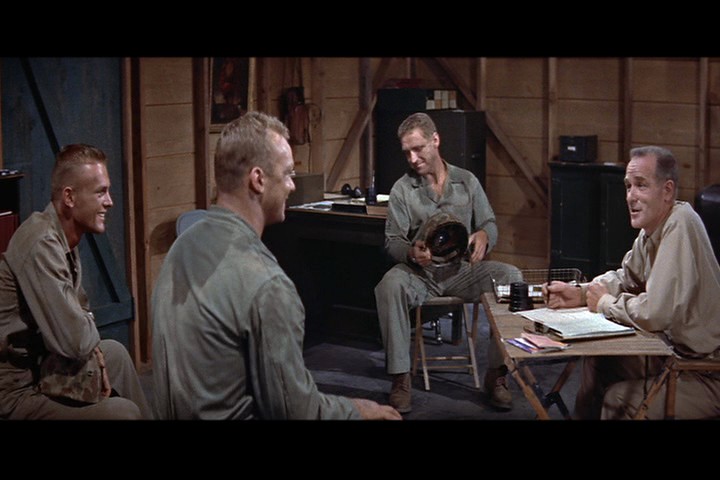

Supplement: S2 Dataset — (ZIP) [file pone.0264302.s002.zip › battle-cry-00164231.jpg]

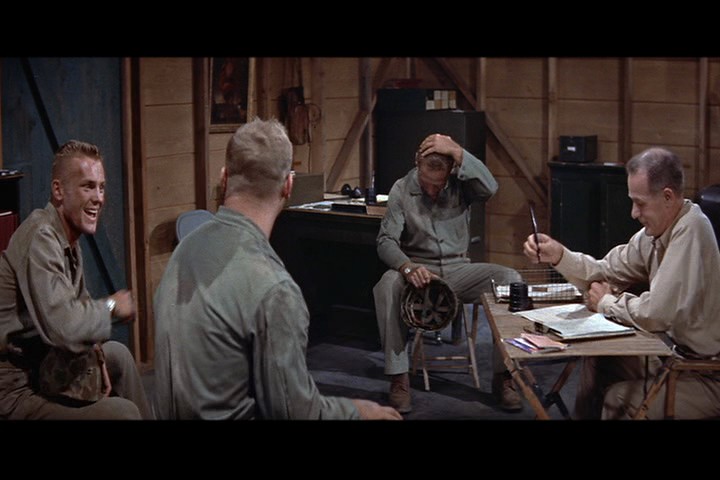

Supplement: S2 Dataset — (ZIP) [file pone.0264302.s002.zip › battle-cry-00164251.jpg]

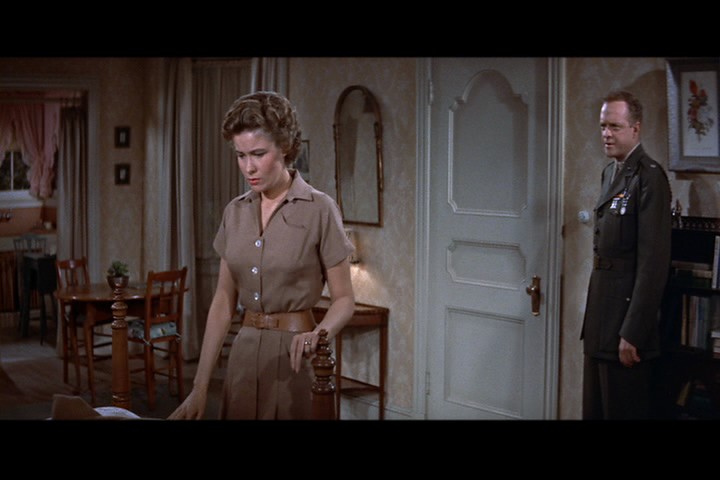

Supplement: S2 Dataset — (ZIP) [file pone.0264302.s002.zip › battle-cry-00167261.jpg]

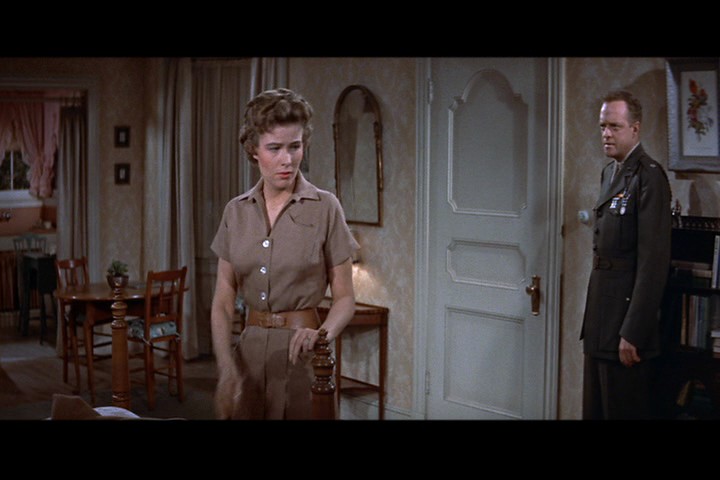

Supplement: S2 Dataset — (ZIP) [file pone.0264302.s002.zip › battle-cry-00167281.jpg]

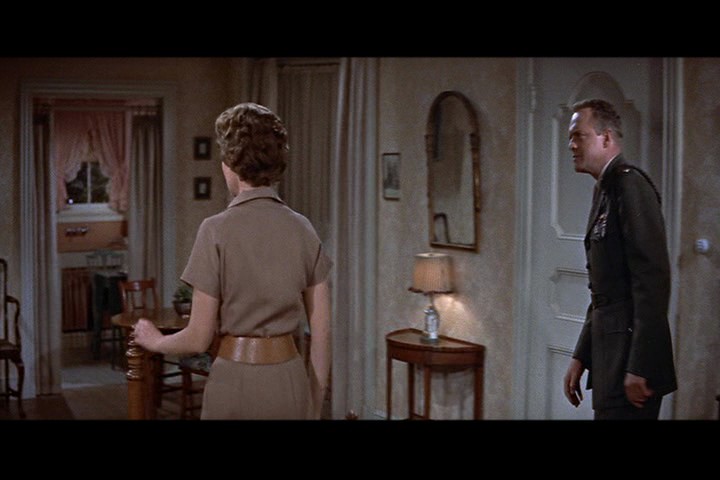

Supplement: S2 Dataset — (ZIP) [file pone.0264302.s002.zip › battle-cry-00167881.jpg]

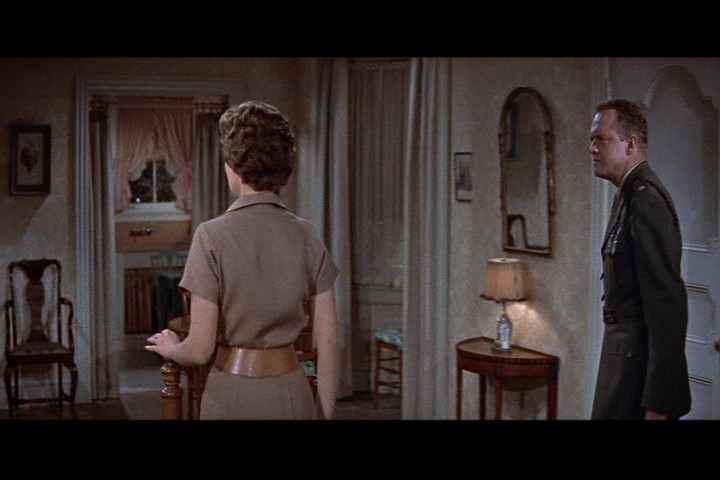

Supplement: S2 Dataset — (ZIP) [file pone.0264302.s002.zip › battle-cry-00167891.jpg]

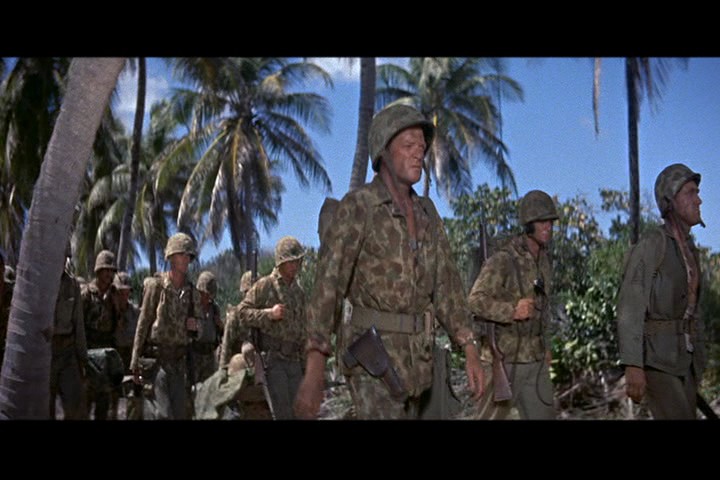

Supplement: S2 Dataset — (ZIP) [file pone.0264302.s002.zip › battle-cry-00175631.jpg]

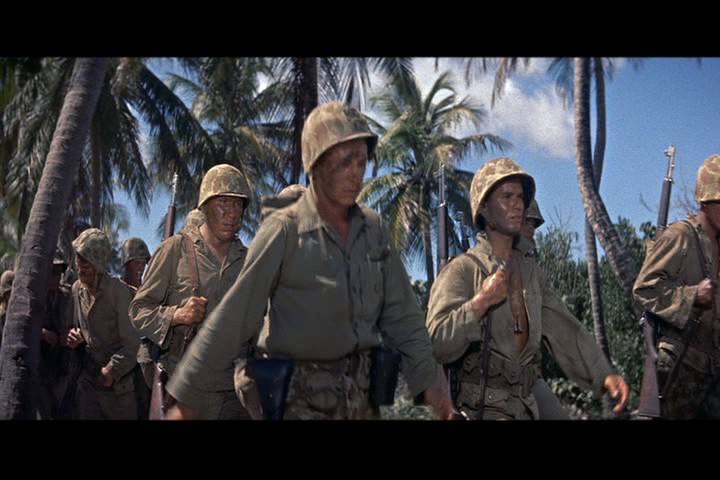

Supplement: S2 Dataset — (ZIP) [file pone.0264302.s002.zip › battle-cry-00175921.jpg]

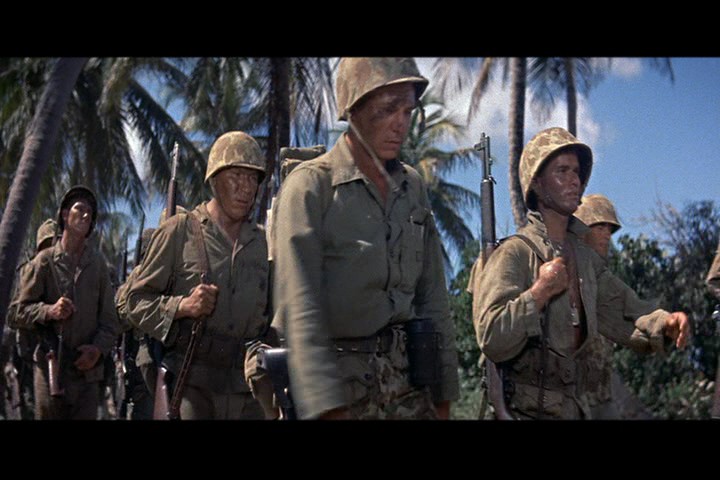

Supplement: S2 Dataset — (ZIP) [file pone.0264302.s002.zip › battle-cry-00175951.jpg]

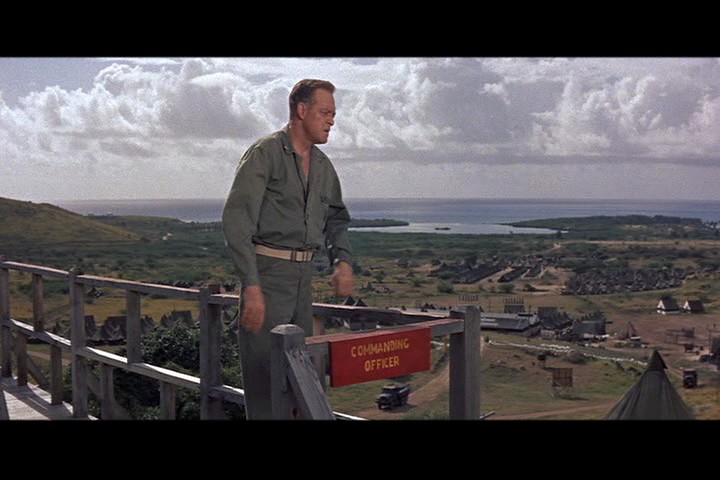

Supplement: S2 Dataset — (ZIP) [file pone.0264302.s002.zip › battle-cry-00176981.jpg]

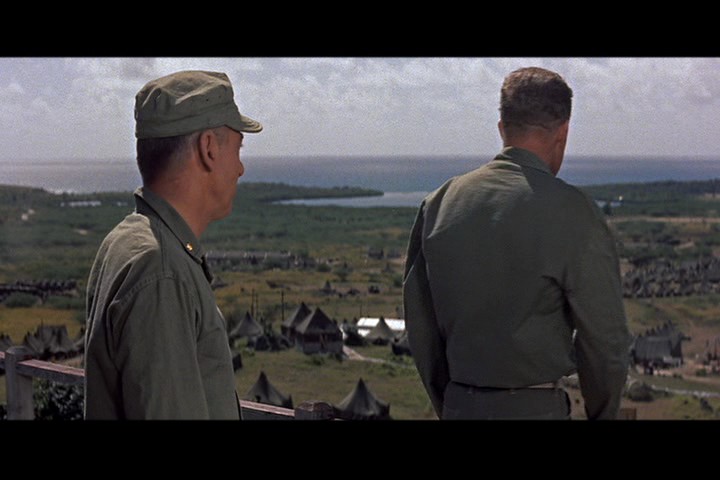

Supplement: S2 Dataset — (ZIP) [file pone.0264302.s002.zip › battle-cry-00177441.jpg]

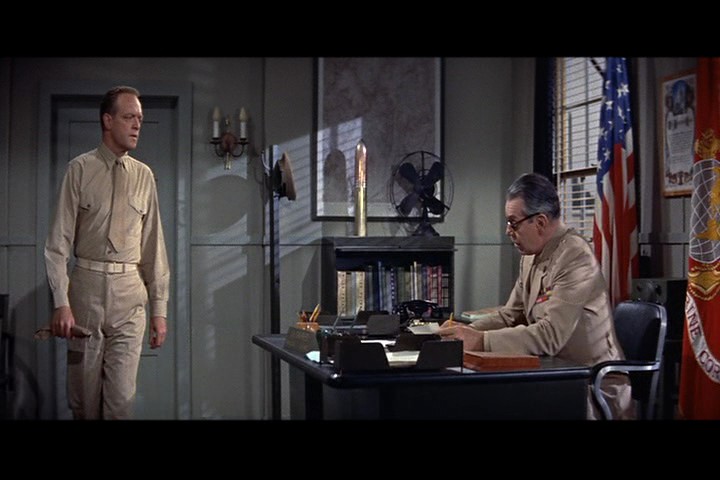

Supplement: S2 Dataset — (ZIP) [file pone.0264302.s002.zip › battle-cry-00177701.jpg]

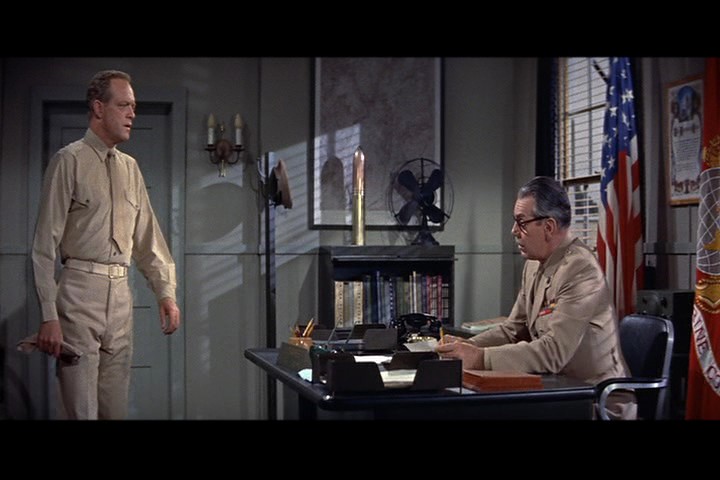

Supplement: S2 Dataset — (ZIP) [file pone.0264302.s002.zip › battle-cry-00177711.jpg]

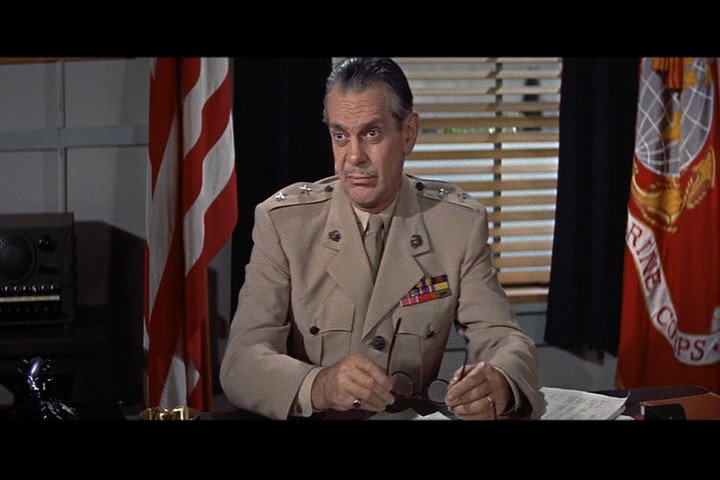

Supplement: S2 Dataset — (ZIP) [file pone.0264302.s002.zip › battle-cry-00177831.jpg]

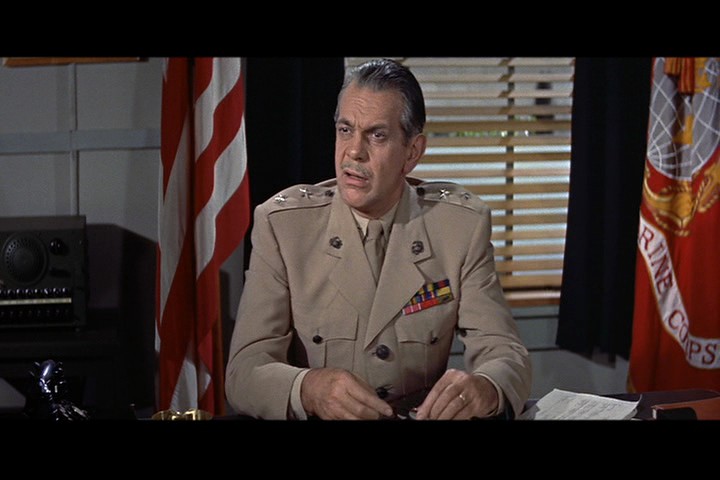

Supplement: S2 Dataset — (ZIP) [file pone.0264302.s002.zip › battle-cry-00178481.jpg]

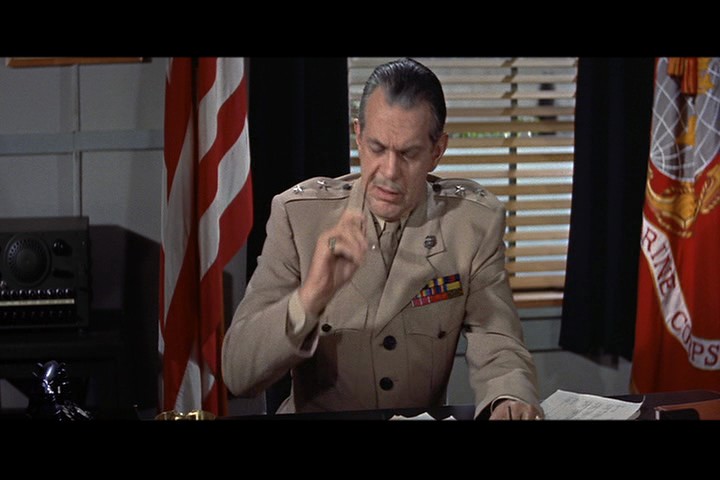

Supplement: S2 Dataset — (ZIP) [file pone.0264302.s002.zip › battle-cry-00178511.jpg]

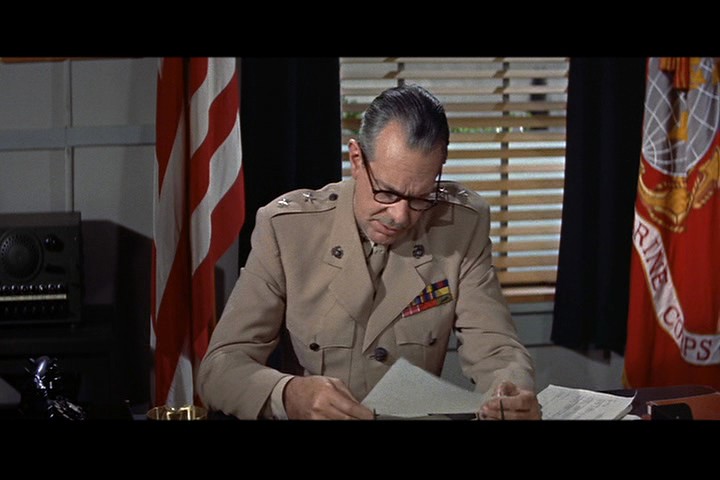

Supplement: S2 Dataset — (ZIP) [file pone.0264302.s002.zip › battle-cry-00178551.jpg]

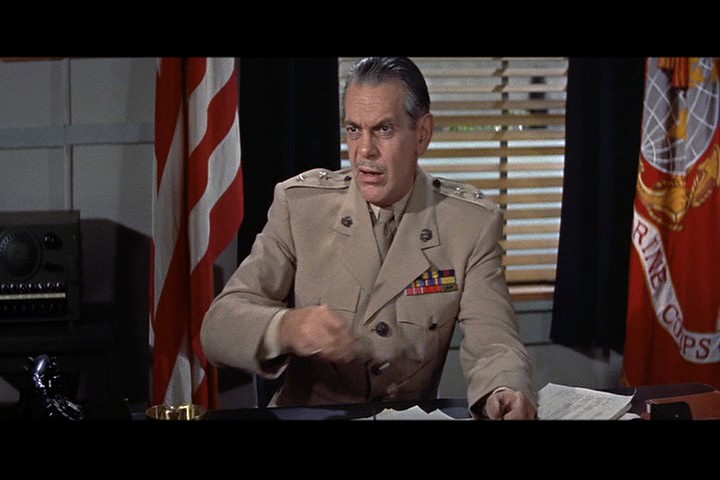

Supplement: S2 Dataset — (ZIP) [file pone.0264302.s002.zip › battle-cry-00178731.jpg]

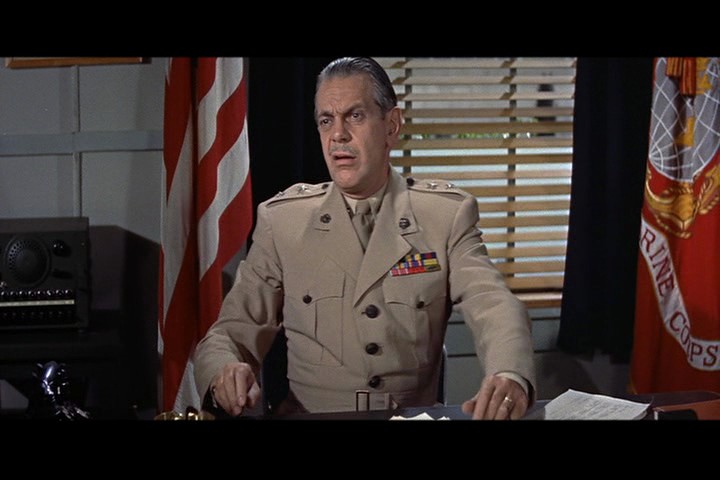

Supplement: S2 Dataset — (ZIP) [file pone.0264302.s002.zip › battle-cry-00178751.jpg]

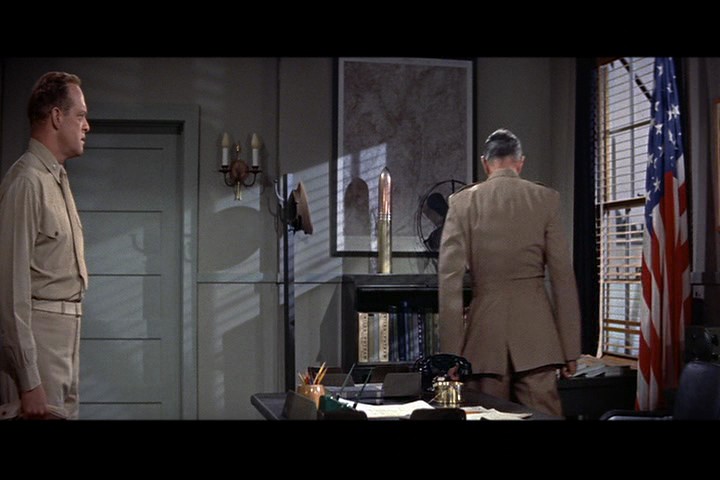

Supplement: S2 Dataset — (ZIP) [file pone.0264302.s002.zip › battle-cry-00179961.jpg]

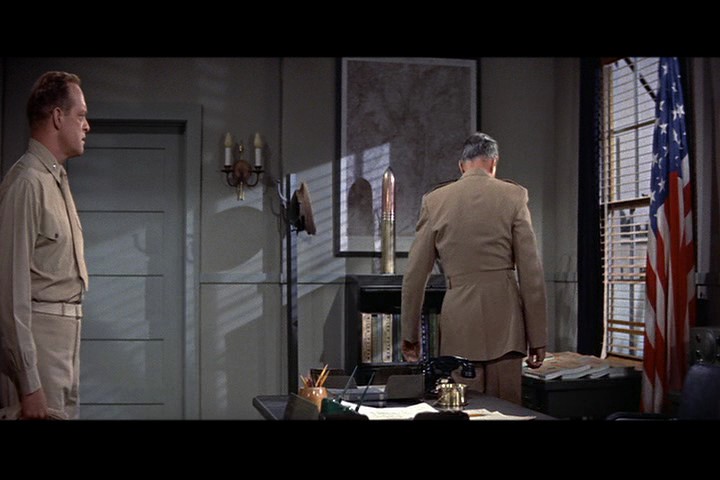

Supplement: S2 Dataset — (ZIP) [file pone.0264302.s002.zip › battle-cry-00179971.jpg]

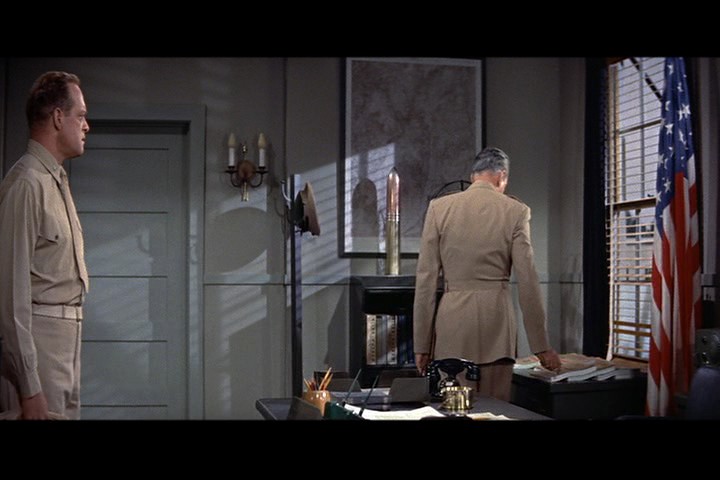

Supplement: S2 Dataset — (ZIP) [file pone.0264302.s002.zip › battle-cry-00179981.jpg]

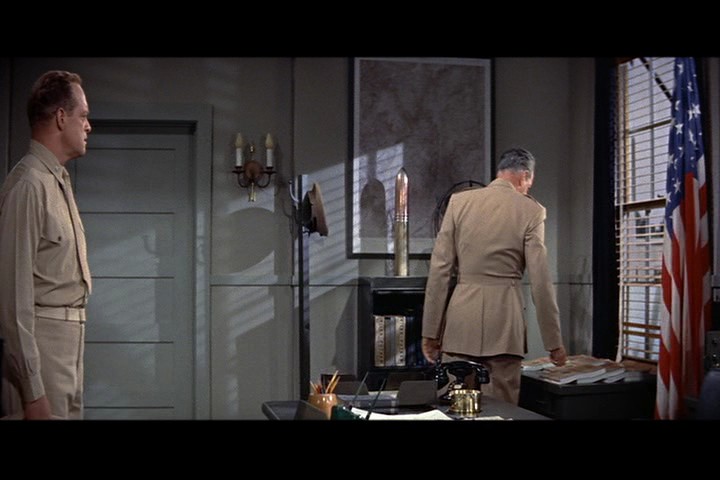

Supplement: S2 Dataset — (ZIP) [file pone.0264302.s002.zip › battle-cry-00179991.jpg]

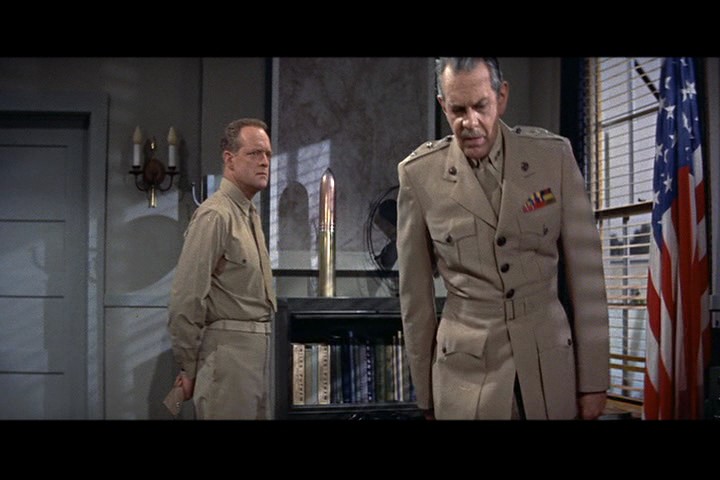

Supplement: S2 Dataset — (ZIP) [file pone.0264302.s002.zip › battle-cry-00180681.jpg]

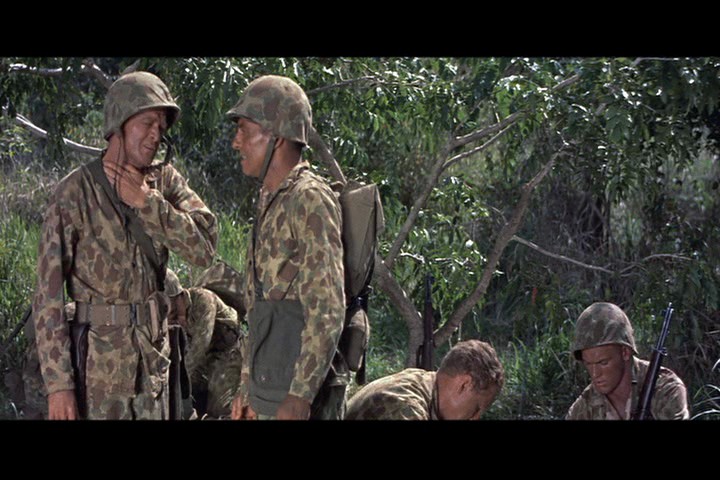

Supplement: S2 Dataset — (ZIP) [file pone.0264302.s002.zip › battle-cry-00191701.jpg]

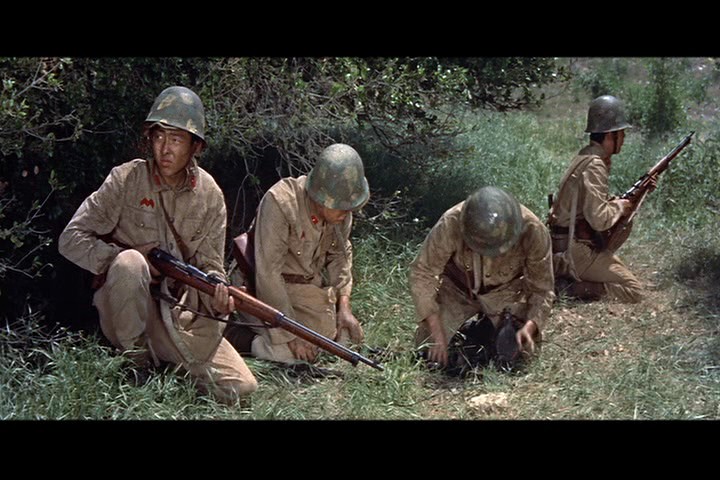

Supplement: S2 Dataset — (ZIP) [file pone.0264302.s002.zip › battle-cry-00193421.jpg]

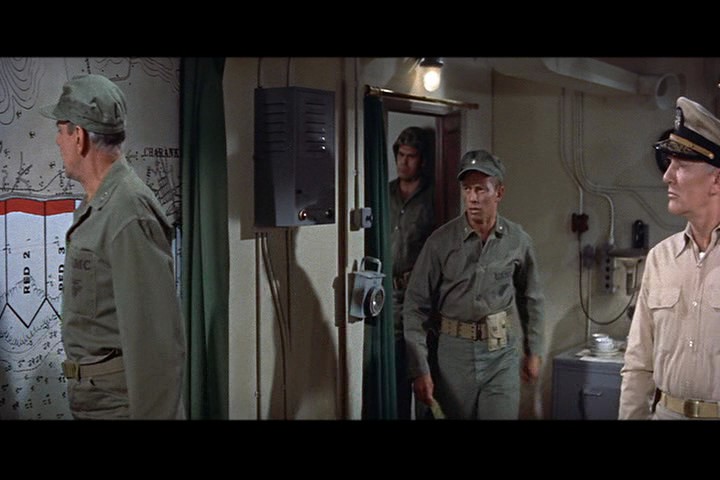

Supplement: S2 Dataset — (ZIP) [file pone.0264302.s002.zip › battle-cry-00194171.jpg]

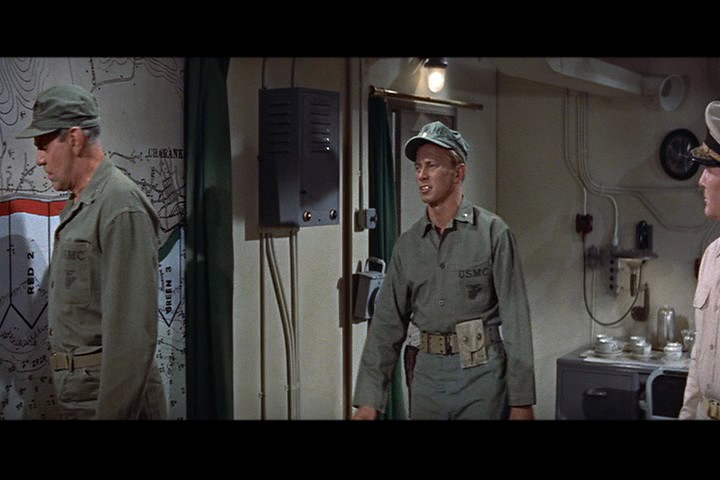

Supplement: S2 Dataset — (ZIP) [file pone.0264302.s002.zip › battle-cry-00194191.jpg]

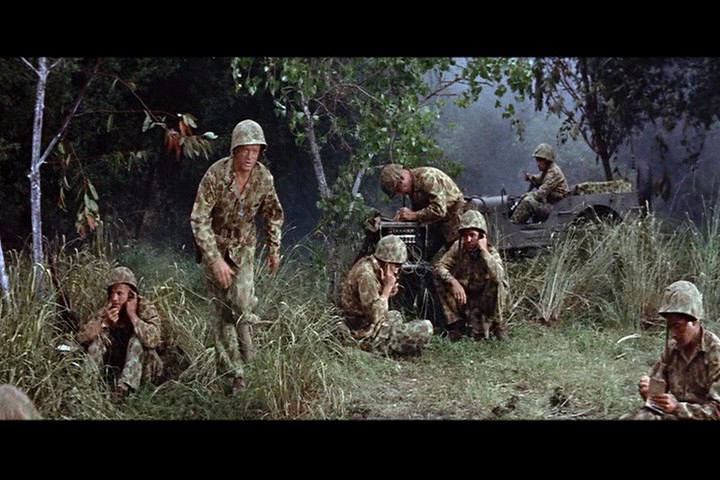

Supplement: S2 Dataset — (ZIP) [file pone.0264302.s002.zip › battle-cry-00194531.jpg]

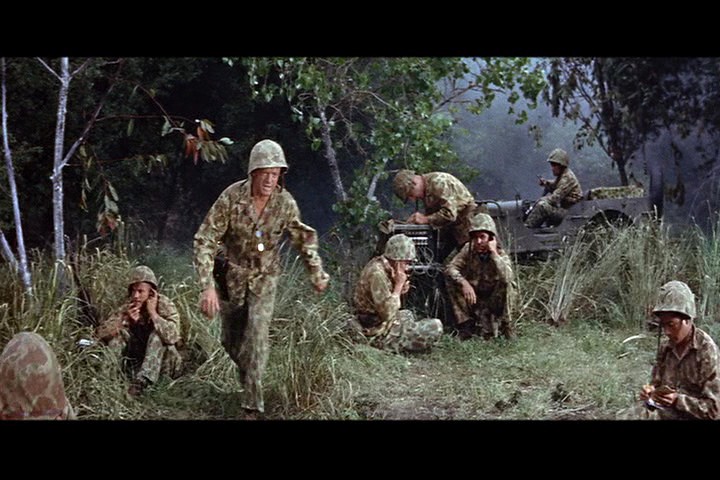

Supplement: S2 Dataset — (ZIP) [file pone.0264302.s002.zip › battle-cry-00194541.jpg]

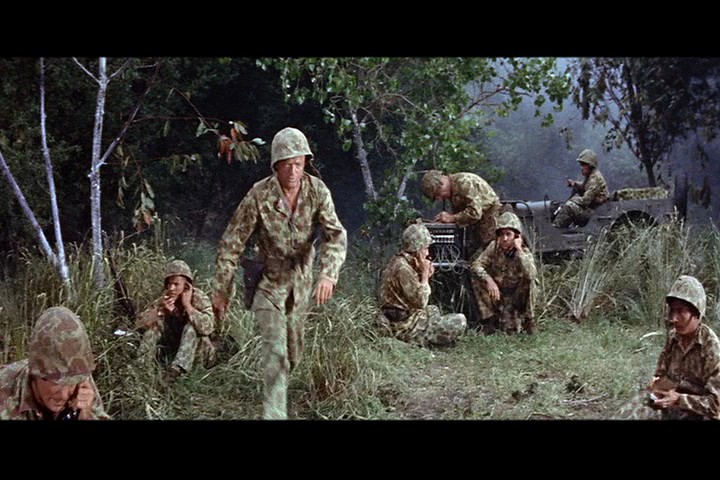

Supplement: S2 Dataset — (ZIP) [file pone.0264302.s002.zip › battle-cry-00194551.jpg]

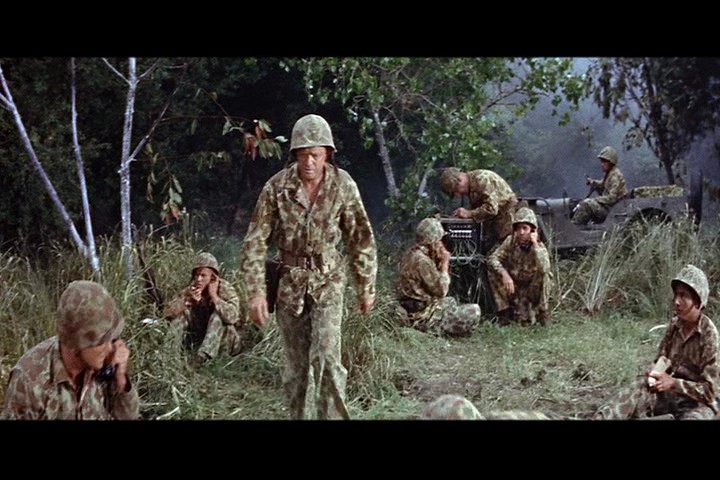

Supplement: S2 Dataset — (ZIP) [file pone.0264302.s002.zip › battle-cry-00194561.jpg]

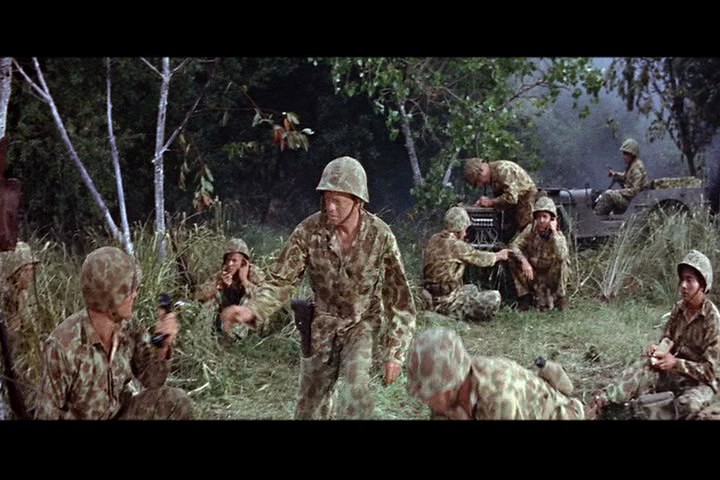

Supplement: S2 Dataset — (ZIP) [file pone.0264302.s002.zip › battle-cry-00194571.jpg]

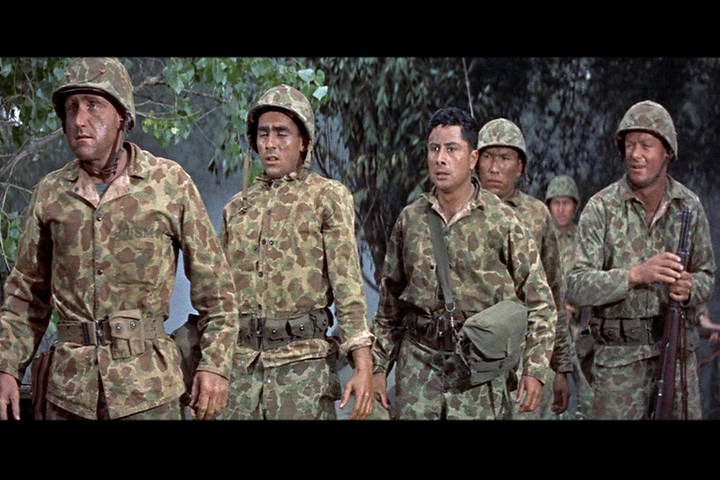

Supplement: S2 Dataset — (ZIP) [file pone.0264302.s002.zip › battle-cry-00197581.jpg]

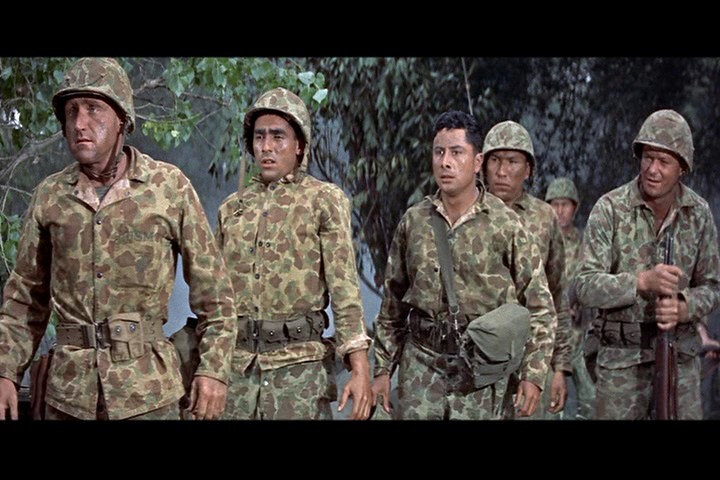

Supplement: S2 Dataset — (ZIP) [file pone.0264302.s002.zip › battle-cry-00197591.jpg]

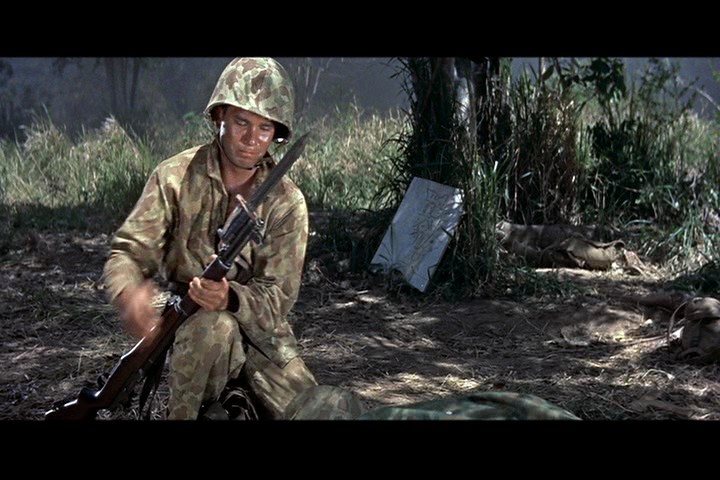

Supplement: S2 Dataset — (ZIP) [file pone.0264302.s002.zip › battle-cry-00198411.jpg]

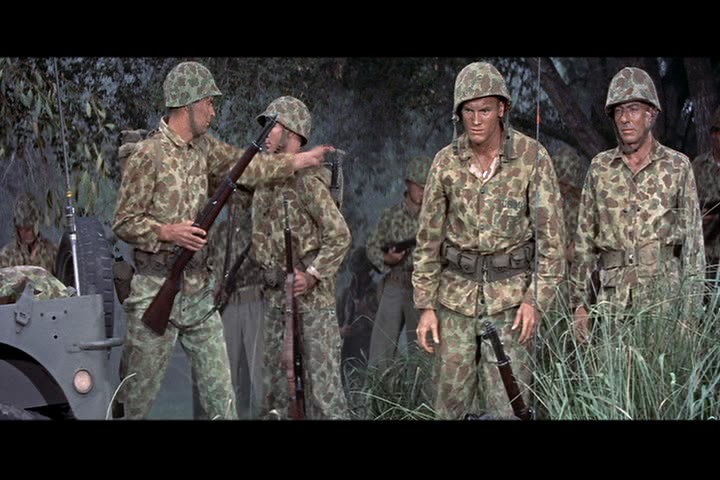

Supplement: S2 Dataset — (ZIP) [file pone.0264302.s002.zip › battle-cry-00198501.jpg]

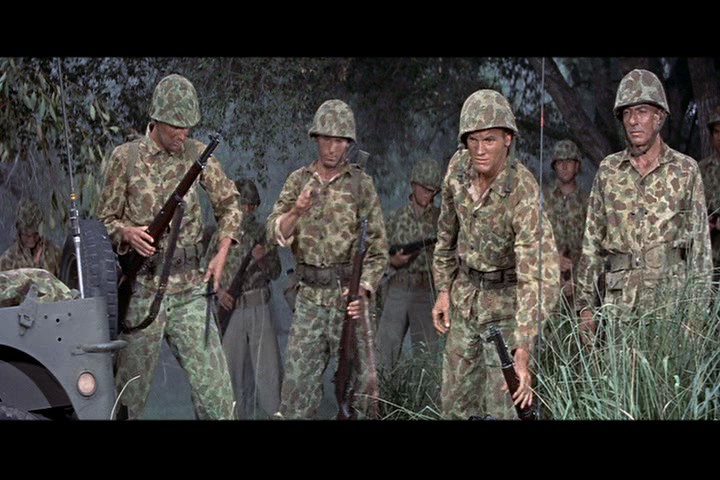

Supplement: S2 Dataset — (ZIP) [file pone.0264302.s002.zip › battle-cry-00198531.jpg]

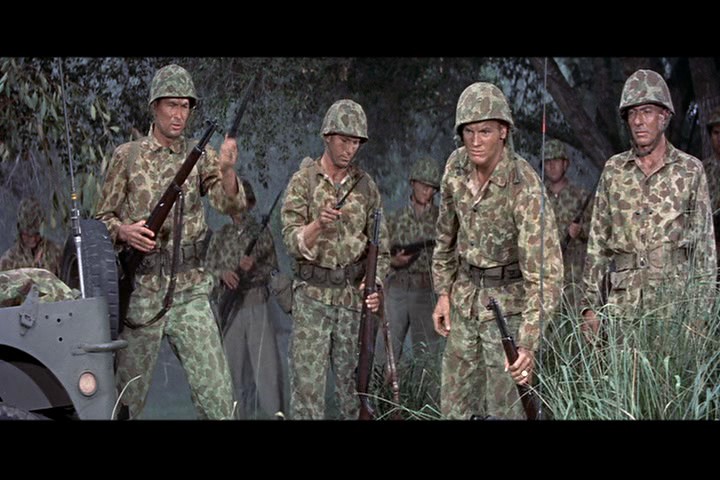

Supplement: S2 Dataset — (ZIP) [file pone.0264302.s002.zip › battle-cry-00198541.jpg]

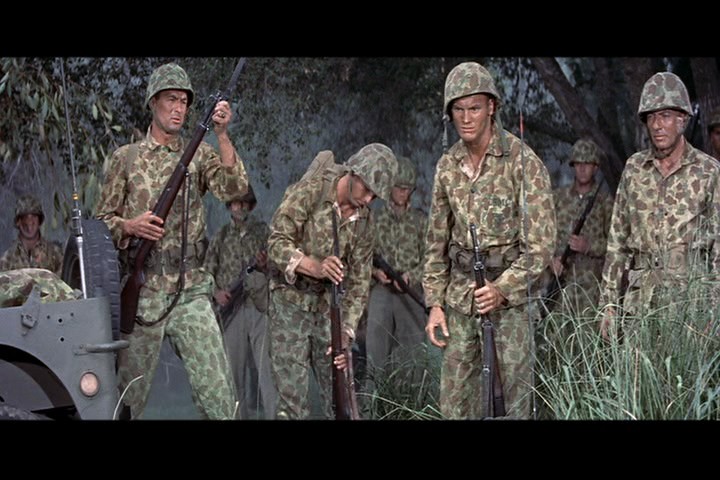

Supplement: S2 Dataset — (ZIP) [file pone.0264302.s002.zip › battle-cry-00198571.jpg]

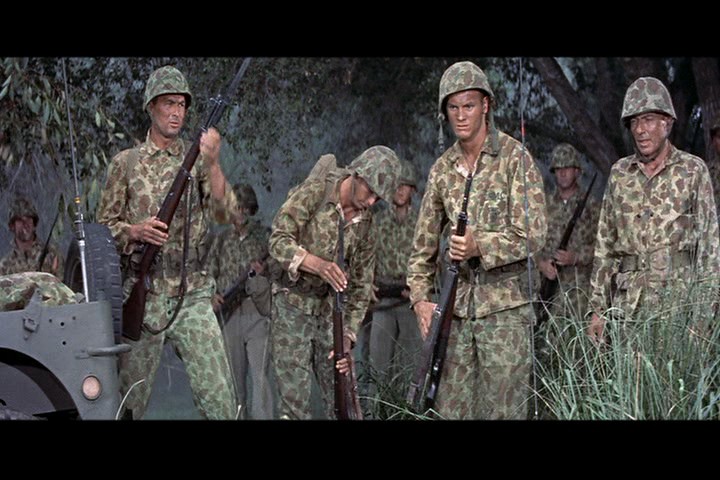

Supplement: S2 Dataset — (ZIP) [file pone.0264302.s002.zip › battle-cry-00198591.jpg]

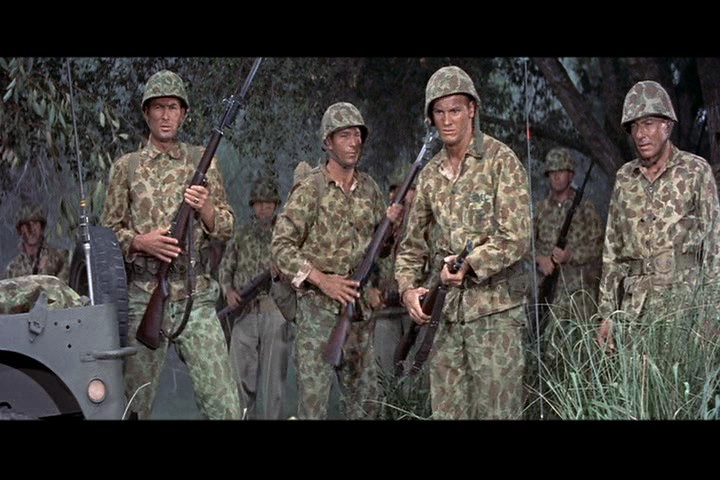

Supplement: S2 Dataset — (ZIP) [file pone.0264302.s002.zip › battle-cry-00198611.jpg]

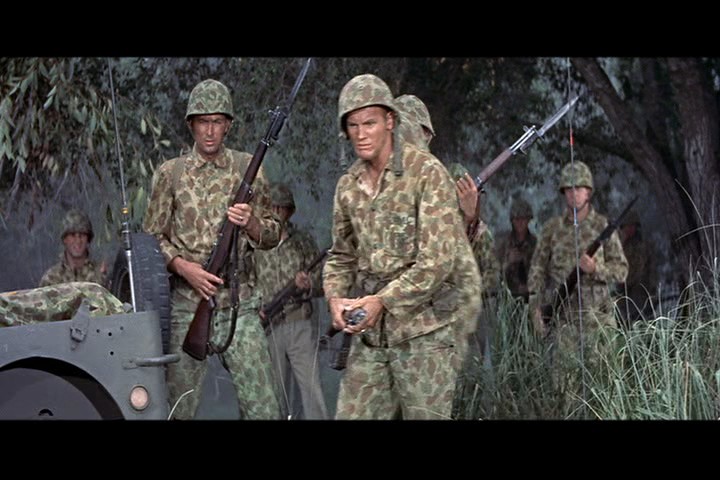

Supplement: S2 Dataset — (ZIP) [file pone.0264302.s002.zip › battle-cry-00198731.jpg]

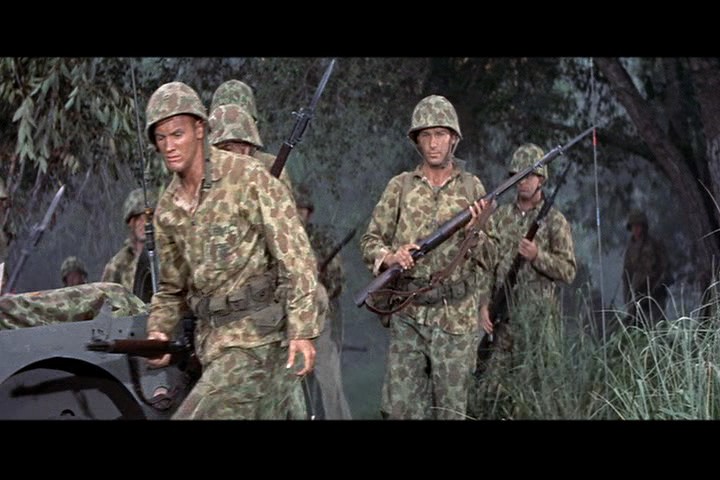

Supplement: S2 Dataset — (ZIP) [file pone.0264302.s002.zip › battle-cry-00198751.jpg]

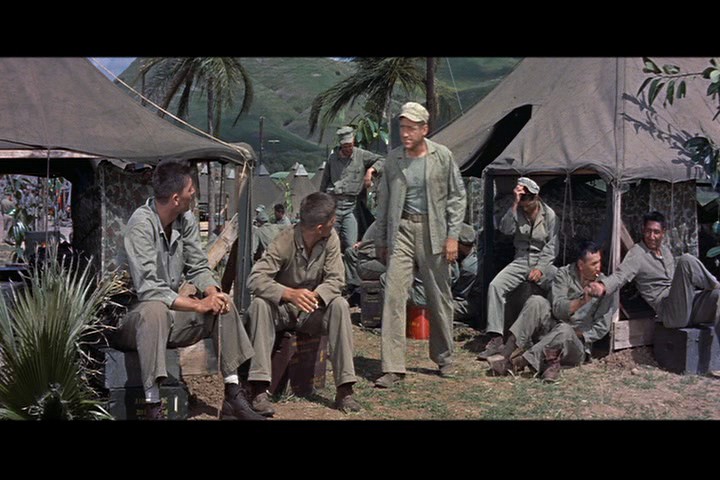

Supplement: S2 Dataset — (ZIP) [file pone.0264302.s002.zip › battle-cry-00202001.jpg]

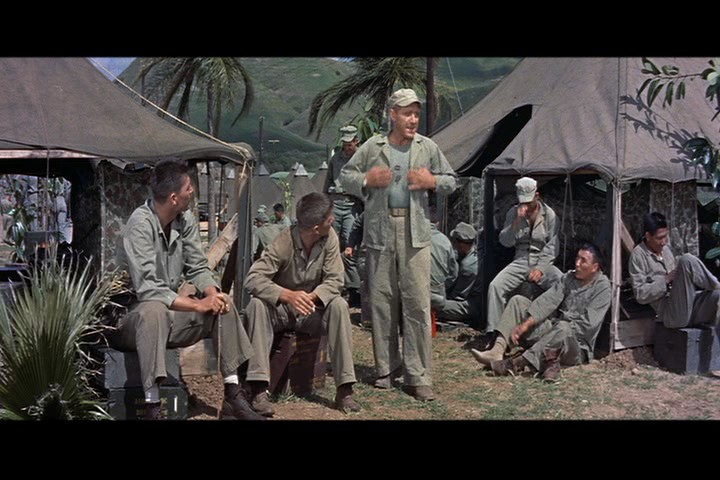

Supplement: S2 Dataset — (ZIP) [file pone.0264302.s002.zip › battle-cry-00202031.jpg]

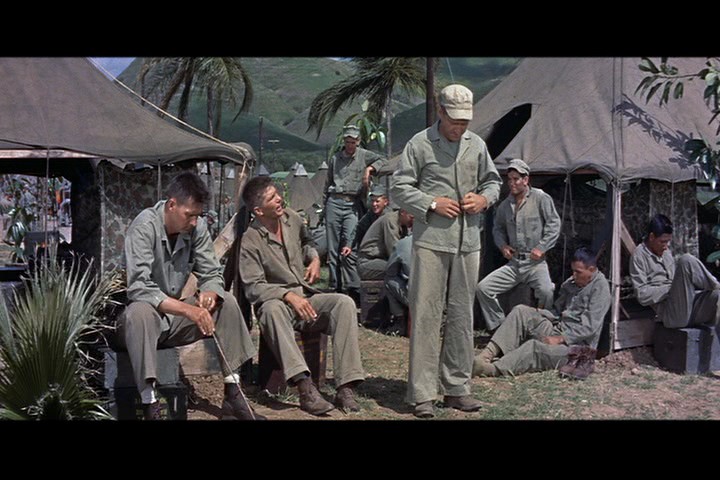

Supplement: S2 Dataset — (ZIP) [file pone.0264302.s002.zip › battle-cry-00202201.jpg]

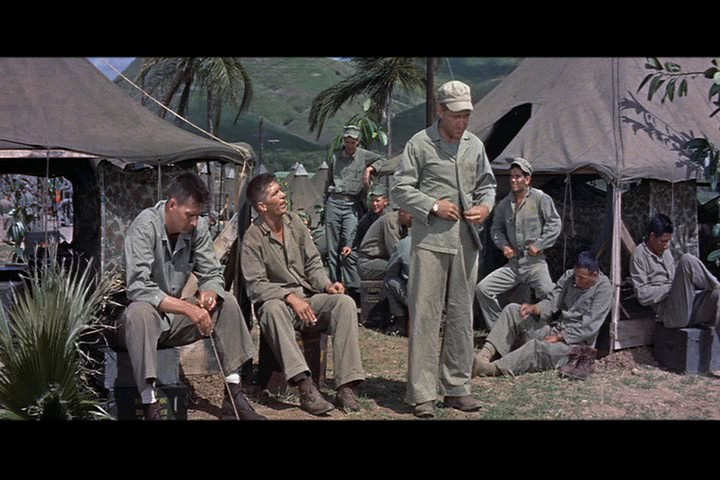

Supplement: S2 Dataset — (ZIP) [file pone.0264302.s002.zip › battle-cry-00202211.jpg]

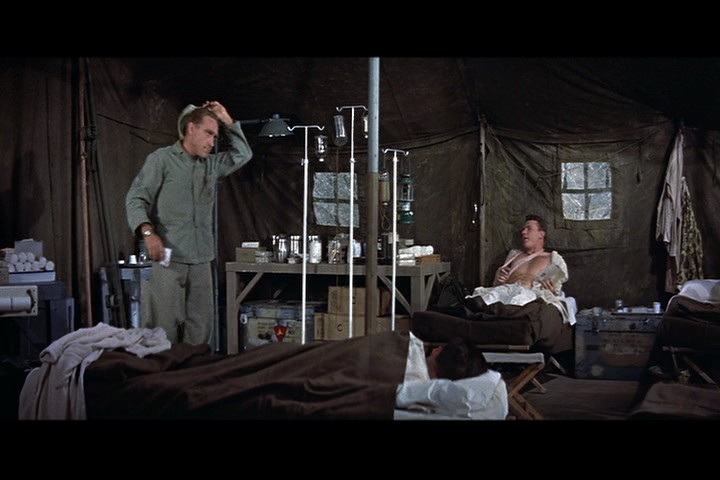

Supplement: S2 Dataset — (ZIP) [file pone.0264302.s002.zip › battle-cry-00202641.jpg]

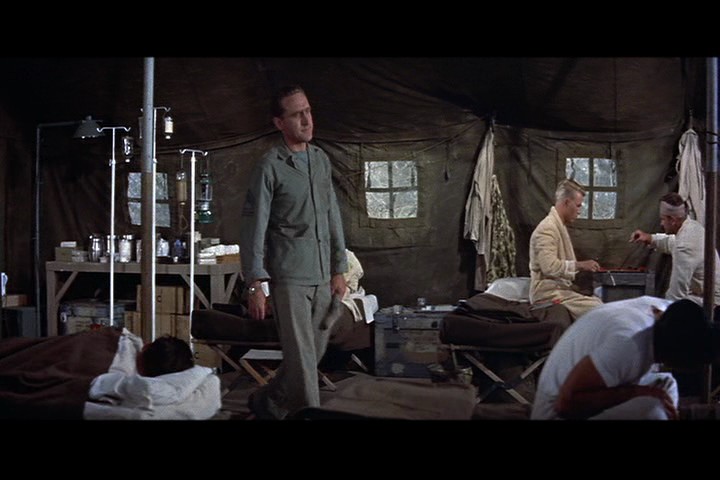

Supplement: S2 Dataset — (ZIP) [file pone.0264302.s002.zip › battle-cry-00202941.jpg]

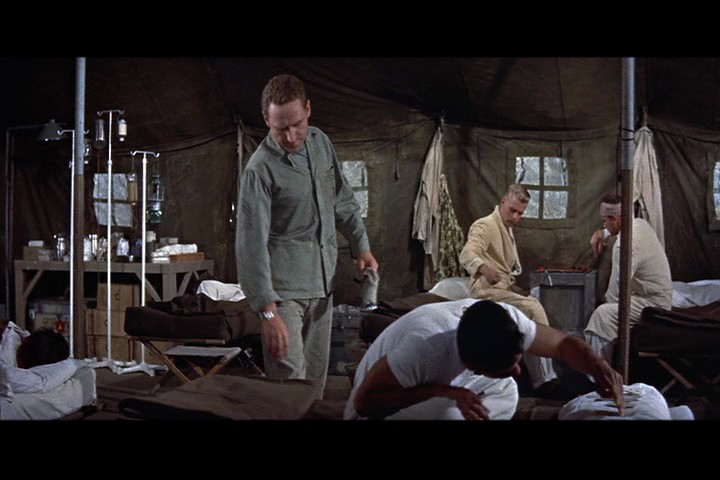

Supplement: S2 Dataset — (ZIP) [file pone.0264302.s002.zip › battle-cry-00202971.jpg]

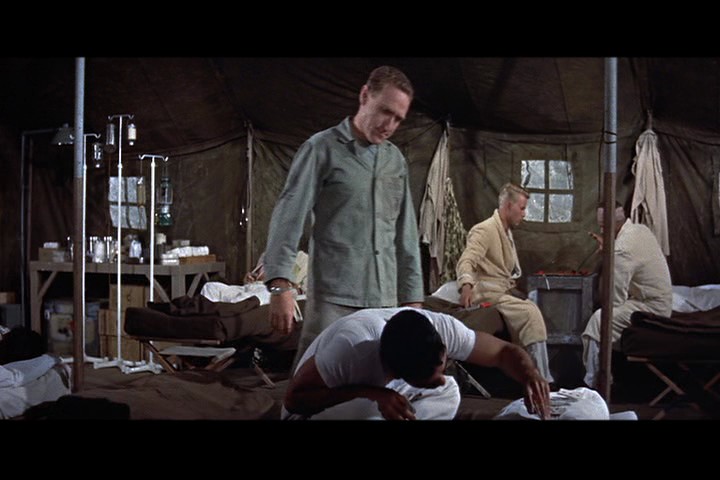

Supplement: S2 Dataset — (ZIP) [file pone.0264302.s002.zip › battle-cry-00203011.jpg]

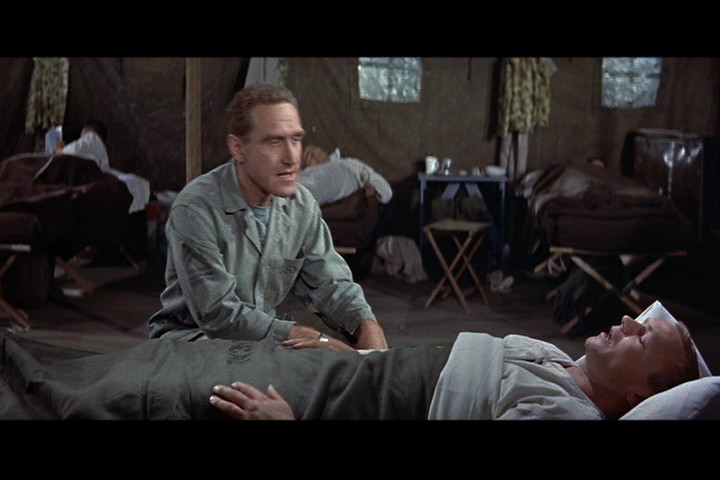

Supplement: S2 Dataset — (ZIP) [file pone.0264302.s002.zip › battle-cry-00204271.jpg]

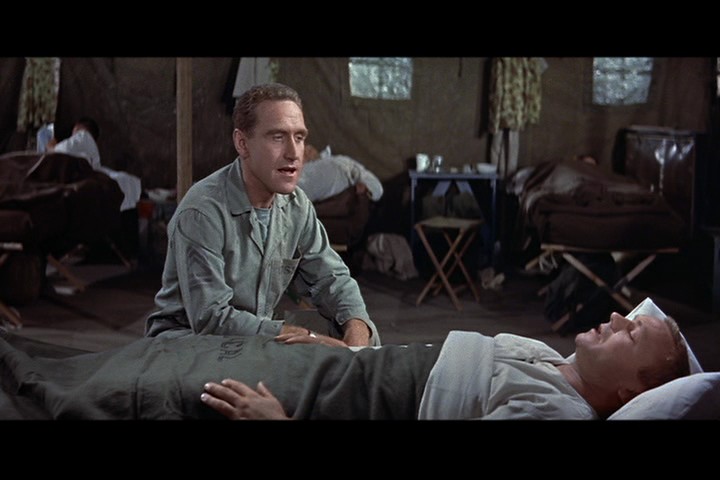

Supplement: S2 Dataset — (ZIP) [file pone.0264302.s002.zip › battle-cry-00204291.jpg]

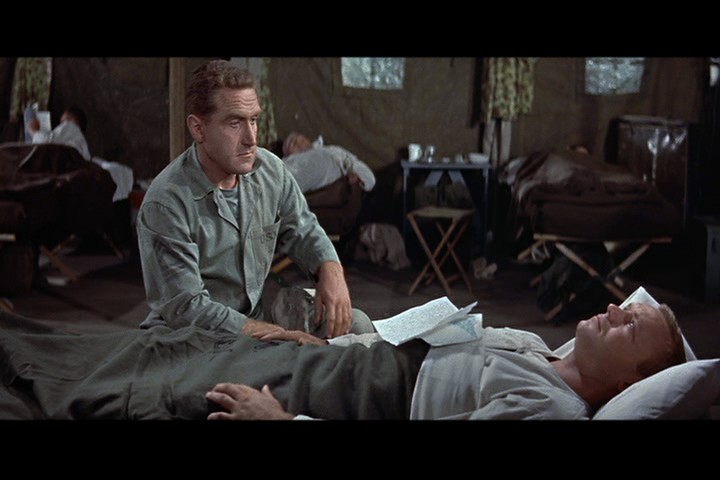

Supplement: S2 Dataset — (ZIP) [file pone.0264302.s002.zip › battle-cry-00207991.jpg]

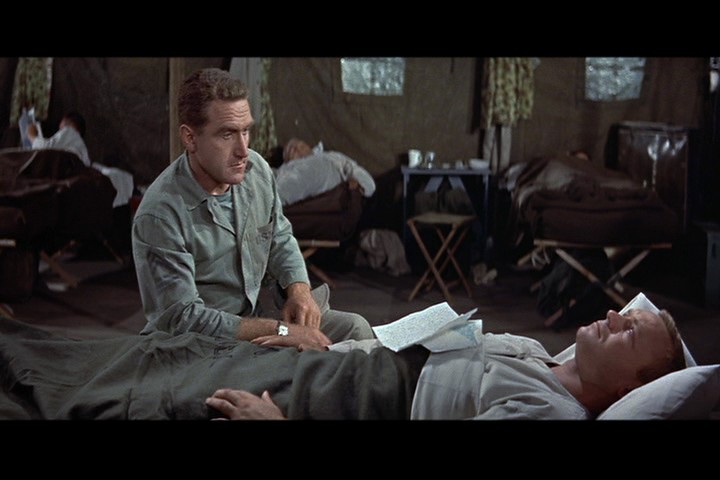

Supplement: S2 Dataset — (ZIP) [file pone.0264302.s002.zip › battle-cry-00208021.jpg]

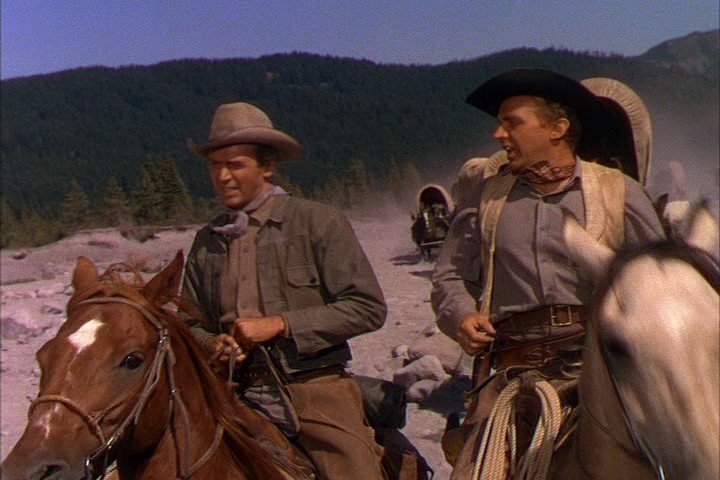

Supplement: S2 Dataset — (ZIP) [file pone.0264302.s002.zip › bend-of-the-river-00028761.jpg]

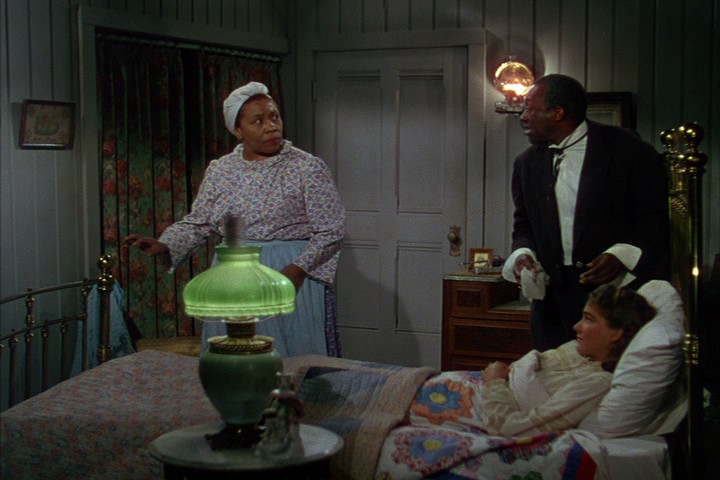

Supplement: S2 Dataset — (ZIP) [file pone.0264302.s002.zip › bend-of-the-river-00035781.jpg]

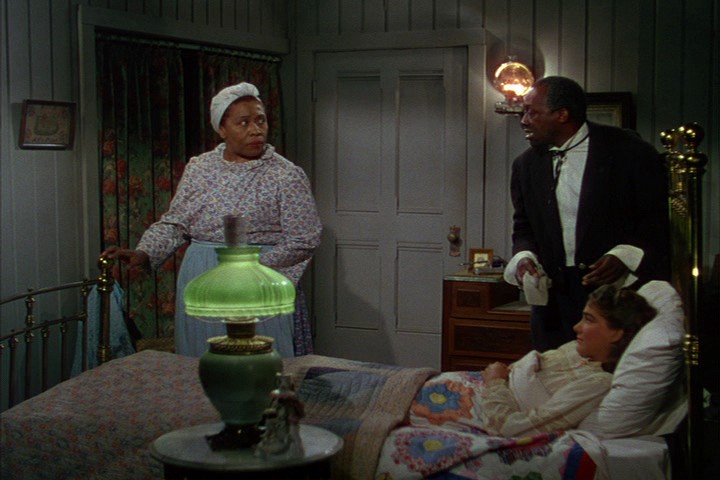

Supplement: S2 Dataset — (ZIP) [file pone.0264302.s002.zip › bend-of-the-river-00035791.jpg]

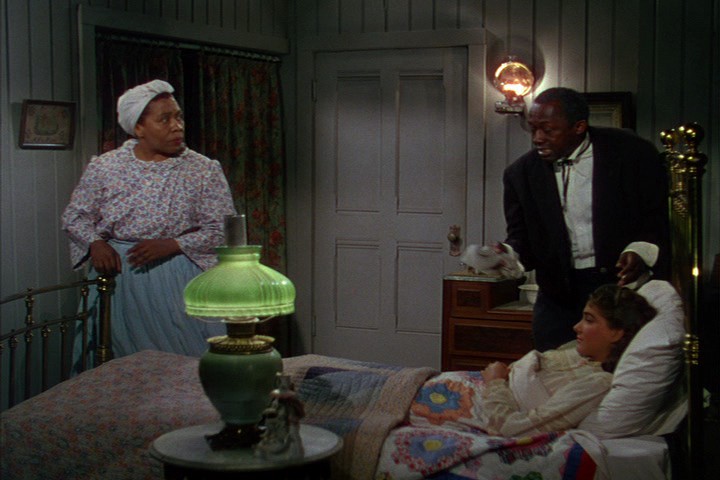

Supplement: S2 Dataset — (ZIP) [file pone.0264302.s002.zip › bend-of-the-river-00035821.jpg]

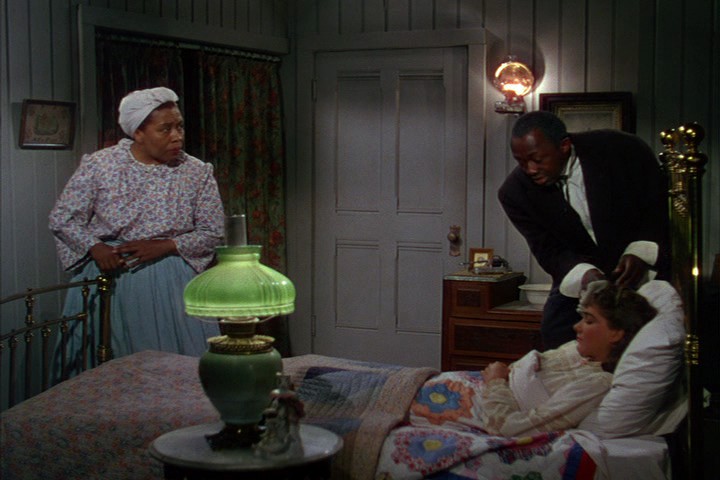

Supplement: S2 Dataset — (ZIP) [file pone.0264302.s002.zip › bend-of-the-river-00035871.jpg]

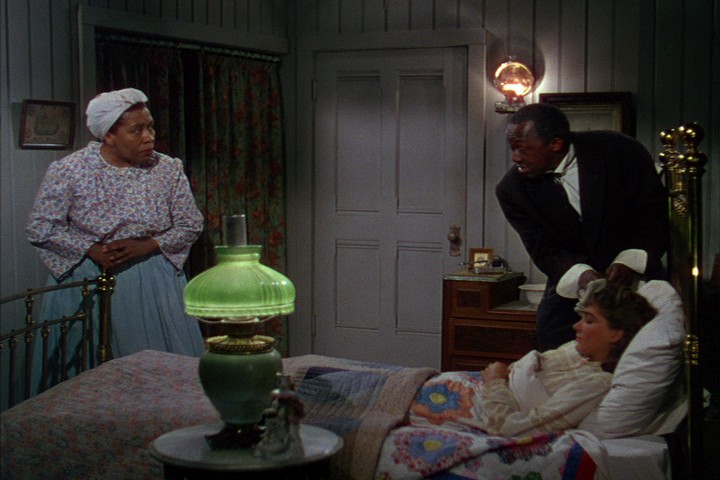

Supplement: S2 Dataset — (ZIP) [file pone.0264302.s002.zip › bend-of-the-river-00035881.jpg]

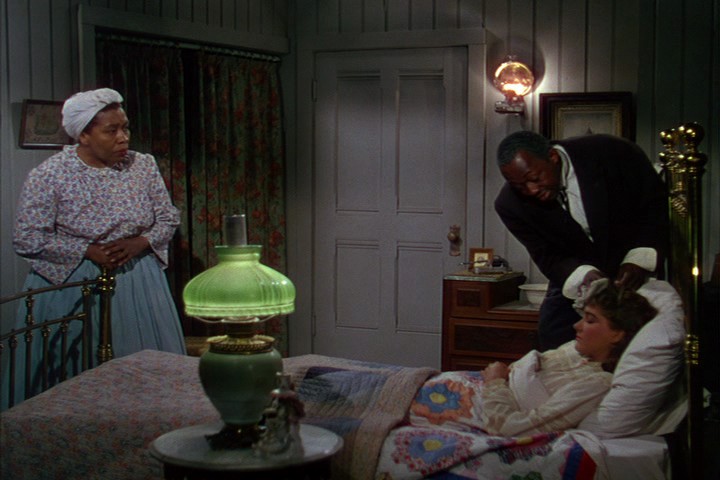

Supplement: S2 Dataset — (ZIP) [file pone.0264302.s002.zip › bend-of-the-river-00035901.jpg]

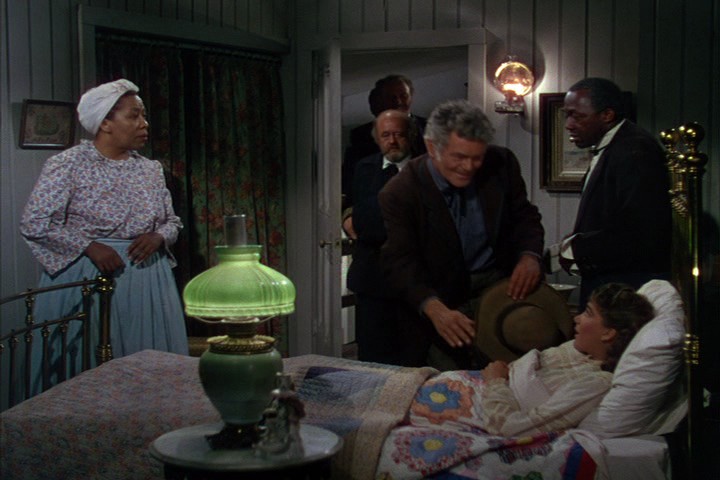

Supplement: S2 Dataset — (ZIP) [file pone.0264302.s002.zip › bend-of-the-river-00036071.jpg]

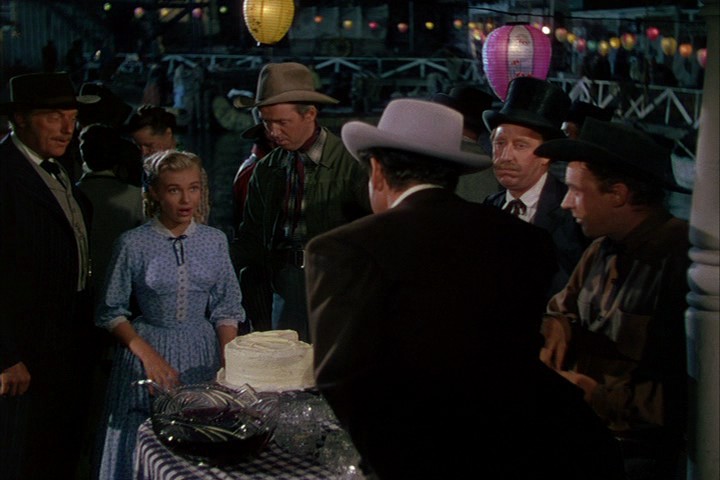

Supplement: S2 Dataset — (ZIP) [file pone.0264302.s002.zip › bend-of-the-river-00039371.jpg]

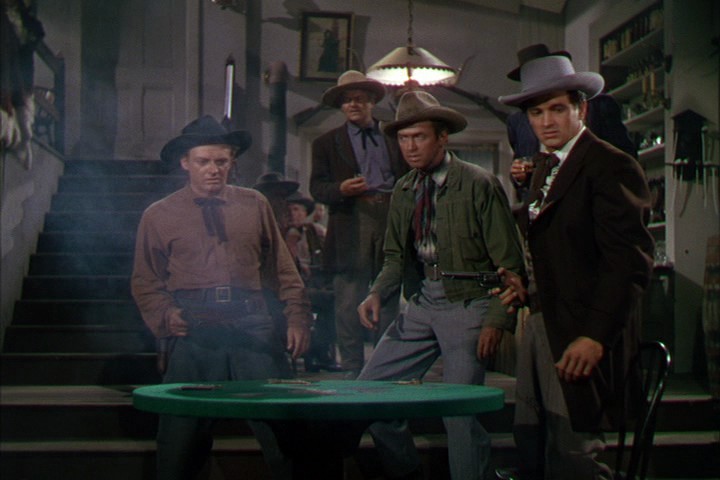

Supplement: S2 Dataset — (ZIP) [file pone.0264302.s002.zip › bend-of-the-river-00041891.jpg]

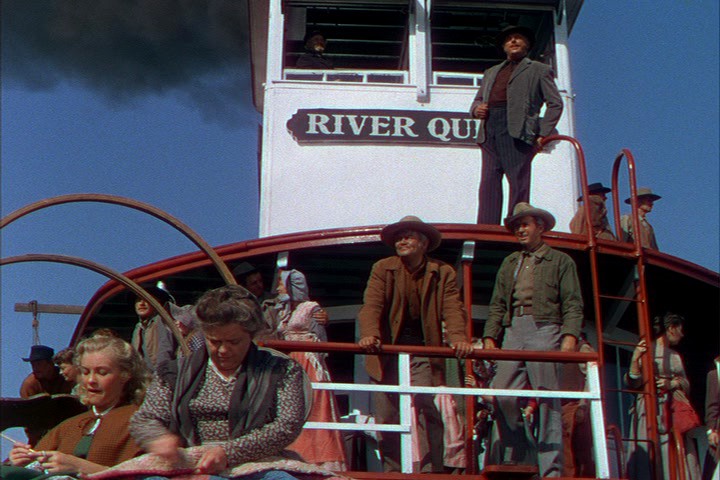

Supplement: S2 Dataset — (ZIP) [file pone.0264302.s002.zip › bend-of-the-river-00047051.jpg]

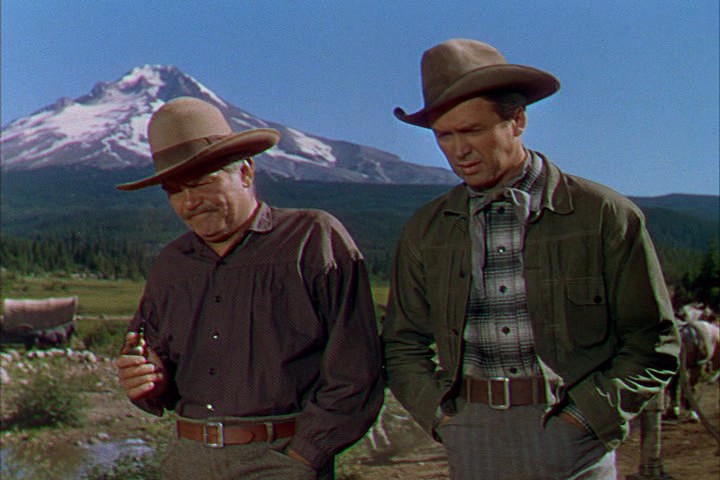

Supplement: S2 Dataset — (ZIP) [file pone.0264302.s002.zip › bend-of-the-river-00050901.jpg]

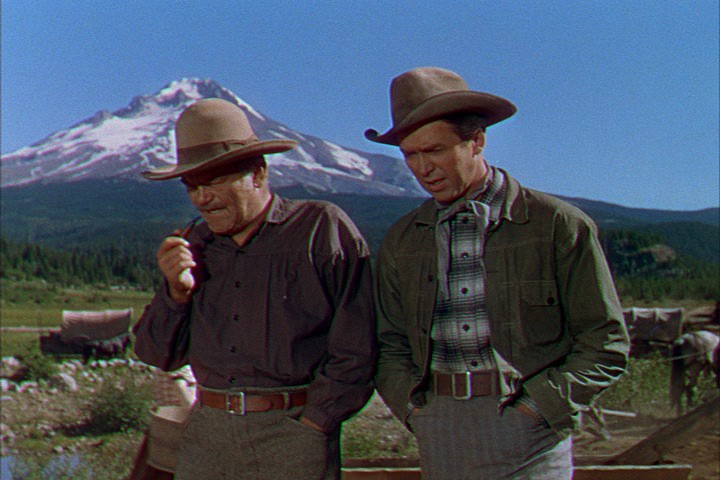

Supplement: S2 Dataset — (ZIP) [file pone.0264302.s002.zip › bend-of-the-river-00050941.jpg]

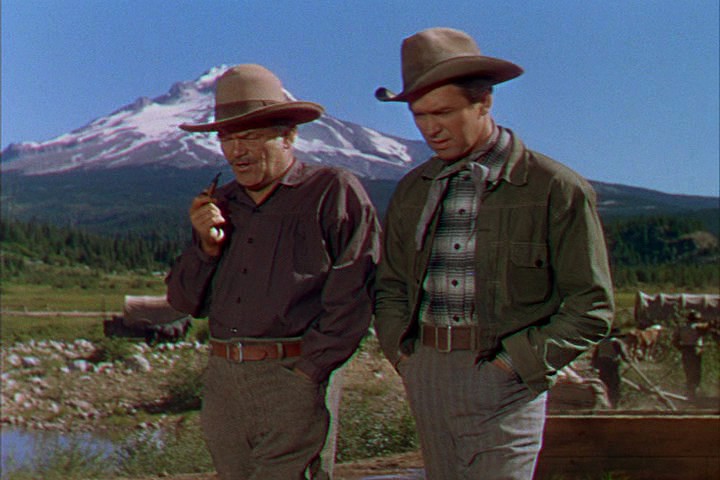

Supplement: S2 Dataset — (ZIP) [file pone.0264302.s002.zip › bend-of-the-river-00050991.jpg]

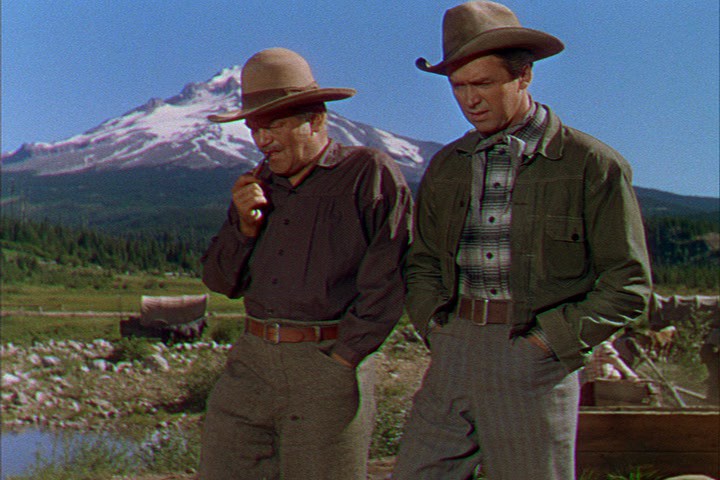

Supplement: S2 Dataset — (ZIP) [file pone.0264302.s002.zip › bend-of-the-river-00051001.jpg]

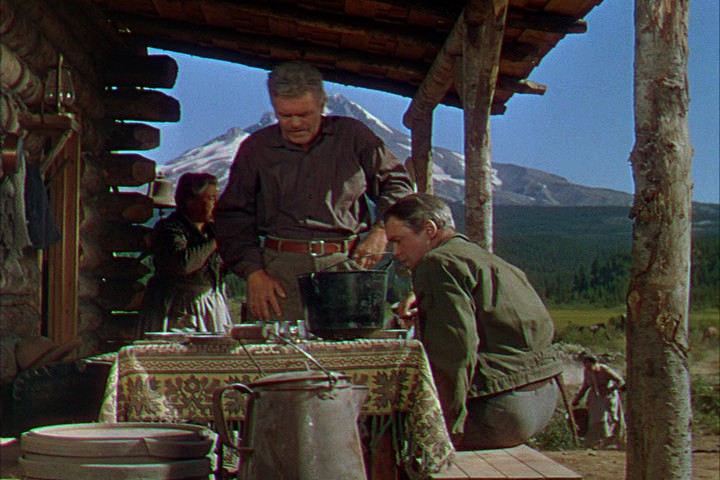

Supplement: S2 Dataset — (ZIP) [file pone.0264302.s002.zip › bend-of-the-river-00051921.jpg]

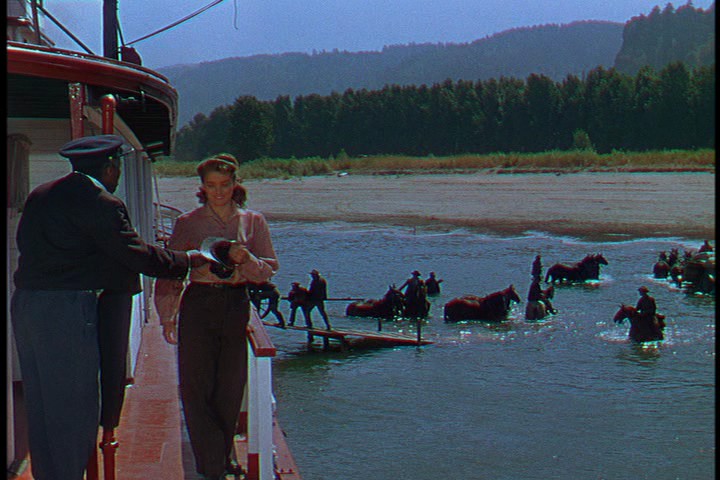

Supplement: S2 Dataset — (ZIP) [file pone.0264302.s002.zip › bend-of-the-river-00072211.jpg]

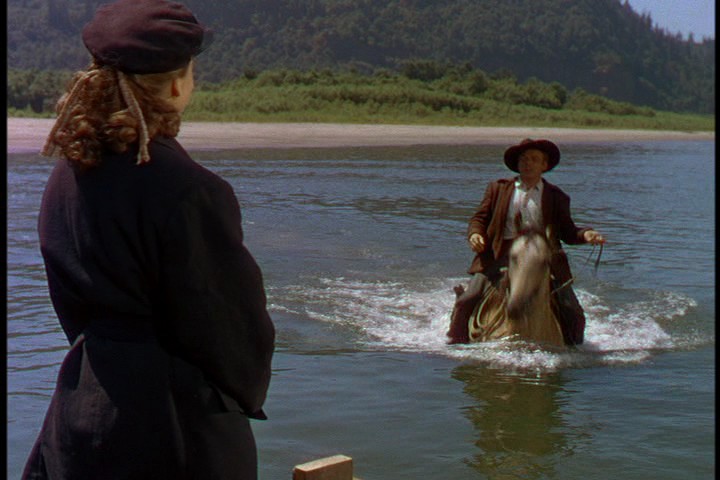

Supplement: S2 Dataset — (ZIP) [file pone.0264302.s002.zip › bend-of-the-river-00073111.jpg]

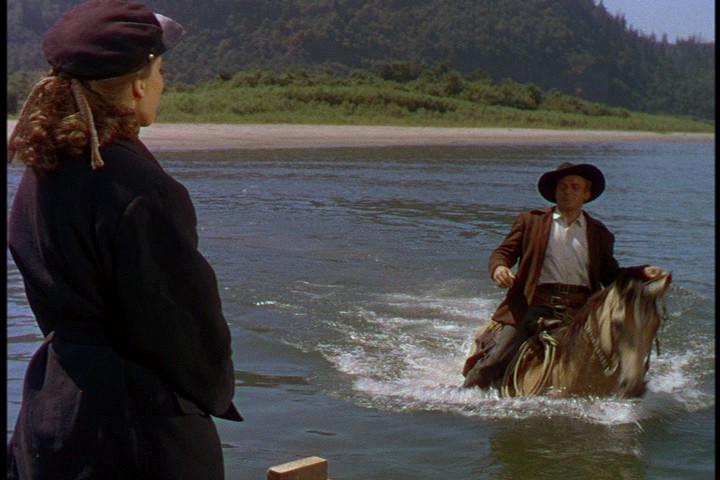

Supplement: S2 Dataset — (ZIP) [file pone.0264302.s002.zip › bend-of-the-river-00073131.jpg]

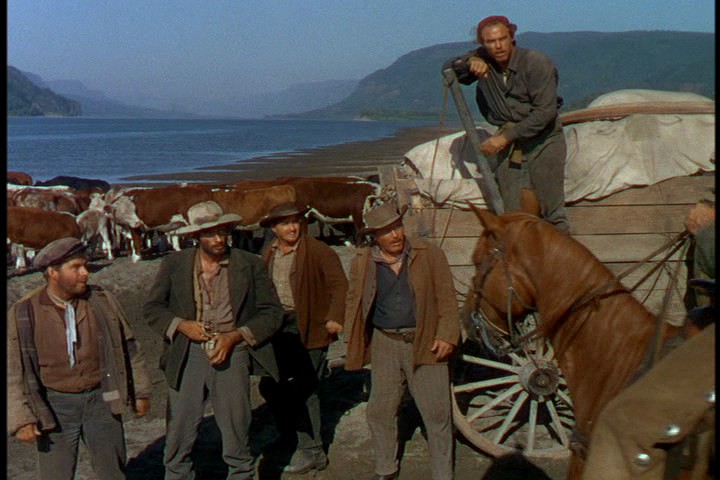

Supplement: S2 Dataset — (ZIP) [file pone.0264302.s002.zip › bend-of-the-river-00074031.jpg]

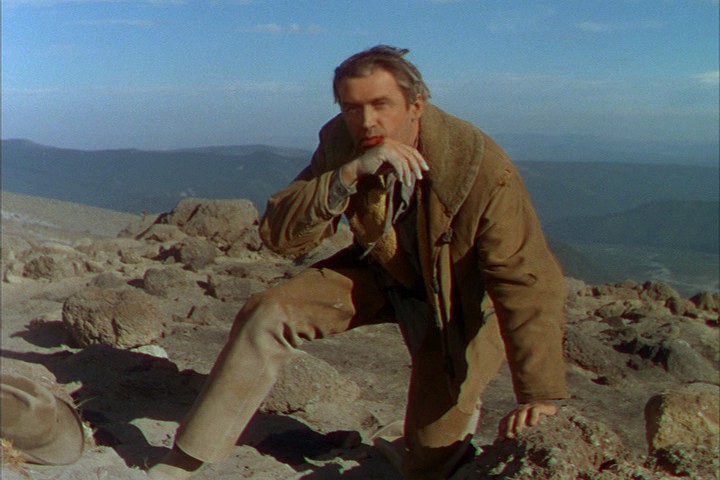

Supplement: S2 Dataset — (ZIP) [file pone.0264302.s002.zip › bend-of-the-river-00102611.jpg]

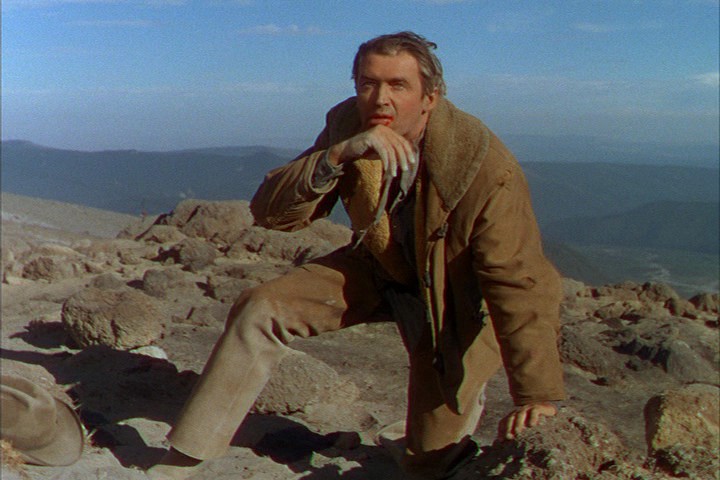

Supplement: S2 Dataset — (ZIP) [file pone.0264302.s002.zip › bend-of-the-river-00102621.jpg]

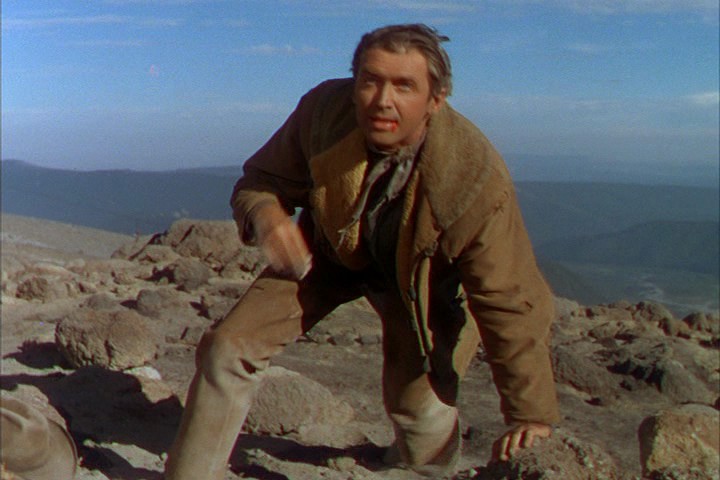

Supplement: S2 Dataset — (ZIP) [file pone.0264302.s002.zip › bend-of-the-river-00102641.jpg]

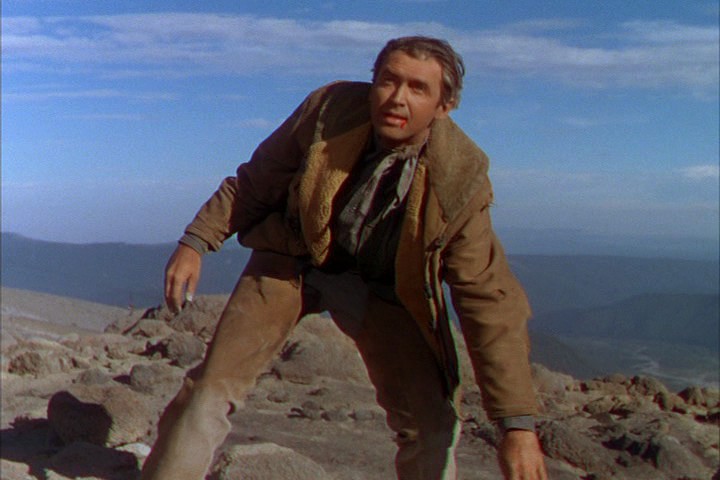

Supplement: S2 Dataset — (ZIP) [file pone.0264302.s002.zip › bend-of-the-river-00102671.jpg]
