# Supplementary figures and images for: LHPE-nets: A lightweight 2D and 3D human pose estimation model with well-structural deep networks and multi-view pose sample simplification method (part 3 of 8)
Source: PLoS One. 2022 Feb 23;17(2):e0264302. doi: 10.1371/journal.pone.0264302 (PMC8865690; doi:10.1371/journal.pone.0264302)

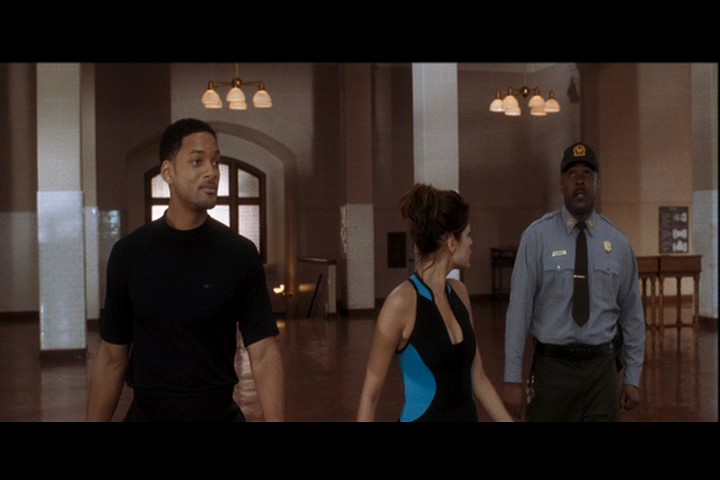

Supplement: S2 Dataset — (ZIP) [file pone.0264302.s002.zip › hitch-00056551.jpg]

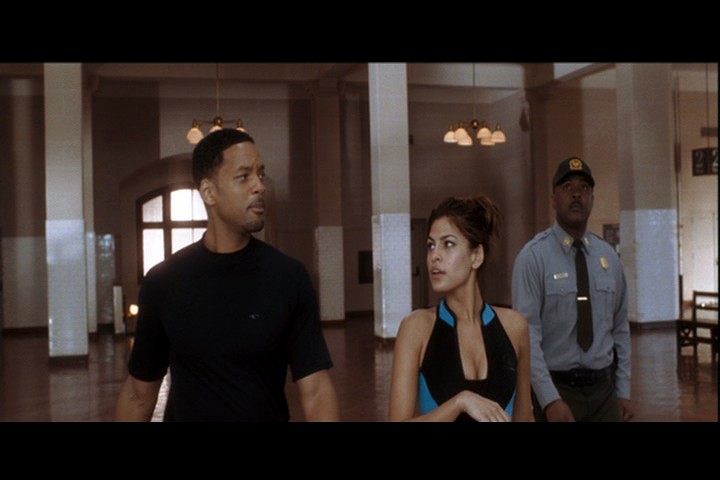

Supplement: S2 Dataset — (ZIP) [file pone.0264302.s002.zip › hitch-00056621.jpg]

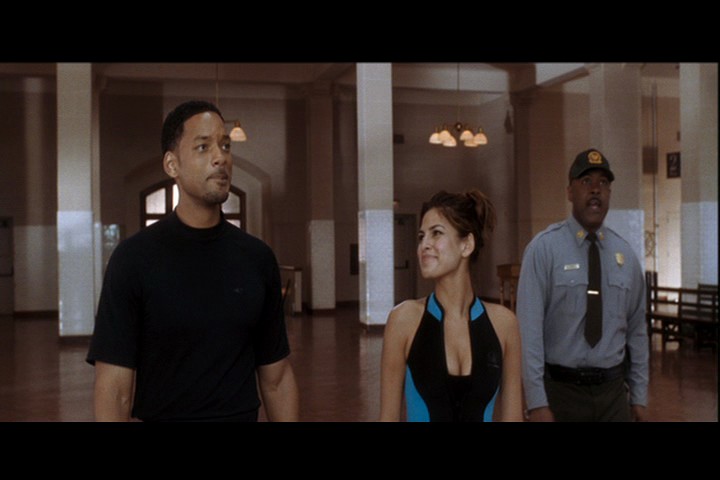

Supplement: S2 Dataset — (ZIP) [file pone.0264302.s002.zip › hitch-00056661.jpg]

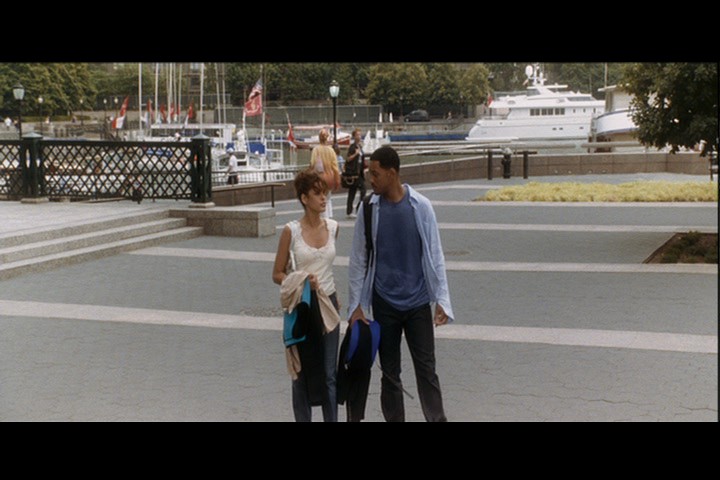

Supplement: S2 Dataset — (ZIP) [file pone.0264302.s002.zip › hitch-00061221.jpg]

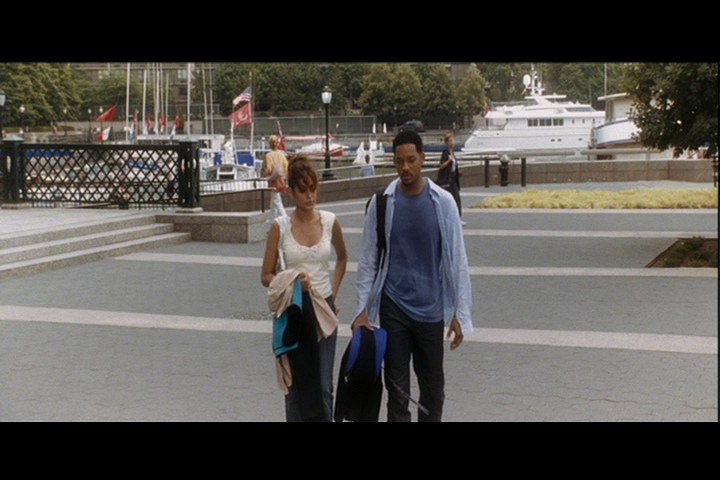

Supplement: S2 Dataset — (ZIP) [file pone.0264302.s002.zip › hitch-00061251.jpg]

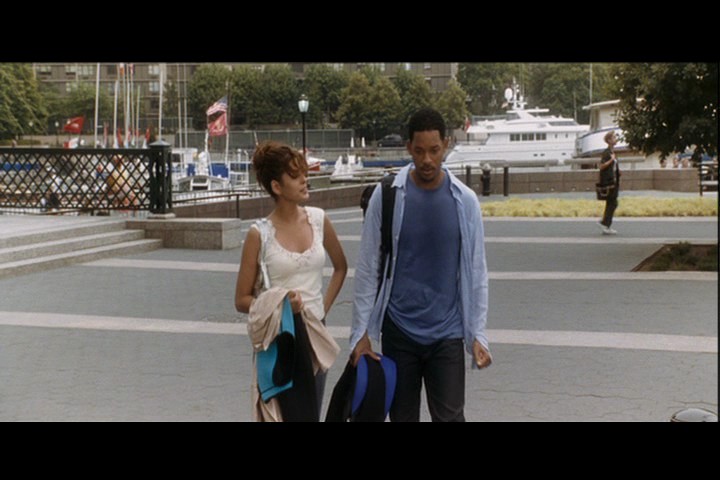

Supplement: S2 Dataset — (ZIP) [file pone.0264302.s002.zip › hitch-00061301.jpg]

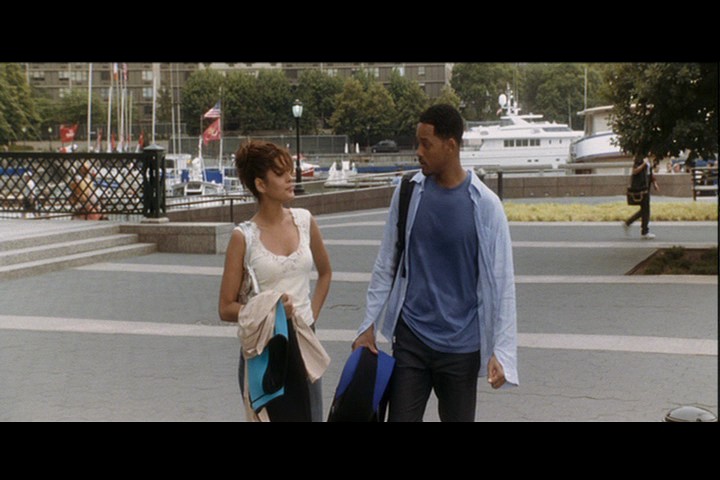

Supplement: S2 Dataset — (ZIP) [file pone.0264302.s002.zip › hitch-00061311.jpg]

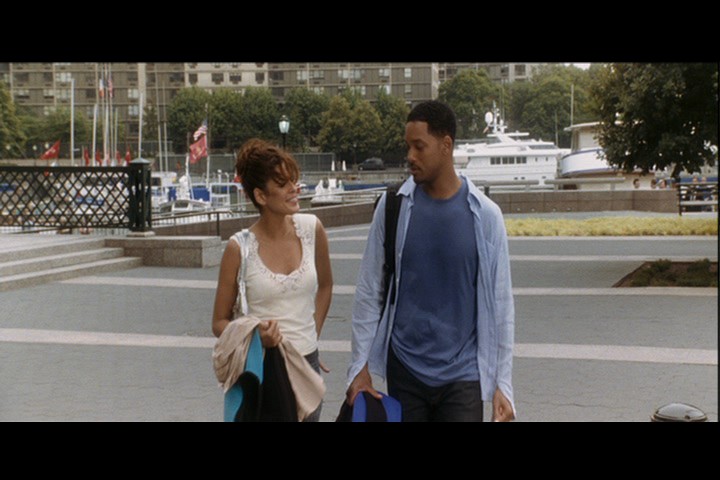

Supplement: S2 Dataset — (ZIP) [file pone.0264302.s002.zip › hitch-00061341.jpg]

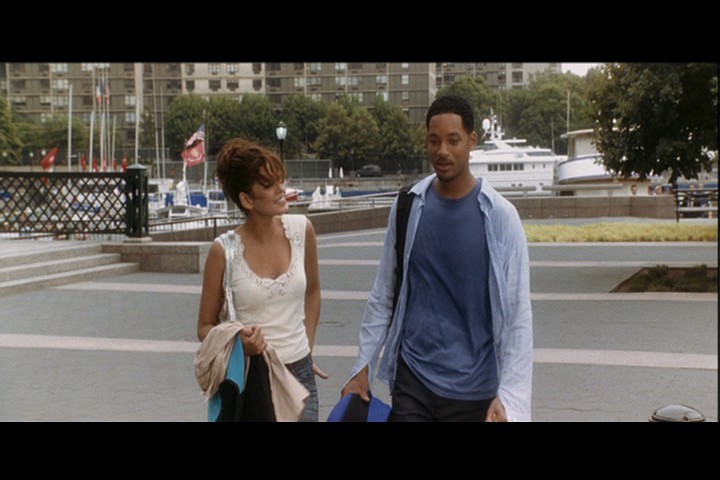

Supplement: S2 Dataset — (ZIP) [file pone.0264302.s002.zip › hitch-00061351.jpg]

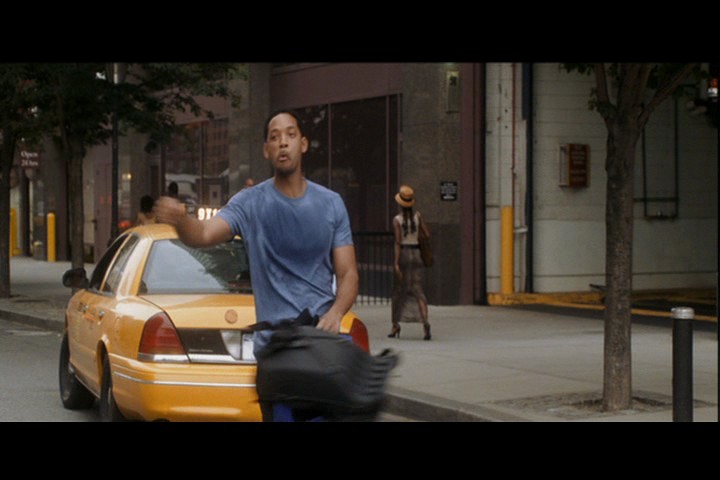

Supplement: S2 Dataset — (ZIP) [file pone.0264302.s002.zip › hitch-00061811.jpg]

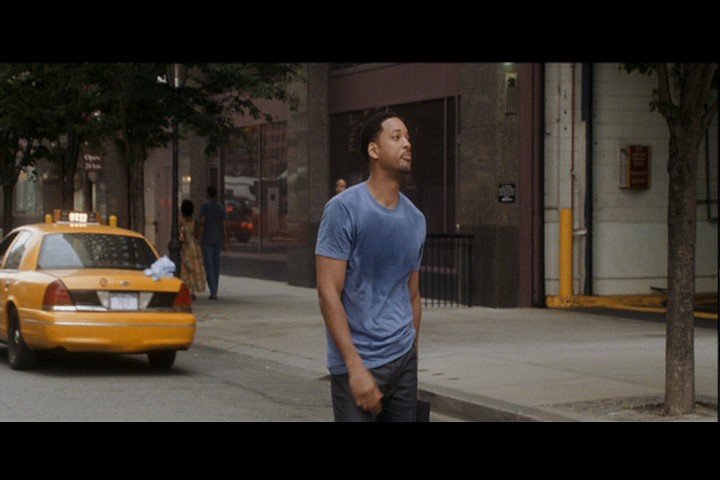

Supplement: S2 Dataset — (ZIP) [file pone.0264302.s002.zip › hitch-00061851.jpg]

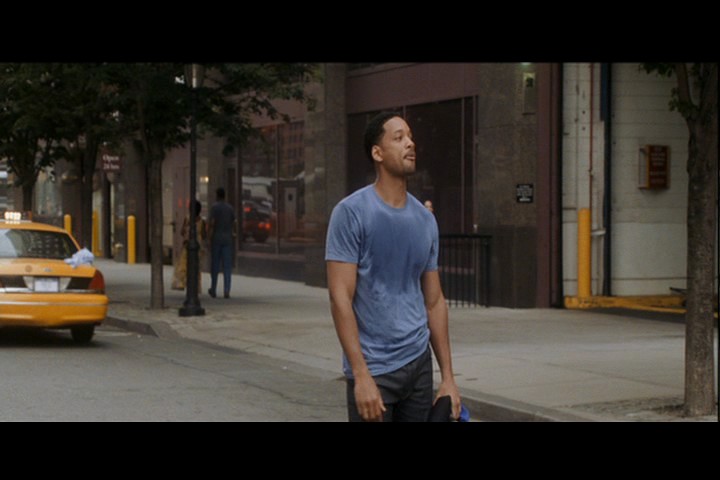

Supplement: S2 Dataset — (ZIP) [file pone.0264302.s002.zip › hitch-00061871.jpg]

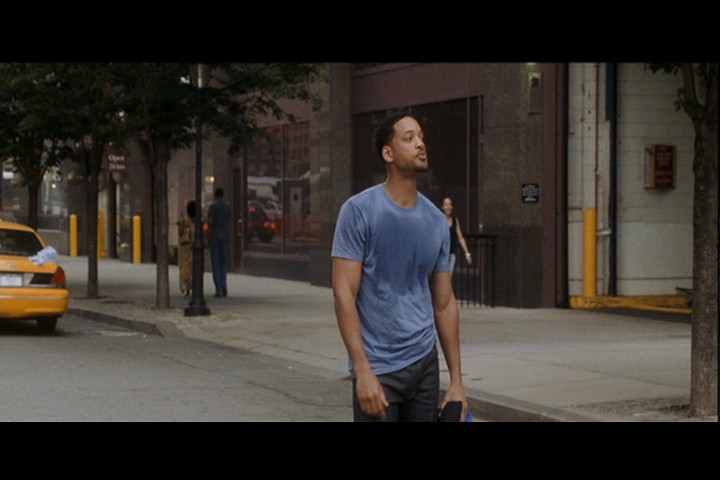

Supplement: S2 Dataset — (ZIP) [file pone.0264302.s002.zip › hitch-00061881.jpg]

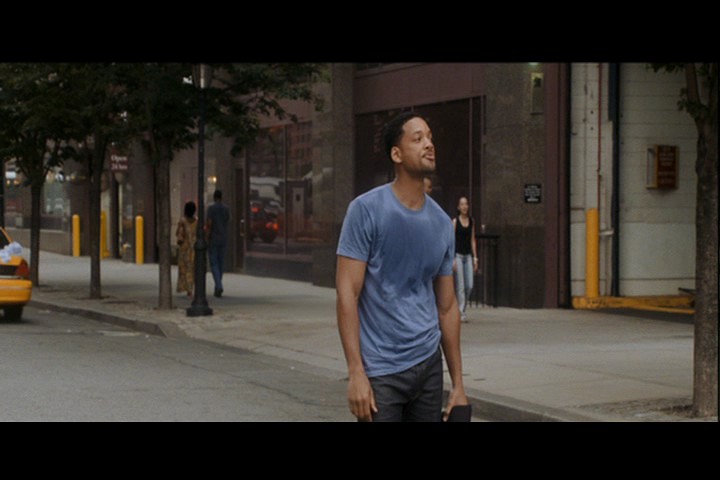

Supplement: S2 Dataset — (ZIP) [file pone.0264302.s002.zip › hitch-00061891.jpg]

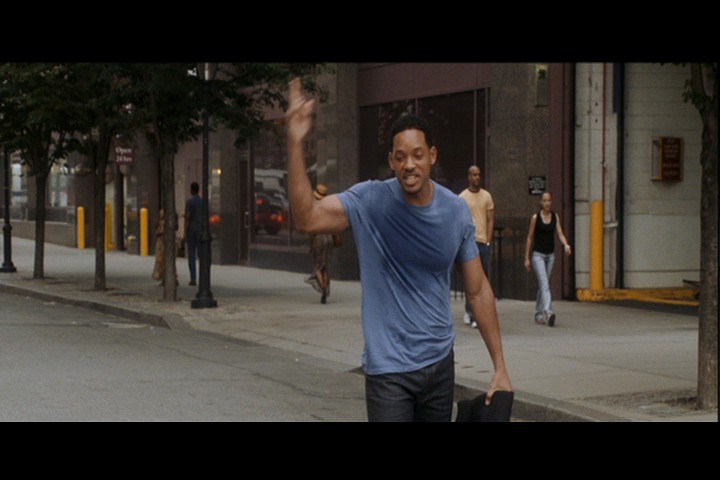

Supplement: S2 Dataset — (ZIP) [file pone.0264302.s002.zip › hitch-00061941.jpg]

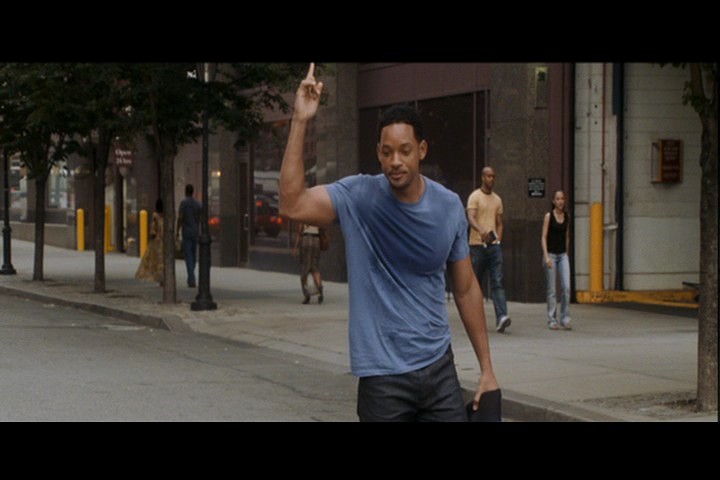

Supplement: S2 Dataset — (ZIP) [file pone.0264302.s002.zip › hitch-00061951.jpg]

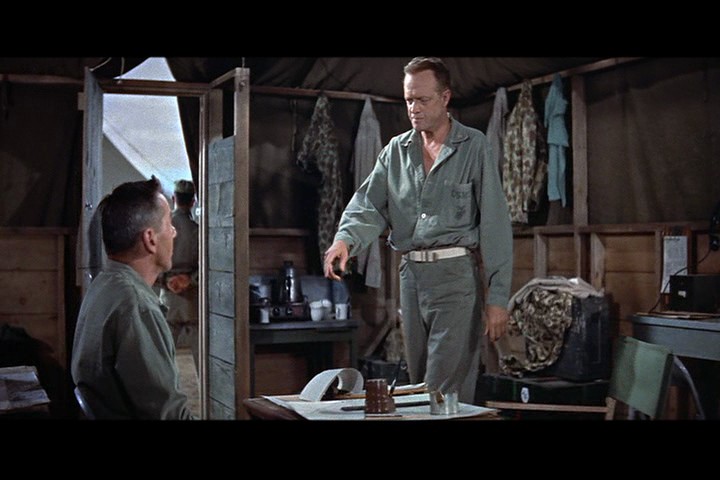

Supplement: S2 Dataset — (ZIP) [file pone.0264302.s002.zip › battle-cry-00135601.jpg]

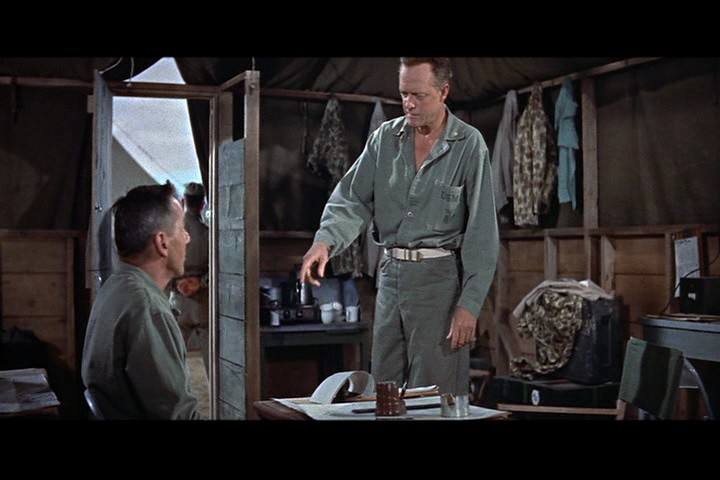

Supplement: S2 Dataset — (ZIP) [file pone.0264302.s002.zip › battle-cry-00135611.jpg]

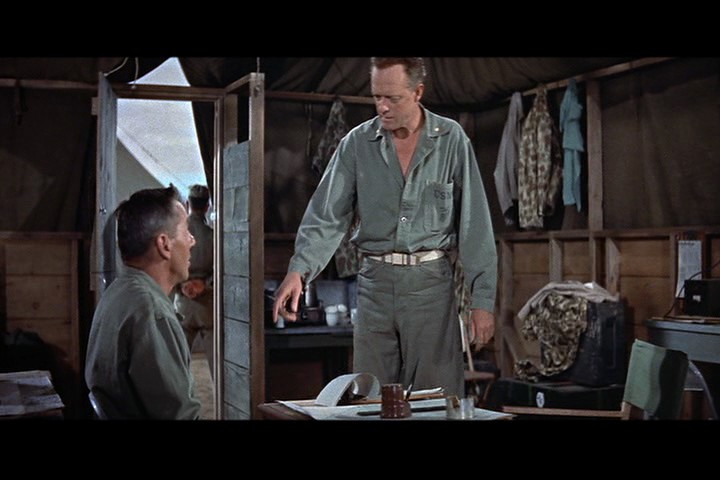

Supplement: S2 Dataset — (ZIP) [file pone.0264302.s002.zip › battle-cry-00135621.jpg]

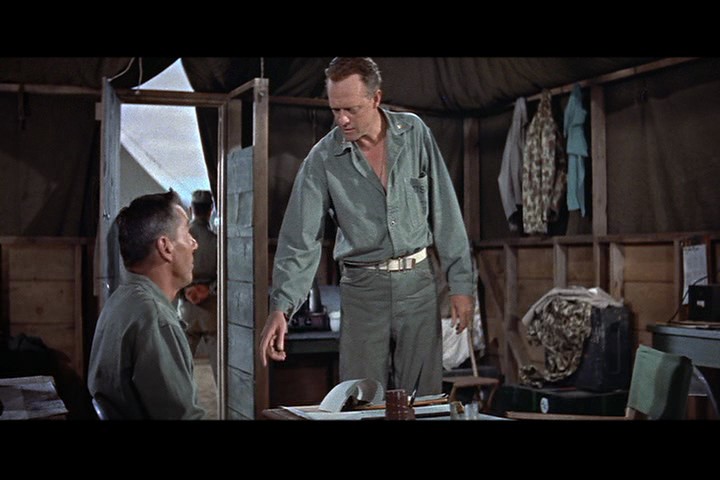

Supplement: S2 Dataset — (ZIP) [file pone.0264302.s002.zip › battle-cry-00135631.jpg]

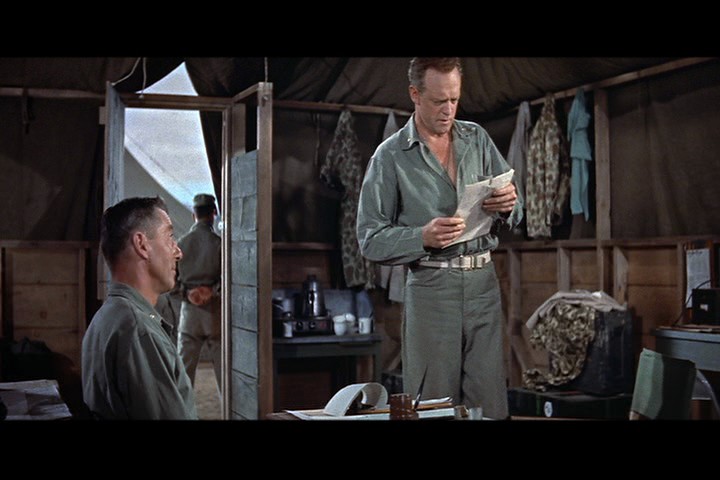

Supplement: S2 Dataset — (ZIP) [file pone.0264302.s002.zip › battle-cry-00135751.jpg]

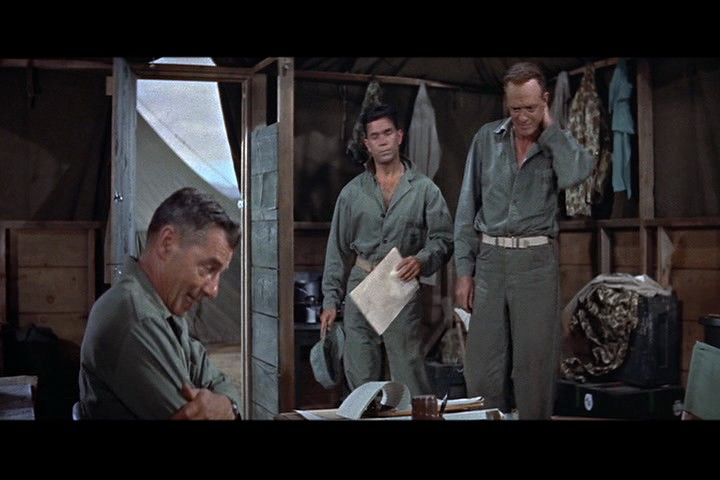

Supplement: S2 Dataset — (ZIP) [file pone.0264302.s002.zip › battle-cry-00136371.jpg]

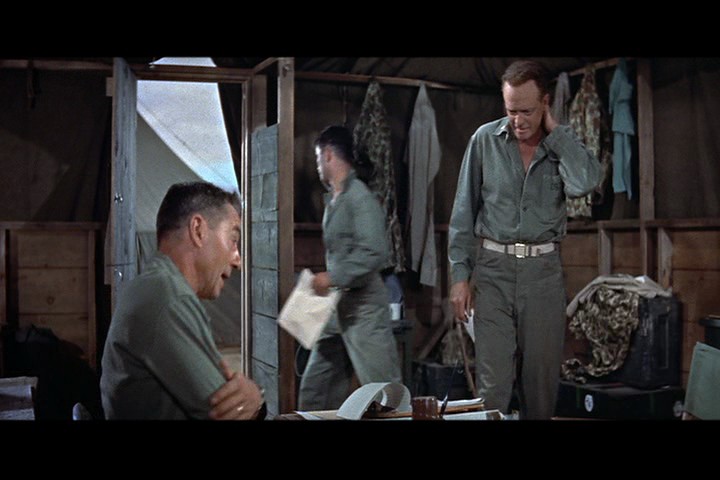

Supplement: S2 Dataset — (ZIP) [file pone.0264302.s002.zip › battle-cry-00136381.jpg]

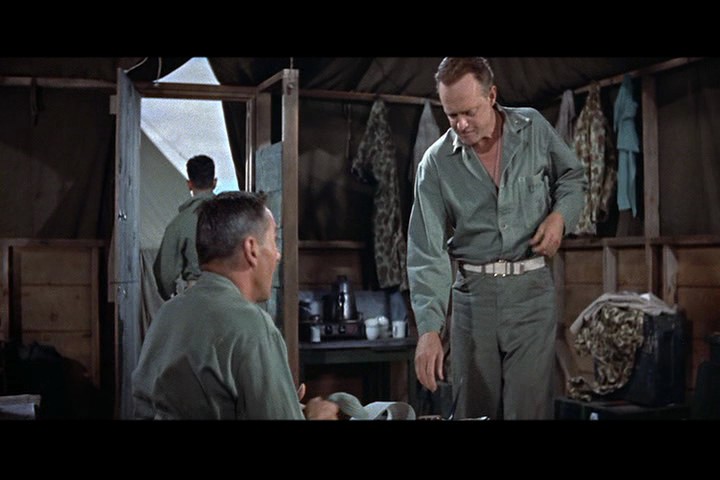

Supplement: S2 Dataset — (ZIP) [file pone.0264302.s002.zip › battle-cry-00136401.jpg]

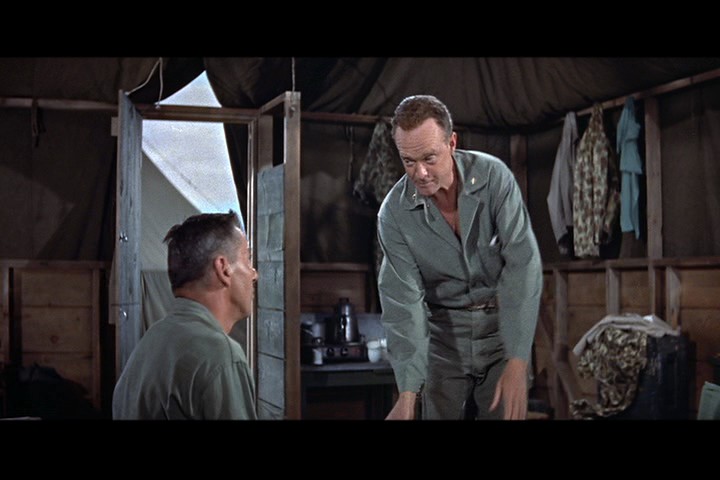

Supplement: S2 Dataset — (ZIP) [file pone.0264302.s002.zip › battle-cry-00136481.jpg]

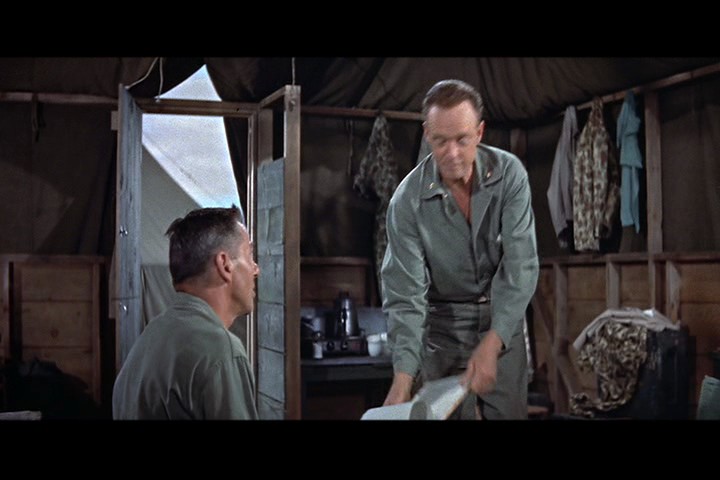

Supplement: S2 Dataset — (ZIP) [file pone.0264302.s002.zip › battle-cry-00136491.jpg]

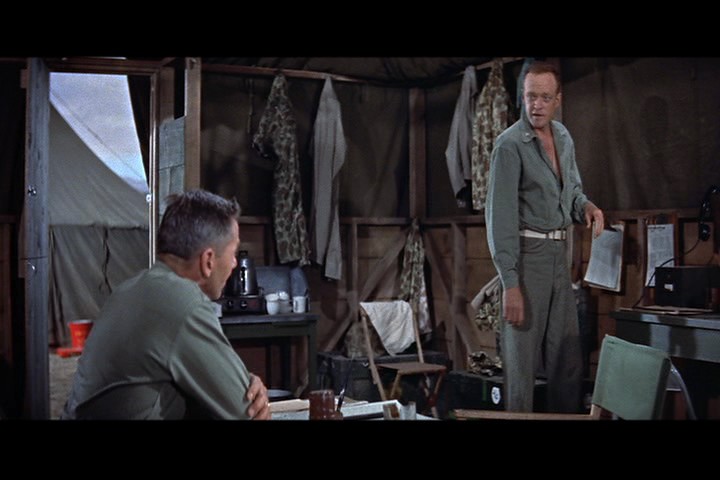

Supplement: S2 Dataset — (ZIP) [file pone.0264302.s002.zip › battle-cry-00136611.jpg]

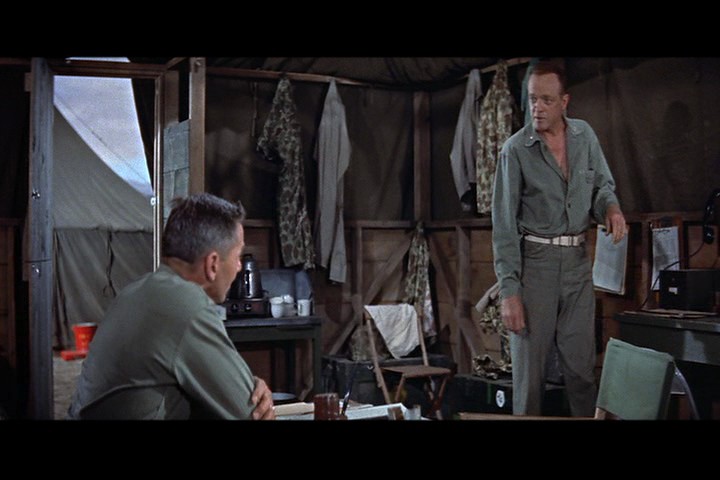

Supplement: S2 Dataset — (ZIP) [file pone.0264302.s002.zip › battle-cry-00136621.jpg]

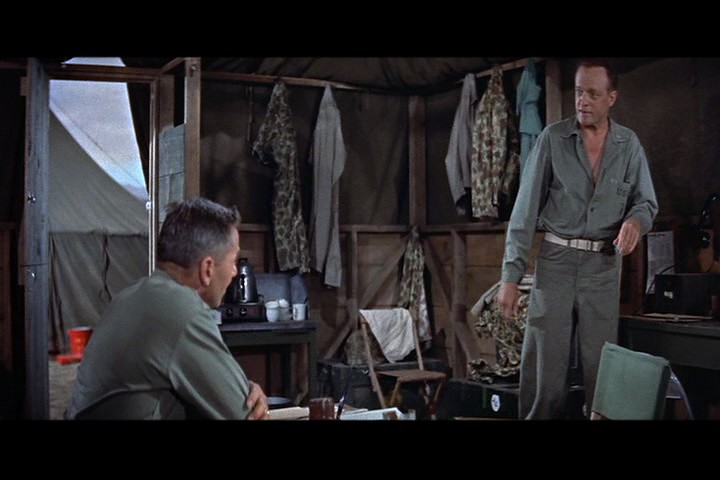

Supplement: S2 Dataset — (ZIP) [file pone.0264302.s002.zip › battle-cry-00136631.jpg]

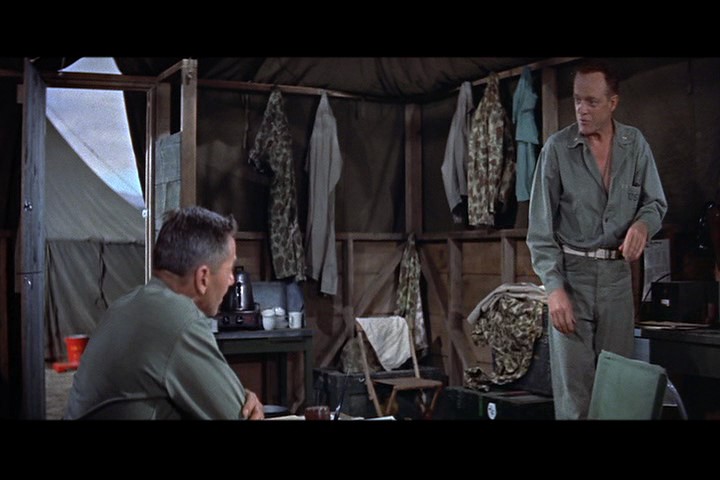

Supplement: S2 Dataset — (ZIP) [file pone.0264302.s002.zip › battle-cry-00136641.jpg]

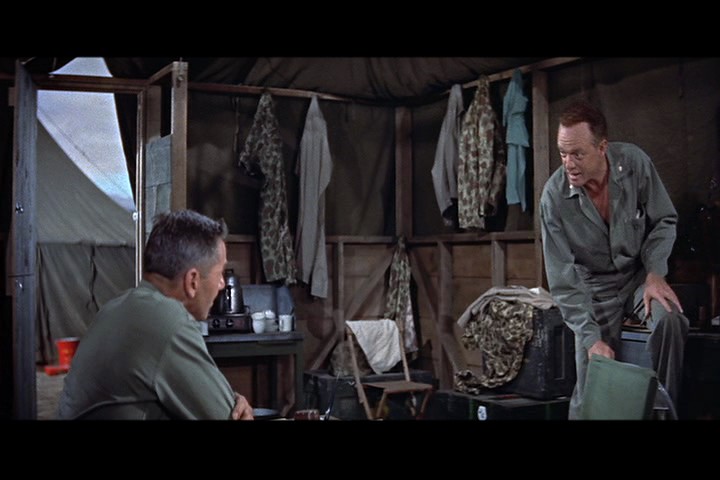

Supplement: S2 Dataset — (ZIP) [file pone.0264302.s002.zip › battle-cry-00136671.jpg]

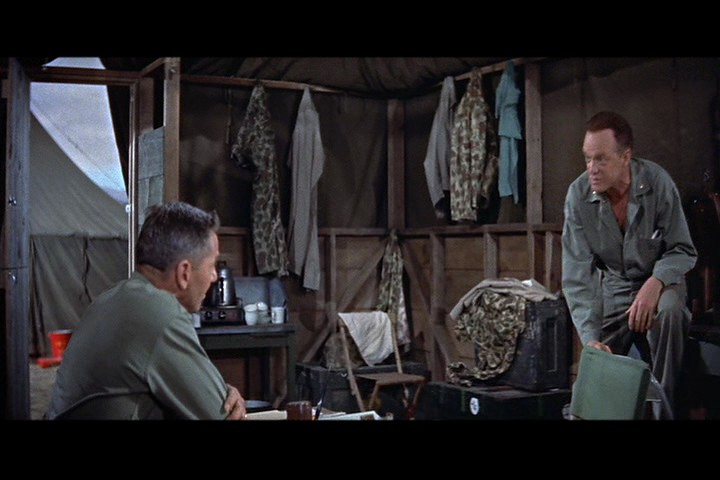

Supplement: S2 Dataset — (ZIP) [file pone.0264302.s002.zip › battle-cry-00136681.jpg]

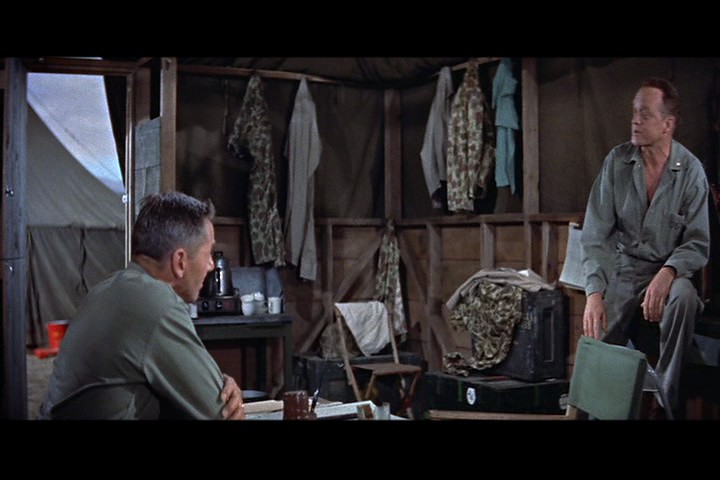

Supplement: S2 Dataset — (ZIP) [file pone.0264302.s002.zip › battle-cry-00136691.jpg]

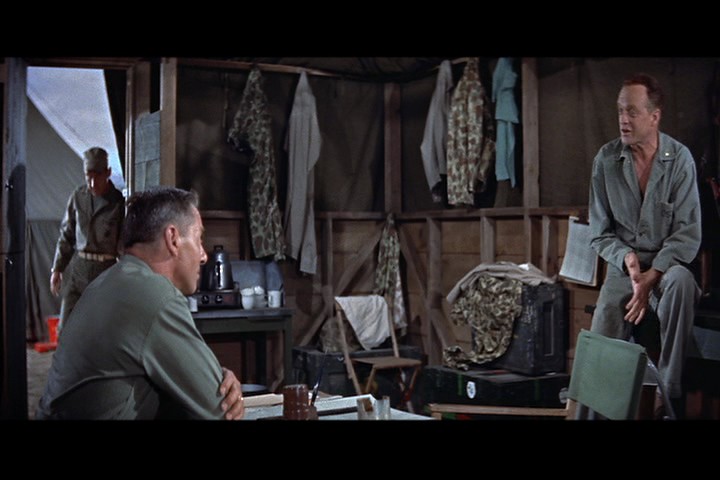

Supplement: S2 Dataset — (ZIP) [file pone.0264302.s002.zip › battle-cry-00136731.jpg]

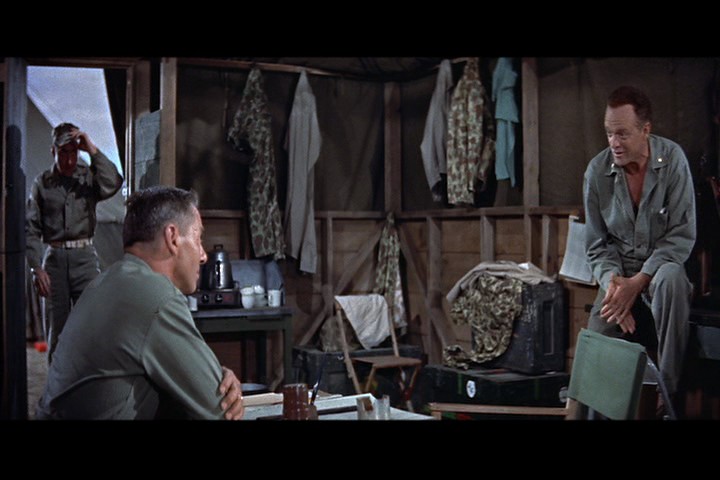

Supplement: S2 Dataset — (ZIP) [file pone.0264302.s002.zip › battle-cry-00136751.jpg]

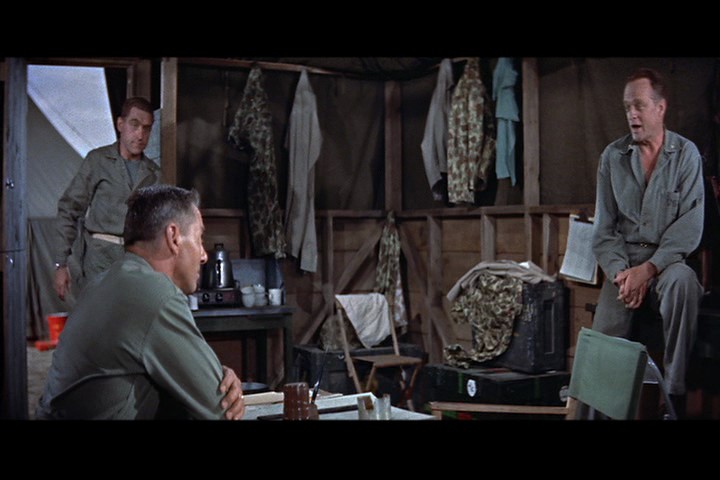

Supplement: S2 Dataset — (ZIP) [file pone.0264302.s002.zip › battle-cry-00136811.jpg]

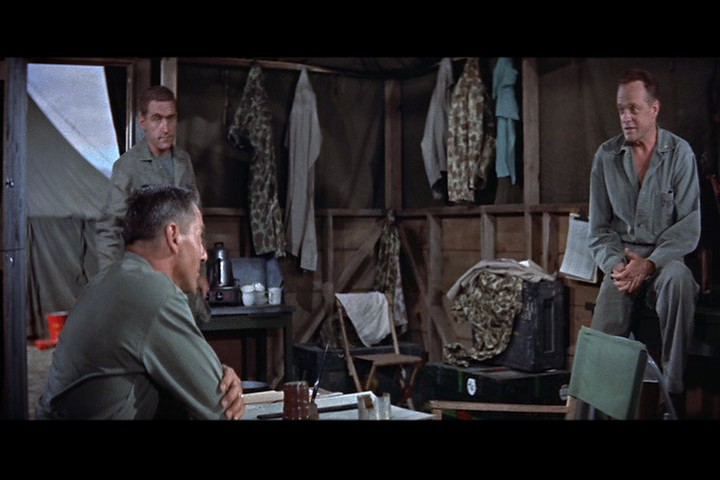

Supplement: S2 Dataset — (ZIP) [file pone.0264302.s002.zip › battle-cry-00136821.jpg]

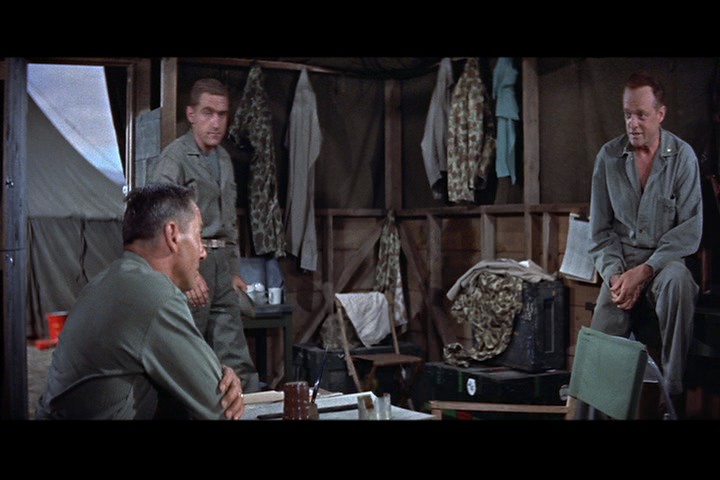

Supplement: S2 Dataset — (ZIP) [file pone.0264302.s002.zip › battle-cry-00136831.jpg]

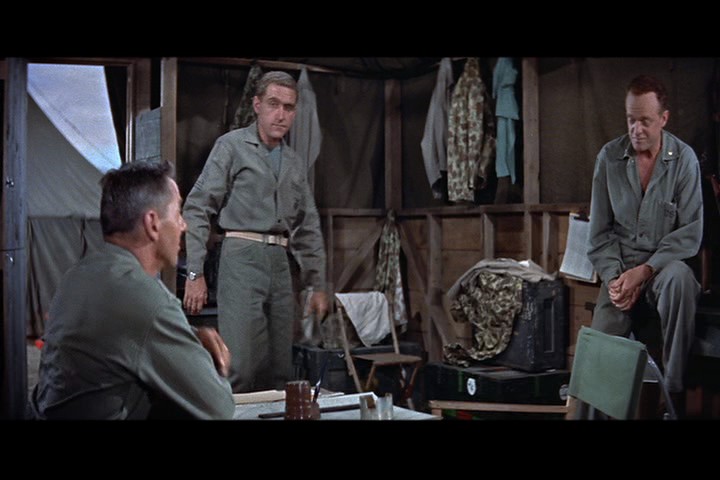

Supplement: S2 Dataset — (ZIP) [file pone.0264302.s002.zip › battle-cry-00136841.jpg]

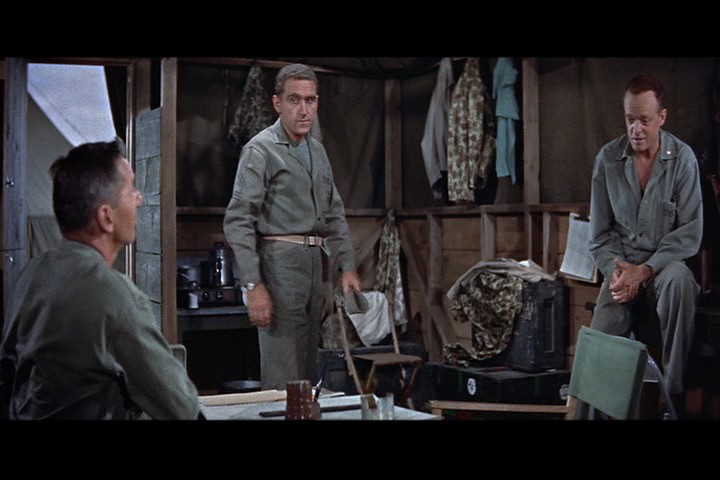

Supplement: S2 Dataset — (ZIP) [file pone.0264302.s002.zip › battle-cry-00136851.jpg]

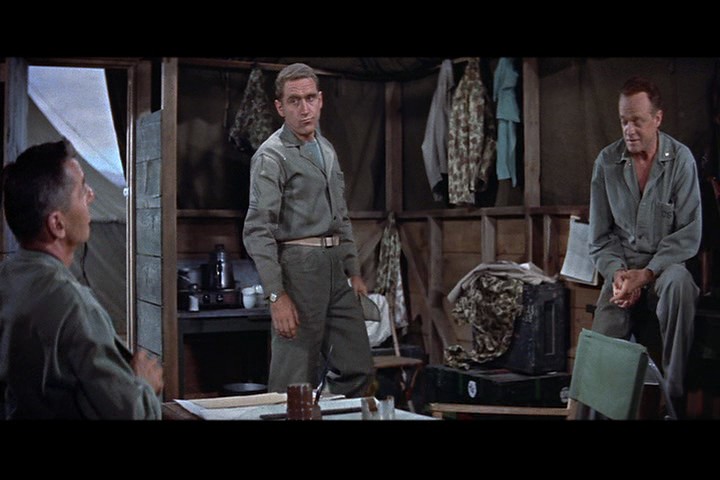

Supplement: S2 Dataset — (ZIP) [file pone.0264302.s002.zip › battle-cry-00136861.jpg]

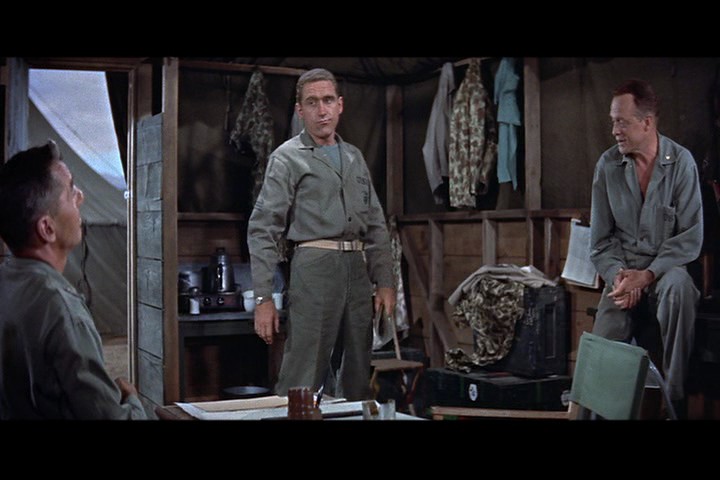

Supplement: S2 Dataset — (ZIP) [file pone.0264302.s002.zip › battle-cry-00136871.jpg]

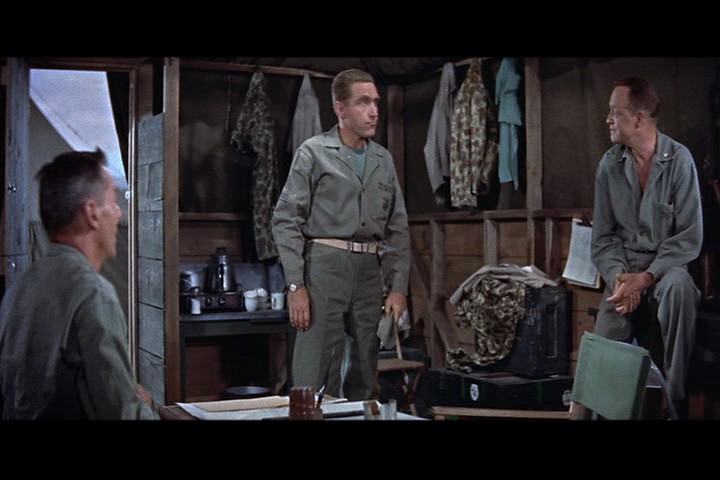

Supplement: S2 Dataset — (ZIP) [file pone.0264302.s002.zip › battle-cry-00136881.jpg]

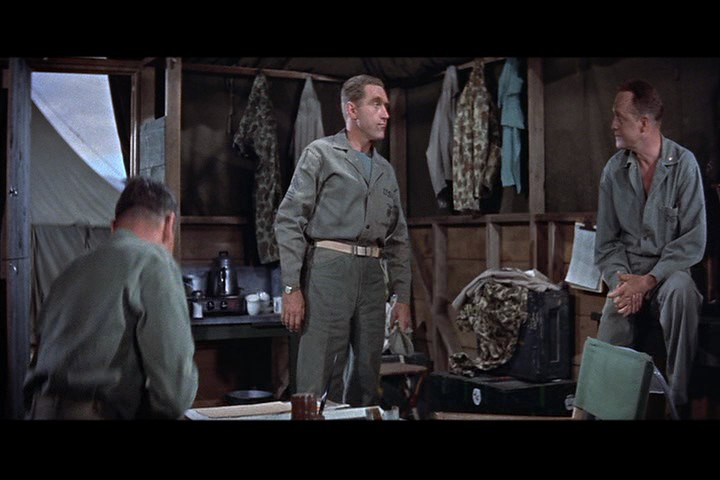

Supplement: S2 Dataset — (ZIP) [file pone.0264302.s002.zip › battle-cry-00136891.jpg]

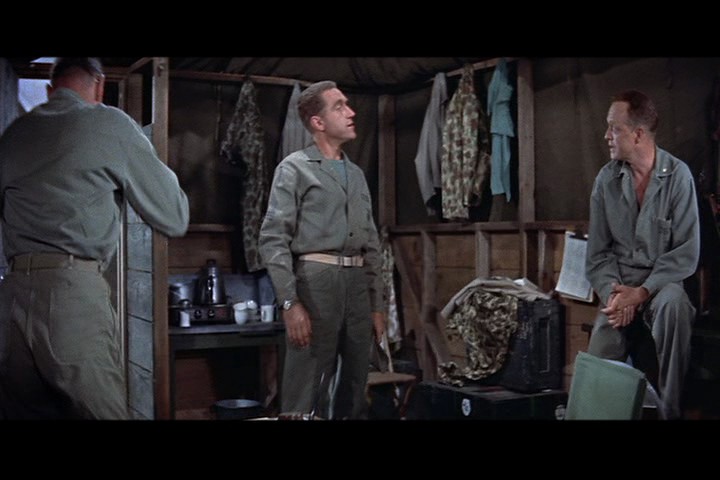

Supplement: S2 Dataset — (ZIP) [file pone.0264302.s002.zip › battle-cry-00136901.jpg]

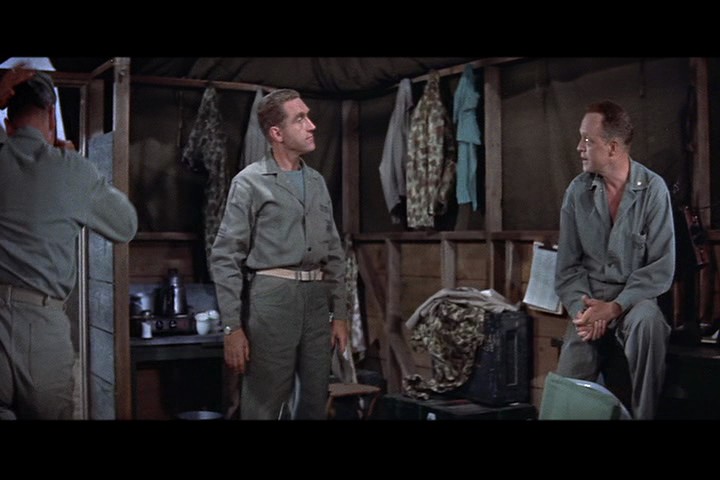

Supplement: S2 Dataset — (ZIP) [file pone.0264302.s002.zip › battle-cry-00136911.jpg]

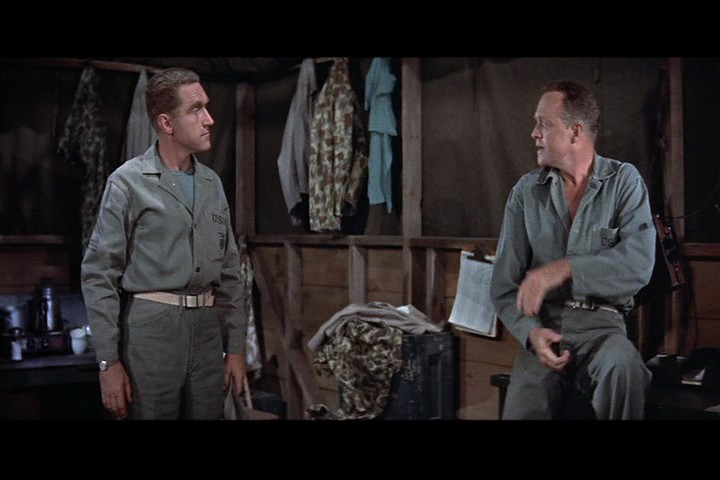

Supplement: S2 Dataset — (ZIP) [file pone.0264302.s002.zip › battle-cry-00136951.jpg]

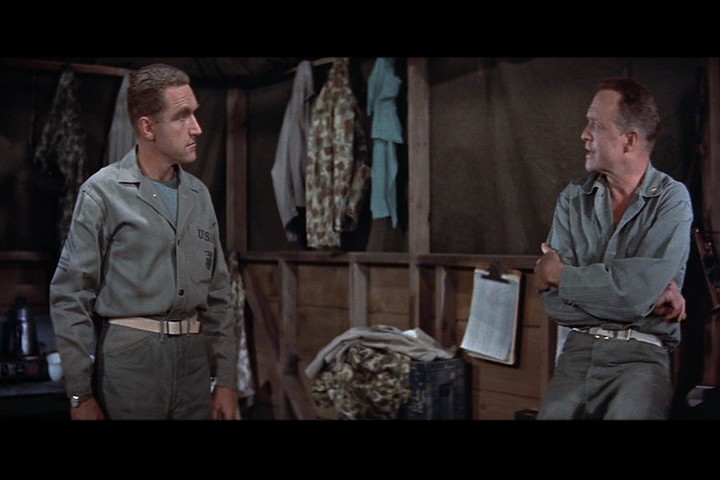

Supplement: S2 Dataset — (ZIP) [file pone.0264302.s002.zip › battle-cry-00137011.jpg]

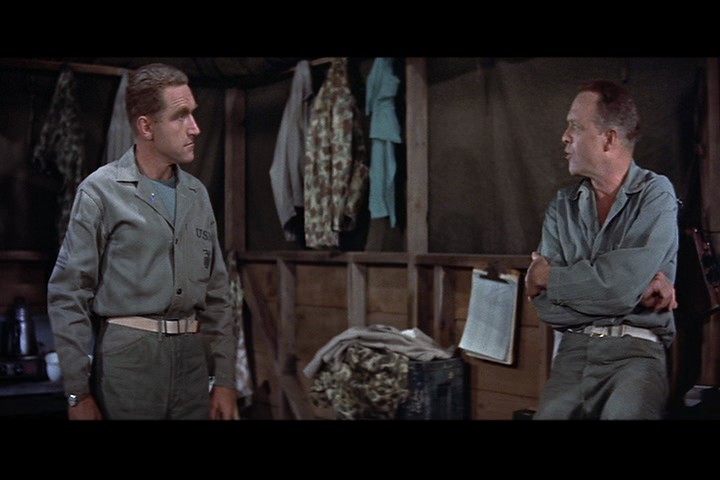

Supplement: S2 Dataset — (ZIP) [file pone.0264302.s002.zip › battle-cry-00137021.jpg]

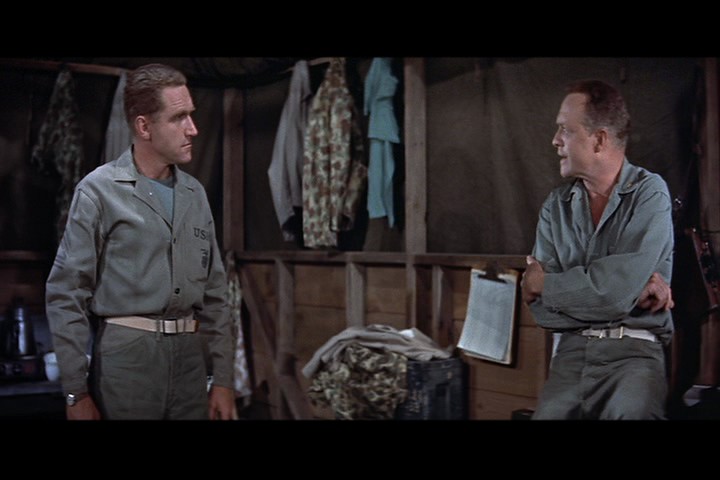

Supplement: S2 Dataset — (ZIP) [file pone.0264302.s002.zip › battle-cry-00137031.jpg]

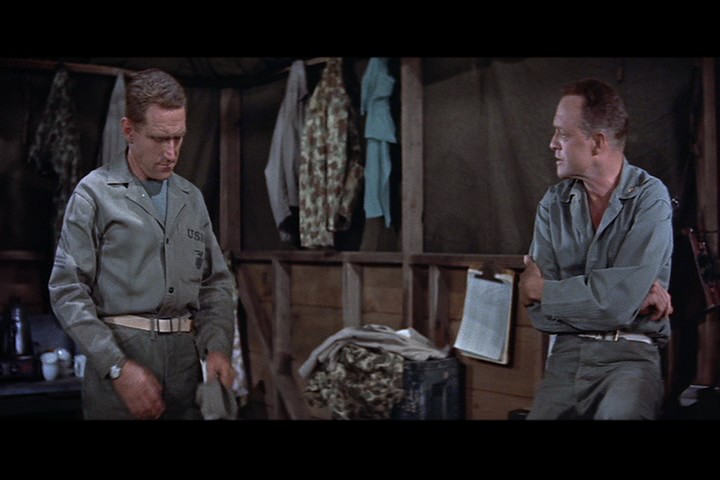

Supplement: S2 Dataset — (ZIP) [file pone.0264302.s002.zip › battle-cry-00137071.jpg]

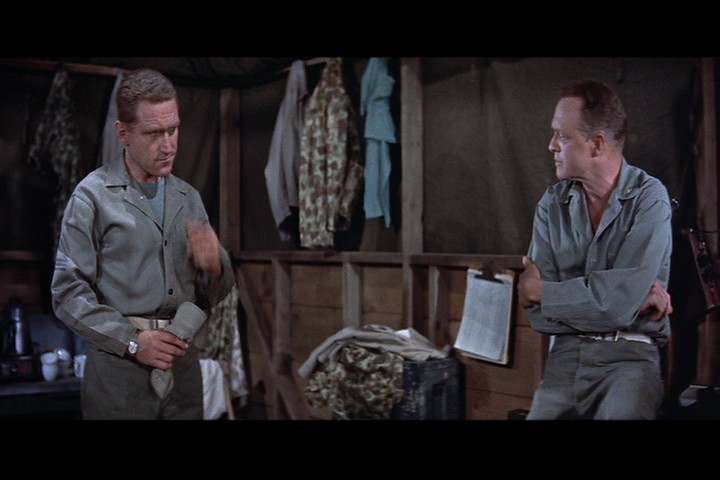

Supplement: S2 Dataset — (ZIP) [file pone.0264302.s002.zip › battle-cry-00137091.jpg]

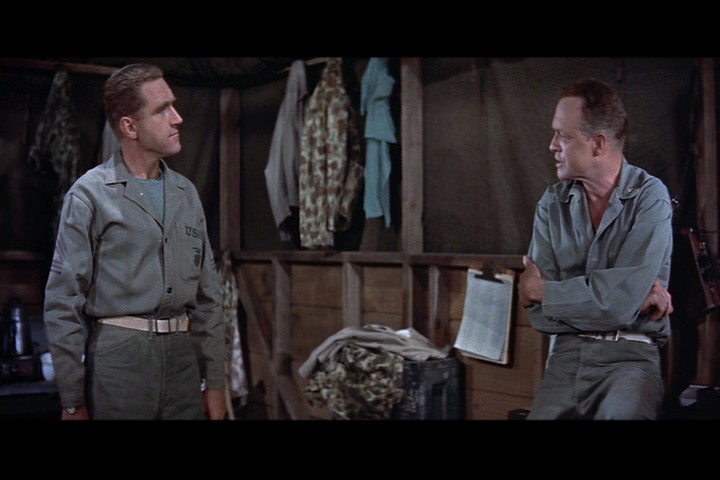

Supplement: S2 Dataset — (ZIP) [file pone.0264302.s002.zip › battle-cry-00137151.jpg]

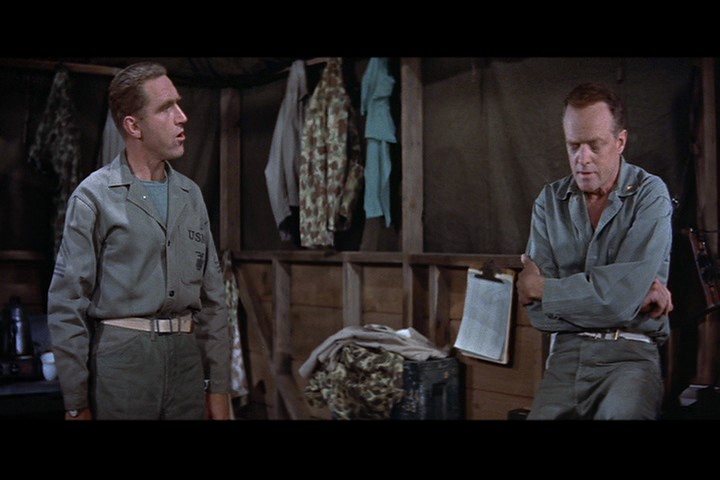

Supplement: S2 Dataset — (ZIP) [file pone.0264302.s002.zip › battle-cry-00137231.jpg]

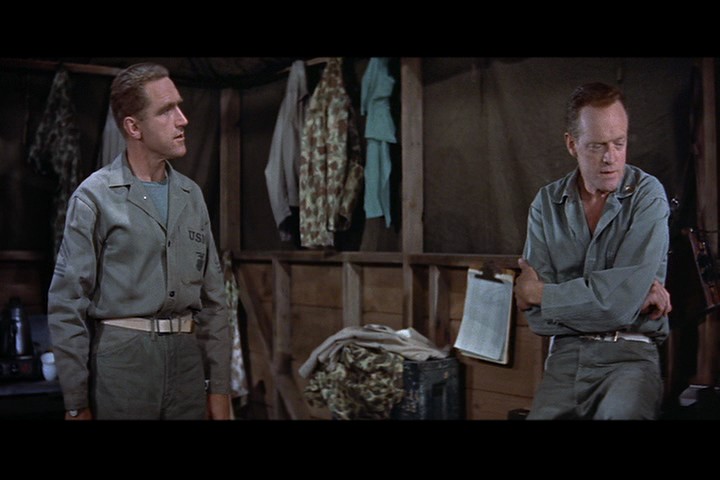

Supplement: S2 Dataset — (ZIP) [file pone.0264302.s002.zip › battle-cry-00137241.jpg]

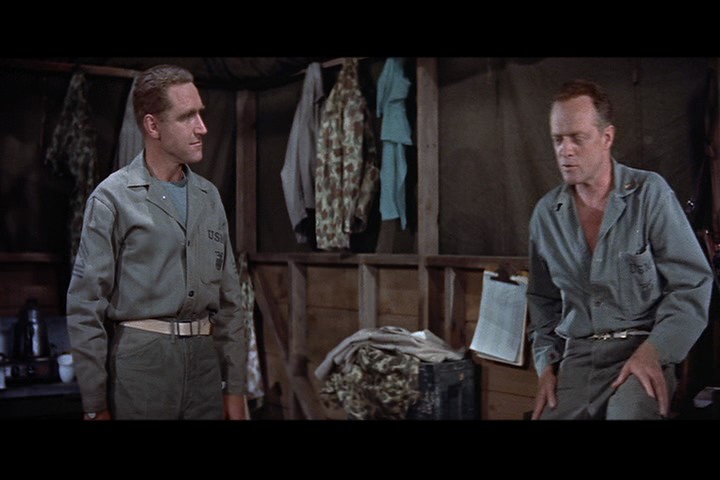

Supplement: S2 Dataset — (ZIP) [file pone.0264302.s002.zip › battle-cry-00137511.jpg]

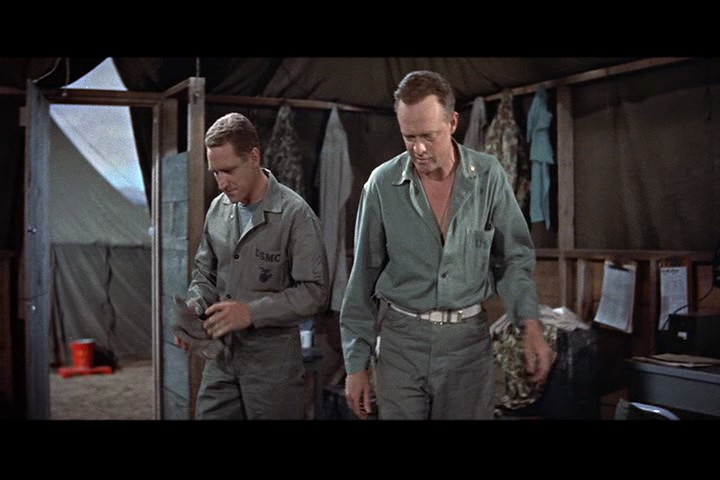

Supplement: S2 Dataset — (ZIP) [file pone.0264302.s002.zip › battle-cry-00137571.jpg]

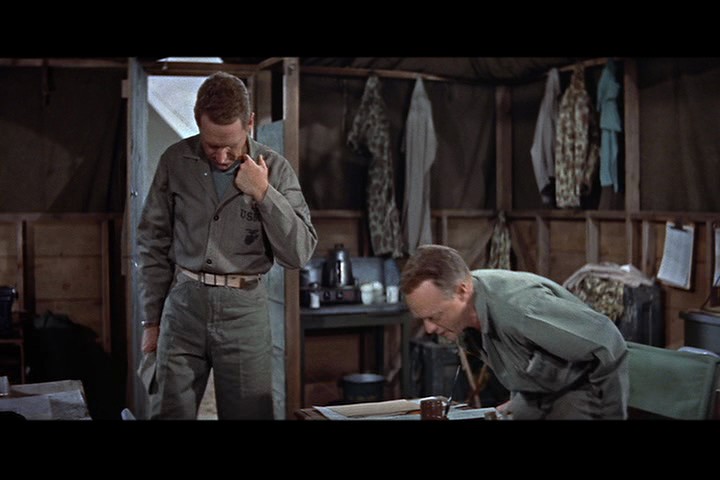

Supplement: S2 Dataset — (ZIP) [file pone.0264302.s002.zip › battle-cry-00137621.jpg]

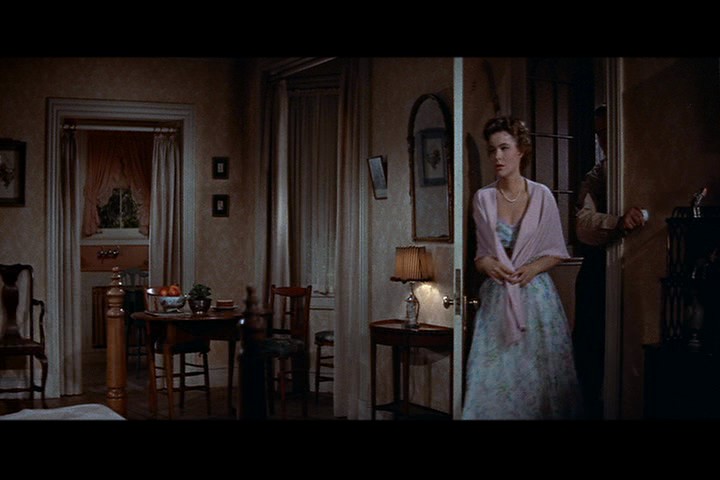

Supplement: S2 Dataset — (ZIP) [file pone.0264302.s002.zip › battle-cry-00144631.jpg]

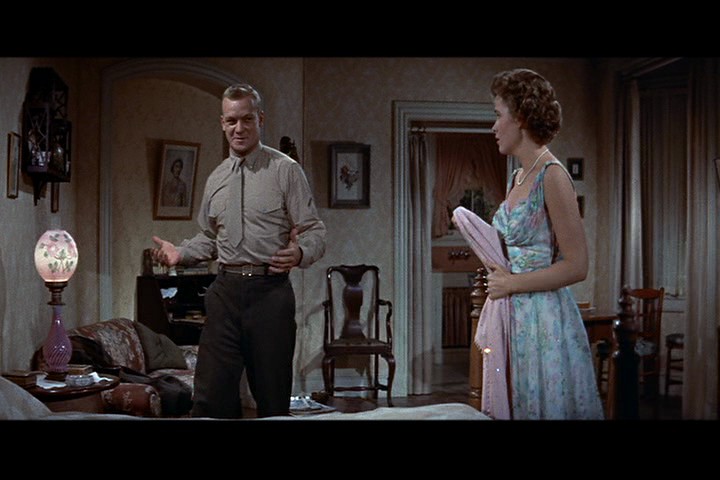

Supplement: S2 Dataset — (ZIP) [file pone.0264302.s002.zip › battle-cry-00144871.jpg]

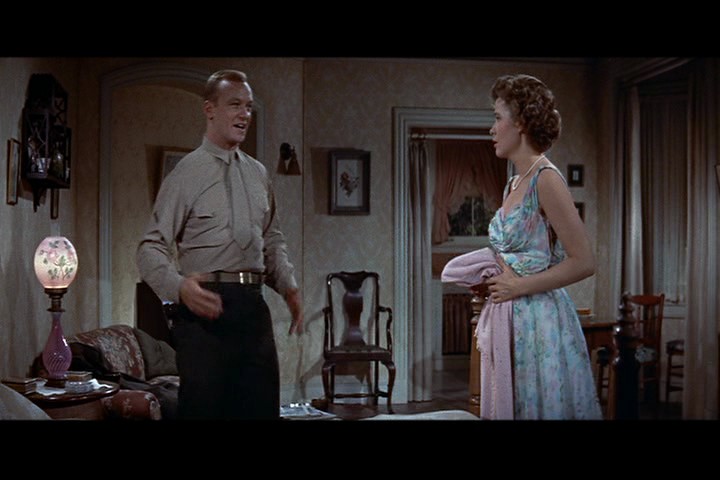

Supplement: S2 Dataset — (ZIP) [file pone.0264302.s002.zip › battle-cry-00144891.jpg]

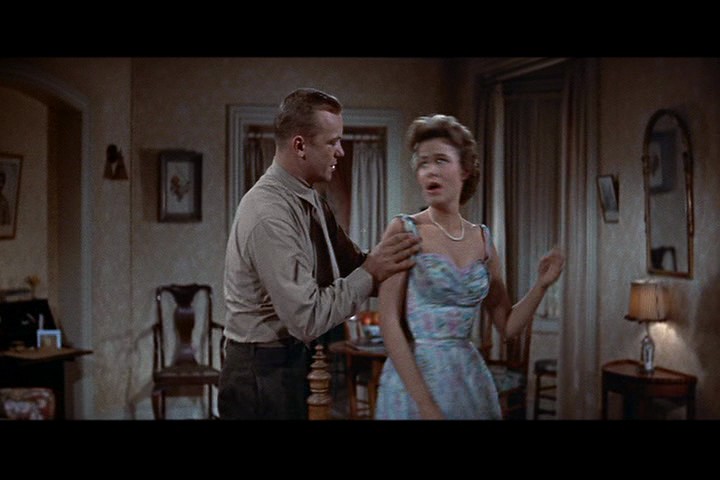

Supplement: S2 Dataset — (ZIP) [file pone.0264302.s002.zip › battle-cry-00145871.jpg]

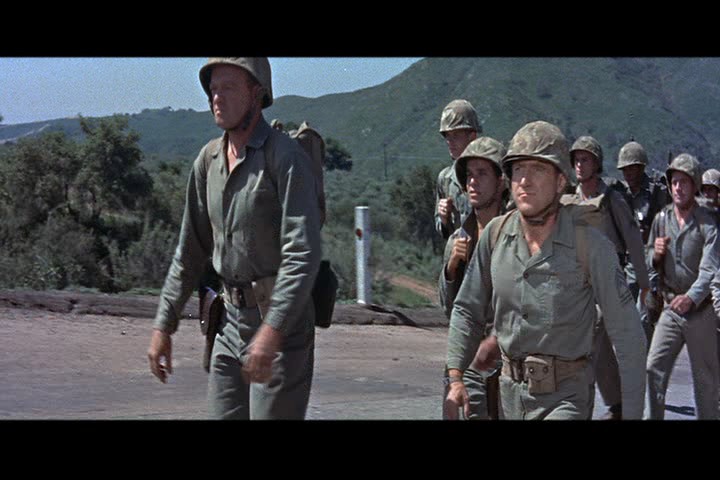

Supplement: S2 Dataset — (ZIP) [file pone.0264302.s002.zip › battle-cry-00149171.jpg]

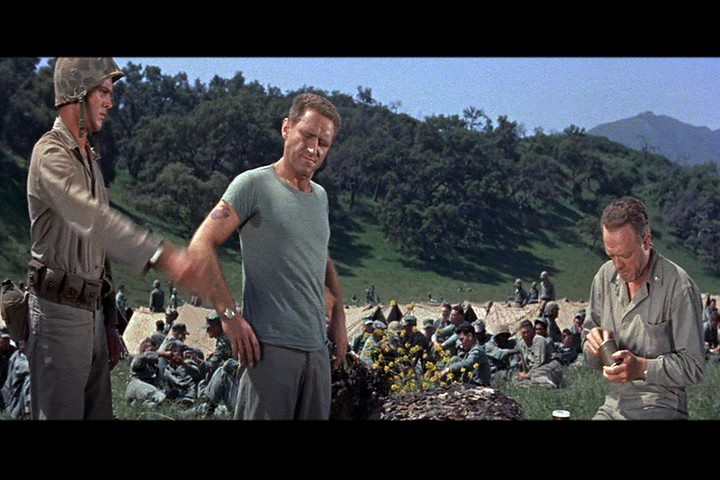

Supplement: S2 Dataset — (ZIP) [file pone.0264302.s002.zip › battle-cry-00152231.jpg]

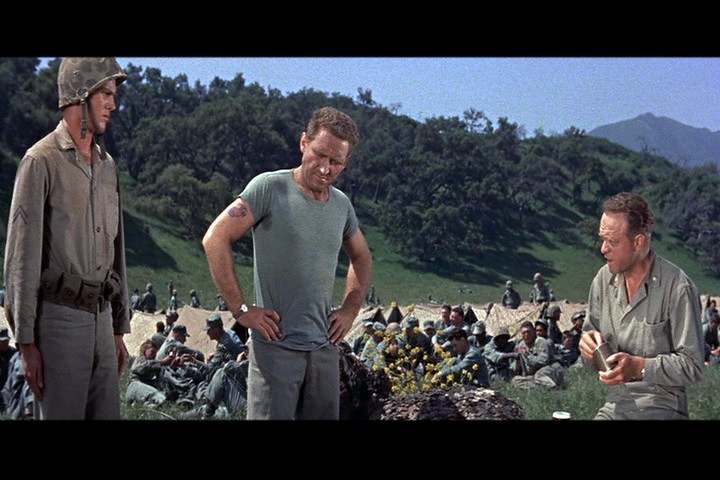

Supplement: S2 Dataset — (ZIP) [file pone.0264302.s002.zip › battle-cry-00152251.jpg]

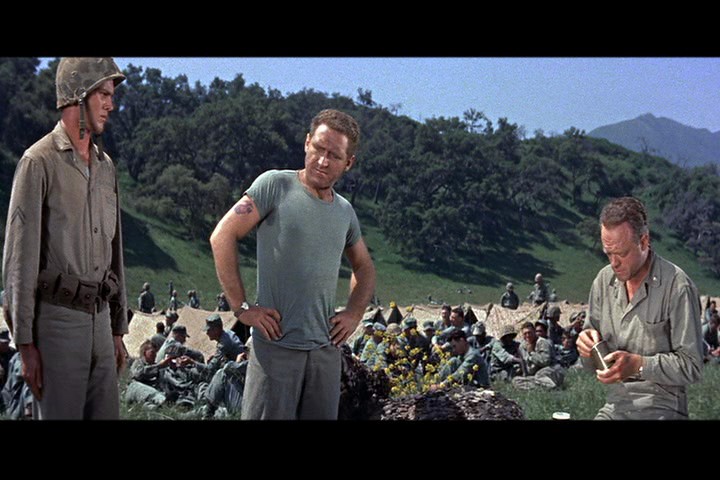

Supplement: S2 Dataset — (ZIP) [file pone.0264302.s002.zip › battle-cry-00152261.jpg]

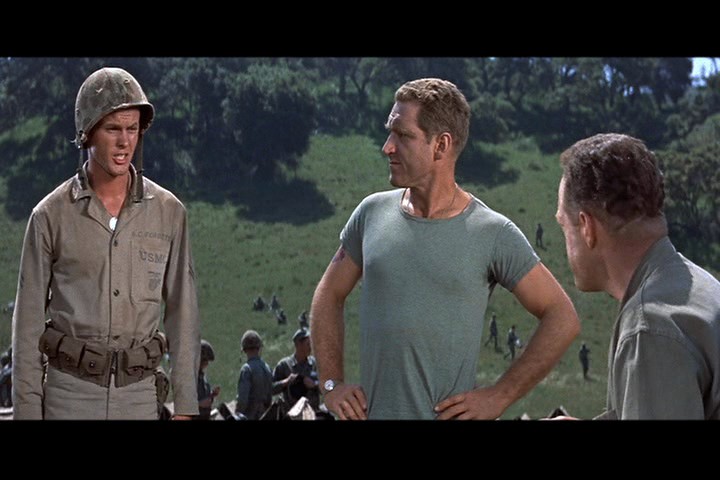

Supplement: S2 Dataset — (ZIP) [file pone.0264302.s002.zip › battle-cry-00152351.jpg]

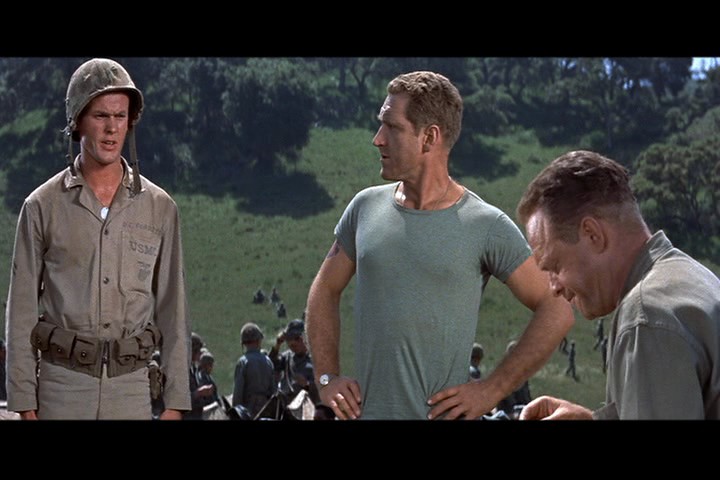

Supplement: S2 Dataset — (ZIP) [file pone.0264302.s002.zip › battle-cry-00152491.jpg]

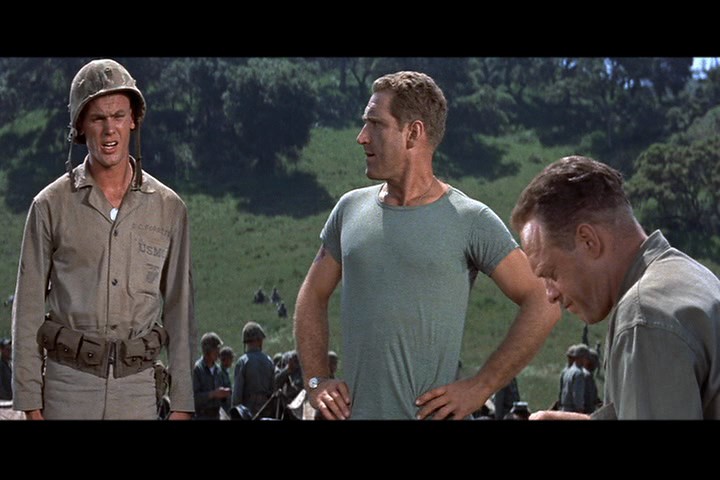

Supplement: S2 Dataset — (ZIP) [file pone.0264302.s002.zip › battle-cry-00152531.jpg]

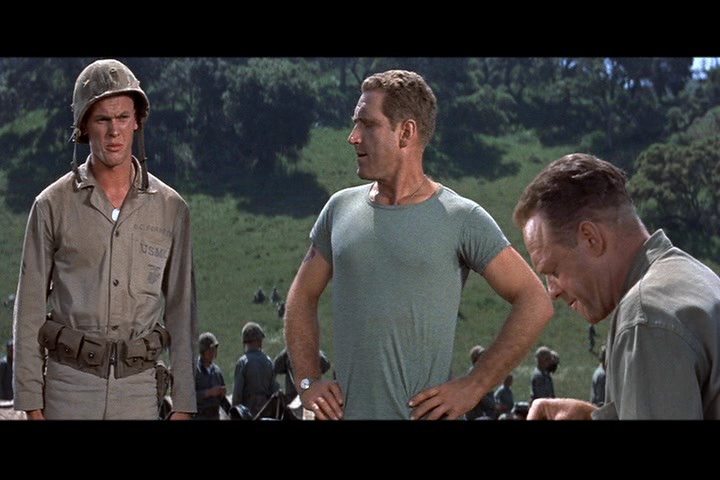

Supplement: S2 Dataset — (ZIP) [file pone.0264302.s002.zip › battle-cry-00152541.jpg]

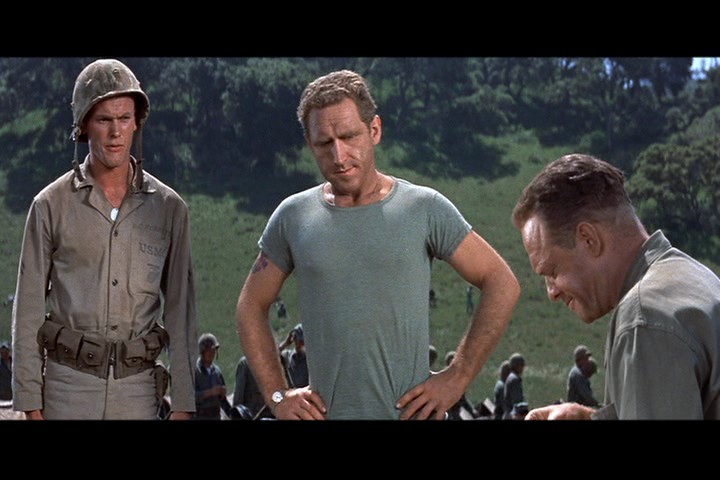

Supplement: S2 Dataset — (ZIP) [file pone.0264302.s002.zip › battle-cry-00152571.jpg]

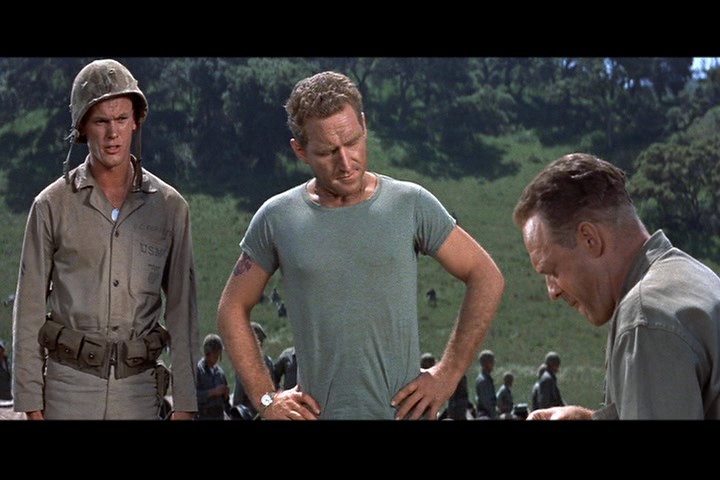

Supplement: S2 Dataset — (ZIP) [file pone.0264302.s002.zip › battle-cry-00152581.jpg]

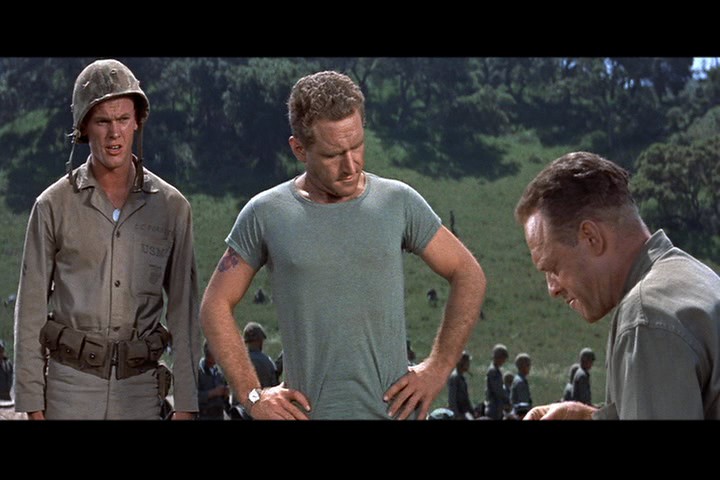

Supplement: S2 Dataset — (ZIP) [file pone.0264302.s002.zip › battle-cry-00152591.jpg]

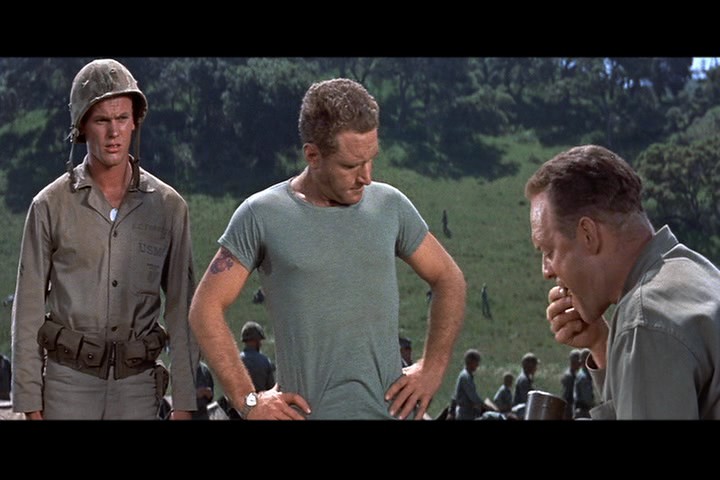

Supplement: S2 Dataset — (ZIP) [file pone.0264302.s002.zip › battle-cry-00152611.jpg]

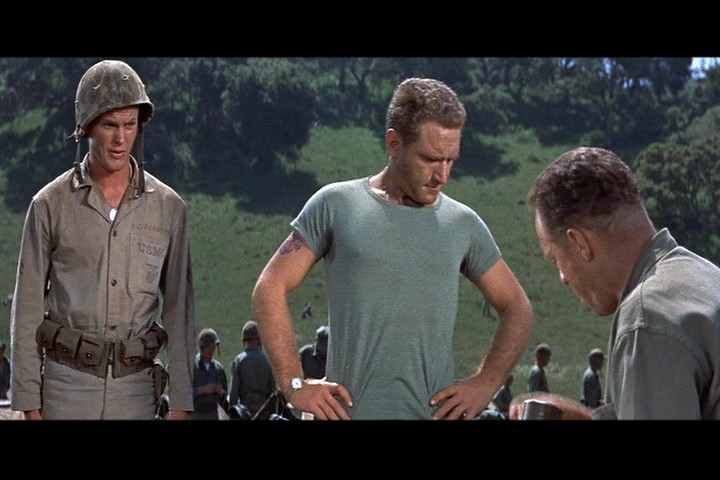

Supplement: S2 Dataset — (ZIP) [file pone.0264302.s002.zip › battle-cry-00152631.jpg]

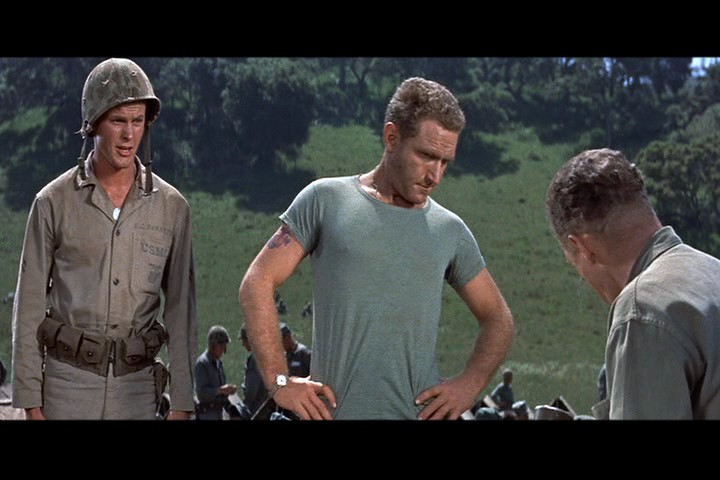

Supplement: S2 Dataset — (ZIP) [file pone.0264302.s002.zip › battle-cry-00152651.jpg]

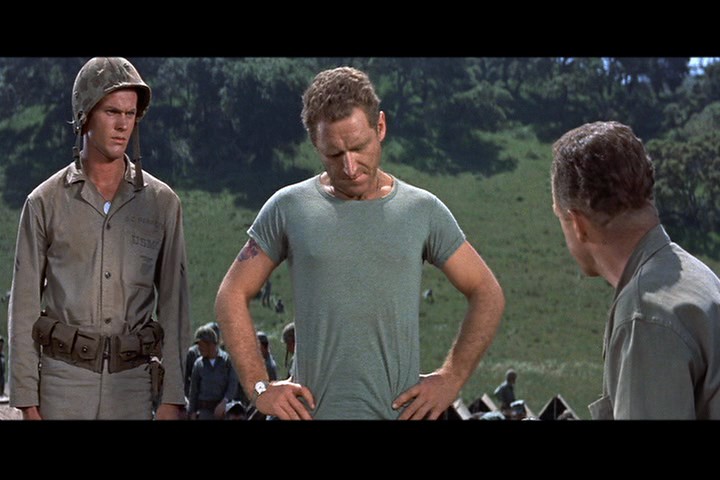

Supplement: S2 Dataset — (ZIP) [file pone.0264302.s002.zip › battle-cry-00152931.jpg]

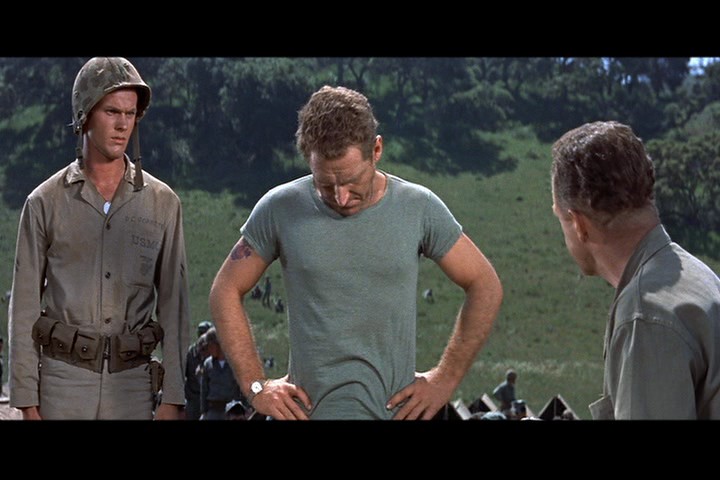

Supplement: S2 Dataset — (ZIP) [file pone.0264302.s002.zip › battle-cry-00152941.jpg]

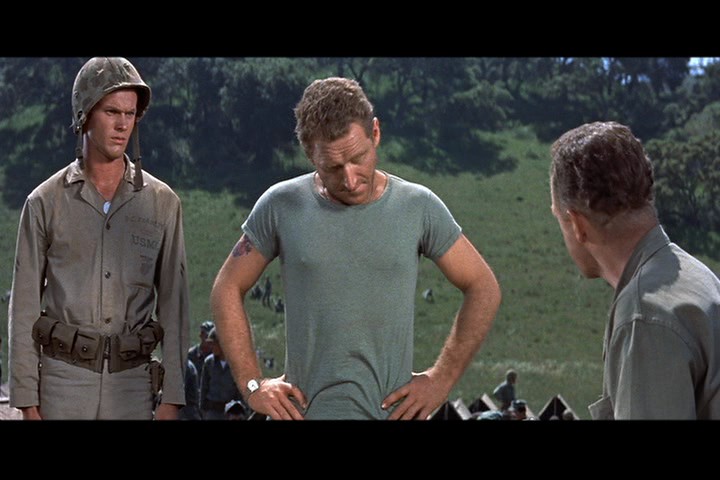

Supplement: S2 Dataset — (ZIP) [file pone.0264302.s002.zip › battle-cry-00152951.jpg]

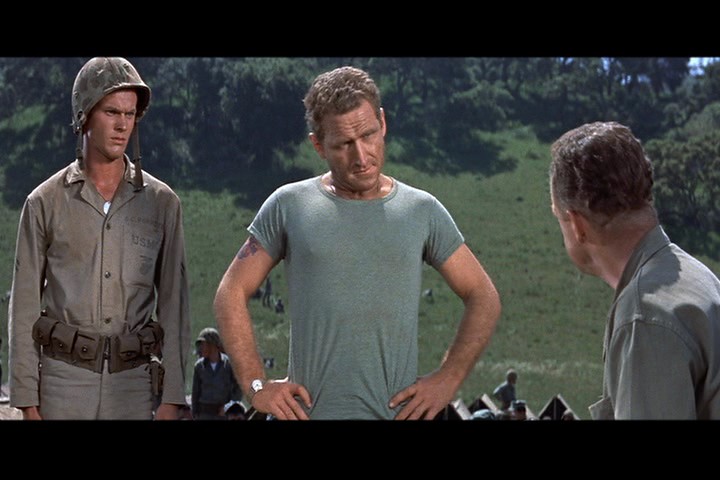

Supplement: S2 Dataset — (ZIP) [file pone.0264302.s002.zip › battle-cry-00152961.jpg]

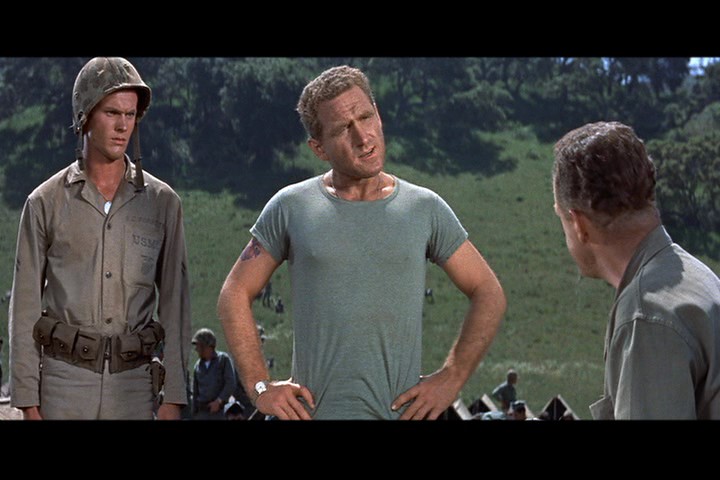

Supplement: S2 Dataset — (ZIP) [file pone.0264302.s002.zip › battle-cry-00152981.jpg]

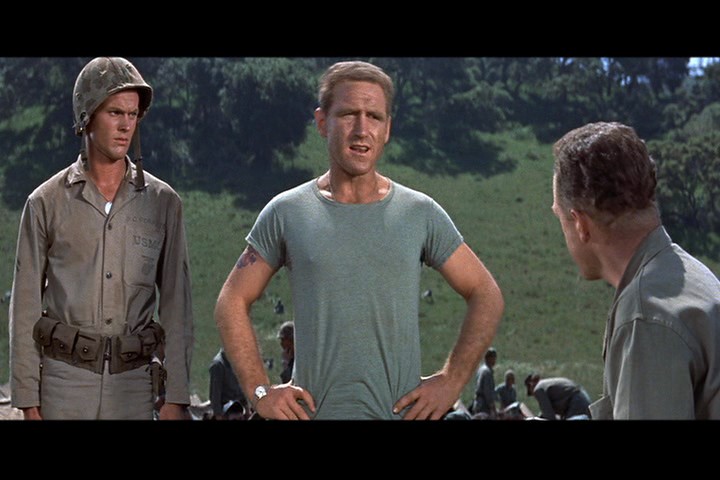

Supplement: S2 Dataset — (ZIP) [file pone.0264302.s002.zip › battle-cry-00153171.jpg]

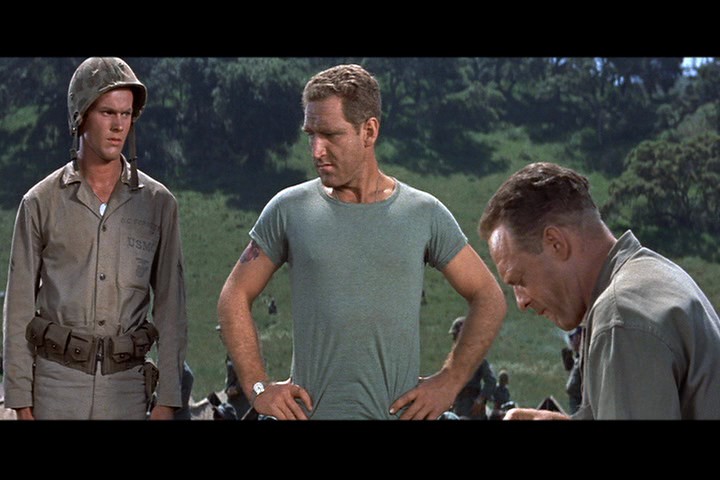

Supplement: S2 Dataset — (ZIP) [file pone.0264302.s002.zip › battle-cry-00153461.jpg]

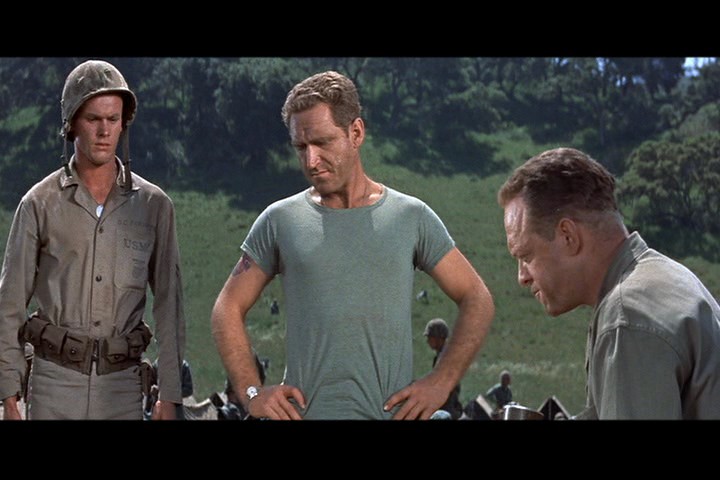

Supplement: S2 Dataset — (ZIP) [file pone.0264302.s002.zip › battle-cry-00153531.jpg]

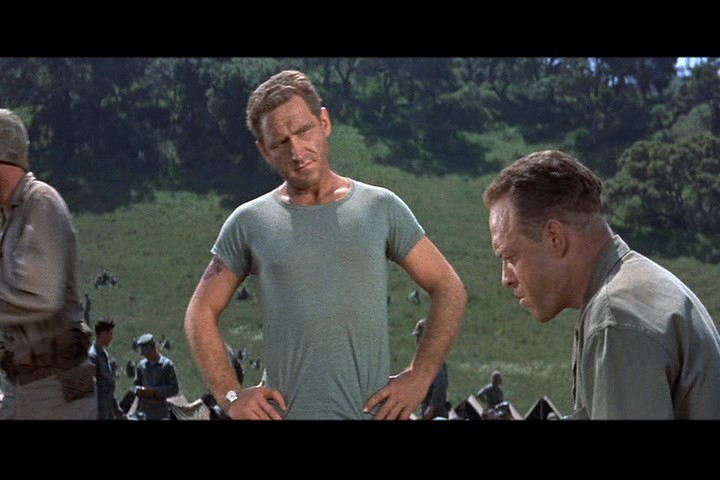

Supplement: S2 Dataset — (ZIP) [file pone.0264302.s002.zip › battle-cry-00153551.jpg]

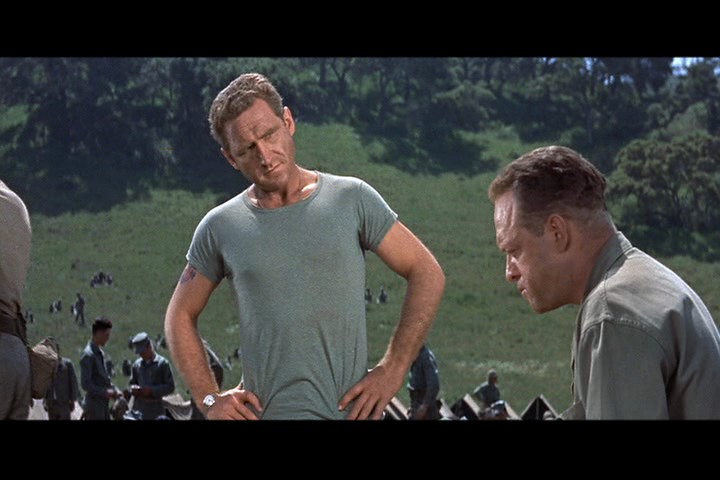

Supplement: S2 Dataset — (ZIP) [file pone.0264302.s002.zip › battle-cry-00153561.jpg]

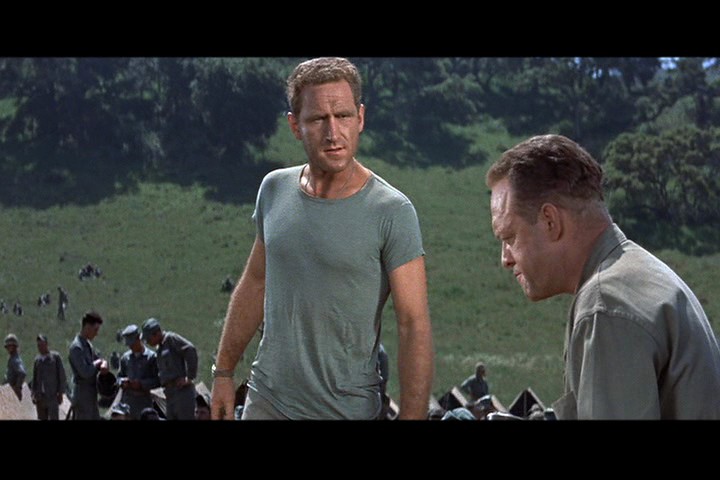

Supplement: S2 Dataset — (ZIP) [file pone.0264302.s002.zip › battle-cry-00153581.jpg]

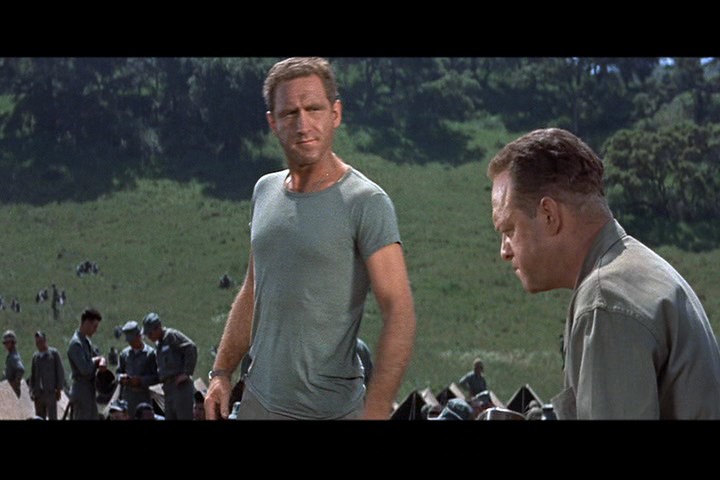

Supplement: S2 Dataset — (ZIP) [file pone.0264302.s002.zip › battle-cry-00153591.jpg]

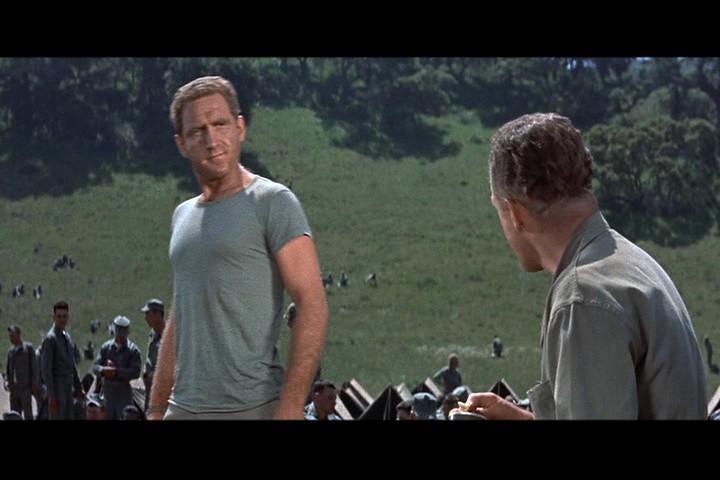

Supplement: S2 Dataset — (ZIP) [file pone.0264302.s002.zip › battle-cry-00153781.jpg]

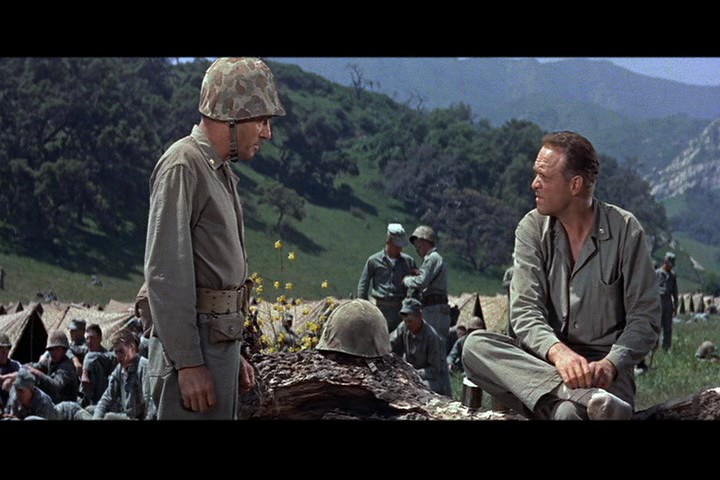

Supplement: S2 Dataset — (ZIP) [file pone.0264302.s002.zip › battle-cry-00154131.jpg]

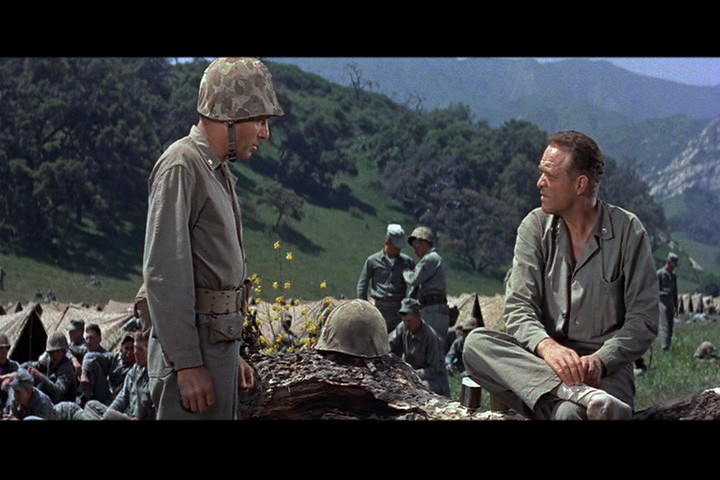

Supplement: S2 Dataset — (ZIP) [file pone.0264302.s002.zip › battle-cry-00154141.jpg]

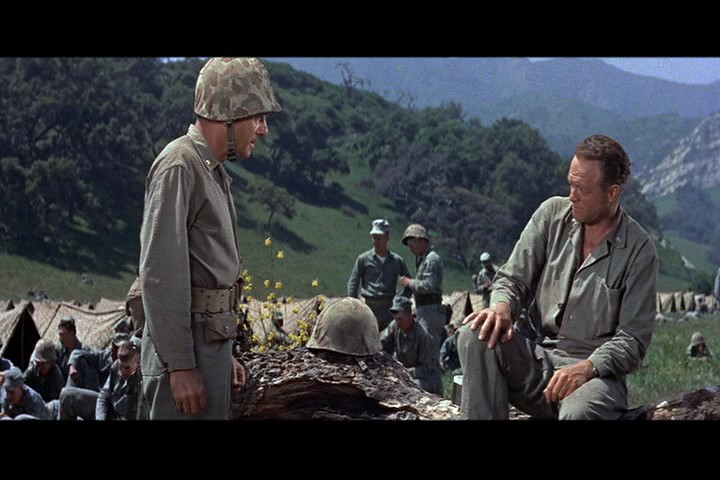

Supplement: S2 Dataset — (ZIP) [file pone.0264302.s002.zip › battle-cry-00154231.jpg]

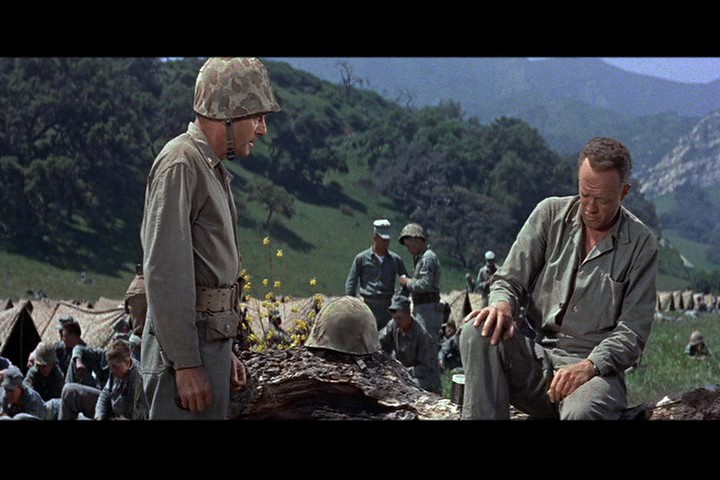

Supplement: S2 Dataset — (ZIP) [file pone.0264302.s002.zip › battle-cry-00154241.jpg]

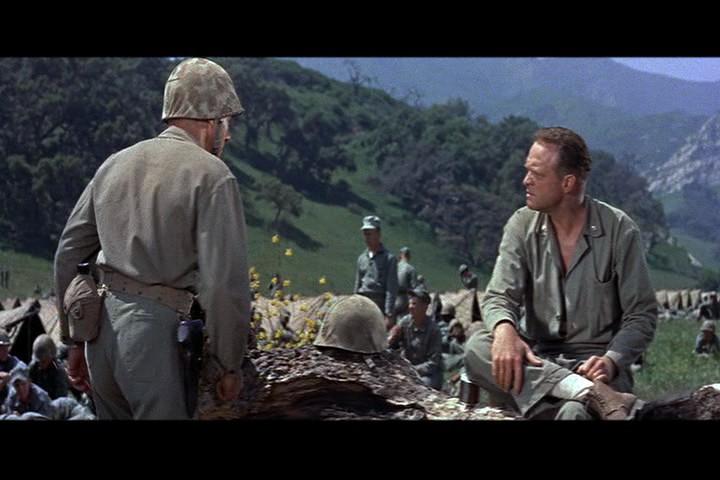

Supplement: S2 Dataset — (ZIP) [file pone.0264302.s002.zip › battle-cry-00155511.jpg]

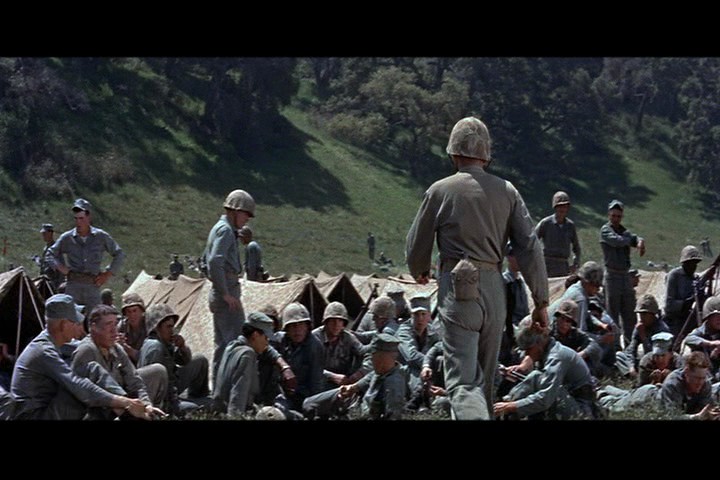

Supplement: S2 Dataset — (ZIP) [file pone.0264302.s002.zip › battle-cry-00155561.jpg]

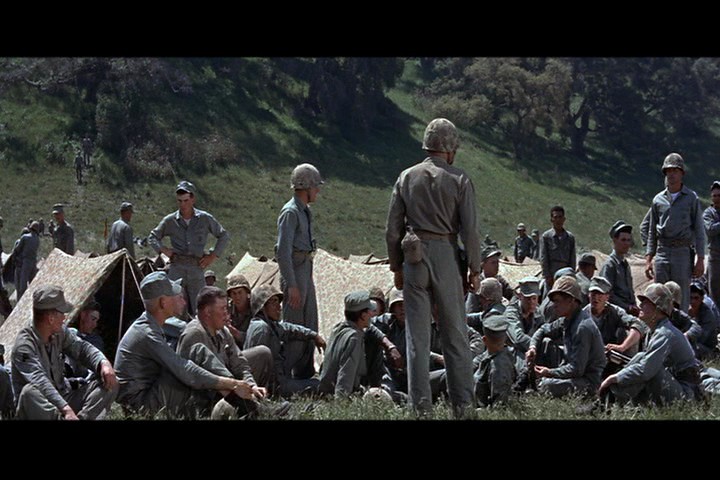

Supplement: S2 Dataset — (ZIP) [file pone.0264302.s002.zip › battle-cry-00155621.jpg]

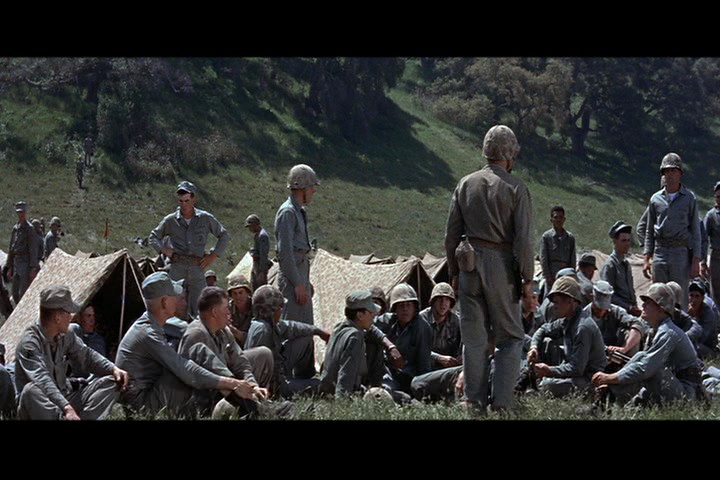

Supplement: S2 Dataset — (ZIP) [file pone.0264302.s002.zip › battle-cry-00155651.jpg]

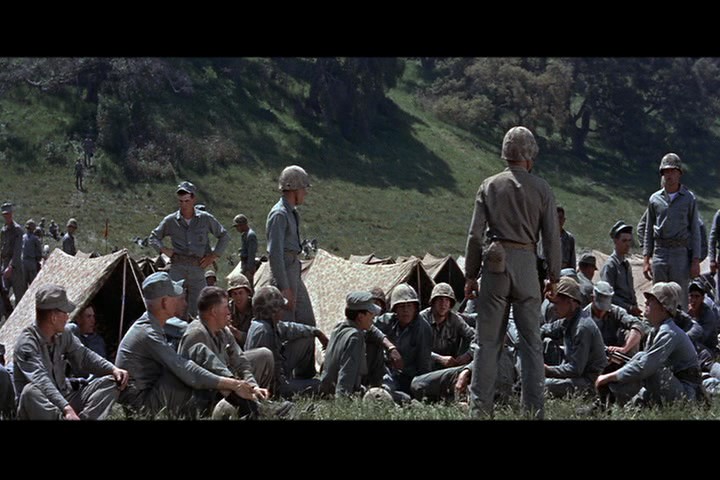

Supplement: S2 Dataset — (ZIP) [file pone.0264302.s002.zip › battle-cry-00155661.jpg]

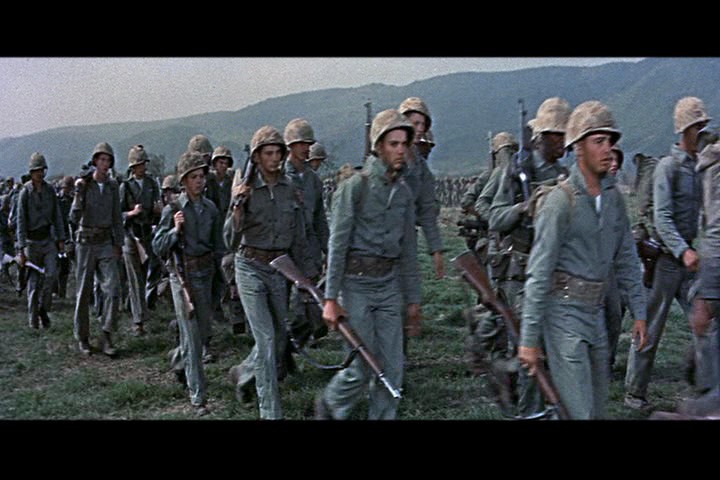

Supplement: S2 Dataset — (ZIP) [file pone.0264302.s002.zip › battle-cry-00156121.jpg]

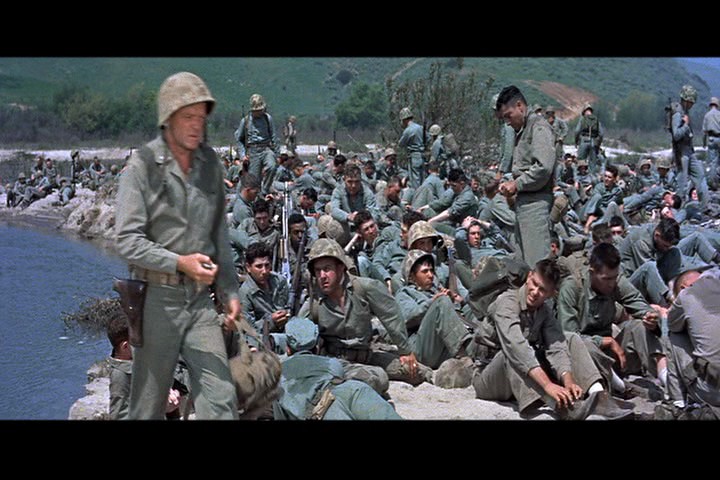

Supplement: S2 Dataset — (ZIP) [file pone.0264302.s002.zip › battle-cry-00156721.jpg]
