# Supplementary figures and images for: LHPE-nets: A lightweight 2D and 3D human pose estimation model with well-structural deep networks and multi-view pose sample simplification method (part 6 of 8)
Source: PLoS One. 2022 Feb 23;17(2):e0264302. doi: 10.1371/journal.pone.0264302 (PMC8865690; doi:10.1371/journal.pone.0264302)

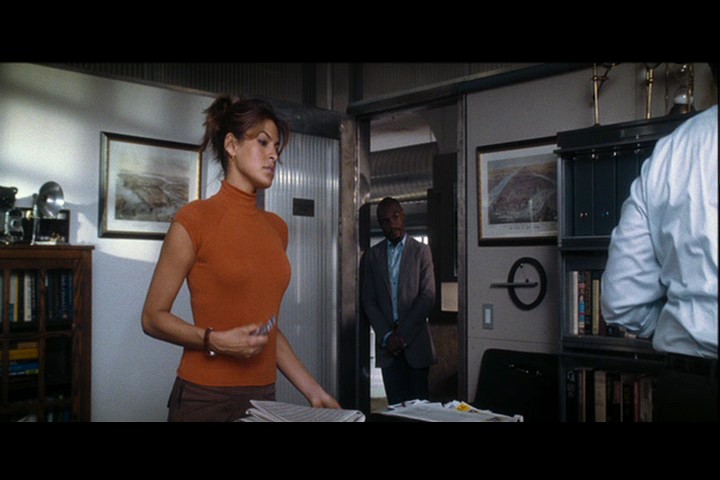

Supplement: S3 Dataset — (ZIP) [file pone.0264302.s003.zip › hitch-00119211.jpg]

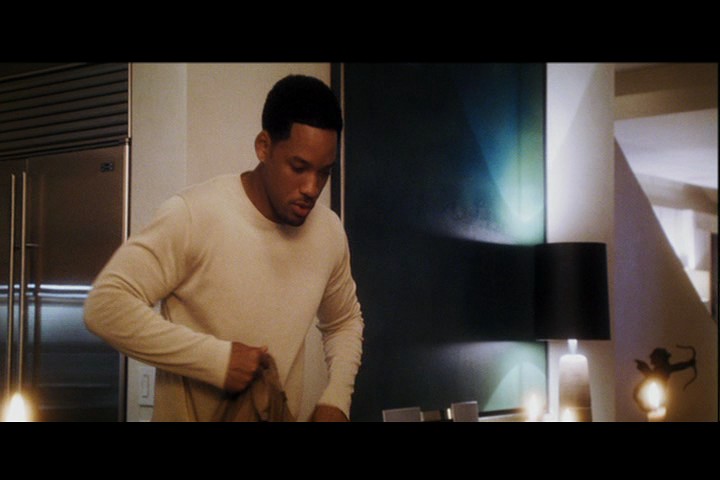

Supplement: S3 Dataset — (ZIP) [file pone.0264302.s003.zip › hitch-00120771.jpg]

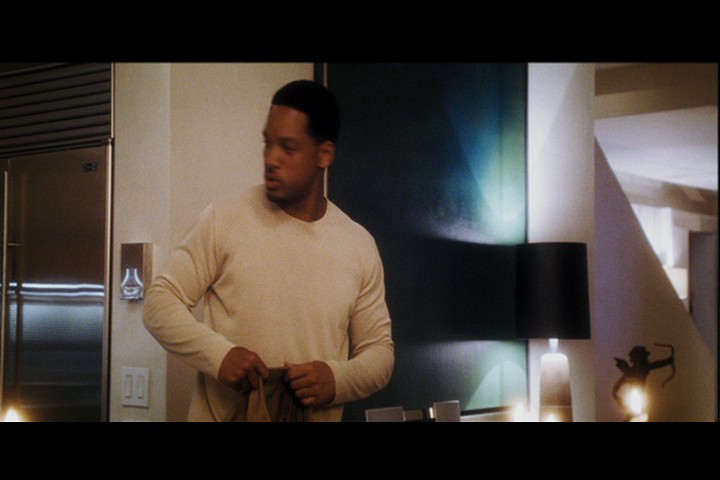

Supplement: S3 Dataset — (ZIP) [file pone.0264302.s003.zip › hitch-00120781.jpg]

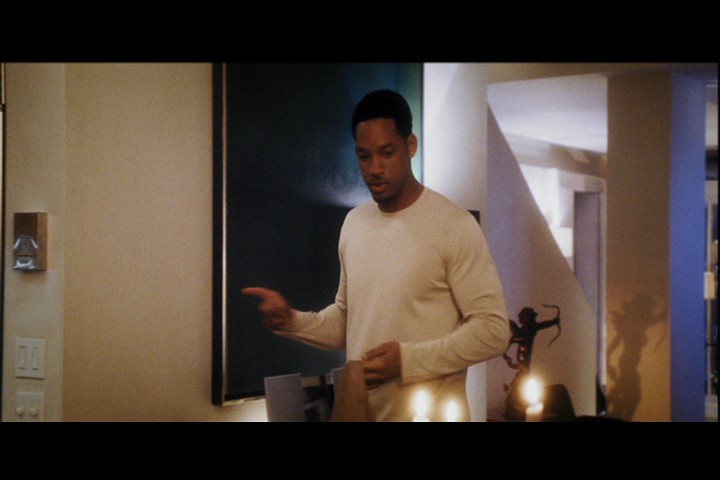

Supplement: S3 Dataset — (ZIP) [file pone.0264302.s003.zip › hitch-00120821.jpg]

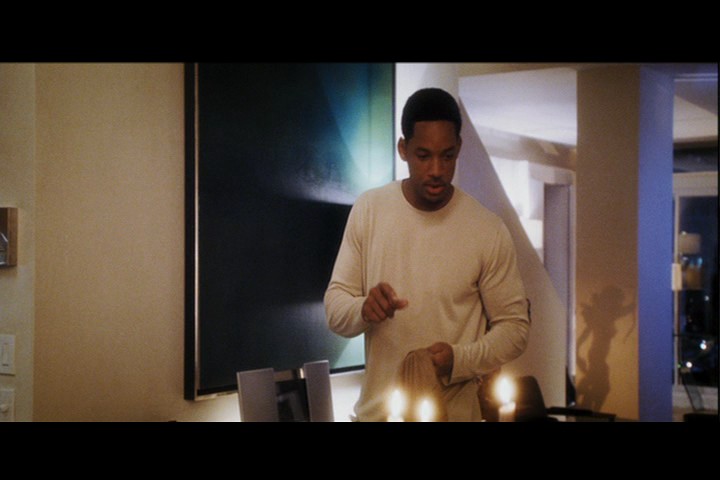

Supplement: S3 Dataset — (ZIP) [file pone.0264302.s003.zip › hitch-00120831.jpg]

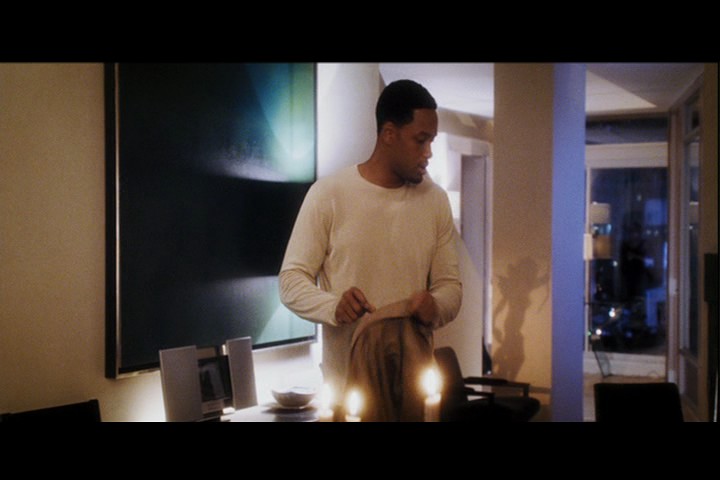

Supplement: S3 Dataset — (ZIP) [file pone.0264302.s003.zip › hitch-00120861.jpg]

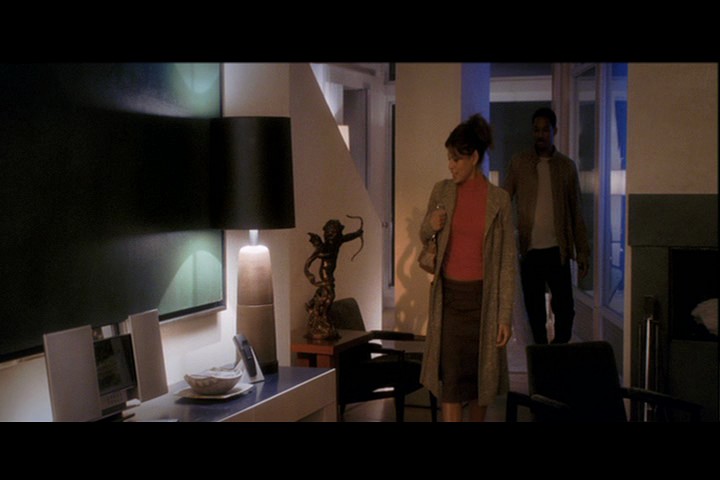

Supplement: S3 Dataset — (ZIP) [file pone.0264302.s003.zip › hitch-00121411.jpg]

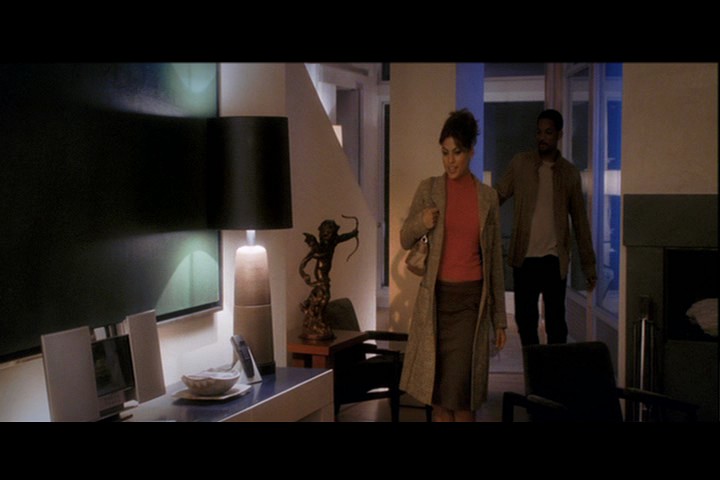

Supplement: S3 Dataset — (ZIP) [file pone.0264302.s003.zip › hitch-00121421.jpg]

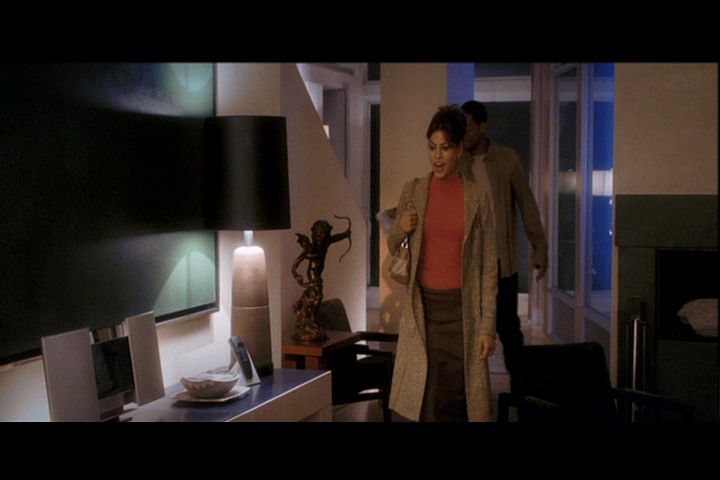

Supplement: S3 Dataset — (ZIP) [file pone.0264302.s003.zip › hitch-00121441.jpg]

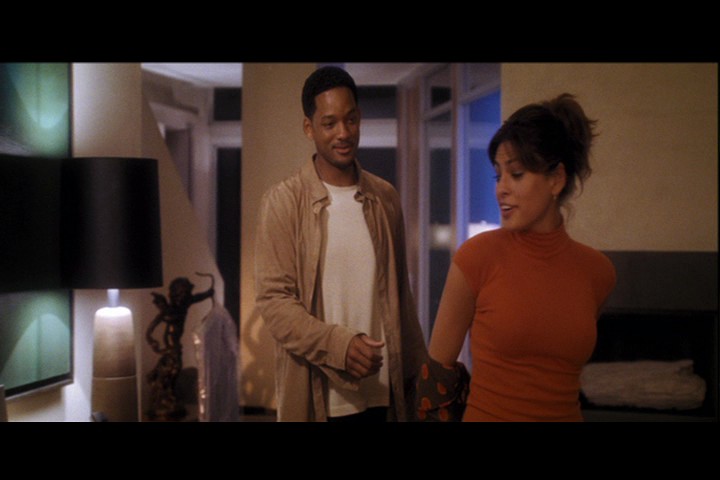

Supplement: S3 Dataset — (ZIP) [file pone.0264302.s003.zip › hitch-00121531.jpg]

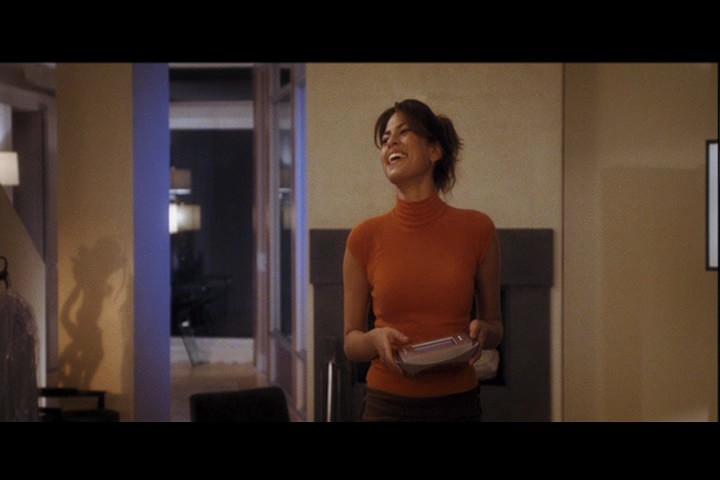

Supplement: S3 Dataset — (ZIP) [file pone.0264302.s003.zip › hitch-00124061.jpg]

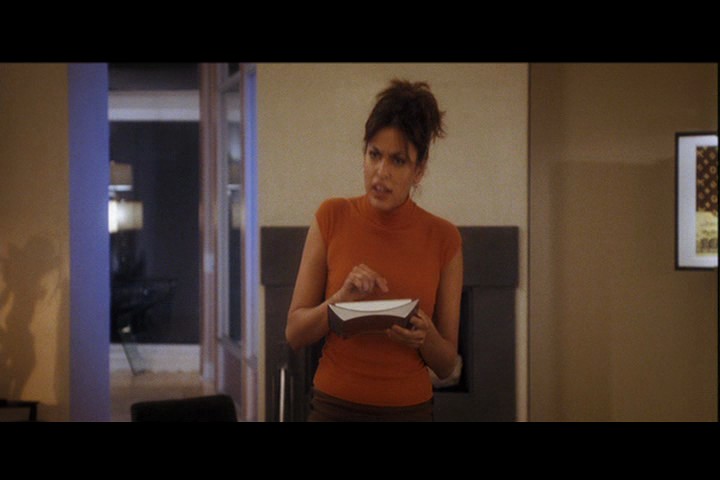

Supplement: S3 Dataset — (ZIP) [file pone.0264302.s003.zip › hitch-00124141.jpg]

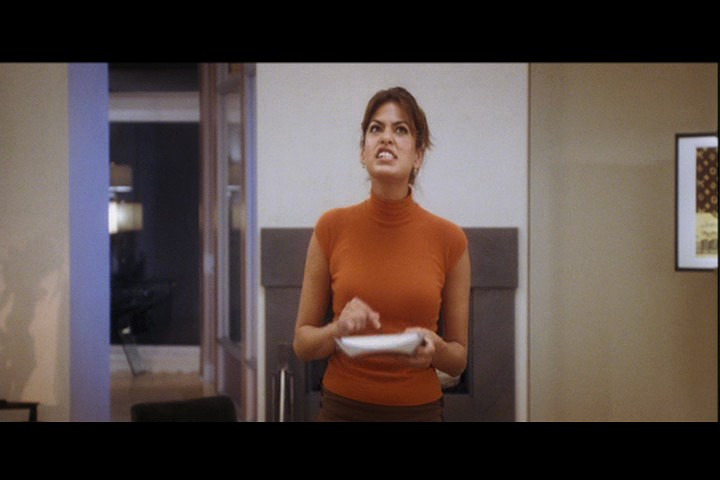

Supplement: S3 Dataset — (ZIP) [file pone.0264302.s003.zip › hitch-00124161.jpg]

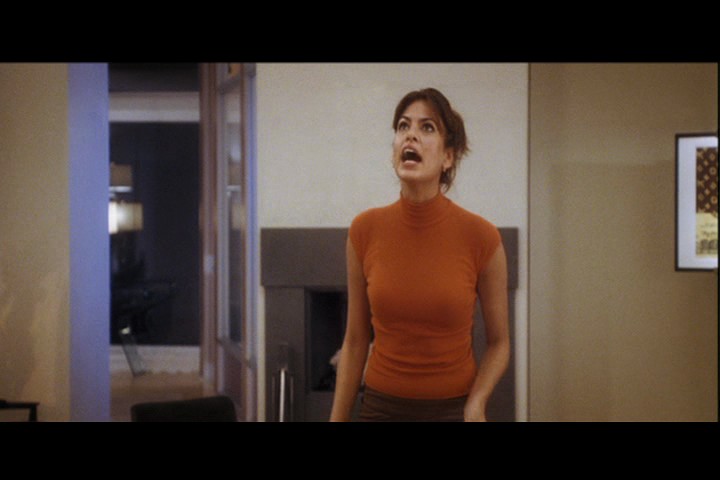

Supplement: S3 Dataset — (ZIP) [file pone.0264302.s003.zip › hitch-00124171.jpg]

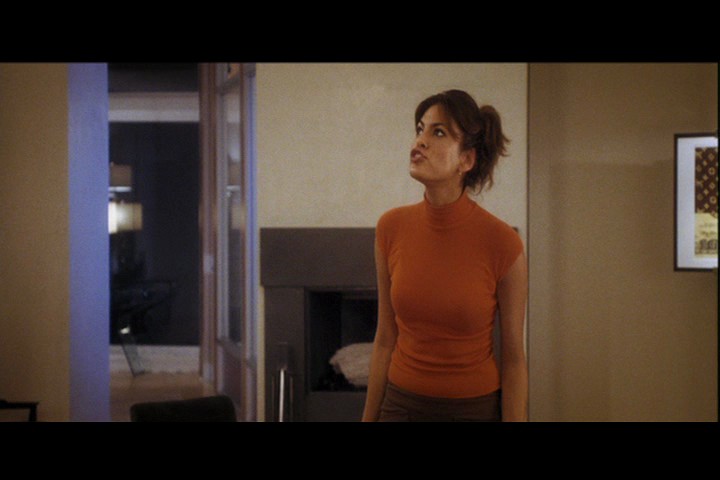

Supplement: S3 Dataset — (ZIP) [file pone.0264302.s003.zip › hitch-00124181.jpg]

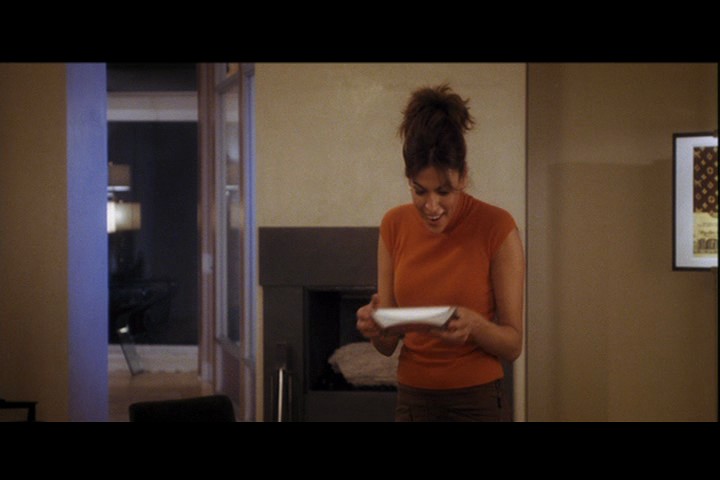

Supplement: S3 Dataset — (ZIP) [file pone.0264302.s003.zip › hitch-00124221.jpg]

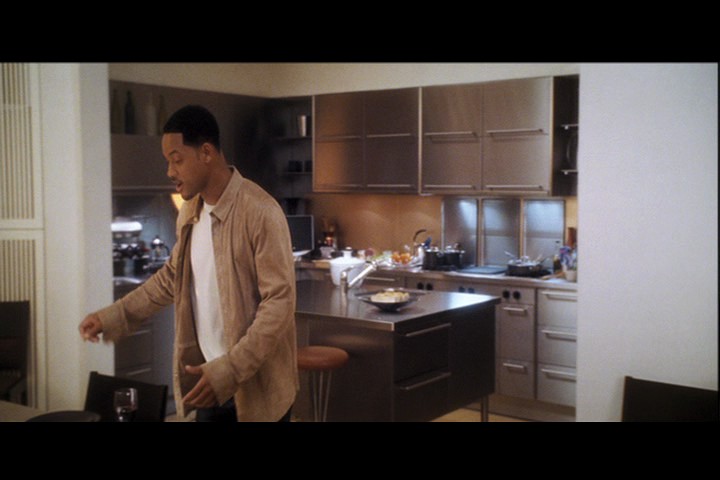

Supplement: S3 Dataset — (ZIP) [file pone.0264302.s003.zip › hitch-00124601.jpg]

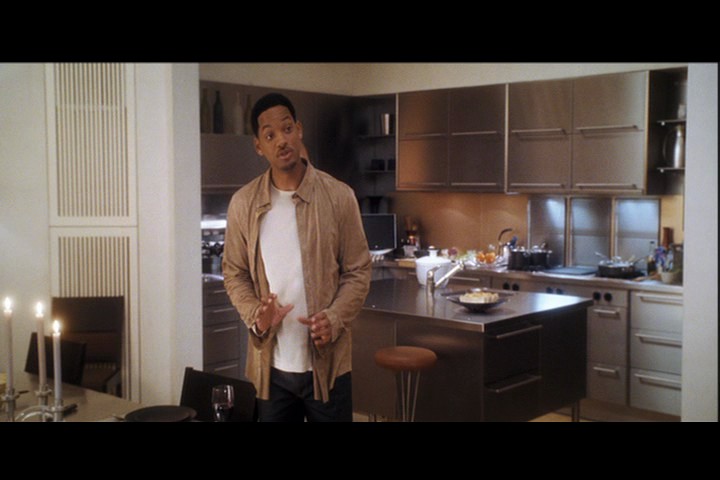

Supplement: S3 Dataset — (ZIP) [file pone.0264302.s003.zip › hitch-00124651.jpg]

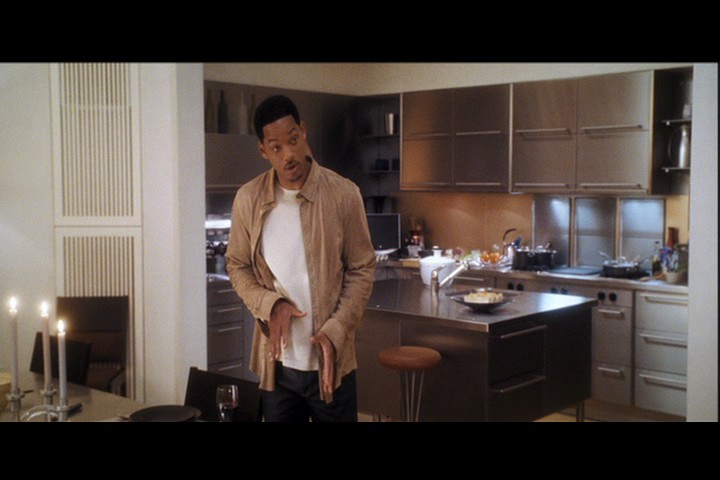

Supplement: S3 Dataset — (ZIP) [file pone.0264302.s003.zip › hitch-00124661.jpg]

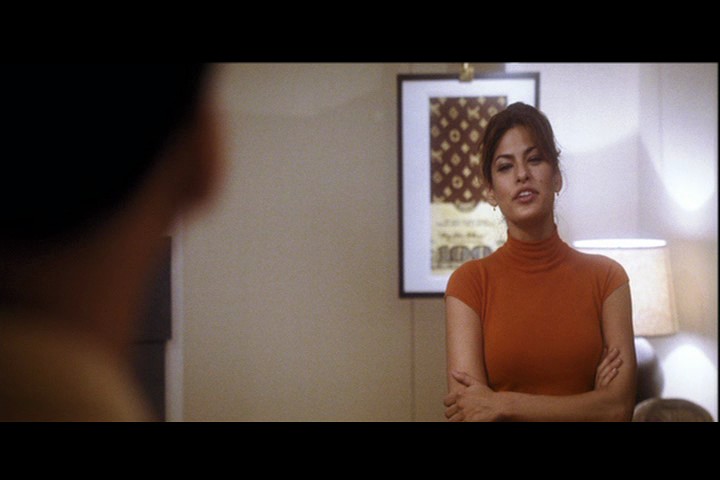

Supplement: S3 Dataset — (ZIP) [file pone.0264302.s003.zip › hitch-00124721.jpg]

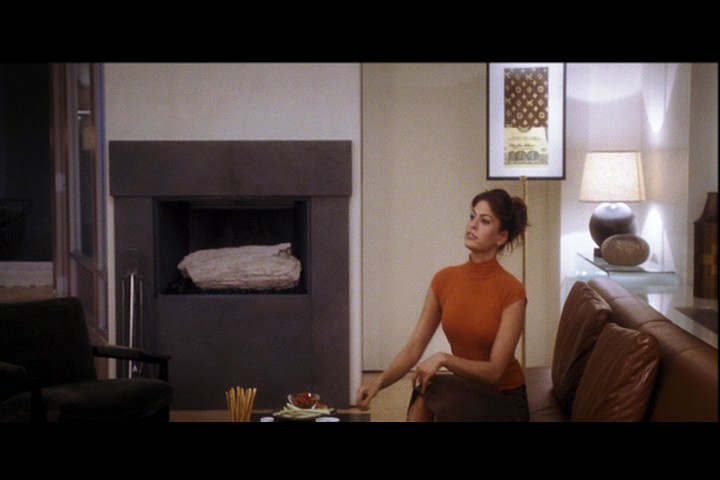

Supplement: S3 Dataset — (ZIP) [file pone.0264302.s003.zip › hitch-00125121.jpg]

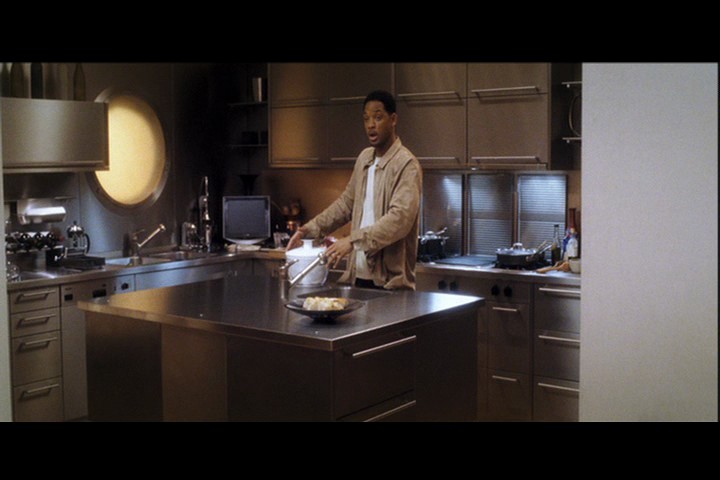

Supplement: S3 Dataset — (ZIP) [file pone.0264302.s003.zip › hitch-00125501.jpg]

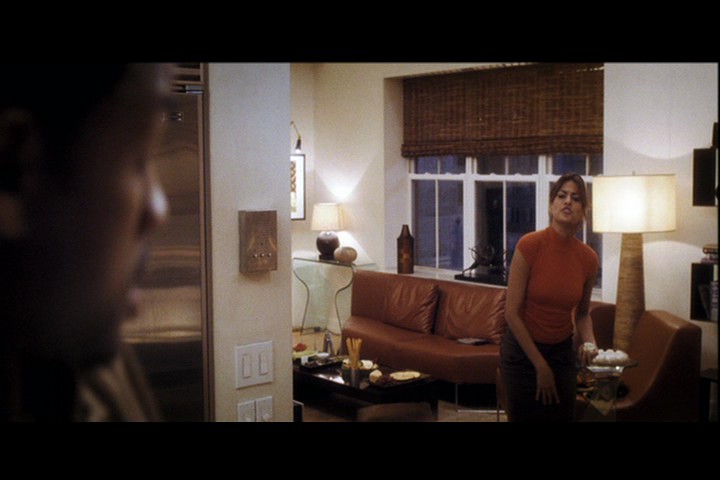

Supplement: S3 Dataset — (ZIP) [file pone.0264302.s003.zip › hitch-00125541.jpg]

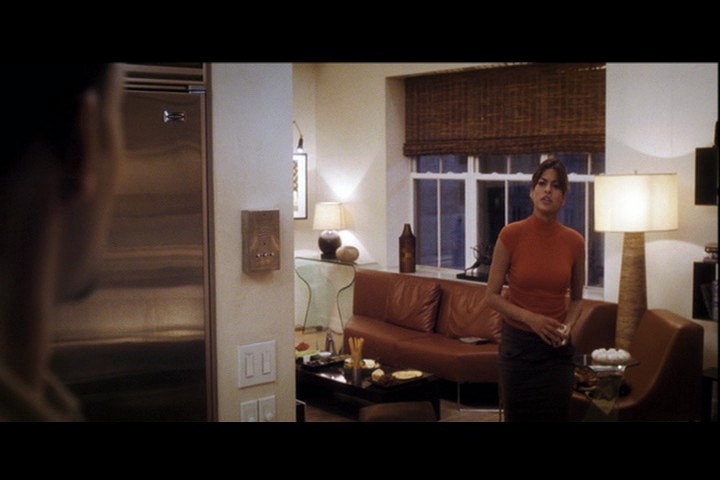

Supplement: S3 Dataset — (ZIP) [file pone.0264302.s003.zip › hitch-00125551.jpg]

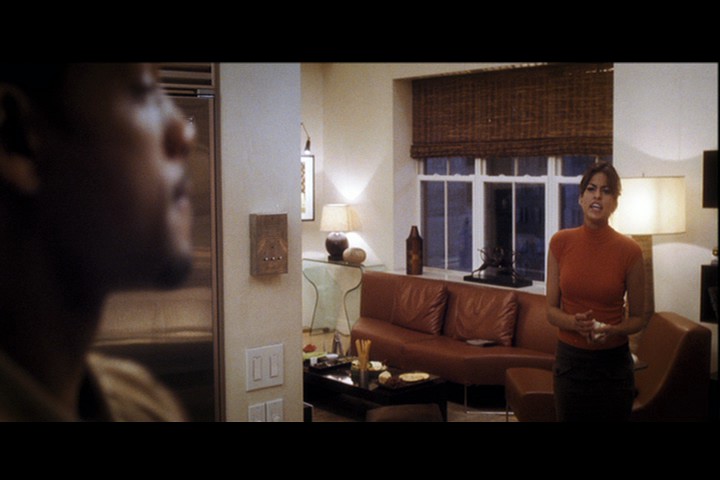

Supplement: S3 Dataset — (ZIP) [file pone.0264302.s003.zip › hitch-00125571.jpg]

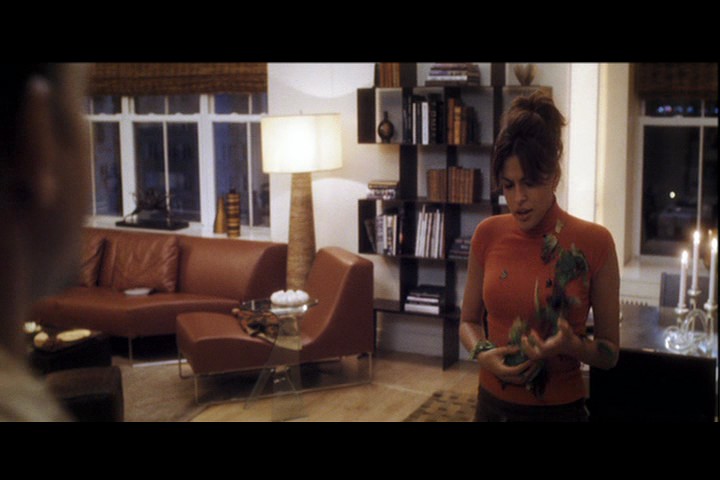

Supplement: S3 Dataset — (ZIP) [file pone.0264302.s003.zip › hitch-00126291.jpg]

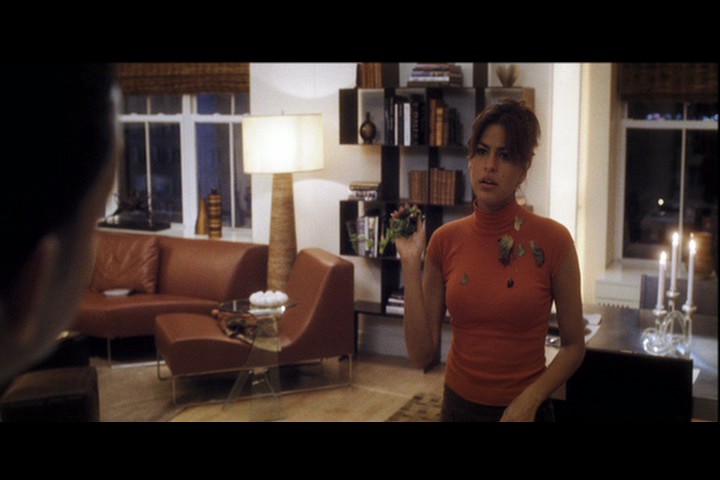

Supplement: S3 Dataset — (ZIP) [file pone.0264302.s003.zip › hitch-00126381.jpg]

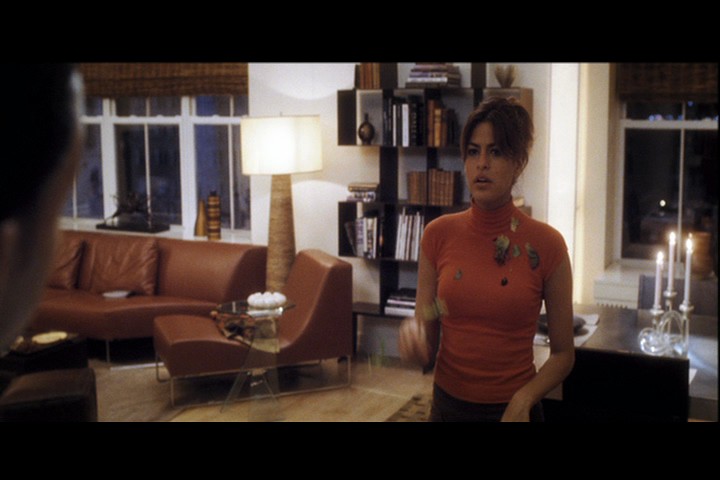

Supplement: S3 Dataset — (ZIP) [file pone.0264302.s003.zip › hitch-00126391.jpg]

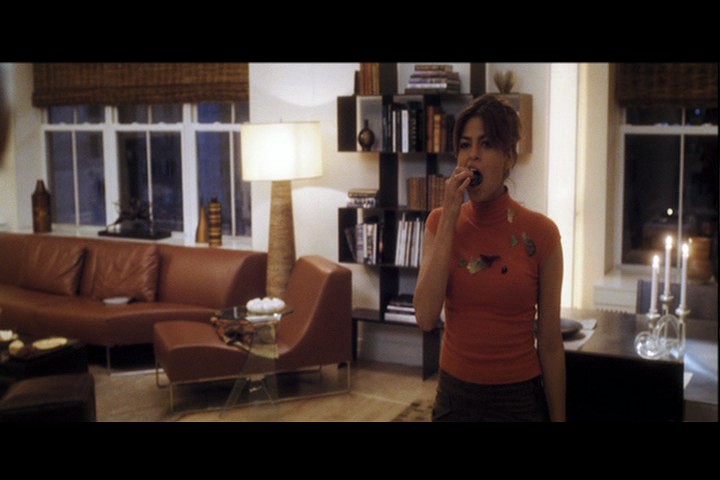

Supplement: S3 Dataset — (ZIP) [file pone.0264302.s003.zip › hitch-00126411.jpg]

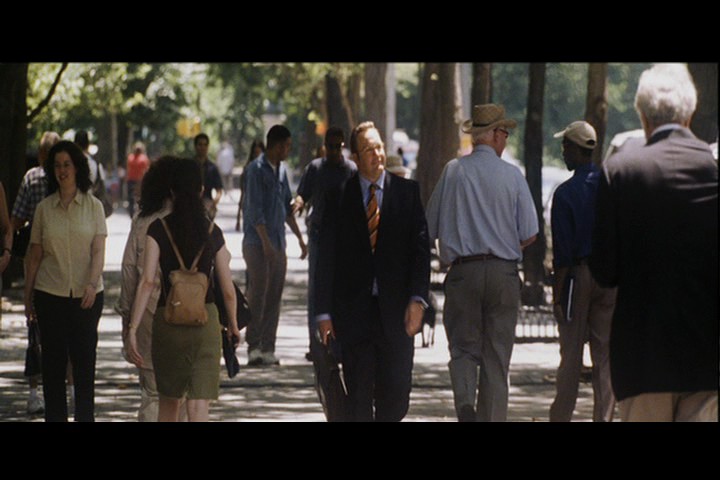

Supplement: S3 Dataset — (ZIP) [file pone.0264302.s003.zip › hitch-00126861.jpg]

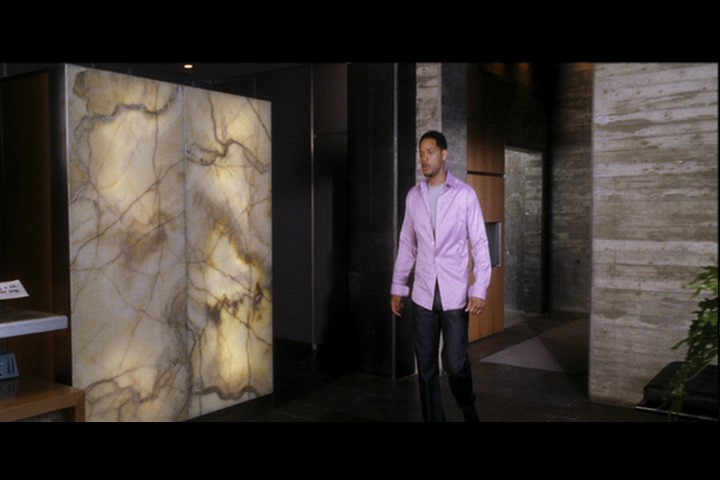

Supplement: S3 Dataset — (ZIP) [file pone.0264302.s003.zip › hitch-00126881.jpg]

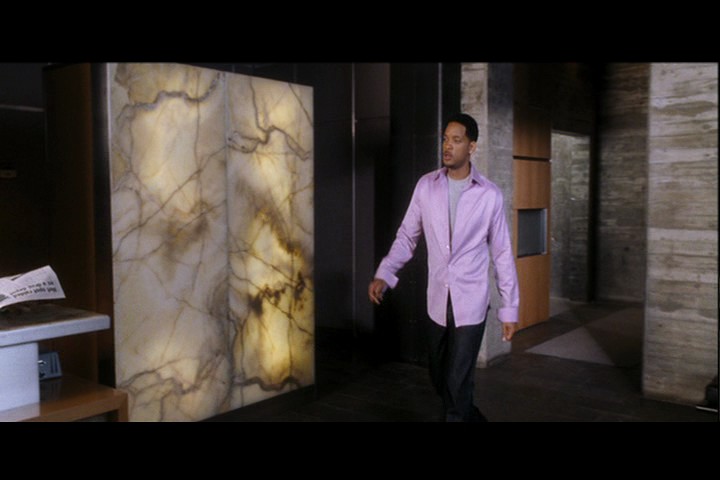

Supplement: S3 Dataset — (ZIP) [file pone.0264302.s003.zip › hitch-00126891.jpg]

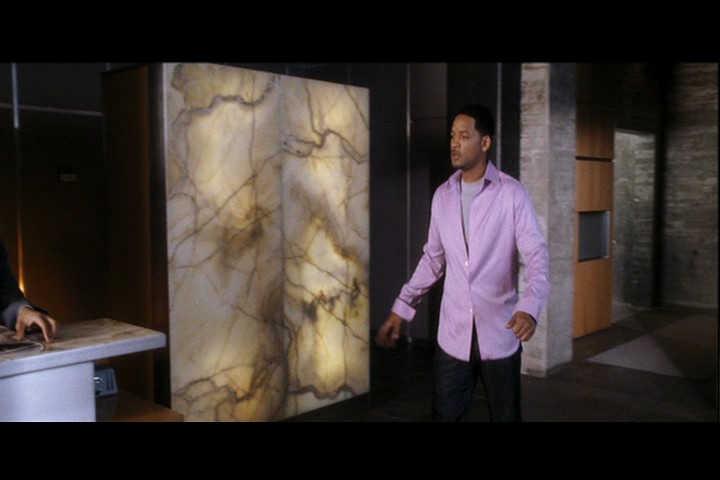

Supplement: S3 Dataset — (ZIP) [file pone.0264302.s003.zip › hitch-00126901.jpg]

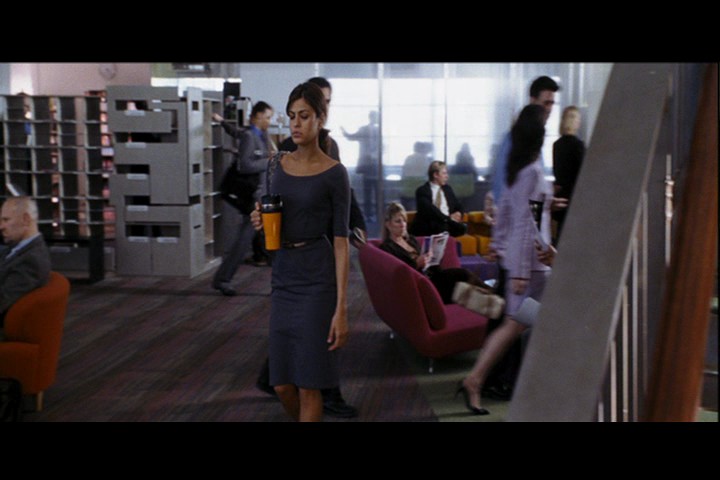

Supplement: S3 Dataset — (ZIP) [file pone.0264302.s003.zip › hitch-00127991.jpg]

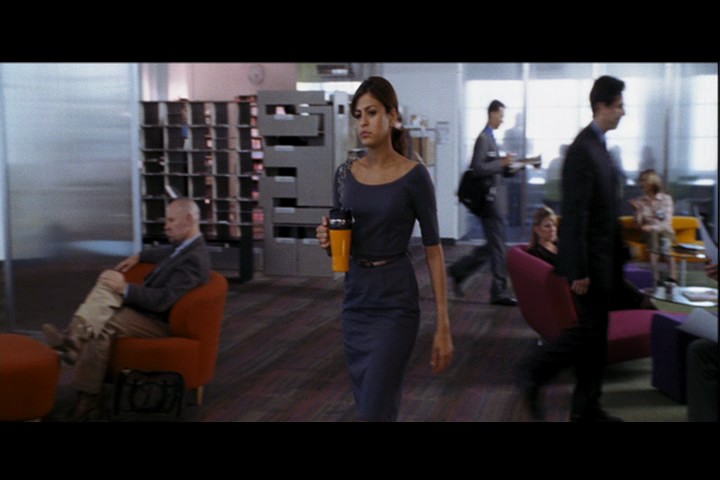

Supplement: S3 Dataset — (ZIP) [file pone.0264302.s003.zip › hitch-00128011.jpg]

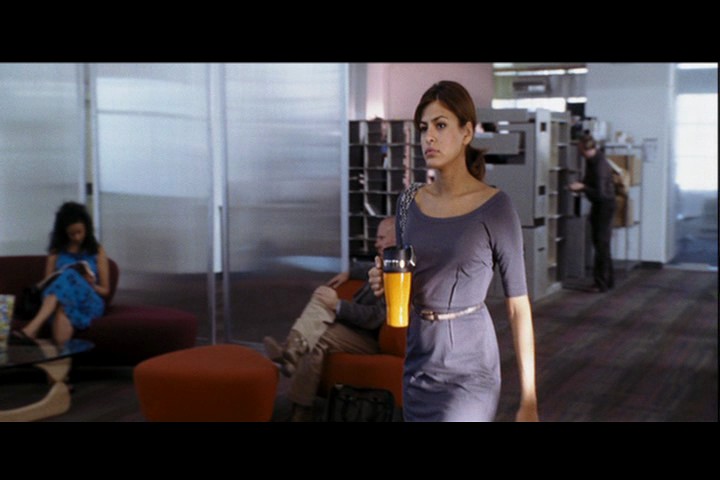

Supplement: S3 Dataset — (ZIP) [file pone.0264302.s003.zip › hitch-00128041.jpg]

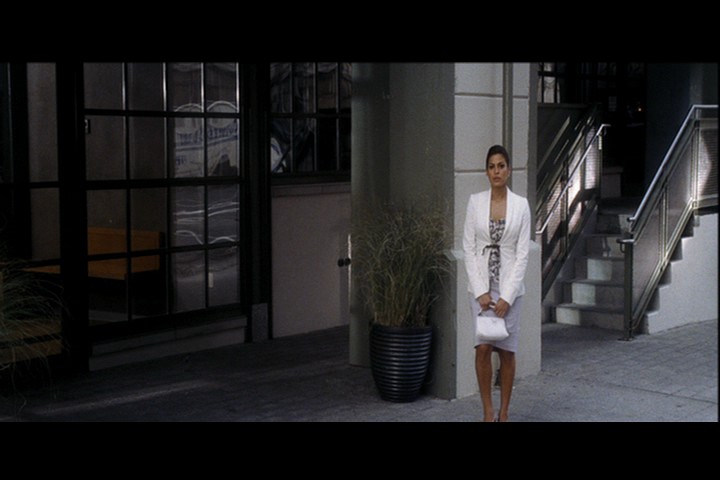

Supplement: S3 Dataset — (ZIP) [file pone.0264302.s003.zip › hitch-00138491.jpg]

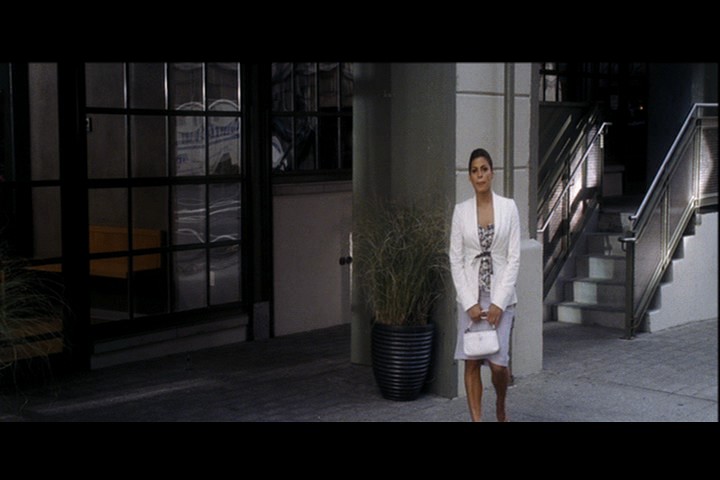

Supplement: S3 Dataset — (ZIP) [file pone.0264302.s003.zip › hitch-00138501.jpg]

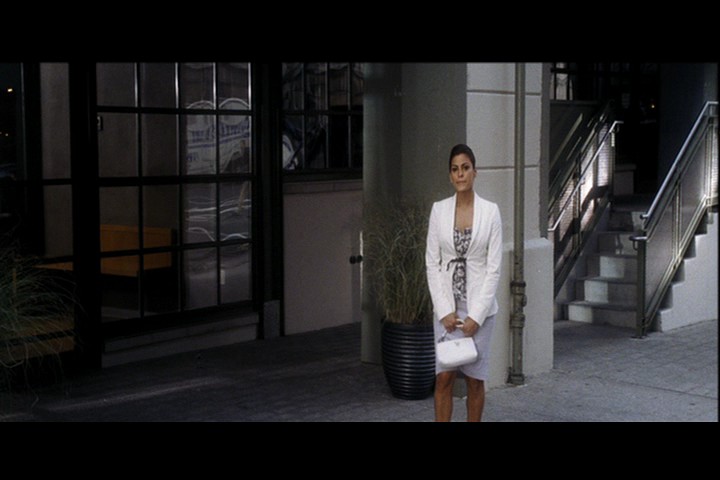

Supplement: S3 Dataset — (ZIP) [file pone.0264302.s003.zip › hitch-00138521.jpg]

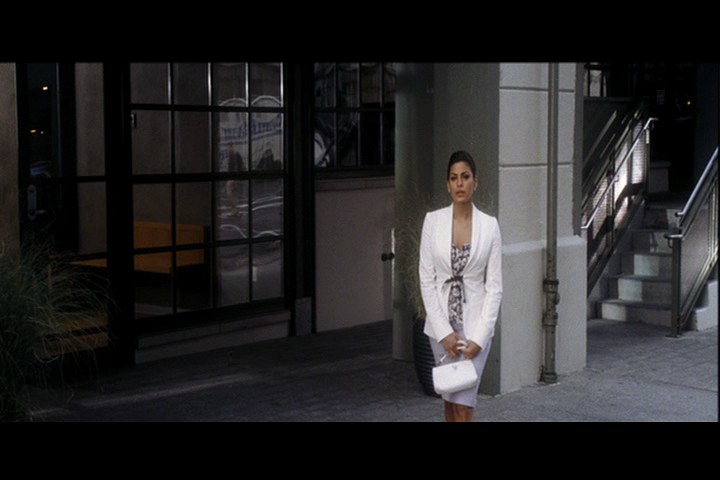

Supplement: S3 Dataset — (ZIP) [file pone.0264302.s003.zip › hitch-00138541.jpg]

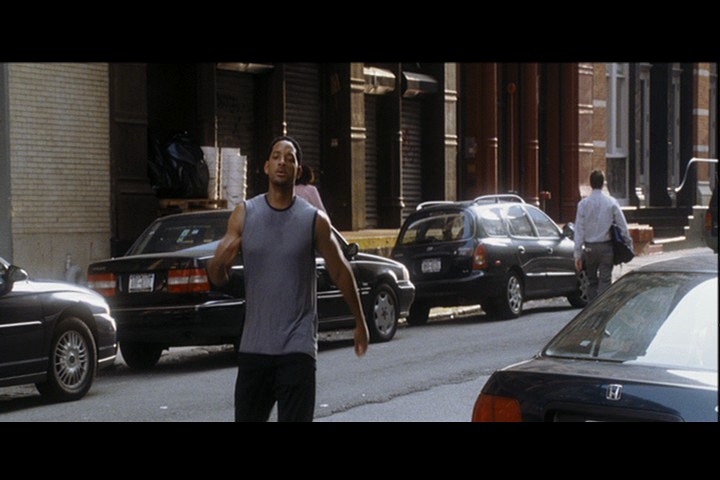

Supplement: S3 Dataset — (ZIP) [file pone.0264302.s003.zip › hitch-00138551.jpg]

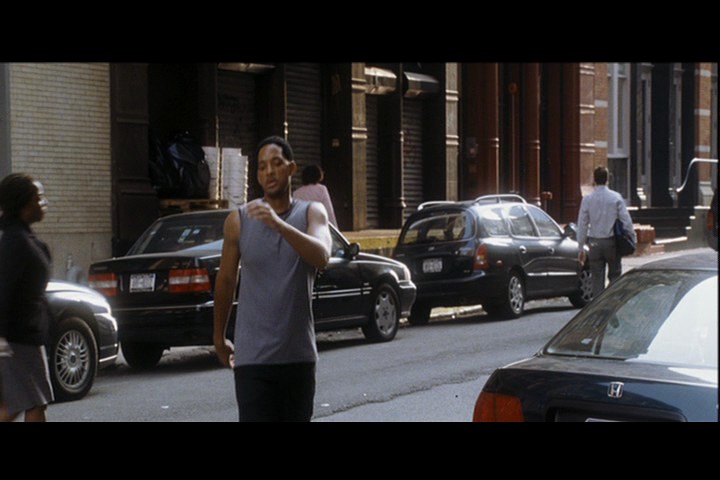

Supplement: S3 Dataset — (ZIP) [file pone.0264302.s003.zip › hitch-00138561.jpg]

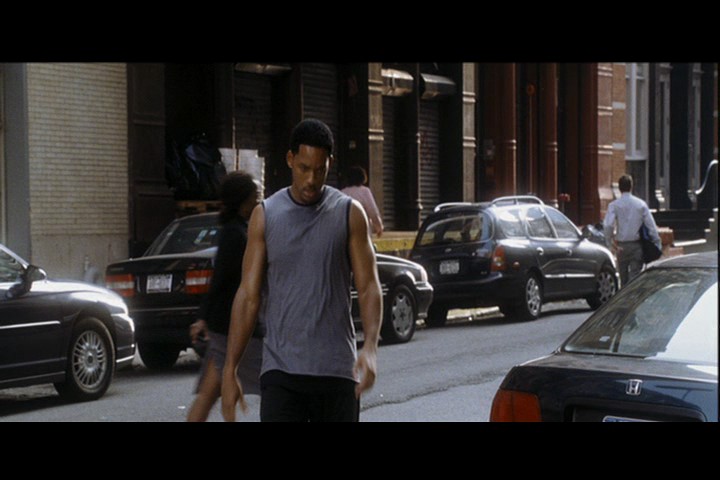

Supplement: S3 Dataset — (ZIP) [file pone.0264302.s003.zip › hitch-00138581.jpg]

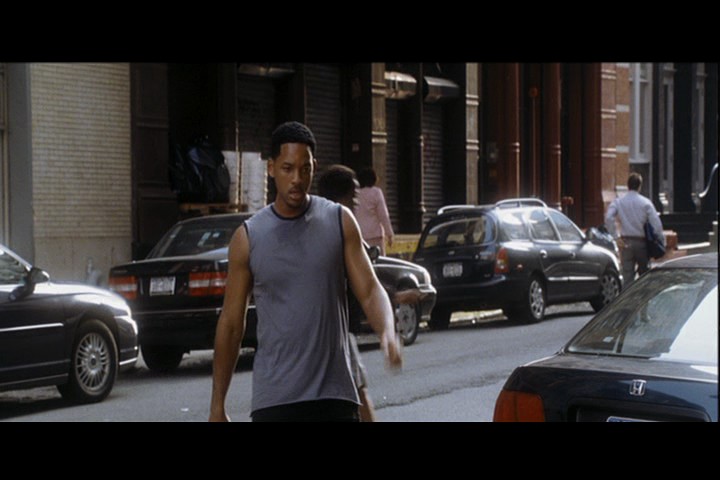

Supplement: S3 Dataset — (ZIP) [file pone.0264302.s003.zip › hitch-00138591.jpg]

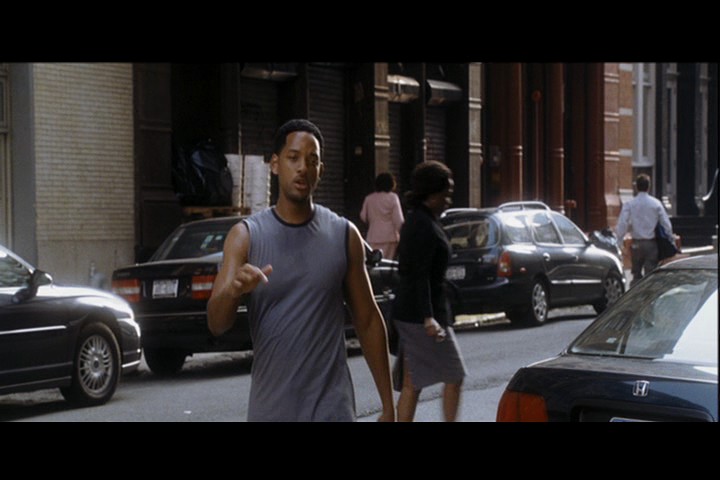

Supplement: S3 Dataset — (ZIP) [file pone.0264302.s003.zip › hitch-00138601.jpg]

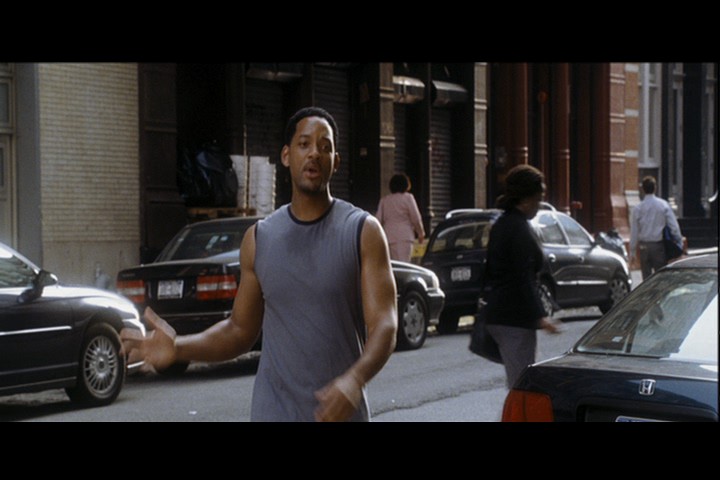

Supplement: S3 Dataset — (ZIP) [file pone.0264302.s003.zip › hitch-00138611.jpg]

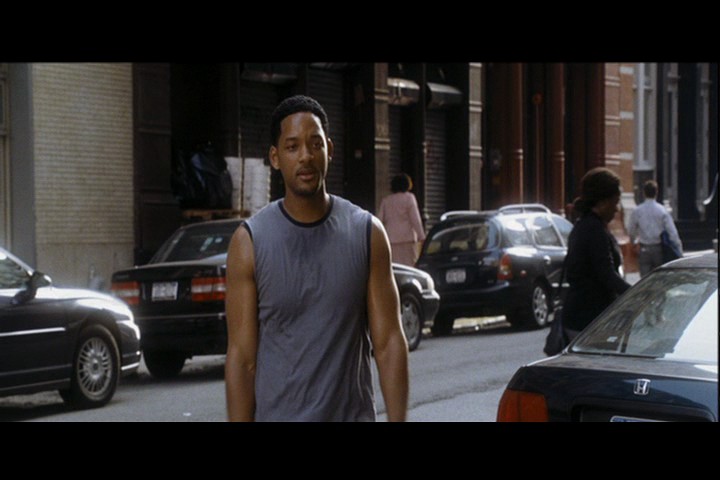

Supplement: S3 Dataset — (ZIP) [file pone.0264302.s003.zip › hitch-00138621.jpg]

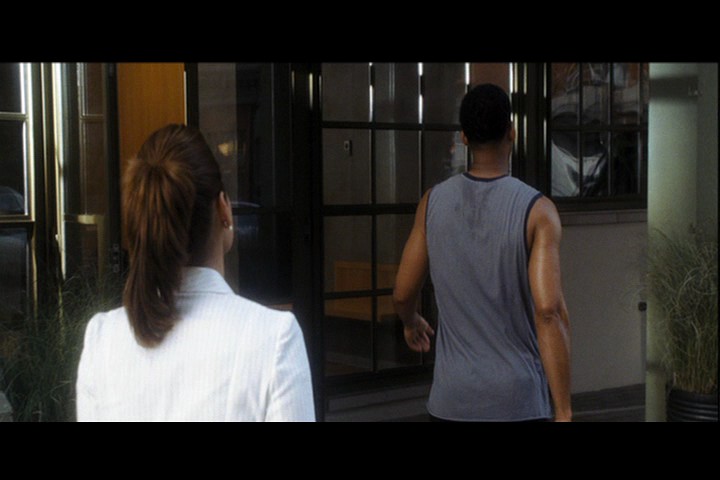

Supplement: S3 Dataset — (ZIP) [file pone.0264302.s003.zip › hitch-00139381.jpg]

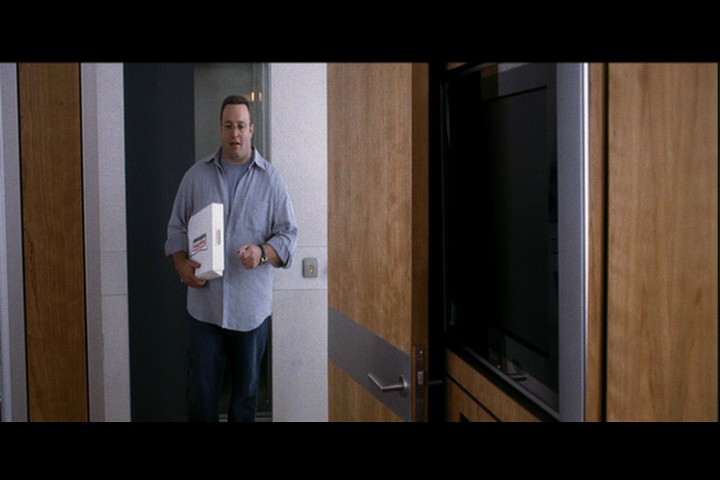

Supplement: S3 Dataset — (ZIP) [file pone.0264302.s003.zip › hitch-00140921.jpg]

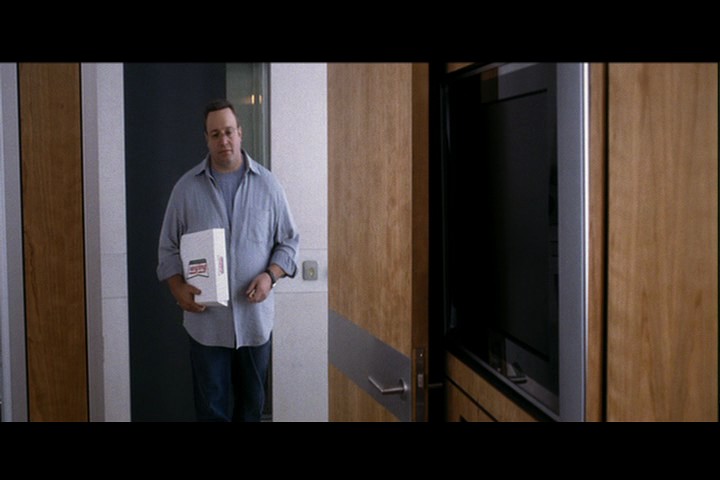

Supplement: S3 Dataset — (ZIP) [file pone.0264302.s003.zip › hitch-00140931.jpg]

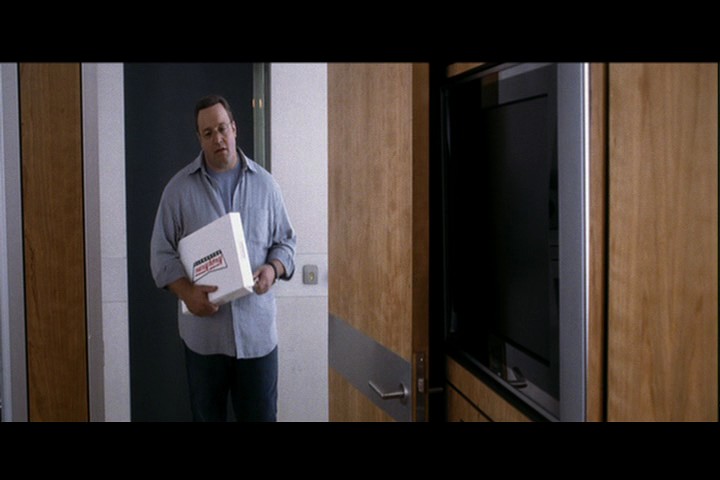

Supplement: S3 Dataset — (ZIP) [file pone.0264302.s003.zip › hitch-00140941.jpg]

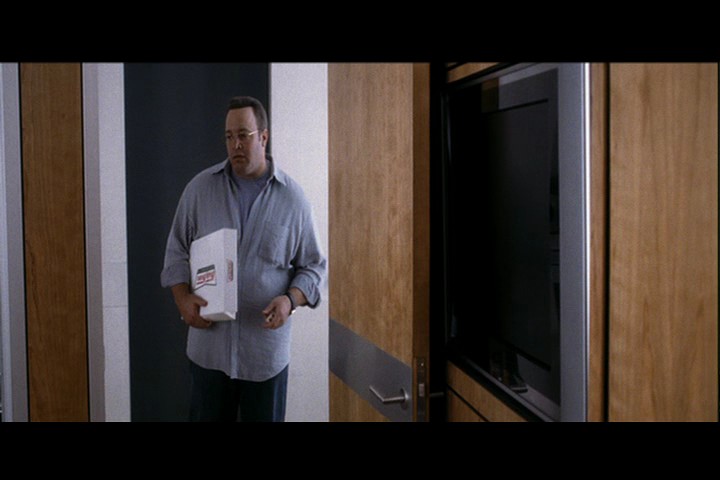

Supplement: S3 Dataset — (ZIP) [file pone.0264302.s003.zip › hitch-00140951.jpg]

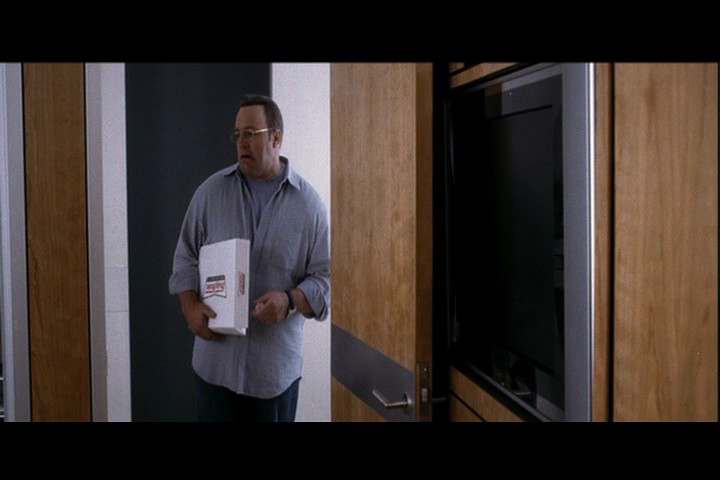

Supplement: S3 Dataset — (ZIP) [file pone.0264302.s003.zip › hitch-00140961.jpg]

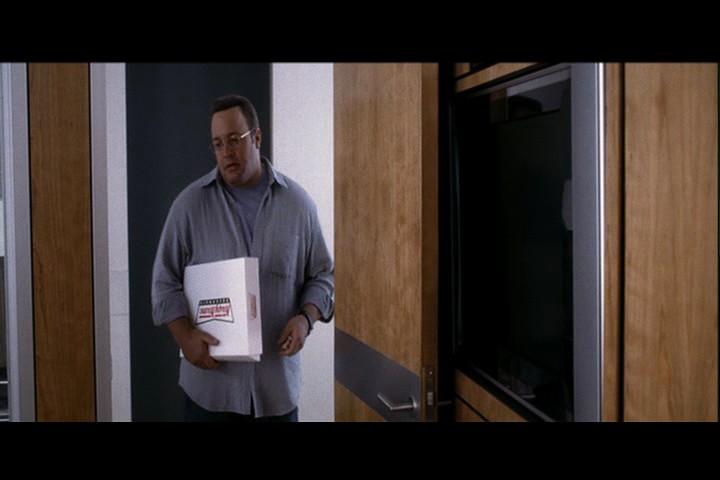

Supplement: S3 Dataset — (ZIP) [file pone.0264302.s003.zip › hitch-00140971.jpg]

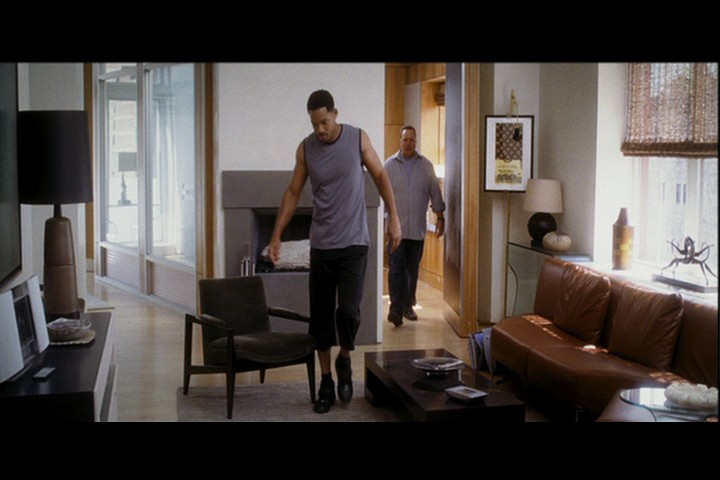

Supplement: S3 Dataset — (ZIP) [file pone.0264302.s003.zip › hitch-00141571.jpg]

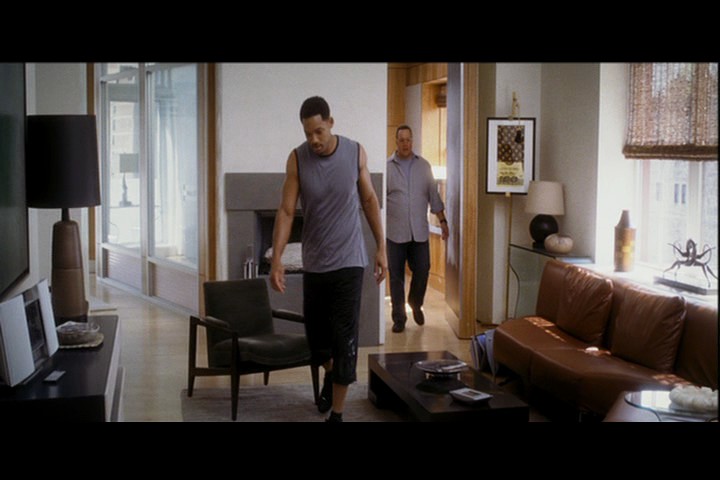

Supplement: S3 Dataset — (ZIP) [file pone.0264302.s003.zip › hitch-00141581.jpg]

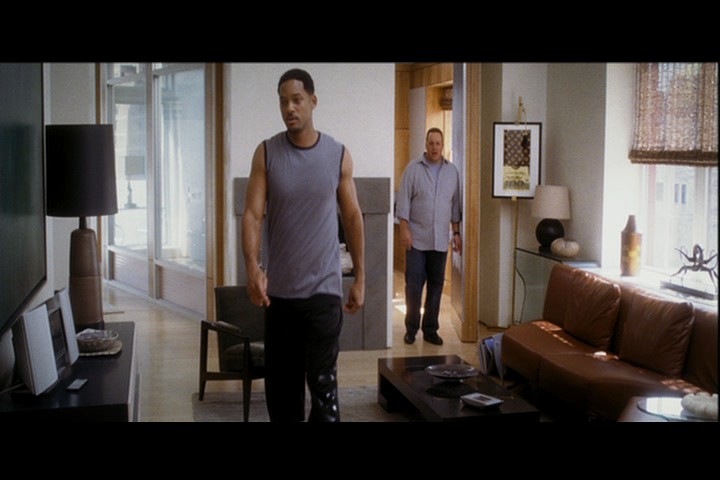

Supplement: S3 Dataset — (ZIP) [file pone.0264302.s003.zip › hitch-00141601.jpg]

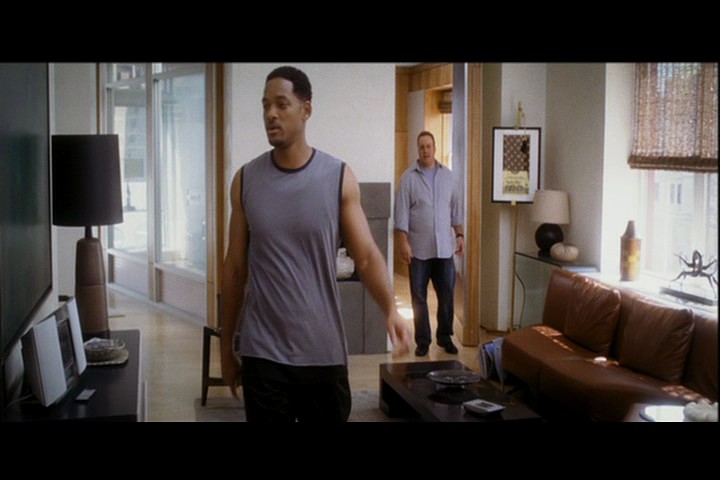

Supplement: S3 Dataset — (ZIP) [file pone.0264302.s003.zip › hitch-00141621.jpg]

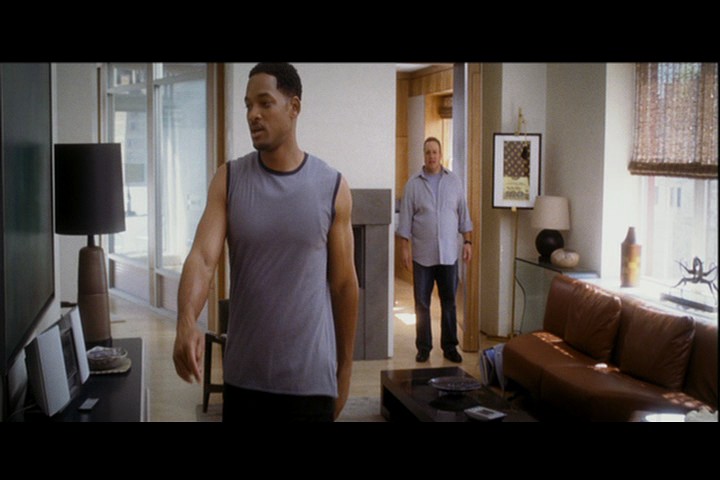

Supplement: S3 Dataset — (ZIP) [file pone.0264302.s003.zip › hitch-00141631.jpg]

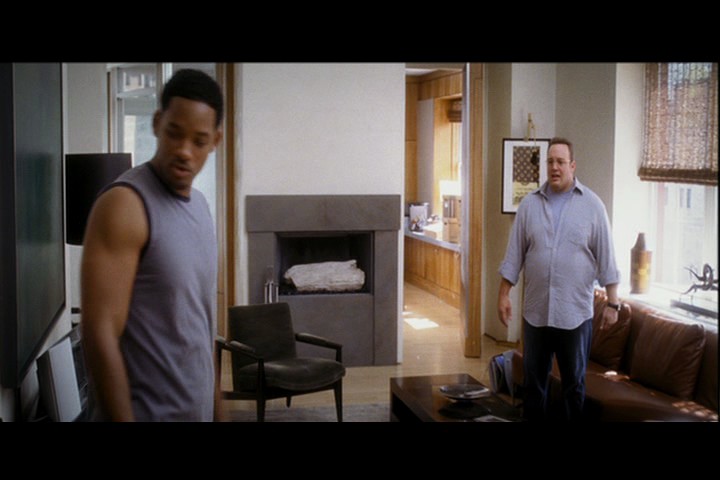

Supplement: S3 Dataset — (ZIP) [file pone.0264302.s003.zip › hitch-00141841.jpg]

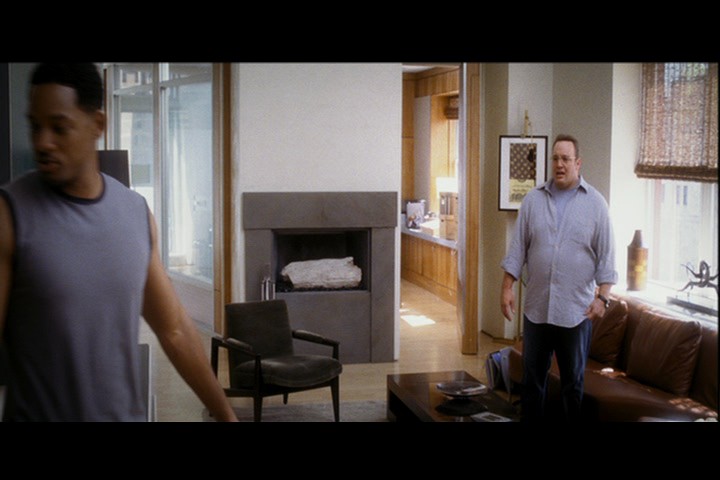

Supplement: S3 Dataset — (ZIP) [file pone.0264302.s003.zip › hitch-00141851.jpg]

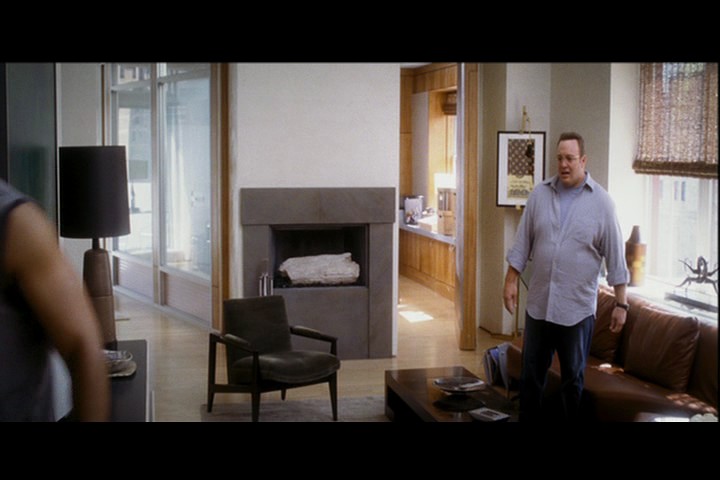

Supplement: S3 Dataset — (ZIP) [file pone.0264302.s003.zip › hitch-00141861.jpg]

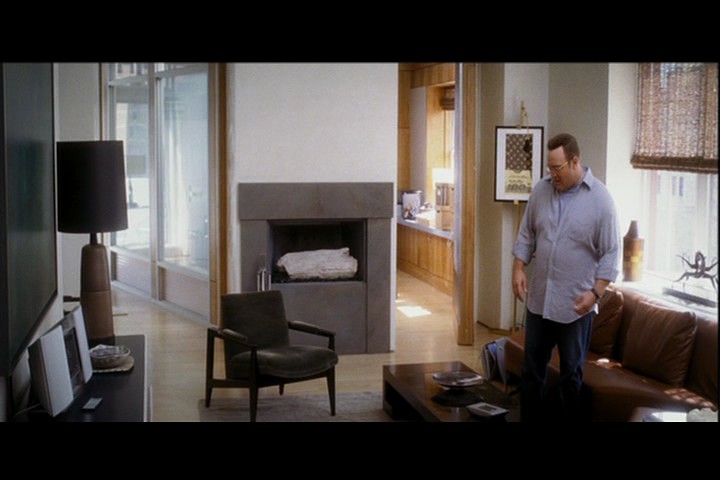

Supplement: S3 Dataset — (ZIP) [file pone.0264302.s003.zip › hitch-00141871.jpg]

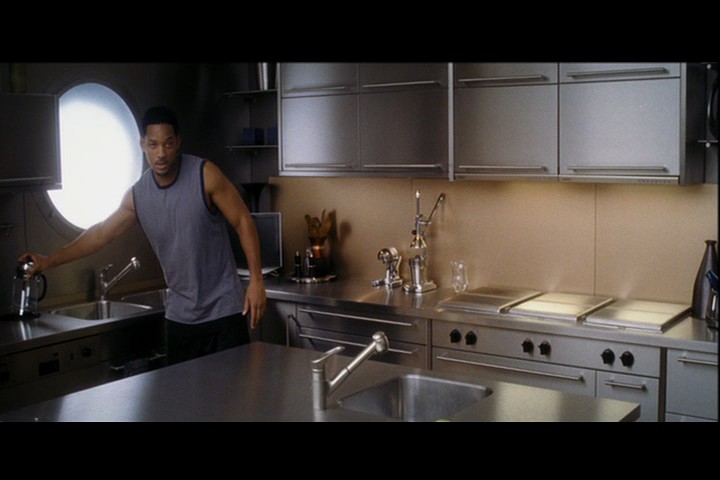

Supplement: S3 Dataset — (ZIP) [file pone.0264302.s003.zip › hitch-00142111.jpg]

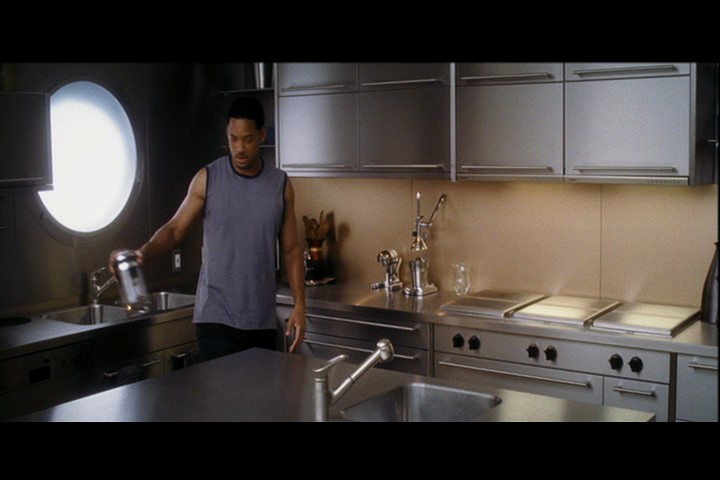

Supplement: S3 Dataset — (ZIP) [file pone.0264302.s003.zip › hitch-00142131.jpg]

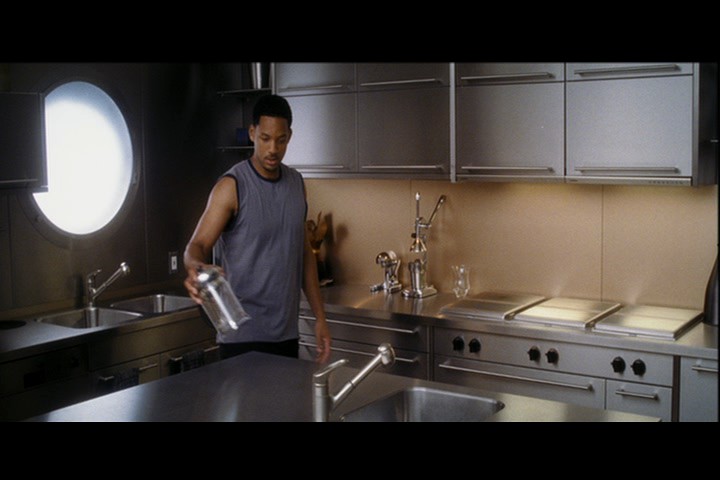

Supplement: S3 Dataset — (ZIP) [file pone.0264302.s003.zip › hitch-00142141.jpg]

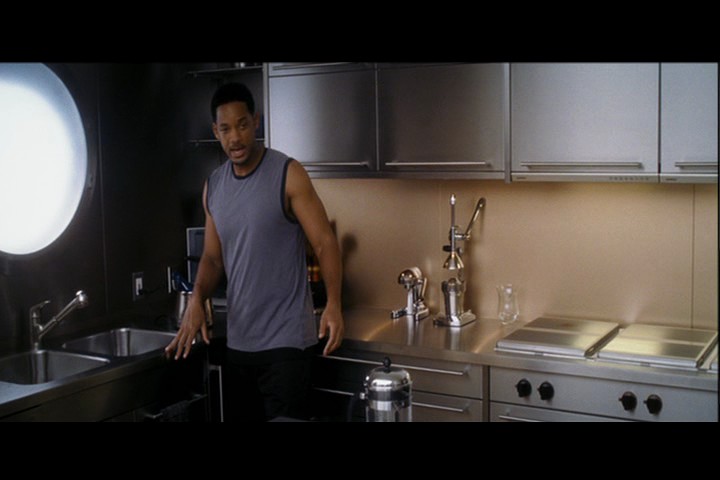

Supplement: S3 Dataset — (ZIP) [file pone.0264302.s003.zip › hitch-00142291.jpg]

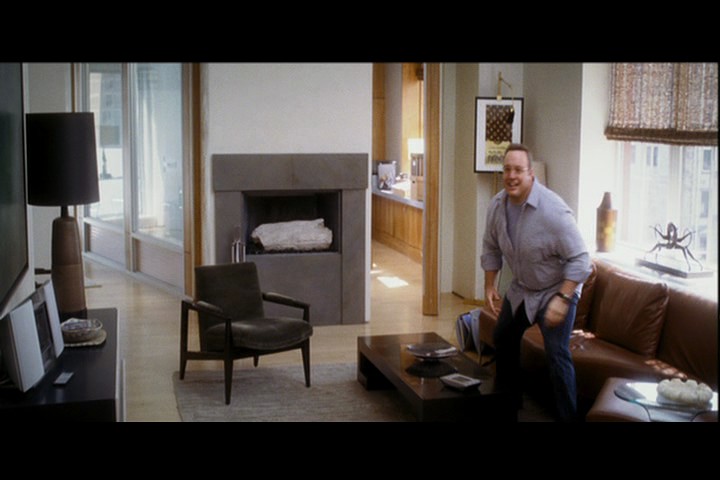

Supplement: S3 Dataset — (ZIP) [file pone.0264302.s003.zip › hitch-00142311.jpg]

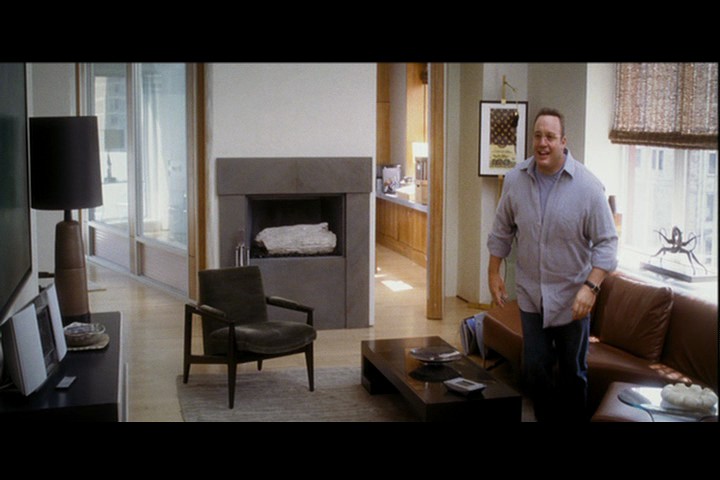

Supplement: S3 Dataset — (ZIP) [file pone.0264302.s003.zip › hitch-00142321.jpg]

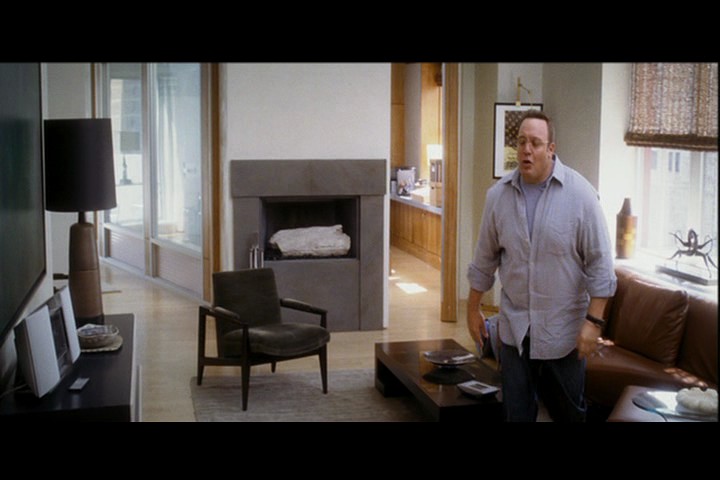

Supplement: S3 Dataset — (ZIP) [file pone.0264302.s003.zip › hitch-00142331.jpg]

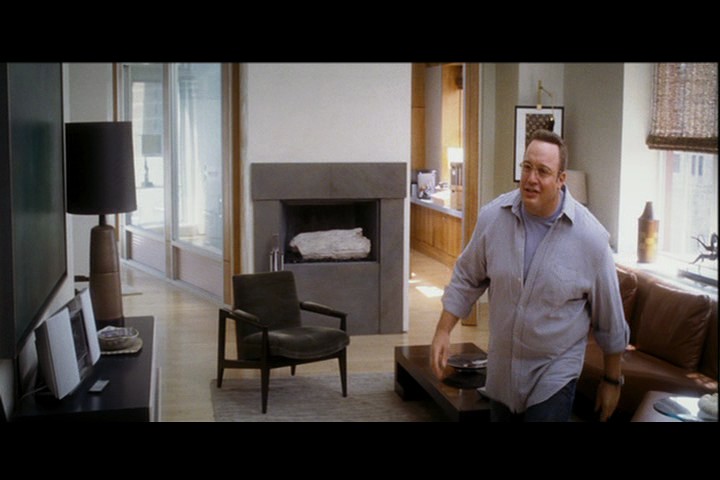

Supplement: S3 Dataset — (ZIP) [file pone.0264302.s003.zip › hitch-00142341.jpg]

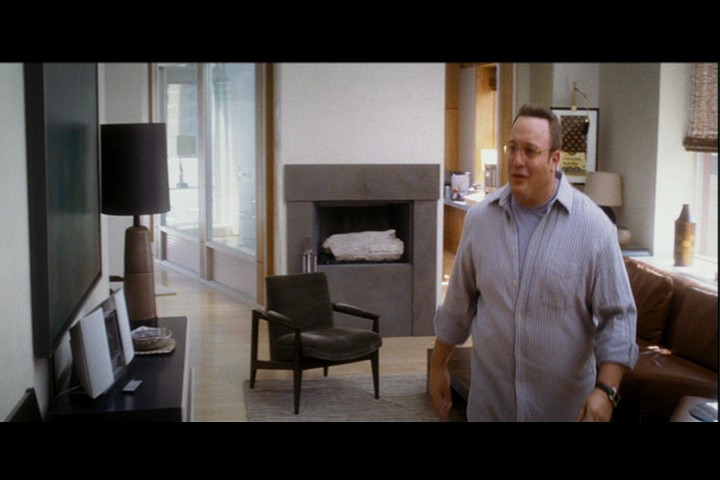

Supplement: S3 Dataset — (ZIP) [file pone.0264302.s003.zip › hitch-00142351.jpg]

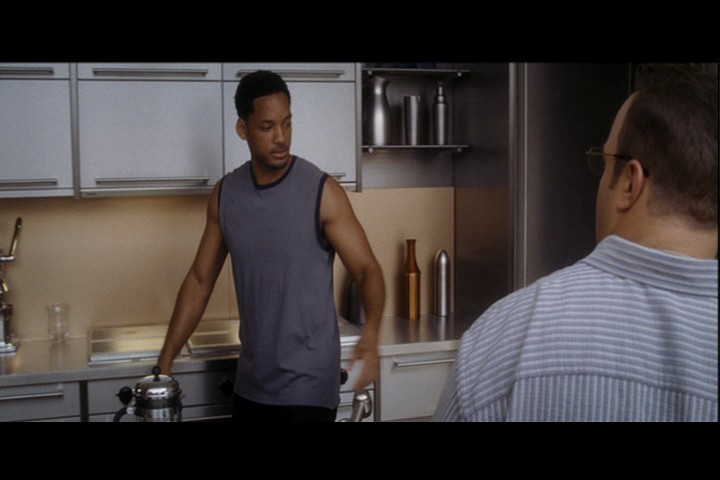

Supplement: S3 Dataset — (ZIP) [file pone.0264302.s003.zip › hitch-00142891.jpg]

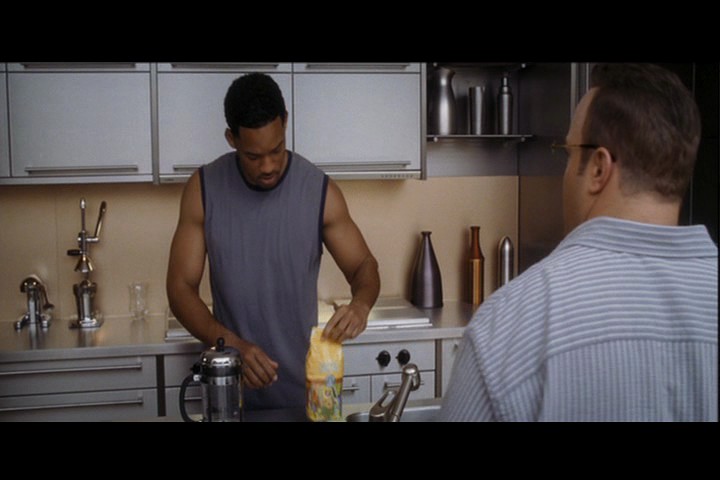

Supplement: S3 Dataset — (ZIP) [file pone.0264302.s003.zip › hitch-00142931.jpg]

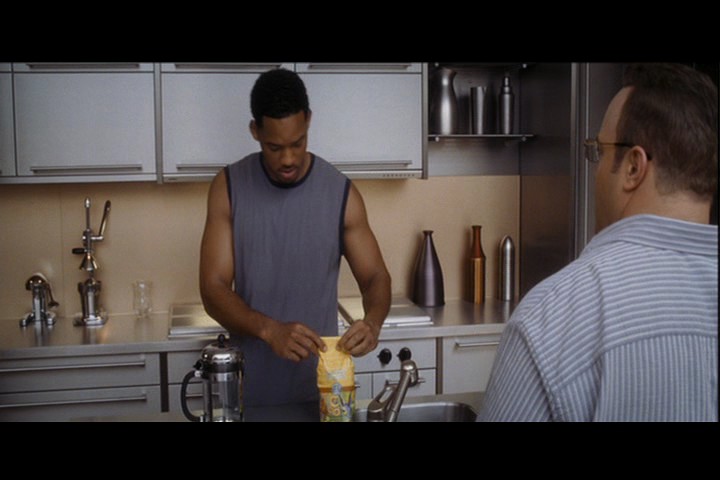

Supplement: S3 Dataset — (ZIP) [file pone.0264302.s003.zip › hitch-00142941.jpg]

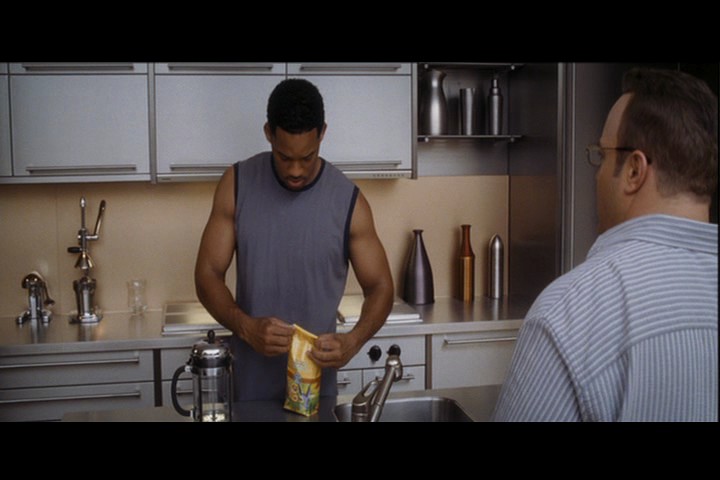

Supplement: S3 Dataset — (ZIP) [file pone.0264302.s003.zip › hitch-00142961.jpg]

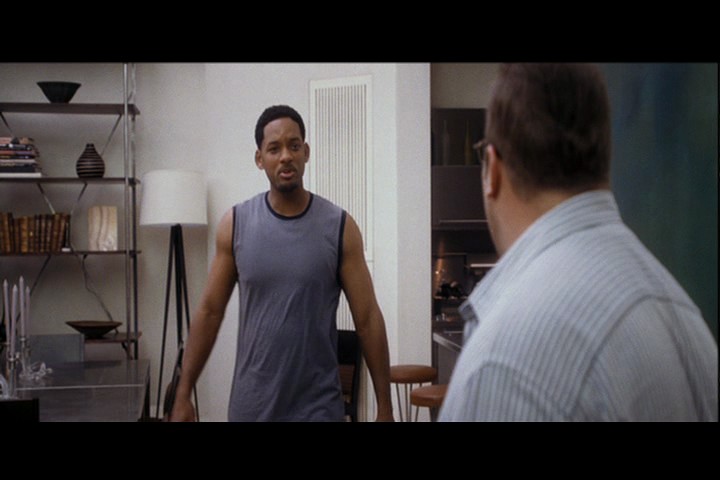

Supplement: S3 Dataset — (ZIP) [file pone.0264302.s003.zip › hitch-00143691.jpg]

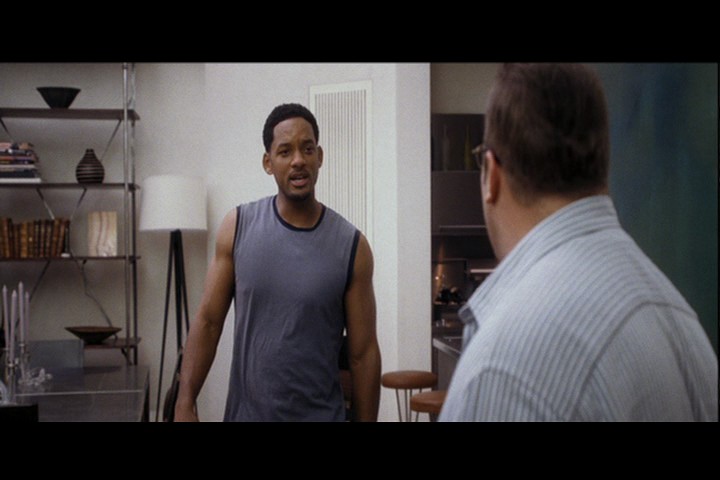

Supplement: S3 Dataset — (ZIP) [file pone.0264302.s003.zip › hitch-00143701.jpg]

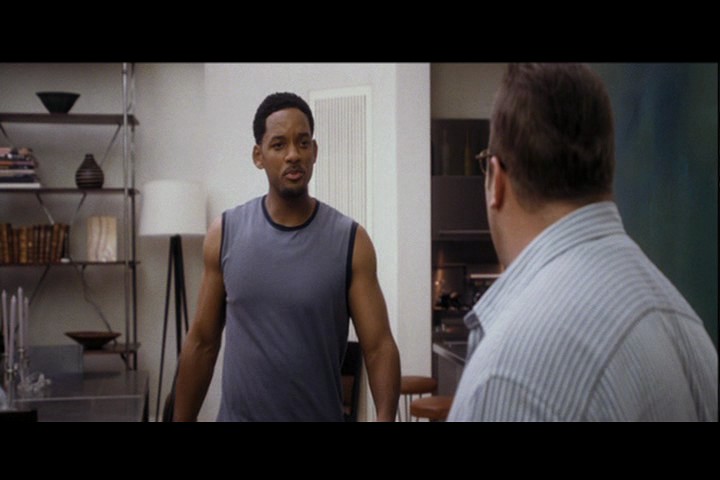

Supplement: S3 Dataset — (ZIP) [file pone.0264302.s003.zip › hitch-00143711.jpg]

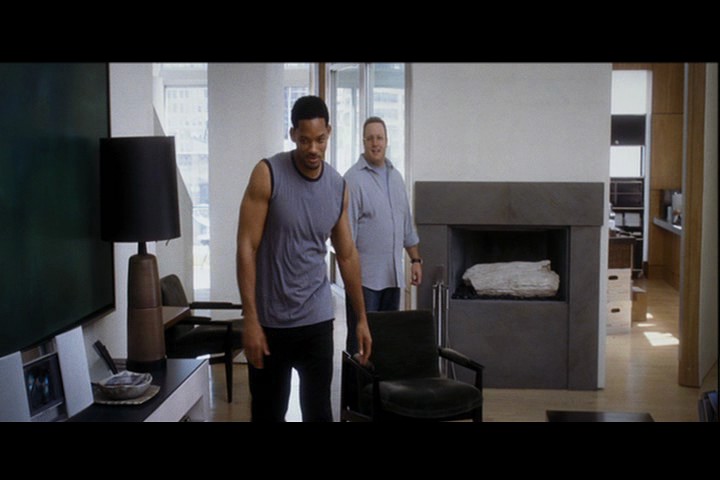

Supplement: S3 Dataset — (ZIP) [file pone.0264302.s003.zip › hitch-00143941.jpg]

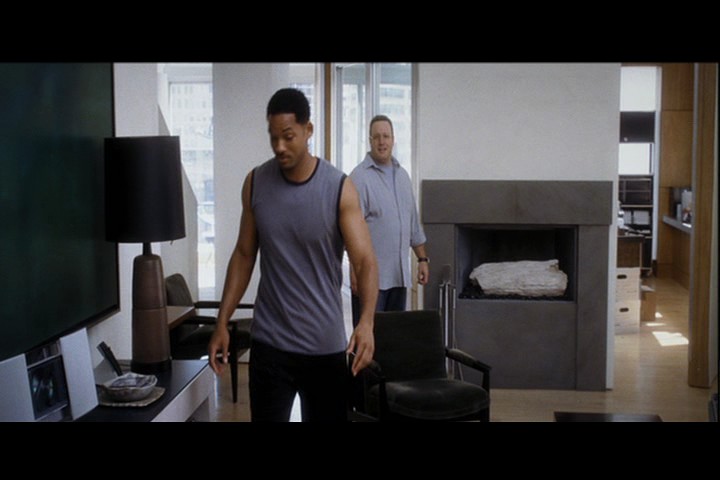

Supplement: S3 Dataset — (ZIP) [file pone.0264302.s003.zip › hitch-00143951.jpg]

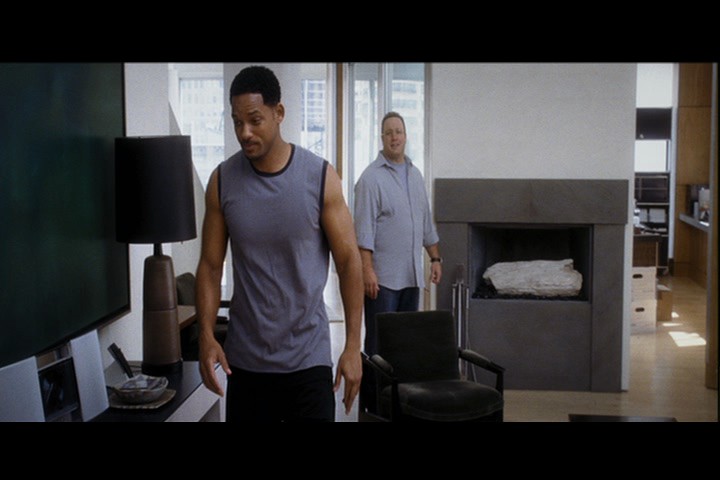

Supplement: S3 Dataset — (ZIP) [file pone.0264302.s003.zip › hitch-00143961.jpg]

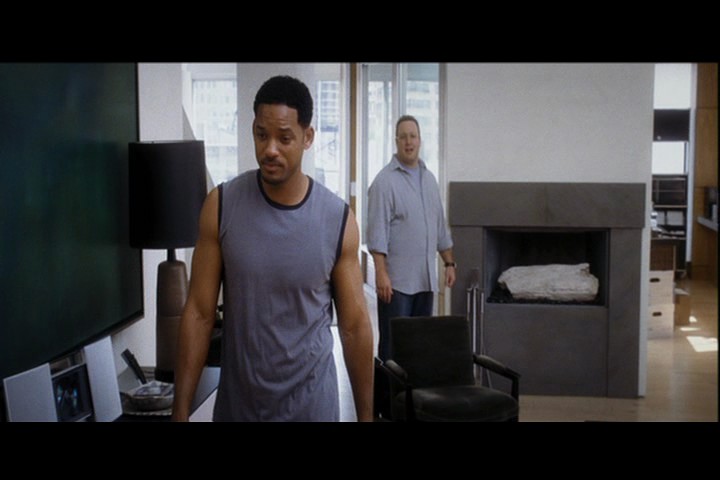

Supplement: S3 Dataset — (ZIP) [file pone.0264302.s003.zip › hitch-00143971.jpg]

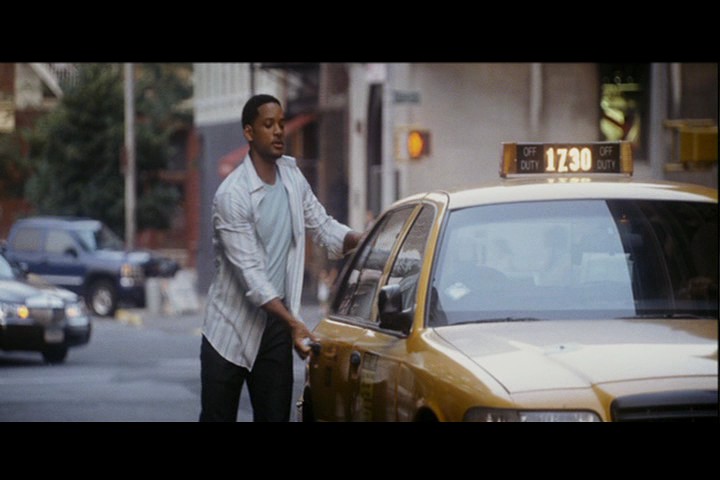

Supplement: S3 Dataset — (ZIP) [file pone.0264302.s003.zip › hitch-00144561.jpg]

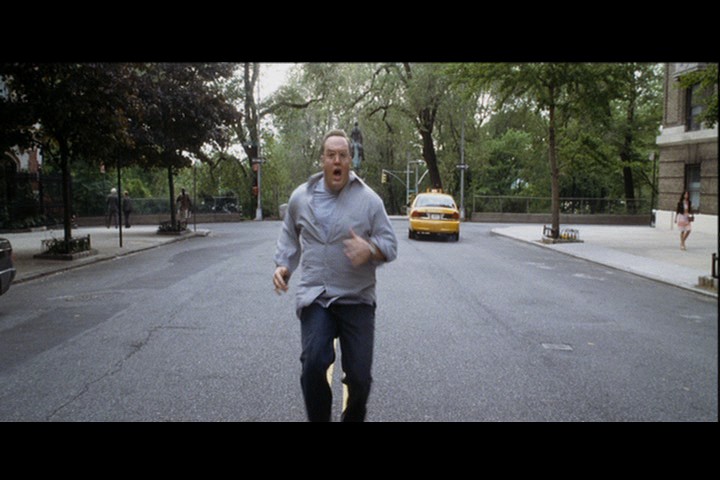

Supplement: S3 Dataset — (ZIP) [file pone.0264302.s003.zip › hitch-00145011.jpg]

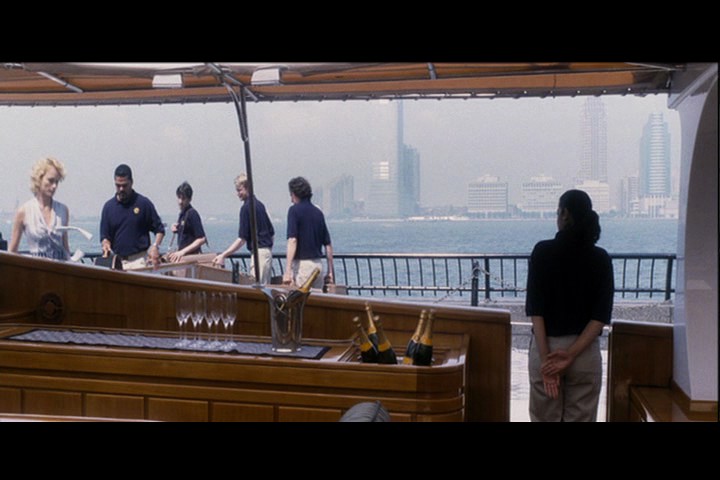

Supplement: S3 Dataset — (ZIP) [file pone.0264302.s003.zip › hitch-00145561.jpg]

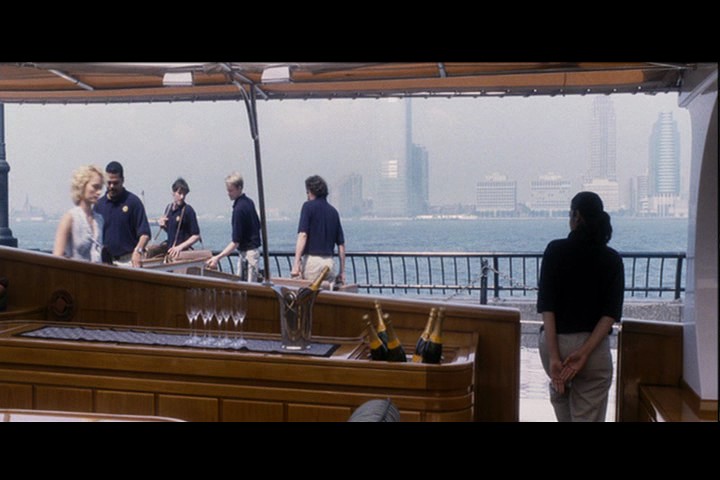

Supplement: S3 Dataset — (ZIP) [file pone.0264302.s003.zip › hitch-00145571.jpg]

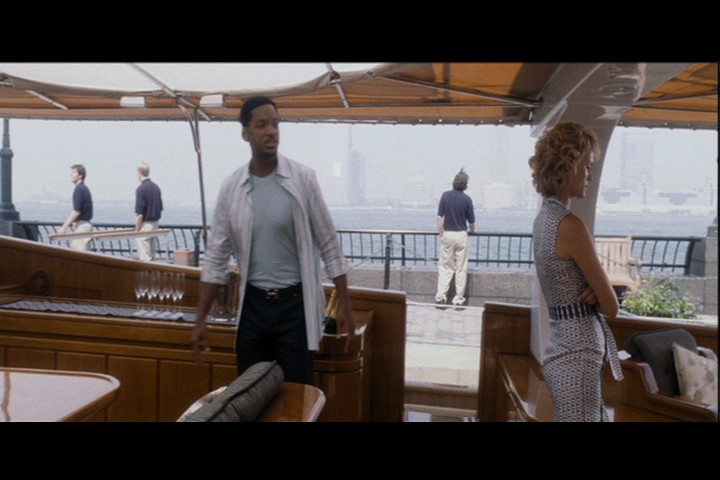

Supplement: S3 Dataset — (ZIP) [file pone.0264302.s003.zip › hitch-00146381.jpg]

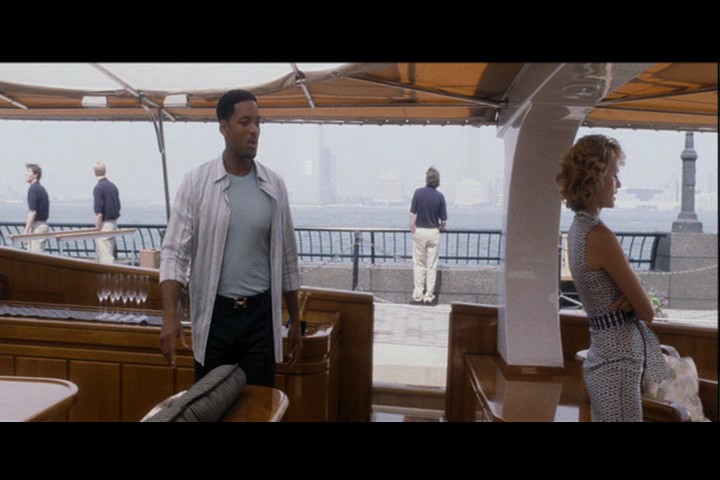

Supplement: S3 Dataset — (ZIP) [file pone.0264302.s003.zip › hitch-00146391.jpg]

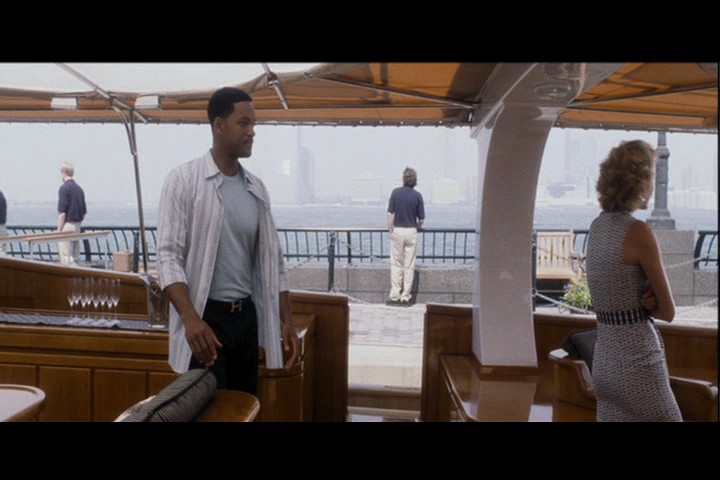

Supplement: S3 Dataset — (ZIP) [file pone.0264302.s003.zip › hitch-00146401.jpg]

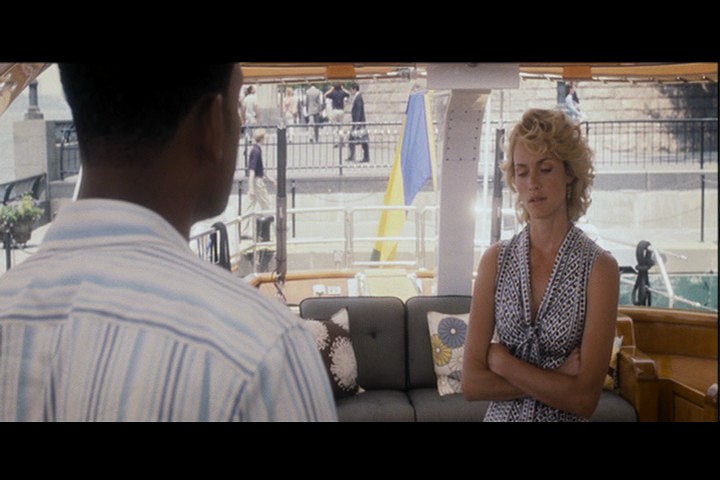

Supplement: S3 Dataset — (ZIP) [file pone.0264302.s003.zip › hitch-00146631.jpg]

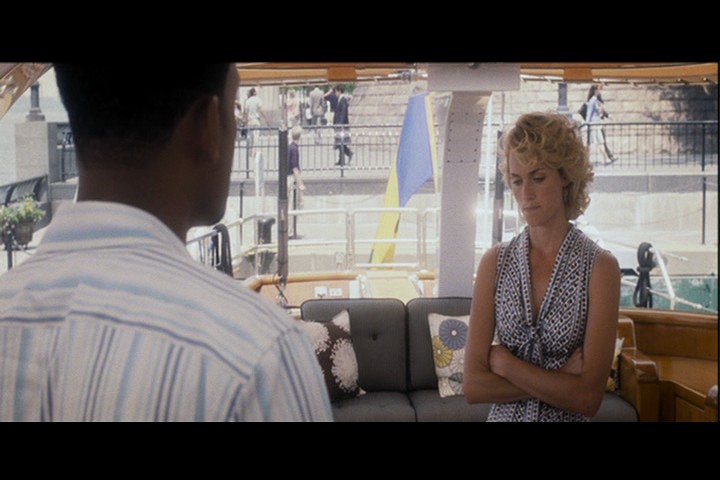

Supplement: S3 Dataset — (ZIP) [file pone.0264302.s003.zip › hitch-00146641.jpg]

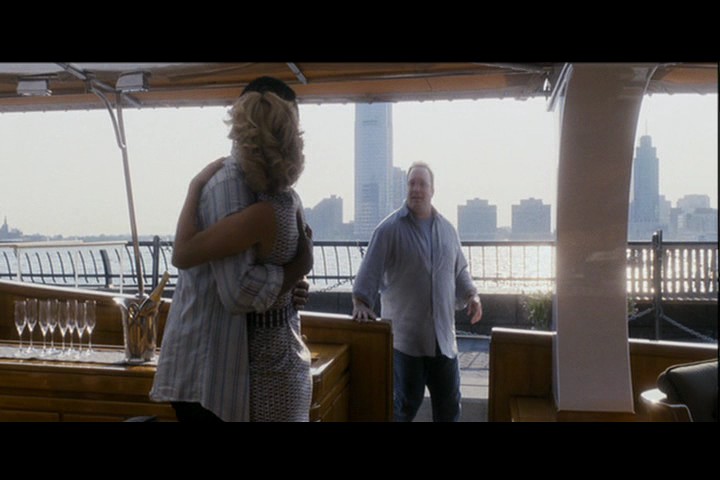

Supplement: S3 Dataset — (ZIP) [file pone.0264302.s003.zip › hitch-00148531.jpg]

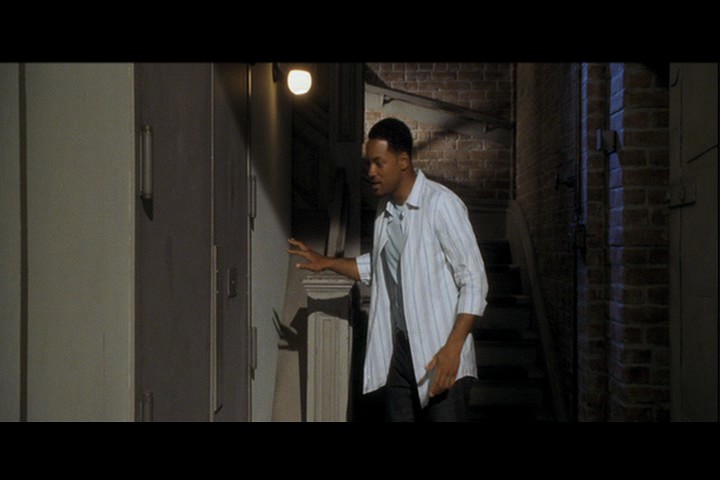

Supplement: S3 Dataset — (ZIP) [file pone.0264302.s003.zip › hitch-00150461.jpg]

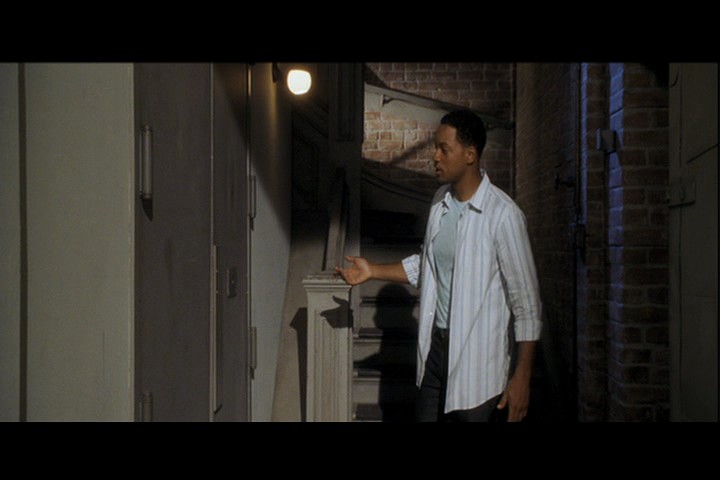

Supplement: S3 Dataset — (ZIP) [file pone.0264302.s003.zip › hitch-00150481.jpg]

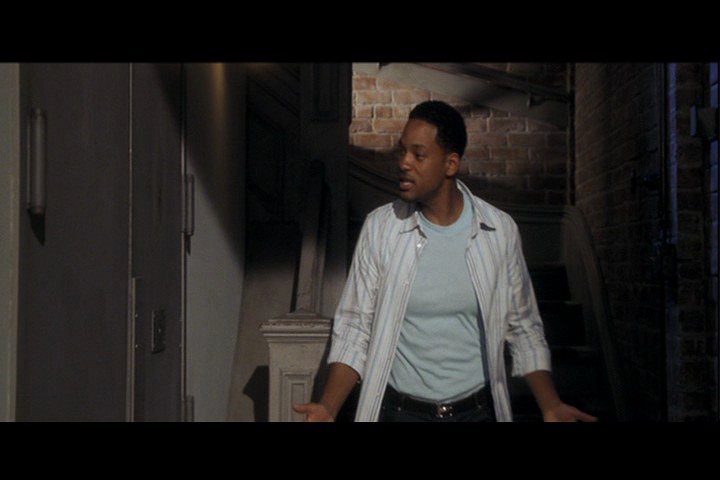

Supplement: S3 Dataset — (ZIP) [file pone.0264302.s003.zip › hitch-00152601.jpg]

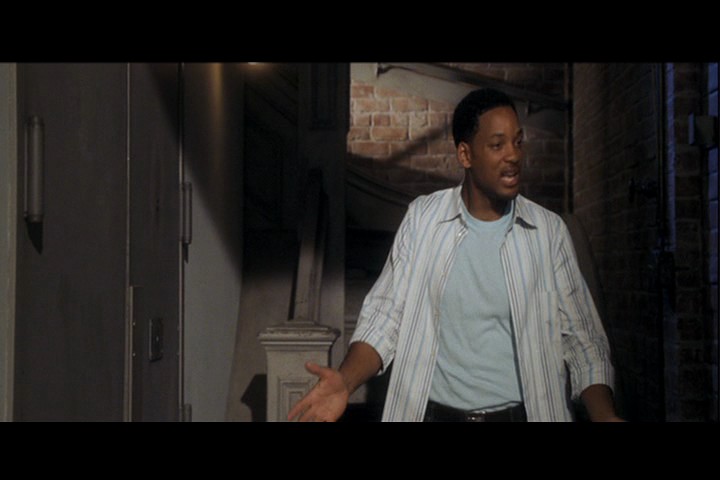

Supplement: S3 Dataset — (ZIP) [file pone.0264302.s003.zip › hitch-00152611.jpg]

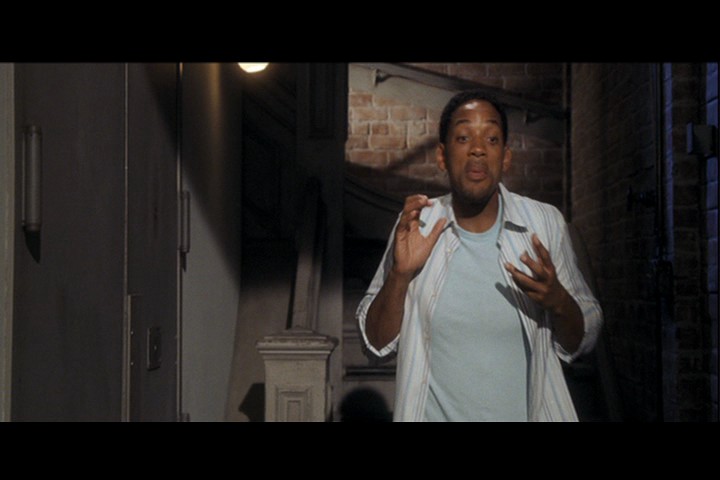

Supplement: S3 Dataset — (ZIP) [file pone.0264302.s003.zip › hitch-00152621.jpg]

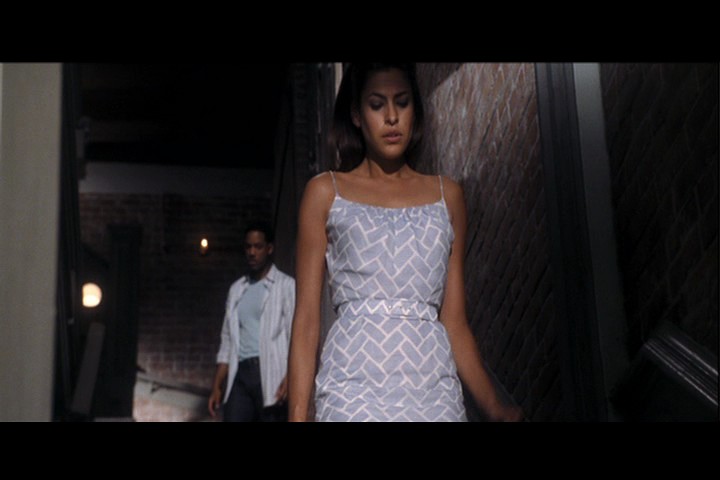

Supplement: S3 Dataset — (ZIP) [file pone.0264302.s003.zip › hitch-00155251.jpg]

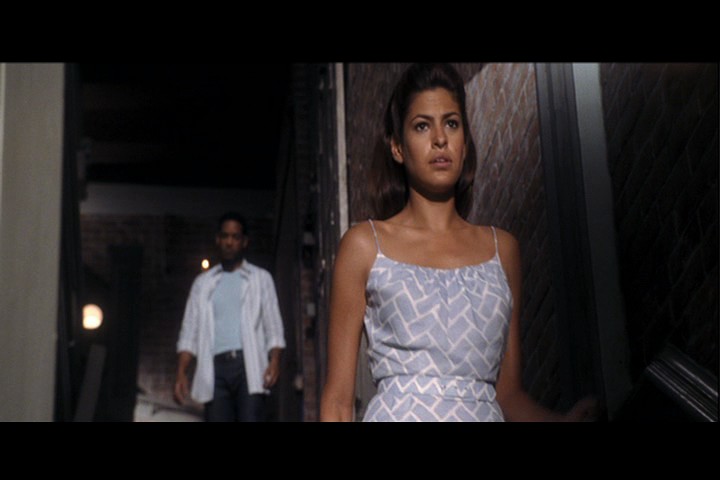

Supplement: S3 Dataset — (ZIP) [file pone.0264302.s003.zip › hitch-00155271.jpg]
