# Supplementary figures and images for: LHPE-nets: A lightweight 2D and 3D human pose estimation model with well-structural deep networks and multi-view pose sample simplification method (part 5 of 8)
Source: PLoS One. 2022 Feb 23;17(2):e0264302. doi: 10.1371/journal.pone.0264302 (PMC8865690; doi:10.1371/journal.pone.0264302)

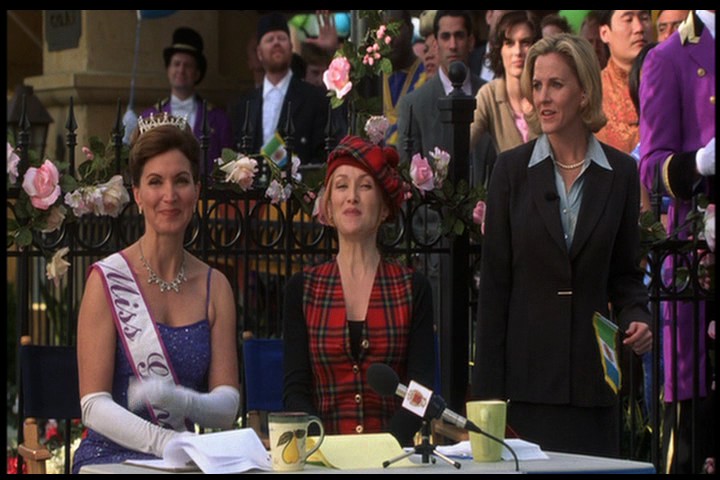

Supplement: S3 Dataset — (ZIP) [file pone.0264302.s003.zip › princess-diaries-2-00077521.jpg]

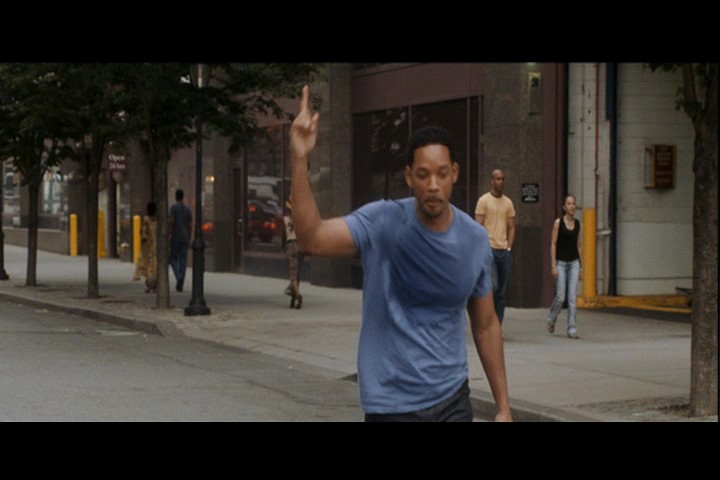

Supplement: S3 Dataset — (ZIP) [file pone.0264302.s003.zip › hitch-00061961.jpg]

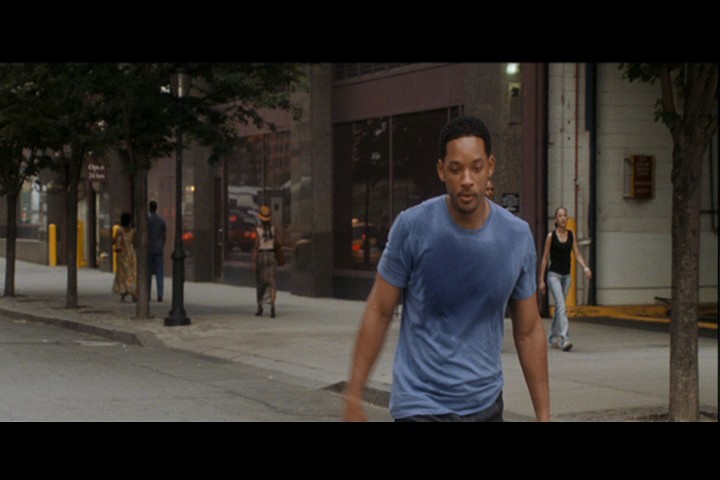

Supplement: S3 Dataset — (ZIP) [file pone.0264302.s003.zip › hitch-00061971.jpg]

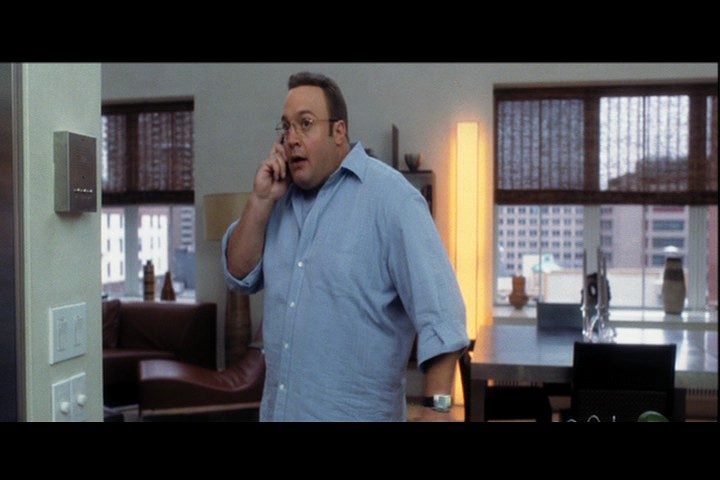

Supplement: S3 Dataset — (ZIP) [file pone.0264302.s003.zip › hitch-00064121.jpg]

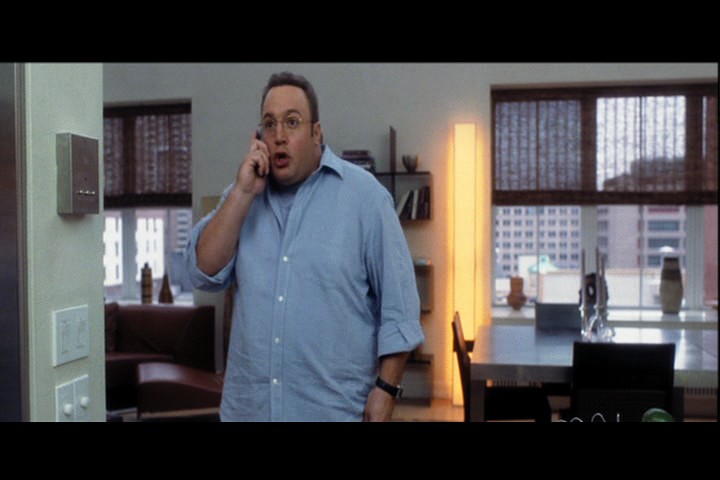

Supplement: S3 Dataset — (ZIP) [file pone.0264302.s003.zip › hitch-00064131.jpg]

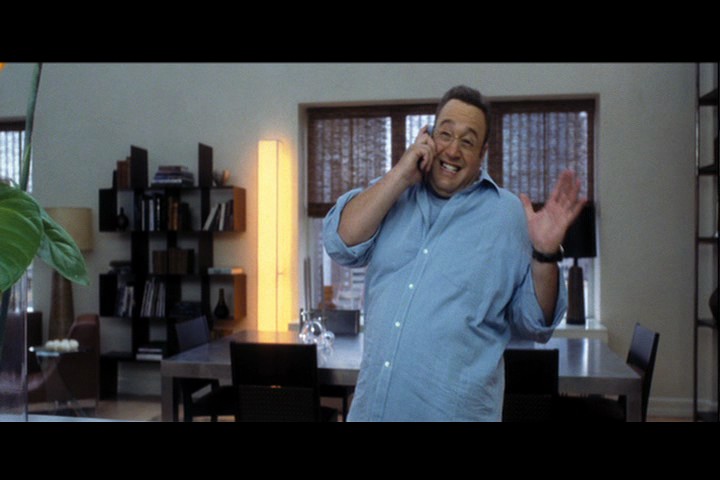

Supplement: S3 Dataset — (ZIP) [file pone.0264302.s003.zip › hitch-00064481.jpg]

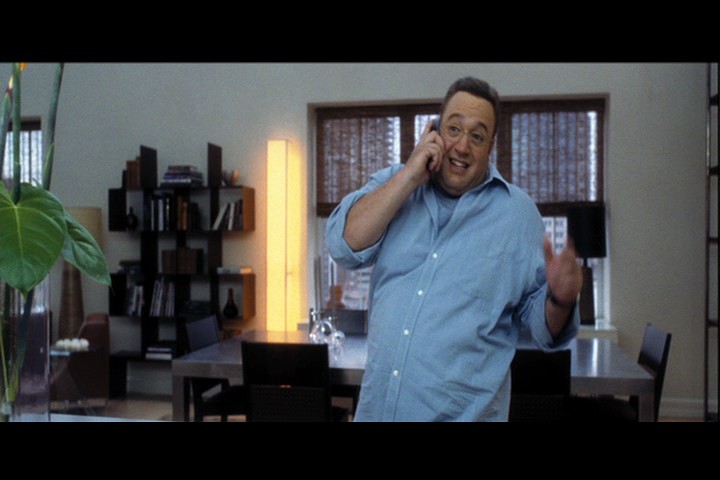

Supplement: S3 Dataset — (ZIP) [file pone.0264302.s003.zip › hitch-00064491.jpg]

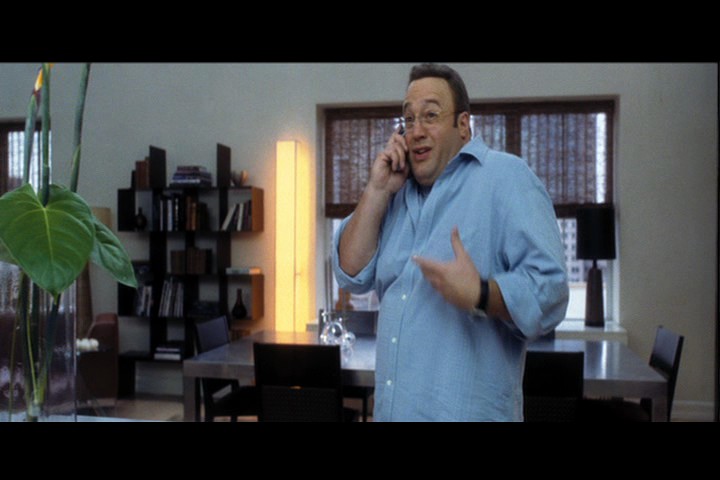

Supplement: S3 Dataset — (ZIP) [file pone.0264302.s003.zip › hitch-00064501.jpg]

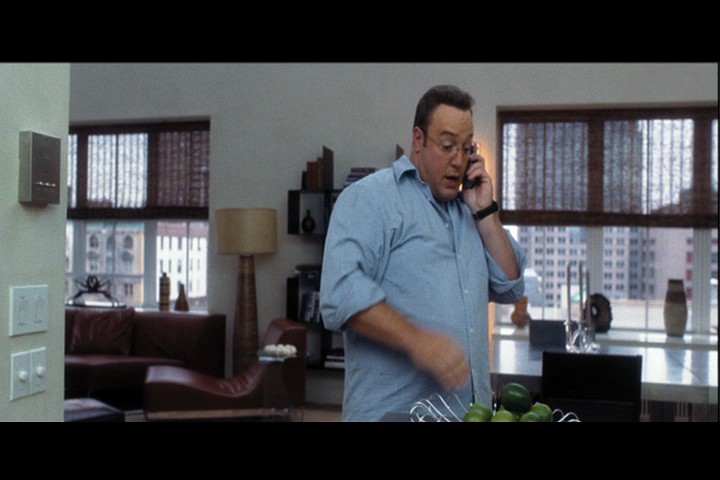

Supplement: S3 Dataset — (ZIP) [file pone.0264302.s003.zip › hitch-00064721.jpg]

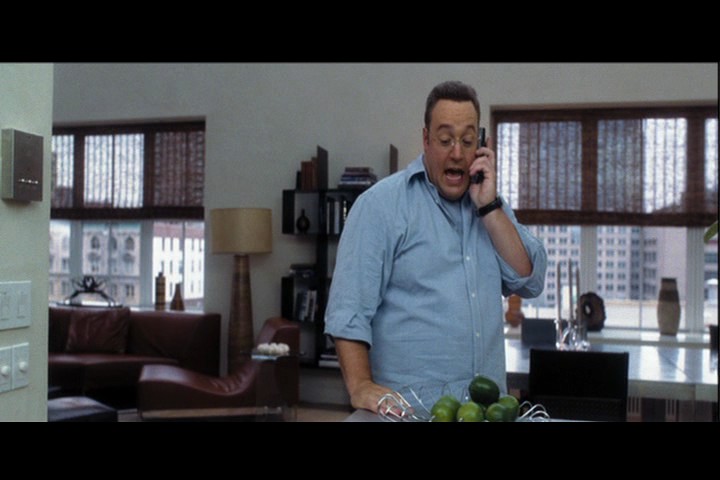

Supplement: S3 Dataset — (ZIP) [file pone.0264302.s003.zip › hitch-00064731.jpg]

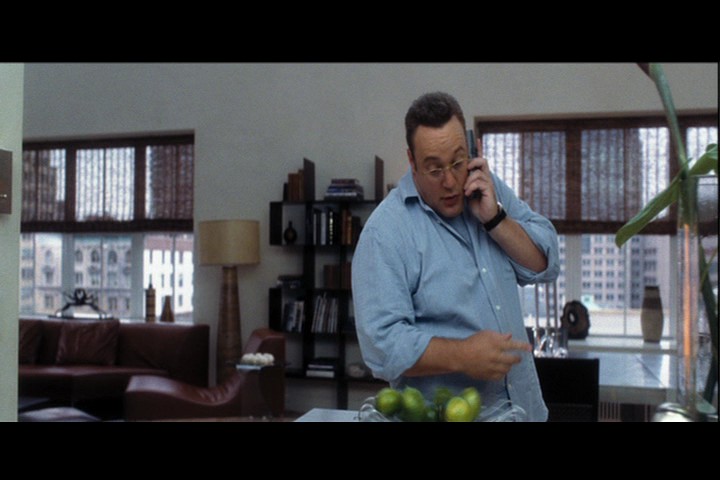

Supplement: S3 Dataset — (ZIP) [file pone.0264302.s003.zip › hitch-00064751.jpg]

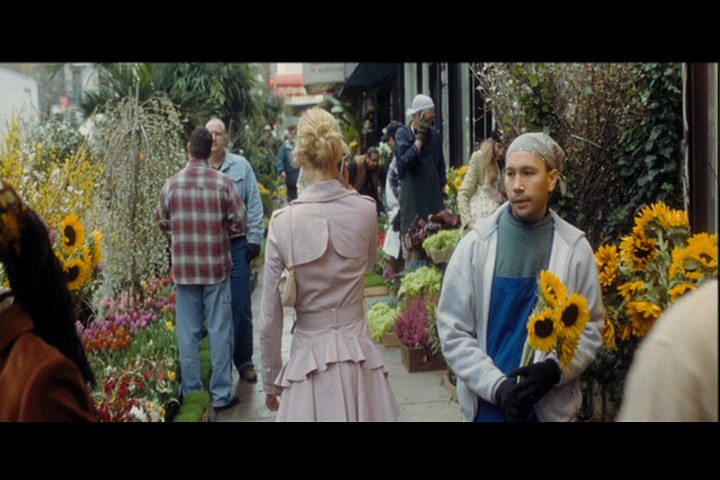

Supplement: S3 Dataset — (ZIP) [file pone.0264302.s003.zip › hitch-00065081.jpg]

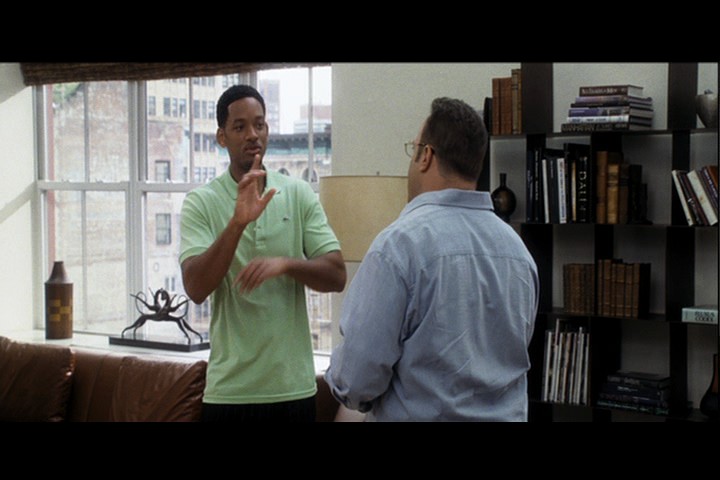

Supplement: S3 Dataset — (ZIP) [file pone.0264302.s003.zip › hitch-00071551.jpg]

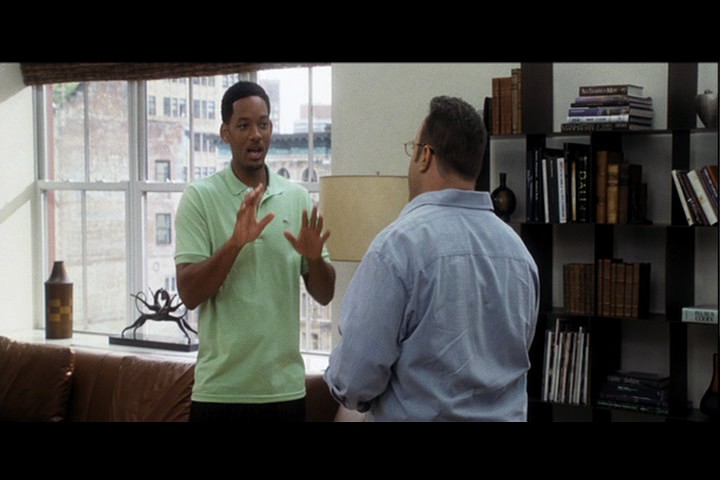

Supplement: S3 Dataset — (ZIP) [file pone.0264302.s003.zip › hitch-00071561.jpg]

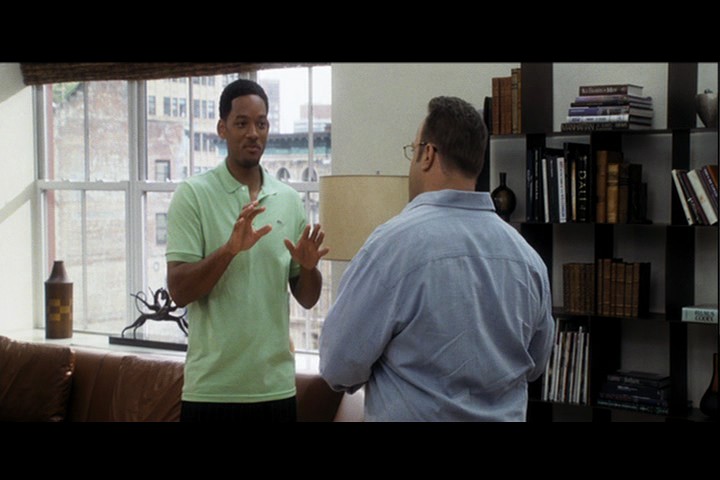

Supplement: S3 Dataset — (ZIP) [file pone.0264302.s003.zip › hitch-00071571.jpg]

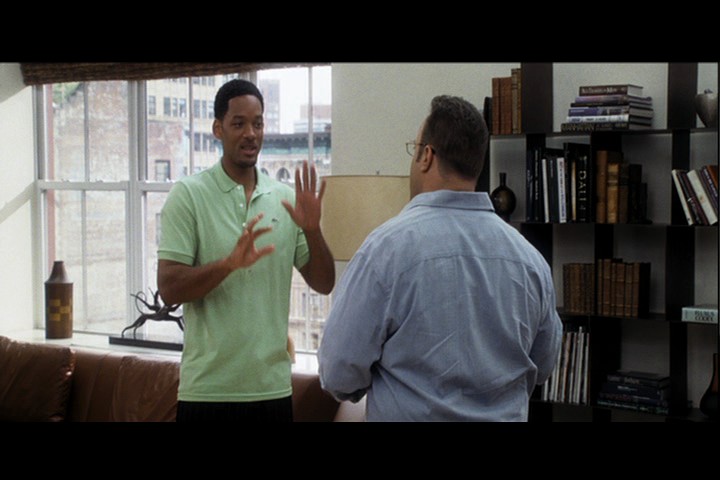

Supplement: S3 Dataset — (ZIP) [file pone.0264302.s003.zip › hitch-00071581.jpg]

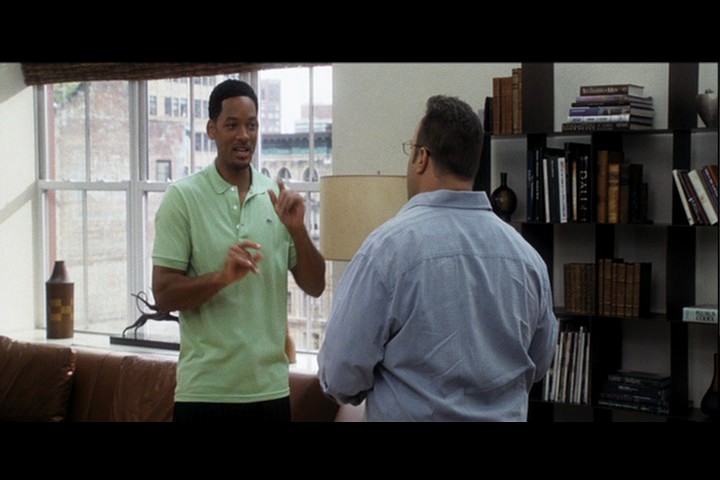

Supplement: S3 Dataset — (ZIP) [file pone.0264302.s003.zip › hitch-00071591.jpg]

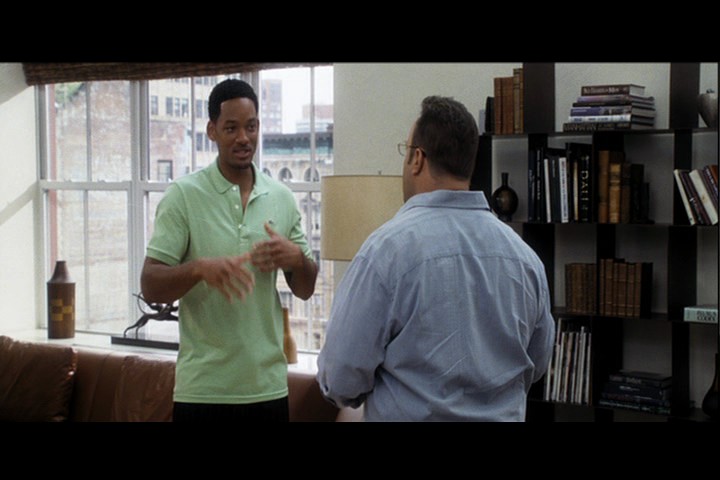

Supplement: S3 Dataset — (ZIP) [file pone.0264302.s003.zip › hitch-00071601.jpg]

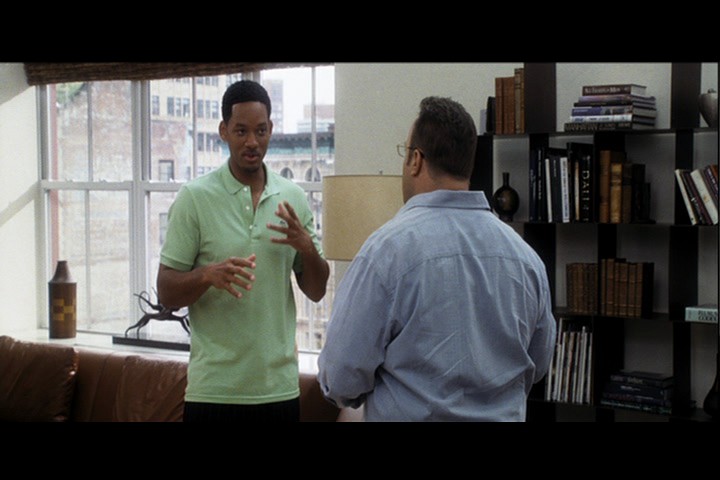

Supplement: S3 Dataset — (ZIP) [file pone.0264302.s003.zip › hitch-00071611.jpg]

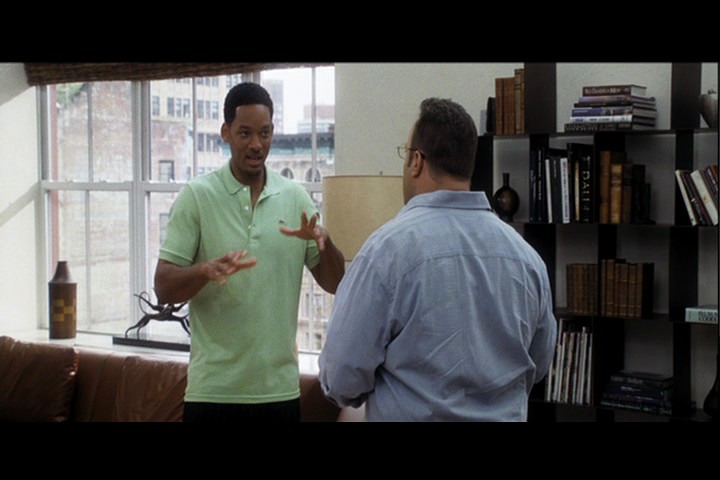

Supplement: S3 Dataset — (ZIP) [file pone.0264302.s003.zip › hitch-00071621.jpg]

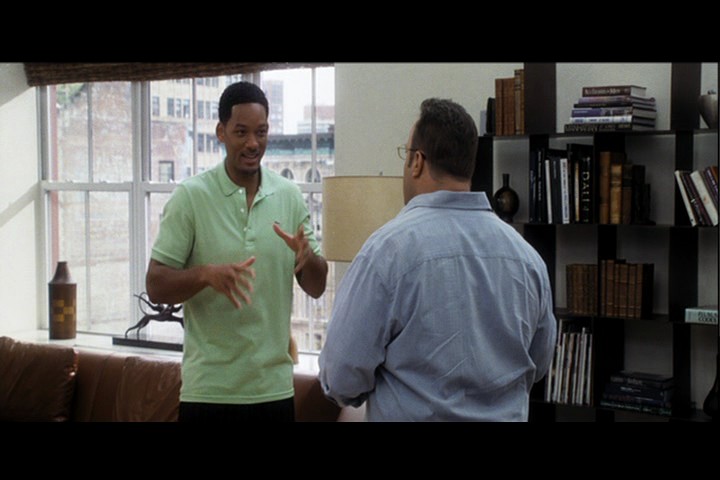

Supplement: S3 Dataset — (ZIP) [file pone.0264302.s003.zip › hitch-00071631.jpg]

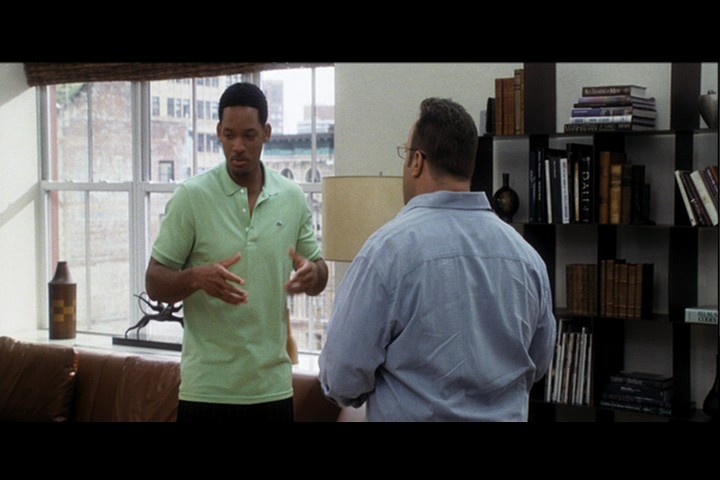

Supplement: S3 Dataset — (ZIP) [file pone.0264302.s003.zip › hitch-00071641.jpg]

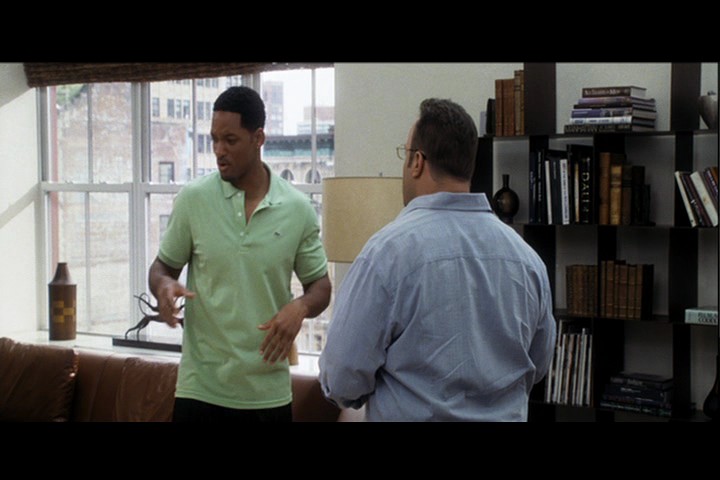

Supplement: S3 Dataset — (ZIP) [file pone.0264302.s003.zip › hitch-00071651.jpg]

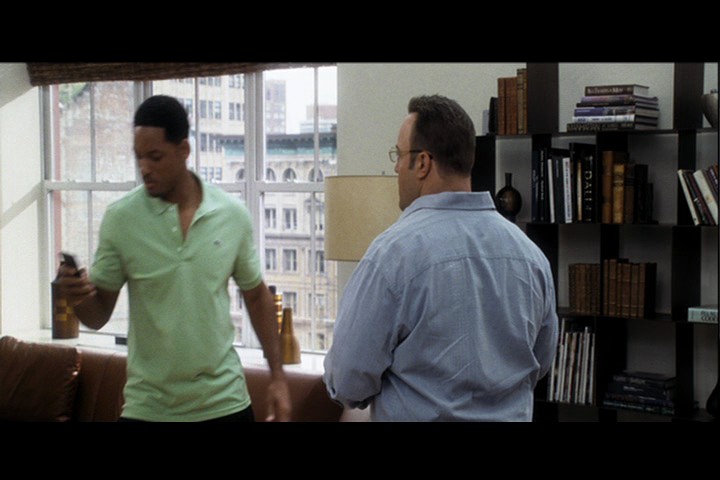

Supplement: S3 Dataset — (ZIP) [file pone.0264302.s003.zip › hitch-00071701.jpg]

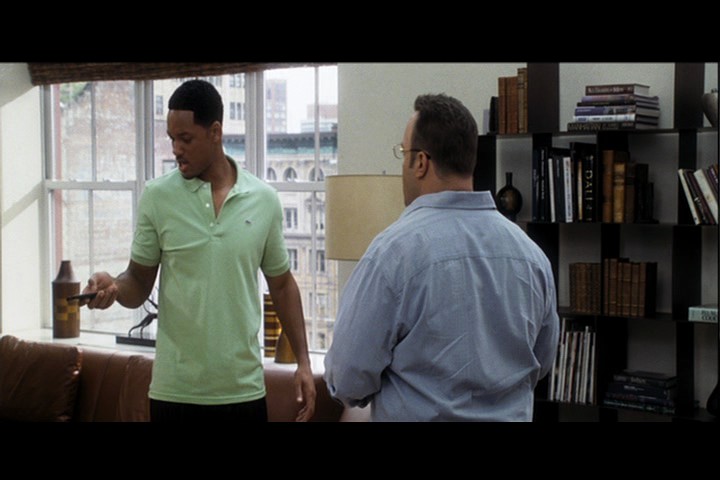

Supplement: S3 Dataset — (ZIP) [file pone.0264302.s003.zip › hitch-00071721.jpg]

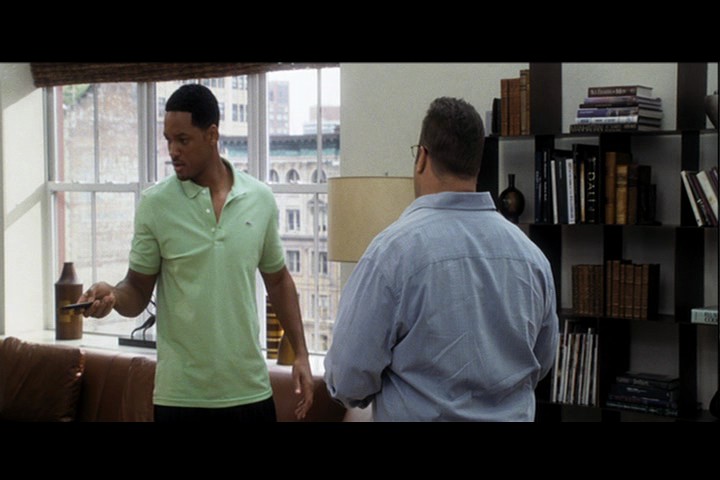

Supplement: S3 Dataset — (ZIP) [file pone.0264302.s003.zip › hitch-00071741.jpg]

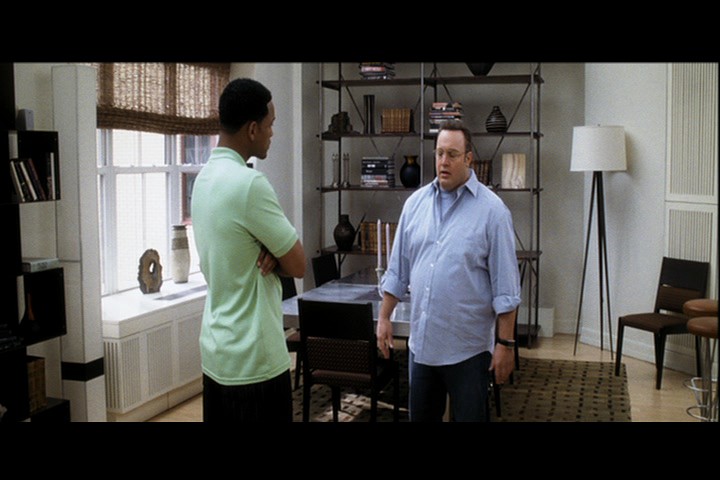

Supplement: S3 Dataset — (ZIP) [file pone.0264302.s003.zip › hitch-00071781.jpg]

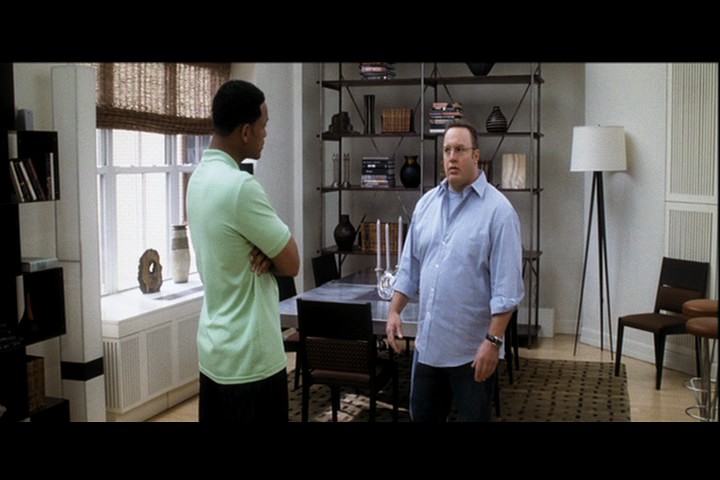

Supplement: S3 Dataset — (ZIP) [file pone.0264302.s003.zip › hitch-00071791.jpg]

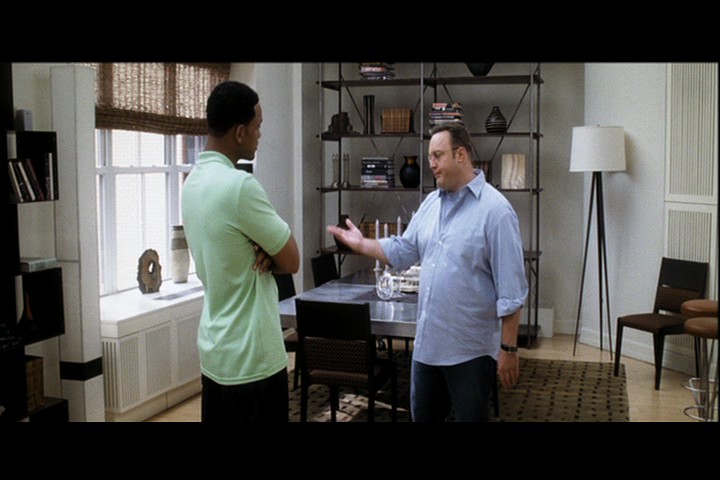

Supplement: S3 Dataset — (ZIP) [file pone.0264302.s003.zip › hitch-00071801.jpg]

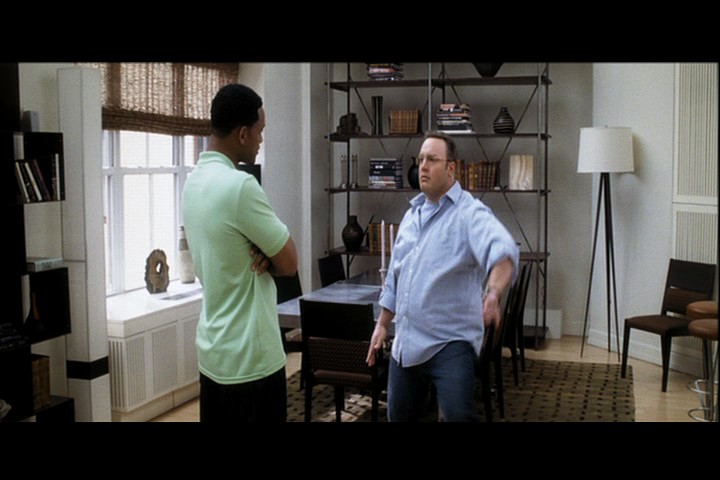

Supplement: S3 Dataset — (ZIP) [file pone.0264302.s003.zip › hitch-00071851.jpg]

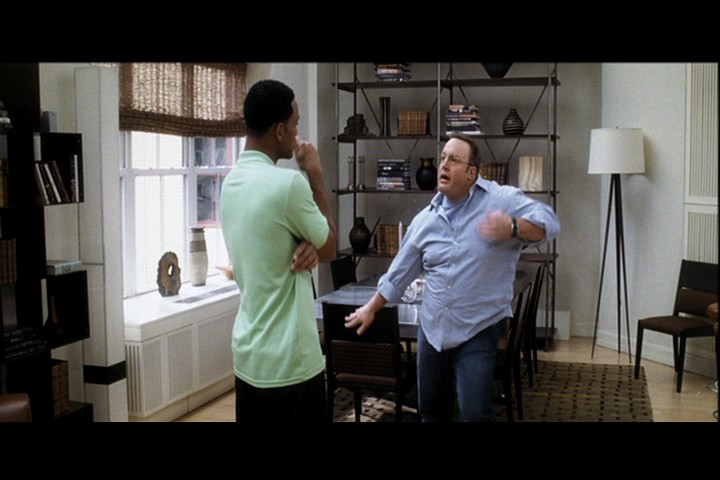

Supplement: S3 Dataset — (ZIP) [file pone.0264302.s003.zip › hitch-00071901.jpg]

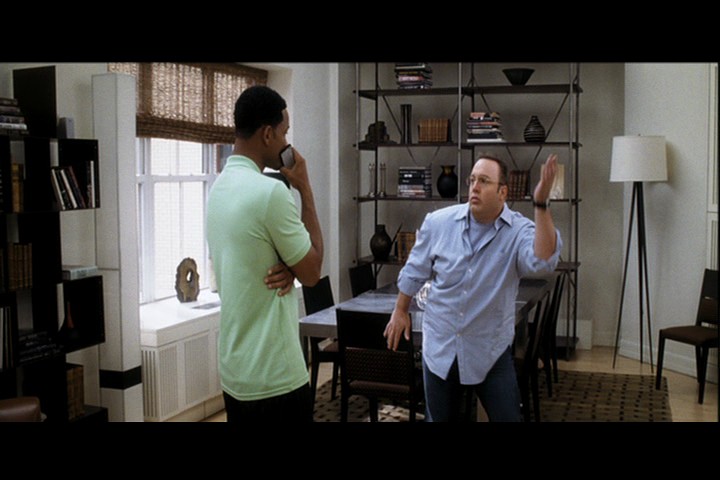

Supplement: S3 Dataset — (ZIP) [file pone.0264302.s003.zip › hitch-00072001.jpg]

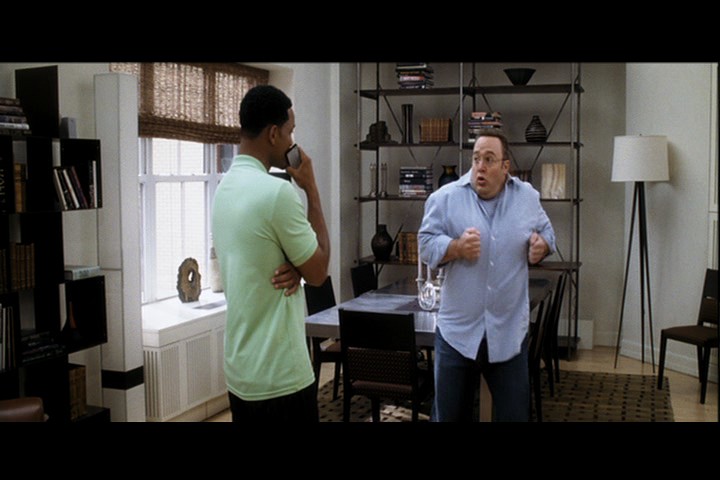

Supplement: S3 Dataset — (ZIP) [file pone.0264302.s003.zip › hitch-00072011.jpg]

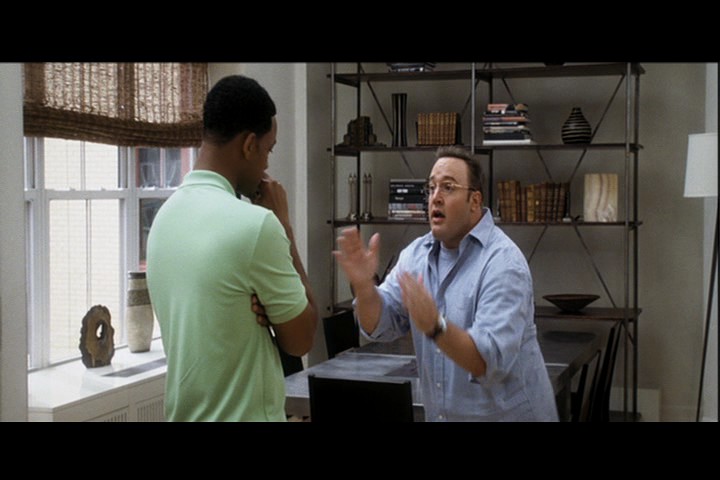

Supplement: S3 Dataset — (ZIP) [file pone.0264302.s003.zip › hitch-00072161.jpg]

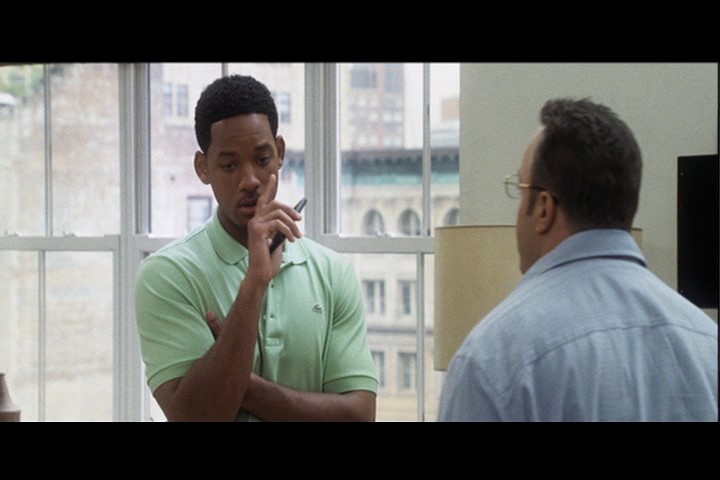

Supplement: S3 Dataset — (ZIP) [file pone.0264302.s003.zip › hitch-00072751.jpg]

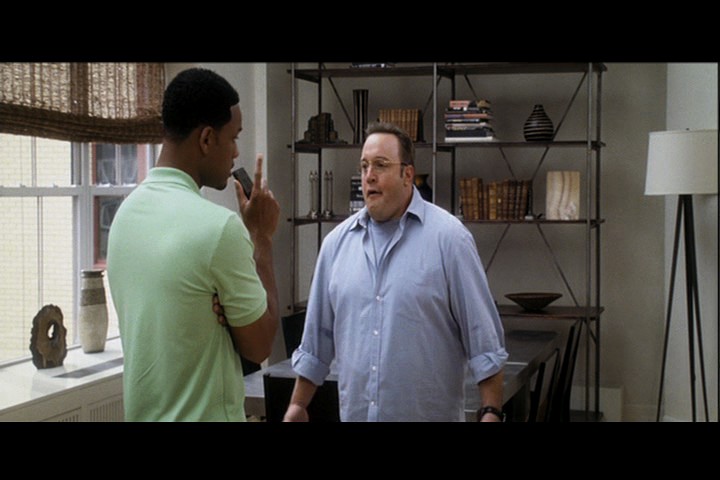

Supplement: S3 Dataset — (ZIP) [file pone.0264302.s003.zip › hitch-00072911.jpg]

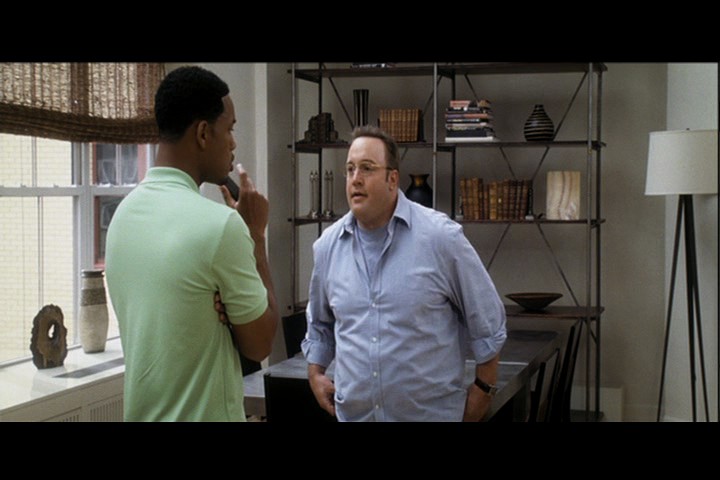

Supplement: S3 Dataset — (ZIP) [file pone.0264302.s003.zip › hitch-00072981.jpg]

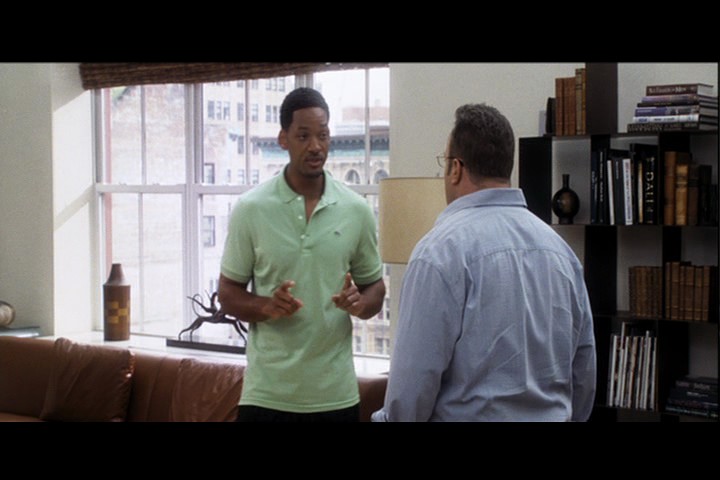

Supplement: S3 Dataset — (ZIP) [file pone.0264302.s003.zip › hitch-00073211.jpg]

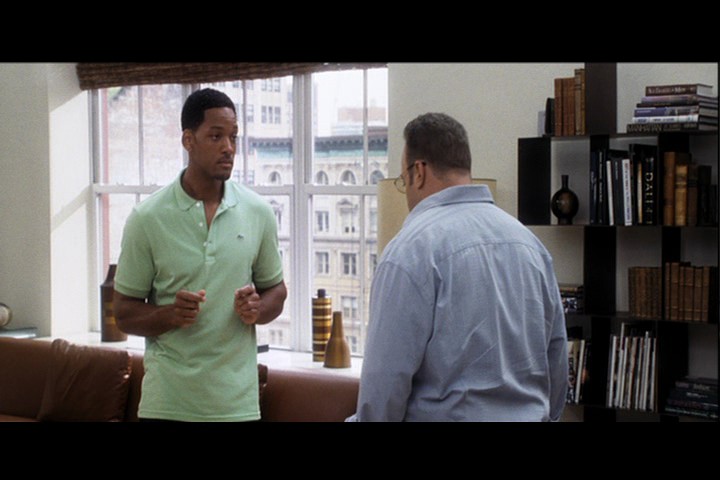

Supplement: S3 Dataset — (ZIP) [file pone.0264302.s003.zip › hitch-00073231.jpg]

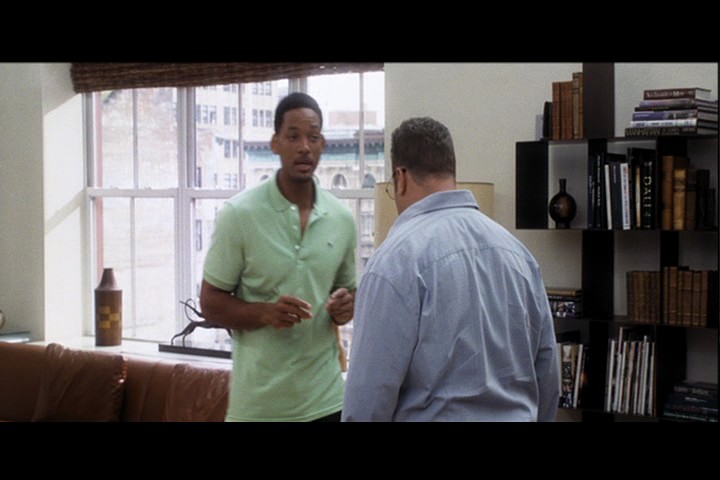

Supplement: S3 Dataset — (ZIP) [file pone.0264302.s003.zip › hitch-00073251.jpg]

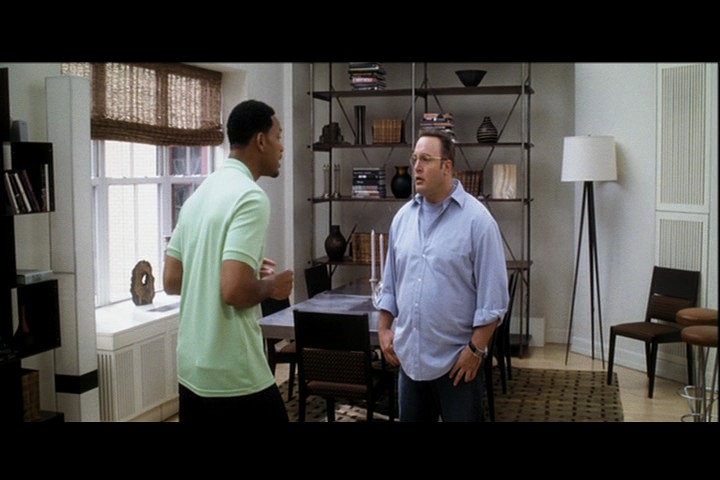

Supplement: S3 Dataset — (ZIP) [file pone.0264302.s003.zip › hitch-00073311.jpg]

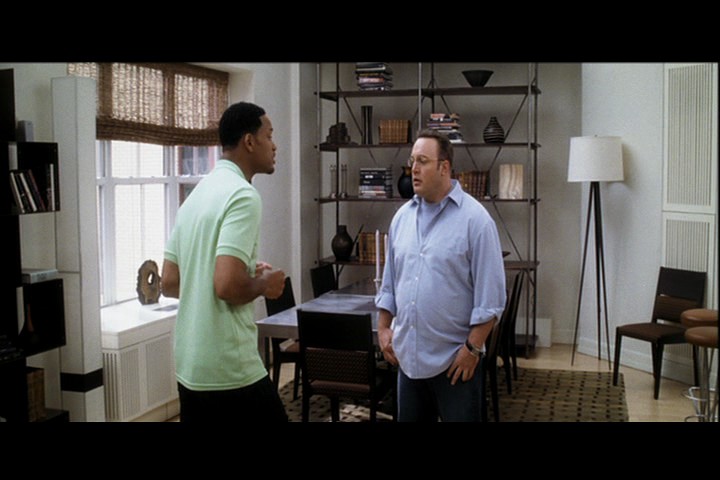

Supplement: S3 Dataset — (ZIP) [file pone.0264302.s003.zip › hitch-00073321.jpg]

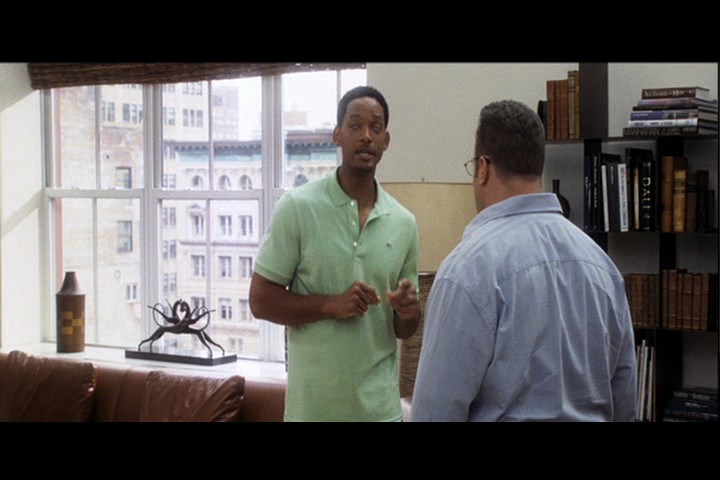

Supplement: S3 Dataset — (ZIP) [file pone.0264302.s003.zip › hitch-00073371.jpg]

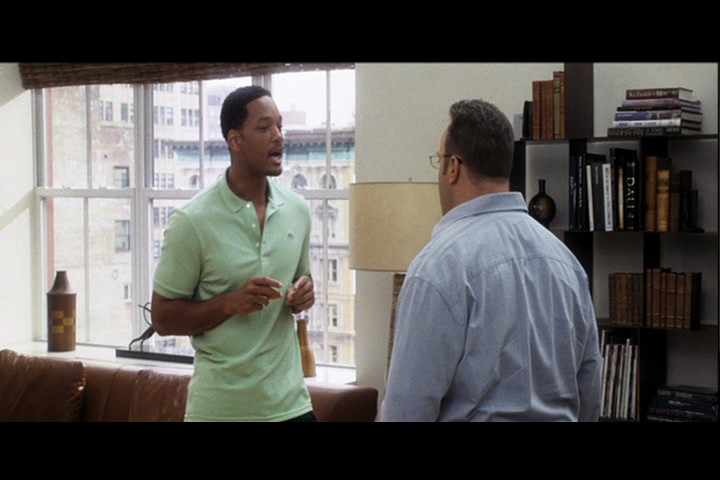

Supplement: S3 Dataset — (ZIP) [file pone.0264302.s003.zip › hitch-00073421.jpg]

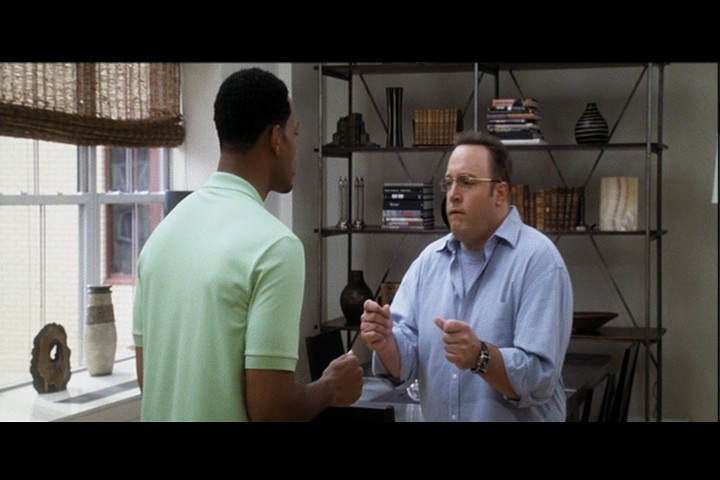

Supplement: S3 Dataset — (ZIP) [file pone.0264302.s003.zip › hitch-00073761.jpg]

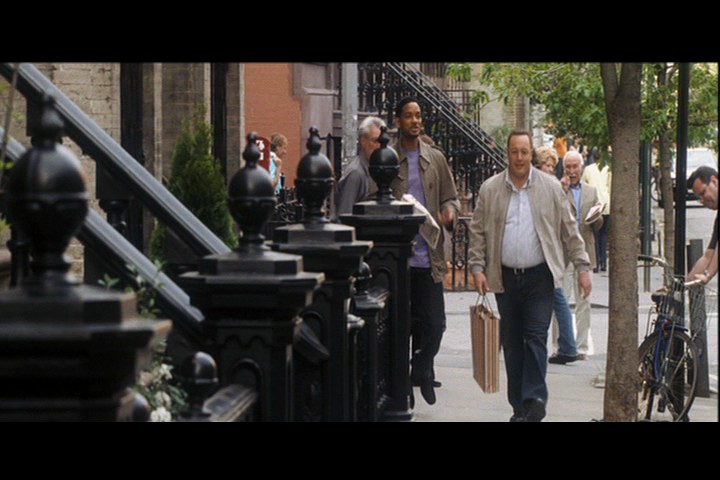

Supplement: S3 Dataset — (ZIP) [file pone.0264302.s003.zip › hitch-00076231.jpg]

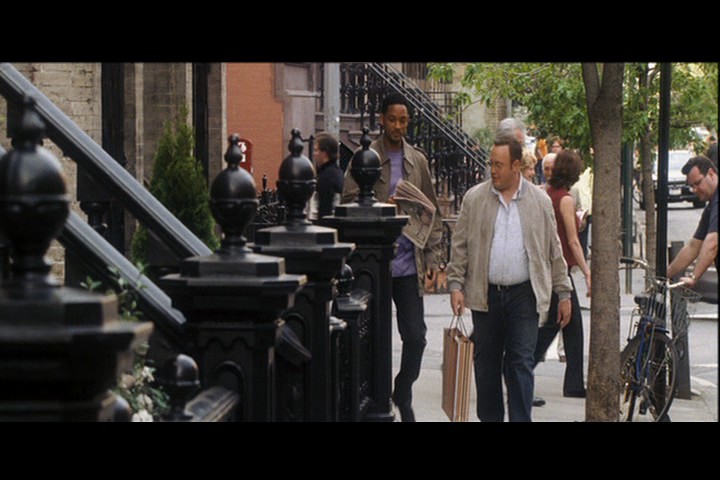

Supplement: S3 Dataset — (ZIP) [file pone.0264302.s003.zip › hitch-00076261.jpg]

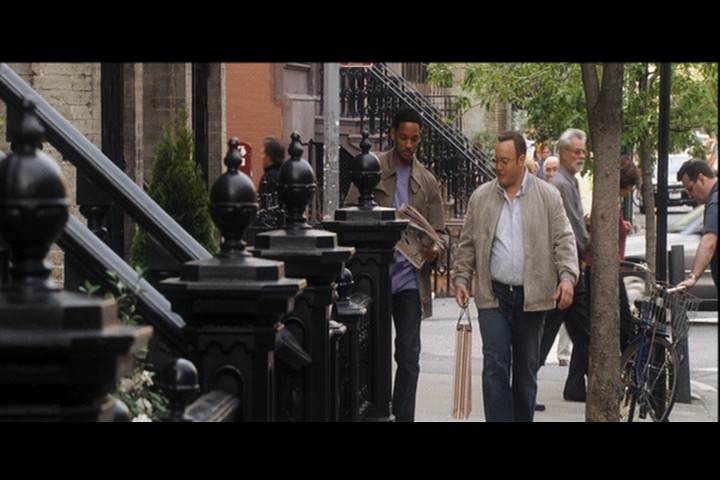

Supplement: S3 Dataset — (ZIP) [file pone.0264302.s003.zip › hitch-00076271.jpg]

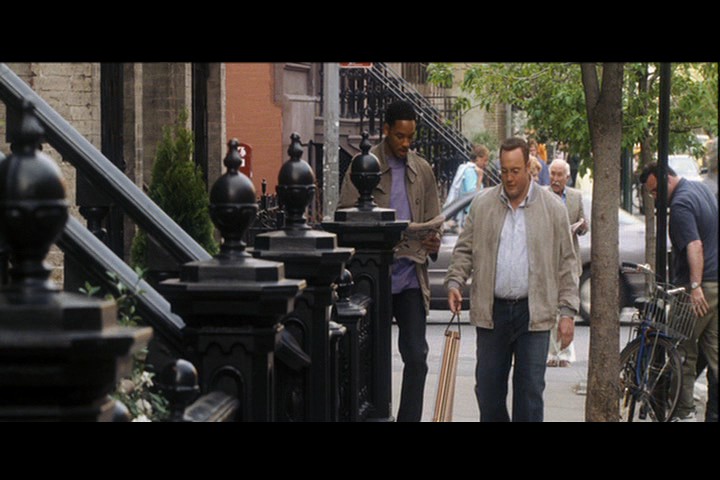

Supplement: S3 Dataset — (ZIP) [file pone.0264302.s003.zip › hitch-00076291.jpg]

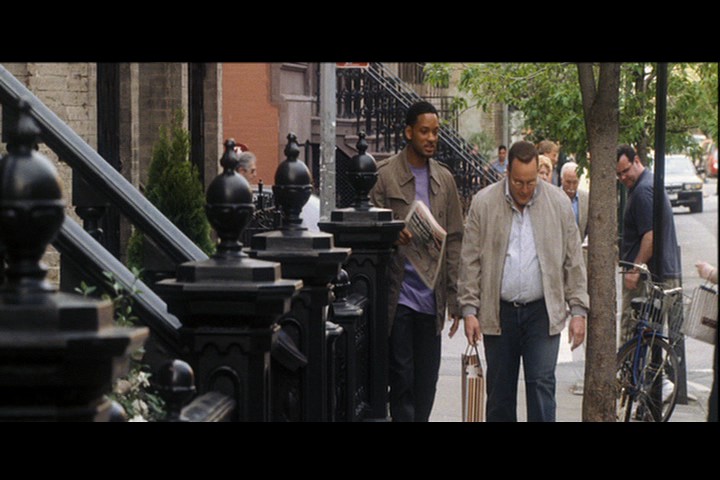

Supplement: S3 Dataset — (ZIP) [file pone.0264302.s003.zip › hitch-00076311.jpg]

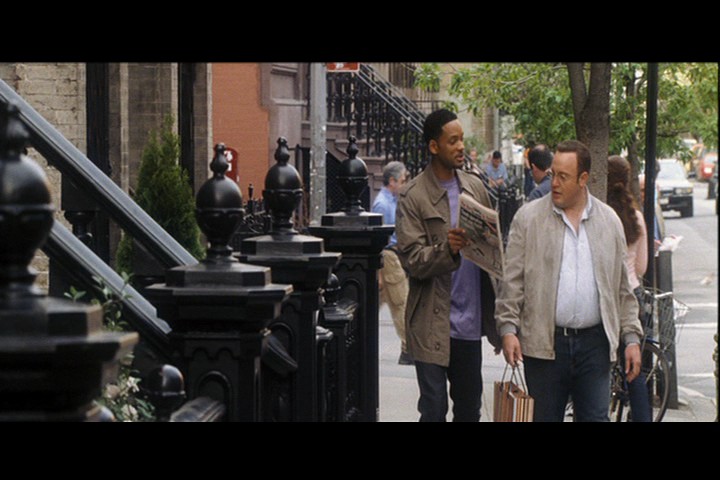

Supplement: S3 Dataset — (ZIP) [file pone.0264302.s003.zip › hitch-00076341.jpg]

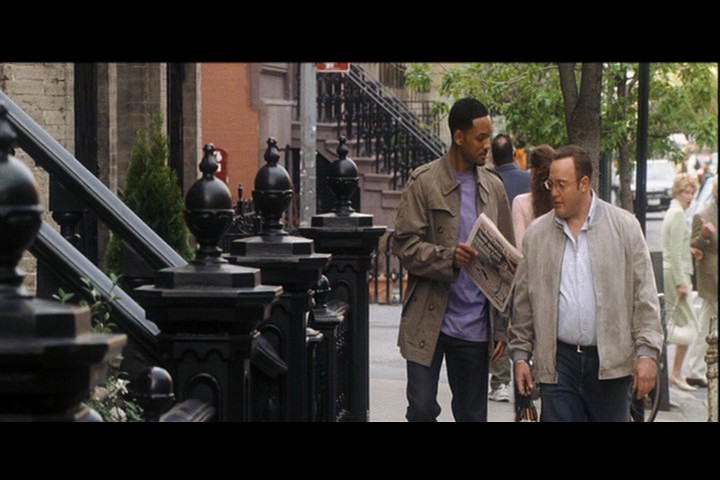

Supplement: S3 Dataset — (ZIP) [file pone.0264302.s003.zip › hitch-00076361.jpg]

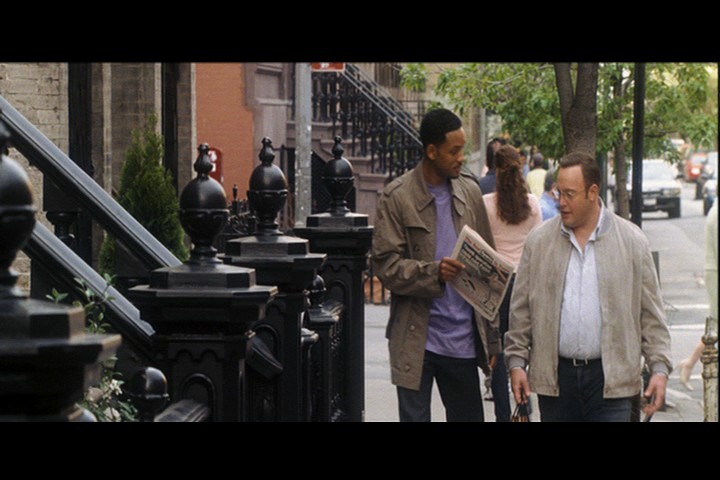

Supplement: S3 Dataset — (ZIP) [file pone.0264302.s003.zip › hitch-00076371.jpg]

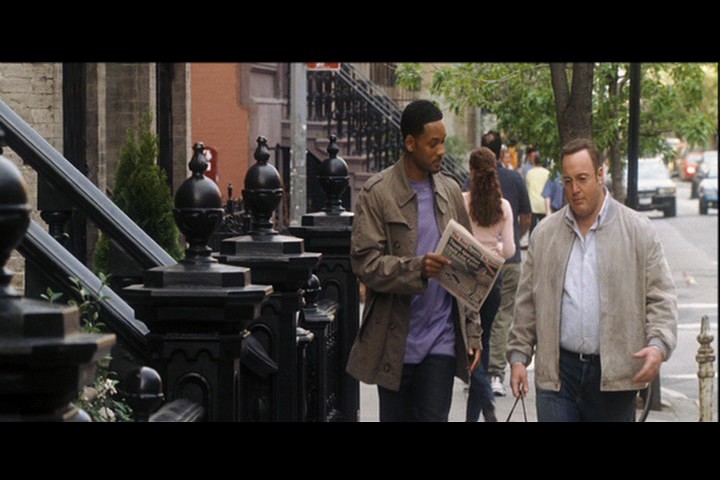

Supplement: S3 Dataset — (ZIP) [file pone.0264302.s003.zip › hitch-00076381.jpg]

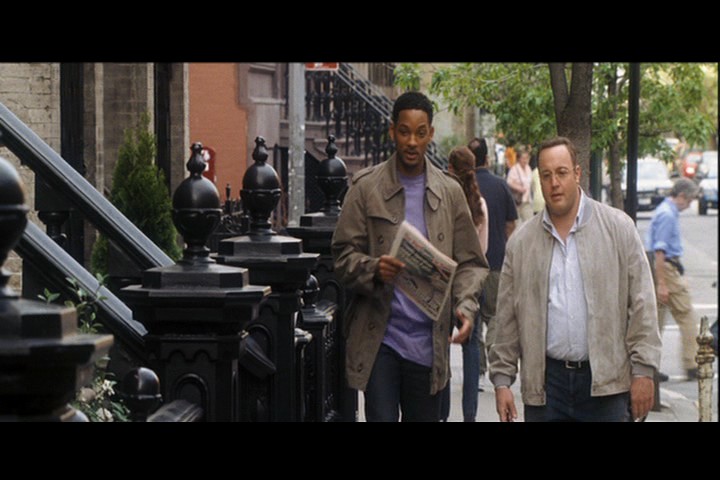

Supplement: S3 Dataset — (ZIP) [file pone.0264302.s003.zip › hitch-00076391.jpg]

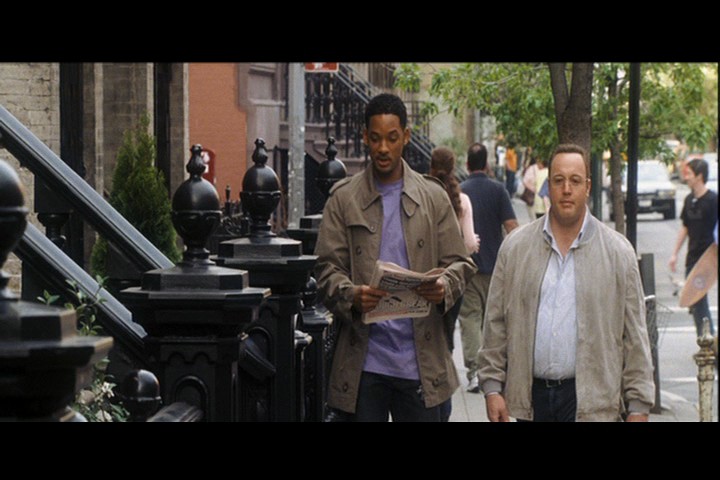

Supplement: S3 Dataset — (ZIP) [file pone.0264302.s003.zip › hitch-00076401.jpg]

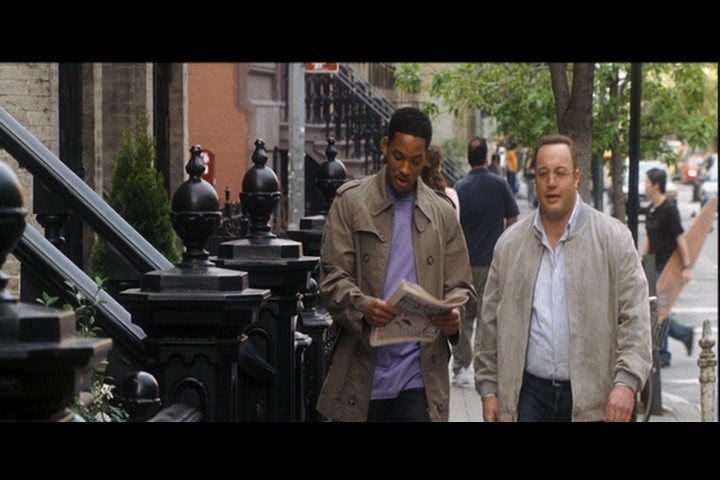

Supplement: S3 Dataset — (ZIP) [file pone.0264302.s003.zip › hitch-00076411.jpg]

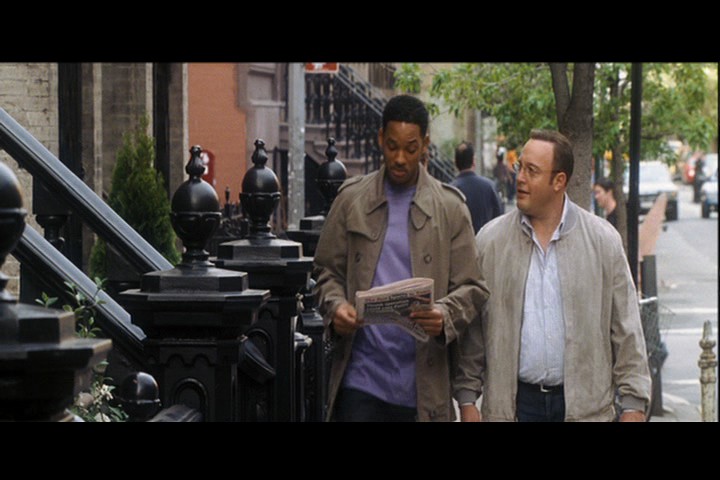

Supplement: S3 Dataset — (ZIP) [file pone.0264302.s003.zip › hitch-00076421.jpg]

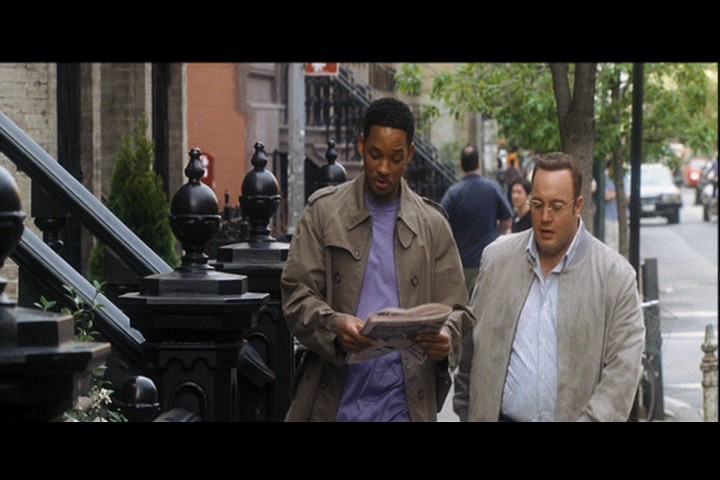

Supplement: S3 Dataset — (ZIP) [file pone.0264302.s003.zip › hitch-00076441.jpg]

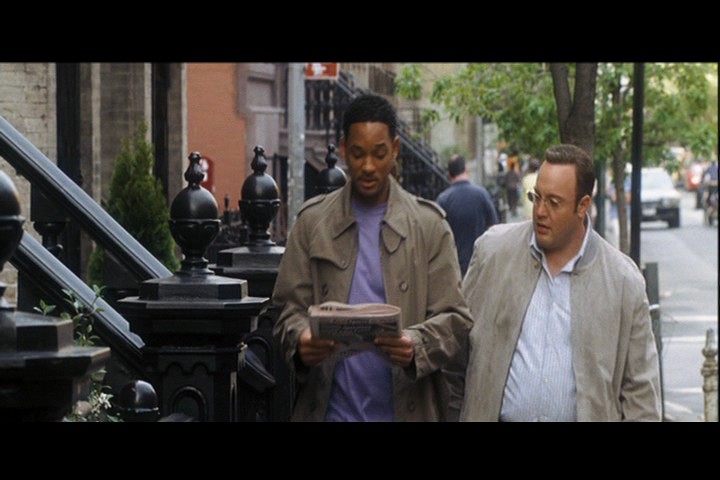

Supplement: S3 Dataset — (ZIP) [file pone.0264302.s003.zip › hitch-00076451.jpg]

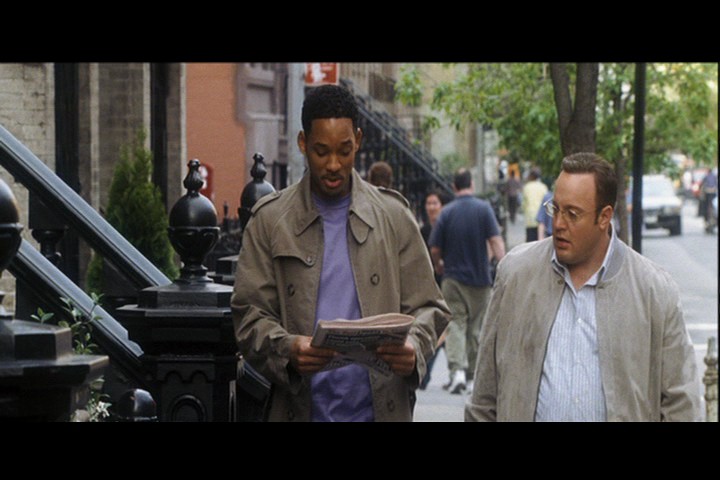

Supplement: S3 Dataset — (ZIP) [file pone.0264302.s003.zip › hitch-00076461.jpg]

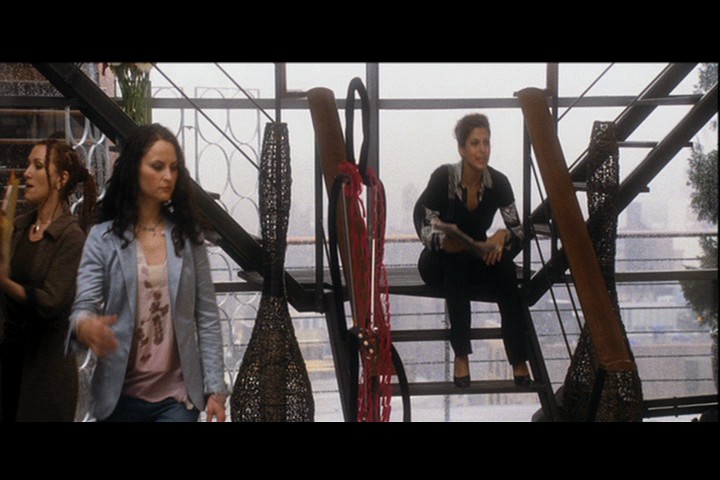

Supplement: S3 Dataset — (ZIP) [file pone.0264302.s003.zip › hitch-00082421.jpg]

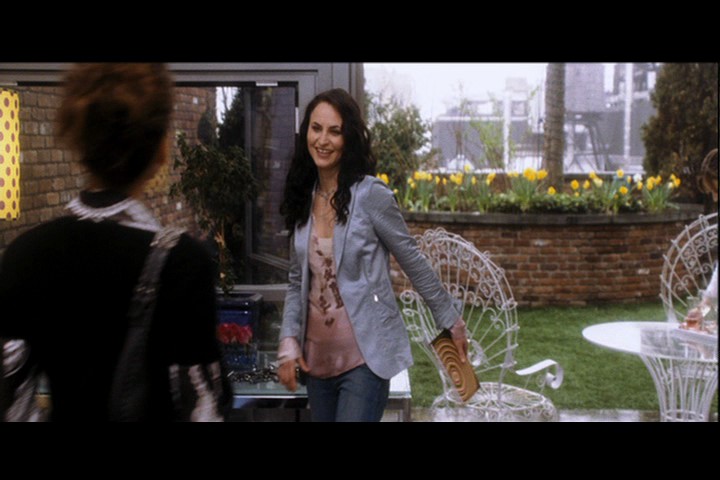

Supplement: S3 Dataset — (ZIP) [file pone.0264302.s003.zip › hitch-00082561.jpg]

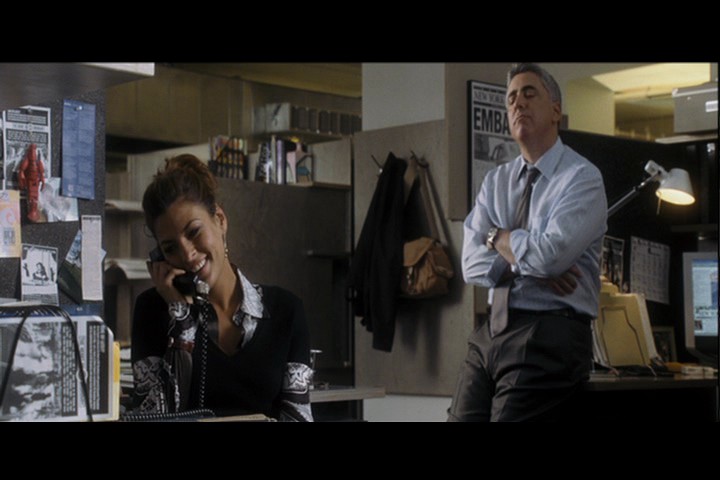

Supplement: S3 Dataset — (ZIP) [file pone.0264302.s003.zip › hitch-00083381.jpg]

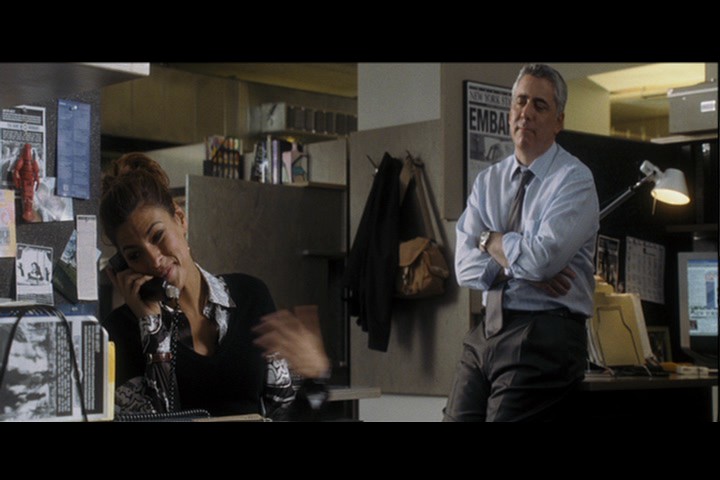

Supplement: S3 Dataset — (ZIP) [file pone.0264302.s003.zip › hitch-00083711.jpg]

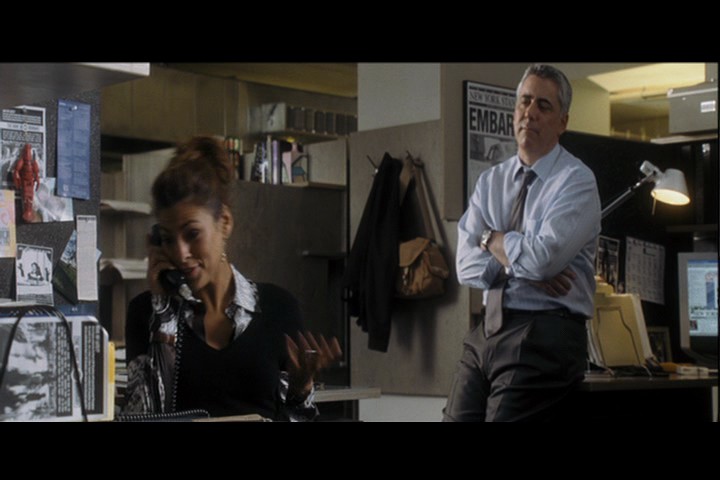

Supplement: S3 Dataset — (ZIP) [file pone.0264302.s003.zip › hitch-00083721.jpg]

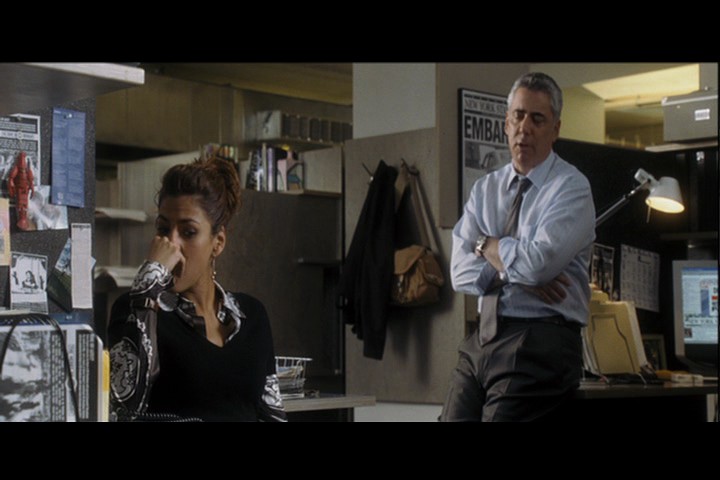

Supplement: S3 Dataset — (ZIP) [file pone.0264302.s003.zip › hitch-00083981.jpg]

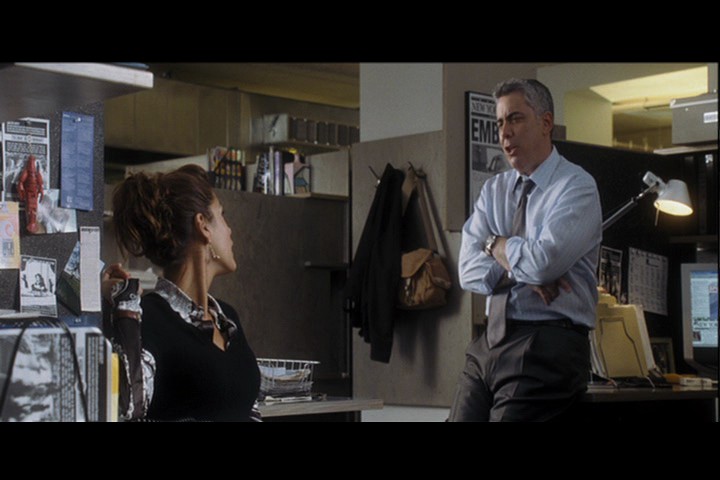

Supplement: S3 Dataset — (ZIP) [file pone.0264302.s003.zip › hitch-00084451.jpg]

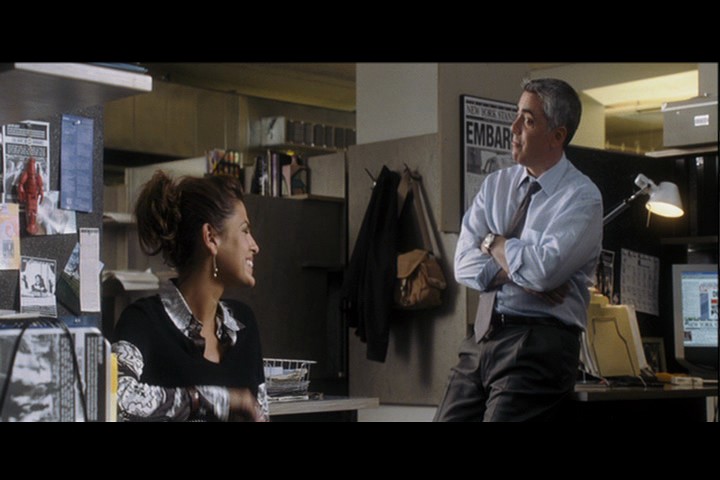

Supplement: S3 Dataset — (ZIP) [file pone.0264302.s003.zip › hitch-00084631.jpg]

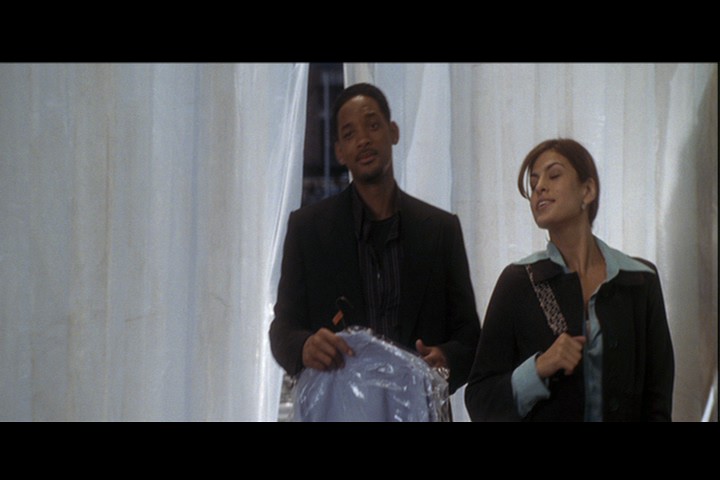

Supplement: S3 Dataset — (ZIP) [file pone.0264302.s003.zip › hitch-00085721.jpg]

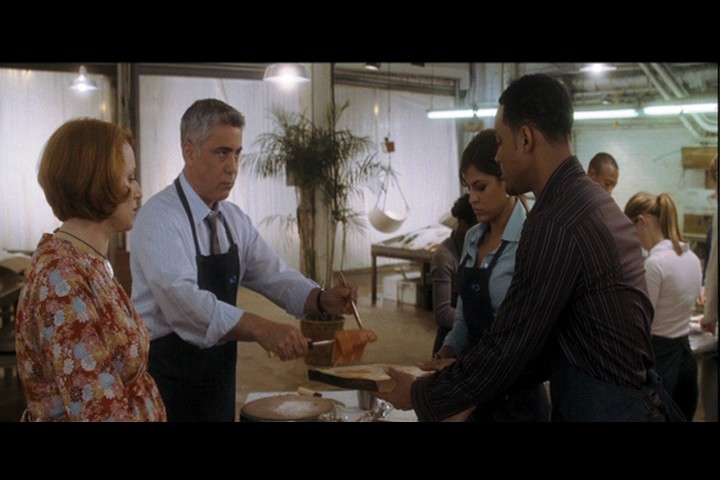

Supplement: S3 Dataset — (ZIP) [file pone.0264302.s003.zip › hitch-00088911.jpg]

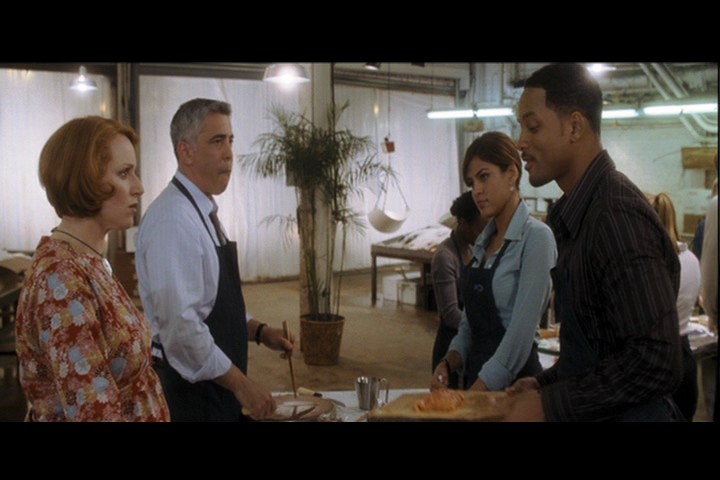

Supplement: S3 Dataset — (ZIP) [file pone.0264302.s003.zip › hitch-00088971.jpg]

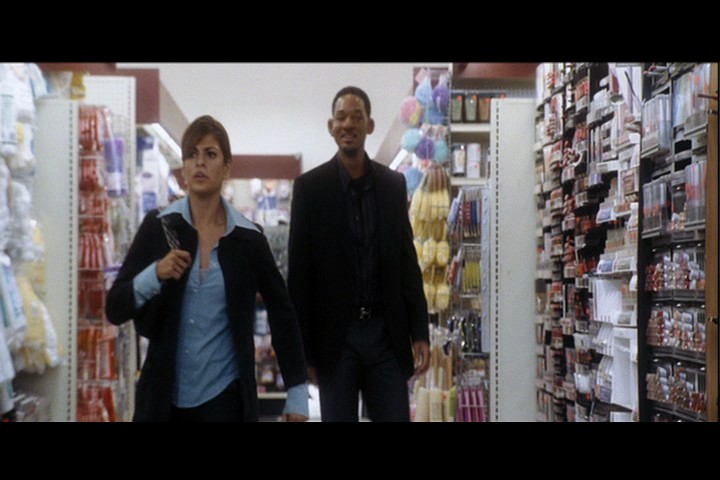

Supplement: S3 Dataset — (ZIP) [file pone.0264302.s003.zip › hitch-00090701.jpg]

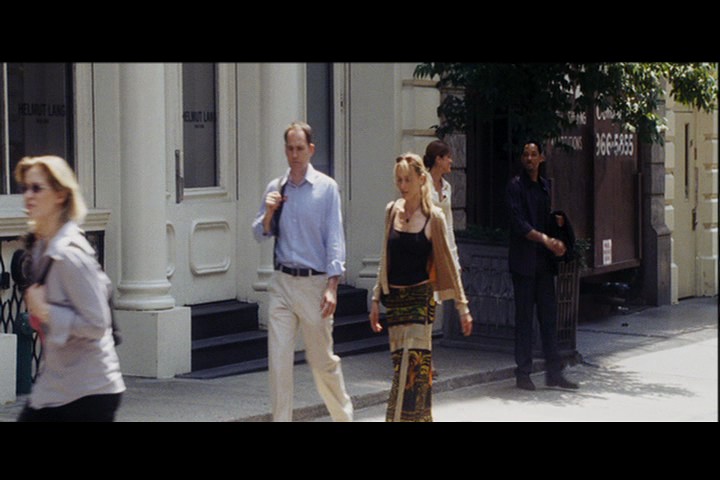

Supplement: S3 Dataset — (ZIP) [file pone.0264302.s003.zip › hitch-00101931.jpg]

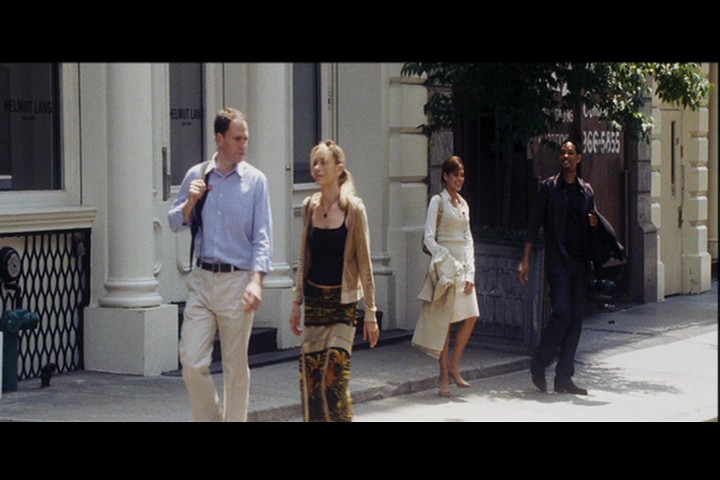

Supplement: S3 Dataset — (ZIP) [file pone.0264302.s003.zip › hitch-00101951.jpg]

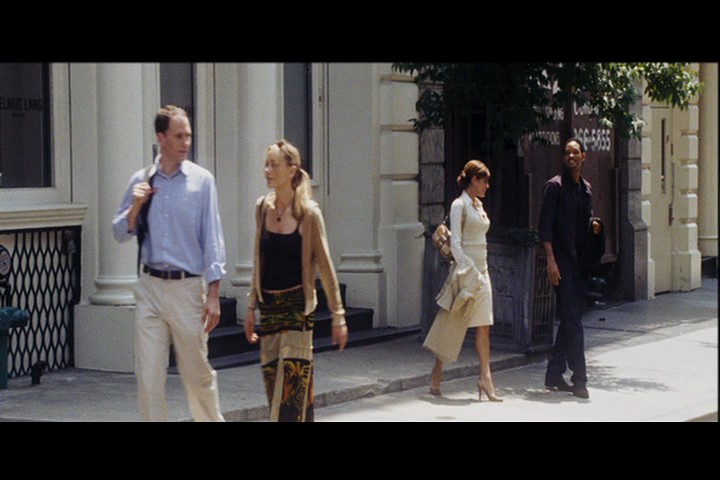

Supplement: S3 Dataset — (ZIP) [file pone.0264302.s003.zip › hitch-00101961.jpg]

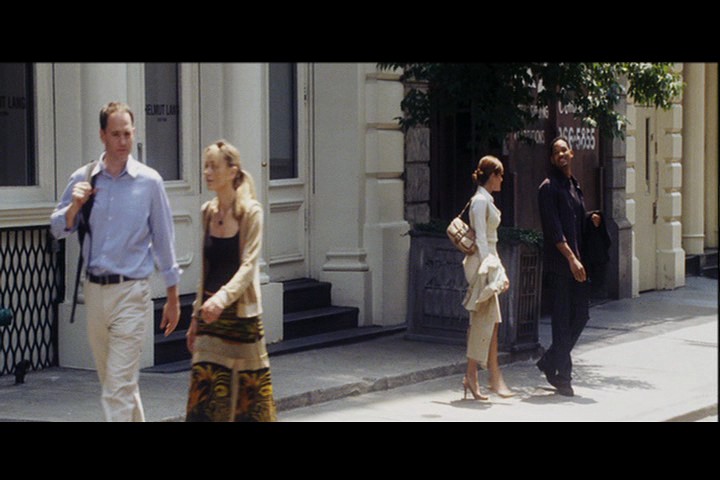

Supplement: S3 Dataset — (ZIP) [file pone.0264302.s003.zip › hitch-00101971.jpg]

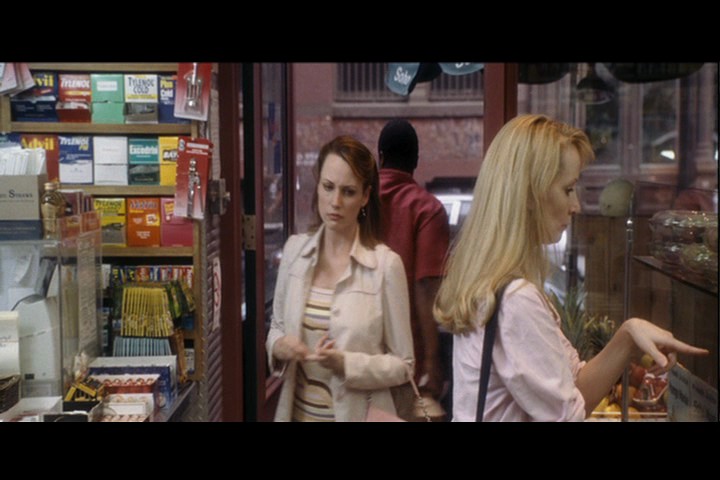

Supplement: S3 Dataset — (ZIP) [file pone.0264302.s003.zip › hitch-00104261.jpg]

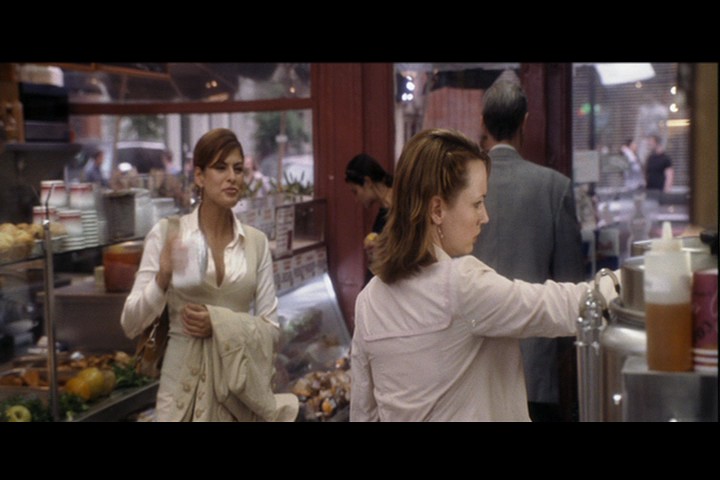

Supplement: S3 Dataset — (ZIP) [file pone.0264302.s003.zip › hitch-00104361.jpg]

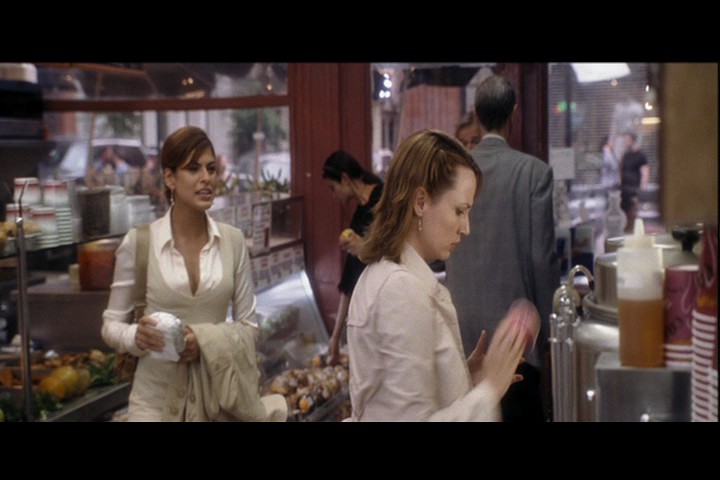

Supplement: S3 Dataset — (ZIP) [file pone.0264302.s003.zip › hitch-00104371.jpg]

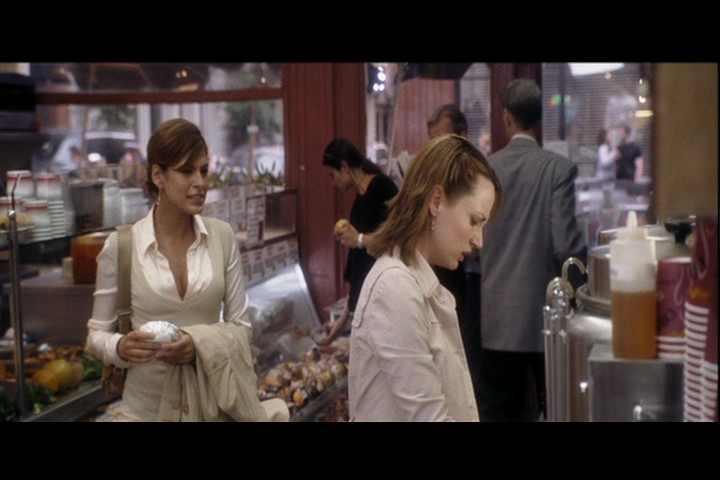

Supplement: S3 Dataset — (ZIP) [file pone.0264302.s003.zip › hitch-00104381.jpg]

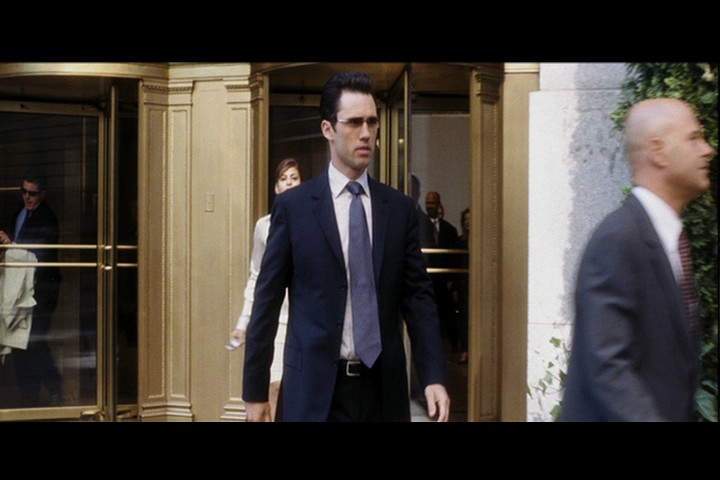

Supplement: S3 Dataset — (ZIP) [file pone.0264302.s003.zip › hitch-00106531.jpg]

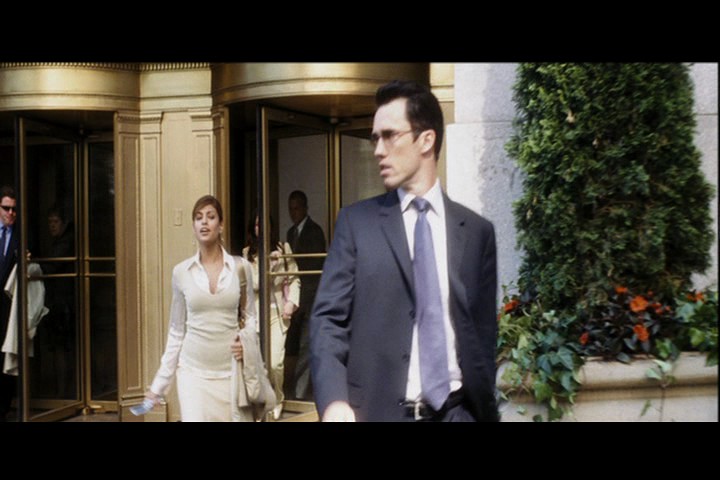

Supplement: S3 Dataset — (ZIP) [file pone.0264302.s003.zip › hitch-00106551.jpg]

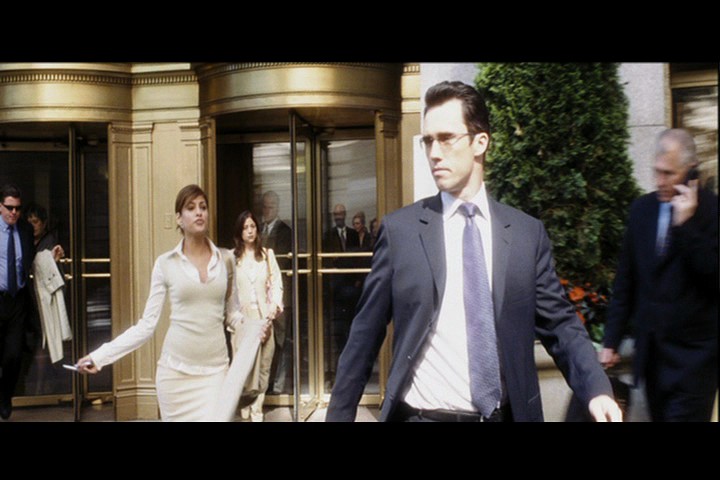

Supplement: S3 Dataset — (ZIP) [file pone.0264302.s003.zip › hitch-00106561.jpg]

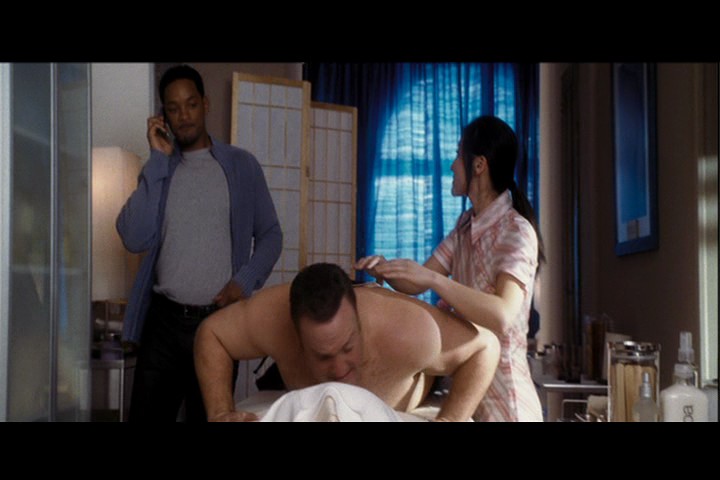

Supplement: S3 Dataset — (ZIP) [file pone.0264302.s003.zip › hitch-00109341.jpg]

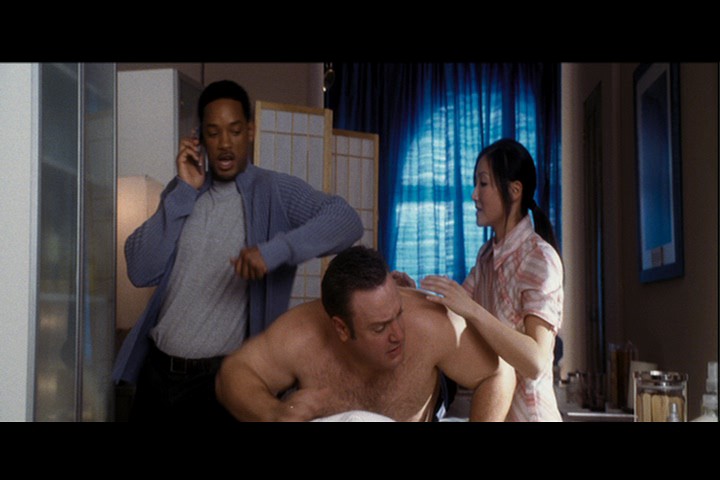

Supplement: S3 Dataset — (ZIP) [file pone.0264302.s003.zip › hitch-00109351.jpg]

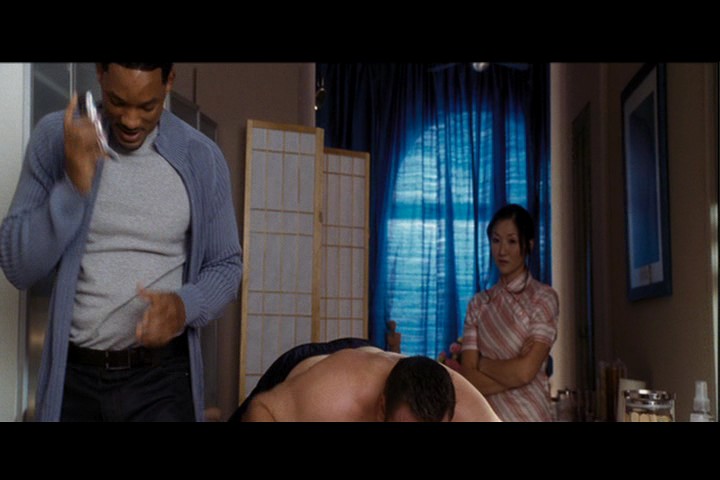

Supplement: S3 Dataset — (ZIP) [file pone.0264302.s003.zip › hitch-00109981.jpg]

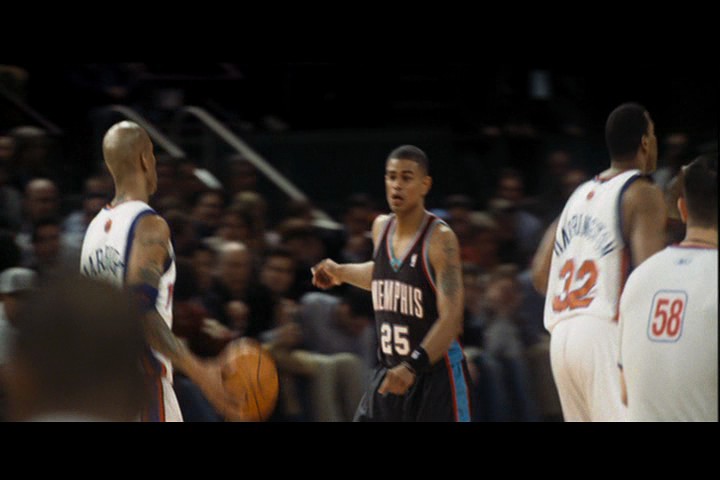

Supplement: S3 Dataset — (ZIP) [file pone.0264302.s003.zip › hitch-00110591.jpg]

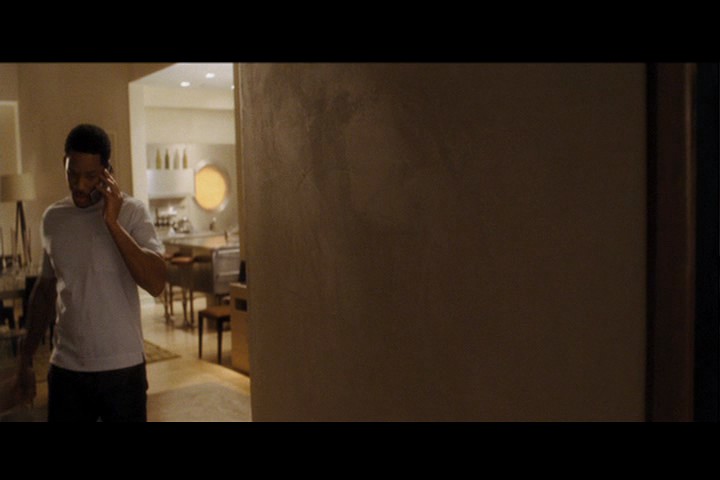

Supplement: S3 Dataset — (ZIP) [file pone.0264302.s003.zip › hitch-00111851.jpg]

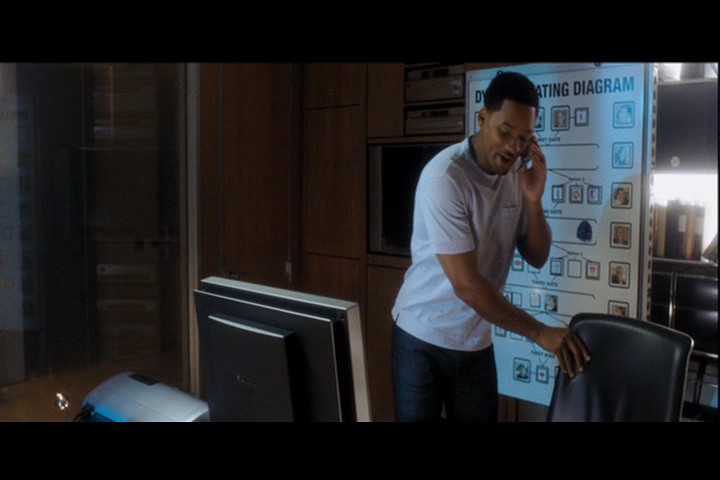

Supplement: S3 Dataset — (ZIP) [file pone.0264302.s003.zip › hitch-00112051.jpg]

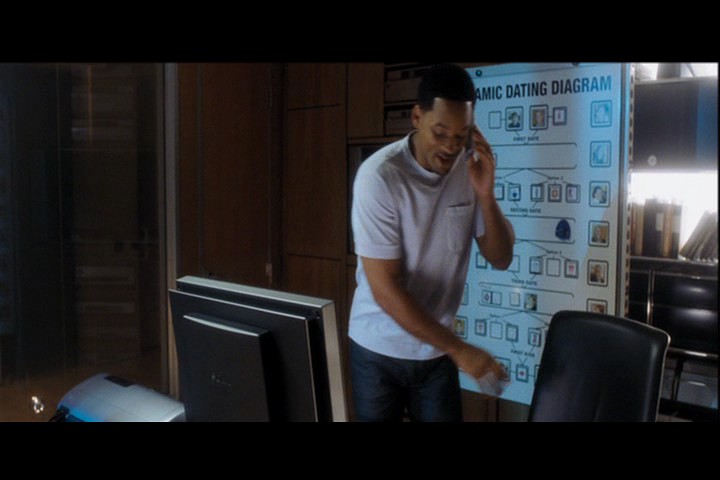

Supplement: S3 Dataset — (ZIP) [file pone.0264302.s003.zip › hitch-00112061.jpg]

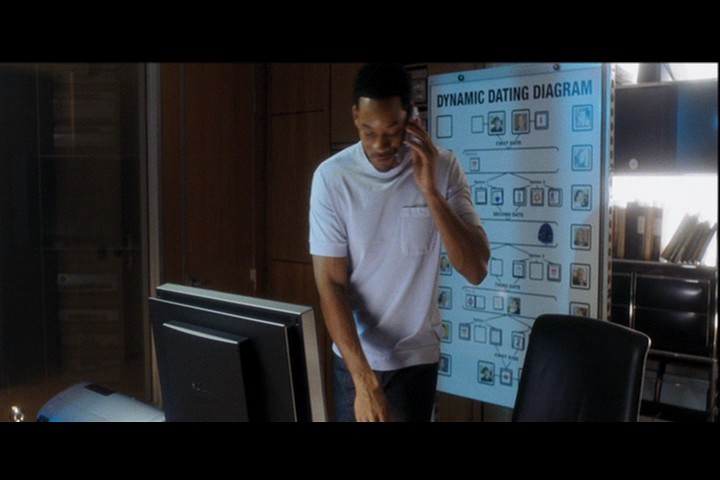

Supplement: S3 Dataset — (ZIP) [file pone.0264302.s003.zip › hitch-00112071.jpg]

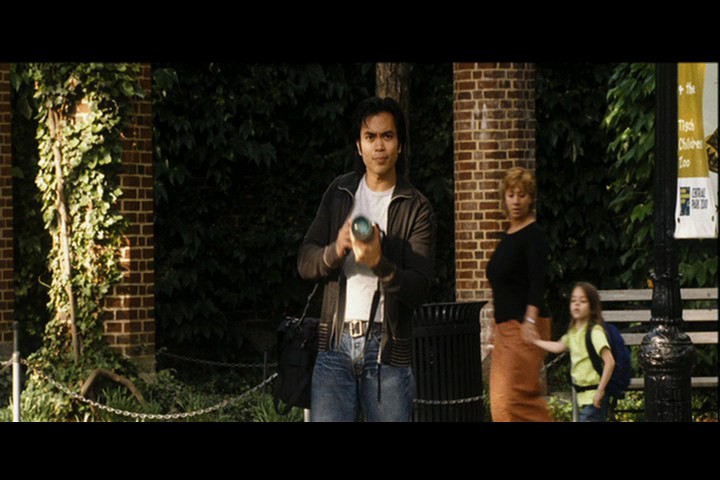

Supplement: S3 Dataset — (ZIP) [file pone.0264302.s003.zip › hitch-00118741.jpg]

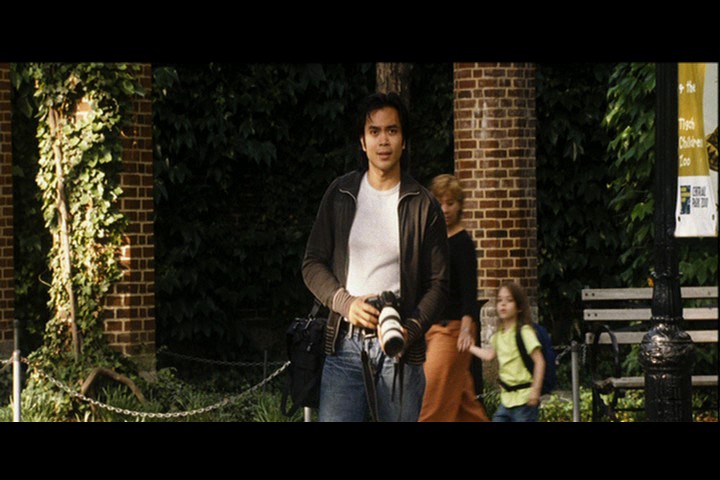

Supplement: S3 Dataset — (ZIP) [file pone.0264302.s003.zip › hitch-00118751.jpg]

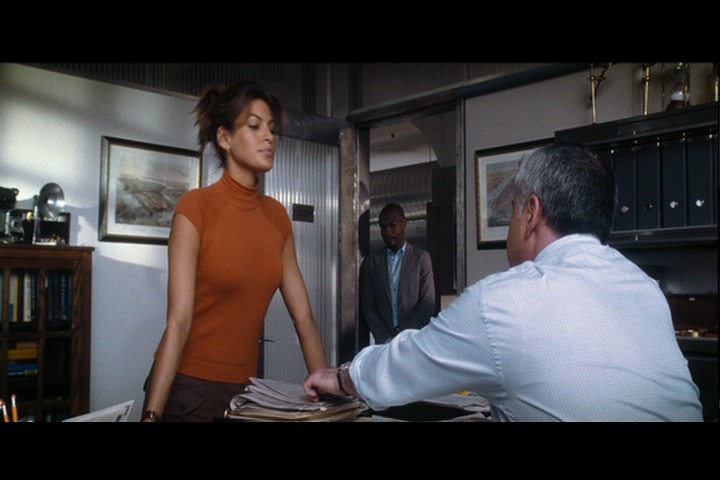

Supplement: S3 Dataset — (ZIP) [file pone.0264302.s003.zip › hitch-00118961.jpg]

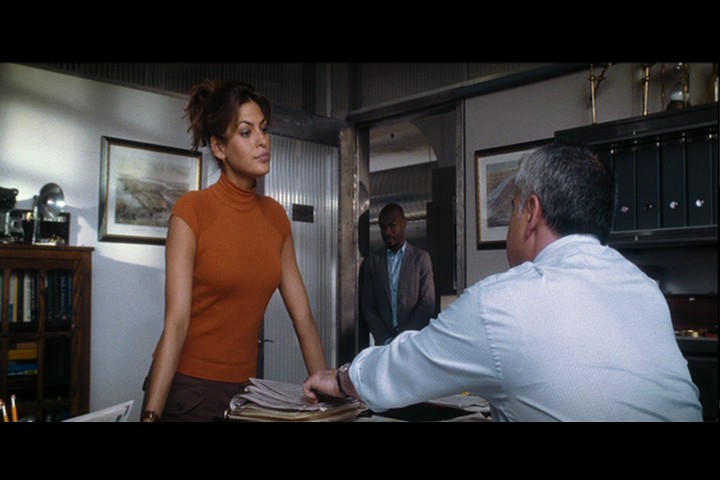

Supplement: S3 Dataset — (ZIP) [file pone.0264302.s003.zip › hitch-00118971.jpg]

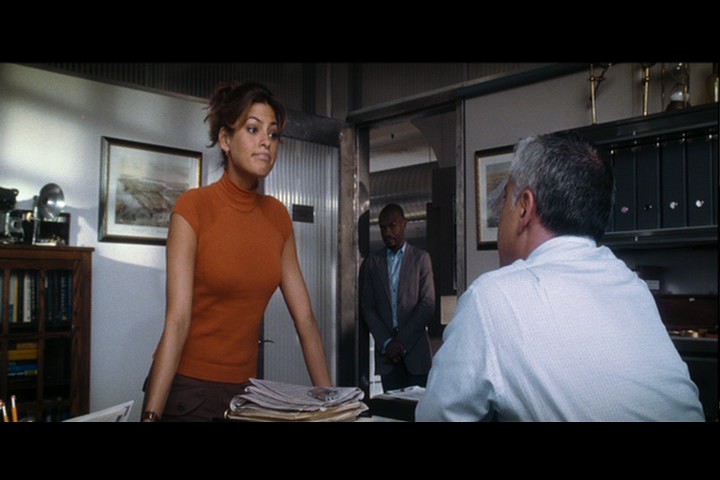

Supplement: S3 Dataset — (ZIP) [file pone.0264302.s003.zip › hitch-00119001.jpg]

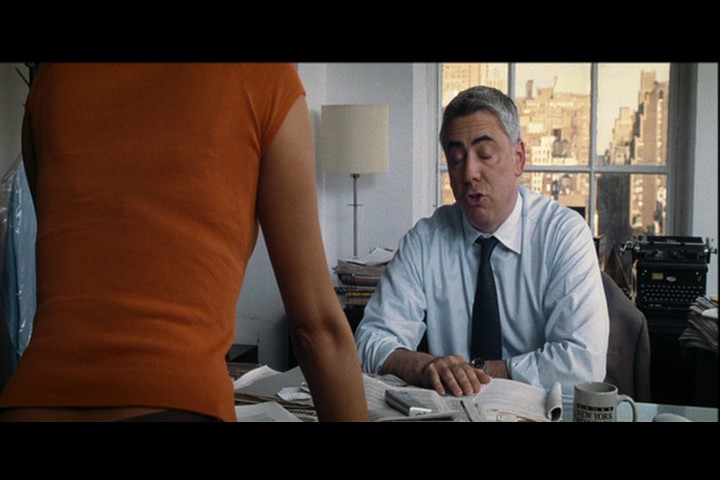

Supplement: S3 Dataset — (ZIP) [file pone.0264302.s003.zip › hitch-00119021.jpg]

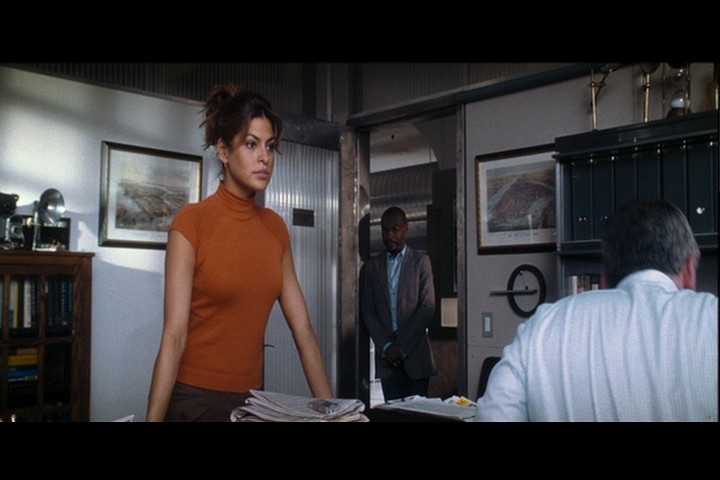

Supplement: S3 Dataset — (ZIP) [file pone.0264302.s003.zip › hitch-00119181.jpg]

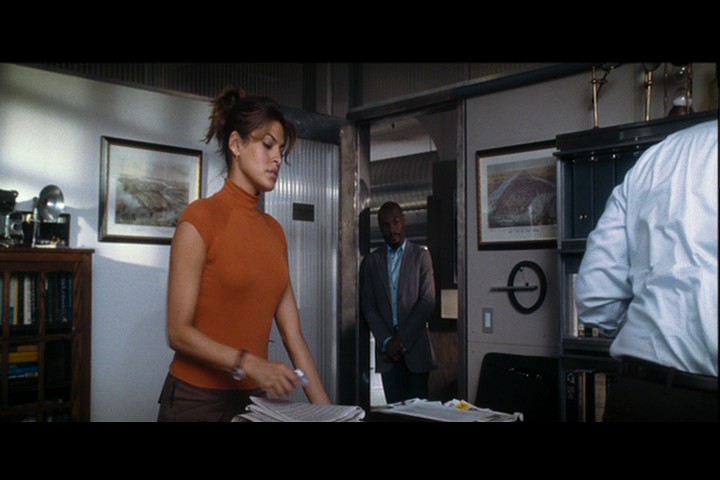

Supplement: S3 Dataset — (ZIP) [file pone.0264302.s003.zip › hitch-00119201.jpg]
