# Supplementary figures and images for: LHPE-nets: A lightweight 2D and 3D human pose estimation model with well-structural deep networks and multi-view pose sample simplification method (part 8 of 8)
Source: PLoS One. 2022 Feb 23;17(2):e0264302. doi: 10.1371/journal.pone.0264302 (PMC8865690; doi:10.1371/journal.pone.0264302)

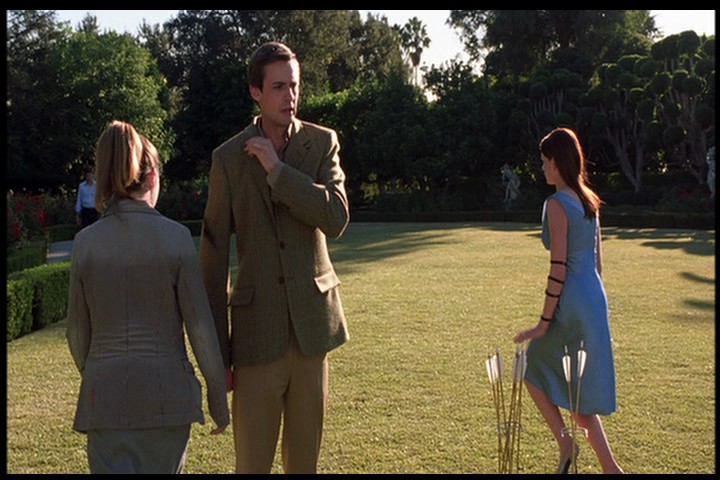

Supplement: S4 Dataset — It also includes pose data and camera parameters. (ZIP) [file pone.0264302.s004.zip › princess-diaries-2-00098791.jpg]

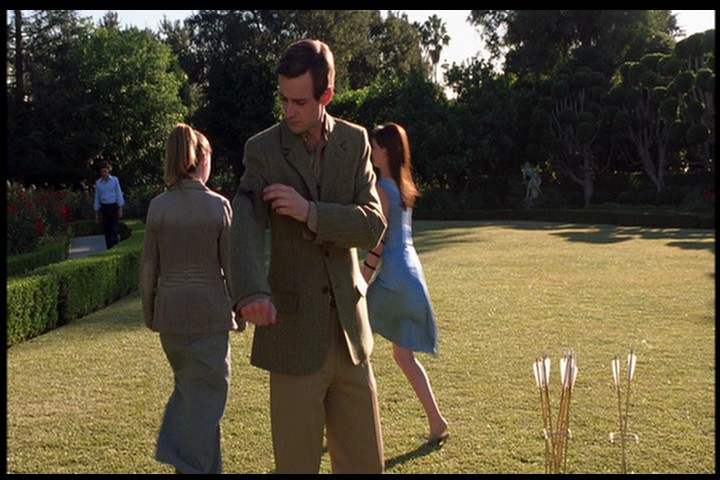

Supplement: S4 Dataset — It also includes pose data and camera parameters. (ZIP) [file pone.0264302.s004.zip › princess-diaries-2-00098821.jpg]

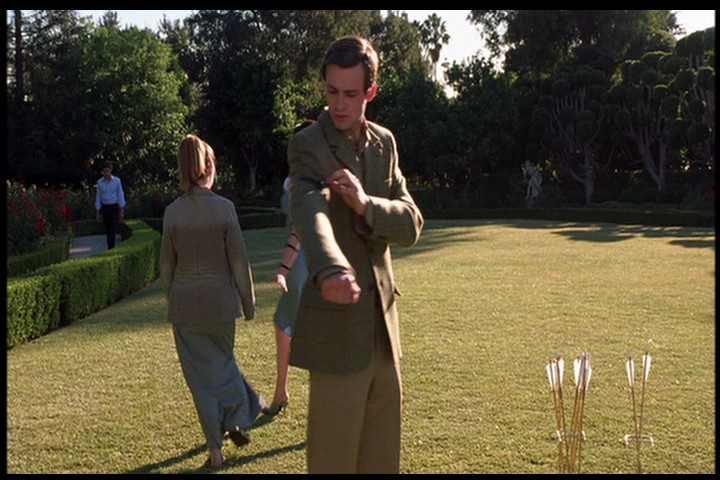

Supplement: S4 Dataset — It also includes pose data and camera parameters. (ZIP) [file pone.0264302.s004.zip › princess-diaries-2-00098831.jpg]

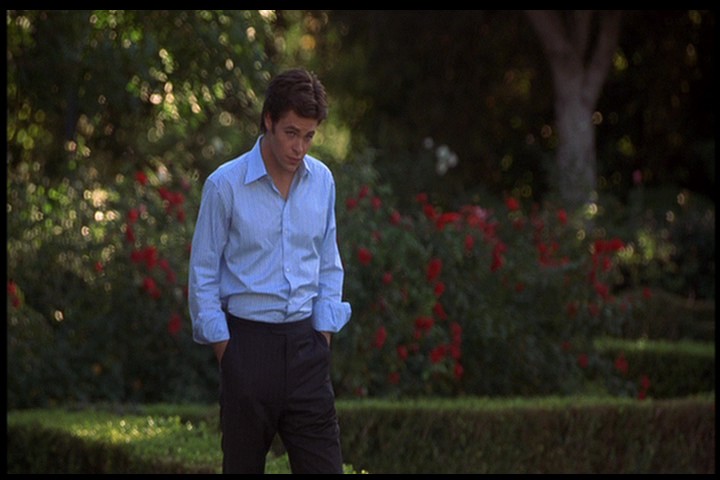

Supplement: S4 Dataset — It also includes pose data and camera parameters. (ZIP) [file pone.0264302.s004.zip › princess-diaries-2-00099101.jpg]

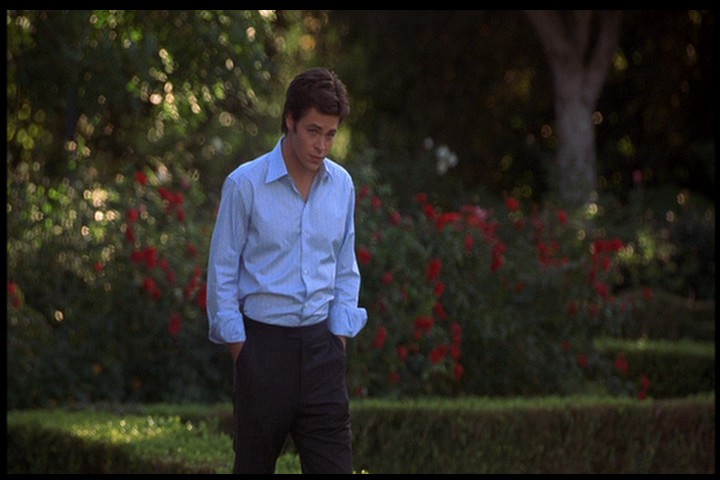

Supplement: S4 Dataset — It also includes pose data and camera parameters. (ZIP) [file pone.0264302.s004.zip › princess-diaries-2-00099111.jpg]

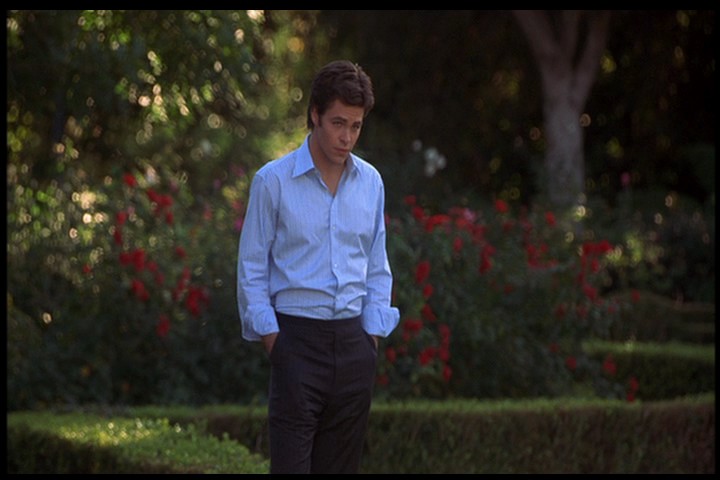

Supplement: S4 Dataset — It also includes pose data and camera parameters. (ZIP) [file pone.0264302.s004.zip › princess-diaries-2-00099141.jpg]

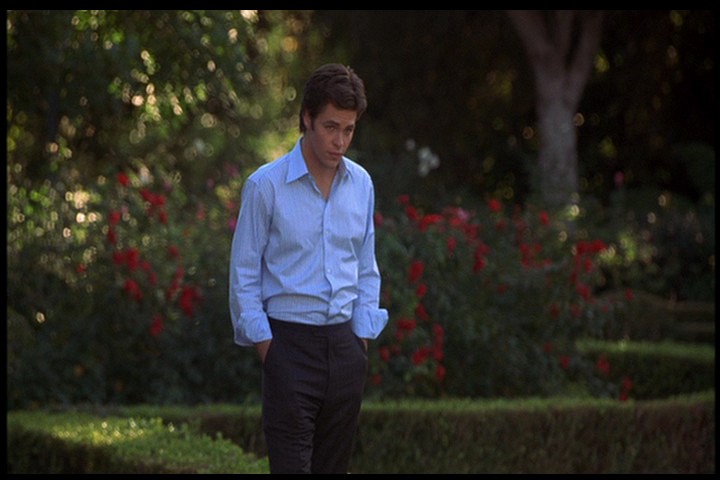

Supplement: S4 Dataset — It also includes pose data and camera parameters. (ZIP) [file pone.0264302.s004.zip › princess-diaries-2-00099151.jpg]

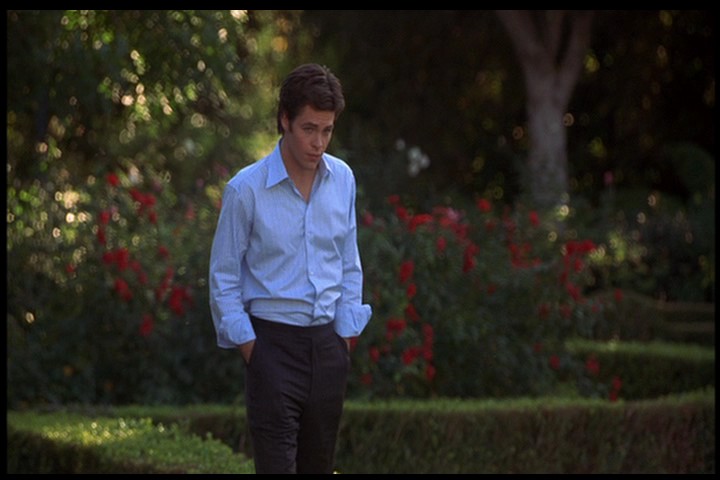

Supplement: S4 Dataset — It also includes pose data and camera parameters. (ZIP) [file pone.0264302.s004.zip › princess-diaries-2-00099161.jpg]

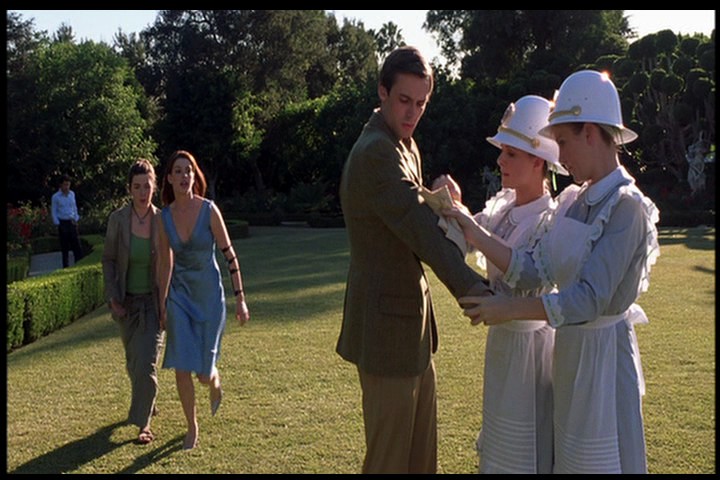

Supplement: S4 Dataset — It also includes pose data and camera parameters. (ZIP) [file pone.0264302.s004.zip › princess-diaries-2-00099251.jpg]

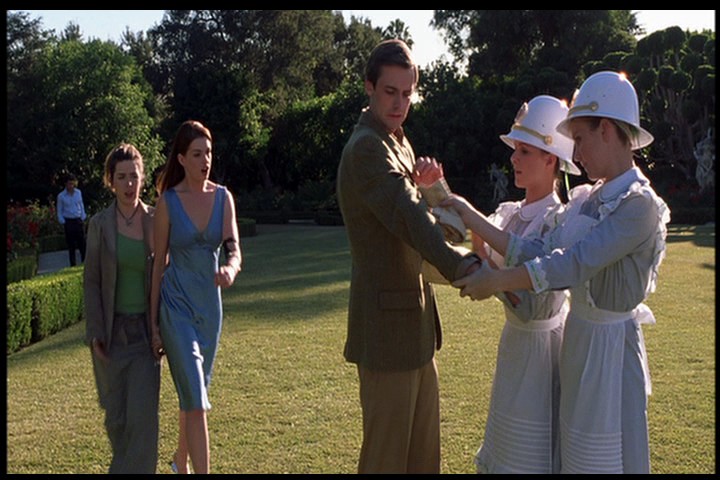

Supplement: S4 Dataset — It also includes pose data and camera parameters. (ZIP) [file pone.0264302.s004.zip › princess-diaries-2-00099271.jpg]

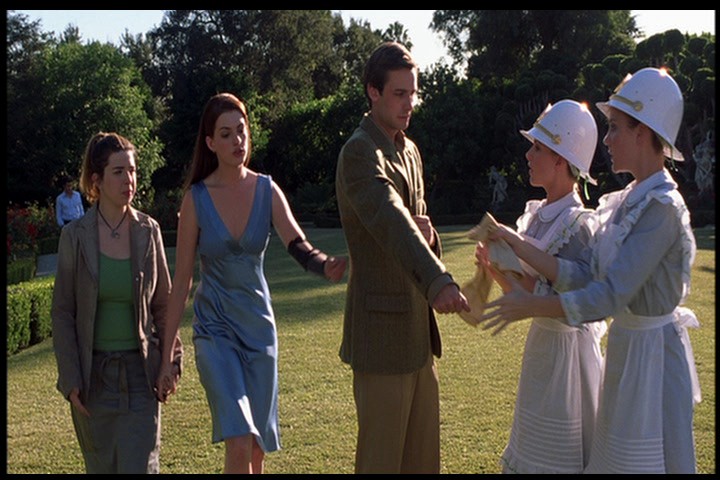

Supplement: S4 Dataset — It also includes pose data and camera parameters. (ZIP) [file pone.0264302.s004.zip › princess-diaries-2-00099291.jpg]

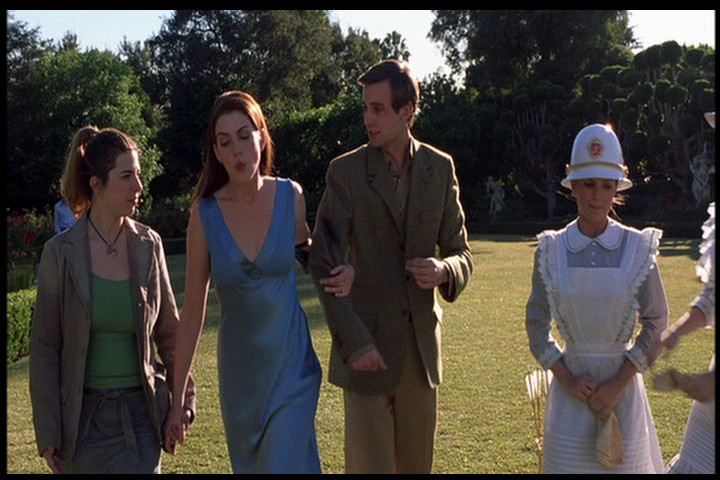

Supplement: S4 Dataset — It also includes pose data and camera parameters. (ZIP) [file pone.0264302.s004.zip › princess-diaries-2-00099311.jpg]

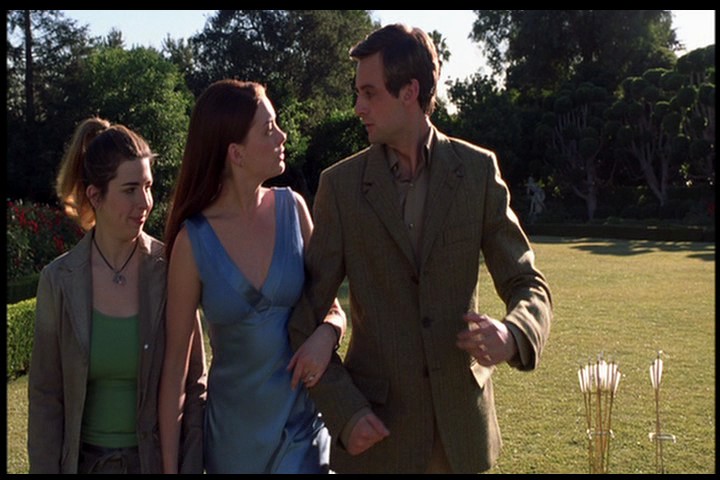

Supplement: S4 Dataset — It also includes pose data and camera parameters. (ZIP) [file pone.0264302.s004.zip › princess-diaries-2-00099341.jpg]

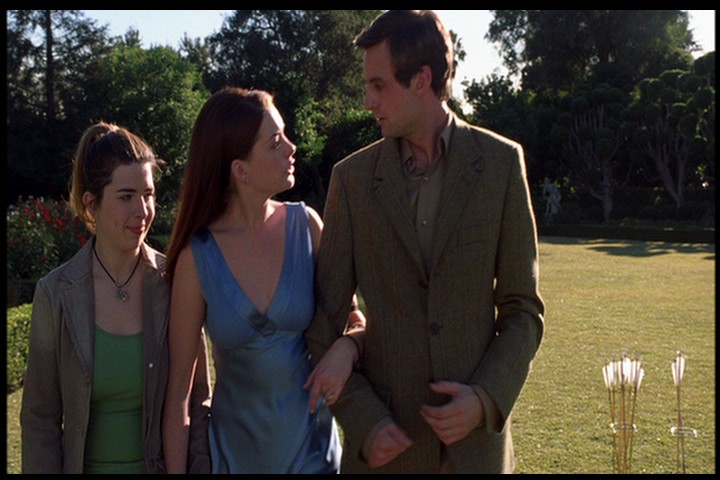

Supplement: S4 Dataset — It also includes pose data and camera parameters. (ZIP) [file pone.0264302.s004.zip › princess-diaries-2-00099351.jpg]

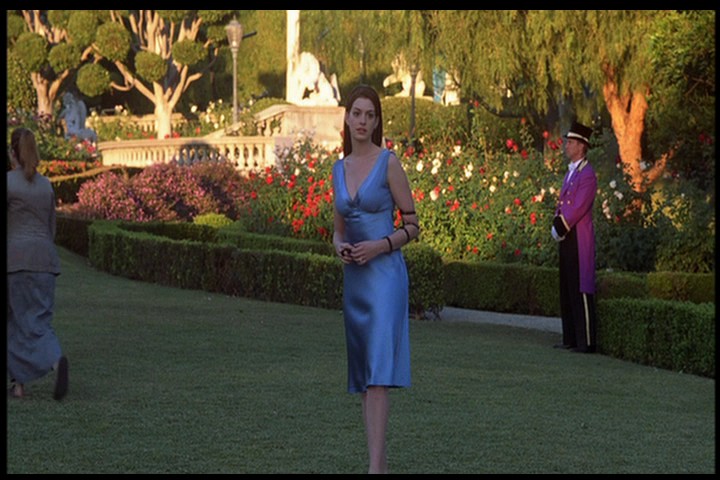

Supplement: S4 Dataset — It also includes pose data and camera parameters. (ZIP) [file pone.0264302.s004.zip › princess-diaries-2-00099511.jpg]

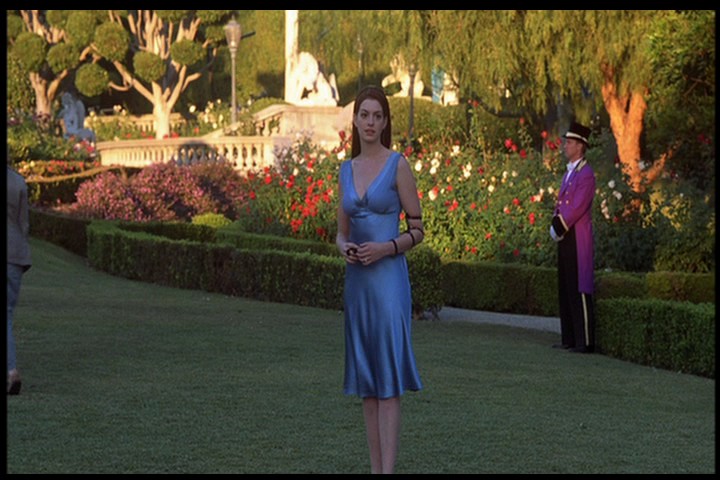

Supplement: S4 Dataset — It also includes pose data and camera parameters. (ZIP) [file pone.0264302.s004.zip › princess-diaries-2-00099521.jpg]

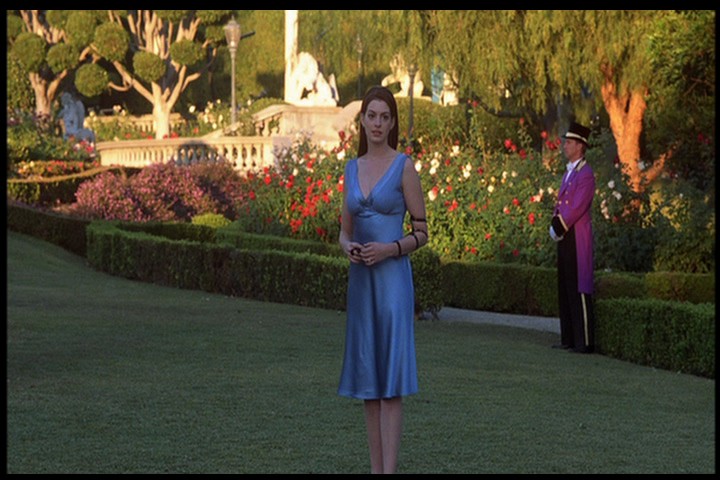

Supplement: S4 Dataset — It also includes pose data and camera parameters. (ZIP) [file pone.0264302.s004.zip › princess-diaries-2-00099531.jpg]

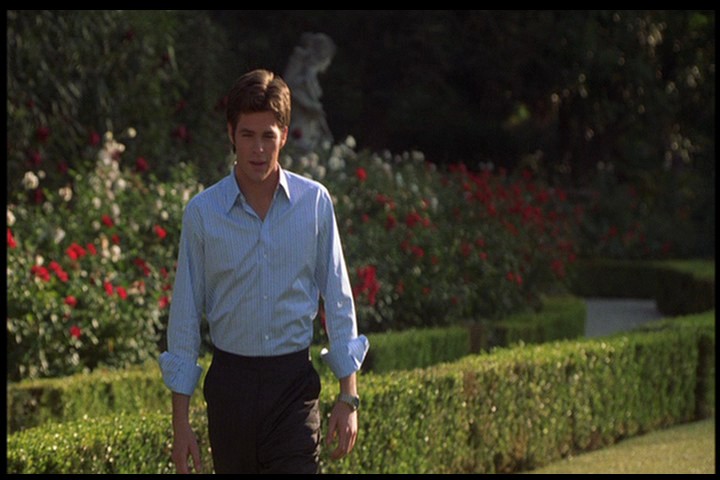

Supplement: S4 Dataset — It also includes pose data and camera parameters. (ZIP) [file pone.0264302.s004.zip › princess-diaries-2-00099541.jpg]

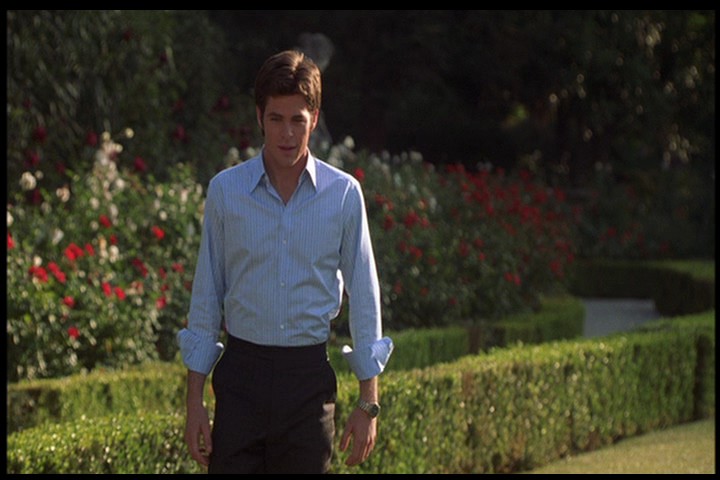

Supplement: S4 Dataset — It also includes pose data and camera parameters. (ZIP) [file pone.0264302.s004.zip › princess-diaries-2-00099551.jpg]

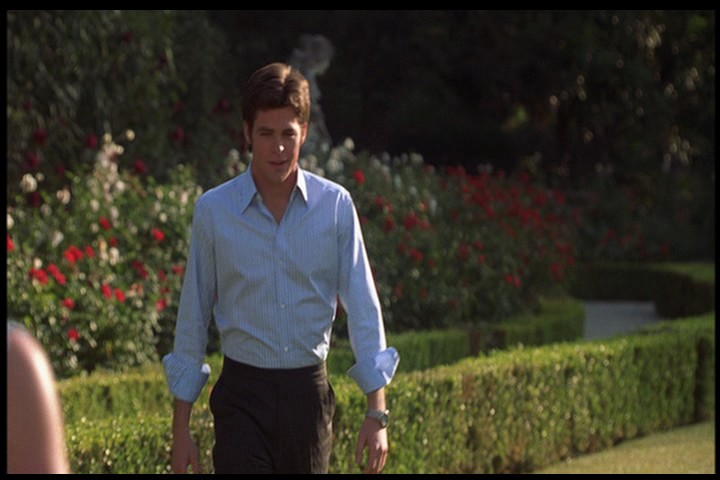

Supplement: S4 Dataset — It also includes pose data and camera parameters. (ZIP) [file pone.0264302.s004.zip › princess-diaries-2-00099561.jpg]

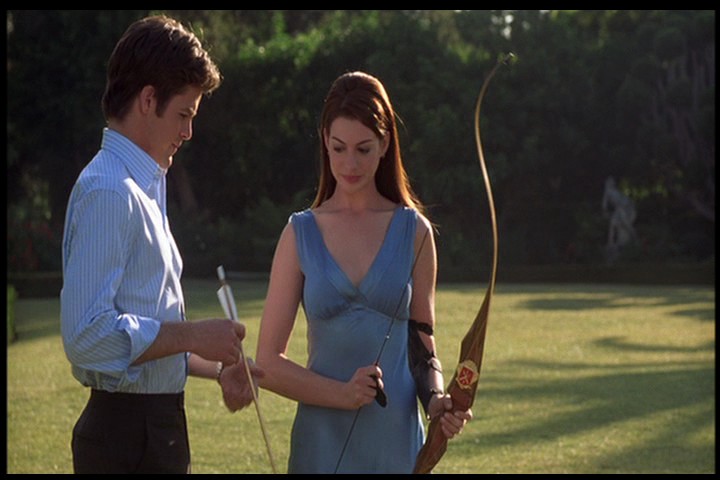

Supplement: S4 Dataset — It also includes pose data and camera parameters. (ZIP) [file pone.0264302.s004.zip › princess-diaries-2-00099761.jpg]

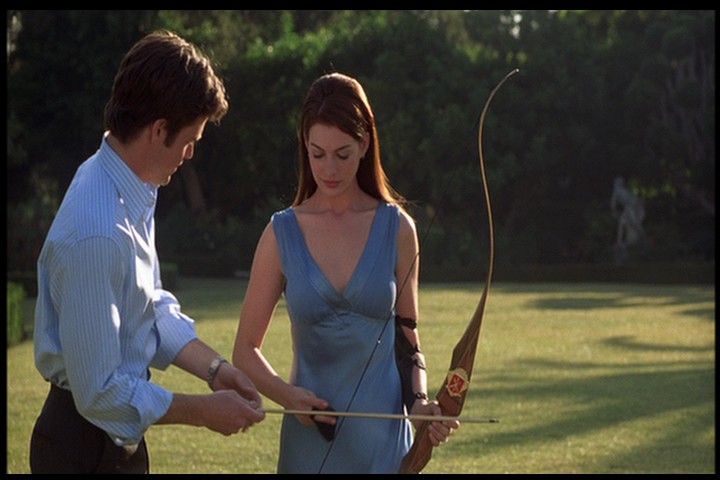

Supplement: S4 Dataset — It also includes pose data and camera parameters. (ZIP) [file pone.0264302.s004.zip › princess-diaries-2-00099781.jpg]

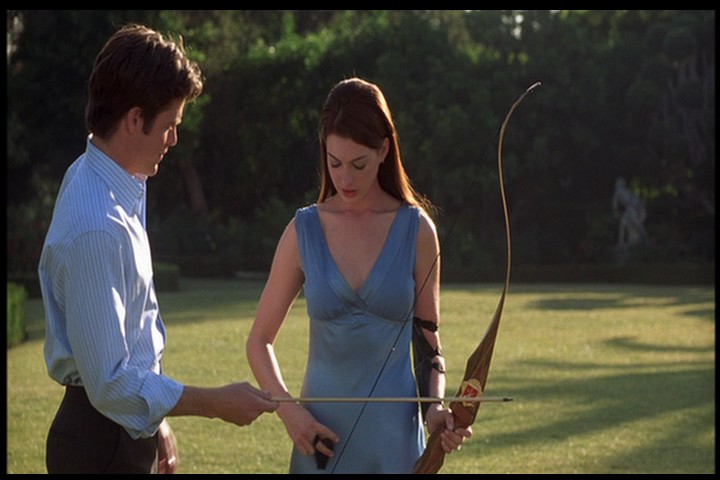

Supplement: S4 Dataset — It also includes pose data and camera parameters. (ZIP) [file pone.0264302.s004.zip › princess-diaries-2-00099791.jpg]

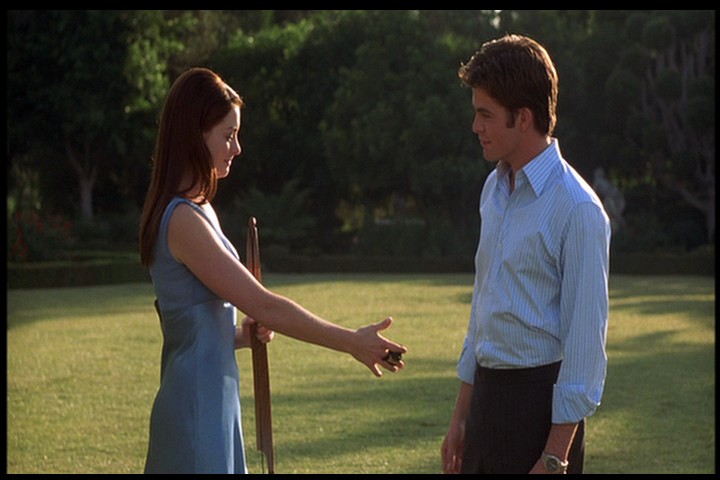

Supplement: S4 Dataset — It also includes pose data and camera parameters. (ZIP) [file pone.0264302.s004.zip › princess-diaries-2-00101561.jpg]

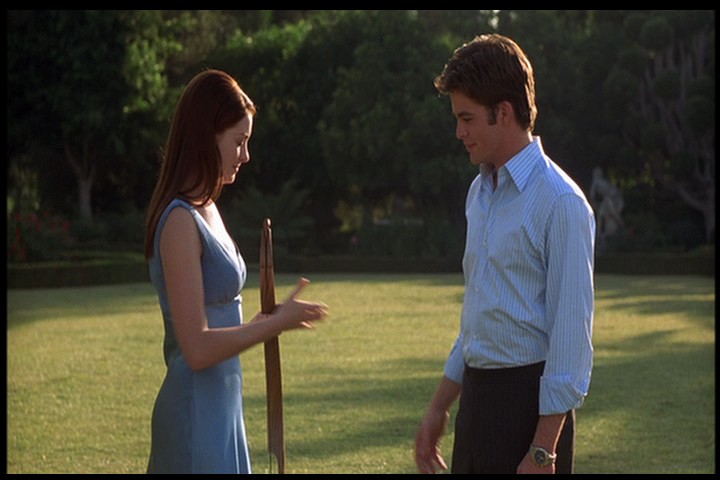

Supplement: S4 Dataset — It also includes pose data and camera parameters. (ZIP) [file pone.0264302.s004.zip › princess-diaries-2-00101601.jpg]

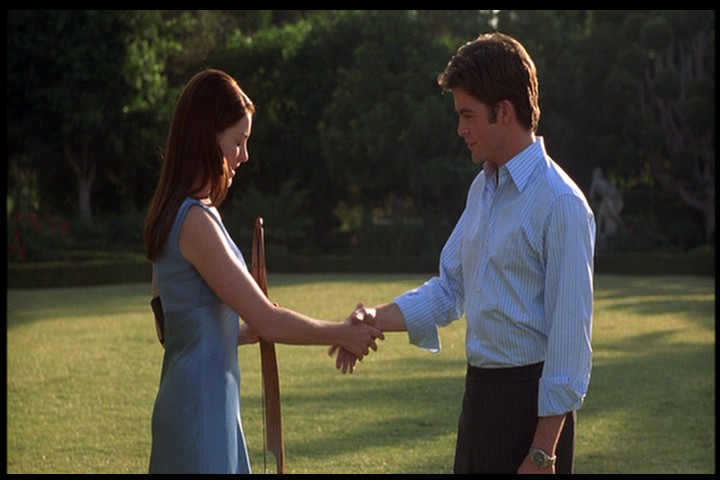

Supplement: S4 Dataset — It also includes pose data and camera parameters. (ZIP) [file pone.0264302.s004.zip › princess-diaries-2-00101611.jpg]

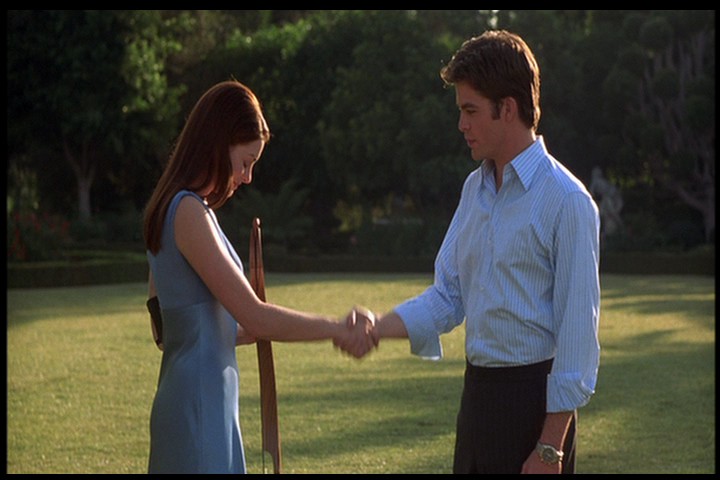

Supplement: S4 Dataset — It also includes pose data and camera parameters. (ZIP) [file pone.0264302.s004.zip › princess-diaries-2-00101621.jpg]

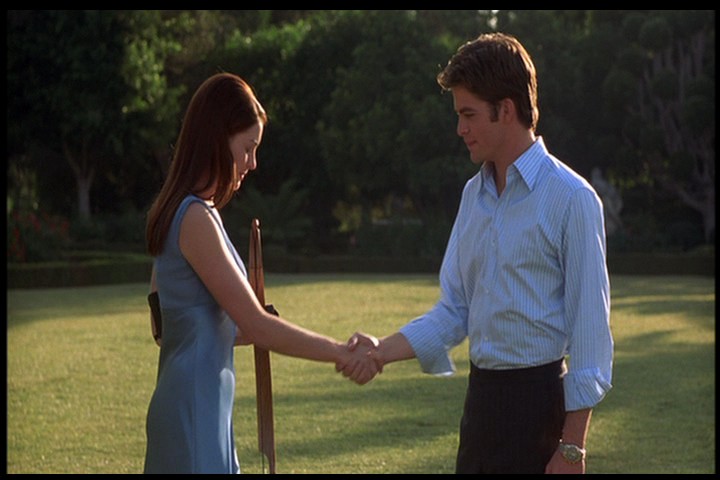

Supplement: S4 Dataset — It also includes pose data and camera parameters. (ZIP) [file pone.0264302.s004.zip › princess-diaries-2-00101631.jpg]

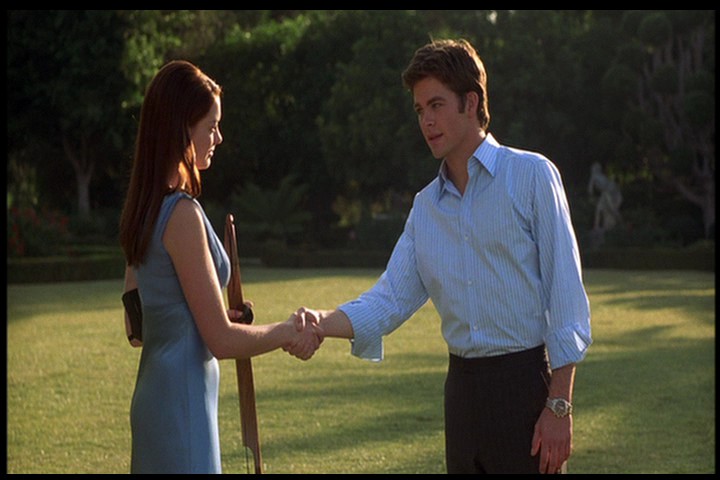

Supplement: S4 Dataset — It also includes pose data and camera parameters. (ZIP) [file pone.0264302.s004.zip › princess-diaries-2-00101691.jpg]

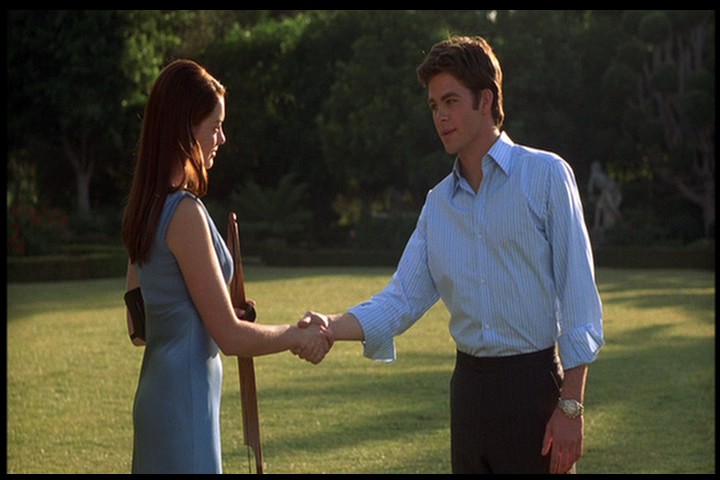

Supplement: S4 Dataset — It also includes pose data and camera parameters. (ZIP) [file pone.0264302.s004.zip › princess-diaries-2-00101701.jpg]

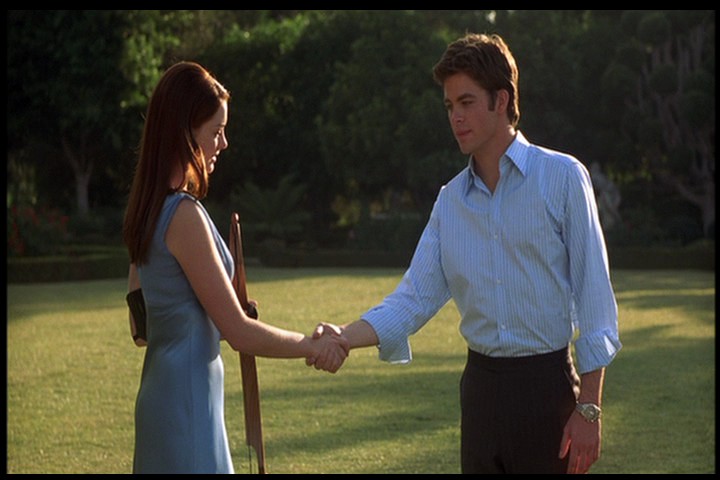

Supplement: S4 Dataset — It also includes pose data and camera parameters. (ZIP) [file pone.0264302.s004.zip › princess-diaries-2-00101711.jpg]

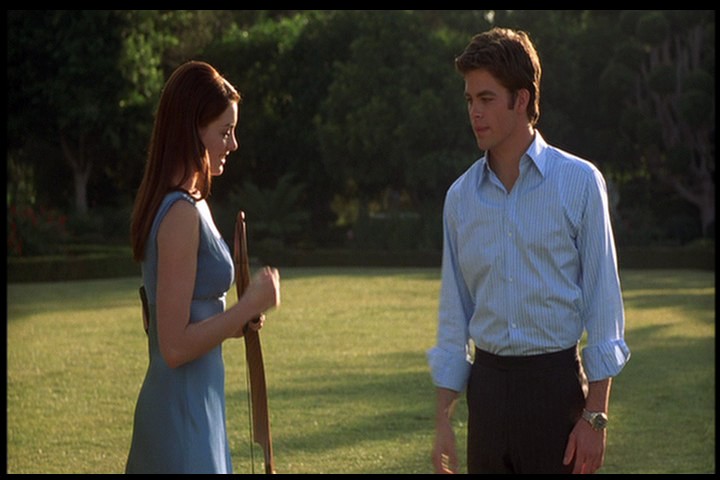

Supplement: S4 Dataset — It also includes pose data and camera parameters. (ZIP) [file pone.0264302.s004.zip › princess-diaries-2-00101731.jpg]

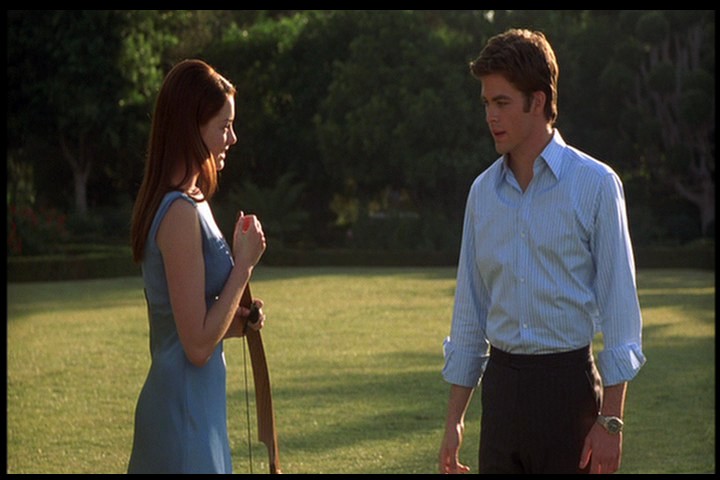

Supplement: S4 Dataset — It also includes pose data and camera parameters. (ZIP) [file pone.0264302.s004.zip › princess-diaries-2-00101741.jpg]

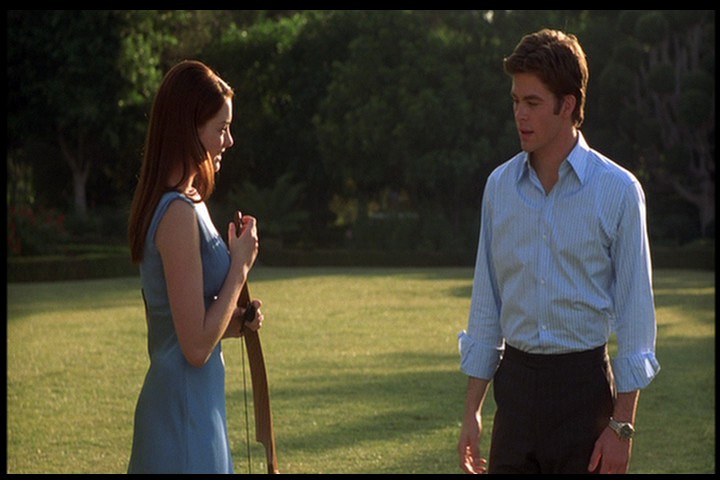

Supplement: S4 Dataset — It also includes pose data and camera parameters. (ZIP) [file pone.0264302.s004.zip › princess-diaries-2-00101751.jpg]

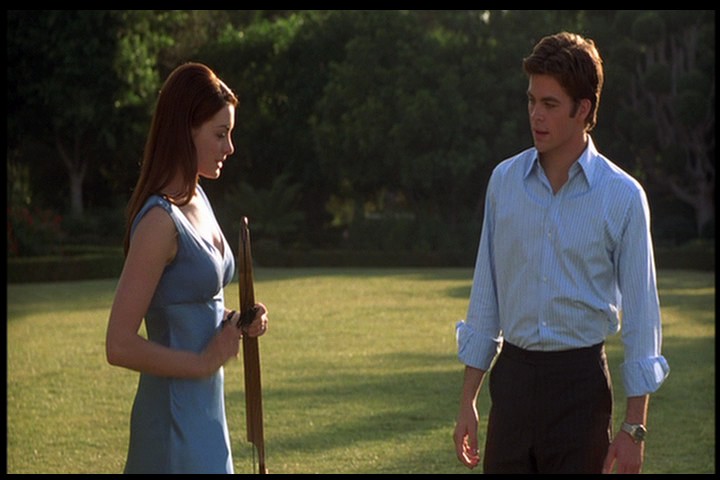

Supplement: S4 Dataset — It also includes pose data and camera parameters. (ZIP) [file pone.0264302.s004.zip › princess-diaries-2-00101771.jpg]

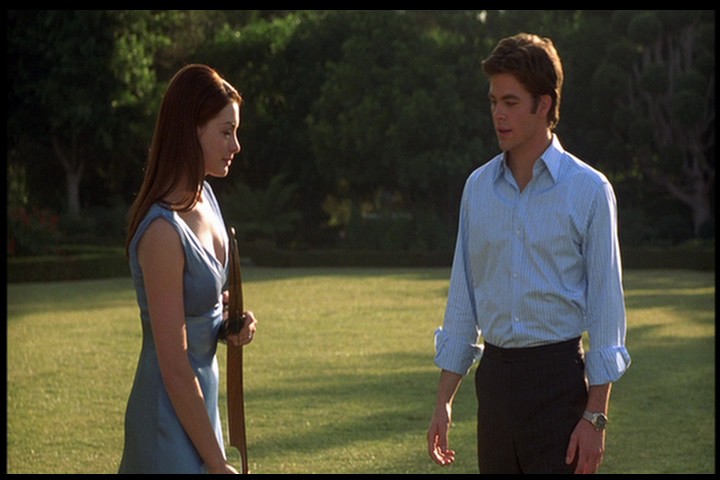

Supplement: S4 Dataset — It also includes pose data and camera parameters. (ZIP) [file pone.0264302.s004.zip › princess-diaries-2-00101781.jpg]

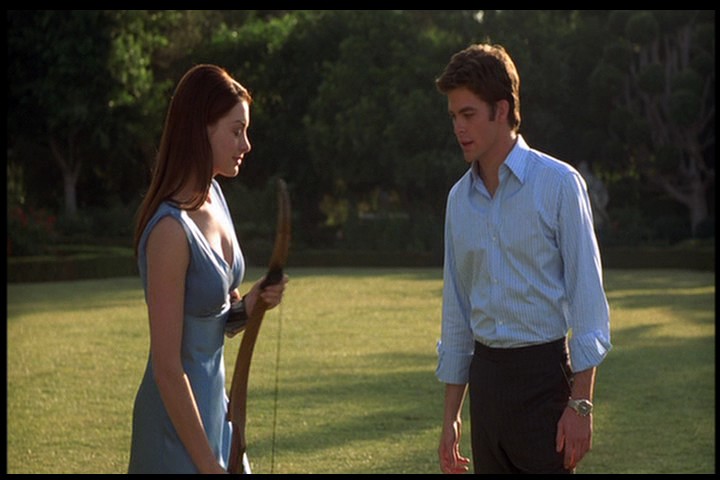

Supplement: S4 Dataset — It also includes pose data and camera parameters. (ZIP) [file pone.0264302.s004.zip › princess-diaries-2-00101791.jpg]

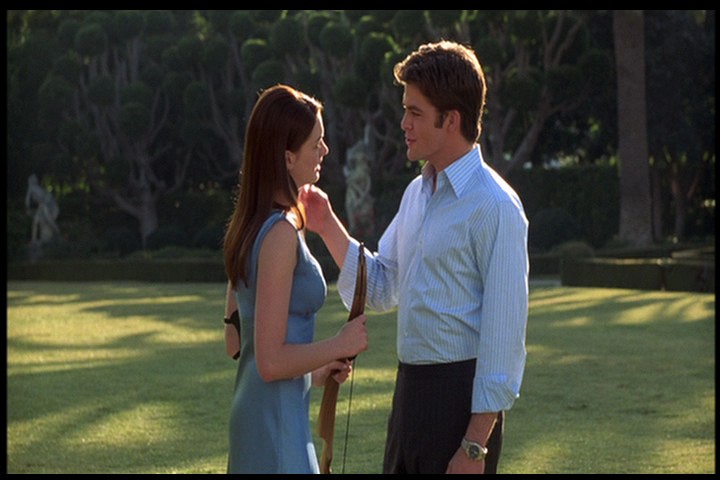

Supplement: S4 Dataset — It also includes pose data and camera parameters. (ZIP) [file pone.0264302.s004.zip › princess-diaries-2-00102061.jpg]

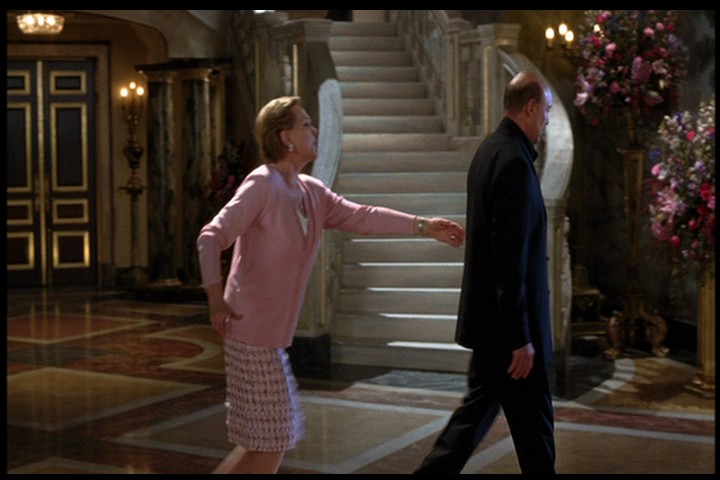

Supplement: S4 Dataset — It also includes pose data and camera parameters. (ZIP) [file pone.0264302.s004.zip › princess-diaries-2-00104551.jpg]

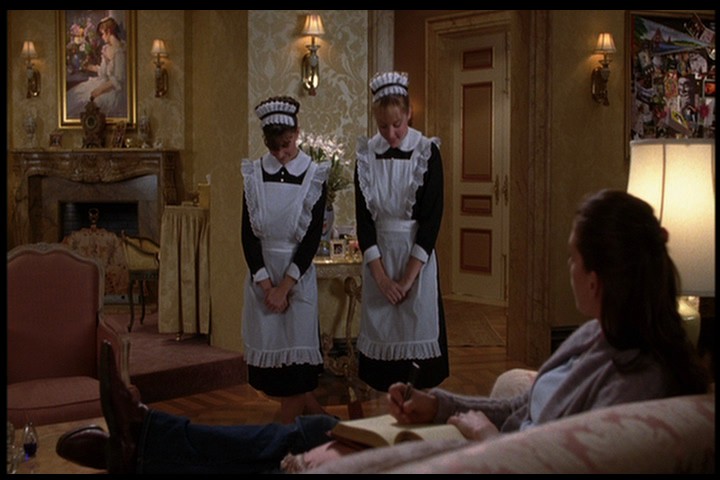

Supplement: S4 Dataset — It also includes pose data and camera parameters. (ZIP) [file pone.0264302.s004.zip › princess-diaries-2-00105931.jpg]

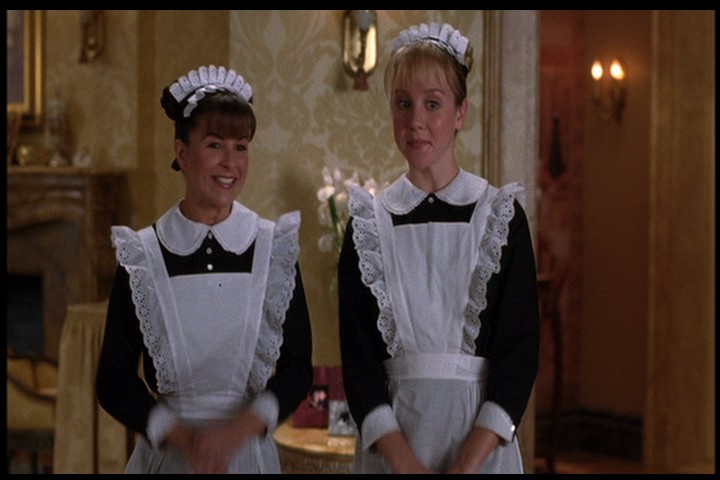

Supplement: S4 Dataset — It also includes pose data and camera parameters. (ZIP) [file pone.0264302.s004.zip › princess-diaries-2-00106061.jpg]

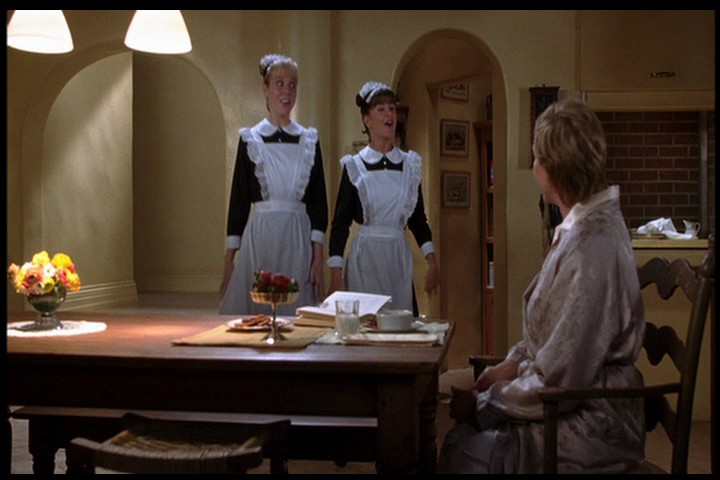

Supplement: S4 Dataset — It also includes pose data and camera parameters. (ZIP) [file pone.0264302.s004.zip › princess-diaries-2-00108861.jpg]

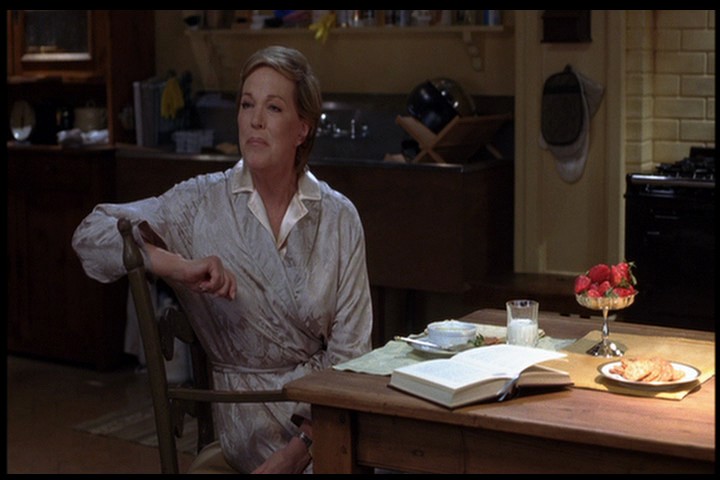

Supplement: S4 Dataset — It also includes pose data and camera parameters. (ZIP) [file pone.0264302.s004.zip › princess-diaries-2-00109471.jpg]

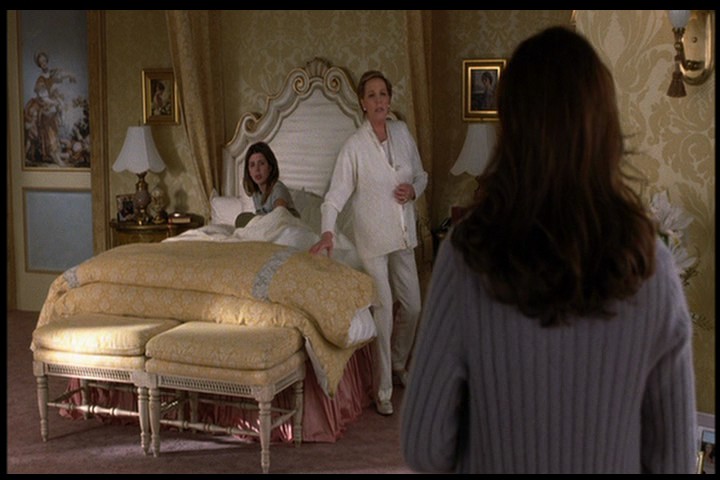

Supplement: S4 Dataset — It also includes pose data and camera parameters. (ZIP) [file pone.0264302.s004.zip › princess-diaries-2-00114691.jpg]

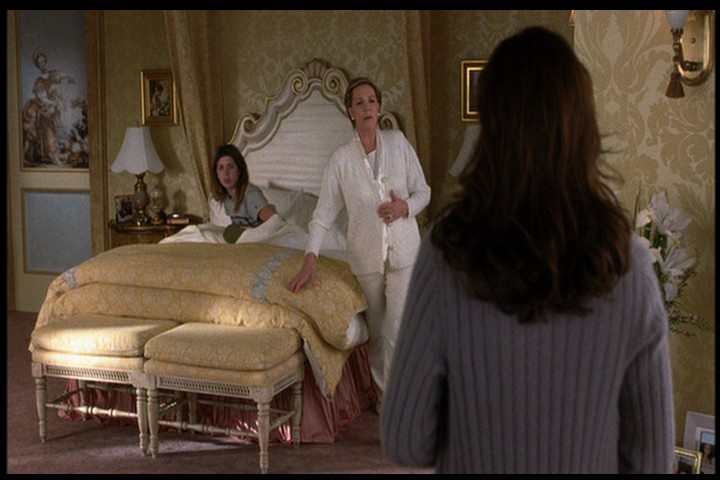

Supplement: S4 Dataset — It also includes pose data and camera parameters. (ZIP) [file pone.0264302.s004.zip › princess-diaries-2-00114701.jpg]

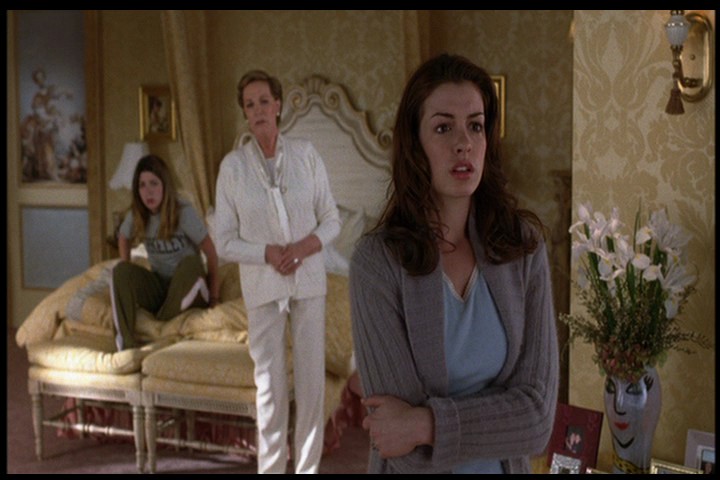

Supplement: S4 Dataset — It also includes pose data and camera parameters. (ZIP) [file pone.0264302.s004.zip › princess-diaries-2-00114821.jpg]

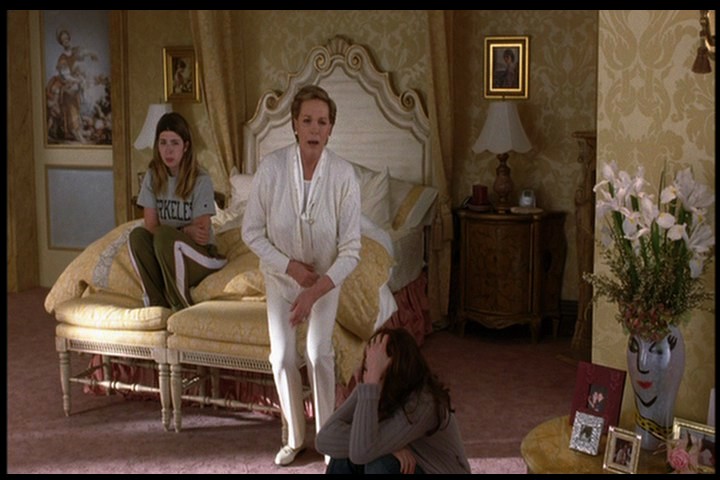

Supplement: S4 Dataset — It also includes pose data and camera parameters. (ZIP) [file pone.0264302.s004.zip › princess-diaries-2-00115071.jpg]

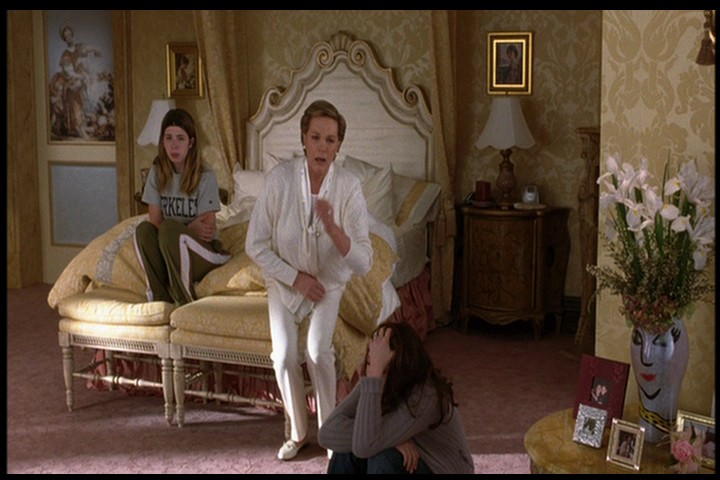

Supplement: S4 Dataset — It also includes pose data and camera parameters. (ZIP) [file pone.0264302.s004.zip › princess-diaries-2-00115081.jpg]

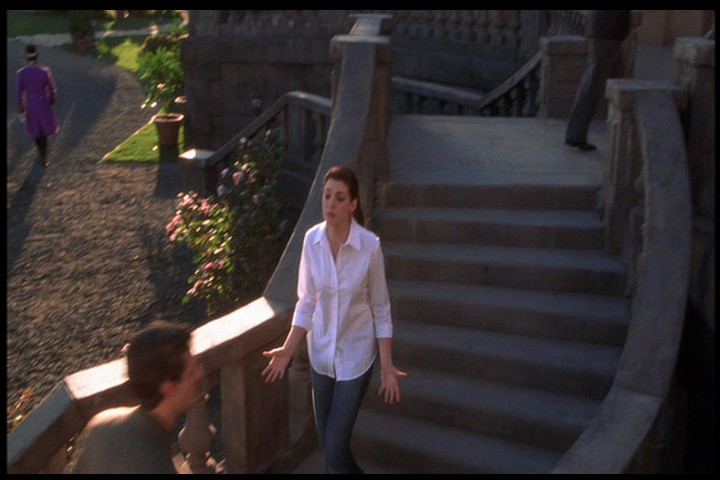

Supplement: S4 Dataset — It also includes pose data and camera parameters. (ZIP) [file pone.0264302.s004.zip › princess-diaries-2-00116471.jpg]

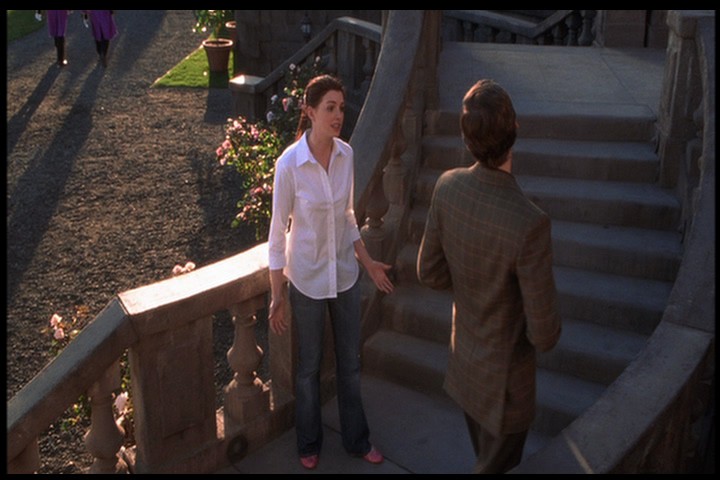

Supplement: S4 Dataset — It also includes pose data and camera parameters. (ZIP) [file pone.0264302.s004.zip › princess-diaries-2-00116501.jpg]

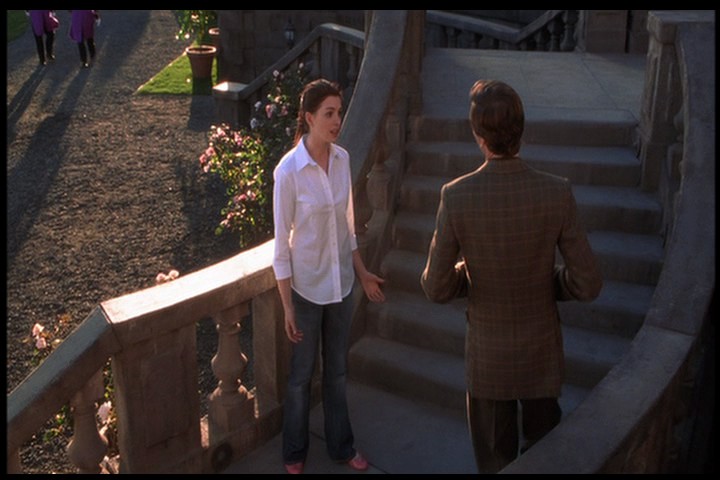

Supplement: S4 Dataset — It also includes pose data and camera parameters. (ZIP) [file pone.0264302.s004.zip › princess-diaries-2-00116511.jpg]

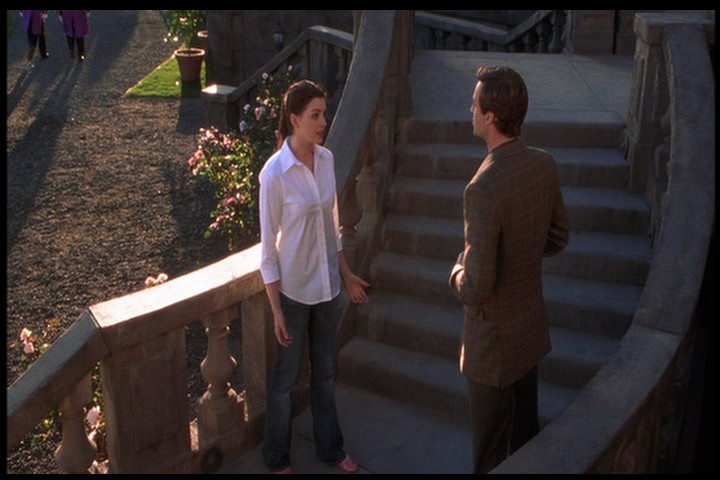

Supplement: S4 Dataset — It also includes pose data and camera parameters. (ZIP) [file pone.0264302.s004.zip › princess-diaries-2-00116521.jpg]

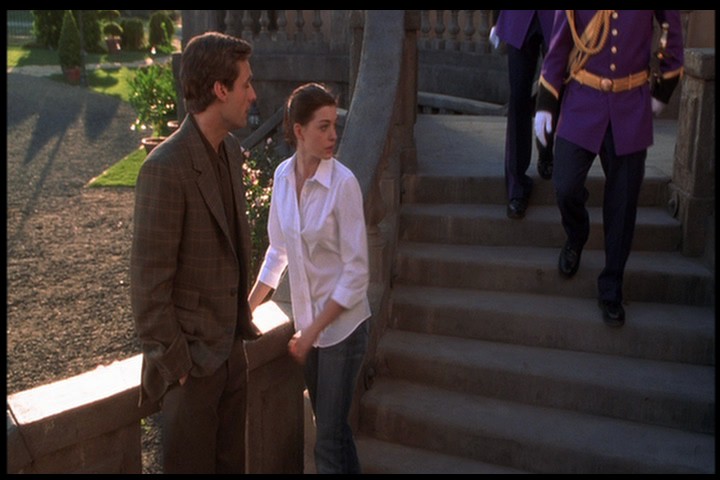

Supplement: S4 Dataset — It also includes pose data and camera parameters. (ZIP) [file pone.0264302.s004.zip › princess-diaries-2-00118301.jpg]

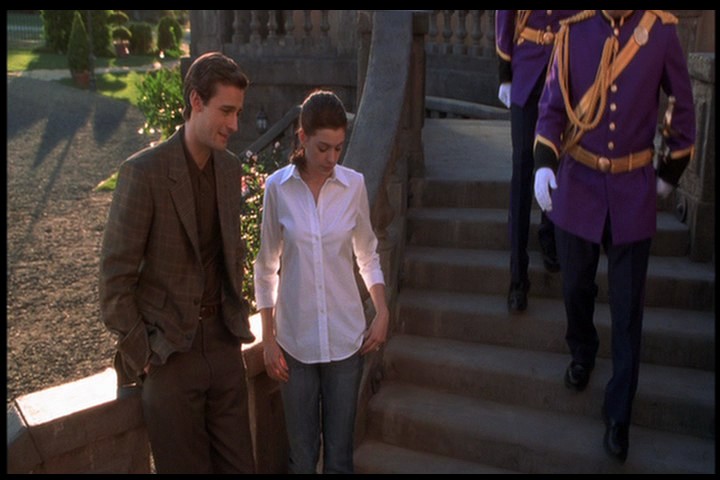

Supplement: S4 Dataset — It also includes pose data and camera parameters. (ZIP) [file pone.0264302.s004.zip › princess-diaries-2-00118321.jpg]

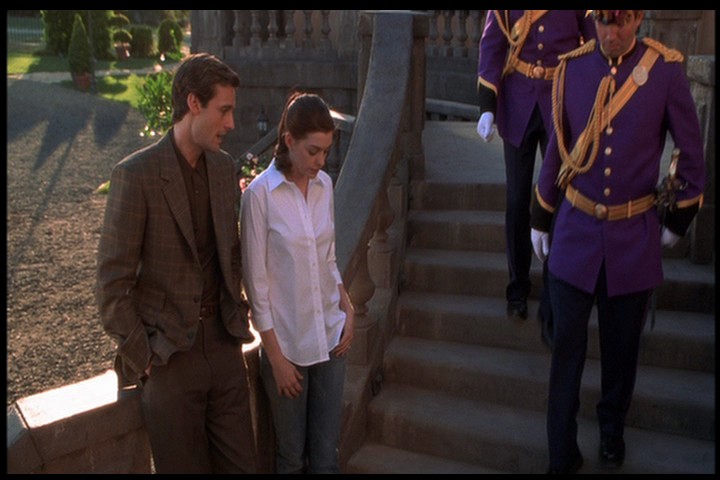

Supplement: S4 Dataset — It also includes pose data and camera parameters. (ZIP) [file pone.0264302.s004.zip › princess-diaries-2-00118331.jpg]

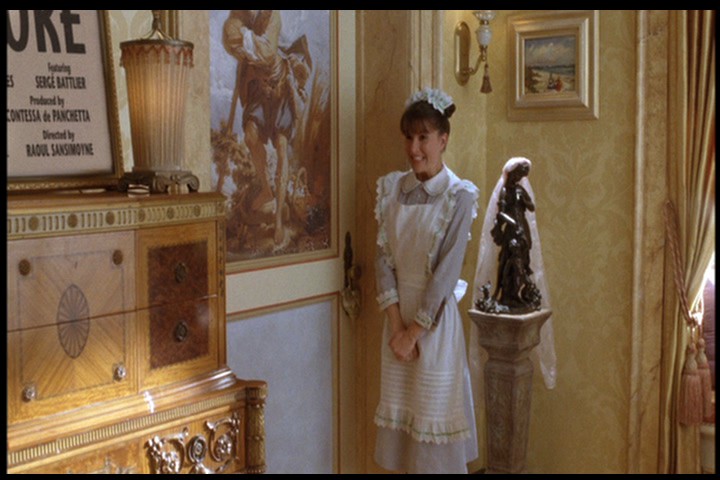

Supplement: S4 Dataset — It also includes pose data and camera parameters. (ZIP) [file pone.0264302.s004.zip › princess-diaries-2-00119761.jpg]

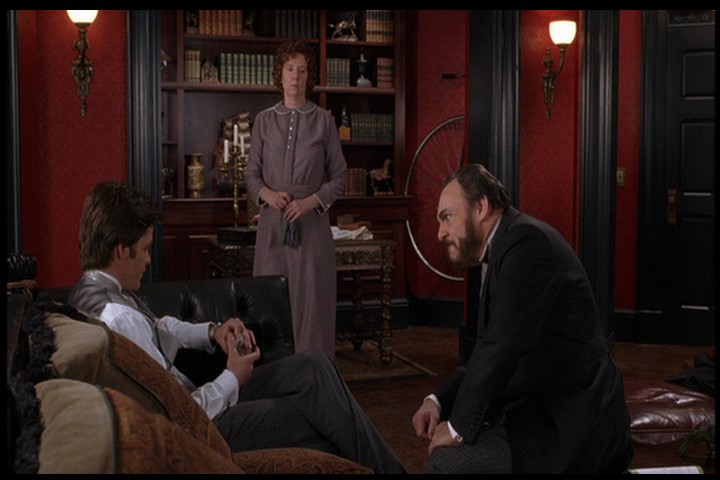

Supplement: S4 Dataset — It also includes pose data and camera parameters. (ZIP) [file pone.0264302.s004.zip › princess-diaries-2-00123391.jpg]

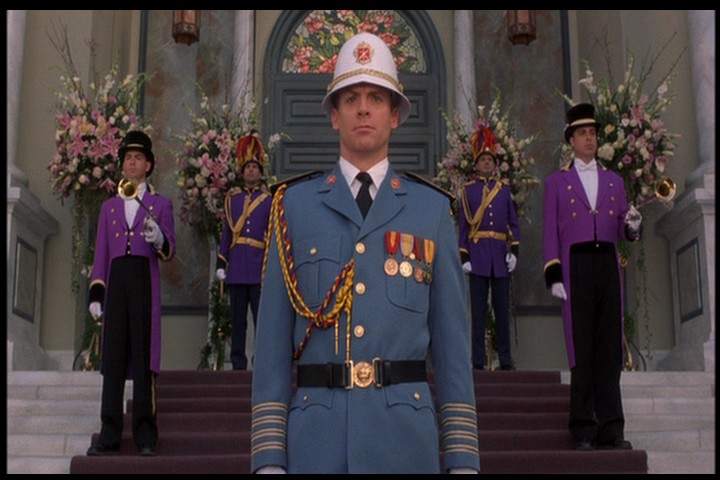

Supplement: S4 Dataset — It also includes pose data and camera parameters. (ZIP) [file pone.0264302.s004.zip › princess-diaries-2-00123821.jpg]

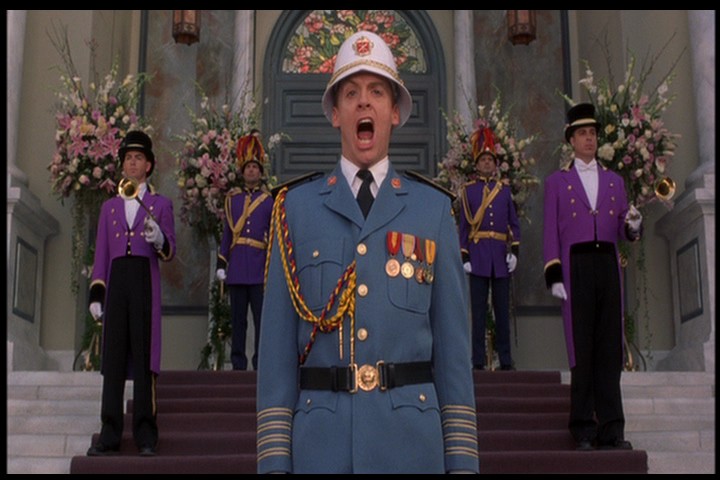

Supplement: S4 Dataset — It also includes pose data and camera parameters. (ZIP) [file pone.0264302.s004.zip › princess-diaries-2-00123831.jpg]

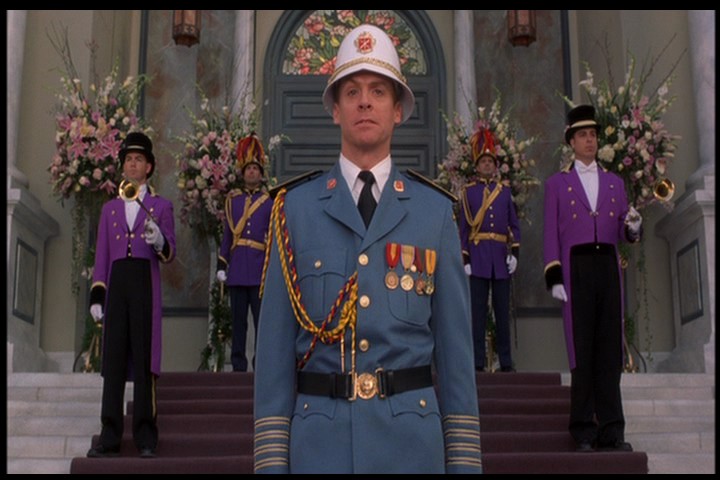

Supplement: S4 Dataset — It also includes pose data and camera parameters. (ZIP) [file pone.0264302.s004.zip › princess-diaries-2-00123871.jpg]

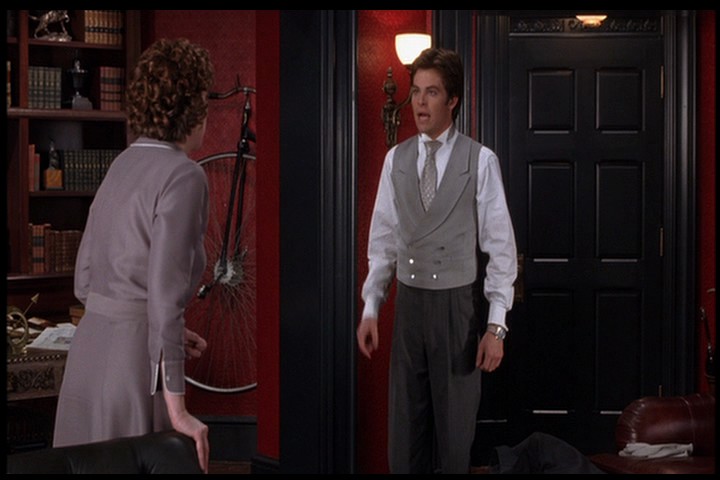

Supplement: S4 Dataset — It also includes pose data and camera parameters. (ZIP) [file pone.0264302.s004.zip › princess-diaries-2-00125071.jpg]

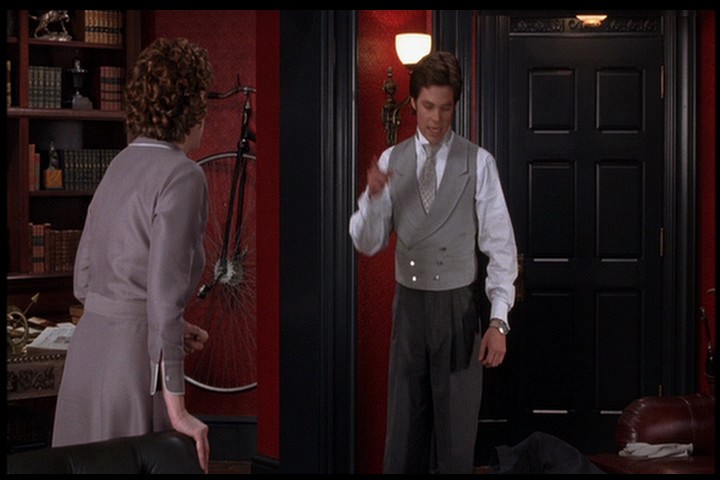

Supplement: S4 Dataset — It also includes pose data and camera parameters. (ZIP) [file pone.0264302.s004.zip › princess-diaries-2-00125081.jpg]

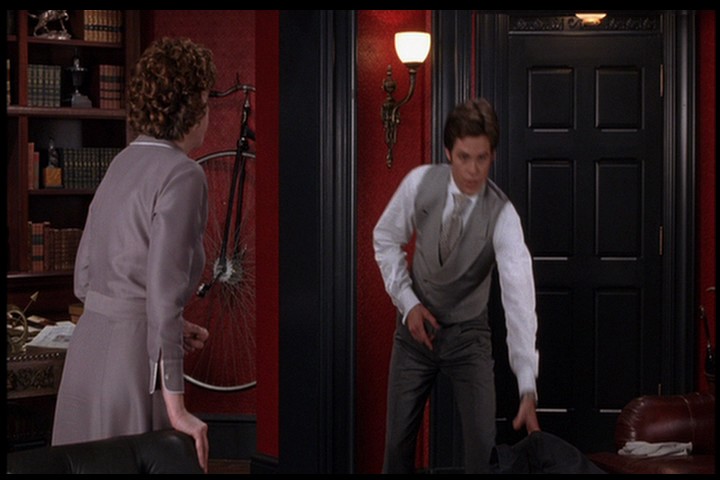

Supplement: S4 Dataset — It also includes pose data and camera parameters. (ZIP) [file pone.0264302.s004.zip › princess-diaries-2-00125091.jpg]

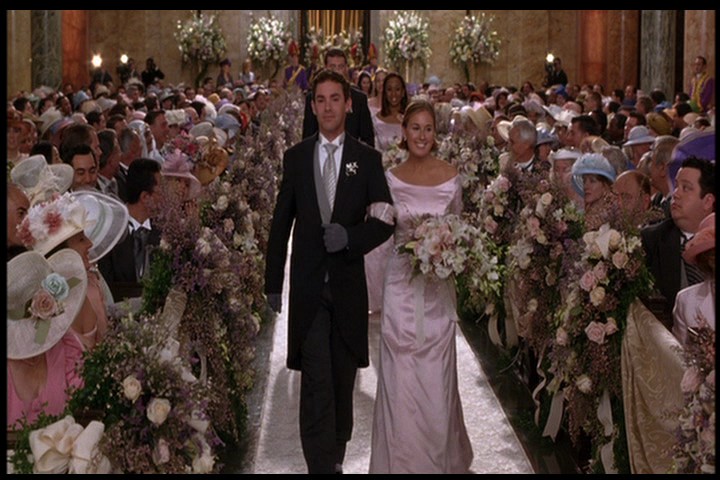

Supplement: S4 Dataset — It also includes pose data and camera parameters. (ZIP) [file pone.0264302.s004.zip › princess-diaries-2-00127031.jpg]

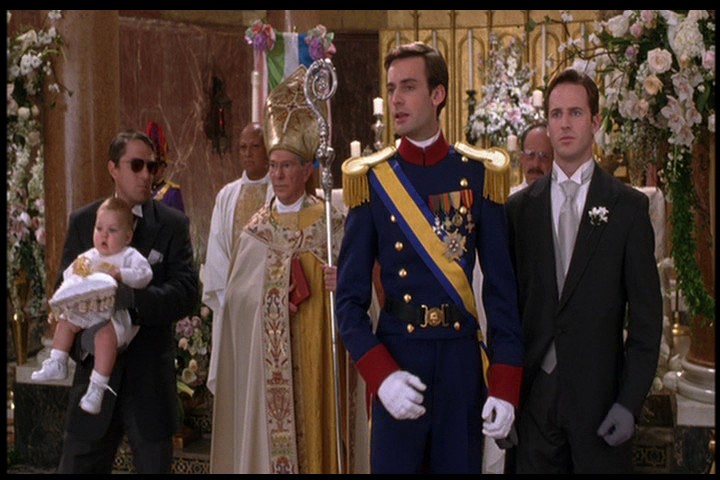

Supplement: S4 Dataset — It also includes pose data and camera parameters. (ZIP) [file pone.0264302.s004.zip › princess-diaries-2-00130871.jpg]

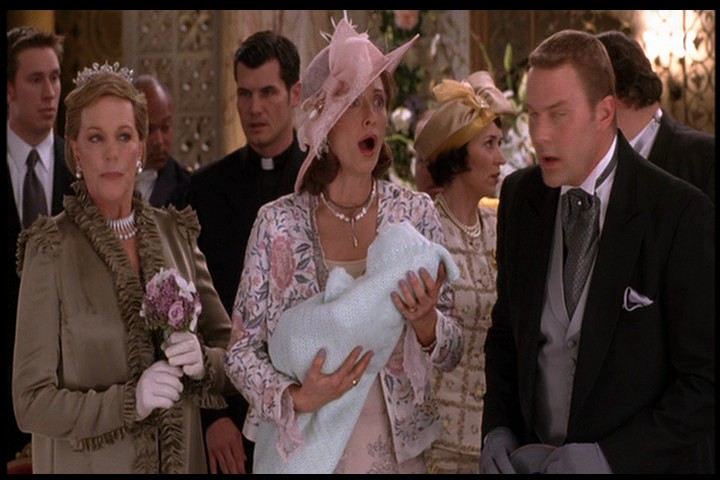

Supplement: S4 Dataset — It also includes pose data and camera parameters. (ZIP) [file pone.0264302.s004.zip › princess-diaries-2-00130931.jpg]

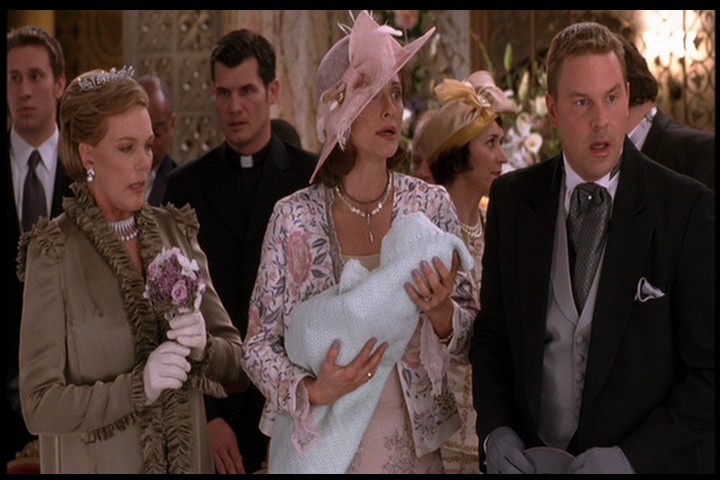

Supplement: S4 Dataset — It also includes pose data and camera parameters. (ZIP) [file pone.0264302.s004.zip › princess-diaries-2-00130941.jpg]

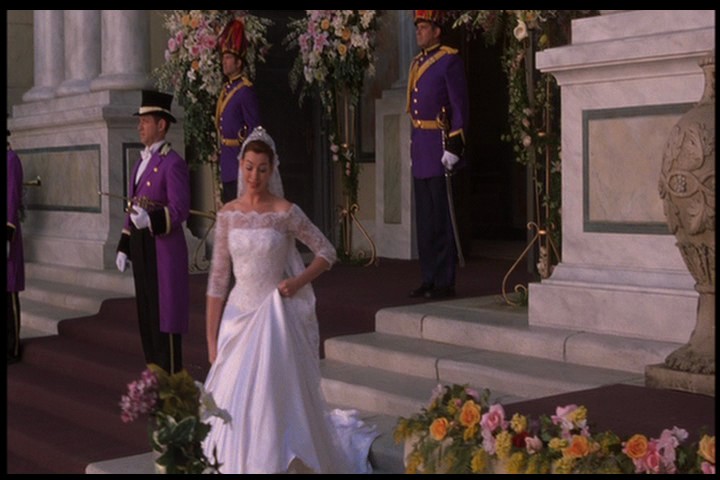

Supplement: S4 Dataset — It also includes pose data and camera parameters. (ZIP) [file pone.0264302.s004.zip › princess-diaries-2-00131211.jpg]

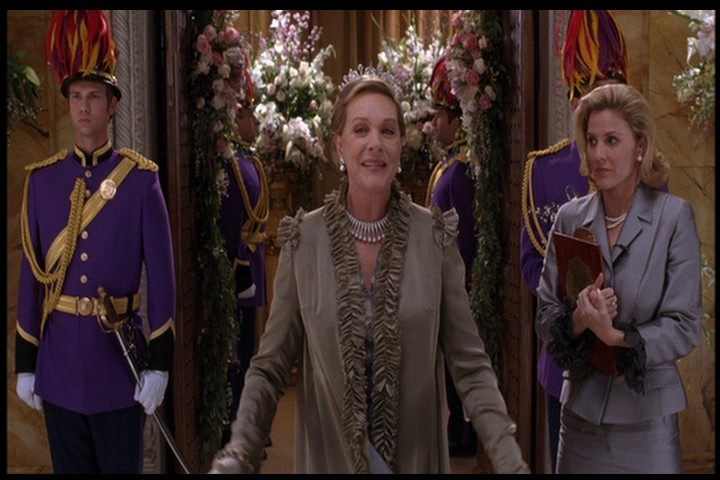

Supplement: S4 Dataset — It also includes pose data and camera parameters. (ZIP) [file pone.0264302.s004.zip › princess-diaries-2-00131481.jpg]

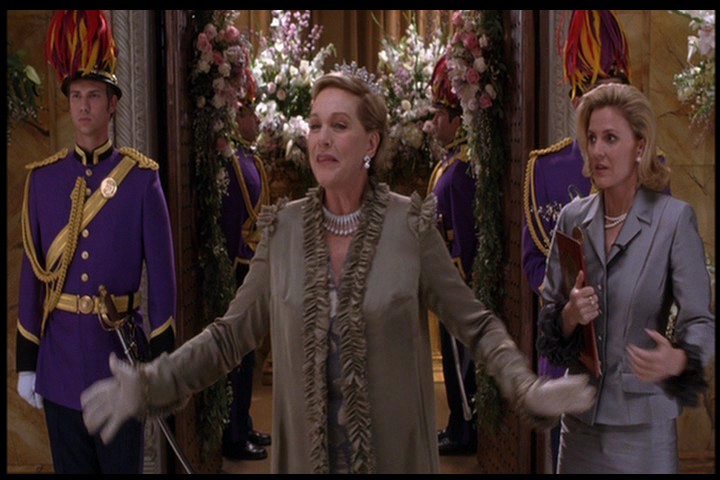

Supplement: S4 Dataset — It also includes pose data and camera parameters. (ZIP) [file pone.0264302.s004.zip › princess-diaries-2-00131501.jpg]

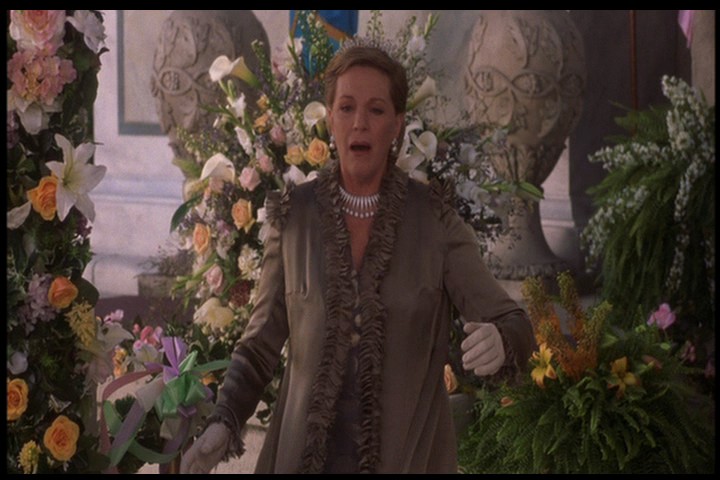

Supplement: S4 Dataset — It also includes pose data and camera parameters. (ZIP) [file pone.0264302.s004.zip › princess-diaries-2-00131981.jpg]

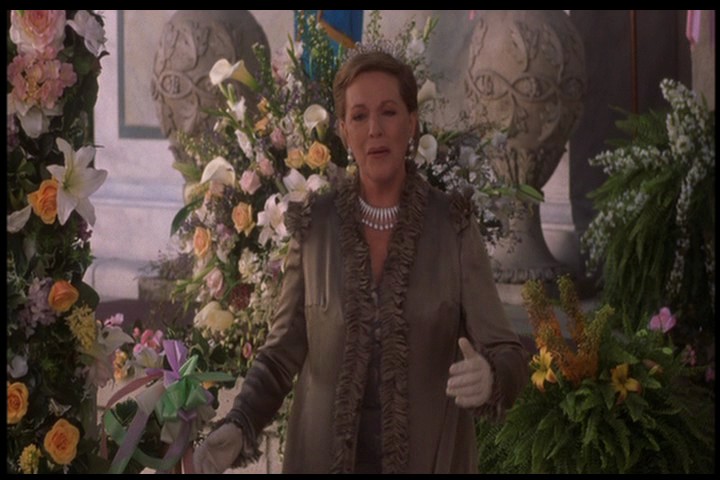

Supplement: S4 Dataset — It also includes pose data and camera parameters. (ZIP) [file pone.0264302.s004.zip › princess-diaries-2-00131991.jpg]

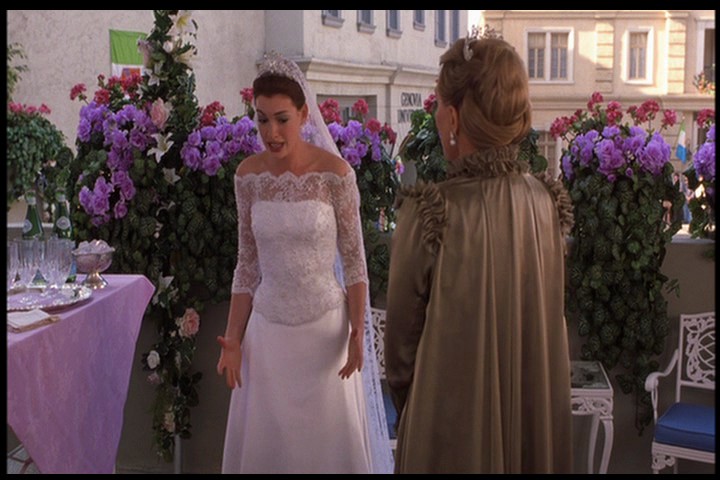

Supplement: S4 Dataset — It also includes pose data and camera parameters. (ZIP) [file pone.0264302.s004.zip › princess-diaries-2-00132231.jpg]

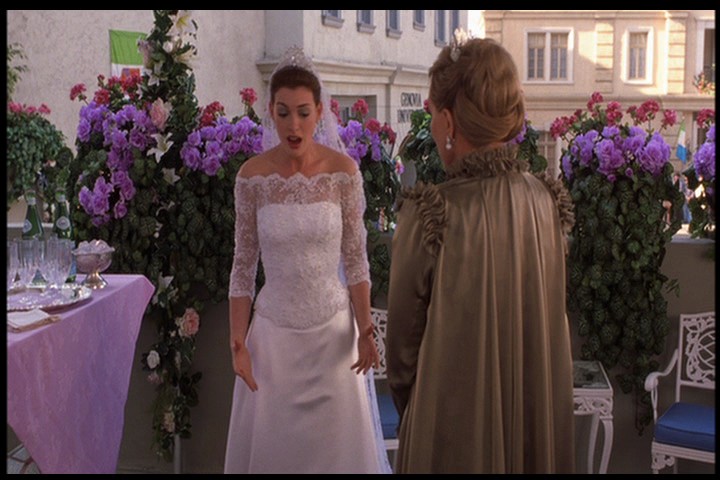

Supplement: S4 Dataset — It also includes pose data and camera parameters. (ZIP) [file pone.0264302.s004.zip › princess-diaries-2-00132241.jpg]

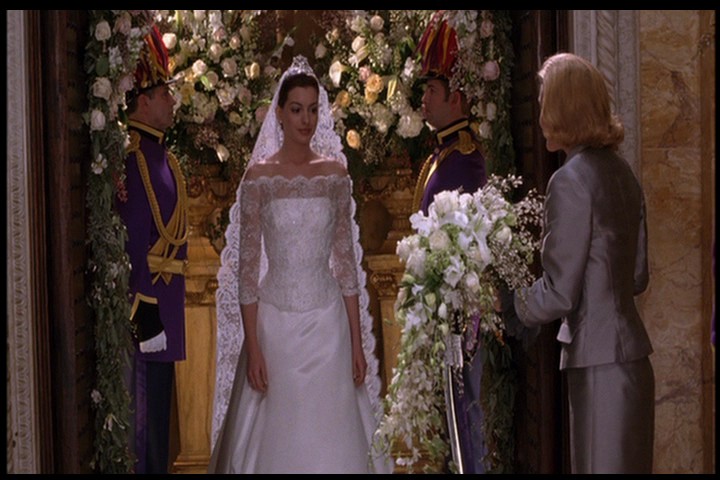

Supplement: S4 Dataset — It also includes pose data and camera parameters. (ZIP) [file pone.0264302.s004.zip › princess-diaries-2-00133591.jpg]

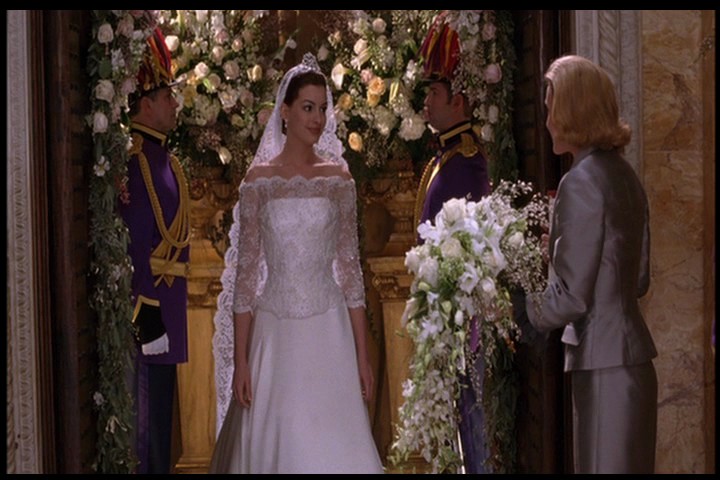

Supplement: S4 Dataset — It also includes pose data and camera parameters. (ZIP) [file pone.0264302.s004.zip › princess-diaries-2-00133601.jpg]

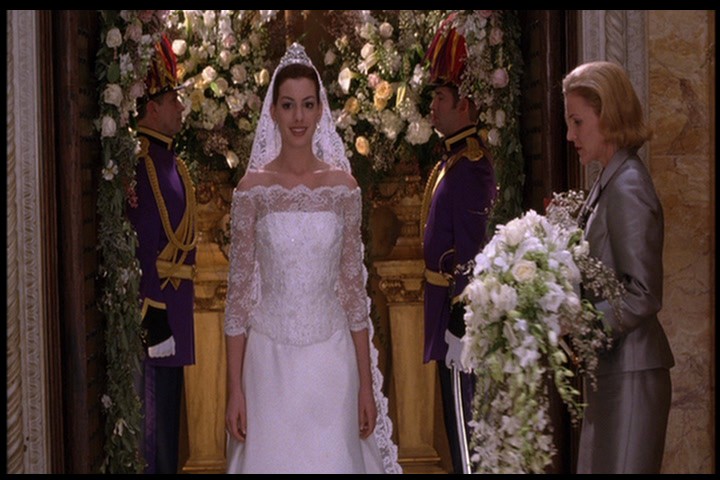

Supplement: S4 Dataset — It also includes pose data and camera parameters. (ZIP) [file pone.0264302.s004.zip › princess-diaries-2-00133621.jpg]

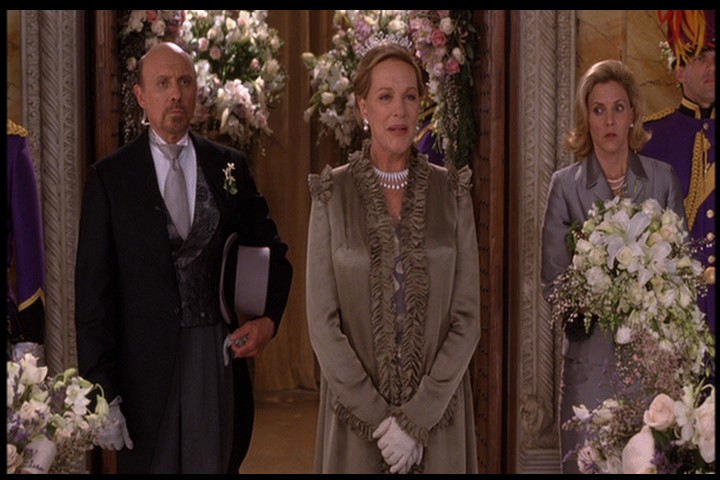

Supplement: S4 Dataset — It also includes pose data and camera parameters. (ZIP) [file pone.0264302.s004.zip › princess-diaries-2-00135671.jpg]

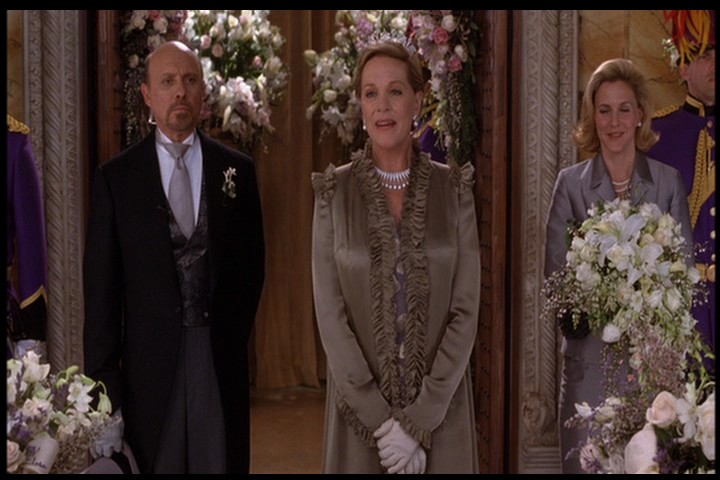

Supplement: S4 Dataset — It also includes pose data and camera parameters. (ZIP) [file pone.0264302.s004.zip › princess-diaries-2-00137641.jpg]

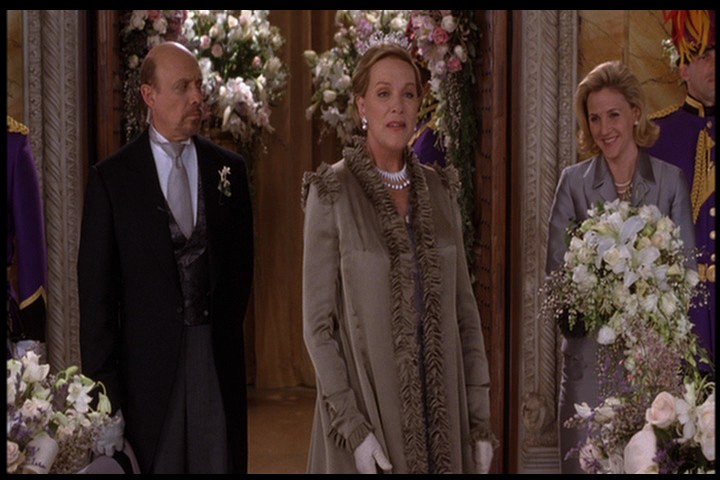

Supplement: S4 Dataset — It also includes pose data and camera parameters. (ZIP) [file pone.0264302.s004.zip › princess-diaries-2-00137681.jpg]

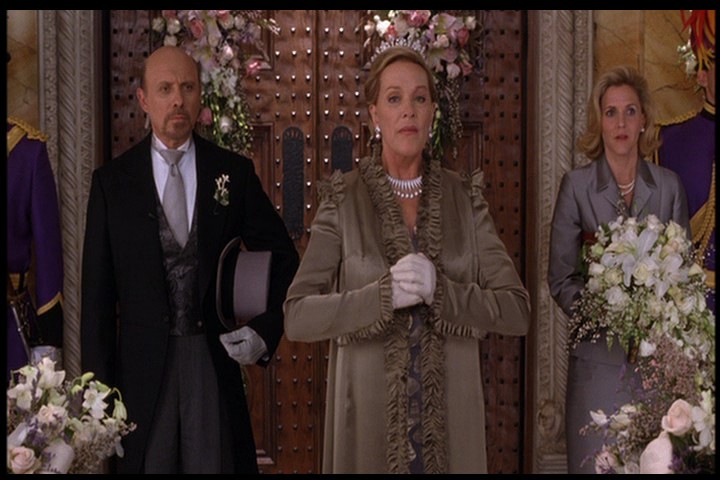

Supplement: S4 Dataset — It also includes pose data and camera parameters. (ZIP) [file pone.0264302.s004.zip › princess-diaries-2-00141181.jpg]

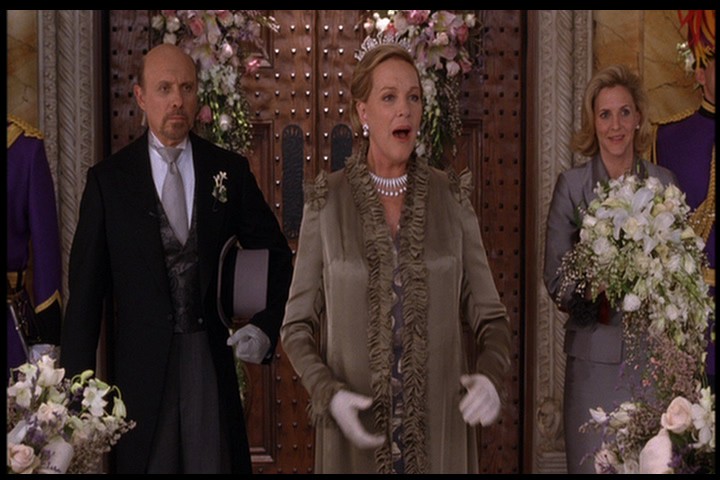

Supplement: S4 Dataset — It also includes pose data and camera parameters. (ZIP) [file pone.0264302.s004.zip › princess-diaries-2-00141421.jpg]

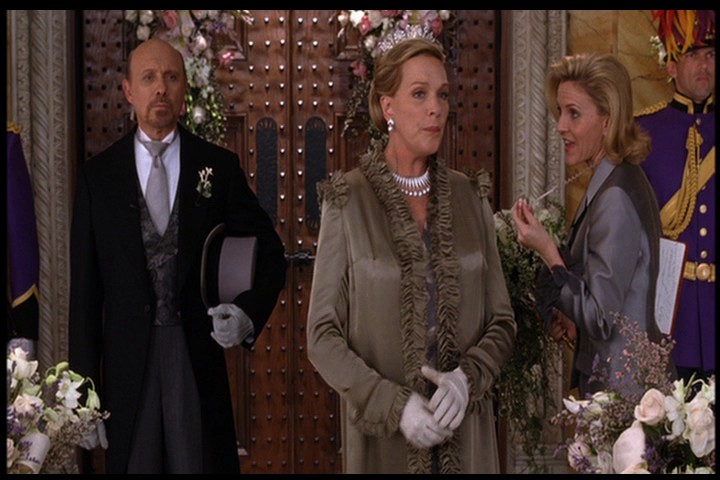

Supplement: S4 Dataset — It also includes pose data and camera parameters. (ZIP) [file pone.0264302.s004.zip › princess-diaries-2-00142821.jpg]

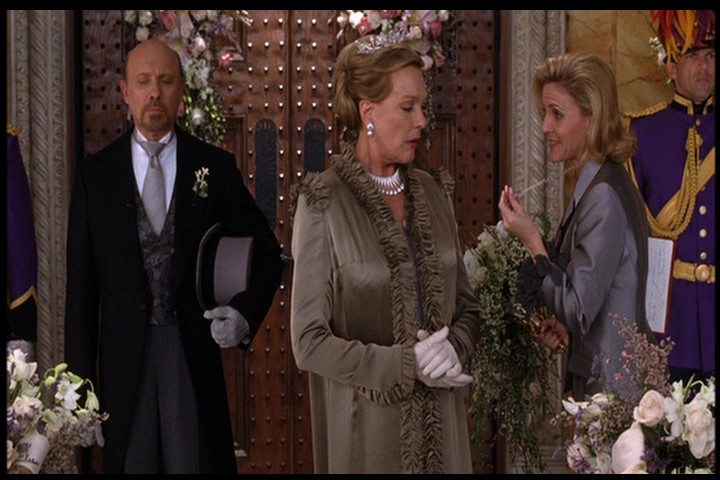

Supplement: S4 Dataset — It also includes pose data and camera parameters. (ZIP) [file pone.0264302.s004.zip › princess-diaries-2-00142921.jpg]

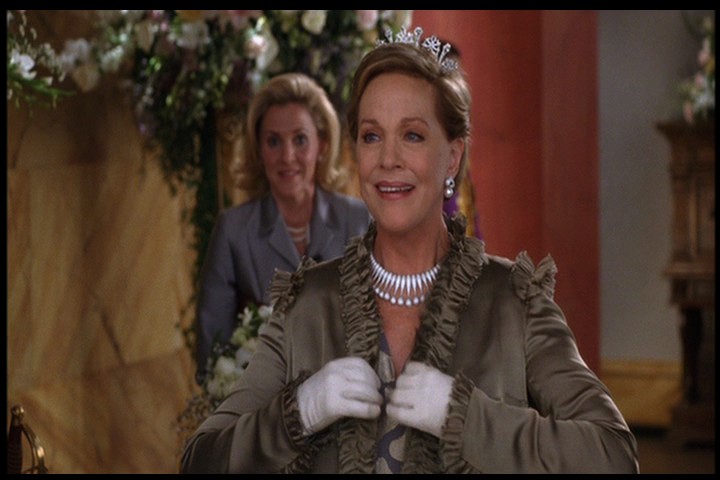

Supplement: S4 Dataset — It also includes pose data and camera parameters. (ZIP) [file pone.0264302.s004.zip › princess-diaries-2-00143621.jpg]

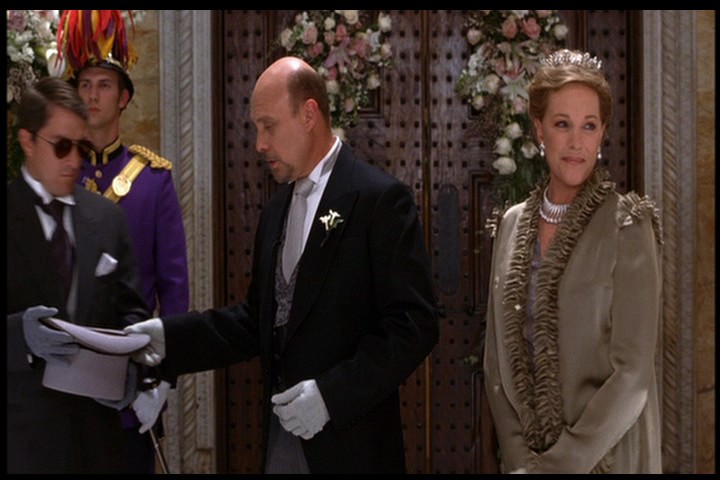

Supplement: S4 Dataset — It also includes pose data and camera parameters. (ZIP) [file pone.0264302.s004.zip › princess-diaries-2-00144431.jpg]

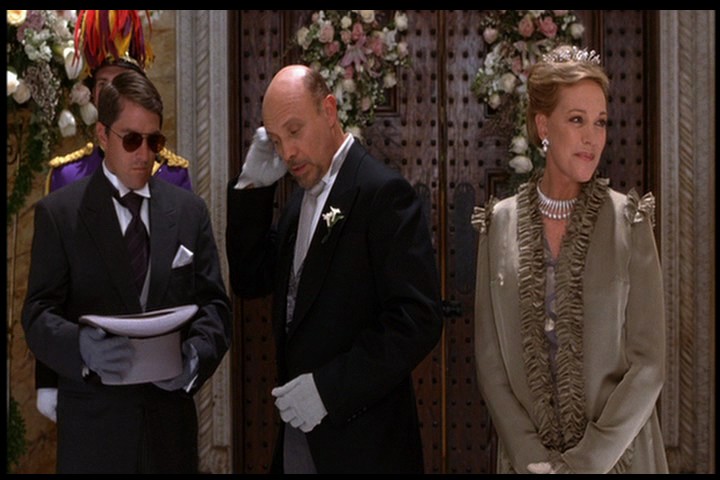

Supplement: S4 Dataset — It also includes pose data and camera parameters. (ZIP) [file pone.0264302.s004.zip › princess-diaries-2-00144441.jpg]

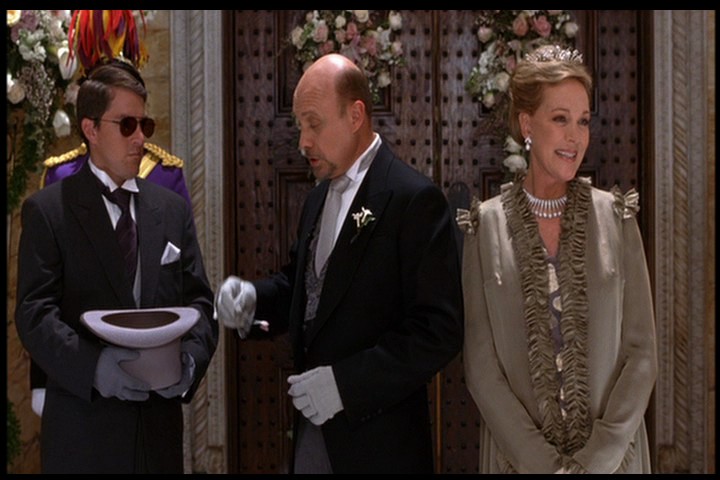

Supplement: S4 Dataset — It also includes pose data and camera parameters. (ZIP) [file pone.0264302.s004.zip › princess-diaries-2-00144461.jpg]

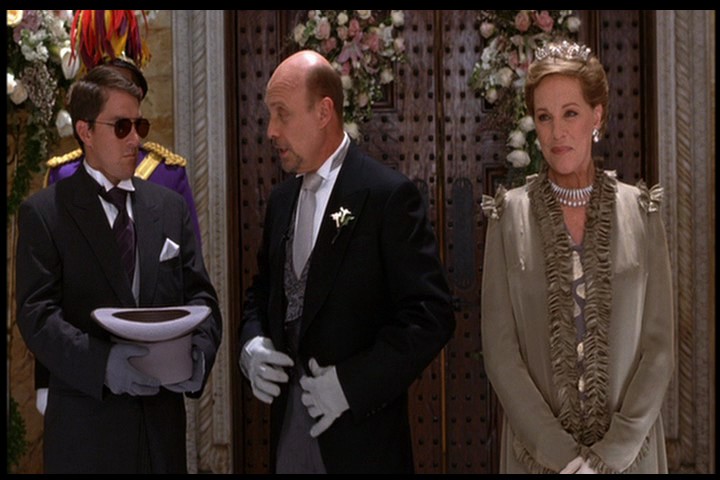

Supplement: S4 Dataset — It also includes pose data and camera parameters. (ZIP) [file pone.0264302.s004.zip › princess-diaries-2-00144501.jpg]

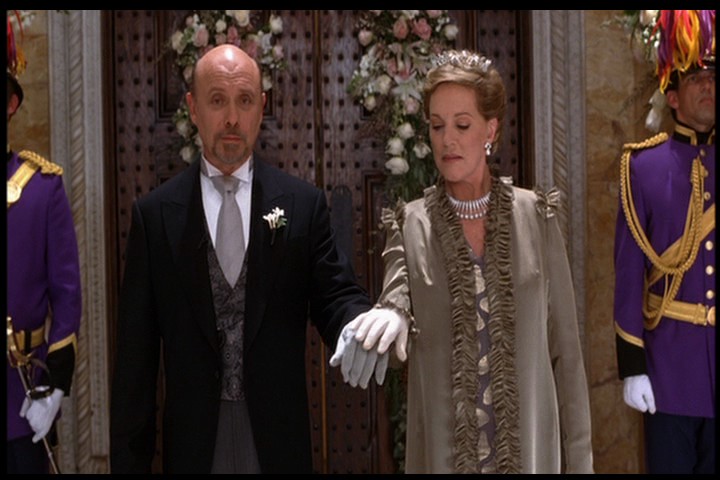

Supplement: S4 Dataset — It also includes pose data and camera parameters. (ZIP) [file pone.0264302.s004.zip › princess-diaries-2-00144651.jpg]

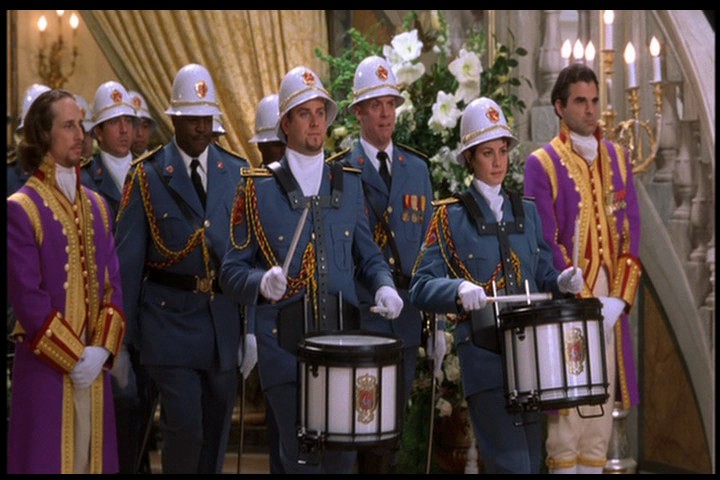

Supplement: S4 Dataset — It also includes pose data and camera parameters. (ZIP) [file pone.0264302.s004.zip › princess-diaries-2-00149771.jpg]

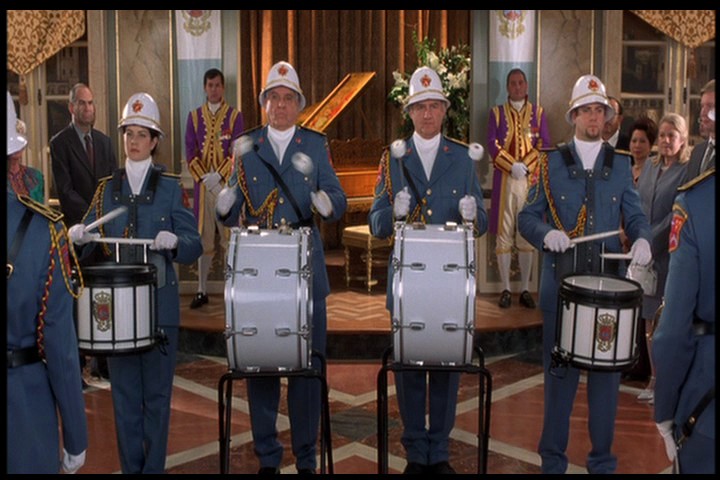

Supplement: S4 Dataset — It also includes pose data and camera parameters. (ZIP) [file pone.0264302.s004.zip › princess-diaries-2-00150111.jpg]

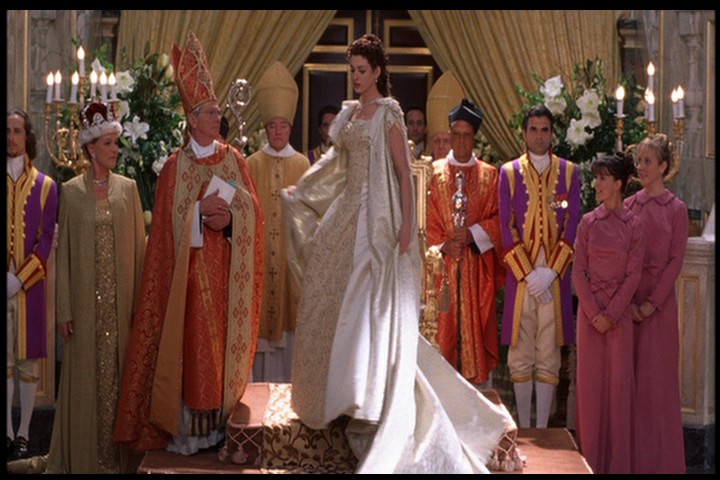

Supplement: S4 Dataset — It also includes pose data and camera parameters. (ZIP) [file pone.0264302.s004.zip › princess-diaries-2-00152091.jpg]

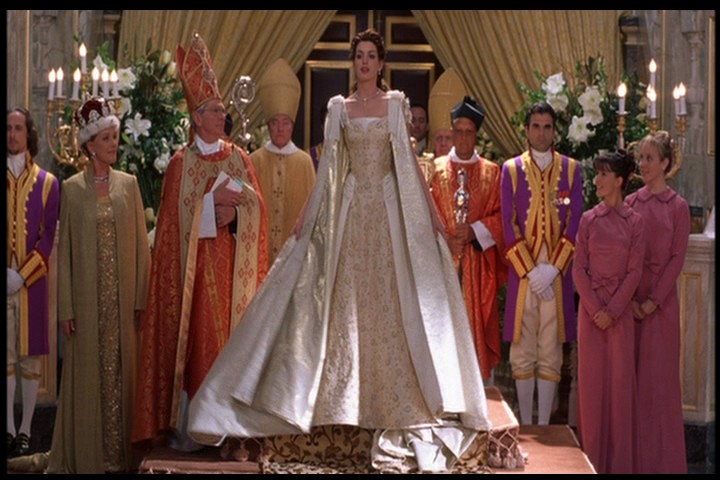

Supplement: S4 Dataset — It also includes pose data and camera parameters. (ZIP) [file pone.0264302.s004.zip › princess-diaries-2-00152121.jpg]

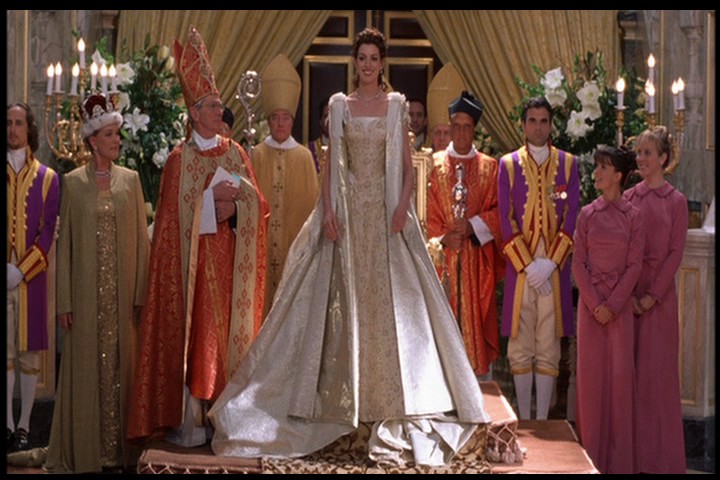

Supplement: S4 Dataset — It also includes pose data and camera parameters. (ZIP) [file pone.0264302.s004.zip › princess-diaries-2-00152181.jpg]

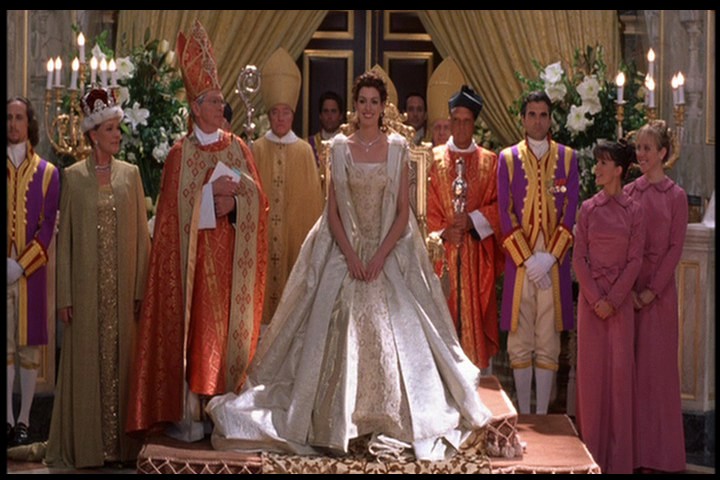

Supplement: S4 Dataset — It also includes pose data and camera parameters. (ZIP) [file pone.0264302.s004.zip › princess-diaries-2-00152201.jpg]

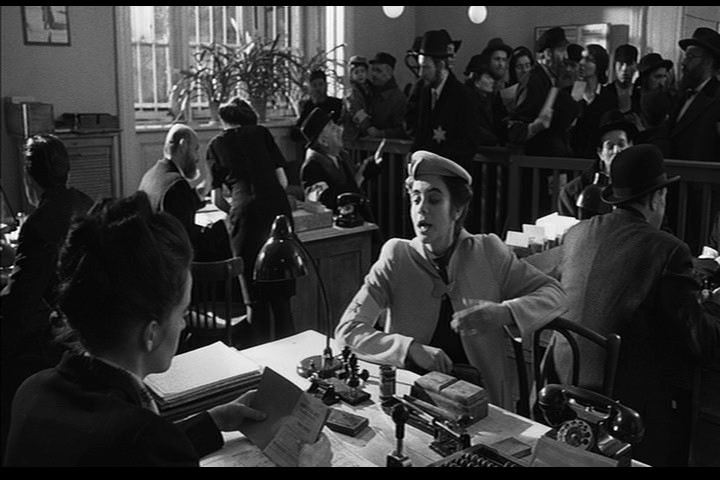

Supplement: S4 Dataset — It also includes pose data and camera parameters. (ZIP) [file pone.0264302.s004.zip › schindlers-list-00016881.jpg]

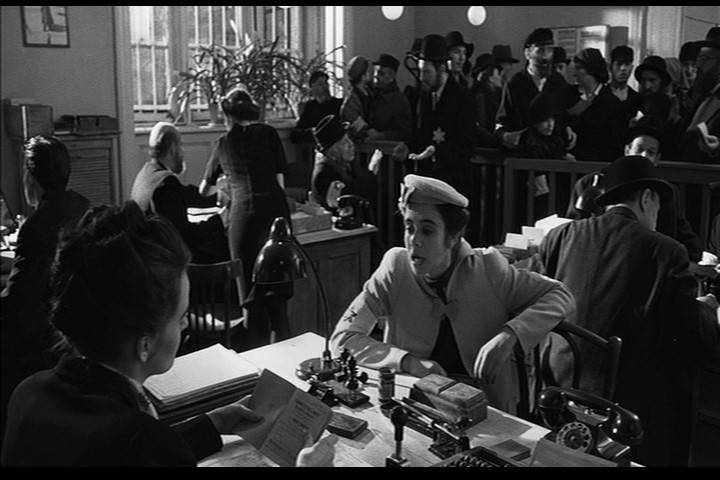

Supplement: S4 Dataset — It also includes pose data and camera parameters. (ZIP) [file pone.0264302.s004.zip › schindlers-list-00016971.jpg]

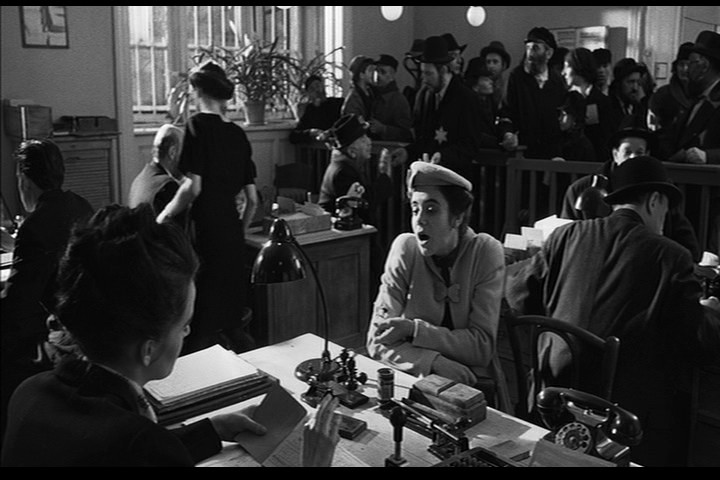

Supplement: S4 Dataset — It also includes pose data and camera parameters. (ZIP) [file pone.0264302.s004.zip › schindlers-list-00017011.jpg]

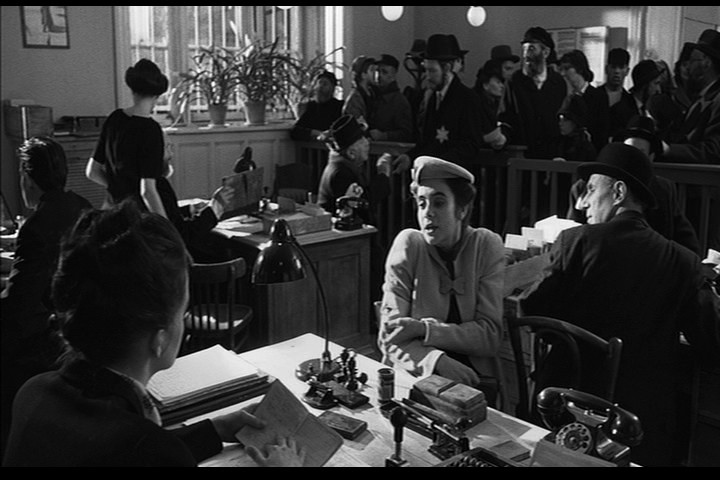

Supplement: S4 Dataset — It also includes pose data and camera parameters. (ZIP) [file pone.0264302.s004.zip › schindlers-list-00017041.jpg]
