# Supplementary figures and images for: LHPE-nets: A lightweight 2D and 3D human pose estimation model with well-structural deep networks and multi-view pose sample simplification method (part 7 of 8)
Source: PLoS One. 2022 Feb 23;17(2):e0264302. doi: 10.1371/journal.pone.0264302 (PMC8865690; doi:10.1371/journal.pone.0264302)

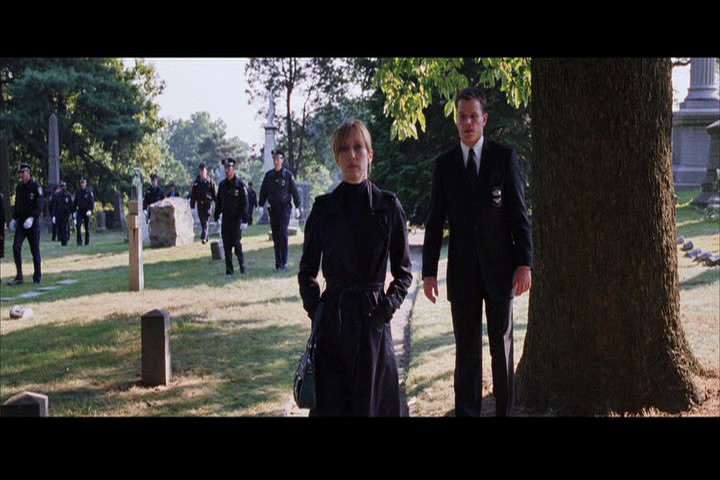

Supplement: S4 Dataset — It also includes pose data and camera parameters. (ZIP) [file pone.0264302.s004.zip › the-departed-00205861.jpg]

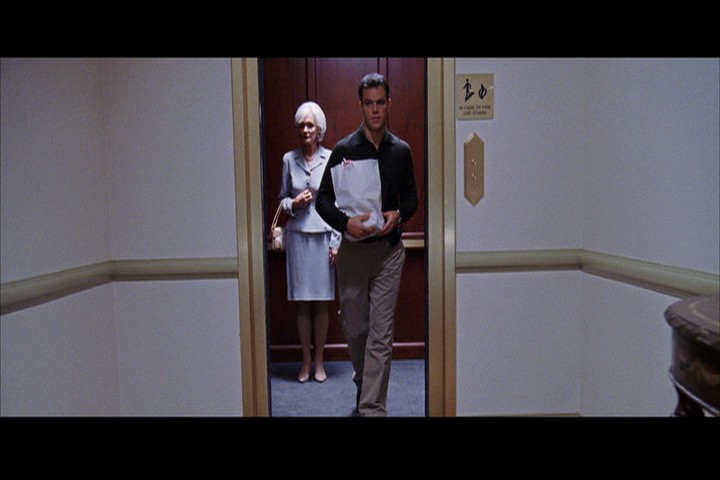

Supplement: S4 Dataset — It also includes pose data and camera parameters. (ZIP) [file pone.0264302.s004.zip › the-departed-00206221.jpg]

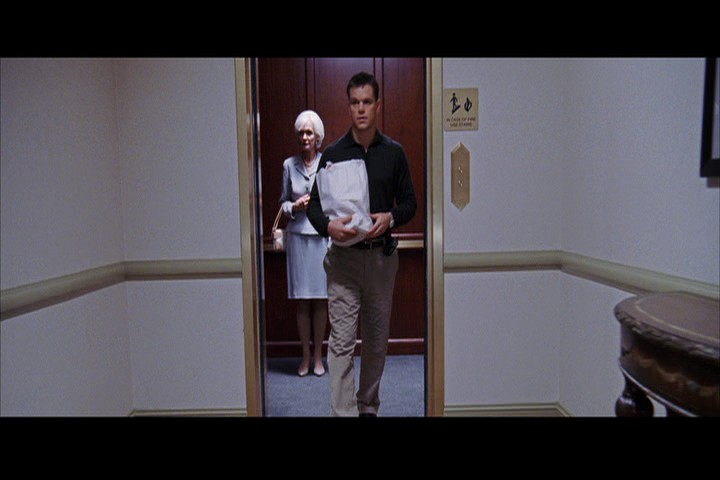

Supplement: S4 Dataset — It also includes pose data and camera parameters. (ZIP) [file pone.0264302.s004.zip › the-departed-00206231.jpg]

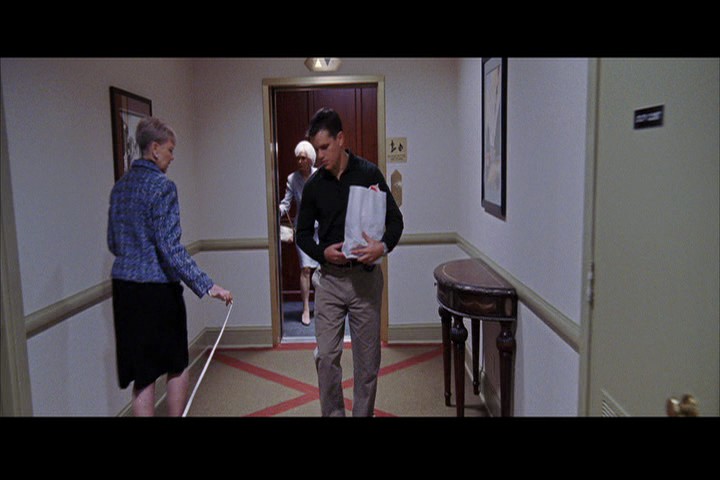

Supplement: S4 Dataset — It also includes pose data and camera parameters. (ZIP) [file pone.0264302.s004.zip › the-departed-00206291.jpg]

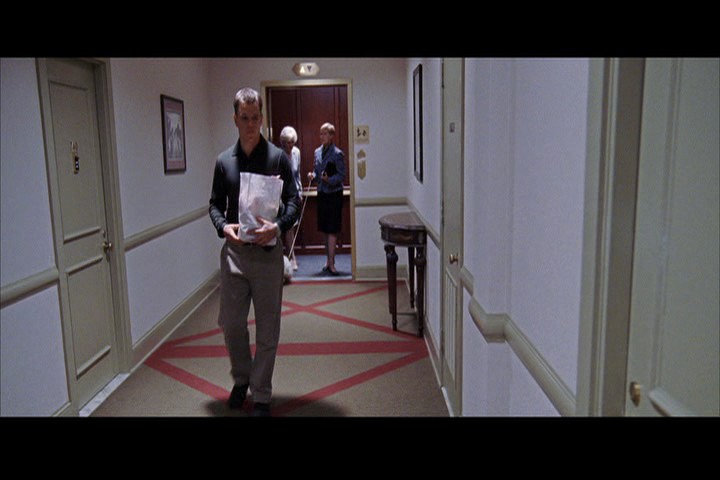

Supplement: S4 Dataset — It also includes pose data and camera parameters. (ZIP) [file pone.0264302.s004.zip › the-departed-00206401.jpg]

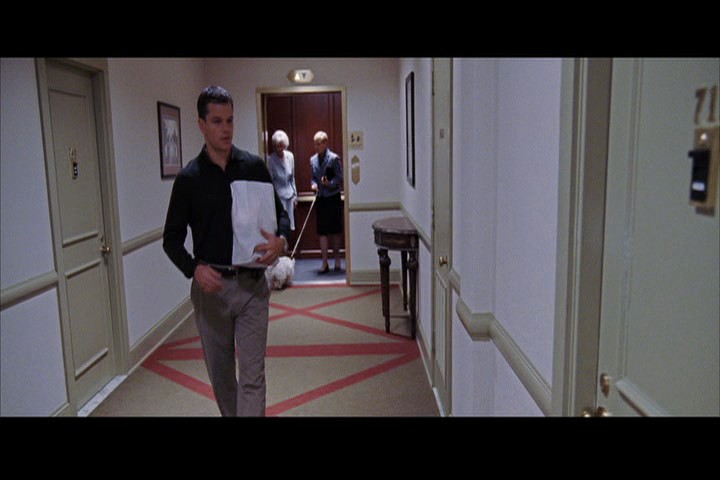

Supplement: S4 Dataset — It also includes pose data and camera parameters. (ZIP) [file pone.0264302.s004.zip › the-departed-00206421.jpg]

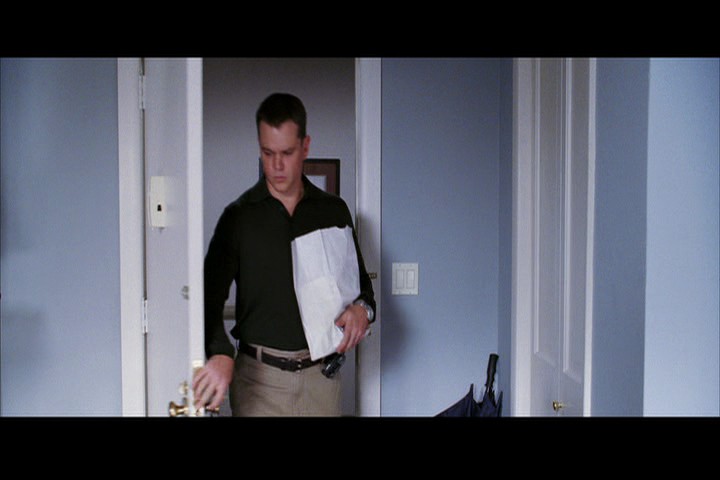

Supplement: S4 Dataset — It also includes pose data and camera parameters. (ZIP) [file pone.0264302.s004.zip › the-departed-00206781.jpg]

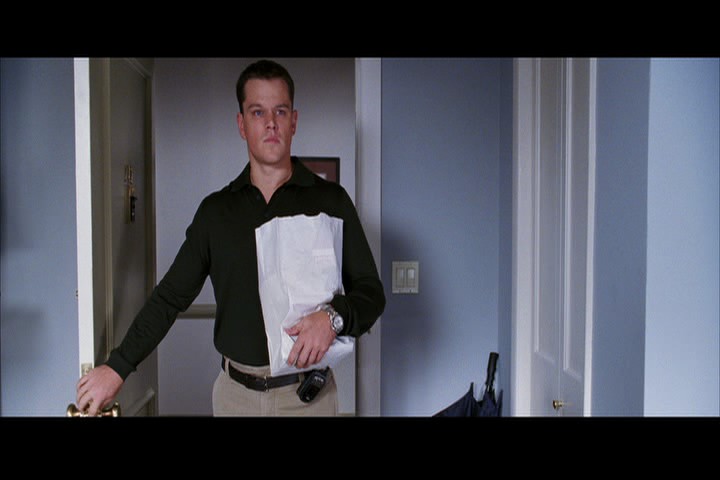

Supplement: S4 Dataset — It also includes pose data and camera parameters. (ZIP) [file pone.0264302.s004.zip › the-departed-00206801.jpg]

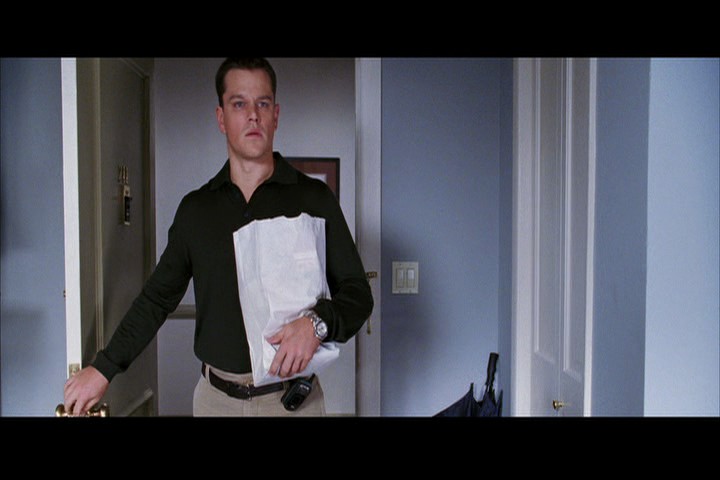

Supplement: S4 Dataset — It also includes pose data and camera parameters. (ZIP) [file pone.0264302.s004.zip › the-departed-00206821.jpg]

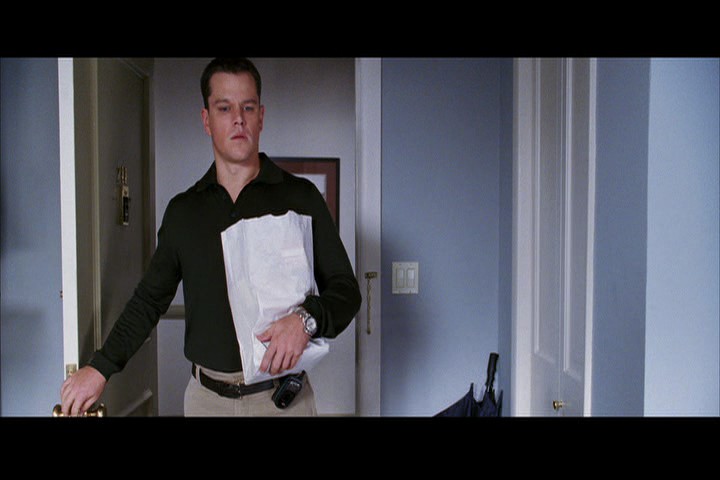

Supplement: S4 Dataset — It also includes pose data and camera parameters. (ZIP) [file pone.0264302.s004.zip › the-departed-00206841.jpg]

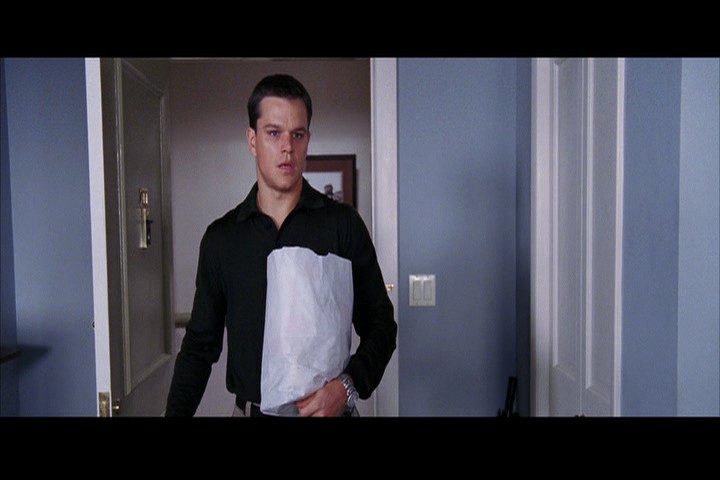

Supplement: S4 Dataset — It also includes pose data and camera parameters. (ZIP) [file pone.0264302.s004.zip › the-departed-00206901.jpg]

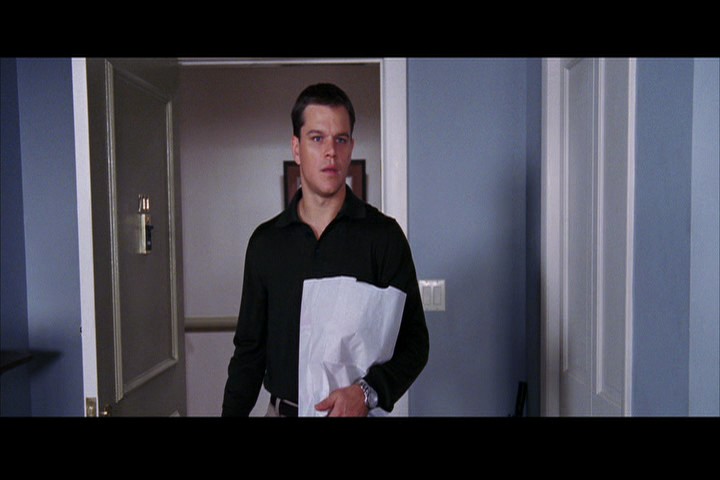

Supplement: S4 Dataset — It also includes pose data and camera parameters. (ZIP) [file pone.0264302.s004.zip › the-departed-00207061.jpg]

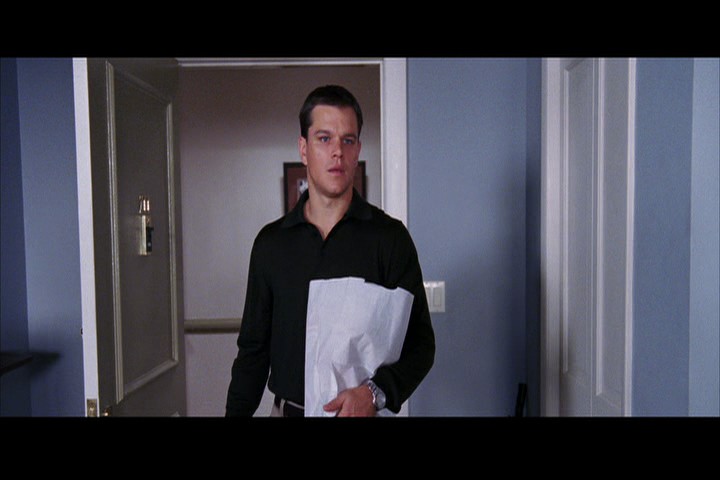

Supplement: S4 Dataset — It also includes pose data and camera parameters. (ZIP) [file pone.0264302.s004.zip › the-departed-00207071.jpg]

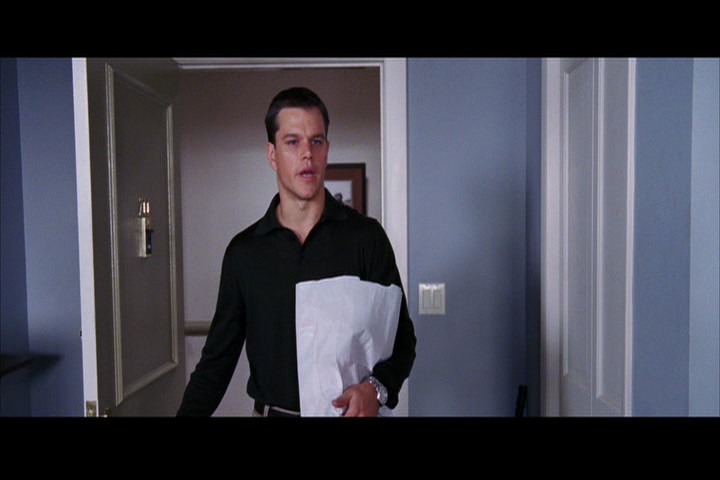

Supplement: S4 Dataset — It also includes pose data and camera parameters. (ZIP) [file pone.0264302.s004.zip › the-departed-00207091.jpg]

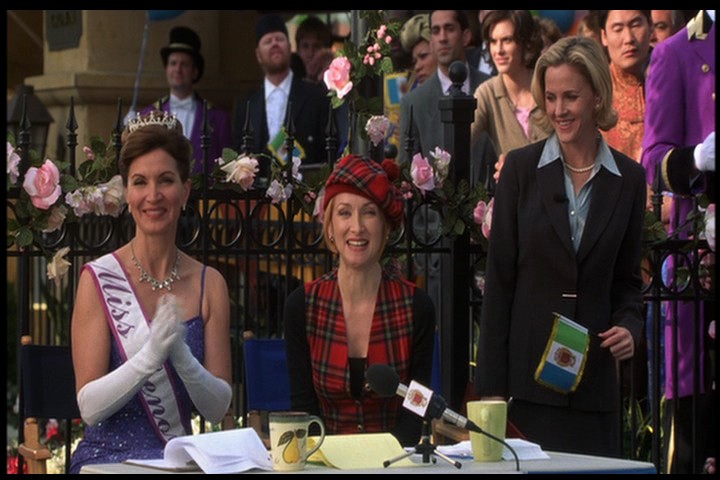

Supplement: S4 Dataset — It also includes pose data and camera parameters. (ZIP) [file pone.0264302.s004.zip › princess-diaries-2-00077571.jpg]

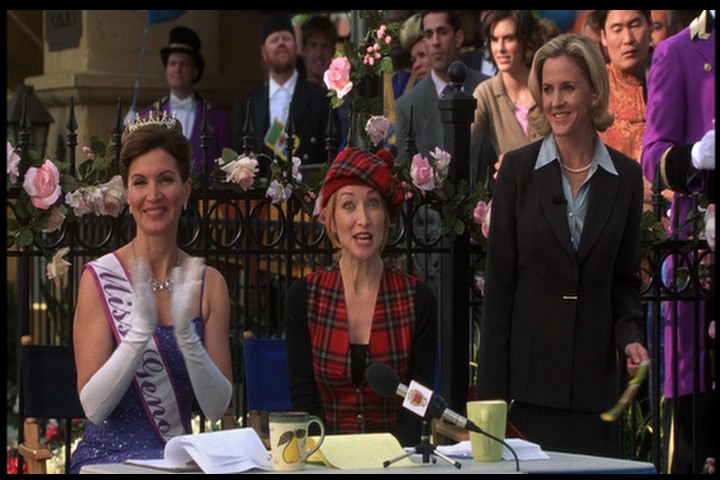

Supplement: S4 Dataset — It also includes pose data and camera parameters. (ZIP) [file pone.0264302.s004.zip › princess-diaries-2-00077581.jpg]

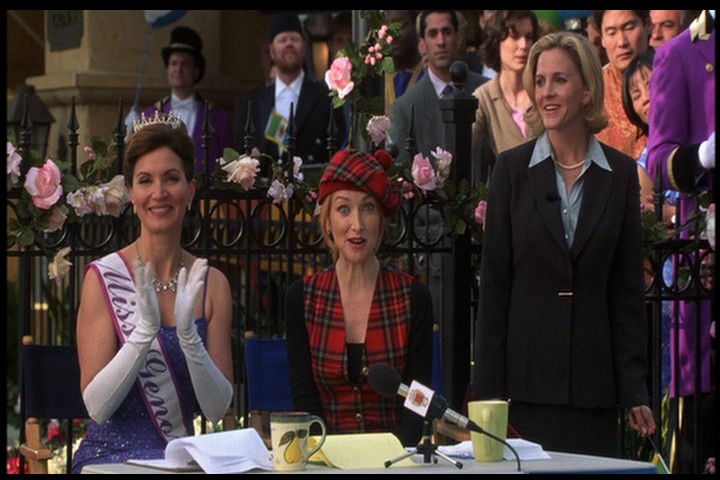

Supplement: S4 Dataset — It also includes pose data and camera parameters. (ZIP) [file pone.0264302.s004.zip › princess-diaries-2-00077601.jpg]

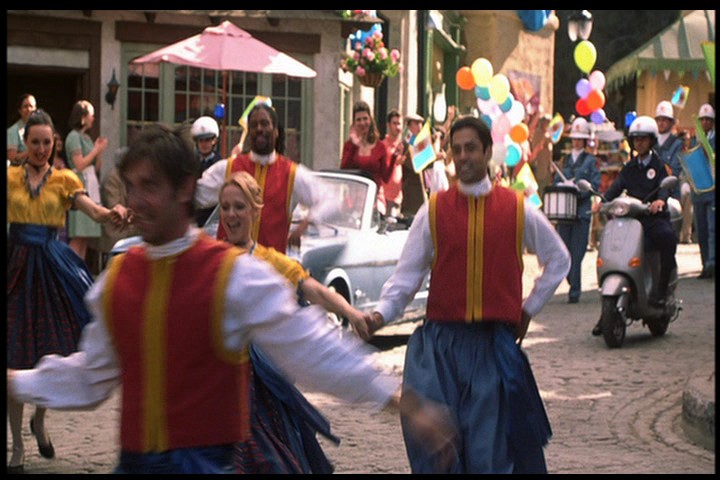

Supplement: S4 Dataset — It also includes pose data and camera parameters. (ZIP) [file pone.0264302.s004.zip › princess-diaries-2-00078271.jpg]

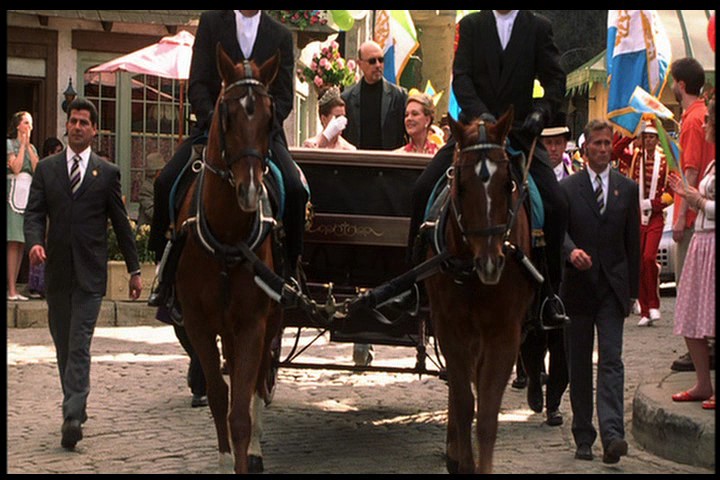

Supplement: S4 Dataset — It also includes pose data and camera parameters. (ZIP) [file pone.0264302.s004.zip › princess-diaries-2-00078411.jpg]

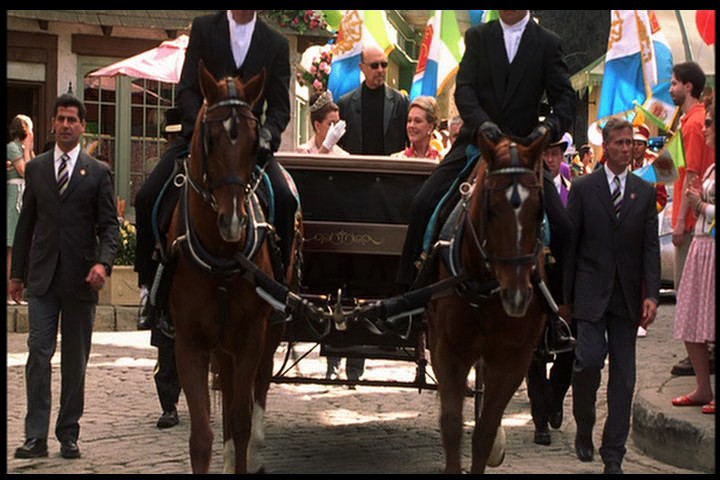

Supplement: S4 Dataset — It also includes pose data and camera parameters. (ZIP) [file pone.0264302.s004.zip › princess-diaries-2-00078421.jpg]

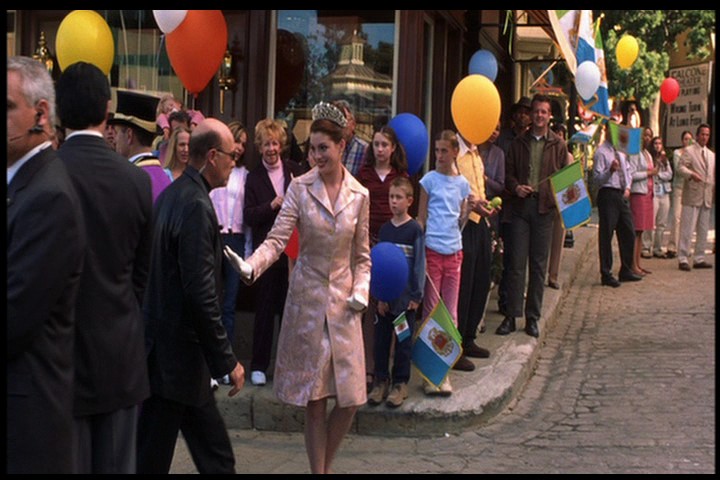

Supplement: S4 Dataset — It also includes pose data and camera parameters. (ZIP) [file pone.0264302.s004.zip › princess-diaries-2-00079041.jpg]

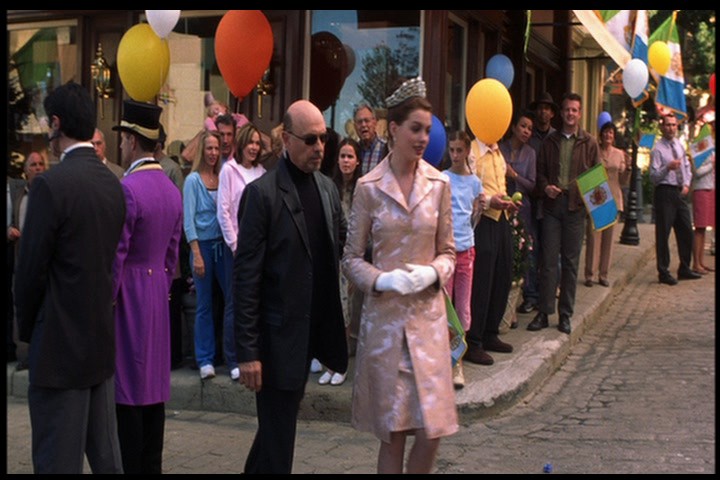

Supplement: S4 Dataset — It also includes pose data and camera parameters. (ZIP) [file pone.0264302.s004.zip › princess-diaries-2-00079071.jpg]

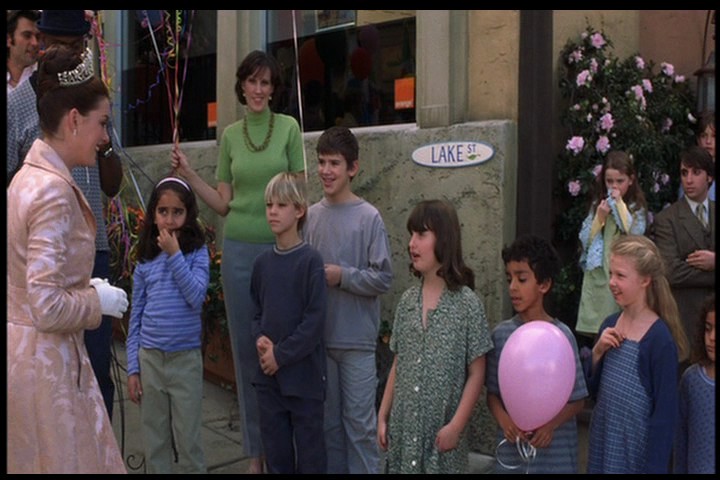

Supplement: S4 Dataset — It also includes pose data and camera parameters. (ZIP) [file pone.0264302.s004.zip › princess-diaries-2-00079211.jpg]

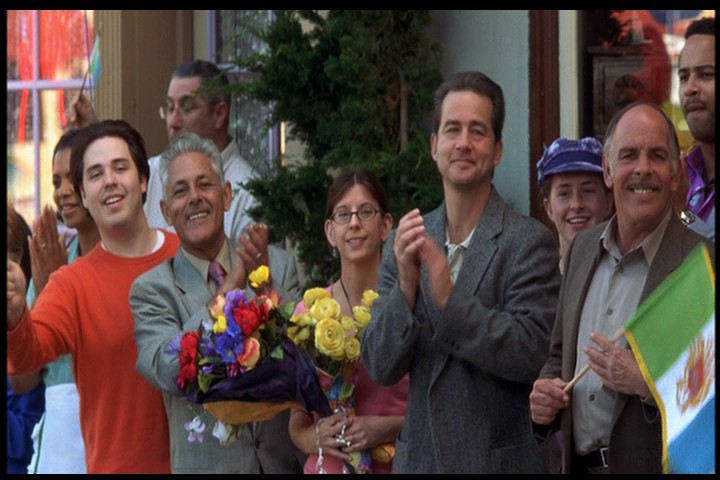

Supplement: S4 Dataset — It also includes pose data and camera parameters. (ZIP) [file pone.0264302.s004.zip › princess-diaries-2-00082441.jpg]

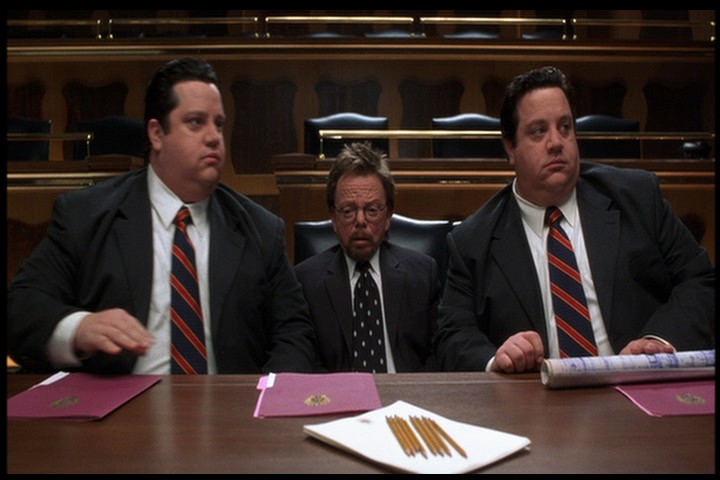

Supplement: S4 Dataset — It also includes pose data and camera parameters. (ZIP) [file pone.0264302.s004.zip › princess-diaries-2-00084871.jpg]

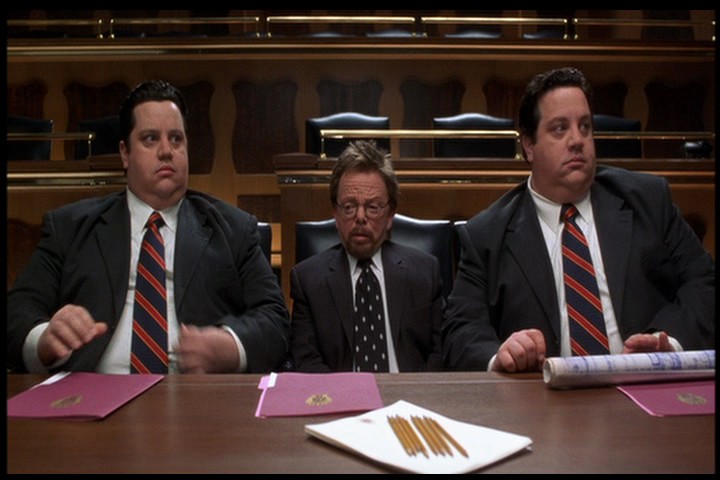

Supplement: S4 Dataset — It also includes pose data and camera parameters. (ZIP) [file pone.0264302.s004.zip › princess-diaries-2-00085001.jpg]

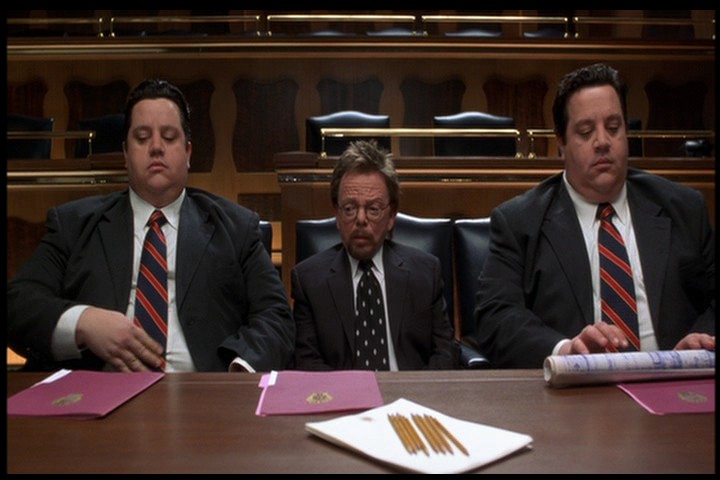

Supplement: S4 Dataset — It also includes pose data and camera parameters. (ZIP) [file pone.0264302.s004.zip › princess-diaries-2-00085021.jpg]

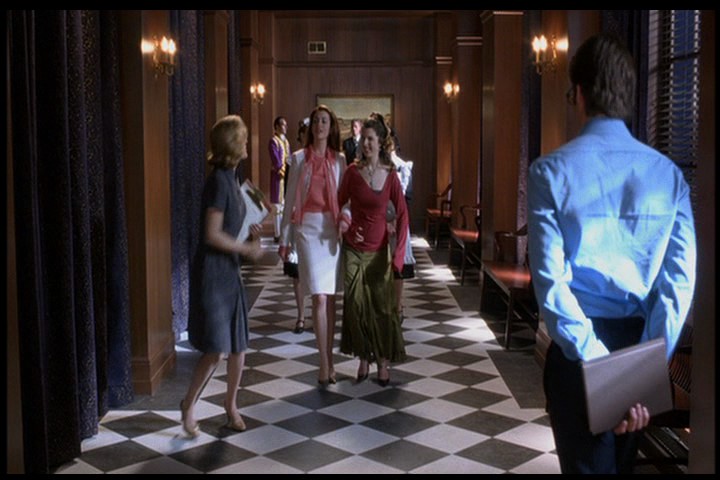

Supplement: S4 Dataset — It also includes pose data and camera parameters. (ZIP) [file pone.0264302.s004.zip › princess-diaries-2-00085201.jpg]

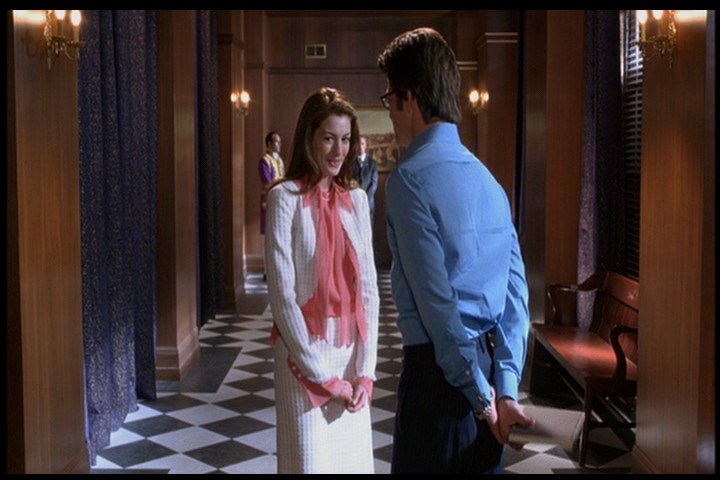

Supplement: S4 Dataset — It also includes pose data and camera parameters. (ZIP) [file pone.0264302.s004.zip › princess-diaries-2-00085931.jpg]

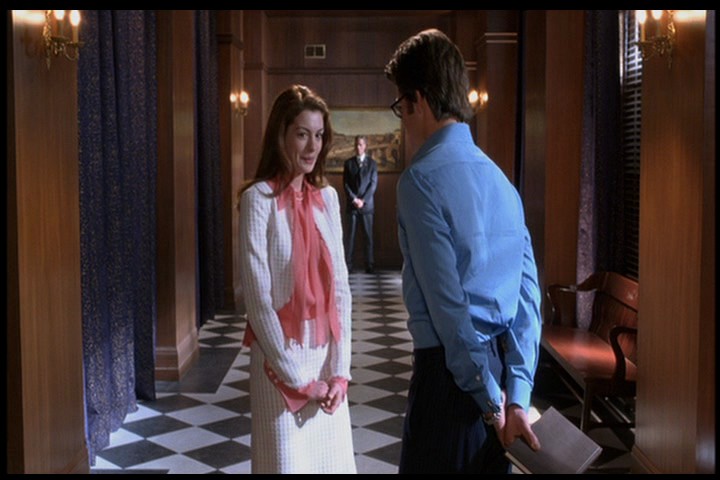

Supplement: S4 Dataset — It also includes pose data and camera parameters. (ZIP) [file pone.0264302.s004.zip › princess-diaries-2-00085961.jpg]

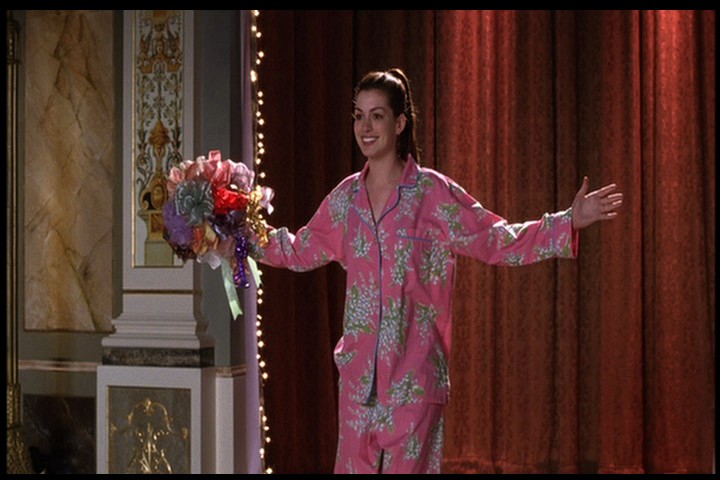

Supplement: S4 Dataset — It also includes pose data and camera parameters. (ZIP) [file pone.0264302.s004.zip › princess-diaries-2-00087521.jpg]

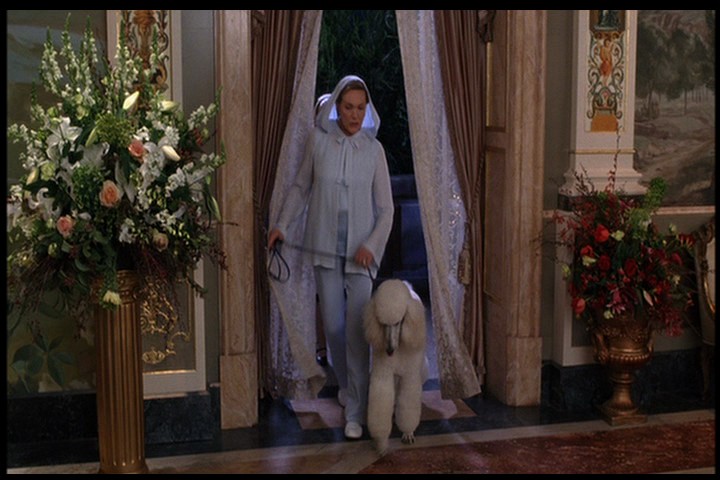

Supplement: S4 Dataset — It also includes pose data and camera parameters. (ZIP) [file pone.0264302.s004.zip › princess-diaries-2-00089391.jpg]

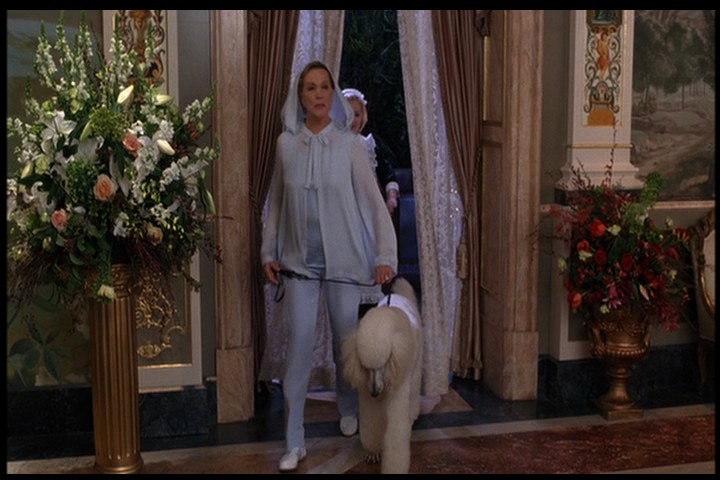

Supplement: S4 Dataset — It also includes pose data and camera parameters. (ZIP) [file pone.0264302.s004.zip › princess-diaries-2-00089401.jpg]

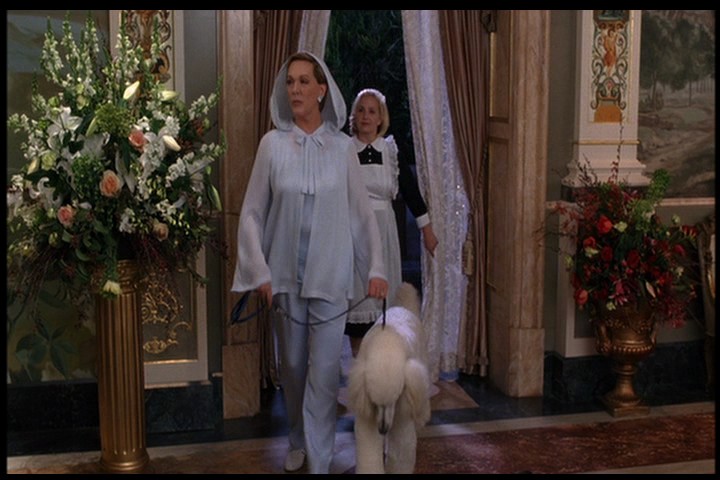

Supplement: S4 Dataset — It also includes pose data and camera parameters. (ZIP) [file pone.0264302.s004.zip › princess-diaries-2-00089411.jpg]

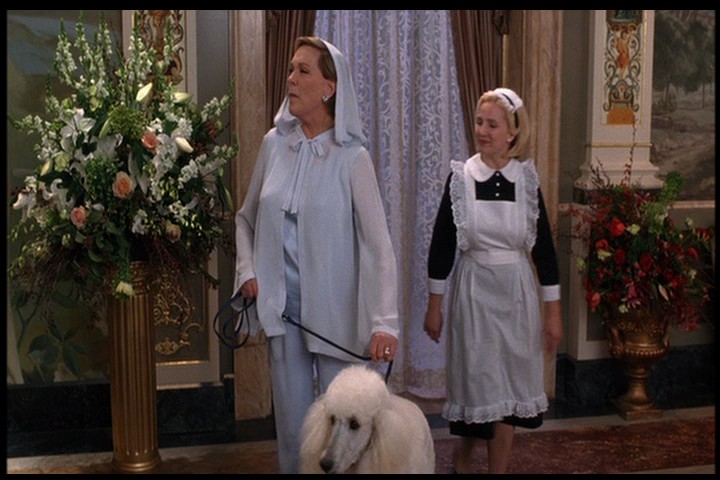

Supplement: S4 Dataset — It also includes pose data and camera parameters. (ZIP) [file pone.0264302.s004.zip › princess-diaries-2-00089451.jpg]

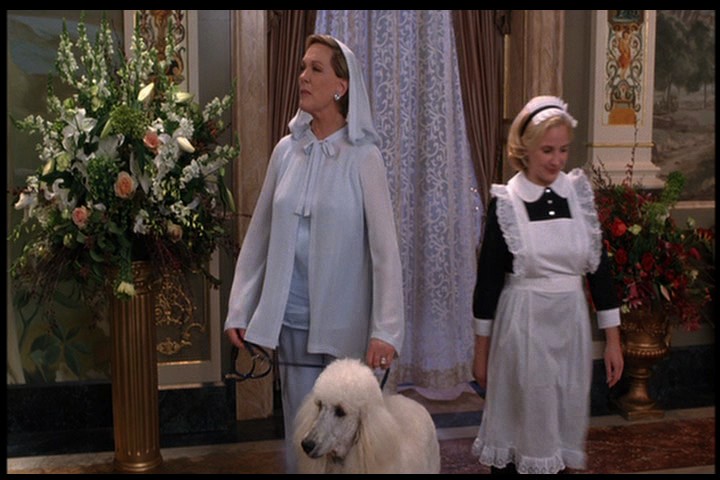

Supplement: S4 Dataset — It also includes pose data and camera parameters. (ZIP) [file pone.0264302.s004.zip › princess-diaries-2-00089461.jpg]

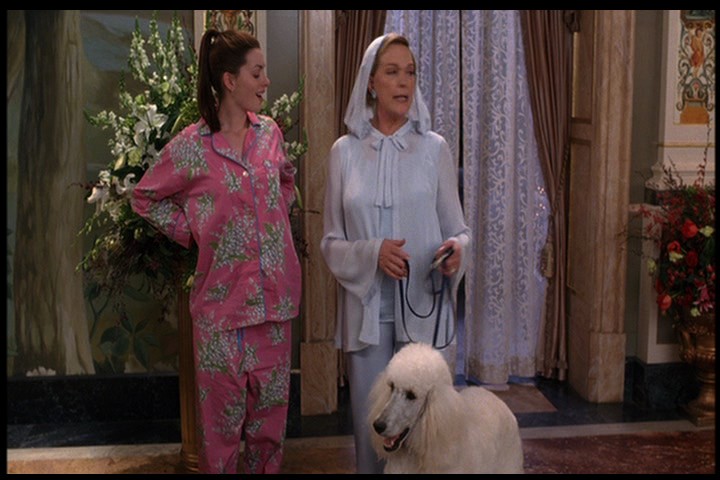

Supplement: S4 Dataset — It also includes pose data and camera parameters. (ZIP) [file pone.0264302.s004.zip › princess-diaries-2-00089861.jpg]

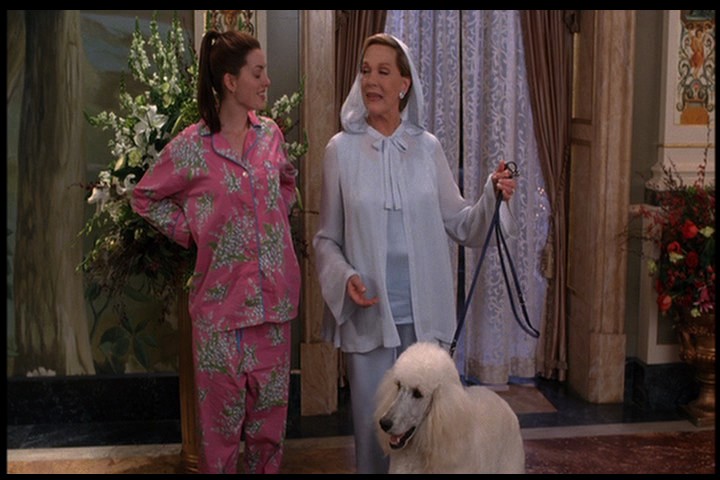

Supplement: S4 Dataset — It also includes pose data and camera parameters. (ZIP) [file pone.0264302.s004.zip › princess-diaries-2-00089881.jpg]

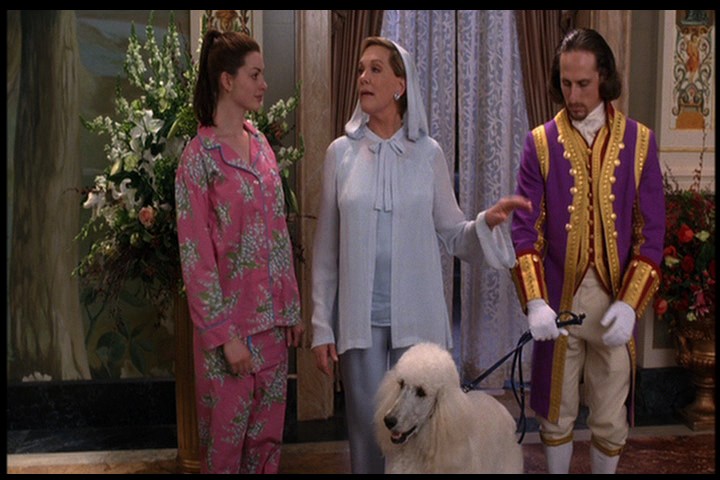

Supplement: S4 Dataset — It also includes pose data and camera parameters. (ZIP) [file pone.0264302.s004.zip › princess-diaries-2-00089961.jpg]

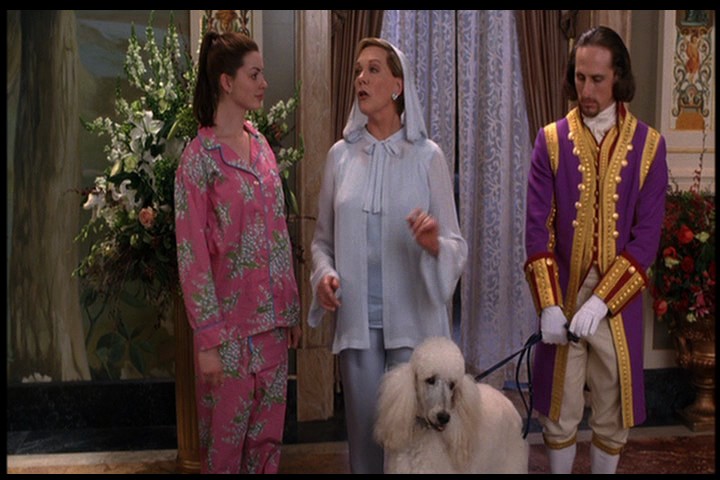

Supplement: S4 Dataset — It also includes pose data and camera parameters. (ZIP) [file pone.0264302.s004.zip › princess-diaries-2-00089971.jpg]

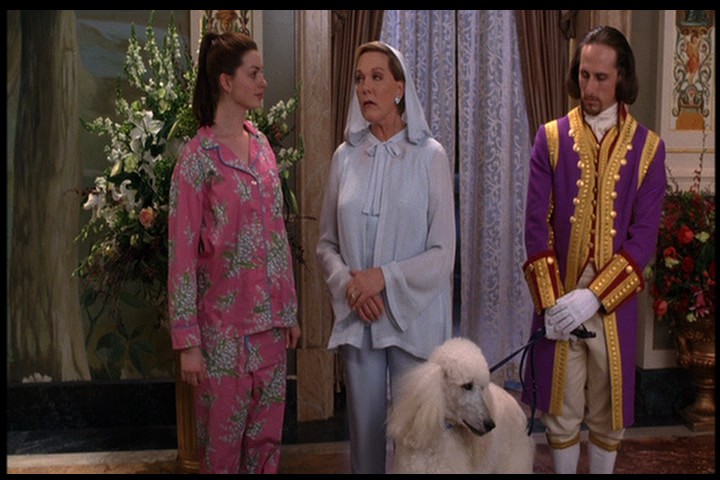

Supplement: S4 Dataset — It also includes pose data and camera parameters. (ZIP) [file pone.0264302.s004.zip › princess-diaries-2-00089981.jpg]

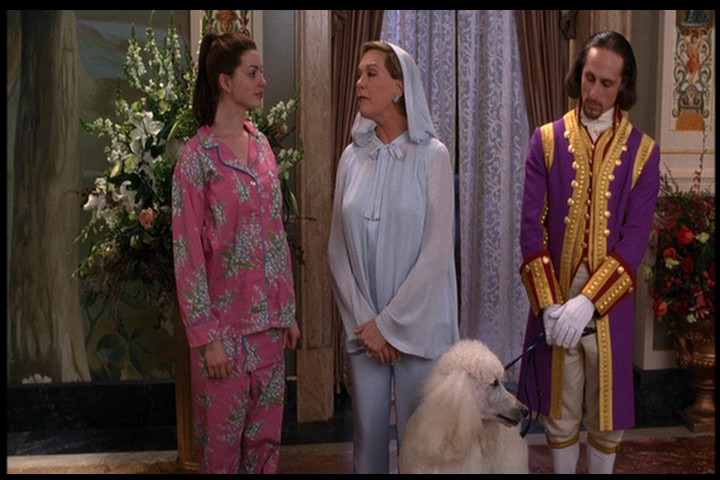

Supplement: S4 Dataset — It also includes pose data and camera parameters. (ZIP) [file pone.0264302.s004.zip › princess-diaries-2-00089991.jpg]

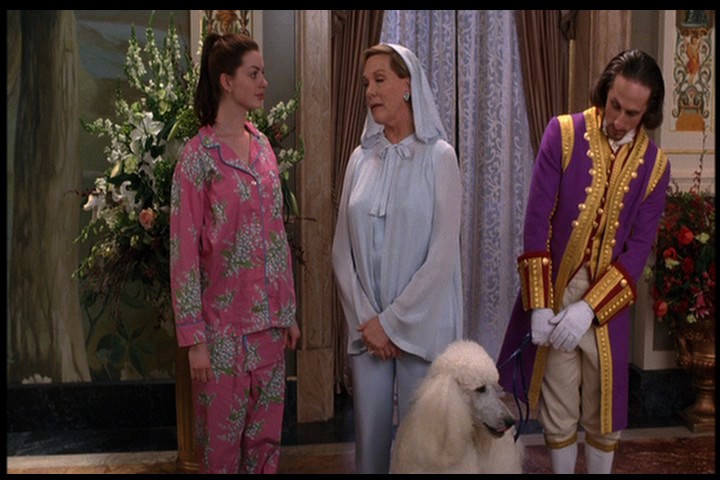

Supplement: S4 Dataset — It also includes pose data and camera parameters. (ZIP) [file pone.0264302.s004.zip › princess-diaries-2-00090001.jpg]

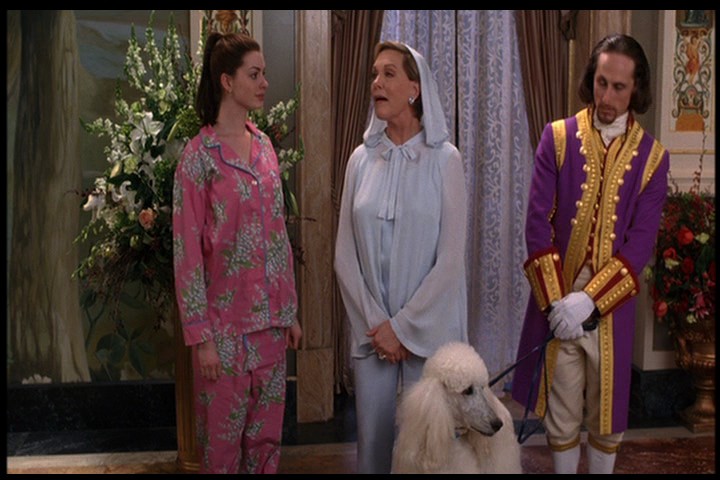

Supplement: S4 Dataset — It also includes pose data and camera parameters. (ZIP) [file pone.0264302.s004.zip › princess-diaries-2-00090011.jpg]

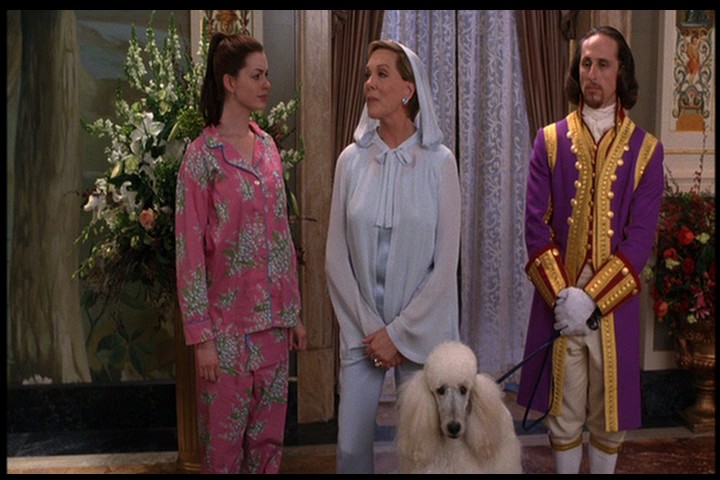

Supplement: S4 Dataset — It also includes pose data and camera parameters. (ZIP) [file pone.0264302.s004.zip › princess-diaries-2-00090021.jpg]

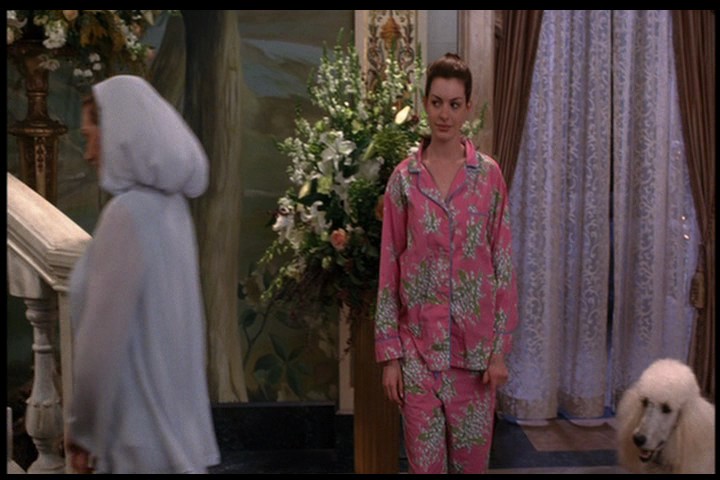

Supplement: S4 Dataset — It also includes pose data and camera parameters. (ZIP) [file pone.0264302.s004.zip › princess-diaries-2-00090061.jpg]

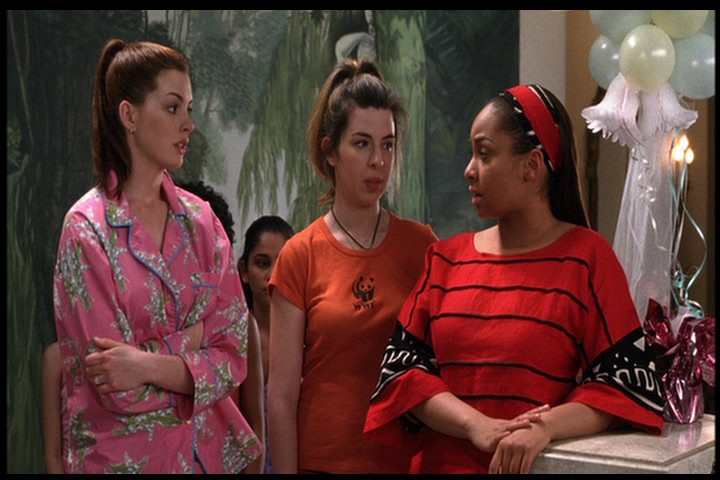

Supplement: S4 Dataset — It also includes pose data and camera parameters. (ZIP) [file pone.0264302.s004.zip › princess-diaries-2-00090161.jpg]

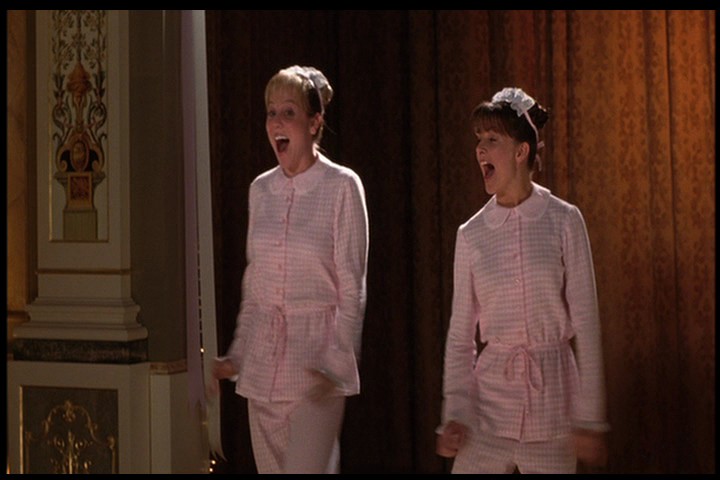

Supplement: S4 Dataset — It also includes pose data and camera parameters. (ZIP) [file pone.0264302.s004.zip › princess-diaries-2-00090651.jpg]

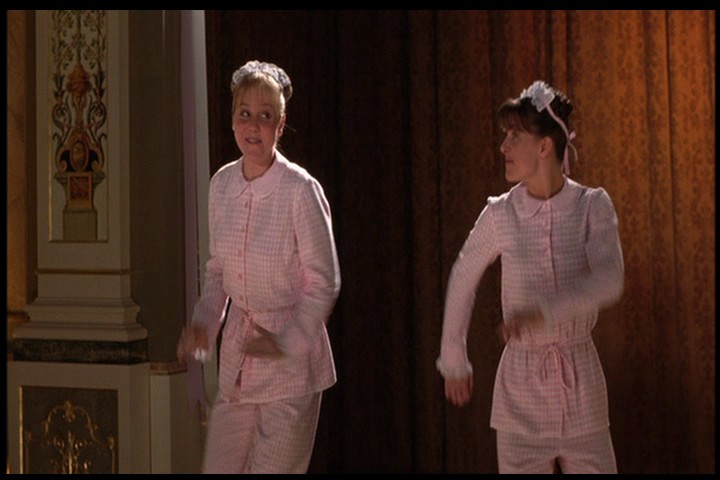

Supplement: S4 Dataset — It also includes pose data and camera parameters. (ZIP) [file pone.0264302.s004.zip › princess-diaries-2-00090661.jpg]

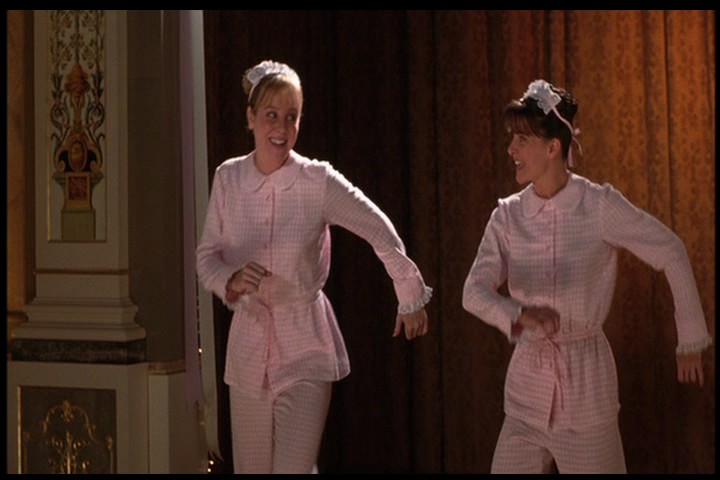

Supplement: S4 Dataset — It also includes pose data and camera parameters. (ZIP) [file pone.0264302.s004.zip › princess-diaries-2-00090671.jpg]

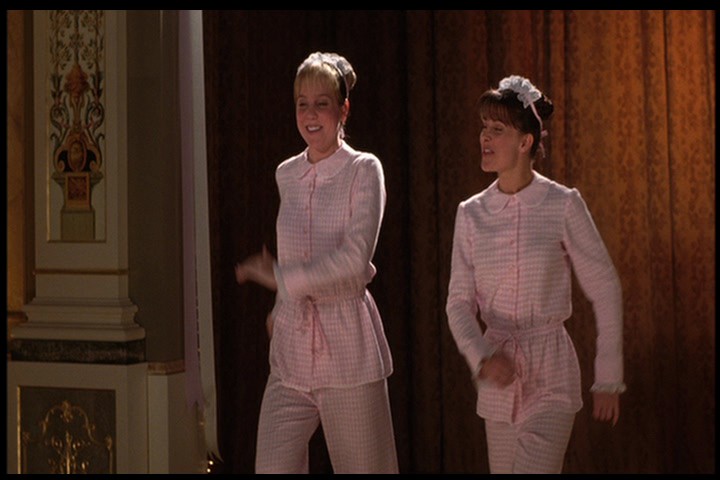

Supplement: S4 Dataset — It also includes pose data and camera parameters. (ZIP) [file pone.0264302.s004.zip › princess-diaries-2-00090681.jpg]

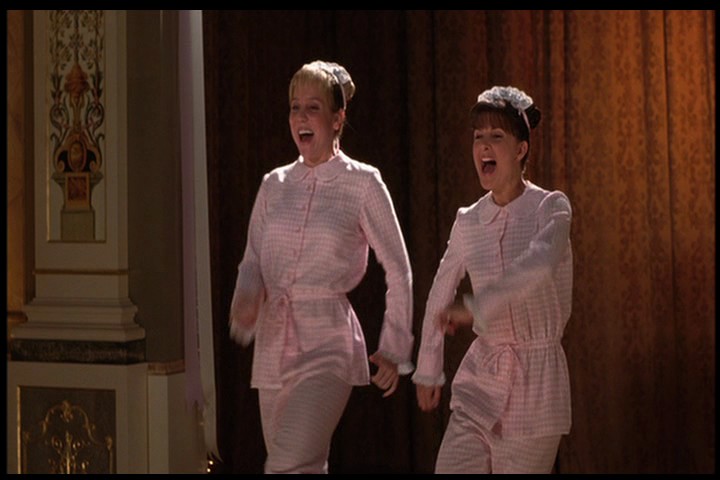

Supplement: S4 Dataset — It also includes pose data and camera parameters. (ZIP) [file pone.0264302.s004.zip › princess-diaries-2-00090691.jpg]

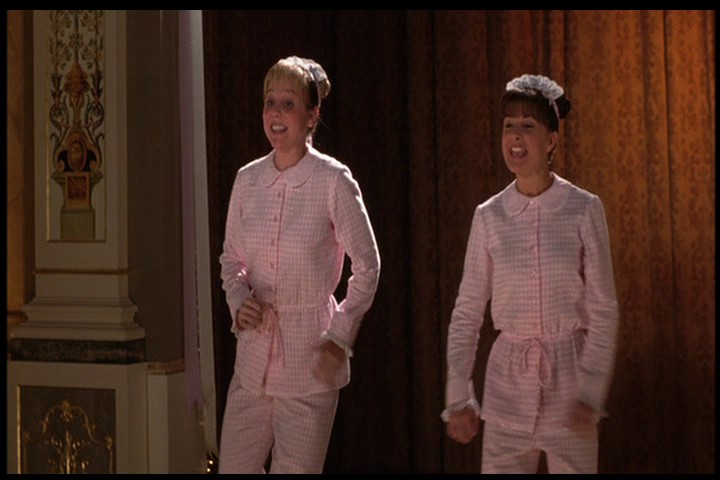

Supplement: S4 Dataset — It also includes pose data and camera parameters. (ZIP) [file pone.0264302.s004.zip › princess-diaries-2-00090701.jpg]

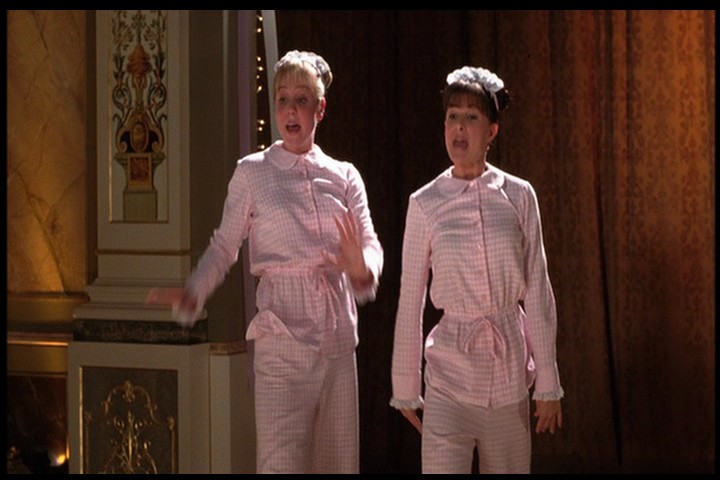

Supplement: S4 Dataset — It also includes pose data and camera parameters. (ZIP) [file pone.0264302.s004.zip › princess-diaries-2-00091021.jpg]

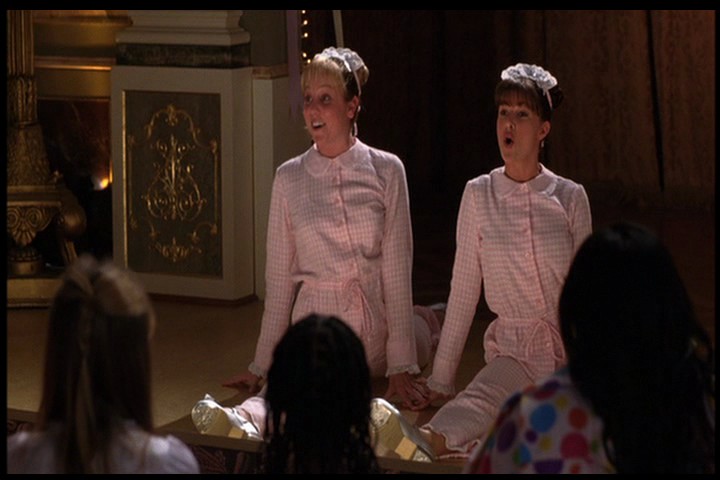

Supplement: S4 Dataset — It also includes pose data and camera parameters. (ZIP) [file pone.0264302.s004.zip › princess-diaries-2-00091061.jpg]

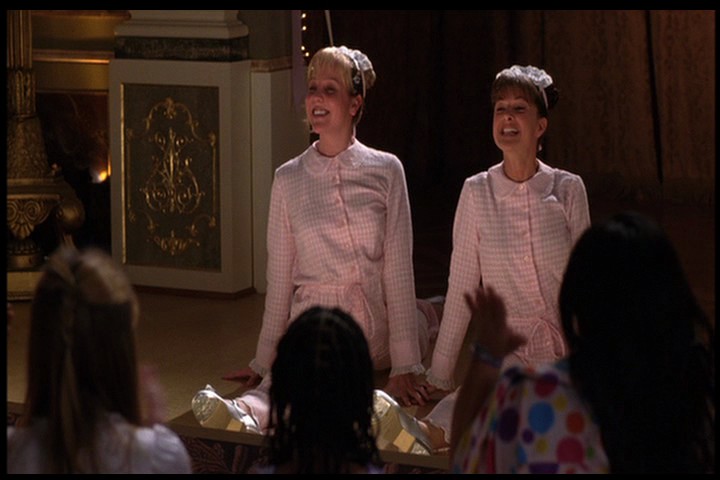

Supplement: S4 Dataset — It also includes pose data and camera parameters. (ZIP) [file pone.0264302.s004.zip › princess-diaries-2-00091081.jpg]

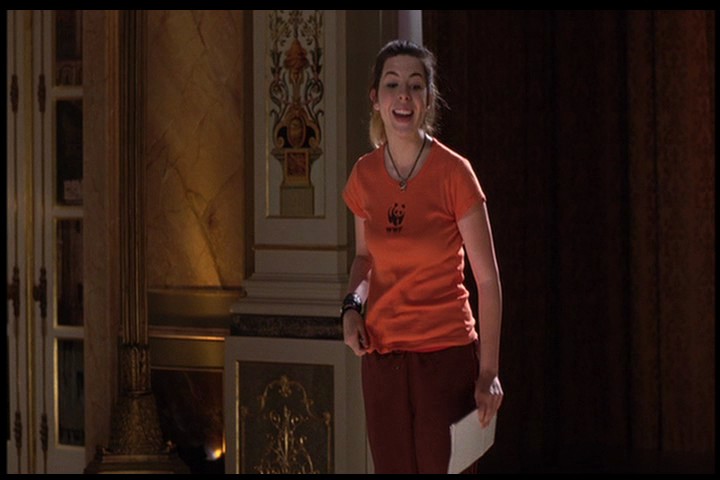

Supplement: S4 Dataset — It also includes pose data and camera parameters. (ZIP) [file pone.0264302.s004.zip › princess-diaries-2-00091201.jpg]

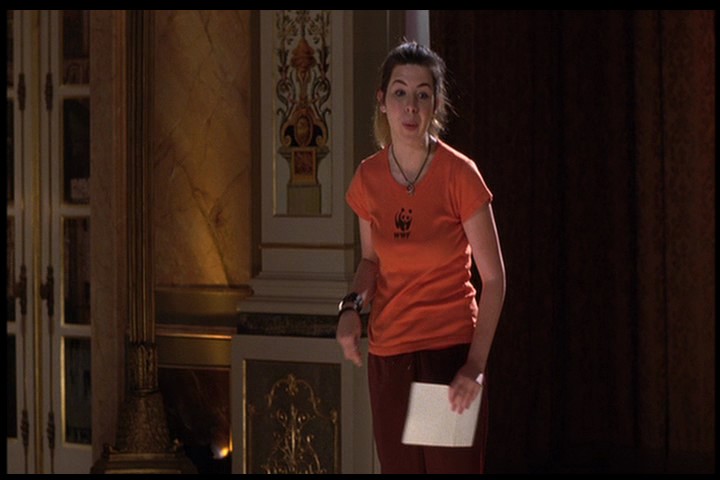

Supplement: S4 Dataset — It also includes pose data and camera parameters. (ZIP) [file pone.0264302.s004.zip › princess-diaries-2-00091211.jpg]

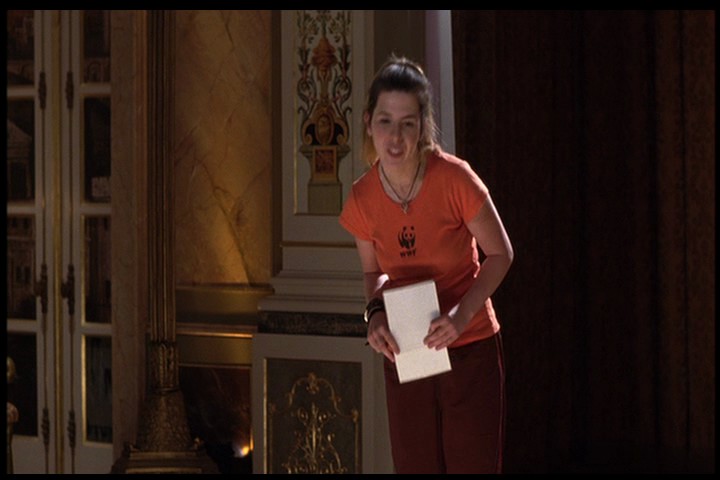

Supplement: S4 Dataset — It also includes pose data and camera parameters. (ZIP) [file pone.0264302.s004.zip › princess-diaries-2-00091291.jpg]

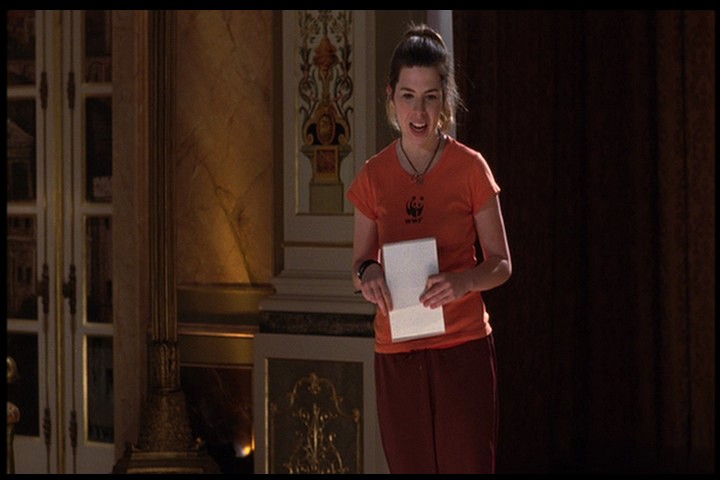

Supplement: S4 Dataset — It also includes pose data and camera parameters. (ZIP) [file pone.0264302.s004.zip › princess-diaries-2-00091301.jpg]

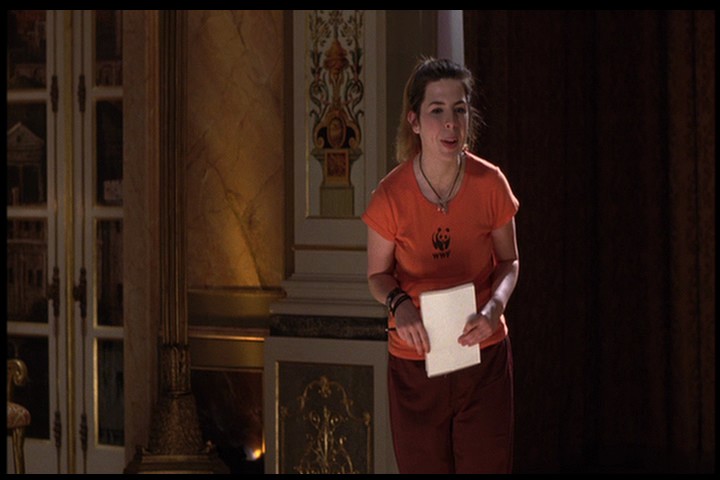

Supplement: S4 Dataset — It also includes pose data and camera parameters. (ZIP) [file pone.0264302.s004.zip › princess-diaries-2-00091351.jpg]

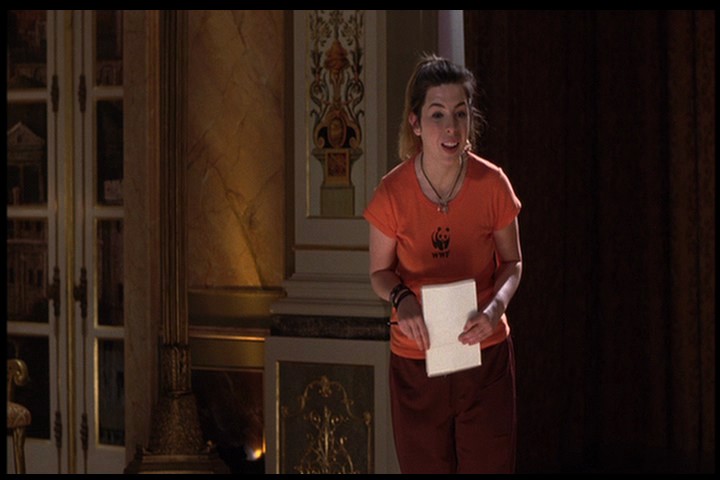

Supplement: S4 Dataset — It also includes pose data and camera parameters. (ZIP) [file pone.0264302.s004.zip › princess-diaries-2-00091361.jpg]

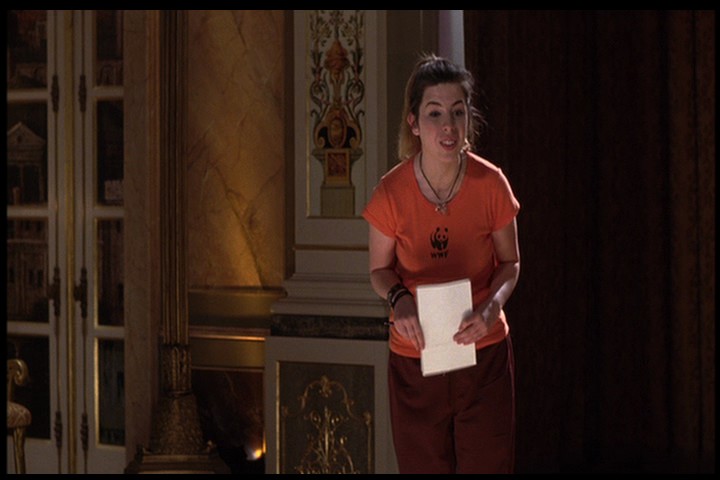

Supplement: S4 Dataset — It also includes pose data and camera parameters. (ZIP) [file pone.0264302.s004.zip › princess-diaries-2-00091371.jpg]

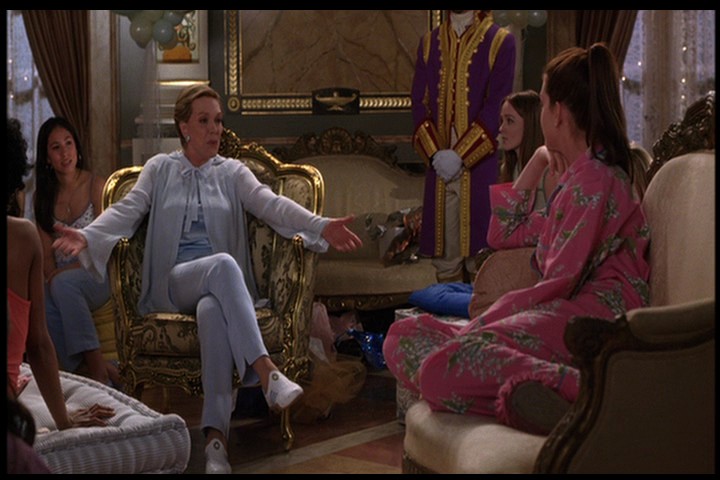

Supplement: S4 Dataset — It also includes pose data and camera parameters. (ZIP) [file pone.0264302.s004.zip › princess-diaries-2-00091631.jpg]

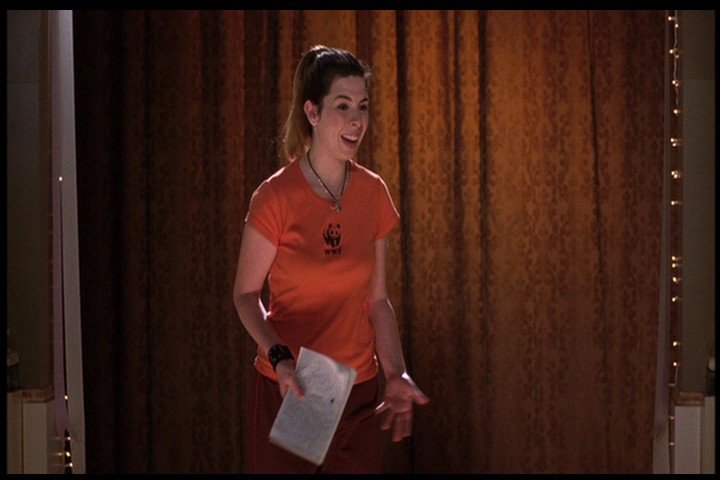

Supplement: S4 Dataset — It also includes pose data and camera parameters. (ZIP) [file pone.0264302.s004.zip › princess-diaries-2-00091681.jpg]

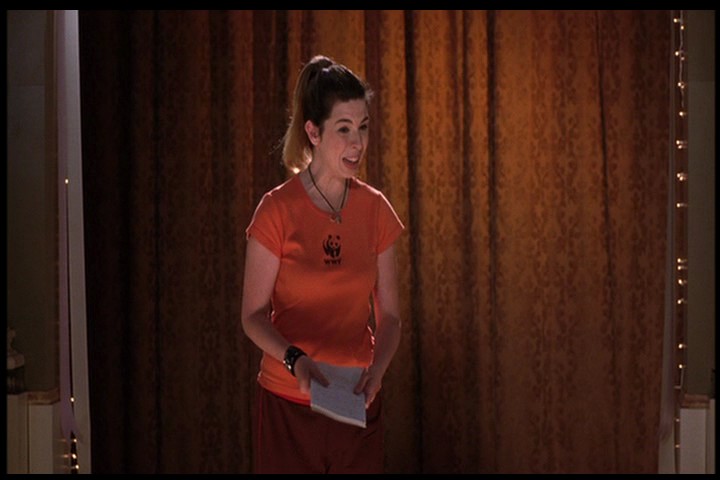

Supplement: S4 Dataset — It also includes pose data and camera parameters. (ZIP) [file pone.0264302.s004.zip › princess-diaries-2-00091691.jpg]

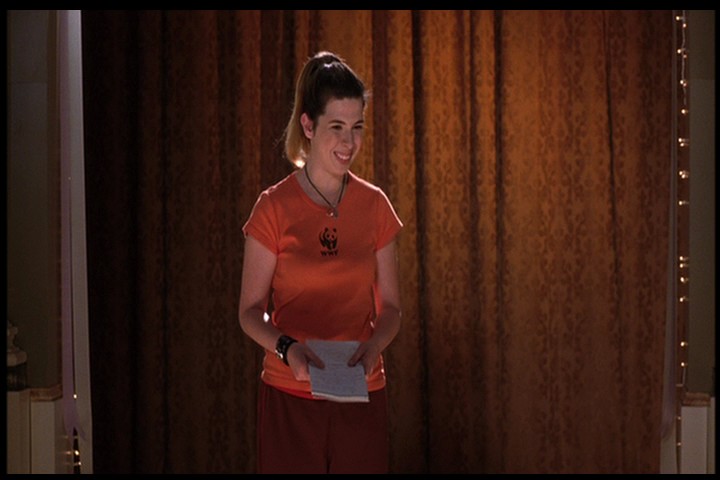

Supplement: S4 Dataset — It also includes pose data and camera parameters. (ZIP) [file pone.0264302.s004.zip › princess-diaries-2-00091711.jpg]

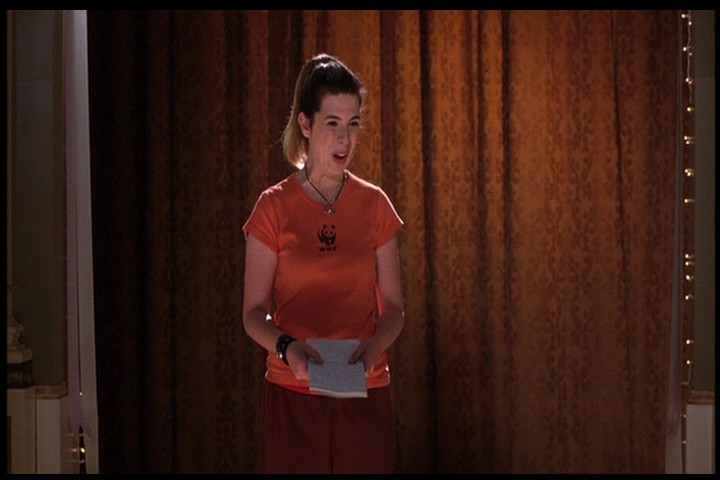

Supplement: S4 Dataset — It also includes pose data and camera parameters. (ZIP) [file pone.0264302.s004.zip › princess-diaries-2-00091721.jpg]

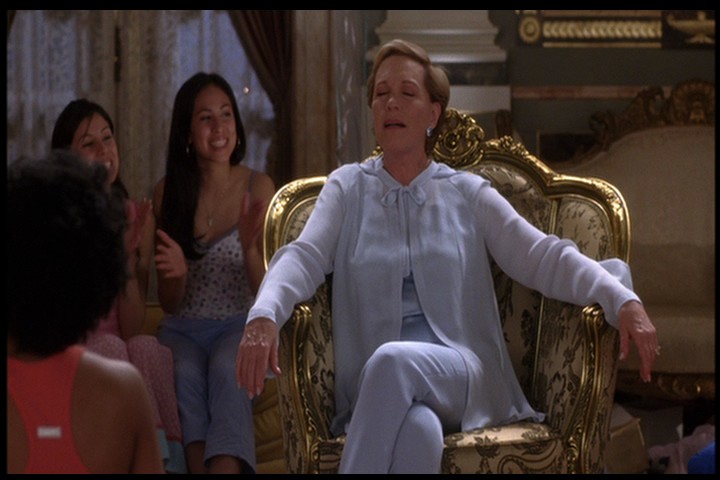

Supplement: S4 Dataset — It also includes pose data and camera parameters. (ZIP) [file pone.0264302.s004.zip › princess-diaries-2-00091831.jpg]

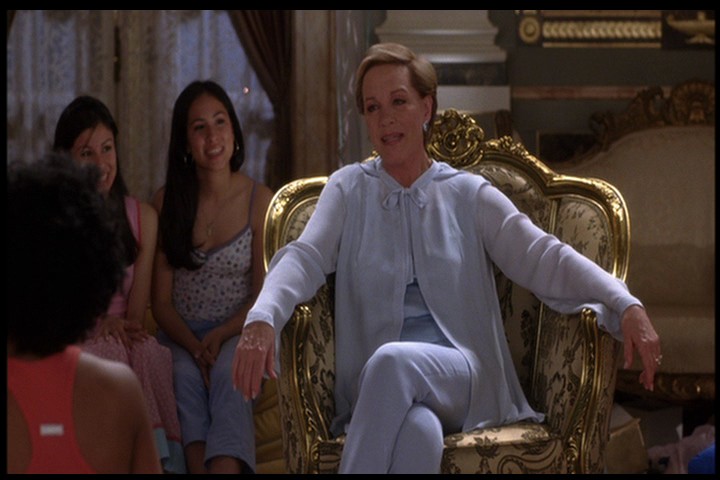

Supplement: S4 Dataset — It also includes pose data and camera parameters. (ZIP) [file pone.0264302.s004.zip › princess-diaries-2-00091901.jpg]

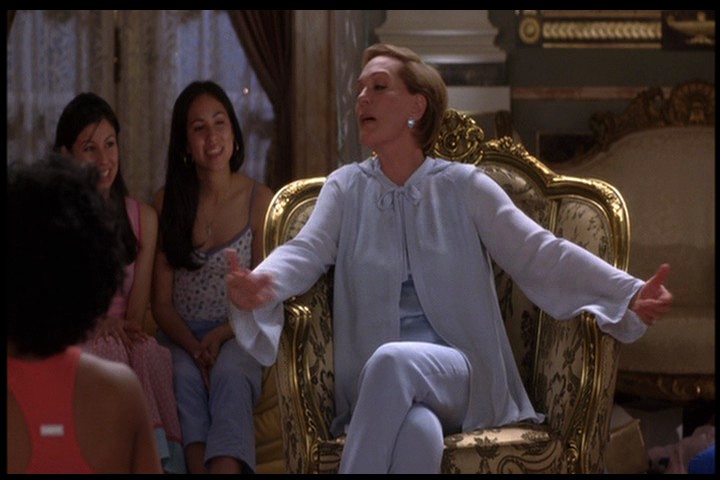

Supplement: S4 Dataset — It also includes pose data and camera parameters. (ZIP) [file pone.0264302.s004.zip › princess-diaries-2-00091911.jpg]

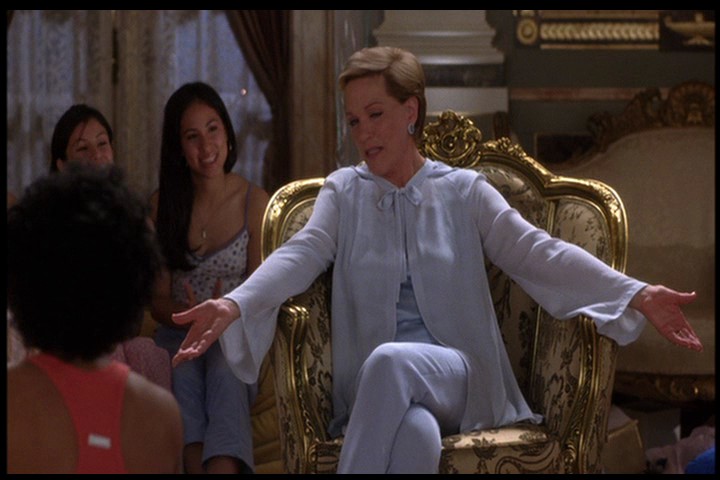

Supplement: S4 Dataset — It also includes pose data and camera parameters. (ZIP) [file pone.0264302.s004.zip › princess-diaries-2-00091961.jpg]

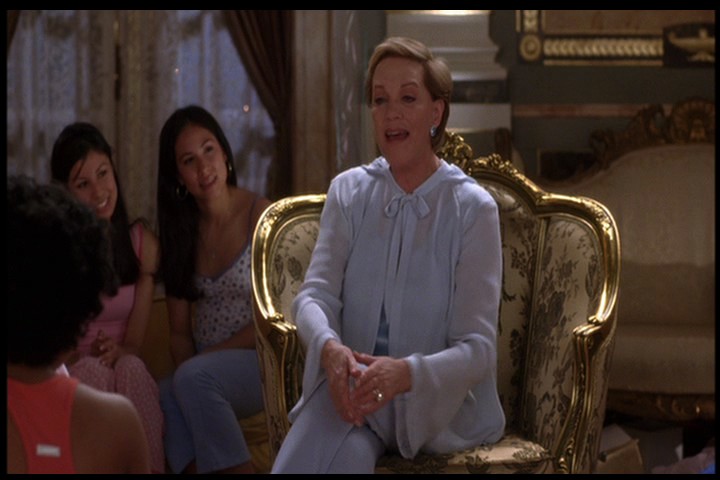

Supplement: S4 Dataset — It also includes pose data and camera parameters. (ZIP) [file pone.0264302.s004.zip › princess-diaries-2-00092301.jpg]

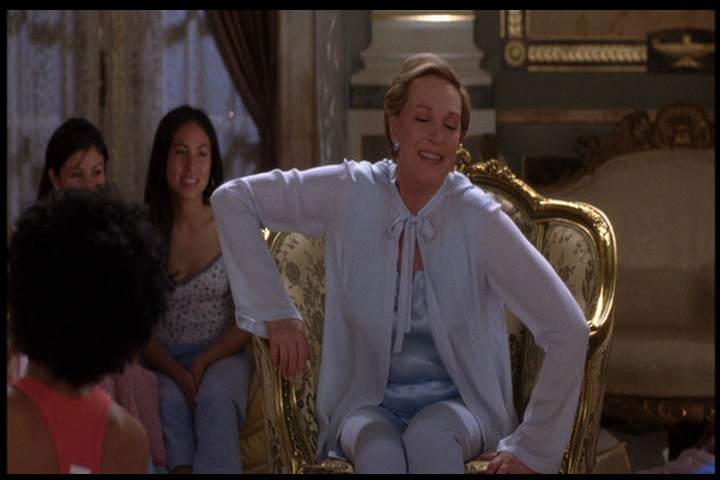

Supplement: S4 Dataset — It also includes pose data and camera parameters. (ZIP) [file pone.0264302.s004.zip › princess-diaries-2-00092391.jpg]

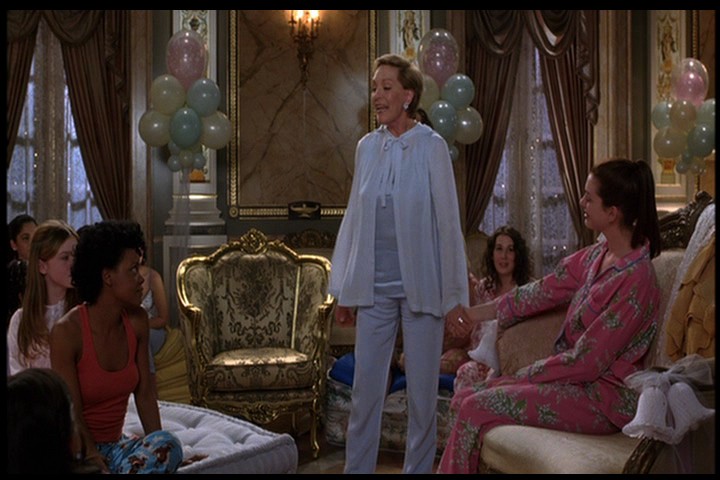

Supplement: S4 Dataset — It also includes pose data and camera parameters. (ZIP) [file pone.0264302.s004.zip › princess-diaries-2-00092831.jpg]

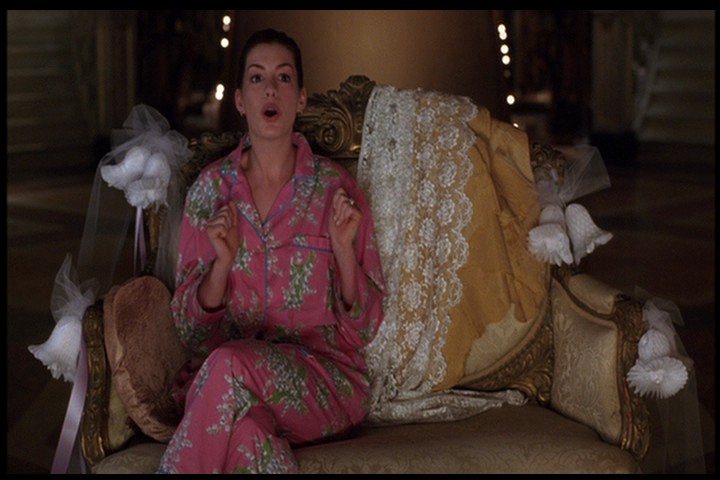

Supplement: S4 Dataset — It also includes pose data and camera parameters. (ZIP) [file pone.0264302.s004.zip › princess-diaries-2-00093181.jpg]

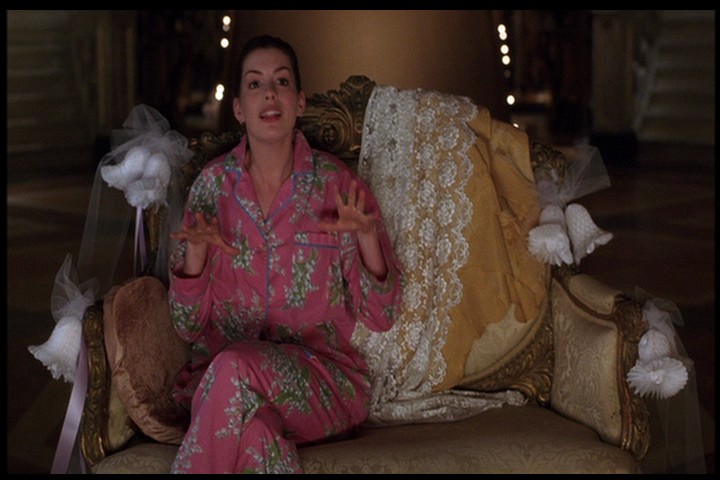

Supplement: S4 Dataset — It also includes pose data and camera parameters. (ZIP) [file pone.0264302.s004.zip › princess-diaries-2-00093191.jpg]

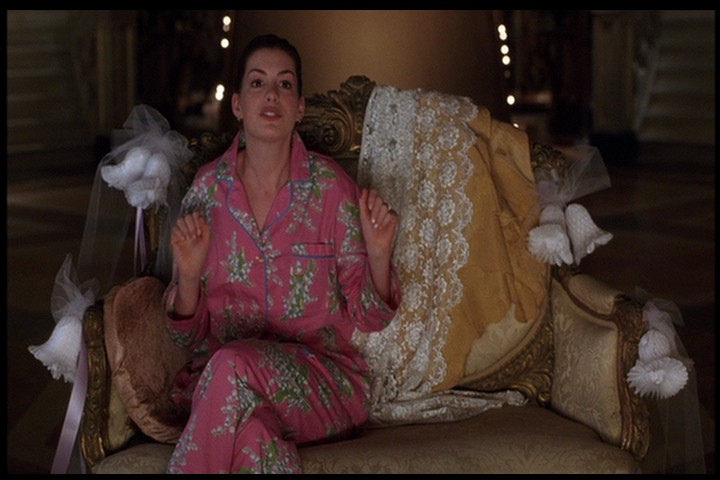

Supplement: S4 Dataset — It also includes pose data and camera parameters. (ZIP) [file pone.0264302.s004.zip › princess-diaries-2-00093201.jpg]

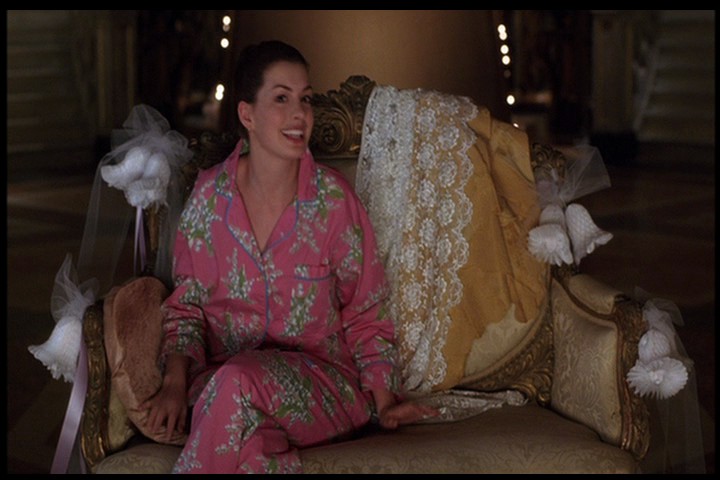

Supplement: S4 Dataset — It also includes pose data and camera parameters. (ZIP) [file pone.0264302.s004.zip › princess-diaries-2-00093211.jpg]

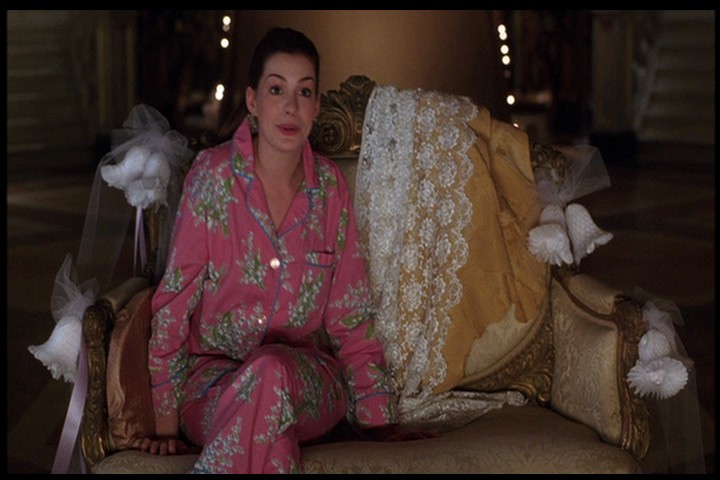

Supplement: S4 Dataset — It also includes pose data and camera parameters. (ZIP) [file pone.0264302.s004.zip › princess-diaries-2-00093231.jpg]

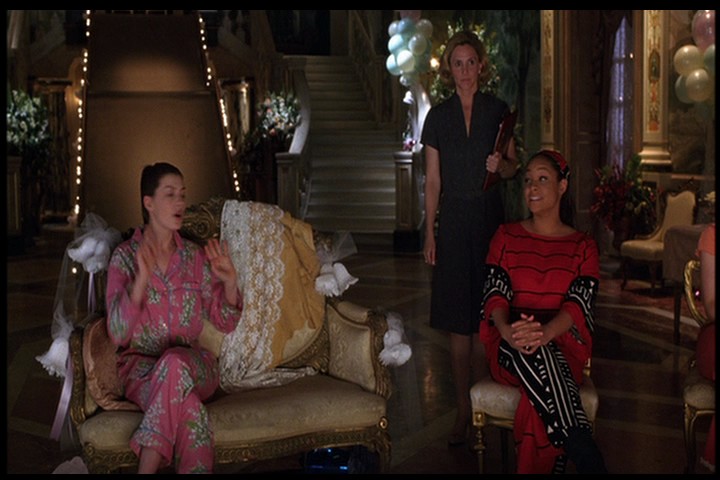

Supplement: S4 Dataset — It also includes pose data and camera parameters. (ZIP) [file pone.0264302.s004.zip › princess-diaries-2-00093301.jpg]

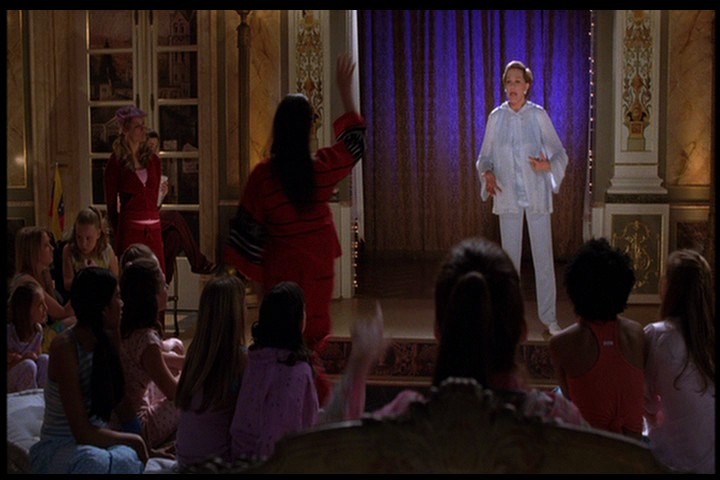

Supplement: S4 Dataset — It also includes pose data and camera parameters. (ZIP) [file pone.0264302.s004.zip › princess-diaries-2-00093381.jpg]

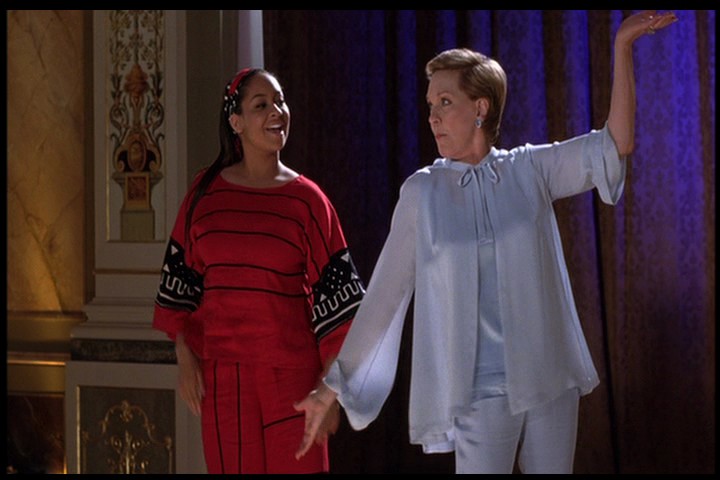

Supplement: S4 Dataset — It also includes pose data and camera parameters. (ZIP) [file pone.0264302.s004.zip › princess-diaries-2-00093481.jpg]

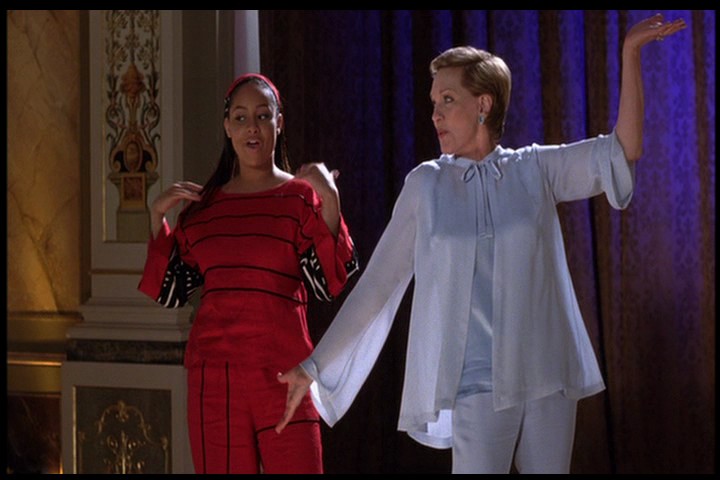

Supplement: S4 Dataset — It also includes pose data and camera parameters. (ZIP) [file pone.0264302.s004.zip › princess-diaries-2-00093491.jpg]

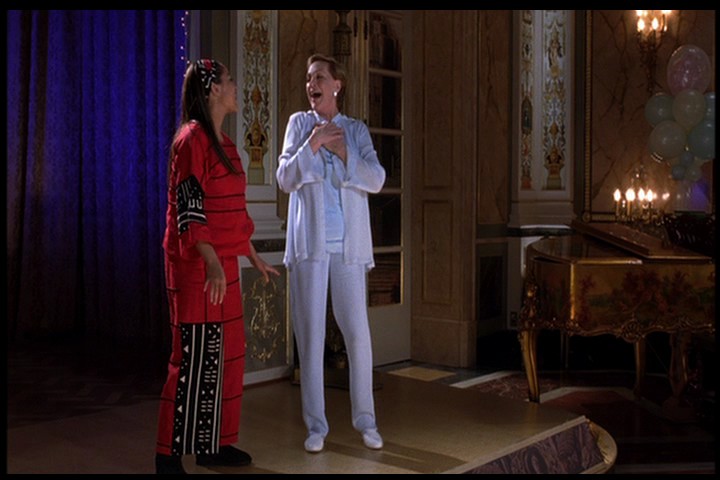

Supplement: S4 Dataset — It also includes pose data and camera parameters. (ZIP) [file pone.0264302.s004.zip › princess-diaries-2-00093721.jpg]

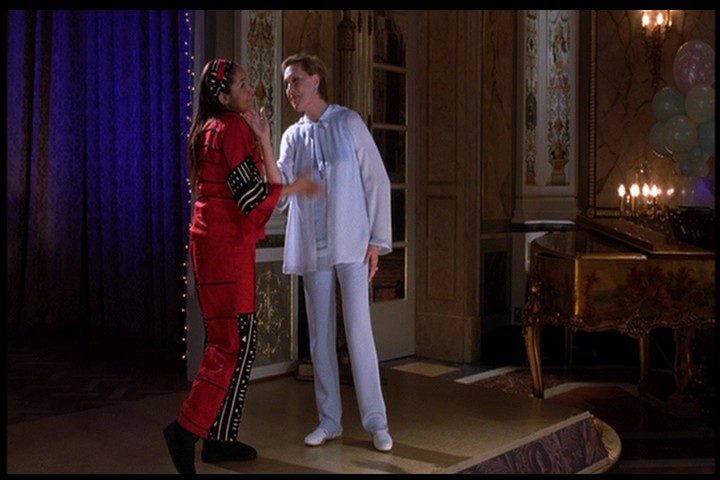

Supplement: S4 Dataset — It also includes pose data and camera parameters. (ZIP) [file pone.0264302.s004.zip › princess-diaries-2-00093791.jpg]

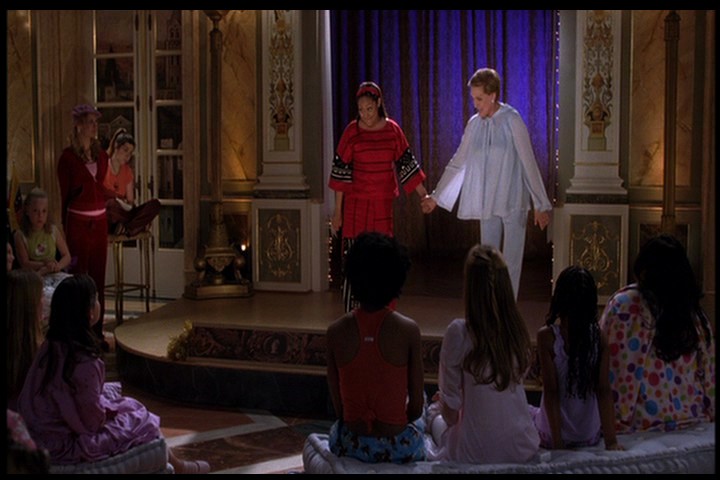

Supplement: S4 Dataset — It also includes pose data and camera parameters. (ZIP) [file pone.0264302.s004.zip › princess-diaries-2-00094271.jpg]

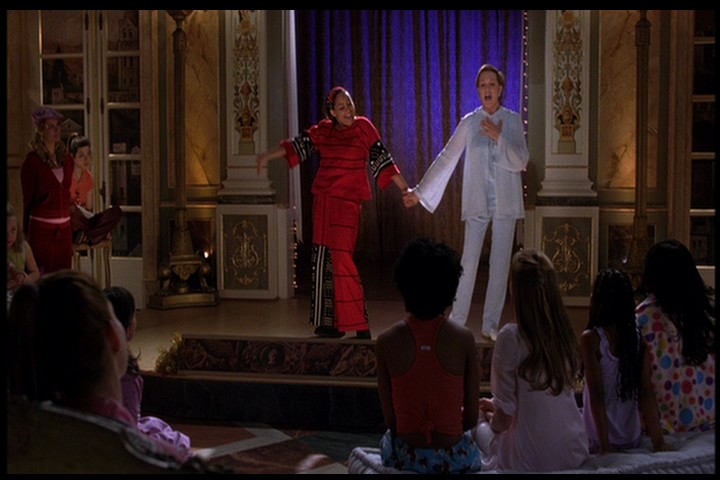

Supplement: S4 Dataset — It also includes pose data and camera parameters. (ZIP) [file pone.0264302.s004.zip › princess-diaries-2-00094441.jpg]

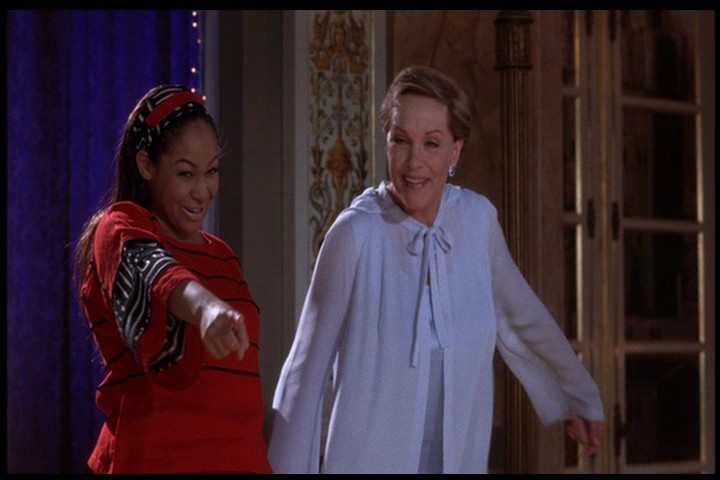

Supplement: S4 Dataset — It also includes pose data and camera parameters. (ZIP) [file pone.0264302.s004.zip › princess-diaries-2-00094531.jpg]

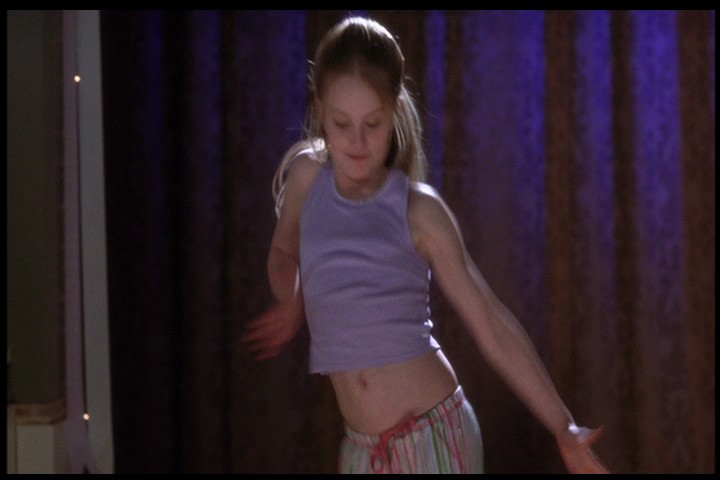

Supplement: S4 Dataset — It also includes pose data and camera parameters. (ZIP) [file pone.0264302.s004.zip › princess-diaries-2-00094721.jpg]

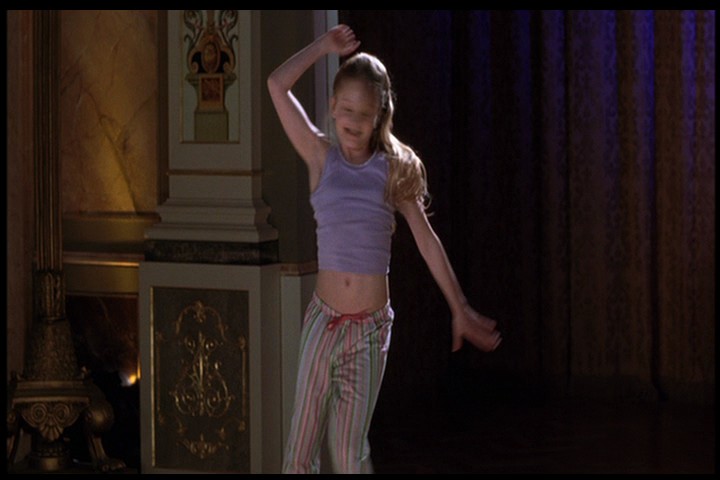

Supplement: S4 Dataset — It also includes pose data and camera parameters. (ZIP) [file pone.0264302.s004.zip › princess-diaries-2-00094761.jpg]

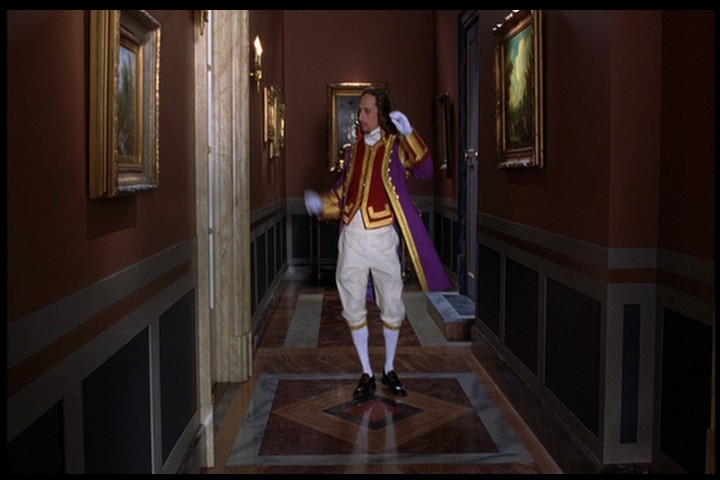

Supplement: S4 Dataset — It also includes pose data and camera parameters. (ZIP) [file pone.0264302.s004.zip › princess-diaries-2-00095161.jpg]

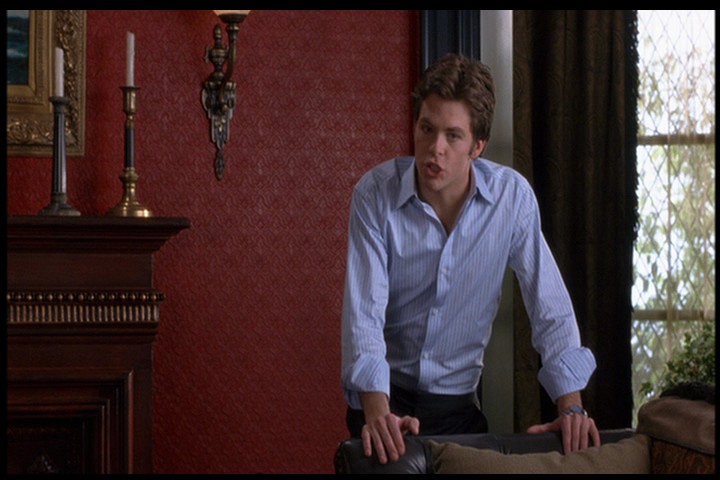

Supplement: S4 Dataset — It also includes pose data and camera parameters. (ZIP) [file pone.0264302.s004.zip › princess-diaries-2-00096191.jpg]

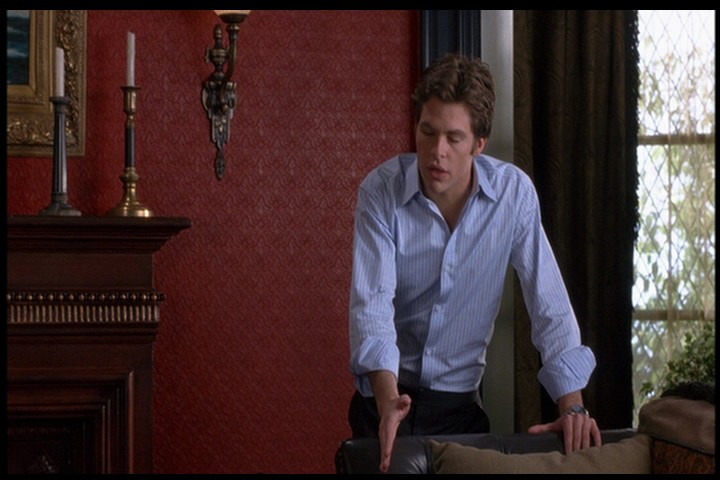

Supplement: S4 Dataset — It also includes pose data and camera parameters. (ZIP) [file pone.0264302.s004.zip › princess-diaries-2-00096211.jpg]

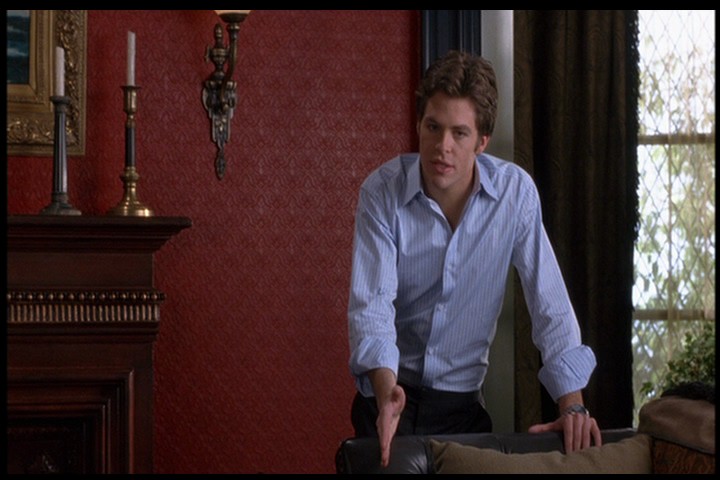

Supplement: S4 Dataset — It also includes pose data and camera parameters. (ZIP) [file pone.0264302.s004.zip › princess-diaries-2-00096221.jpg]

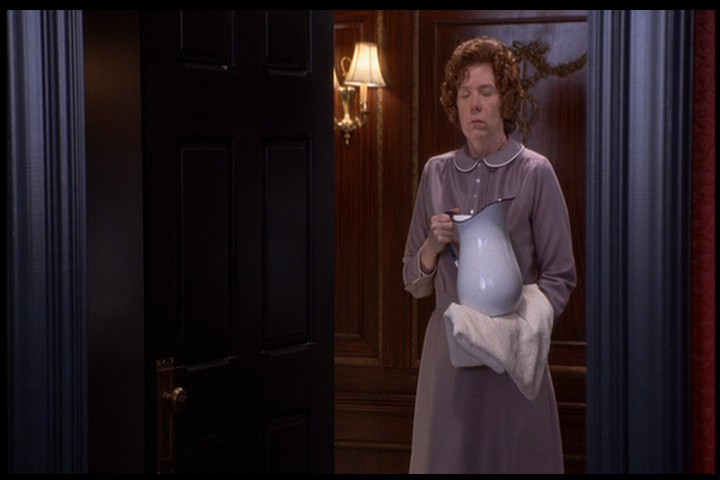

Supplement: S4 Dataset — It also includes pose data and camera parameters. (ZIP) [file pone.0264302.s004.zip › princess-diaries-2-00097001.jpg]

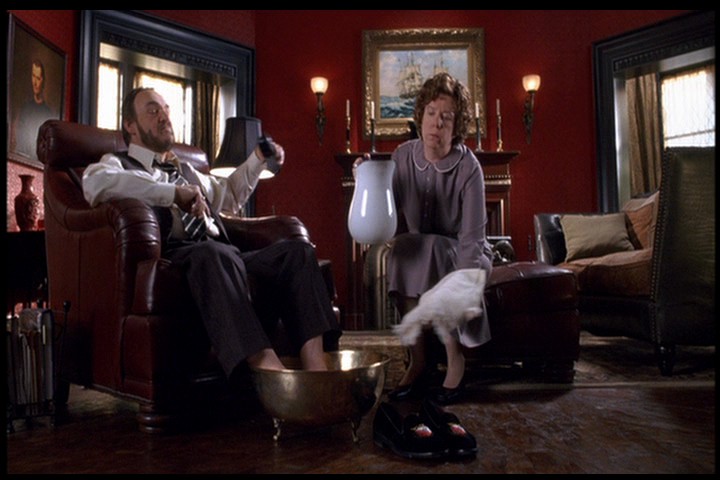

Supplement: S4 Dataset — It also includes pose data and camera parameters. (ZIP) [file pone.0264302.s004.zip › princess-diaries-2-00097841.jpg]

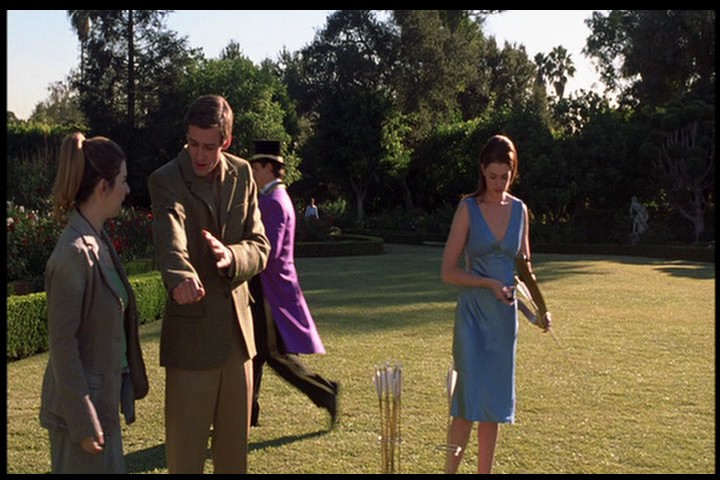

Supplement: S4 Dataset — It also includes pose data and camera parameters. (ZIP) [file pone.0264302.s004.zip › princess-diaries-2-00098421.jpg]

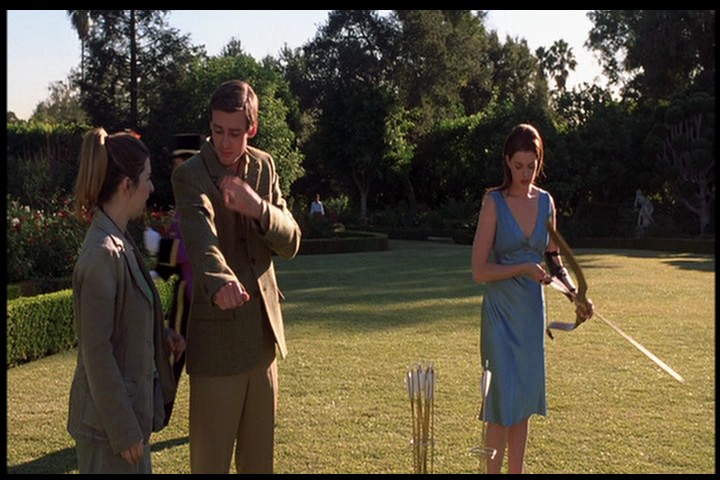

Supplement: S4 Dataset — It also includes pose data and camera parameters. (ZIP) [file pone.0264302.s004.zip › princess-diaries-2-00098431.jpg]

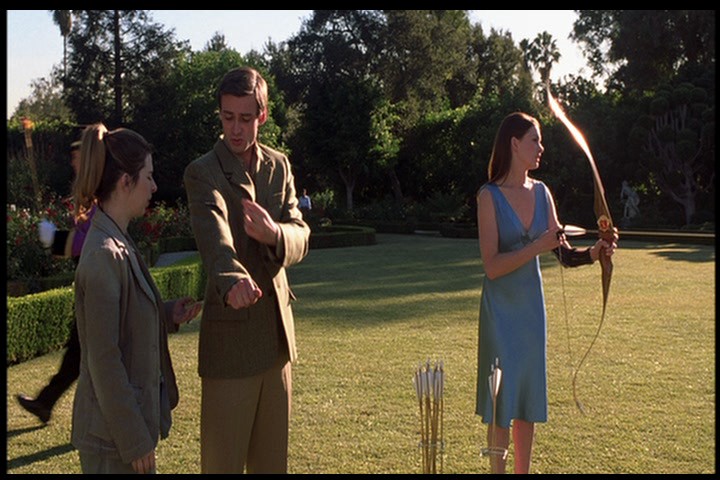

Supplement: S4 Dataset — It also includes pose data and camera parameters. (ZIP) [file pone.0264302.s004.zip › princess-diaries-2-00098441.jpg]
